# Ovarian Circular RNAs Associated with High and Low Fertility in Large White Sows during the Follicular and Luteal Phases of the Estrous Cycle

**DOI:** 10.3390/ani10040696

**Published:** 2020-04-17

**Authors:** Huiyan Hu, Jianzhong Xi, Bo Zhou, Jing Zhang, Zhiqiang Li, Zhongwu Liu, Qing Jia

**Affiliations:** 1 Department of Animal Genetics, Breeding and Reproduction, College of Animal Science and Technology, Hebei Agricultural University, Baoding 071000, China; huhuiyan315@163.com (H.H.); xijianzzz@sohu.com (J.X.); dough@126.com (B.Z.); m15830917592@163.com (J.Z.); luochen0729@163.com (Z.L.); lzw15732855664@163.com (Z.L.); 2 Engineering Research Center for Agriculture in Hebei Mountainous Areas, Baoding 071000, China

**Keywords:** Large White sows, ovarian circular RNAs, fertility, protein-protein network, circRNA–miRNA network

## Abstract

**Simple Summary:**

The fertility of sows is considered to be their most important economic trait, as it is critical for swine farm profitability. There are considerable differences in fertility rates among individual pigs, but the underlying molecular mechanisms remain unclear at present. The ovary is the most important reproductive organ. Folliculogenesis, ovulation, and corpus luteum formation and regression occur in the ovary, repeatedly taking place over the reproductive life and regulating mammalian reproduction. Circular RNAs (circRNAs) represent a novel type of noncoding RNA (ncRNA), which have great potential to exert effects on multiple physiological processes. In particular, some circRNAs are closely involved in reproductive processes and function as potent microRNA (miRNA) sponges to regulate target gene expression levels. In this study, ovarian circular RNAs and miRNAs associated with high and low fertility Large White sows are identified during the follicular and luteal phases of the estrous cycle, and their potential biological functions are predicted through bioinformatic analysis. Protein–protein networks and circRNA–miRNA networks are constructed based on gene expression profiles and the bioinformatic analyses, providing novel insight into fertility regulation in pigs.

**Abstract:**

In this study, the ovarian tissues of Large White pigs were mined for novel circular RNAs (circRNAs), following which, their molecular characteristics and potential mechanisms for fertility regulation were examined. RNA sequencing was used for transcriptome analysis of ovarian follicles and corpora lutea in Large White sows with high (H) and low (L) fertility during the follicular (F) and luteal (L) phases of the estrous cycle. In total, 21,386 circRNA derived from 4535 host genes were identified. Differentially expressed circRNAs were detected in the LH vs. LL (1079) and in the FH vs. FL (1077) comparisons, and their host genes were enriched in steroid biosynthesis and forkhead box O (FOXO), thyroid hormone, cell cycle, and tumor growth factor (TGF)-beta signaling pathways. Protein–protein interaction networks were constructed on the basis of the host genes that were significantly enriched in pathways related to reproductive processes, with AKT3 and PP2CB serving as the hub genes in the networks of the LH vs. LL and FH vs. FL comparisons, respectively. The microRNA (miRNA) binding sites of the differentially expressed circRNAs were predicted, and 128 (LH vs. LL) and 113 (FH vs. FL) circRNA–miRNA pairs were identified. Finally, circRNA–miRNA negative regulatory networks were established on the basis of the gene expression profiles and bioinformatic analyses. In the current study, differentially expressed circRNAs were observed in ovarian tissues between the H and L fertility groups in both F and L phases of the estrous cycle, which suggested roles in pig fertility regulation. These findings provide new clues for elucidating fertility differences in pigs.

## 1. Introduction

Circular RNAs (circRNAs) represent a novel type of noncoding RNA (ncRNA), which are largely derived from back-spliced exonic or intronic sequences and are characterized by covalent closed-loop structures. As their 3′- and 5′-termini are joined together [1], circRNAs are resistant to digestion by RNase R and are exceptionally stable in vivo, compared with their linear counterparts [2]. circRNAs are abundant in the cytoplasm and can be co-expressed with the linear transcripts from which they are derived [3].

In recent decades, genome-wide analyses of RNA-seq data have revealed that circRNAs are widespread in the tissues of humans and other animals [4]. circRNAs have also been commonly observed in tissue-/stage-specific expression patterns [5,6,7]. Three types of circRNAs have been recently recognized: intronic, exon–intron, and exonic [8]. The first two types are found in the nucleus and act as cis-regulatory elements to enhance the expression of their parental genes [9,10]. However, exonic circRNAs are found in the cytoplasm and can function as competing endogenous RNAs (ceRNAs) to regulate the expression levels of the corresponding target genes [11]. For example, circHIPK3 functions as a sponge for miR-124a in human cells [12], and the circRNA chi_circ_0008219 functions as a sponge for multiple microRNAs (miRNAs) [13]. In addition, emerging evidence has indicated that circRNAs can encode proteins [14].

As a novel type of ncRNA, circRNAs have been the focus of extensive exploration in recent years. Evidence has been accumulating that circRNAs have great potential to exert effects on multiple physiological processes [15]. Specifically, some circRNAs are involved in reproductive processes. Numerous circRNAs have been identified from RNA-seq data obtained from Caenorhabditis elegans oocytes [4], Drosophila ovarian tissue [16], and the granulose cells of humans [17]. Cheng et al. (2017) reported that the expression levels of circRNA_103827 and circRNA_104816 in human granulose cells are highly correlated with female reproductive capacity [18]. The potential effects of circRNAs have also been discovered in the pre-ovulatory ovarian follicles of goats [13], and the expression profile of circRNAs in honeybee ovaries during oviposition has also been reported [19]. Collectively, these findings indicate that circRNAs are closely associated with reproductive processes. Zhang et al. (2015) and Huang et al. (2016) prospectively described the transcriptome profiles of the ovary in pigs. Meanwhile, prolificacy-related candidate genes and miRNAs have also been reported [20,21]. However, the potential roles of circRNAs in the regulation of pig fertility are largely unknown.

In the present study, Large White pigs with high (H) and low (L) fertility were selected, and RNA sequencing was conducted using RNA isolated separately from ovarian follicles and corpora lutea in the follicular (F) and luteal (L) phases of the estrous cycle. The aim of this study is to investigate the expression profiles of circRNAs in ovarian tissues and to identify their molecular biological characteristics. The circRNAs involved in fertility regulation are identified, and their potential biological functions are predicted through bioinformatics.

## 2. Materials and Methods

### 2.1. Ethics Statement

The animal experiments were performed in accordance with the guidelines for the care and use of experimental animals established by the Ministry of Science and Technology of the People’s Republic of China (Approval Number 2006-398), and the work was approved by Hebei Agricultural University, Baoding, China.

### 2.2. Sample Preparation

Pig documents of 590 multiparous Canadian Large White cyclic sows were obtained from the Hebei Shunde-Tianzhao Livestock Technology Co., Ltd., Wanquan, Hebei, China. Referring to the methods of Zhang et al. (2015) and Huang et al. (2016) [20,21], the total number of piglets born (TNB) was used to evaluate the fertility of sows in the present study, which was calculated with the SPSS 19.0 statistical software package (IBM Corp, Armonk, NY, USA). The average TNB was 13.42 piglets (standard deviation: 2.75), with the 10% lower tail probability at 11.11 piglets and the 10% upper tail probability at 15.73 piglets per litter. The sows were then divided into two fertility groups: H (TNB > 15.73) and L (TNB < 11.11). The average TNB of the H and L groups were 16.86 and 9.21 piglets, respectively. A total of 16 healthy sows were selected for this study, based on the TNB criterion and with records of consecutive births: the extremely high TNB group (*n* = 8 for the H group) and the extremely low TNB group (*n* = 8 for the L group); see Table 1. Meanwhile, in order to reduce the effects of age (months) and parity on TNB, eight pigs of similar age and parity from each group were selected. The animals were housed under the same environmental conditions, receiving water and diets ad libitum. Given the importance of ovarian activities, ovaries in the L or F phase of the estrous cycle were analyzed for the differences between H and L fertility sows. Four animals from each group were regarded as biological replicates. The animals from post-weaning sows were monitored twice daily for behavioral estrus. On Day 14 and on Day 20 after estrus (Day 1 = first day of estrus), the sows were considered to be in the L and F phase, respectively, according to Soede et al. (2011) [22]. Sows with H and L fertility were sacrificed at each of the two stages: on Day 14 after estrus (*n* = 4 for H fertility sows during the L phase (LH) and *n* = 4 for L fertility sows during the L phase (LL)) and on Day 20 after estrus (*n* = 4 for H fertility sows during the F phase (FH) and *n* = 4 for L fertility sows during the F phase (FL)). The H fertility groups served as controls in this study. Intact ovaries were harvested immediately at the above sacrificial time points and then placed on ice blocks for processing. Ovaries were dissected, and healthy follicles >5 mm [23] and corpora lutea were collected. All tissues were rapidly frozen in liquid nitrogen and stored at −80 °C until RNA extraction.

### 2.3. RNA Isolation and Quality Control

Total RNA samples were isolated from ovarian tissue at different stages using TRIzol reagent (Invitrogen Life Technologies, Carlsbad, CA, USA). The quality of total RNA was determined with an Agilent 2100 Bioanalyzer (Agilent Technologies, Santa Clara, CA, USA). The concentration was assessed by a NanoDrop 2000 (Thermo Fisher Scientific Inc., Waltham, MA, USA), and the integrity was tested by denaturing 1% agarose gel electrophoresis.

### 2.4. Library Construction and circRNA Sequencing

Approximately 3 μg total RNA per sample (four individuals per group) were used for next-generation sequencing library preparation using a NEBNext^®^ Ultra™ Directional RNA Library Prep Kit for Illumina^®^ (New England Biolabs, Ipswich, MA, USA). The ribosomal RNA was depleted from the total RNA using a Ribo-Zero™ rRNA removal kit (Epicentre, Madison, WI, USA). The residual RNA was fragmented and reverse-transcribed. The first-strand cDNA was synthesized using ProtoScript II Reverse Transcriptase (NEB) with random primers and actinomycin D. The second-strand cDNA was synthesized using Second Strand Synthesis Enzyme Mix (including dACG-TP/dUTP; NEB). The cDNA was purified using an AxyPrep Mag PCR Clean-up kit (Axygen, Union City, CA, USA). End-repair of the purified cDNA was performed using End Prep Enzyme Mix (NEB); a poly (A) tail was added to the cDNA, and sequencing adaptors were ligated to both ends. Subsequently, size selection was performed using an AxyPrep Mag PCR Clean-up kit (Axygen). PCR amplification was performed for 11 cycles using P5 and P7 primers, with both primers carrying sequences that annealed with the flow cell to perform bridge PCR and P7 primer carrying a six-base index allowing for multiplexing. The PCR products were cleaned up using an AxyPrep Mag PCR Clean-up kit (Axygen), validated with an Agilent 2100 Bioanalyzer (Agilent Technologies), and quantified using a Qubit 2.0 Fluorometer (Invitrogen). After the quality of the library was evaluated, sequencing was conducted on an Illumina HiSeq X10 instrument (Illumina, San Diego, CA, USA).

### 2.5. Identification of circRNAs and Differential Expression Analysis

After the sequencing data were obtained, the raw data were processed, trimming adaptor sequences and removing low-quality reads with Trimmomatic (v0.30) [24]. High-quality clean reads were obtained and aligned to the reference genome (Sscrofa10.2; downloaded from the Ensembl database using the BWA program (0.7.12) [25]. The alignment results (Sequence Alignment/Map format) were scanned to search for GT/AG splicing signals and paired chiastic clipping (PCC). The circRNAs were detected and identified by the CIRI software (v2.0) [26]. The junction reads at the back-splicing loci of circRNA were normalized using spliced reads per billion mapping (SRPBM) to calculate expression levels [27]. DESeq2 (v1.6.3, released in 2013; Anders and Huber, European Molecular Biology Laboratory (EMBL), Heidelberg, Germany) was used for differential expression analysis. In the comparison of the H and L fertility groups, differential expression was determined by |log_2_(fold change)| ≥ 1 and *p*-value < 0.05. The sequencing data obtained from the RNA-seq were released to the National Center for Biotechnology Information (NCBI) Gene Expression Omnibus (GEO) database under Accession Number GSE134001.

### 2.6. GO and KEGG Pathway Enrichment Analysis

The GO-TermFinder software was used to identify Gene Ontology (GO) terms, which annotated a list of enriched genes with significant *p*-values. The Kyoto Encyclopedia of Genes and Genomes (KEGG) is a collection of databases of genomes, biological pathways, diseases, drugs, and chemical substances. In-house scripts were used to identify KEGG pathways that were significantly enriched with differentially expressed genes. The test was based on the hypergeometric distribution [28,29], with *p* < 0.05 considered as a significantly enriched pathway.

### 2.7. Protein-Protein Interaction Network Analysis

The STRING database was used to identify the protein–protein interaction (PPI) networks; it uses multiple sources of evidence to obtain high-confidence interactions among proteins [30]. In the present study, the host genes of differentially expressed circRNAs, which were significantly enriched in pathways related to reproductive processes, were extracted from the STRING database. Then, the interaction networks were mined for their corresponding proteins, on the basis of multiple sources of information from the STRING database, including text mining, co-expression, experiments, and database evidence. Finally, the PPI networks were established using STRING (v11.0 released in 2019; Szklarczyk et al., Search Tool for the Retrieval of Interacting Genes/Proteins, https://string-db.org/) and visualized by the Cytoscape software (v3.5.1) [31]. Each interaction in a network was derived from the STRING database with a confidence score >0.9 [30].

### 2.8. Small RNA Sequencing Data Analysis

To construct the circRNA–miRNA regulating networks, we used the small RNA sequencing data from the same 16 samples of the four groups (i.e., LH, LL, FH, and FL). This second dataset was also released to NCBI, under Accession Number GSE132307. The differentially expressed miRNAs of each comparison (i.e., LH vs. LL and FH vs. FL) were determined using the Bioconductor package DEseq2 [32], and those with |log_2_(fold change)| ≥ 1 and *p*-value < 0.05 were treated as differentially expressed.

### 2.9. Bioinformatic Analysis and Target Prediction

The miRNA binding sites of differentially expressed circRNAs were predicted with the miRanda software [33]. The differentially expressed miRNAs in the LH vs. LL and FH vs. FL comparisons were regarded as potential circRNA targets. To explore the potential functions of the circRNAs in pig fertility, Cytoscape (v3.5.1) [31] used the circRNA–miRNA negative interactions to construct the circRNA–miRNA networks.

### 2.10. Reverse-Transcription Real-Time Quantitative PCR

To validate the accuracy of the sequencing data, RT-qPCR was conducted. Each group consisted of four samples (the same RNA samples used for sequencing). For the circRNAs, the total RNA was converted into cDNA using a Thermo First Strand cDNA Synthesis Kit (Sinogene, Beijing, China) with the random primer included in the kit. Quantitative PCR was conducted with SYBR Green qPCR Mix (Sinogene, Beijing, China) on a StepOne real-time PCR system (Applied Biosystems, Foster City, CA, USA) under the following conditions: initial denaturation at 95 °C for 10 min, followed by 40 cycles of denaturation at 95 °C for 20 s, annealing at 60 °C for 30 s, and dissociation curve analysis. For the miRNAs, the reverse transcription of total RNA was performed using a One-Step miRNA RT Kit (Sinogene). The relative expression levels of the circRNAs and miRNAs were calculated by the 2^−ΔΔCt^ method [34] using ACTB (XM_021086047.1) and U6 as reference genes, respectively. All RT-qPCR primer sequences used in this study are presented in Table 2. 

### 2.11. Statistical Analyses

All experimental data were analyzed using the SPSS 19.0 statistical software (IBM Corp) and expressed as the mean ± standard deviation (SD). Comparisons between groups were performed using Student’s *t*-tests, and *p* < 0.05 was considered statistically significant.

## 3. Results

### 3.1. Characterization of circRNAs in Ovarian Tissues

**Figure 1:**
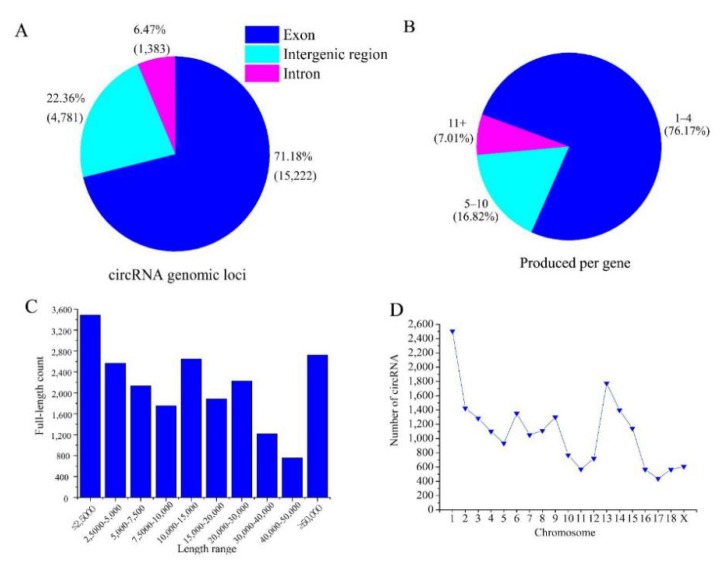
Characterization of circular RNAs in porcine ovarian follicles and corpora lutea: (**A**) classification of the circRNAs expressed in the ovarian tissues based on genomic origins; (**B**) number of circRNAs produced per gene (21,386 circRNAs from 4535 host genes); (**C**) number of circRNAs in different full-length categories; and (**D**) number of circRNAs detected in different pig chromosomes.

In total, 21,386 circRNA were found in the ovarian follicles and corpora lutea of Large White pigs; detailed information is provided in Supplementary Table S1. More than 70% of the circRNAs originated from protein-coding exons, whereas smaller fractions aligned with introns and intergenic regions of known transcripts (Figure 1A). We further investigated the number of circRNAs from their host genes and found that one gene could produce multiple circRNAs (Figure 1B; 21,386 circRNAs derived from 4535 host genes). The most abundant circRNAs had a full length less than 2500 nucleotides (nt) (Figure 1C), but larger ones were also observed. When the numbers were compared in the pig chromosomes, circRNAs were most abundant on chromosome 1 (2506), followed by chromosomes 13 (1774) and 2 (1436); see Figure 1D.

### 3.2. Differential Expression of circRNAs

**Figure 2:**
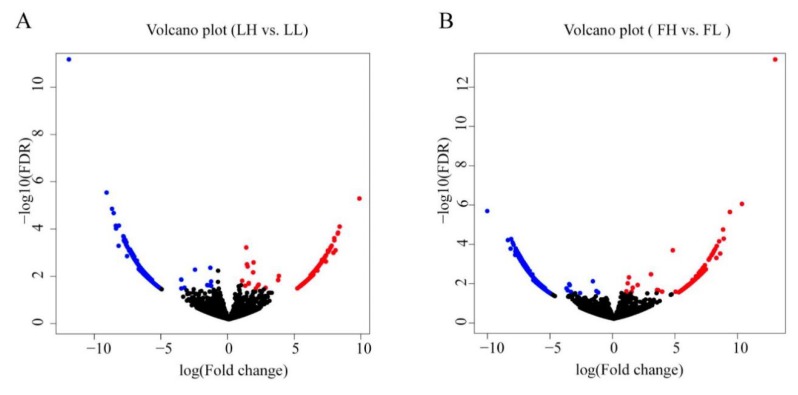
Differentially expressed circRNAs between H and L fertility groups: (**A**) Volcano plot of differentially expressed circRNAs (*p* < 0.05) during the L phase of the estrous cycle (LH vs. LL). The blue (downregulated) and red (upregulated) points represent the differentially expressed circRNAs. (**B**) Volcano plot of differentially expressed circRNAs during the F phase of the estrous cycle (FH vs. FL). The blue (downregulated) and red (upregulated) points represent the differentially expressed circRNAs. FDR, false discovery rate.

**Figure 3:**
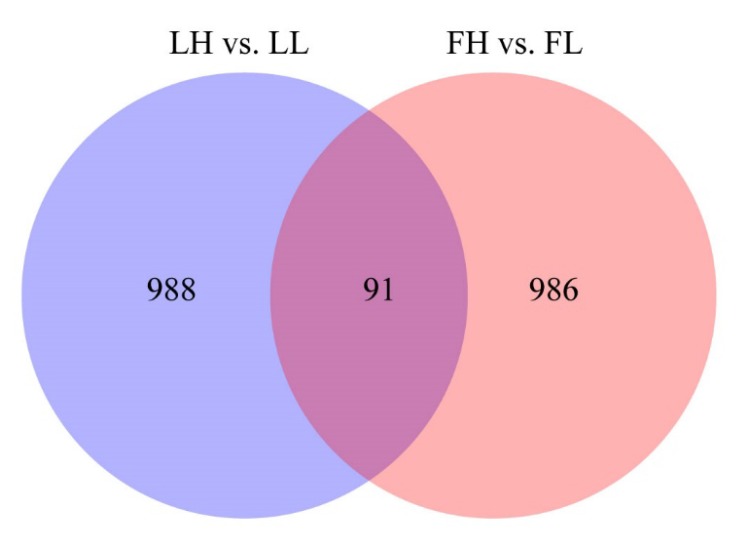
Venn diagram of the differentially expressed circRNAs common to the two comparison groups (LH vs. LL and FH vs. FL).

The expression of circRNAs between the H and L fertility groups was compared, in order to identify the circRNAs that potentially regulate fertility. Based on the differential expression analysis, 1079 circRNAs were differentially expressed (458 upregulated and 621 downregulated) in the LH vs. LL comparison (*p* < 0.05; Figure 2A, Supplementary Table S2-1), and 1077 circRNAs were differentially expressed (347 upregulated and 730 downregulated) in the FH vs. FL comparison (*p* < 0.05; Figure 2B, Supplementary Table S2-2). As shown in Figure 3, 91 differentially expressed circRNAs were common to both comparisons.

### 3.3. GO and KEGG Enrichment of Host Genes

To explore the functions of the differentially expressed circRNAs in ovarian tissues, GO and KEGG enrichment analyses were performed. The GO analysis of the host genes for differentially expressed circRNAs revealed 45 significant GO terms (*p* < 0.05) for the LH vs. LL comparison (Supplementary Table S3-1) and 45 for the FH vs. FL comparison (Supplementary Table S3-2). In the molecular function, cellular components, and biological processes categories, the two groups shared their top enriched GO terms of catalytic activity, organelle part, biological regulation, and reproductive processes, respectively. During the L phase of the estrous cycle, KEGG pathway enrichment analysis indicated that the host genes of the differentially expressed circRNAs were enriched in steroid biosynthesis, the forkhead box O (FOXO) signaling pathway, the thyroid hormone signaling pathway, cell cycle, and insulin resistance (Supplementary Table S3-3). In addition, during the F phase, the most meaningful pathways were related to the tumor growth factor (TGF)-beta signaling pathway, cell cycle, and the Wnt signaling pathway (Supplementary Table S3-4).

### 3.4. Protein–Protein Interaction Networks

**Figure 4:**
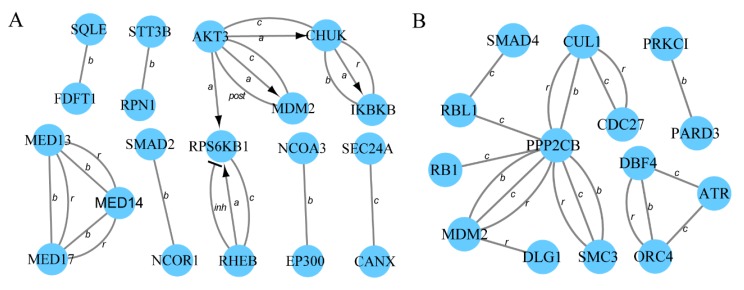
Protein–protein interaction network visualization (made using Cytoscape v3.5.1). The host genes of differentially expressed circRNAs significantly enriched in pathways related to reproductive processes were used to construct the interaction networks. Each interaction in a network is derived from the STRING database with a confidence score > 0.9. (**A**) Interaction network for the LH vs. LL comparison. (**B**) Interaction network for the FH vs. FL comparison. In the networks, nodes represent proteins, and solid lines (known as edges) indicate the action types among the host genes of differentially expressed circRNAs, including activation (a), inhibition (inh), binding (b), catalysis (c), reaction (r), and post-translational modification (post). Arrowed lines represent positive effects, and terminated (T-shaped) lines represent negative effects.

In the LH vs. LL comparison, 36 (Supplementary Table S4-1) host genes of differentially expressed circRNAs were significantly enriched in nine (Supplementary Table S4-2) pathways related to reproduction. In the FH vs. FL comparison, 21 (Supplementary Table S4-3) host genes of differentially expressed circRNAs were significantly enriched in three (Supplementary Table S4-4) pathways related to reproduction. To further investigate the complex relationships among these host genes, the associated PPI networks were constructed using the STRING database and Cytoscape (v3.5.1) software (Figure 4). For the LH vs. LL comparison, 36 proteins were identified, of which 19 were found to interact highly with one or more proteins (confidence score > 0.9; Figure 4A, Supplementary Table S4-5). Based on the results, AKT3 was determined to be at the core of the PPI network. For the FH vs. FL comparison, 14 of 21 proteins directly interacted with at least one of the other proteins (confidence score > 0.9; Figure 4B, Supplementary Table S4-6), which further authenticated their role in reproduction-associated processes.

### 3.5. Prediction of miRNA Binding Sites

**Figure 5:**
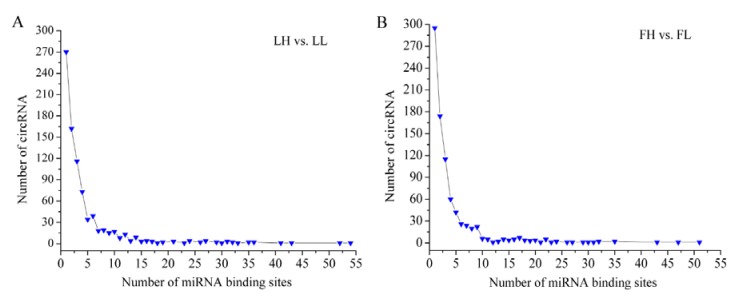
The miRNA binding sites of differentially expressed circRNAs: (**A**) LH vs. LL; and (**B**) FH vs. FL.

circRNAs can function as miRNA sponges [12,13]. In the present study, miRNA binding sites on the differentially expressed circRNAs were predicted using the miRanda software [32]. In the LH vs. LL comparison, 841 of 1079 circRNAs had miRNA binding sites (Figure 5A). In the FH vs. FL comparison, 845 of 1077 circRNAs had miRNA binding sites (Figure 5B). Most circRNAs had one or more miRNA binding sites. Furthermore, one circRNA (circRNA_001214) had 54 miRNA binding sites.

### 3.6. circRNA–miRNA Interaction Networks

For the LH vs. LL comparison, 122 (including 29 known miRNAs) differentially expressed miRNAs were isolated, and for the FH vs. FL comparison, 46 (including 11 known miRNAs) differentially expressed miRNAs were isolated (see Supplementary Table S5-1 and S5-2, respectively). To investigate the potential roles of the differentially expressed circRNAs in fertility regulation, the potential target miRNAs that were differentially expressed were further analyzed. As a result, 128 circRNA–miRNA pairs were identified in the LH vs. LL comparison, and 113 were identified in the FH vs. FL comparison (see Supplementary Table S6-1 and S6-2, respectively).

**Figure 6:**
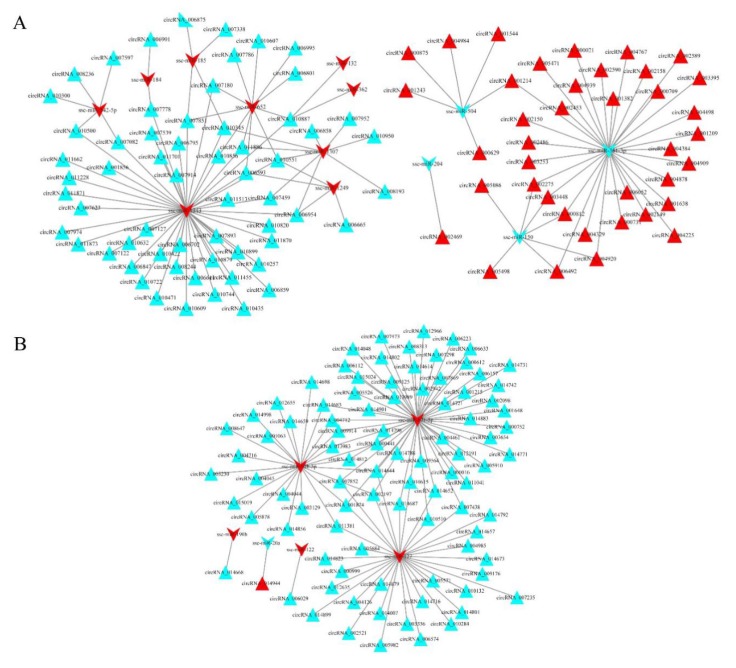
circRNA–miRNA interaction networks: (**A**) for the LH vs. LL comparison; and (**B**) for the FH vs. FL comparison. In the networks, the red and blue triangular nodes denote upregulated and downregulated circRNAs, respectively. The red “V” and blue “V” nodes represent upregulated and downregulated miRNAs, respectively.

The circRNA–miRNA interaction networks are shown in Figure 6. The network of the LH vs. LL comparison is composed of 113 nodes and 128 edges (Figure 6A), where the nodes include 13 miRNAs and 100 circRNAs. Ssc-miR-1343 and ssc-miR-361-3p had the most interactions, implying that they were hub genes in the network. circRNA_010551 had the most interactions with miRNAs (ssc-miR-1343, ssc-miR-652, ssc-miR-1249, and ssc-miR-1307). Notably, ssc-miR-361-3p and ssc-miR-150 were coupled miRNAs, which had several of the same target genes (circRNA_002275, circRNA_003448, circRNA_000812, circRNA_006492, and circRNA_004920), indicating that they might coregulate target genes in the network.

The interaction network for the FH vs. FL comparison was composed of 99 nodes and 113 edges (Figure 6B), consisting of 93 circRNAs and 6 miRNAs. Three (ssc-miR-127, ssc-miR-671-5p, and ssc-miR-92b-5p) of the six miRNAs had more degrees and shared four of the same target genes (circRNA_002197, circRNA_014644, circRNA_007852, and circRNA_001874), demonstrating their roles in the regulation networks.

### 3.7. Validation of RNA-Seq Data Using RT-qPCR

**Figure 7:**
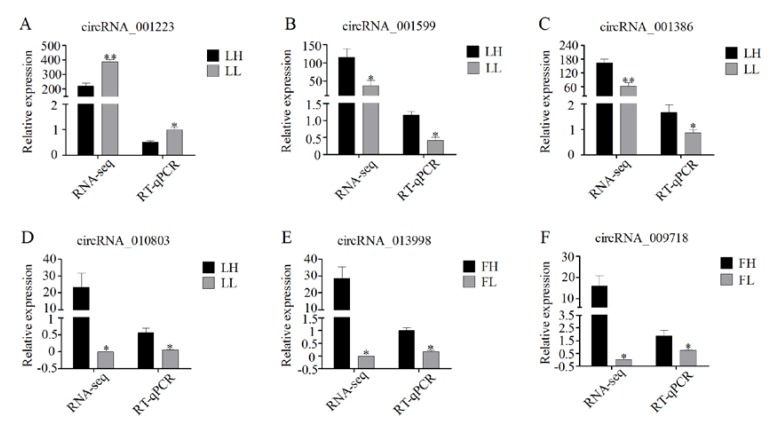
RT-qPCR validation of differentially expressed circRNAs in the H and L fertility Large White sows during the F and L phases of the estrous cycle: (**A–D**) relative expression levels of circRNAs in the LH vs. LL comparison; and (**E,F**) relative expression levels of circRNAs in the FH vs. FL comparison. Data are presented as the mean ± SD of three experiments. Statistical significance was assessed with Student’s *t*-tests. * *p* ≤ 0.05 and ** *p* ≤ 0.01 (compared with the H fertility group).

**Figure 8:**
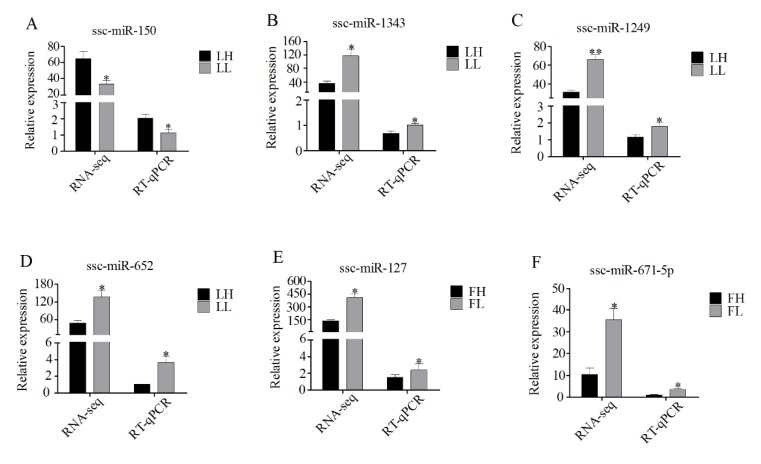
RT-qPCR validations of differentially expressed miRNAs: (**A–D**) relative expression levels of miRNAs in the LH vs. LL comparison; and (**E,F**) relative expression levels of miRNAs in the FH vs. FL comparison. Data are presented as the mean ± SD of three experiments. Statistical significance was assessed by Student’s *t*-tests. * *p* ≤ 0.05 and ** *p* ≤ 0.01 (compared with the H fertility group).

To confirm the accuracy of the RNA-seq data, several circRNAs differentially expressed between the H and L fertility groups were randomly selected for validation. RT-qPCR was conducted to examine the expression levels of the following circRNAs (each group, *n* = 4): LH vs. LL: circRNA_001223, circRNA_001599, circRNA_001386, and circRNA_010803 and FH vs. FL: circRNA_013998, and circRNA_009718. The circRNAs were the same as those used for RNA-seq. The expression patterns of the six circRNAs were highly consistent with those of the RNA-seq data, suggesting that the circRNA profile was reliable (Figure 7). Furthermore, six key miRNAs were used to validate the expression levels from the RNA-seq, with four miRNAs (ssc-miR-150, ssc-miR-1343, ssc-miR-1249, and ssc-miR-652) from the LH vs. LL networks and two miRNAs (ssc-miR-127 and ssc-miR-671-5p) from the FH vs. FL networks. The expression patterns of all six miRNAs were consistent with those of the corresponding RNA-seq data (Figure 8), demonstrating the reliability of the small RNA sequencing data.

## 4. Discussion

The circRNA expression profiles in human ovarian components, including granulose cells [17], cumulus cells [35], and follicular fluid [36], are closely associated with reproductive processes. In this study, the circRNA expression profiles of porcine ovarian follicles and corpora lutea during the estrous cycle were examined. In total, 21,386 distinct circRNA were identified in these samples (*n* = 16). Most of the circRNAs were derived from the exons of protein-coding genes and were less than 2500 nt in length, similar to those observed in the pig spleen [37]. One host gene could generate one or more circRNAs, consistent with a previous report [37]. The PTK2 gene is a typical example, which can produce 47 different circRNA isoforms [38]. In addition, thousands of differentially expressed circRNAs were also identified between the H and L fertility groups in the two different phases. One of the most important new findings was that some differentially expressed circRNAs were common to the two comparisons (LH vs. LL and FH vs. FL), suggesting that they served a vital role in fertility regulation.

GO and KEGG pathway analyses were used to explore the functions of differentially expressed circRNAs. In the LH vs. LL comparison, several vitally important biological processes and pathways that are highly related to ovarian function, such as reproductive processes, steroid biosynthesis, the FOXO and thyroid hormone signaling pathways, and other important pathways, were identified. In the FH vs. FL comparison, the most meaningful pathways were related to the TGF-beta and Wnt signaling pathways. The ovary is an extremely important organ, which is involved in a series of reproductive processes including ovarian folliculogenesis, ovulation, and the formation and regression of the corpora lutea [39,40]. Furthermore, steroid biosynthesis, TGF-beta signaling, and Wnt signaling are critical for follicle growth, oocyte maturation, and ovulation [41,42,43,44]. FOXO signaling is associated with fertility through regulation of ovarian prostaglandins [45]. Thus, the roles of these circRNAs in ovarian tissues may be related to fertility regulation (Table S4-1 and S4-4 ). The action mechanisms of circRNAs were investigated by constructing PPI networks. Several hub genes were identified, such as AKT, RPS6KB1 (Figure 4A), and PPP2CB (Figure 4B). These three genes have important roles in the ovary [46,47,48]. For example, insulin and insulin growth factor 1 (IGF1) have been shown to activate RPS6KB1 and induce proliferation of ovarian theca-interstitial cells [47]. However, RPS6KB1 activation is largely mediated by the kinase AKT [46]. Liu et al. (2006) also demonstrated that AKT could increase mammalian oocyte growth and survival [47]. Recent research has indicated that circ-AKT3, a circRNA found in the clinical glioblastoma (GBM)GBM tissues and paired adjacent normal tissues [49], could encode a novel protein, which is a newly identified negative regulator of the RTK/PI3K pathway [50]. However, further investigation is required to understand the role of circ-AKT3 in pig fertility regulation. Moreover, the gene MDM2 was observed in the two networks (Figure 4). Portela et al. (2015) reported that MDM2 is an antiapoptotic protein that inhibits apoptosis in cattle ovarian granulosa cells [51]. These findings imply that the circRNAs may also participate in regulatory networks. However, the specific molecular mechanisms remain to be elucidated.

Importantly, the circRNAs had abundant miRNA binding sites. In previous studies, cytoplasmic circRNAs have been shown to function as potent miRNA sponges to regulate target gene expression levels [3,13]. A striking example is circHIPK3, which contains multiple miRNA binding sites and combines with miR-124 to inhibit its expression [12]. Consistent with these studies, our results showed that most circRNAs harbored numerous miRNA binding sites (Figure 5A,B). On the basis of bioinformatic analysis, regulatory networks were built for the differentially expressed circRNAs and miRNAs in ovarian tissues between the H and L fertility groups (Figure 6). From the co-expression network (Figure 6A), several host genes of the circRNAs were annotated in the process of reproduction, such as SEC23B (circRNA_011513), NCOR1 (circRNA_006954), and SMAD2 (circRNA_006665). The host gene provides one clue about the function of a circRNA. Previous reports have shown that NCOR1, as a component of a large protein complex, interacts with estrogen receptors, which are essential for normal reproduction and development [52,53]. The host gene of circRNA_006665, SMAD2, is significantly enriched in the FOXO signaling pathway and cell cycle. SMAD2, one of the TGF-β superfamily members, is highly expressed in primordial and early growing follicles. Several studies have demonstrated that SMAD2 is involved in follicle development [44,45]. Furthermore, Xing et al. (2014) suggested that SMAD2 is a key regulator of the follicular development and growth of oocytes in the porcine ovary [54]. As mentioned above, NCOR1 and SMAD2 may be linked (Figure 4A). These results suggest that these circRNAs play a regulatory role in pig fertility. Notably, both circRNA_006954 and circRNA_006665 contained ssc-miR-1249 binding sites and were dramatically downregulated in the LL group. The RT-qPCR results confirmed that ssc-miR-1249 was highly expressed in the LL group (Figure 8), which was consistent with the RNA-seq results. A previous study demonstrated that miR-1249 has important roles in improving the fertility of bull spermatozoa [55]. The expression of miR-1249 has been found to be significantly higher in bulls with moderate fertility compared with a high fertility group, indicating that miR-1249 negatively regulates the expression of protein-coding genes, which leads to problems during reproduction [55]. Remarkably, miR-1249 was located on BTA5, which is a candidate gene associated with reproduction efficiency in cattle [56]. In this network, ssc-miR-1249 may directly target these circRNAs to regulate their expression. However, this finding deserves exploration in future work. 

In addition, circRNA_007852 and circRNA_001874 were derived from the OSBPL1A gene and shared three miRNAs, as shown in Figure 6B. Although these circRNAs were not annotated in the process of reproduction, one of these miRNAs—ssc-miR-127—is functionally important in reproduction [57]. These results suggested that circRNAs regulate fertility in pigs by binding to miRNAs that regulate target gene expression. Moreover, circRNA_001648 is another important circRNA. The host gene of circRNA_001648, CDC14, is involved in the cell cycle. CDC14 is a highly conserved, dual-specificity phosphatase, which is necessary for cytokinesis and mitotic exit in somatic cells [58]. In mouse oocytes, CDC14A expression during female meiosis is critical for healthy egg development [59]. Recently, Imtiaz et al. (2018) reported that CDC14A is essential for male fertility in mice and humans [60]. Thus, circRNA_001648 may function as an ssc-miR-671-5p sponge and play a critical role in the ovary.

## 5. Conclusions

The present study investigated the circRNA transcripts of the ovarian follicles and corpora lutea of Large White sows with high and low fertility in the follicular and luteal phases of the estrous cycle. We found a total of 21,386 circRNA derived from 4535 host genes. Our results contribute to a deeper understanding of the characteristics of ovarian circular RNAs. Subsequently, the differentially expressed circRNAs between high and low fertility sows were identified during follicular and luteal phases. Function enrichment analysis revealed that their host genes were enriched in several reproduction-related signaling pathways, such as steroid biosynthesis and the FOXO, TGF-beta, and Wnt signaling pathways. Moreover, several hub genes were identified by protein–protein interaction network analysis. Based on gene expression profiles and bioinformatic analysis, the constructed circRNA–miRNA networks provided novel insight into fertility regulation. In summary, these findings provided valuable resources for further exploration into new ways to improve fertility in pigs.

## Figures and Tables

**Table 1:** Phenotypic characteristics of Canadian Large White sows used in this study.

Group	Sample ID	Age (Months)	Parities	TNB ^3^	*p* Value	NBA ^4^	Estrous Cycle
LH ^1^	LH1	38	6	17.00 ± 1.45	2.65 × 10^−5^	15.83 ± 0.92	Luteal phase
	LH2	37	6	16.00 ± 1.51		14.67 ± 1.20	
	LH3	33	5	15.50 ± 1.85		14.80 ± 0.82	
	LH4	33	5	16.20 ± 2.01		14.60 ± 1.02	
LL ^1^	LL1	31	4	9.50 ± 1.84		8.75 ± 0.70	
	LL2	30	4	7.33 ± 1.64		7.00 ± 0.34	
	LL3	23	3	9.33 ± 1.92		8.67 ± 0.43	
	LL4	24	3	9.67 ± 2.10		8.67 ± 1.05	
FH ^2^	FH1	29	4	15.75 ± 0.50	5.06 × 10^−5^	14.00 ± 0.72	Follicular phase
	FH2	43	7	16.71 ± 1.39		15.85 ± 1.38	
	FH3	42	7	16.57 ± 1.39		14.57 ± 1.05	
	FH4	30	4	17.00 ± 1.84		15.75 ± 0.86	
FL ^2^	FL1	29	4	6.00 ± 1.84		5.25 ± 0.51	
	FL2	23	3	8.33 ± 2.12		7.67 ± 0.40	
	FL3	23	3	4.67 ± 1.53		4.33 ± 1.60	
	FL4	24	3	8.33 ± 2.12		7.33±0.72	

^1^ LH and LL represent Large White sows with high and low litter sizes, respectively, during the luteal phase of the estrous cycle; ^2^ FH and FL represent pigs with high and low litter sizes, respectively, during the follicular phase. ^3^ TNB: total number of piglets born. ^4^ NBA: number born alive. Values are the mean ± SD.

**Table 2:** Quantitative real-time PCR primer sequences used in this study.

Groups	Gene Name	Primer Sequences (5′-3′)		Amplicon Size (bp)
LH vs. LL	*circRNA_001223*	Forward	TTCTGGAGACATCTCGGAGG	124
		Reverse	TCAGGCGGATCTGTTCTTCT	
	*circRNA_001599*	Forward	GCAGAAATGGCTTCCAATAA	133
		Reverse	CTTAACCTGGGAAGTGGAACC	
	*circRNA_001386*	Forward	CACTTGCTTCCGGAGCTTAG	122
		Reverse	AGCCCTGGGAGTAATTCGA	
	*circRNA_010803*	Forward	GAATCACACACCACAGGCAC	143
		Reverse	GCTTTCCAGGCGATCATAAA	
FH vs. FL	*circRNA_013998*	Forward	TCCTTCCCAAGAATGTGCTC	129
		Reverse	TGATGGTGCTGAGATCTCCA	
	*circRNA_009718*	Forward	TCCTGCAATTGAATTCCGTT	155
		Reverse	TAGGCTCTGGCTTTTTCTCTG	
control	*ACTB*	Forward	GGCATCCTGACCCTCAAGTA	100
		Reverse	CACGCAGCTCGTTGTAGAAG	
control	*U6*		CTCGCTTCGGCAGCACATAT	
LH vs. LL	*ssc-miR-150*		TCTCCCAACCCTTGTACCA	
	*ssc-miR-1343*		ATATCTCCTGGGGCCCGCACTCT	
	*ssc-miR-1249*		ATATACGCCCTTCCCCCCCTT	
	*ssc-miR-652*		ATATACAACCCTAGGAGAGGG	
FH vs. FL	*ssc-miR-127*		TCGGATCCGTCTGAGCTTGG	
	*ssc-miR-671-5p*		AGGAAGCCCTGGAGGGGCTG	

## Supplementary Materials

The following are available online at https://www.mdpi.com/2076-2615/10/4/696/s1: Table S1: Identification of the circRNAs expressed in ovarian tissues; Table S2: Differential expression analysis of circRNAs; Table S3: GO and KEGG pathway analyses of the host genes for differentially expressed circRNAs; Table S4: Protein–protein interaction information; Table S5: Differential expression analysis of miRNAs; Table S6: Identification of circRNA–miRNA pairs.
