# Supplementary material for: Ovarian Circular RNAs Associated with High and Low Fertility in Large White Sows during the Follicular and Luteal Phases of the Estrous Cycle
Source: Animals (Basel). 2020 Apr 17;10(4):696. doi: 10.3390/ani10040696 (PMC7222767; doi:10.3390/ani10040696)
Supplement: Supplementary file 1 [file animals-10-00696-s001.pdf]

Table S1. Identification of the circRNAs expressed in ovarian tissues.

circRNA_ID	Locus	circRNA_ID	Locus	circRNA_ID	Locus	circRNA_ID	Locus
circRNA_006212	5:64083714|64083753	circRNA_014947	8:69661028|69668389	circRNA_000411	11:26295949|26307201	circRNA_001039	16:24967931|24994149
circRNA_005257	GL894918.2:5936|5976	circRNA_013243	6:65898325|65905687	circRNA_006090	3:92528897|92540150	circRNA_004259	4:41064761|41090991
circRNA_009180	18:14756244|14756289	circRNA_013870	18:1579412|1586775	circRNA_000303	10:29321805|29333059	circRNA_010685	4:129623874|129650114
circRNA_015250	1:210274912|210274960	circRNA_010859	8:145151051|145158415	circRNA_010891	9:98637346|98648601	circRNA_008623	11:72371861|72398104
circRNA_016097	4:109137018|109137067	circRNA_004763	7:48313648|48321014	circRNA_020232	6:105317507|105328769	circRNA_019214	8:76057138|76083381
circRNA_020345	GL896530.1:2067|2116	circRNA_007232	15:53792889|53800256	circRNA_003080	13:25498737|25510003	circRNA_007868	6:132124985|132151229
circRNA_017373	GL894918.2:7171|7222	circRNA_002595	1:141982455|141989824	circRNA_001535	3:135140353|135151621	circRNA_007279	15:120964226|120990471
circRNA_017335	9:70493197|70493250	circRNA_020680	14:49996854|50004223	circRNA_013209	5:101632432|101643702	circRNA_007149	14:77561976|77588230
circRNA_020908	2:122392177|122392235	circRNA_015752	16:25460680|25468051	circRNA_020568	12:62424103|62435373	circRNA_014069	13:41597859|41624117
circRNA_016748	12:3329263|3329322	circRNA_010812	7:109814818|109822191	circRNA_010135	X:70652880|70664153	circRNA_004109	3:74752613|74778873
circRNA_018037	GL896437.1:3014|3073	circRNA_013743	1:86947219|86954593	circRNA_020517	10:76245922|76257196	circRNA_014710	5:43354518|43380786
circRNA_019381	1:140776191|140776252	circRNA_012171	X:126562222|126569599	circRNA_000518	12:62435246|62446522	circRNA_003596	15:106836857|106863127
circRNA_018090	1:184071290|184071356	circRNA_016220	6:73234581|73241959	circRNA_015071	1:25518797|25530074	circRNA_010063	GL894502.2:2767|29047
circRNA_017364	GL893678.1:26038|26105	circRNA_017529	10:54210961|54218339	circRNA_002400	MT:2300|13579	circRNA_015259	1:218540056|218566342
circRNA_004303	4:81090189|81090257	circRNA_000794	14:64096632|64104011	circRNA_014151	10:29520111|29531391	circRNA_013626	13:97278057|97304344
circRNA_017928	7:42775070|42775138	circRNA_008862	13:210782621|210790000	circRNA_004759	7:43157539|43168820	circRNA_003063	13:4620378|4646667
circRNA_018103	10:29789070|29789139	circRNA_016533	GL894608.1:9016|16395	circRNA_000463	12:20308389|20319673	circRNA_011350	14:95553577|95579872
circRNA_019580	15:151450851|151450923	circRNA_017408	X:127490700|127498080	circRNA_000797	14:66220252|66231536	circRNA_008786	13:90760724|90787030
circRNA_019661	3:11528044|11528119	circRNA_017725	17:49617273|49624654	circRNA_013110	4:83633287|83644571	circRNA_020693	14:77567920|77594239
circRNA_019985	12:52734833|52734908	circRNA_010362	13:35728481|35735863	circRNA_005071	9:19308688|19319973	circRNA_006114	3:121345252|121371575
circRNA_000876	14:146935108|146935184	circRNA_016770	12:49868097|49875480	circRNA_013413	8:114724143|114735429	circRNA_018329	7:14645264|14671601
circRNA_003736	16:78290142|78290218	circRNA_018990	3:90748108|90755491	circRNA_020474	1:270324830|270336118	circRNA_013442	9:28992224|29018562
circRNA_005912	17:1345662|1345738	circRNA_003734	16:75044687|75052071	circRNA_002651	1:199959801|199971098	circRNA_018924	2:83928361|83954707
circRNA_010819	8:8576353|8576429	circRNA_012020	8:112186509|112193894	circRNA_008649	12:20097144|20108444	circRNA_010394	13:131006932|131033279
circRNA_017524	10:48227182|48227258	circRNA_019369	1:85505386|85512772	circRNA_002927	11:55451269|55462575	circRNA_003907	2:47596217|47622565
circRNA_007457	2:71193761|71193838	circRNA_009666	6:90321035|90328424	circRNA_012699	14:138412943|138424259	circRNA_008658	12:27541665|27568015
circRNA_012152	X:37969700|37969777	circRNA_019671	3:45551530|45558919	circRNA_000287	10:16280794|16292113	circRNA_005626	12:37710316|37736668
circRNA_005827	15:18722783|18722861	circRNA_013818	13:84626419|84633809	circRNA_007787	6:13077795|13089115	circRNA_020242	6:146042987|146069344
circRNA_005307	MT:3040|3119	circRNA_017196	6:118035375|118042767	circRNA_002996	12:36509145|36520467	circRNA_000728	14:7176205|7202575
circRNA_014935	4:134769688|134769768	circRNA_004236	4:16778493|16785890	circRNA_001129	17:59478778|59490101	circRNA_018182	15:104515243|104541616
circRNA_005956	18:48236333|48236414	circRNA_014970	1:293362010|293369407	circRNA_009662	6:88343002|88354325	circRNA_020945	3:33343644|33370024
circRNA_006438	8:145003544|145003625	circRNA_001058	16:37500291|37507694	circRNA_011889	6:85261799|85273122	circRNA_005565	10:58597086|58623477
circRNA_009453	4:45556597|45556678	circRNA_001665	4:141826350|141833754	circRNA_014780	13:31934660|31945984	circRNA_001724	5:61488096|61514489
circRNA_019852	9:114147300|114147381	circRNA_013695	15:97974673|97982078	circRNA_005397	X:143993560|144004888	circRNA_017933	7:61606817|61633213
circRNA_010556	2:5807377|5807460	circRNA_000900	15:34890619|34898026	circRNA_005497	1:218540056|218551385	circRNA_000902	15:36556955|36583358
circRNA_003702	16:44272761|44272845	circRNA_009741	7:37118868|37126277	circRNA_013929	5:87192177|87203507	circRNA_008488	1:283798123|283824543
circRNA_015664	14:122899944|122900030	circRNA_009065	15:150359980|150367390	circRNA_020696	14:81445743|81457074	circRNA_013952	8:75546785|75573210
circRNA_016279	7:27754321|27754407	circRNA_002712	1:249850891|249858302	circRNA_002303	9:143198081|143209415	circRNA_016915	15:118023419|118049849
circRNA_017750	2:29224763|29224849	circRNA_017729	18:4978783|4986195	circRNA_011143	11:55451240|55462575	circRNA_020398	1:107808627|107835060
circRNA_015380	11:21831570|21831657	circRNA_016852	14:64122422|64129840	circRNA_019723	5:38567709|38579044	circRNA_004195	3:120136072|120162510
circRNA_001527	3:128300036|128300125	circRNA_016255	6:125774248|125781670	circRNA_008496	1:297457504|297468840	circRNA_006483	9:78882583|78909023
circRNA_002405	MT:15062|15151	circRNA_012905	2:17755529|17762952	circRNA_000541	13:24055472|24066809	circRNA_002625	1:161745226|161771667
circRNA_002678	1:205508191|205508280	circRNA_014703	4:121746651|121754075	circRNA_005471	1:181055349|181066687	circRNA_005083	9:25250548|25276993
circRNA_012386	10:56118543|56118632	circRNA_003578	15:88315046|88322471	circRNA_010331	12:25981967|25993306	circRNA_013791	1:270342348|270368801
circRNA_016985	18:21512696|21512785	circRNA_011498	16:55484876|55492301	circRNA_012570	13:103721266|103732607	circRNA_008302	1:23262198|23288653
circRNA_018247	3:10993419|10993508	circRNA_012674	14:98659351|98666777	circRNA_000628	13:103784117|103795460	circRNA_006233	5:88132144|88158602
circRNA_018394	MT:9226|9319	circRNA_001965	7:46279250|46286677	circRNA_017612	13:167798432|167809776	circRNA_014743	9:78882583|78909044
circRNA_018332	7:43743727|43743822	circRNA_001898	6:125857136|125864564	circRNA_020921	2:142524037|142535381	circRNA_017330	9:55394479|55420950
circRNA_013588	X:105390087|105390183	circRNA_006765	1:257051731|257059164	circRNA_000849	14:117074459|117085804	circRNA_002235	9:55394479|55420953

circRNA_021027	4:93617446|93617542	circRNA_006725	1:181164975|181172411	circRNA_000219	1:244821954|244833302	circRNA_017024	2:82153520|82179998
circRNA_003992	2:118846356|118846453	circRNA_008181	9:146392829|146400265	circRNA_015131	7:120232943|120244293	circRNA_007840	6:85428674|85455157
circRNA_018290	5:82902344|82902441	circRNA_005950	18:31060264|31067701	circRNA_009977	9:70888223|70899574	circRNA_000409	11:26280706|26307201
circRNA_012032	8:145215369|145215468	circRNA_006571	X:68698170|68705607	circRNA_010633	3:103907854|103919205	circRNA_001720	5:56928608|56955103
circRNA_010097	MT:1496|1597	circRNA_015100	6:136150997|136158436	circRNA_003116	13:43406481|43417833	circRNA_020266	7:78911500|78937998
circRNA_012039	9:7346002|7346103	circRNA_001905	6:134537810|134545250	circRNA_004398	5:3457119|3468473	circRNA_013568	X:28845516|28872018
circRNA_014833	4:34617495|34617596	circRNA_007063	13:144058321|144065761	circRNA_021040	4:139773211|139784570	circRNA_018109	10:50920940|50947448
circRNA_019645	2:125240482|125240584	circRNA_006335	7:57604786|57612229	circRNA_016757	12:20308389|20319752	circRNA_000878	14:148062722|148089231
circRNA_010333	12:34023377|34023480	circRNA_014324	16:78213889|78221332	circRNA_009865	8:89363712|89375078	circRNA_011446	15:124154633|124181153
circRNA_012201	1:32031037|32031140	circRNA_010393	13:126803398|126810842	circRNA_007474	2:83218053|83229420	circRNA_021367	X:60379780|60406307
circRNA_013250	6:82980243|82980346	circRNA_017591	13:50560048|50567492	circRNA_012645	14:52842304|52853674	circRNA_003534	15:62969256|62995786
circRNA_015860	2:5807414|5807517	circRNA_000057	1:60436312|60443757	circRNA_018264	4:55318260|55329633	circRNA_019780	6:149190784|149217315
circRNA_015888	2:73698731|73698834	circRNA_005655	13:29719703|29727150	circRNA_015023	2:52846487|52857861	circRNA_008203	GL894055.2:43525|70058
circRNA_018170	14:134683950|134684054	circRNA_006736	1:202490137|202497584	circRNA_001939	6:156310075|156321453	circRNA_005447	1:132973441|133000011
circRNA_016324	7:120269082|120269187	circRNA_019817	8:34670670|34678117	circRNA_004114	3:81201375|81212753	circRNA_008235	X:12899507|12926080
circRNA_003525	15:55462961|55463067	circRNA_002132	8:118377701|118385149	circRNA_010480	15:86611316|86622694	circRNA_019026	4:51506590|51533165
circRNA_017657	15:3665057|3665163	circRNA_020030	14:30856427|30863875	circRNA_017040	2:107117026|107128405	circRNA_020536	11:56114607|56141183
circRNA_016925	16:20279626|20279733	circRNA_012754	15:111901622|111909071	circRNA_001708	5:38567660|38579044	circRNA_019346	X:70821837|70848414
circRNA_018161	14:71943951|71944059	circRNA_019699	4:68360198|68367651	circRNA_020349	X:14167665|14179050	circRNA_004300	4:79089337|79115918
circRNA_001205	2:1445190|1445300	circRNA_002994	12:32369890|32377346	circRNA_007888	6:148969098|148980488	circRNA_017813	3:130366351|130392936
circRNA_008671	12:39191734|39191844	circRNA_004351	4:122750450|122757907	circRNA_013819	13:84704237|84715627	circRNA_009443	4:39611818|39638405
circRNA_019740	5:87303631|87303741	circRNA_017201	6:132131215|132138673	circRNA_015072	1:85692377|85703772	circRNA_000981	15:105424453|105451047
circRNA_000279	10:11757763|11757874	circRNA_012961	2:108987945|108995405	circRNA_000749	14:21564778|21576174	circRNA_011222	13:50591169|50617766
circRNA_010678	4:93663660|93663771	circRNA_019160	7:57927231|57934691	circRNA_013182	5:76369649|76381045	circRNA_017586	13:29358370|29384973
circRNA_019959	10:21590612|21590724	circRNA_005111	9:44381642|44389104	circRNA_006147	4:55368160|55379557	circRNA_009008	15:59683347|59709969
circRNA_020952	3:43048869|43048981	circRNA_005181	9:132895736|132903198	circRNA_001736	5:70576776|70588176	circRNA_000639	13:117567899|117594523
circRNA_005308	MT:3342|3455	circRNA_021004	4:31389361|31396823	circRNA_011312	14:27785622|27797023	circRNA_001357	2:142522062|142548691
circRNA_015718	15:106597020|106597133	circRNA_002890	11:15977687|15985150	circRNA_017013	2:45059358|45070759	circRNA_003007	12:37398861|37425492
circRNA_016145	5:48589782|48589895	circRNA_013187	5:79942875|79950340	circRNA_012732	15:85110060|85121462	circRNA_017127	4:127136597|127163229
circRNA_021232	8:131120984|131121098	circRNA_002253	9:79874576|79882043	circRNA_012275	1:176846274|176857677	circRNA_003697	16:38531076|38557717
circRNA_018342	8:20747626|20747741	circRNA_013421	8:140732914|140740383	circRNA_003690	16:36914607|36926011	circRNA_000319	10:37017761|37044410
circRNA_005337	X:53124841|53124957	circRNA_020627	13:132241829|132249301	circRNA_007654	4:55368160|55379564	circRNA_007022	13:90923792|90950445
circRNA_014366	2:104279865|104279981	circRNA_017954	8:49952578|49960052	circRNA_021208	8:75758787|75770191	circRNA_002023	7:98982345|99009001
circRNA_013560	MT:5981|6098	circRNA_005075	9:22396993|22404468	circRNA_015910	2:111528891|111540296	circRNA_010174	1:40042621|40069284
circRNA_016355	8:43035559|43035676	circRNA_005706	13:109310069|109317545	circRNA_009358	3:51879967|51891373	circRNA_016492	9:113288178|113314849
circRNA_017351	9:137067130|137067247	circRNA_017772	2:119280884|119288360	circRNA_015432	12:21146891|21158301	circRNA_004647	6:111908180|111934854
circRNA_013877	2:5807398|5807517	circRNA_006739	1:202805859|202813336	circRNA_012858	18:5616649|5628060	circRNA_014101	1:49668384|49695061
circRNA_018688	13:198490204|198490323	circRNA_020769	15:157055344|157062821	circRNA_001241	2:45056046|45067459	circRNA_007657	4:67752436|67779115
circRNA_019787	7:10046989|10047108	circRNA_000080	1:103173037|103180516	circRNA_014570	9:134303800|134315213	circRNA_010937	X:73940741|73967422
circRNA_015945	2:157284602|157284723	circRNA_009937	9:37001749|37009229	circRNA_013320	7:54518702|54530118	circRNA_018295	5:94025566|94052254
circRNA_019624	2:52023284|52023405	circRNA_007318	16:38670029|38677510	circRNA_015982	3:71982481|71993897	circRNA_001738	5:70620860|70647555
circRNA_010441	14:71986097|71986219	circRNA_010099	MT:2300|9782	circRNA_013936	6:99536269|99547687	circRNA_012838	17:31763922|31790621
circRNA_018842	17:16315807|16315929	circRNA_010949	1:13625476|13632958	circRNA_007143	14:65749789|65761210	circRNA_005208	9:149676091|149702797
circRNA_010487	15:97057107|97057230	circRNA_013437	9:12135530|12143013	circRNA_000976	15:101657780|101669202	circRNA_001501	3:114349157|114375867
circRNA_006402	8:45785031|45785156	circRNA_002536	1:93269648|93277132	circRNA_009618	6:23827113|23838537	circRNA_010521	17:20681839|20708552
circRNA_015278	1:231014121|231014246	circRNA_006890	11:52209155|52216641	circRNA_017183	6:91829524|91840949	circRNA_020309	9:29427479|29454192
circRNA_017296	8:135157764|135157889	circRNA_010086	GL896504.1:47972|55460	circRNA_013144	5:2679469|2690899	circRNA_000426	11:70152479|70179198
circRNA_019078	5:73984918|73985043	circRNA_016669	1:201668388|201675876	circRNA_010335	12:37174265|37185696	circRNA_000605	13:81190634|81217362
circRNA_001542	4:2426289|2426415	circRNA_012409	11:536233|543722	circRNA_011767	4:45157523|45168955	circRNA_004488	5:88193297|88220026
circRNA_012425	11:12827206|12827332	circRNA_011785	4:107280131|107287623	circRNA_004357	4:128228734|128240169	circRNA_007347	17:28241701|28268431
circRNA_017392	X:57086047|57086173	circRNA_012805	16:38550225|38557717	circRNA_004581	6:79809424|79820861	circRNA_018830	16:42292392|42319123
circRNA_005825	14:153759423|153759551	circRNA_001224	2:17228897|17236390	circRNA_006558	X:19232020|19243457	circRNA_010881	9:63116301|63143034

<table>
<tr><td>circRNA_011057</td><td>1:265258086|265258214</td><td>circRNA_005281</td><td>GL896504.1:47967|55460</td><td>circRNA_012228</td><td>1:101178705|101190143</td><td>circRNA_004793</td><td>7:68179475|68206209</td></tr>
<tr><td>circRNA_019403</td><td>1:231014118|231014246</td><td>circRNA_006308</td><td>6:147179715|147187208</td><td>circRNA_010023</td><td>9:140007511|140018950</td><td>circRNA_002877</td><td>11:7796074|7822810</td></tr>
<tr><td>circRNA_000591</td><td>13:58571575|58571704</td><td>circRNA_011429</td><td>15:101520858|101528351</td><td>circRNA_019806</td><td>7:102326380|102337819</td><td>circRNA_016433</td><td>9:19293237|19319973</td></tr>
<tr><td>circRNA_008583</td><td>11:12827206|12827335</td><td>circRNA_013005</td><td>3:47969752|47977246</td><td>circRNA_004634</td><td>6:101802150|101813590</td><td>circRNA_003503</td><td>15:22560247|22586988</td></tr>
<tr><td>circRNA_017273</td><td>8:47684335|47684464</td><td>circRNA_013469</td><td>9:79212011|79219505</td><td>circRNA_008054</td><td>8:124317943|124329383</td><td>circRNA_018505</td><td>1:245209387|245236128</td></tr>
<tr><td>circRNA_009367</td><td>3:62273318|62273448</td><td>circRNA_016817</td><td>13:131230388|131237882</td><td>circRNA_007238</td><td>15:61774735|61786177</td><td>circRNA_010669</td><td>4:67866336|67893087</td></tr>
<tr><td>circRNA_010454</td><td>14:120033131|120033261</td><td>circRNA_006784</td><td>1:284315283|284322778</td><td>circRNA_012743</td><td>15:97937425|97948869</td><td>circRNA_019278</td><td>9:81468461|81495224</td></tr>
<tr><td>circRNA_016187</td><td>6:905731|905861</td><td>circRNA_008825</td><td>13:144274234|144281734</td><td>circRNA_012491</td><td>12:40351351|40362796</td><td>circRNA_019006</td><td>3:121657951|121684717</td></tr>
<tr><td>circRNA_019200</td><td>8:32346845|32346975</td><td>circRNA_018588</td><td>12:13573401|13580901</td><td>circRNA_015033</td><td>3:114476160|114487607</td><td>circRNA_000844</td><td>14:115712680|115739454</td></tr>
<tr><td>circRNA_009078</td><td>16:19294496|19294627</td><td>circRNA_006804</td><td>10:11887317|11894819</td><td>circRNA_003064</td><td>13:8006226|8017674</td><td>circRNA_011991</td><td>8:20720967|20747741</td></tr>
<tr><td>circRNA_011894</td><td>6:91873462|91873593</td><td>circRNA_006800</td><td>10:137102|144607</td><td>circRNA_011827</td><td>5:70617986|70629435</td><td>circRNA_014625</td><td>11:486920|513694</td></tr>
<tr><td>circRNA_016957</td><td>17:33086104|33086237</td><td>circRNA_013917</td><td>4:116017070|116024577</td><td>circRNA_017806</td><td>3:114476157|114487607</td><td>circRNA_005999</td><td>2:82538960|82565744</td></tr>
<tr><td>circRNA_001216</td><td>2:5808018|5808152</td><td>circRNA_010990</td><td>1:125491718|125499226</td><td>circRNA_002792</td><td>10:29339600|29351054</td><td>circRNA_002520</td><td>1:74443452|74470252</td></tr>
<tr><td>circRNA_008570</td><td>10:76305977|76306111</td><td>circRNA_005022</td><td>8:141457586|141465095</td><td>circRNA_017860</td><td>5:50061586|50073045</td><td>circRNA_014746</td><td>9:100572778|100599583</td></tr>
<tr><td>circRNA_013670</td><td>12:22280097|22280231</td><td>circRNA_003047</td><td>12:52161469|52168979</td><td>circRNA_014430</td><td>4:75094374|75105835</td><td>circRNA_000061</td><td>1:63702370|63729178</td></tr>
<tr><td>circRNA_018084</td><td>1:127997793|127997927</td><td>circRNA_008523</td><td>10:33050271|33057782</td><td>circRNA_016495</td><td>9:115682682|115694143</td><td>circRNA_007137</td><td>14:60727790|60754598</td></tr>
<tr><td>circRNA_005902</td><td>16:54726827|54726962</td><td>circRNA_011882</td><td>6:76824404|76831918</td><td>circRNA_016992</td><td>18:47246849|47258311</td><td>circRNA_016956</td><td>17:30927979|30954795</td></tr>
<tr><td>circRNA_007327</td><td>16:54880272|54880407</td><td>circRNA_013118</td><td>4:93621825|93629339</td><td>circRNA_012322</td><td>1:283964284|283975748</td><td>circRNA_013669</td><td>12:17059096|17085916</td></tr>
<tr><td>circRNA_017559</td><td>12:14554938|14555074</td><td>circRNA_014295</td><td>15:89632723|89640237</td><td>circRNA_009614</td><td>6:16298300|16309767</td><td>circRNA_000552</td><td>13:33157395|33184216</td></tr>
<tr><td>circRNA_017882</td><td>6:50816128|50816264</td><td>circRNA_016503</td><td>9:138374829|138382345</td><td>circRNA_017198</td><td>6:124817728|124829198</td><td>circRNA_000401</td><td>11:21787387|21814210</td></tr>
<tr><td>circRNA_017625</td><td>14:33473737|33473875</td><td>circRNA_009210</td><td>18:60606054|60613571</td><td>circRNA_007962</td><td>7:119678549|119690020</td><td>circRNA_003275</td><td>13:211128404|211155228</td></tr>
<tr><td>circRNA_018331</td><td>7:29526790|29526928</td><td>circRNA_013709</td><td>3:45690728|45698245</td><td>circRNA_004108</td><td>3:74752613|74764088</td><td>circRNA_017132</td><td>4:136390669|136417498</td></tr>
<tr><td>circRNA_019602</td><td>17:61174085|61174224</td><td>circRNA_008642</td><td>12:16046492|16054013</td><td>circRNA_012977</td><td>2:142541102|142552577</td><td>circRNA_016345</td><td>8:29175726|29202562</td></tr>
<tr><td>circRNA_012551</td><td>13:75597855|75597995</td><td>circRNA_003400</td><td>14:82403576|82411103</td><td>circRNA_016302</td><td>7:73317228|73328704</td><td>circRNA_011845</td><td>5:94418719|94445562</td></tr>
<tr><td>circRNA_016674</td><td>1:205484419|205484559</td><td>circRNA_015842</td><td>18:44788959|44796486</td><td>circRNA_005377</td><td>X:121797075|121808553</td><td>circRNA_015014</td><td>17:55235758|55262601</td></tr>
<tr><td>circRNA_019913</td><td>1:84068387|84068527</td><td>circRNA_016052</td><td>4:17069575|17077103</td><td>circRNA_017835</td><td>4:84307833|84319316</td><td>circRNA_015508</td><td>13:49853429|49880272</td></tr>
<tr><td>circRNA_002665</td><td>1:202616050|202616191</td><td>circRNA_016013</td><td>3:109451708|109459237</td><td>circRNA_007612</td><td>3:122092308|122103795</td><td>circRNA_018444</td><td>1:132969136|132995990</td></tr>
<tr><td>circRNA_010353</td><td>13:17268015|17268157</td><td>circRNA_017159</td><td>6:836074|843606</td><td>circRNA_010124</td><td>X:61023114|61034602</td><td>circRNA_004158</td><td>3:104182825|104209684</td></tr>
<tr><td>circRNA_011462</td><td>16:6865245|6865387</td><td>circRNA_007378</td><td>18:5609233|5616766</td><td>circRNA_015181</td><td>1:86322010|86333500</td><td>circRNA_020043</td><td>14:95622297|95649157</td></tr>
<tr><td>circRNA_019945</td><td>1:269001117|269001260</td><td>circRNA_016791</td><td>13:44098037|44105570</td><td>circRNA_006561</td><td>X:43137995|43149487</td><td>circRNA_008728</td><td>13:33722489|33749350</td></tr>
<tr><td>circRNA_020403</td><td>1:117662822|117662965</td><td>circRNA_020997</td><td>3:135190695|135198230</td><td>circRNA_011905</td><td>6:127528135|127539627</td><td>circRNA_016696</td><td>1:270449484|270476354</td></tr>
<tr><td>circRNA_010513</td><td>16:75277503|75277647</td><td>circRNA_003979</td><td>2:98275617|98283156</td><td>circRNA_020886</td><td>2:81112457|81123949</td><td>circRNA_008865</td><td>13:216669717|216696594</td></tr>
<tr><td>circRNA_019706</td><td>4:107061693|107061837</td><td>circRNA_002740</td><td>1:276557775|276565315</td><td>circRNA_006824</td><td>10:32008346|32019842</td><td>circRNA_017474</td><td>1:181041196|181068076</td></tr>
<tr><td>circRNA_009729</td><td>7:20662798|20662943</td><td>circRNA_018466</td><td>1:176389138|176396678</td><td>circRNA_009632</td><td>6:48344625|48356123</td><td>circRNA_017405</td><td>X:116389266|116416155</td></tr>
<tr><td>circRNA_015631</td><td>14:72355782|72355927</td><td>circRNA_013355</td><td>7:120175105|120182646</td><td>circRNA_016613</td><td>X:142762776|142774274</td><td>circRNA_011176</td><td>12:38757575|38784472</td></tr>
<tr><td>circRNA_005407</td><td>1:18615841|18615988</td><td>circRNA_001078</td><td>16:81590815|81598359</td><td>circRNA_020808</td><td>17:30273315|30284814</td><td>circRNA_009676</td><td>6:99216722|99243627</td></tr>
<tr><td>circRNA_015311</td><td>1:294080186|294080333</td><td>circRNA_011757</td><td>4:37099017|37106561</td><td>circRNA_020085</td><td>16:86793699|86805207</td><td>circRNA_005535</td><td>10:11033710|11060621</td></tr>
<tr><td>circRNA_009346</td><td>3:41663907|41664055</td><td>circRNA_016977</td><td>18:11518468|11526015</td><td>circRNA_000802</td><td>14:77561976|77573487</td><td>circRNA_003417</td><td>14:104732283|104759197</td></tr>
<tr><td>circRNA_010547</td><td>18:40306215|40306364</td><td>circRNA_008490</td><td>1:283916128|283923677</td><td>circRNA_007105</td><td>14:30050907|30062418</td><td>circRNA_002439</td><td>X:121797075|121823999</td></tr>
<tr><td>circRNA_015493</td><td>13:35653154|35653303</td><td>circRNA_003720</td><td>16:51795728|51803278</td><td>circRNA_018381</td><td>GL892397.1:141798|153309</td><td>circRNA_013755</td><td>12:11456322|11483250</td></tr>
<tr><td>circRNA_021186</td><td>8:8778414|8778564</td><td>circRNA_004267</td><td>4:45167066|45174616</td><td>circRNA_003385</td><td>14:78014861|78026373</td><td>circRNA_012372</td><td>10:49568556|49595487</td></tr>
<tr><td>circRNA_021276</td><td>9:85856790|85856941</td><td>circRNA_008224</td><td>GL896381.1:8217|15767</td><td>circRNA_009705</td><td>6:145916377|145927889</td><td>circRNA_007957</td><td>7:109722597|109749551</td></tr>
<tr><td>circRNA_000808</td><td>14:77896328|77896480</td><td>circRNA_016844</td><td>14:43947193|43954743</td><td>circRNA_012693</td><td>14:127943716|127955228</td><td>circRNA_016035</td><td>3:128253444|128280401</td></tr>
<tr><td>circRNA_009364</td><td>3:60664159|60664311</td><td>circRNA_001960</td><td>7:35362834|35370390</td><td>circRNA_013886</td><td>2:108987945|108999458</td><td>circRNA_005853</td><td>15:94283009|94309970</td></tr>
<tr><td>circRNA_010619</td><td>3:55619049|55619201</td><td>circRNA_001699</td><td>5:33828511|33836070</td><td>circRNA_001621</td><td>4:104224060|104235575</td><td>circRNA_012953</td><td>2:90131799|90158760</td></tr>
<tr><td>circRNA_016961</td><td>17:46544919|46545071</td><td>circRNA_005723</td><td>13:142933751|142941310</td><td>circRNA_004329</td><td>4:104224057|104235575</td><td>circRNA_004196</td><td>3:120260142|120287104</td></tr>
<tr><td>circRNA_019980</td><td>12:35313709|35313861</td><td>circRNA_008631</td><td>12:4630206|4637765</td><td>circRNA_012139</td><td>GL896549.1:11995|23514</td><td>circRNA_012881</td><td>18:44432877|44459840</td></tr>
<tr><td>circRNA_018215</td><td>2:3109700|3109853</td><td>circRNA_016487</td><td>9:101353554|101361113</td><td>circRNA_017445</td><td>1:101856740|101868259</td><td>circRNA_015204</td><td>1:123266412|123293376</td></tr>
<tr><td>circRNA_008569</td><td>10:72992488|72992642</td><td>circRNA_019591</td><td>16:51837857|51845416</td><td>circRNA_016927</td><td>16:22536691|22548212</td><td>circRNA_018472</td><td>1:181145447|181172411</td></tr>
<tr><td>circRNA_010839</td><td>8:78377694|78377848</td><td>circRNA_017787</td><td>3:34697222|34704782</td><td>circRNA_004596</td><td>6:85419249|85430771</td><td>circRNA_013524</td><td>GL893957.1:794|27759</td></tr>
<tr><td>circRNA_016094</td><td>4:107484284|107484439</td><td>circRNA_018608</td><td>12:45358111|45365671</td><td>circRNA_017785</td><td>3:26967939|26979461</td><td>circRNA_001014</td><td>15:124693193|124720160</td></tr>
<tr><td>circRNA_016931</td><td>16:25574906|25575062</td><td>circRNA_014299</td><td>15:112469260|112476823</td><td>circRNA_005057</td><td>9:11740106|11751629</td><td>circRNA_021019</td><td>4:73641913|73668882</td></tr>
</table>

circRNA_002404	MT:14866|15023	circRNA_005886	16:23448740|23456305	circRNA_000935	15:71715508|71727032	circRNA_004983	8:103111572|103138543
circRNA_003594	15:105807527|105807684	circRNA_015607	14:48218251|48225819	circRNA_011601	2:76948304|76959829	circRNA_010902	9:133364153|133391130
circRNA_004678	6:143689203|143689360	circRNA_016933	16:27101321|27108891	circRNA_016604	X:113343809|113355334	circRNA_019123	6:118026497|118053482
circRNA_011105	10:44193604|44193761	circRNA_007107	14:31890499|31898074	circRNA_016104	4:117945775|117957301	circRNA_008665	12:37358205|37385204
circRNA_014927	2:5807333|5807491	circRNA_010404	13:156006479|156014054	circRNA_005150	9:102582514|102594047	circRNA_010511	16:65140674|65167681
circRNA_016627	1:40240290|40240448	circRNA_009330	3:16572731|16580308	circRNA_007328	16:55484876|55496410	circRNA_011503	16:65140674|65167684
circRNA_011097	10:29569283|29569442	circRNA_005700	13:97307290|97314870	circRNA_020919	2:140760854|140772388	circRNA_004869	8:413584|440597
circRNA_014094	1:23262198|23262358	circRNA_011879	6:72115133|72122713	circRNA_001515	3:122008499|122020038	circRNA_000919	15:59970423|59997451
circRNA_010586	2:109012727|109012889	circRNA_015758	16:36889845|36897425	circRNA_007357	17:43054927|43066466	circRNA_017027	2:85460738|85487777
circRNA_014063	GL895967.2:567|729	circRNA_017096	4:19845213|19852793	circRNA_012812	16:49039795|49051336	circRNA_011949	7:61582111|61609166
circRNA_014513	7:128613568|128613730	circRNA_013904	3:109890287|109897869	circRNA_020664	14:935394|946936	circRNA_003327	14:31377807|31404870
circRNA_015624	14:65053003|65053165	circRNA_018476	1:184001051|184008633	circRNA_015394	11:26425715|26437258	circRNA_006626	1:40737383|40764451
circRNA_018368	9:32270995|32271157	circRNA_009815	7:130875508|130883093	circRNA_014801	15:117901943|117913489	circRNA_015917	2:125045156|125072226
circRNA_019655	2:142533463|142533625	circRNA_007546	3:33370869|33378455	circRNA_016570	X:3677199|3688745	circRNA_018152	14:34675990|34703061
circRNA_017934	7:62090955|62091118	circRNA_012673	14:98634790|98642376	circRNA_020243	6:148108975|148120523	circRNA_004862	7:128933811|128960887
circRNA_010546	18:33748584|33748748	circRNA_015780	16:65133423|65141009	circRNA_003755	17:24573209|24584759	circRNA_019986	12:54446133|54473216
circRNA_010946	X:114558018|114558182	circRNA_019720	5:32056661|32064247	circRNA_012205	1:35449666|35461217	circRNA_003844	18:31040615|31067701
circRNA_012064	9:49493418|49493582	circRNA_018131	13:99498246|99505833	circRNA_019927	1:150198978|150210530	circRNA_008611	11:53956652|53983742
circRNA_015730	15:127627862|127628026	circRNA_016378	8:76848829|76856418	circRNA_001352	2:140760854|140772407	circRNA_004239	4:31369704|31396823
circRNA_017743	18:44321915|44322079	circRNA_018242	2:142541102|142548691	circRNA_003602	15:111918691|111930245	circRNA_014763	1:143267765|143294885
circRNA_020133	3:40623157|40623321	circRNA_010943	X:101995031|102002622	circRNA_001817	6:69143298|69154853	circRNA_008272	X:121758116|121785242
circRNA_019465	12:48991484|48991649	circRNA_004550	6:39555556|39563150	circRNA_009413	3:122055728|122067285	circRNA_016478	9:78882583|78909716
circRNA_020863	2:29127206|29127371	circRNA_006481	9:78742154|78749748	circRNA_015184	1:87750873|87762430	circRNA_010111	X:17788131|17815267
circRNA_000661	13:141209108|141209274	circRNA_002033	7:116968349|116975944	circRNA_017289	8:112720112|112731673	circRNA_010561	2:26857203|26884351
circRNA_012018	8:108946304|108946472	circRNA_016461	9:51073473|51081071	circRNA_004431	5:38338234|38349797	circRNA_000180	1:202644306|202671463
circRNA_006311	6:148574747|148574916	circRNA_006095	3:104192112|104199715	circRNA_006182	4:129481287|129492850	circRNA_018953	2:145725178|145752338
circRNA_009517	5:4796010|4796179	circRNA_008017	8:64483430|64491033	circRNA_001369	2:148134331|148145898	circRNA_014704	4:122750450|122777613
circRNA_014471	6:64444239|64444408	circRNA_005470	1:181051819|181059423	circRNA_008239	X:16343606|16355173	circRNA_011182	12:47804289|47831461
circRNA_016593	X:68446792|68446961	circRNA_012203	1:33407640|33415246	circRNA_009788	7:91938950|91950519	circRNA_013353	7:116819453|116846628
circRNA_003413	14:98575671|98575841	circRNA_015798	17:44400340|44407946	circRNA_009371	3:73637777|73649349	circRNA_003673	16:24933969|24961157
circRNA_005893	16:36648272|36648442	circRNA_010979	1:94549066|94556674	circRNA_014594	X:15946735|15958311	circRNA_000451	12:16036543|16063733
circRNA_019060	5:11965181|11965352	circRNA_004467	5:73481981|73489592	circRNA_016113	4:135613831|135625407	circRNA_017702	16:25433584|25460777
circRNA_004685	6:146642198|146642370	circRNA_015890	2:80455704|80463317	circRNA_008930	14:81076242|81087819	circRNA_007030	13:92217338|92244533
circRNA_004928	8:57827300|57827472	circRNA_012508	13:14154446|14162061	circRNA_011129	10:76272294|76283871	circRNA_008902	14:49996854|50024055
circRNA_010557	2:5808933|5809105	circRNA_012114	GL893654.2:8369|15986	circRNA_011610	2:87535724|87547302	circRNA_003863	18:52580507|52607709
circRNA_016211	6:51995850|51996022	circRNA_004614	6:92172007|92179625	circRNA_001478	3:102403868|102415447	circRNA_000588	13:49972583|49999786
circRNA_020834	18:20529164|20529336	circRNA_005267	GL895423.1:7115|14733	circRNA_015326	10:14918794|14930373	circRNA_020477	1:282164941|282192144
circRNA_021130	6:122107782|122107954	circRNA_018467	1:176833200|176840819	circRNA_002631	1:181041196|181052777	circRNA_007408	18:52580507|52607712
circRNA_017131	4:135989206|135989379	circRNA_003085	13:28307594|28315215	circRNA_019735	5:65026933|65038516	circRNA_016679	1:218548517|218575722
circRNA_017855	5:23107665|23107838	circRNA_007907	7:36615306|36622927	circRNA_012550	13:73331002|73342587	circRNA_004579	6:79246954|79274163
circRNA_019682	3:110477015|110477188	circRNA_018042	JH118990.1:97664|105288	circRNA_007055	13:133765083|133776669	circRNA_002104	8:80764338|80791563
circRNA_008797	13:97383429|97383604	circRNA_019984	12:37370893|37378518	circRNA_002065	8:20670183|20681770	circRNA_019794	7:39286085|39313313
circRNA_018535	10:19935323|19935498	circRNA_003394	14:79600633|79608261	circRNA_020537	11:69828125|69839712	circRNA_010434	14:51507654|51534899
circRNA_019514	13:217389424|217389599	circRNA_006490	9:96045833|96053461	circRNA_015999	3:82781299|82792891	circRNA_016118	4:139682020|139709266
circRNA_011130	10:78817191|78817370	circRNA_002504	1:60892775|60900407	circRNA_017798	3:72499745|72511337	circRNA_019524	14:33446619|33473875
circRNA_014481	6:102946173|102946352	circRNA_002761	1:300390656|300398288	circRNA_007930	7:73100247|73111840	circRNA_008600	11:24610341|24637599
circRNA_018384	GL893815.1:4961|5142	circRNA_017318	9:31047914|31055546	circRNA_020101	18:26368044|26379639	circRNA_016349	8:32865811|32893069
circRNA_018125	13:34782276|34782458	circRNA_001894	6:120005757|120013390	circRNA_009752	7:57275722|57287321	circRNA_014718	6:81553639|81580900
circRNA_018391	GL896305.1:21454|21636	circRNA_001727	5:63597843|63605477	circRNA_019898	1:8508285|8519889	circRNA_005159	9:117813587|117840855
circRNA_002210	9:35501413|35501596	circRNA_018897	2:16918297|16925931	circRNA_008754	13:51765369|51776974	circRNA_017119	4:98044696|98071965
circRNA_020111	2:5807333|5807517	circRNA_007944	7:97739664|97747301	circRNA_004996	8:118373542|118385149	circRNA_004532	6:16298300|16325581
circRNA_020344	GL895864.2:178494|178678	circRNA_013201	5:92253413|92261050	circRNA_007409	18:57411111|57422720	circRNA_006879	11:23622906|23650191

circRNA_001421	3:51429296|51429481	circRNA_016498	9:115697945|115705584	circRNA_003436	14:120300440|120312050	circRNA_001031	15:157067652|157094938
circRNA_010530	17:58806246|58806433	circRNA_020589	13:44001591|44009230	circRNA_018782	15:65960044|65971654	circRNA_002725	1:259301794|259329084
circRNA_016423	9:380437|380624	circRNA_011534	18:8233785|8241425	circRNA_003628	15:122449873|122461484	circRNA_009753	7:57942275|57969573
circRNA_019224	8:93154227|93154416	circRNA_016515	GL893374.2:642|8283	circRNA_018452	1:143087217|143098829	circRNA_004610	6:92070354|92097655
circRNA_019556	14:142879963|142880152	circRNA_009327	3:11499694|11507336	circRNA_002851	10:62755545|62767160	circRNA_013321	7:56899158|56926465
circRNA_013916	4:93621825|93622015	circRNA_013361	7:124987073|124994717	circRNA_003075	13:20319159|20330776	circRNA_002290	9:129664193|129691506
circRNA_018627	13:33956135|33956326	circRNA_013736	9:78899642|78907286	circRNA_013933	6:45079594|45091211	circRNA_007859	6:118020841|118048164
circRNA_006550	MT:2669|2862	circRNA_020005	13:84704237|84711882	circRNA_016225	6:79863286|79874911	circRNA_001860	6:99075904|99103249
circRNA_011843	5:92243038|92243231	circRNA_002309	9:144916302|144923948	circRNA_017462	1:153683539|153695164	circRNA_005394	X:139941678|139969029
circRNA_018195	16:57312562|57312755	circRNA_014144	1:282164941|282172587	circRNA_018088	1:153683536|153695164	circRNA_006381	8:3290947|3318329
circRNA_017877	6:33100382|33100576	circRNA_010590	2:128528893|128536541	circRNA_001295	2:87204233|87215862	circRNA_014631	12:13904160|13931543
circRNA_019840	9:9101193|9101387	circRNA_008743	13:43344526|43352176	circRNA_013148	5:16676591|16688220	circRNA_007645	4:39915930|39943326
circRNA_020154	3:118060748|118060942	circRNA_017594	13:73334937|73342587	circRNA_008836	13:157174806|157186436	circRNA_020244	6:150861612|150889008
circRNA_001434	3:67300428|67300623	circRNA_005483	1:202611390|202619041	circRNA_015046	7:87883201|87894833	circRNA_010451	14:116594582|116621990
circRNA_002587	1:134677508|134677703	circRNA_020996	3:134958503|134966155	circRNA_017269	8:40669625|40681257	circRNA_018334	7:73039410|73066822
circRNA_008953	14:117660562|117660757	circRNA_016617	1:8838522|8846175	circRNA_000415	11:52232763|52244398	circRNA_020122	2:119891914|119919334
circRNA_012168	X:114341699|114341894	circRNA_008700	13:2391632|2399288	circRNA_009143	17:36500876|36512513	circRNA_021132	6:129989880|130017301
circRNA_015672	14:140331091|140331286	circRNA_000685	13:166909686|166917343	circRNA_014036	1:143995379|144007016	circRNA_020734	15:66073507|66100930
circRNA_018121	12:44818088|44818283	circRNA_006072	3:46253973|46261633	circRNA_005923	17:39442884|39454524	circRNA_016172	5:87192177|87219626
circRNA_017189	6:100562469|100562665	circRNA_014540	9:7105929|7113589	circRNA_019391	1:200653848|200665488	circRNA_011818	5:54862446|54889896
circRNA_017640	14:80559604|80559800	circRNA_007111	14:34695400|34703061	circRNA_017460	1:140671383|140683030	circRNA_012291	1:206446036|206473489
circRNA_012817	16:52475183|52475380	circRNA_017679	15:97749979|97757643	circRNA_012141	JH118478.1:115535|127183	circRNA_004690	6:147024893|147052351
circRNA_002230	9:46861375|46861574	circRNA_010274	10:21583058|21590724	circRNA_010327	12:23727465|23739114	circRNA_002014	7:94505656|94533117
circRNA_007056	13:135148300|135148500	circRNA_014143	1:264888802|264896468	circRNA_000641	13:117594277|117605927	circRNA_019636	2:91513977|91541449
circRNA_018106	10:46681560|46681760	circRNA_000321	10:37123723|37131391	circRNA_011710	3:92450105|92461755	circRNA_007478	2:86103896|86131375
circRNA_006358	7:99573866|99574068	circRNA_004232	4:15264775|15272443	circRNA_015457	12:47645731|47657382	circRNA_019244	9:8159257|8186742
circRNA_014026	7:69682421|69682624	circRNA_008311	1:40759887|40767556	circRNA_003426	14:115746772|115758424	circRNA_008965	14:137819183|137846669
circRNA_017367	GL893931.2:2453|2656	circRNA_015459	12:48643105|48650775	circRNA_018309	6:74980742|74992395	circRNA_021126	6:102006733|102034254
circRNA_017949	8:11470254|11470458	circRNA_017215	7:1921718|1929389	circRNA_019159	7:56914812|56926465	circRNA_017446	1:103188266|103215801
circRNA_021234	8:139795277|139795481	circRNA_013740	9:133364153|133371827	circRNA_002671	1:202974507|202986161	circRNA_009086	16:25363856|25391399
circRNA_000776	14:52830738|52830945	circRNA_014856	6:144709992|144717668	circRNA_008367	1:128019978|128031634	circRNA_008331	1:80328846|80356402
circRNA_018607	12:44738008|44738215	circRNA_000376	11:3766438|3774119	circRNA_010612	3:39564463|39576119	circRNA_014694	3:72492406|72519969
circRNA_017572	12:45172833|45173042	circRNA_012432	11:22173482|22181163	circRNA_016794	13:59883791|59895449	circRNA_018470	1:181050309|181077882
circRNA_017748	2:20031534|20031743	circRNA_014977	11:18597764|18605445	circRNA_000783	14:59040543|59052202	circRNA_014598	X:73903076|73930652
circRNA_018704	14:29959860|29960069	circRNA_004911	8:40451377|40459063	circRNA_000677	13:150105423|150117084	circRNA_013541	GL895238.2:49303|76883
circRNA_004247	4:36253818|36254028	circRNA_007339	17:5971454|5979140	circRNA_018029	GL894466.1:10576|22237	circRNA_016636	1:97924611|97952202
circRNA_011236	13:86781175|86781385	circRNA_010663	4:42114119|42121807	circRNA_004004	2:128479853|128491516	circRNA_016643	1:124822521|124850118
circRNA_015183	1:86364308|86364519	circRNA_014748	GL893689.1:3069|10757	circRNA_004033	2:146504494|146516158	circRNA_007487	2:91169756|91197361
circRNA_000921	15:61519059|61519271	circRNA_015936	2:142518692|142526384	circRNA_007557	3:60994827|61006491	circRNA_010366	13:42364695|42392300
circRNA_001777	6:870107|870319	circRNA_014091	1:9432506|9440203	circRNA_011248	13:102283761|102295425	circRNA_008759	13:55847657|55875263
circRNA_009329	3:13957642|13957854	circRNA_002153	8:138126751|138134451	circRNA_003296	14:10459275|10470943	circRNA_015167	1:48735903|48763509
circRNA_020675	14:39955674|39955886	circRNA_002340	GL893953.2:86386|94086	circRNA_005217	GL892486.1:11033|22703	circRNA_008510	10:17019078|17046690
circRNA_016428	9:11017516|11017729	circRNA_016010	3:107388014|107395714	circRNA_008833	13:149623912|149635582	circRNA_009807	7:117145061|117172676
circRNA_006833	10:48227051|48227265	circRNA_014358	2:48835399|48843100	circRNA_002469	1:17123107|17134778	circRNA_019663	3:16566447|16594063
circRNA_016588	X:51413426|51413641	circRNA_017604	13:102280273|102287975	circRNA_004490	5:91474738|91486409	circRNA_013156	5:31795585|31823209
circRNA_007575	3:92448071|92448287	circRNA_003942	2:83659629|83667335	circRNA_020346	MT:1378|13051	circRNA_018320	6:138061194|138088819
circRNA_019090	6:868443|868659	circRNA_006323	7:20415577|20423283	circRNA_019010	3:122067100|122078774	circRNA_007518	2:145725178|145752821
circRNA_019337	X:41055142|41055358	circRNA_010869	9:29829631|29837338	circRNA_010769	6:137375783|137387459	circRNA_003765	17:36391525|36419177
circRNA_017487	1:209531997|209532215	circRNA_007483	2:90151050|90158760	circRNA_013499	9:132597635|132609312	circRNA_014015	17:43054927|43082579
circRNA_019811	7:119245301|119245519	circRNA_001867	6:99360832|99368547	circRNA_020998	3:135192228|135203906	circRNA_014225	13:160212018|160239674
circRNA_000503	12:50456560|50456779	circRNA_002807	10:35747877|35755595	circRNA_011701	3:80412430|80424109	circRNA_002221	9:42973775|43001433
circRNA_014498	7:72641076|72641295	circRNA_011986	8:2826774|2834492	circRNA_020405	1:126936876|126948555	circRNA_011058	1:269912309|269939970

circRNA_017745	2:8548642|8548863	circRNA_015402	11:56299050|56306768	circRNA_004486	5:87932536|87944218	circRNA_003416	14:104579807|104607474
circRNA_021049	5:31768082|31768303	circRNA_013533	GL894963.1:306|8025	circRNA_016365	8:57403788|57415474	circRNA_020118	2:89007457|89035133
circRNA_016936	16:37502252|37502474	circRNA_011522	17:43623889|43631612	circRNA_016649	1:137673225|137684911	circRNA_003331	14:32154185|32181866
circRNA_017532	10:58661836|58662058	circRNA_018713	14:31729382|31737109	circRNA_015756	16:36619422|36631111	circRNA_015525	13:86957566|86985258
circRNA_010878	9:51072580|51072804	circRNA_006766	1:257754306|257762035	circRNA_019054	4:137136976|137148667	circRNA_008441	1:216036130|216063829
circRNA_012580	13:130133705|130133929	circRNA_020425	1:176789554|176797284	circRNA_005576	11:501996|513694	circRNA_007780	5:94451221|94478927
circRNA_017315	9:21816249|21816473	circRNA_011977	7:121224372|121232103	circRNA_013042	3:102852283|102863985	circRNA_020653	13:190890857|190918571
circRNA_019173	7:81414210|81414434	circRNA_002814	10:37486613|37494346	circRNA_009121	16:74204582|74216287	circRNA_001494	3:113380479|113408214
circRNA_013017	3:62333358|62333584	circRNA_003835	18:25350802|25358537	circRNA_014329	17:42867884|42879589	circRNA_013282	6:140633018|140660768
circRNA_017953	8:49271034|49271260	circRNA_009309	2:142115988|142123724	circRNA_000843	14:115712680|115724388	circRNA_001102	17:42172958|42200711
circRNA_017001	2:8548647|8548874	circRNA_001518	3:122048175|122055914	circRNA_003672	16:23716606|23728315	circRNA_016106	4:126532107|126559860
circRNA_020494	10:12266445|12266672	circRNA_016713	10:14918794|14926534	circRNA_000275	10:241981|253691	circRNA_018220	2:30841324|30869078
circRNA_007834	6:78302432|78302660	circRNA_015912	2:118300508|118308249	circRNA_005942	18:15102684|15114394	circRNA_016020	3:113380459|113408214
circRNA_015813	18:10605179|10605407	circRNA_003006	12:37266915|37274657	circRNA_011362	14:115775888|115787598	circRNA_019611	18:48032085|48059841
circRNA_017360	GL893259.1:38835|39063	circRNA_021037	4:136151405|136159147	circRNA_012397	10:66171809|66183519	circRNA_003915	2:52849516|52877281
circRNA_017722	17:43359048|43359276	circRNA_020783	16:29443058|29450805	circRNA_013293	6:152386478|152398188	circRNA_015726	15:117772992|117800760
circRNA_014268	14:142840673|142840902	circRNA_006972	13:21212492|21220240	circRNA_011253	13:117723642|117735353	circRNA_011625	2:112510330|112538105
circRNA_008031	8:86052355|86052585	circRNA_015698	15:66305700|66313450	circRNA_014180	12:23732147|23743858	circRNA_016989	18:27278420|27306195
circRNA_011149	11:74761647|74761877	circRNA_007070	13:150216320|150224071	circRNA_003718	16:51317149|51328861	circRNA_002535	1:92259145|92286929
circRNA_019441	11:6734231|6734463	circRNA_002743	1:280627976|280635728	circRNA_013796	10:49576020|49587733	circRNA_016735	11:18571298|18599085
circRNA_015899	2:92130737|92130971	circRNA_010038	GL892407.1:19130|26882	circRNA_009209	18:59551551|59563265	circRNA_006319	7:1987011|2014810
circRNA_003128	13:58515765|58516000	circRNA_015998	3:77628023|77635775	circRNA_016069	4:55378652|55390366	circRNA_002677	1:205486844|205514653
circRNA_007640	4:39577226|39577461	circRNA_018435	1:116834276|116842031	circRNA_001471	3:101033374|101045089	circRNA_019097	6:33613086|33640899
circRNA_010291	10:58014709|58014944	circRNA_018675	13:156040164|156047922	circRNA_003355	14:53590430|53602149	circRNA_005479	1:200310792|200338610
circRNA_010211	1:153748223|153748459	circRNA_004857	7:124821065|124828826	circRNA_002876	11:6990316|7002040	circRNA_011563	2:3011482|3039309
circRNA_012183	1:17075533|17075769	circRNA_020598	13:75396974|75404735	circRNA_010020	9:138716542|138728267	circRNA_014156	10:44171899|44199737
circRNA_018339	7:102319883|102320119	circRNA_010791	7:58178875|58186637	circRNA_016601	X:101641970|101653701	circRNA_002537	1:94523212|94551052
circRNA_004337	4:109346740|109346978	circRNA_005268	GL895741.1:18267|26033	circRNA_003449	14:124746430|124758162	circRNA_013798	10:63447505|63475346
circRNA_020347	MT:3612|3850	circRNA_006711	1:161224496|161232262	circRNA_005898	16:47813571|47825304	circRNA_019653	2:140473312|140501161
circRNA_004089	3:55783850|55784090	circRNA_016831	13:212906970|212914737	circRNA_009006	15:54571013|54582748	circRNA_020967	3:74739616|74767479
circRNA_003892	2:29224763|29225004	circRNA_013363	7:128880005|128887774	circRNA_019025	4:48110722|48122458	circRNA_021245	9:18937518|18965384
circRNA_016812	13:109620091|109620332	circRNA_003022	12:44468807|44476577	circRNA_019931	1:195022523|195034259	circRNA_018223	2:50780520|50808396
circRNA_019698	4:60858276|60858517	circRNA_018118	12:23750110|23757880	circRNA_020622	13:130090199|130101937	circRNA_003312	14:21576022|21603902
circRNA_019660	2:157644665|157644907	circRNA_016091	4:98120612|98128385	circRNA_020009	13:117188877|117200616	circRNA_006287	6:96170997|96198889
circRNA_008779	13:85560202|85560445	circRNA_004740	7:31326169|31333945	circRNA_005867	15:116425424|116437165	circRNA_005887	16:23470173|23498068
circRNA_008567	10:70577541|70577785	circRNA_020108	2:1595082|1602858	circRNA_007587	3:104197942|104209684	circRNA_011177	12:39197540|39225437
circRNA_011632	2:124685612|124685857	circRNA_006080	3:76474842|76482619	circRNA_003696	16:38531076|38542819	circRNA_012577	13:117695880|117723777
circRNA_014046	15:80723697|80723942	circRNA_009810	7:120157255|120165036	circRNA_004045	2:150648583|150660330	circRNA_009021	15:73545877|73573786
circRNA_015439	12:25090844|25091090	circRNA_020409	1:134653309|134661090	circRNA_009645	6:73282196|73293946	circRNA_004975	8:93140698|93168617
circRNA_006656	1:97996195|97996442	circRNA_016516	GL893655.1:37492|45274	circRNA_006592	1:1175729|1187482	circRNA_013708	2:119185369|119213291
circRNA_015678	15:3820390|3820638	circRNA_002741	1:280567722|280575505	circRNA_017104	4:45564608|45576363	circRNA_019918	1:84754696|84782618
circRNA_018217	2:5812521|5812769	circRNA_016221	6:73334893|73342678	circRNA_019262	9:40775540|40787296	circRNA_001636	4:117099716|117127644
circRNA_019869	GL894261.2:66477|66725	circRNA_016654	1:143245293|143253079	circRNA_009176	18:10795154|10806911	circRNA_008835	13:150586617|150614548
circRNA_010573	2:80139481|80139731	circRNA_020902	2:112591635|112599422	circRNA_019036	4:73698969|73710726	circRNA_014372	2:119260427|119288360
circRNA_011173	12:34156551|34156801	circRNA_007993	8:32687757|32695547	circRNA_006071	3:45686483|45698245	circRNA_004886	8:18856400|18884334
circRNA_007248	15:73552151|73552402	circRNA_007609	3:122012247|122020038	circRNA_016973	18:10795154|10806919	circRNA_008952	14:117079612|117107550
circRNA_011803	5:15824331|15824582	circRNA_004020	2:138757331|138765124	circRNA_004154	3:102653727|102665493	circRNA_003242	13:167080830|167108777
circRNA_002845	10:58923707|58923959	circRNA_007146	14:68471642|68479436	circRNA_009290	2:121372041|121383808	circRNA_020468	1:256839802|256867750
circRNA_015422	12:13871060|13871312	circRNA_014142	1:257085934|257093728	circRNA_000525	13:2382115|2393883	circRNA_019055	4:139828383|139856337
circRNA_017093	4:15272191|15272443	circRNA_009864	8:89359060|89366855	circRNA_002370	GL896270.1:12583|24351	circRNA_004465	5:70819577|70847537
circRNA_021338	GL896447.1:74778|75030	circRNA_020445	1:200290313|200298109	circRNA_010108	X:10418106|10429874	circRNA_007230	15:53189660|53217621
circRNA_019877	GL896294.1:106241|106494	circRNA_008868	14:933854|941652	circRNA_013402	8:93306923|93318694	circRNA_017272	8:45694664|45722626

<table>
<tr><td>circRNA_009322</td><td>2:154314345|154314599</td><td>circRNA_014623</td><td>10:54023860|54031658</td><td>circRNA_005367</td><td>X:116361755|116373528</td><td>circRNA_005214</td><td>GL892397.1:95227|123194</td></tr>
<tr><td>circRNA_020912</td><td>2:122626987|122627241</td><td>circRNA_019539</td><td>14:82334754|82342553</td><td>circRNA_013962</td><td>9:78746589|78758362</td><td>circRNA_004451</td><td>5:63577496|63605477</td></tr>
<tr><td>circRNA_001346</td><td>2:133350705|133350960</td><td>circRNA_005360</td><td>X:105400134|105407934</td><td>circRNA_013028</td><td>3:84263223|84274997</td><td>circRNA_004511</td><td>5:110441061|110469054</td></tr>
<tr><td>circRNA_003911</td><td>2:50007088|50007343</td><td>circRNA_009000</td><td>15:53105398|53113201</td><td>circRNA_011634</td><td>2:128435275|128447051</td><td>circRNA_008755</td><td>13:53872565|53900565</td></tr>
<tr><td>circRNA_006304</td><td>6:132195039|132195294</td><td>circRNA_018591</td><td>12:20311870|20319673</td><td>circRNA_016691</td><td>1:256867659|256879435</td><td>circRNA_005624</td><td>12:36516640|36544644</td></tr>
<tr><td>circRNA_010668</td><td>4:67726227|67726482</td><td>circRNA_007271</td><td>15:111745404|111753209</td><td>circRNA_005674</td><td>13:50636313|50648094</td><td>circRNA_000214</td><td>1:241875289|241903294</td></tr>
<tr><td>circRNA_011036</td><td>1:208261443|208261698</td><td>circRNA_016401</td><td>8:122227452|122235259</td><td>circRNA_020717</td><td>14:136748505|136760286</td><td>circRNA_010660</td><td>4:40712731|40740740</td></tr>
<tr><td>circRNA_016560</td><td>JH118496.1:23989|24244</td><td>circRNA_015884</td><td>2:60037167|60044976</td><td>circRNA_018516</td><td>1:270342348|270354137</td><td>circRNA_018998</td><td>3:104155199|104183209</td></tr>
<tr><td>circRNA_002470</td><td>1:17131976|17132232</td><td>circRNA_009128</td><td>17:5196694|5204505</td><td>circRNA_008624</td><td>11:75120545|75132336</td><td>circRNA_004687</td><td>6:147017881|147045896</td></tr>
<tr><td>circRNA_012147</td><td>MT:1532|1788</td><td>circRNA_015787</td><td>17:5196690|5204501</td><td>circRNA_011936</td><td>7:37121359|37133150</td><td>circRNA_009987</td><td>9:88615508|88643532</td></tr>
<tr><td>circRNA_014976</td><td>11:13281157|13281413</td><td>circRNA_009984</td><td>9:78901228|78909044</td><td>circRNA_011978</td><td>7:122497132|122508924</td><td>circRNA_008268</td><td>X:109048761|109076797</td></tr>
<tr><td>circRNA_016046</td><td>4:129714|129970</td><td>circRNA_018122</td><td>13:15541838|15549655</td><td>circRNA_014016</td><td>2:132248051|132259847</td><td>circRNA_012440</td><td>11:53898878|53926927</td></tr>
<tr><td>circRNA_021349</td><td>MT:3033|3289</td><td>circRNA_015117</td><td>12:62112316|62120134</td><td>circRNA_006161</td><td>4:90924840|90936640</td><td>circRNA_013482</td><td>9:112750007|112778058</td></tr>
<tr><td>circRNA_010443</td><td>14:79264964|79265221</td><td>circRNA_012355</td><td>10:33080703|33088522</td><td>circRNA_015750</td><td>16:25053149|25064949</td><td>circRNA_000351</td><td>10:58881715|58909767</td></tr>
<tr><td>circRNA_015495</td><td>13:35739632|35739889</td><td>circRNA_000925</td><td>15:66305630|66313450</td><td>circRNA_017961</td><td>8:88578660|88590460</td><td>circRNA_016632</td><td>1:60917934|60945988</td></tr>
<tr><td>circRNA_021081</td><td>5:105296613|105296870</td><td>circRNA_013031</td><td>3:90576383|90584203</td><td>circRNA_006923</td><td>12:23389673|23401474</td><td>circRNA_002745</td><td>1:280627976|280656034</td></tr>
<tr><td>circRNA_005300</td><td>JH118993.1:13015|13273</td><td>circRNA_009946</td><td>9:40767814|40775635</td><td>circRNA_018303</td><td>6:26453983|26465785</td><td>circRNA_004679</td><td>6:143689203|143717262</td></tr>
<tr><td>circRNA_008712</td><td>13:15403921|15404179</td><td>circRNA_002550</td><td>1:110525008|110532832</td><td>circRNA_001619</td><td>4:102984325|102996128</td><td>circRNA_000765</td><td>14:34702864|34730945</td></tr>
<tr><td>circRNA_000825</td><td>14:86827182|86827441</td><td>circRNA_008529</td><td>10:35382114|35389938</td><td>circRNA_008099</td><td>9:36642256|36654059</td><td>circRNA_015409</td><td>11:76373396|76401491</td></tr>
<tr><td>circRNA_018539</td><td>10:27824642|27824901</td><td>circRNA_006802</td><td>10:2279240|2287065</td><td>circRNA_014319</td><td>16:53246049|53257852</td><td>circRNA_005545</td><td>10:25127029|25155126</td></tr>
<tr><td>circRNA_009832</td><td>8:32779527|32779787</td><td>circRNA_007633</td><td>4:34609769|34617596</td><td>circRNA_019002</td><td>3:114020620|114032423</td><td>circRNA_015781</td><td>16:68471430|68499529</td></tr>
<tr><td>circRNA_019394</td><td>1:208268088|208268350</td><td>circRNA_000880</td><td>15:3338983|3346811</td><td>circRNA_005368</td><td>X:116966324|116978128</td><td>circRNA_020823</td><td>18:814124|842224</td></tr>
<tr><td>circRNA_020067</td><td>15:118280698|118280960</td><td>circRNA_006049</td><td>2:147049942|147057770</td><td>circRNA_009876</td><td>8:103914218|103926027</td><td>circRNA_020220</td><td>6:74096530|74124642</td></tr>
<tr><td>circRNA_005171</td><td>9:128949520|128949783</td><td>circRNA_011327</td><td>14:53840980|53848809</td><td>circRNA_009007</td><td>15:55345812|55357622</td><td>circRNA_004750</td><td>7:36383966|36412083</td></tr>
<tr><td>circRNA_018872</td><td>18:30313540|30313804</td><td>circRNA_019505</td><td>13:190918409|190926244</td><td>circRNA_000405</td><td>11:25429385|25441196</td><td>circRNA_020645</td><td>13:160211541|160239674</td></tr>
<tr><td>circRNA_003818</td><td>18:14695794|14696059</td><td>circRNA_008107</td><td>9:40614116|40621954</td><td>circRNA_006372</td><td>7:121217521|121229341</td><td>circRNA_011472</td><td>16:25363265|25391399</td></tr>
<tr><td>circRNA_019497</td><td>13:149285194|149285459</td><td>circRNA_009089</td><td>16:27954830|27962669</td><td>circRNA_020940</td><td>3:29685091|29696912</td><td>circRNA_006419</td><td>8:87965563|87993701</td></tr>
<tr><td>circRNA_019445</td><td>11:17292364|17292630</td><td>circRNA_013703</td><td>2:43809127|43816968</td><td>circRNA_001466</td><td>3:98116845|98128668</td><td>circRNA_010182</td><td>1:60931627|60959765</td></tr>
<tr><td>circRNA_020233</td><td>6:109021549|109021815</td><td>circRNA_006957</td><td>13:1433020|1440862</td><td>circRNA_002720</td><td>1:256867612|256879435</td><td>circRNA_009116</td><td>16:57092936|57121075</td></tr>
<tr><td>circRNA_003114</td><td>13:43392088|43392355</td><td>circRNA_020910</td><td>2:122545704|122553546</td><td>circRNA_014140</td><td>1:247961314|247973138</td><td>circRNA_003469</td><td>14:142775864|142804005</td></tr>
<tr><td>circRNA_021015</td><td>4:61068208|61068475</td><td>circRNA_012002</td><td>8:57799376|57807219</td><td>circRNA_011573</td><td>2:29213178|29225004</td><td>circRNA_006878</td><td>11:23622033|23650191</td></tr>
<tr><td>circRNA_018778</td><td>15:52949969|52950237</td><td>circRNA_003518</td><td>15:53153877|53161722</td><td>circRNA_015415</td><td>12:11466494|11478325</td><td>circRNA_017378</td><td>GL896504.1:27302|55460</td></tr>
<tr><td>circRNA_002662</td><td>1:202611390|202611659</td><td>circRNA_007229</td><td>15:51895302|51903148</td><td>circRNA_021242</td><td>9:9089546|9101387</td><td>circRNA_019158</td><td>7:56643754|56671934</td></tr>
<tr><td>circRNA_007093</td><td>14:7344496|7344765</td><td>circRNA_008011</td><td>8:57987889|57995735</td><td>circRNA_008225</td><td>GL896423.1:11293|23135</td><td>circRNA_000948</td><td>15:81483550|81511732</td></tr>
<tr><td>circRNA_015449</td><td>12:40198683|40198952</td><td>circRNA_013825</td><td>13:131025432|131033279</td><td>circRNA_020624</td><td>13:131341476|131353318</td><td>circRNA_001240</td><td>2:43788778|43816968</td></tr>
<tr><td>circRNA_007713</td><td>5:4426333|4426603</td><td>circRNA_019598</td><td>17:44925256|44933103</td><td>circRNA_014423</td><td>4:70410831|70422678</td><td>circRNA_016306</td><td>7:73495104|73523294</td></tr>
<tr><td>circRNA_015581</td><td>13:210621737|210622008</td><td>circRNA_013376</td><td>8:32680016|32687865</td><td>circRNA_000890</td><td>15:22575140|22586988</td><td>circRNA_004046</td><td>2:153574244|153602436</td></tr>
<tr><td>circRNA_007399</td><td>18:33305187|33305459</td><td>circRNA_008297</td><td>1:17240890|17248742</td><td>circRNA_000336</td><td>10:47064149|47075999</td><td>circRNA_019040</td><td>4:96774978|96803170</td></tr>
<tr><td>circRNA_011682</td><td>3:49148795|49149067</td><td>circRNA_013109</td><td>4:83633287|83641141</td><td>circRNA_013726</td><td>7:102205505|102217361</td><td>circRNA_011907</td><td>6:134517053|134545250</td></tr>
<tr><td>circRNA_005309</td><td>MT:10140|10413</td><td>circRNA_007224</td><td>15:37831362|37839219</td><td>circRNA_013302</td><td>7:10553476|10565336</td><td>circRNA_017489</td><td>1:225622829|225651027</td></tr>
<tr><td>circRNA_014923</td><td>15:73179254|73179527</td><td>circRNA_013027</td><td>3:81716262|81724119</td><td>circRNA_011340</td><td>14:71889478|71901340</td><td>circRNA_008119</td><td>9:52649358|52677558</td></tr>
<tr><td>circRNA_005445</td><td>1:132954733|132955007</td><td>circRNA_018503</td><td>1:242153420|242161279</td><td>circRNA_002816</td><td>10:44171899|44183763</td><td>circRNA_008364</td><td>1:127648623|127676835</td></tr>
<tr><td>circRNA_013731</td><td>8:112183571|112183845</td><td>circRNA_002800</td><td>10:33271094|33278954</td><td>circRNA_007415</td><td>18:59685146|59697010</td><td>circRNA_012514</td><td>13:24038595|24066809</td></tr>
<tr><td>circRNA_016763</td><td>12:37778824|37779098</td><td>circRNA_010190</td><td>1:85695289|85703152</td><td>circRNA_003340</td><td>14:42057244|42069112</td><td>circRNA_012430</td><td>11:21053208|21081424</td></tr>
<tr><td>circRNA_001277</td><td>2:78551140|78551415</td><td>circRNA_012668</td><td>14:81031680|81039544</td><td>circRNA_016133</td><td>5:29902554|29914422</td><td>circRNA_012986</td><td>2:156366504|156394730</td></tr>
<tr><td>circRNA_018144</td><td>13:206126722|206126997</td><td>circRNA_006476</td><td>9:43047664|43055529</td><td>circRNA_006931</td><td>12:32369890|32381760</td><td>circRNA_014934</td><td>4:83569528|83597759</td></tr>
<tr><td>circRNA_010835</td><td>8:58339082|58339359</td><td>circRNA_007544</td><td>3:29719350|29727215</td><td>circRNA_007179</td><td>14:120779066|120790937</td><td>circRNA_008185</td><td>GL892789.1:66335|94569</td></tr>
<tr><td>circRNA_001838</td><td>6:83163559|83163837</td><td>circRNA_006283</td><td>6:90255339|90263205</td><td>circRNA_008463</td><td>1:245505757|245517628</td><td>circRNA_019712</td><td>4:143285894|143314129</td></tr>
<tr><td>circRNA_009768</td><td>7:69814148|69814426</td><td>circRNA_007540</td><td>3:29113882|29121751</td><td>circRNA_003758</td><td>17:30263142|30275014</td><td>circRNA_001455</td><td>3:84263223|84291468</td></tr>
<tr><td>circRNA_009985</td><td>9:78959290|78959568</td><td>circRNA_008851</td><td>13:202682827|202690697</td><td>circRNA_007765</td><td>5:79148742|79160617</td><td>circRNA_009740</td><td>7:36751630|36779878</td></tr>
<tr><td>circRNA_011055</td><td>1:257059754|257060032</td><td>circRNA_005105</td><td>9:40753526|40761397</td><td>circRNA_018576</td><td>11:19732646|19744523</td><td>circRNA_015098</td><td>5:87167848|87196099</td></tr>
<tr><td>circRNA_011869</td><td>6:46025639|46025917</td><td>circRNA_007769</td><td>5:82759652|82767524</td><td>circRNA_012164</td><td>X:105396055|105407934</td><td>circRNA_021092</td><td>6:18418914|18447166</td></tr>
</table>

circRNA_020858	2:8545690|8545968	circRNA_020817	17:57246222|57254096	circRNA_018369	9:50821415|50833295	circRNA_017921	6:150846440|150874694
circRNA_011965	7:100935504|100935783	circRNA_015230	1:188398368|188406243	circRNA_002657	1:200299009|200310890	circRNA_000718	13:210819581|210847843
circRNA_018352	8:75758787|75759066	circRNA_008043	8:108424880|108432759	circRNA_011597	2:61766031|61777912	circRNA_017456	1:134651078|134679346
circRNA_007421	2:9733475|9733755	circRNA_013194	5:88187371|88195251	circRNA_008368	1:132148098|132159987	circRNA_005748	13:211128404|211156675
circRNA_005895	16:42908481|42908762	circRNA_001514	3:122008499|122016380	circRNA_004549	6:39328900|39340790	circRNA_020755	15:105424453|105452728
circRNA_018845	17:29455168|29455449	circRNA_012606	13:206307834|206315716	circRNA_001362	2:145740930|145752821	circRNA_020462	1:244418080|244446360
circRNA_002532	1:89940534|89940816	circRNA_012682	14:116552528|116560410	circRNA_006022	2:100736163|100748055	circRNA_018863	18:12766762|12795054
circRNA_007161	14:96767080|96767362	circRNA_002960	12:14508500|14516384	circRNA_008019	8:71459662|71471554	circRNA_015597	14:21564778|21593078
circRNA_009276	2:95629644|95629926	circRNA_010942	X:97827755|97835641	circRNA_007908	7:37126123|37138017	circRNA_006123	4:12615332|12643641
circRNA_012604	13:199218141|199218423	circRNA_001520	3:122510653|122518540	circRNA_011918	6:148463817|148475711	circRNA_018411	1:45632972|45661284
circRNA_015171	1:59728051|59728333	circRNA_007772	5:87112433|87120323	circRNA_016260	6:147455617|147467514	circRNA_002446	X:130735402|130763718
circRNA_017723	17:45464077|45464359	circRNA_006531	GL894608.1:7692|15583	circRNA_019697	4:55200814|55212711	circRNA_021123	6:99212305|99240623
circRNA_020663	13:217426027|217426309	circRNA_012286	1:200427414|200435305	circRNA_017879	6:41561594|41573492	circRNA_007259	15:85490256|85518580
circRNA_002943	11:81586451|81586734	circRNA_012815	16:51753464|51761357	circRNA_009720	6:157208869|157220769	circRNA_014766	1:254908743|254937077
circRNA_013561	MT:14348|14631	circRNA_014149	10:19587694|19595587	circRNA_006585	X:126963525|126975426	circRNA_006788	1:287936886|287965225
circRNA_015911	2:112591635|112591920	circRNA_002753	1:284490232|284498130	circRNA_009928	9:22861306|22873213	circRNA_010757	6:113086834|113115194
circRNA_006775	1:270368515|270368801	circRNA_011072	1:303952596|303960495	circRNA_008245	X:30898666|30910575	circRNA_002817	10:45755013|45783374
circRNA_008004	8:41183979|41184265	circRNA_018809	15:133563077|133570976	circRNA_008392	1:161192141|161204051	circRNA_020850	18:59667683|59696045
circRNA_000578	13:42377857|42378144	circRNA_007118	14:43480945|43488845	circRNA_014408	3:122012247|122024159	circRNA_017569	12:38659145|38687508
circRNA_009041	15:92657331|92657618	circRNA_015749	16:23723018|23730920	circRNA_018287	5:63818388|63830303	circRNA_006640	1:62359384|62387755
circRNA_011198	13:19730728|19731015	circRNA_014827	3:39543227|39551131	circRNA_006748	1:215715817|215727733	circRNA_005262	GL895110.2:173471|201844
circRNA_010103	MT:3120|3408	circRNA_010134	X:70638360|70646265	circRNA_002827	10:47811460|47823378	circRNA_010360	13:34455803|34484182
circRNA_014179	12:20847738|20848026	circRNA_010970	1:64737843|64745751	circRNA_018817	16:23574659|23586581	circRNA_018062	1:228942|257333
circRNA_001300	2:88694251|88694540	circRNA_002335	GL893762.1:67815|75724	circRNA_020339	GL893884.1:41214|53137	circRNA_001167	18:18661914|18690307
circRNA_020127	2:140376825|140377114	circRNA_005633	12:48418687|48426596	circRNA_000132	1:141982455|141994380	circRNA_001543	4:2602892|2631285
circRNA_020211	6:42817785|42818074	circRNA_006873	11:21429243|21437155	circRNA_017065	3:42971689|42983616	circRNA_001568	4:42001869|42030271
circRNA_021323	GL894772.1:17608|17897	circRNA_007199	14:141173275|141181187	circRNA_005771	14:30073185|30085114	circRNA_018821	16:25363856|25392263
circRNA_007353	17:39599087|39599377	circRNA_005408	1:18675426|18683344	circRNA_008327	1:72029648|72041578	circRNA_012999	3:33342594|33371003
circRNA_013923	5:23209144|23209435	circRNA_007234	15:55337963|55345882	circRNA_017504	1:282203470|282215400	circRNA_005659	13:33720938|33749350
circRNA_000704	13:205921704|205921996	circRNA_020103	18:42650857|42658779	circRNA_004829	7:97693049|97704983	circRNA_014134	1:210127511|210155927
circRNA_014389	3:50658315|50658607	circRNA_010029	9:143476417|143484342	circRNA_011702	3:84698909|84710843	circRNA_021183	7:125239034|125267450
circRNA_018004	9:120410682|120410974	circRNA_002939	11:75102199|75110126	circRNA_014282	15:67040124|67052063	circRNA_012950	2:87148623|87177048
circRNA_001776	6:869994|870288	circRNA_015589	14:941571|949498	circRNA_013840	14:83673622|83685562	circRNA_015337	10:35260661|35289091
circRNA_011260	13:138999103|138999397	circRNA_019061	5:22724516|22732443	circRNA_003121	13:50643382|50655324	circRNA_008679	12:45469143|45497578
circRNA_010489	15:104101112|104101407	circRNA_005804	14:117614090|117622018	circRNA_018178	15:74266319|74278262	circRNA_008527	10:33582739|33611200
circRNA_010816	7:134318713|134319009	circRNA_008372	1:134607759|134615687	circRNA_012208	1:44457512|44469456	circRNA_018446	1:134284841|134313308
circRNA_017047	2:142043541|142043837	circRNA_008814	13:131099610|131107538	circRNA_018175	14:146799398|146811348	circRNA_014343	18:36452161|36480633
circRNA_017317	9:22872917|22873213	circRNA_015751	16:25383469|25391399	circRNA_014219	13:144543873|144555825	circRNA_010152	X:116341278|116369754
circRNA_014693	3:60664014|60664311	circRNA_020827	18:9562088|9570018	circRNA_001393	3:24136275|24148233	circRNA_015532	13:91050770|91079260
circRNA_015486	13:29503941|29504239	circRNA_012949	2:86103896|86111828	circRNA_015189	1:101052496|101064454	circRNA_016085	4:75094374|75122865
circRNA_004331	4:107280131|107280430	circRNA_014068	10:20007|27939	circRNA_010762	6:128407265|128419226	circRNA_000725	14:475829|504329
circRNA_000834	14:98695887|98696187	circRNA_015605	14:42003054|42010992	circRNA_006484	9:78959290|78971257	circRNA_020382	1:60896460|60924967
circRNA_004391	4:139885466|139885766	circRNA_013451	9:40955217|40963156	circRNA_000240	1:270464385|270476354	circRNA_014980	12:13065330|13093840
circRNA_005066	9:13992620|13992920	circRNA_016416	8:143393950|143401890	circRNA_001066	16:47892129|47904101	circRNA_019627	2:74365575|74394090
circRNA_014200	13:55492221|55492521	circRNA_001672	5:3454359|3462302	circRNA_014920	14:31011017|31022994	circRNA_006098	3:107382061|107410577
circRNA_021070	5:88010463|88010764	circRNA_004150	3:100607920|100615863	circRNA_000181	1:202861414|202873392	circRNA_020081	16:48674810|48703328
circRNA_001642	4:121647126|121647428	circRNA_020179	4:83746578|83754522	circRNA_010045	GL892553.2:91193|103174	circRNA_010116	X:43357944|43386463
circRNA_008882	14:30891028|30891330	circRNA_003002	12:37227369|37235315	circRNA_020441	1:188147577|188159561	circRNA_005421	1:45692534|45721056
circRNA_018034	GL896265.2:93645|93947	circRNA_007781	5:94452814|94460760	circRNA_004991	8:112040320|112052306	circRNA_017356	GL892680.2:154968|183490
circRNA_003621	15:118068018|118068321	circRNA_001963	7:43186596|43194543	circRNA_016441	9:29006576|29018562	circRNA_017303	8:144646658|144675196
circRNA_019828	8:89434966|89435269	circRNA_014486	7:10291894|10299841	circRNA_005885	16:23393877|23405864	circRNA_010154	X:122519791|122548340
circRNA_021353	X:14262458|14262761	circRNA_015202	1:121462435|121470382	circRNA_005536	10:11705431|11717419	circRNA_017946	7:129884224|129912773

circRNA_001658	4:134125263|134125567	circRNA_016129	5:17119726|17127674	circRNA_005903	16:56200707|56212695	circRNA_001889	6:108173849|108202404
circRNA_006376	7:129328763|129329067	circRNA_006433	8:139497435|139505384	circRNA_008688	12:52769179|52781167	circRNA_018547	10:46114484|46143041
circRNA_009458	4:53468554|53468858	circRNA_008917	14:65156356|65164305	circRNA_016110	4:129725177|129737167	circRNA_015509	13:50655026|50683600
circRNA_012383	10:54179586|54179891	circRNA_002614	1:151141440|151149390	circRNA_009966	9:47658915|47670908	circRNA_014495	7:65666367|65694943
circRNA_016554	GL896401.1:2659|2964	circRNA_011444	15:122453532|122461484	circRNA_000035	1:35194844|35206839	circRNA_014866	7:91949364|91977940
circRNA_010058	GL893852.2:67234|67540	circRNA_020480	1:283056939|283064891	circRNA_009824	8:12573552|12585547	circRNA_010755	6:103883315|103911899
circRNA_012553	13:79284414|79284720	circRNA_013358	7:124823524|124831477	circRNA_012188	1:21231837|21243834	circRNA_017677	15:94303061|94331645
circRNA_013759	6:47905556|47905862	circRNA_012480	12:27121764|27129718	circRNA_016848	14:60123994|60135991	circRNA_010713	5:74581540|74610130
circRNA_009138	17:29448874|29449181	circRNA_006871	11:19715398|19723353	circRNA_002366	GL895741.1:14035|26033	circRNA_012760	15:116343384|116371977
circRNA_017116	4:75175318|75175625	circRNA_018826	16:29673172|29681127	circRNA_004917	8:44891481|44903479	circRNA_020023	13:159562754|159591350
circRNA_016451	9:40532481|40532789	circRNA_008979	15:8281464|8289420	circRNA_002579	1:132441607|132453607	circRNA_002850	10:62738557|62767160
circRNA_018154	14:42896771|42897079	circRNA_016708	1:306168025|306175981	circRNA_014969	1:283102747|283114748	circRNA_000230	1:256850826|256879435
circRNA_004955	8:80672499|80672808	circRNA_020662	13:216378701|216386658	circRNA_018260	3:120140092|120152093	circRNA_011433	15:111901622|111930245
circRNA_006140	4:40633771|40634081	circRNA_008047	8:110783890|110791848	circRNA_002489	1:35488669|35500672	circRNA_015031	3:100012366|100040994
circRNA_018134	13:119598506|119598816	circRNA_009335	3:19652520|19660479	circRNA_021185	8:3231744|3243751	circRNA_017919	6:148109542|148138176
circRNA_019612	18:55667989|55668299	circRNA_007120	14:44349184|44357146	circRNA_020712	14:131881858|131893868	circRNA_014303	15:133542340|133570976
circRNA_003607	15:112581609|112581920	circRNA_012255	1:134518965|134526927	circRNA_003192	13:117326011|117338022	circRNA_001981	7:62801435|62830084
circRNA_008186	GL892813.1:35018|35329	circRNA_004807	7:80516201|80524164	circRNA_005072	9:19308688|19320699	circRNA_000988	15:112138368|112167019
circRNA_004070	3:24388457|24388769	circRNA_016758	12:23721321|23729286	circRNA_014615	1:255073188|255085199	circRNA_002415	X:17765641|17794304
circRNA_014589	JH118898.1:133707|134020	circRNA_014480	6:90255239|90263205	circRNA_018919	2:82165469|82177480	circRNA_001236	2:36302936|36331608
circRNA_009718	6:153309526|153309840	circRNA_021210	8:82520911|82528878	circRNA_007917	7:62090955|62102967	circRNA_004102	3:72491295|72519969
circRNA_007171	14:115462781|115463097	circRNA_010033	GL892277.2:75154|83123	circRNA_004881	8:11361730|11373743	circRNA_005919	17:35217389|35246066
circRNA_013057	3:125908209|125908525	circRNA_002336	GL893811.1:6502|14473	circRNA_005972	2:28201552|28213565	circRNA_010034	GL892277.2:90379|119059
circRNA_002868	11:450034|450351	circRNA_003378	14:77581776|77589748	circRNA_011465	16:23458294|23470307	circRNA_017940	7:91884279|91912965
circRNA_002069	8:32181173|32181491	circRNA_006349	7:80516192|80524164	circRNA_016964	17:53885029|53897046	circRNA_018975	3:58157485|58186175
circRNA_005983	2:61866062|61866380	circRNA_004586	6:82340960|82348935	circRNA_010934	X:70686832|70698850	circRNA_015399	11:53956652|53985349
circRNA_019088	5:107244098|107244416	circRNA_004130	3:84814587|84822563	circRNA_000425	11:69835593|69847612	circRNA_020465	1:247785597|247814321
circRNA_004228	4:15217343|15217662	circRNA_008206	GL894320.1:28024|36001	circRNA_011356	14:112935251|112947270	circRNA_000121	1:134632355|134661090
circRNA_010101	MT:2782|3101	circRNA_010234	1:210246828|210254806	circRNA_004408	5:13962122|13974142	circRNA_001622	4:106690698|106719435
circRNA_019757	6:57980044|57980363	circRNA_004850	7:119682039|119690020	circRNA_019999	13:43108607|43120627	circRNA_003496	15:18701372|18730119
circRNA_019944	1:267383678|267383997	circRNA_005596	11:26002135|26010117	circRNA_000020	1:17236721|17248742	circRNA_004903	8:34188701|34217450
circRNA_002945	11:86192190|86192510	circRNA_008324	1:60924835|60932817	circRNA_001989	7:69670601|69682624	circRNA_015334	10:33291015|33319767
circRNA_016778	13:28927726|28928046	circRNA_000058	1:60924835|60932819	circRNA_014783	13:90683227|90695250	circRNA_019694	4:39859616|39888370
circRNA_019573	15:111337121|111337441	circRNA_014557	9:79158926|79166910	circRNA_008382	1:143462517|143474541	circRNA_008146	9:101227171|101255938
circRNA_000207	1:227210490|227210811	circRNA_019367	1:72192716|72200701	circRNA_012762	15:116361198|116373225	circRNA_018730	14:59623322|59652089
circRNA_012356	10:33088200|33088522	circRNA_020610	13:90980638|90988624	circRNA_001054	16:36907823|36919852	circRNA_004964	8:85952805|85981573
circRNA_013846	15:69340374|69340697	circRNA_012078	9:113153990|113161978	circRNA_016741	11:56114607|56126636	circRNA_003560	15:85355707|85384479
circRNA_004021	2:138957448|138957772	circRNA_017619	13:211120471|211128459	circRNA_013445	9:32082467|32094497	circRNA_009099	16:37046960|37075733
circRNA_002305	9:143383683|143384008	circRNA_020667	14:6687380|6695370	circRNA_012296	1:212203384|212215417	circRNA_010914	GL893689.1:3069|31842
circRNA_003249	13:188218247|188218573	circRNA_007697	4:129642123|129650114	circRNA_014857	6:146057311|146069344	circRNA_017108	4:53440076|53468858
circRNA_001992	7:69988389|69988716	circRNA_004725	7:16472245|16480237	circRNA_007435	2:43933963|43945997	circRNA_005575	11:466843|495629
circRNA_003281	13:212960778|212961105	circRNA_017229	7:73108056|73116048	circRNA_012445	11:56114607|56126645	circRNA_015809	18:9534503|9563293
circRNA_004513	6:869929|870256	circRNA_005696	13:90755537|90763531	circRNA_008408	1:181043371|181055410	circRNA_005056	9:11319664|11348456
circRNA_020420	1:157504844|157505171	circRNA_013604	12:23393480|23401474	circRNA_011266	13:144274234|144286276	circRNA_011218	13:43394660|43423452
circRNA_007289	15:133851987|133852315	circRNA_002574	1:128682226|128690221	circRNA_008891	14:33552349|33564397	circRNA_021379	X:109068407|109097201
circRNA_009518	5:4840164|4840492	circRNA_001990	7:69674622|69682624	circRNA_000670	13:144109883|144121936	circRNA_007989	8:24517237|24546036
circRNA_007568	3:85291843|85292172	circRNA_020433	1:181164408|181172411	circRNA_006814	10:17555075|17567128	circRNA_009794	7:97127936|97156735
circRNA_001264	2:65415192|65415523	circRNA_012067	9:51507059|51515063	circRNA_015701	15:67048310|67060363	circRNA_001138	18:2751499|2780302
circRNA_010994	1:133185637|133185968	circRNA_017324	9:40787181|40795185	circRNA_008189	GL893036.1:49548|61604	circRNA_019815	8:32202298|32231116
circRNA_011393	15:28837231|28837562	circRNA_010467	15:22563847|22571854	circRNA_002963	12:14819804|14831861	circRNA_006827	10:37117371|37146225
circRNA_010880	9:62346488|62346821	circRNA_010315	11:53880654|53888663	circRNA_003606	15:112569863|112581920	circRNA_014346	18:44199254|44228109
circRNA_018186	15:116339014|116339347	circRNA_007606	3:121950526|121958538	circRNA_014199	13:52675780|52687838	circRNA_011046	1:227157790|227186649

circRNA_008029	8:83700344|83700678	circRNA_003317	14:30058998|30067011	circRNA_016336	8:3341344|3353402	circRNA_002165	8:145847321|145876183
circRNA_004782	7:63556778|63557113	circRNA_009828	8:30550155|30558168	circRNA_010504	16:36038379|36050438	circRNA_004212	3:134639294|134668164
circRNA_006607	1:16603165|16603500	circRNA_014451	5:57066528|57074841	circRNA_019316	GL895038.1:37303|49369	circRNA_012863	18:10771396|10800270
circRNA_015791	17:19332804|19333139	circRNA_013893	2:154050733|154058749	circRNA_017664	15:65918361|65930429	circRNA_005588	11:18665770|18694664
circRNA_020526	11:12782496|12782831	circRNA_016387	8:91914715|91922731	circRNA_017611	13:167748157|167760227	circRNA_013425	8:142091460|142120358
circRNA_005305	MT:2447|2783	circRNA_006842	10:54054021|54062038	circRNA_003194	13:117695880|117707954	circRNA_008737	13:37965105|37994009
circRNA_020942	3:29696576|29696912	circRNA_002250	9:78881650|78889668	circRNA_013511	GL892789.1:62629|74703	circRNA_011796	4:137107382|137136291
circRNA_006565	X:56081694|56082031	circRNA_003000	12:37094024|37102042	circRNA_017079	3:92608281|92620356	circRNA_001108	17:43061013|43089928
circRNA_012427	11:15729934|15730271	circRNA_004603	6:88343002|88351020	circRNA_007982	8:8759050|8771126	circRNA_008776	13:82846779|82875709
circRNA_018802	15:116266642|116266979	circRNA_011302	14:10462923|10470943	circRNA_016332	7:129597579|129609656	circRNA_002583	1:132967048|132995990
circRNA_001583	4:55349876|55350215	circRNA_016252	6:118018542|118026564	circRNA_009216	2:7617774|7629852	circRNA_006990	13:42356939|42385893
circRNA_002591	1:136392704|136393043	circRNA_002983	12:23258768|23266798	circRNA_017444	1:101708893|101720971	circRNA_000433	11:86933456|86962417
circRNA_014794	14:108265647|108265986	circRNA_005805	14:118032415|118040445	circRNA_021199	8:57798326|57810406	circRNA_007727	5:17065100|17094072
circRNA_004254	4:38937573|38937913	circRNA_016508	GL892407.1:101874|109905	circRNA_012295	1:212203332|212215417	circRNA_002129	8:110783890|110812866
circRNA_017818	4:12615332|12615672	circRNA_005182	9:132900172|132908204	circRNA_016310	7:94502841|94514929	circRNA_009934	9:36603896|36632877
circRNA_018045	X:30693504|30693844	circRNA_009796	7:97696950|97704983	circRNA_005589	11:18975972|18988063	circRNA_016103	4:117893543|117922524
circRNA_017508	1:302985522|302985863	circRNA_016462	9:51124702|51132736	circRNA_004026	2:140760854|140772947	circRNA_021157	7:32849470|32878460
circRNA_000690	13:167103118|167103460	circRNA_016866	14:95245306|95253340	circRNA_005373	X:121785129|121797223	circRNA_016975	18:10898787|10927784
circRNA_010285	10:52861856|52862198	circRNA_001115	17:45685173|45693212	circRNA_002706	1:245115123|245127219	circRNA_020748	15:87046948|87075962
circRNA_012726	15:71907134|71907476	circRNA_016026	3:117259306|117267345	circRNA_007747	5:49575425|49587521	circRNA_017908	6:132138607|132167622
circRNA_015051	8:1269897|1270239	circRNA_000046	1:45669933|45677973	circRNA_018232	2:80451220|80463317	circRNA_004799	7:73042261|73071300
circRNA_018049	X:52140097|52140439	circRNA_004052	3:11333157|11341198	circRNA_007748	5:49634023|49646121	circRNA_020435	1:181348908|181377948
circRNA_020867	2:43972990|43973332	circRNA_014515	8:3343742|3351783	circRNA_018691	13:202685143|202697241	circRNA_001193	18:57721198|57750266
circRNA_015489	13:34826944|34827287	circRNA_016095	4:109034693|109042735	circRNA_005883	16:18621775|18633876	circRNA_002440	X:121797075|121826148
circRNA_013756	17:53886898|53887242	circRNA_003715	16:50156141|50164184	circRNA_015737	15:133558875|133570976	circRNA_019884	JH118996.1:12596|41669
circRNA_018064	1:3692035|3692379	circRNA_020431	1:181116229|181124272	circRNA_012972	2:137663522|137675624	circRNA_021219	8:93279522|93308596
circRNA_021029	4:103416402|103416746	circRNA_017633	14:64345406|64353452	circRNA_000889	15:22572696|22584801	circRNA_004981	8:93279522|93308599
circRNA_007420	2:9664995|9665340	circRNA_001008	15:122869162|122877209	circRNA_007825	6:64561328|64573433	circRNA_008001	8:40572430|40601513
circRNA_012423	11:12096497|12096843	circRNA_019836	8:141381057|141389104	circRNA_004438	5:49634014|49646121	circRNA_006172	4:108244663|108273747
circRNA_019323	GL896359.1:1555|1901	circRNA_006134	4:37695059|37703107	circRNA_020380	1:59703028|59715138	circRNA_005785	14:52842304|52871390
circRNA_019387	1:169304335|169304681	circRNA_005762	14:11439831|11447880	circRNA_014229	13:210819581|210831693	circRNA_018276	4:136401988|136431078
circRNA_019501	13:160517759|160518106	circRNA_003201	13:130082229|130090279	circRNA_017630	14:57744737|57756855	circRNA_007114	14:41055275|41084368
circRNA_019557	14:144071871|144072218	circRNA_001576	4:49402905|49410956	circRNA_014278	15:52501687|52513807	circRNA_008599	11:23621098|23650191
circRNA_004788	7:65571757|65572105	circRNA_008807	13:117331417|117339469	circRNA_020175	4:73737881|73750002	circRNA_019873	GL895038.1:37303|66400
circRNA_011539	18:14099894|14100242	circRNA_021048	5:31629973|31638026	circRNA_018347	8:42865871|42877994	circRNA_006951	12:53698948|53728048
circRNA_016356	8:43069841|43070190	circRNA_004349	4:121552657|121560712	circRNA_007359	17:43098460|43110584	circRNA_016823	13:159947182|159976282
circRNA_006917	12:18987508|18987858	circRNA_014869	7:109866218|109874274	circRNA_001124	17:55235758|55247885	circRNA_005864	15:115388257|115417375
circRNA_012522	13:35455429|35455779	circRNA_004378	4:135021450|135029507	circRNA_016631	1:60893530|60905657	circRNA_019029	4:56555763|56584883
circRNA_019736	5:70270305|70270656	circRNA_005605	11:75407183|75415240	circRNA_000933	15:71703524|71715654	circRNA_015847	18:57721131|57750266
circRNA_008943	14:110425401|110425753	circRNA_019462	12:37851010|37859067	circRNA_020586	13:42286359|42298490	circRNA_020603	13:83142279|83171419
circRNA_012819	16:59904365|59904717	circRNA_001440	3:74730617|74738677	circRNA_002667	1:202644306|202656440	circRNA_000846	14:115719362|115748510
circRNA_020180	4:84484862|84485214	circRNA_017866	5:91292773|91300833	circRNA_004613	6:92090650|92102784	circRNA_010667	4:55335507|55364656
circRNA_021269	9:70218032|70218384	circRNA_015265	1:227185988|227194049	circRNA_009534	5:38344302|38356438	circRNA_012767	15:117701762|117730915
circRNA_010760	6:127502432|127502785	circRNA_018575	11:19718544|19726605	circRNA_011519	17:42006762|42018906	circRNA_000601	13:75848549|75877703
circRNA_010813	7:124821065|124821420	circRNA_011306	14:11439831|11447894	circRNA_007779	5:94439224|94451370	circRNA_003149	13:82870481|82899646
circRNA_013375	8:31681808|31682163	circRNA_000855	14:131878915|131886980	circRNA_018233	2:82188989|82201141	circRNA_008319	1:56080746|56109914
circRNA_015286	1:250128808|250129163	circRNA_012072	9:88607587|88615652	circRNA_015640	14:79059565|79071718	circRNA_002641	1:188150674|188179860
circRNA_012808	16:43232224|43232580	circRNA_019382	1:141486242|141494309	circRNA_004168	3:109447083|109459237	circRNA_018316	6:125341645|125370845
circRNA_005253	GL894642.2:8365|8722	circRNA_016182	5:99094905|99102973	circRNA_010066	GL894735.1:3944|16099	circRNA_005721	13:142599903|142629113
circRNA_010384	13:98829090|98829447	circRNA_002408	X:13331565|13339634	circRNA_010176	1:45701230|45713385	circRNA_014731	8:63565251|63594474
circRNA_005017	8:140931560|140931918	circRNA_003721	16:51821929|51829999	circRNA_004348	4:118121144|118133302	circRNA_013505	9:143209323|143238551
circRNA_001775	6:869929|870288	circRNA_006662	1:101848832|101856904	circRNA_003247	13:172805132|172817294	circRNA_007154	14:82894668|82923904

circRNA_016420	8:145215369|145215728	circRNA_019315	GL894943.1:28102|36174	circRNA_019635	2:88734312|88746479	circRNA_001490	3:109745262|109774507
circRNA_009326	3:11030041|11030401	circRNA_014395	3:72585580|72593654	circRNA_005491	1:208501653|208513821	circRNA_011852	5:105276876|105306144
circRNA_012989	3:4568871|4569231	circRNA_000140	1:143998940|144007016	circRNA_007601	3:117261346|117273517	circRNA_010421	14:11399022|11428296
circRNA_014679	2:69189833|69190193	circRNA_007611	3:122022811|122030888	circRNA_004315	4:95581648|95593820	circRNA_006007	2:87164147|87193426
circRNA_008124	9:70812448|70812810	circRNA_008242	X:21357964|21366041	circRNA_008774	13:81190634|81202807	circRNA_015211	1:134640969|134670263
circRNA_012903	2:16189245|16189607	circRNA_019693	4:16728437|16736515	circRNA_019243	9:7134810|7146985	circRNA_017592	13:51113982|51143281
circRNA_020566	12:54989431|54989793	circRNA_019789	7:32849470|32857550	circRNA_017909	6:132201517|132213693	circRNA_014187	13:12413791|12443091
circRNA_007323	16:50765133|50765496	circRNA_015006	15:133531311|133539392	circRNA_002161	8:144641851|144654028	circRNA_001935	6:154523123|154552424
circRNA_011320	14:33762085|33762448	circRNA_015614	14:53336077|53344161	circRNA_010985	1:124772986|124785165	circRNA_015109	X:101973310|102002622
circRNA_011822	5:57093473|57093836	circRNA_009995	9:101476729|101484815	circRNA_008563	10:63625465|63637645	circRNA_014948	9:13674798|13704113
circRNA_007257	15:85209680|85210044	circRNA_006527	GL893957.1:794|8881	circRNA_018716	14:34693433|34705614	circRNA_002431	X:85723095|85752413
circRNA_017561	12:23468473|23468837	circRNA_011404	15:61774735|61782824	circRNA_015016	18:5622335|5634518	circRNA_007043	13:126270468|126299787
circRNA_002914	11:32036344|32036709	circRNA_002598	1:142958354|142966444	circRNA_005375	X:121793875|121806061	circRNA_000206	1:227181487|227210811
circRNA_011823	5:63725747|63726112	circRNA_017480	1:195112533|195120623	circRNA_014238	14:34693427|34705614	circRNA_015271	1:229799248|229828576
circRNA_012747	15:104097152|104097517	circRNA_020836	18:27440896|27448992	circRNA_018589	12:15250677|15262868	circRNA_012710	15:44253492|44282828
circRNA_010585	2:106419586|106419952	circRNA_015825	18:19941902|19949999	circRNA_015850	18:59549924|59562118	circRNA_008262	X:85723095|85752440
circRNA_014675	2:12197294|12197660	circRNA_006930	12:27608857|27616955	circRNA_020846	18:44795192|44807387	circRNA_018387	GL894982.1:2829|32184
circRNA_011783	4:98306908|98307275	circRNA_014552	9:49676527|49684625	circRNA_002078	8:39365193|39377393	circRNA_020353	X:35465814|35495178
circRNA_012938	2:77619456|77619823	circRNA_018044	X:17742124|17750223	circRNA_015551	13:142931343|142943544	circRNA_000370	10:77690752|77720129
circRNA_015310	1:293503530|293503898	circRNA_016984	18:20913412|20921513	circRNA_015564	13:155842598|155854800	circRNA_021222	8:111990593|112019970
circRNA_004317	4:96968371|96968740	circRNA_017593	13:67539381|67547482	circRNA_018781	15:62963006|62975208	circRNA_000625	13:98162592|98191973
circRNA_011696	3:75950705|75951076	circRNA_017893	6:83154360|83162461	circRNA_017780	2:157390156|157402360	circRNA_017910	6:137375783|137405178
circRNA_020780	16:27110482|27110853	circRNA_006033	2:120422878|120430980	circRNA_000572	13:38002797|38015007	circRNA_007578	3:92579061|92608458
circRNA_009754	7:59876012|59876384	circRNA_004723	7:14663497|14671601	circRNA_001291	2:86306052|86318262	circRNA_005770	14:30050907|30080315
circRNA_011530	17:57233648|57234021	circRNA_005609	12:315609|323714	circRNA_017034	2:98820974|98833184	circRNA_001206	2:1487673|1517085
circRNA_012135	GL896427.1:1964|2337	circRNA_010232	1:208219319|208227424	circRNA_003050	12:55913418|55925631	circRNA_015116	12:48558810|48588228
circRNA_018623	13:23653601|23653974	circRNA_017114	4:60005466|60013573	circRNA_019142	7:125461|137678	circRNA_002969	12:16041804|16071225
circRNA_006552	MT:3033|3408	circRNA_005935	18:834115|842224	circRNA_013523	GL893953.2:81861|94086	circRNA_016176	5:88158511|88187943
circRNA_008857	13:207199797|207200173	circRNA_015350	10:46711919|46720028	circRNA_018838	16:78165123|78177348	circRNA_004202	3:121683590|121713024
circRNA_010803	7:80266702|80267078	circRNA_005271	GL896137.1:66267|74377	circRNA_006981	13:33210204|33222430	circRNA_009186	18:19920552|19949999
circRNA_001368	2:147434535|147434915	circRNA_016191	6:14369022|14377132	circRNA_007550	3:46748462|46760689	circRNA_006002	2:85460738|85490186
circRNA_013459	9:49790828|49791208	circRNA_002223	9:44284359|44292473	circRNA_019032	4:68358494|68370721	circRNA_002888	11:13305769|13335221
circRNA_016861	14:82062422|82062803	circRNA_003351	14:50023889|50032008	circRNA_009029	15:85065639|85077868	circRNA_002830	10:50688302|50717769
circRNA_017763	2:83313668|83314049	circRNA_018140	13:147202724|147210843	circRNA_014512	7:120152807|120165036	circRNA_018672	13:154082898|154112366
circRNA_020337	9:143414817|143415198	circRNA_014195	13:42311419|42319539	circRNA_019565	15:66053781|66066010	circRNA_019455	11:86932930|86962417
circRNA_003940	2:83206943|83207326	circRNA_015978	3:60073410|60081530	circRNA_008139	9:85909359|85921589	circRNA_019250	9:19263852|19293342
circRNA_010104	MT:3378|3761	circRNA_005900	16:51837295|51845416	circRNA_001375	2:157941303|157953538	circRNA_011257	13:131093962|131123453
circRNA_017777	2:145502702|145503085	circRNA_004313	4:90702984|90711107	circRNA_005064	9:13306787|13319023	circRNA_017151	5:88023420|88052914
circRNA_020048	14:118455496|118455879	circRNA_007912	7:54579388|54587514	circRNA_006035	2:121998264|122010500	circRNA_018231	2:79850166|79879663
circRNA_008234	MT:9816|10200	circRNA_002358	GL895028.1:36544|44671	circRNA_020068	15:128558331|128570567	circRNA_019179	7:91952612|91982115
circRNA_009229	2:29213178|29213562	circRNA_019721	5:38348311|38356438	circRNA_008986	15:22565123|22577367	circRNA_020166	4:38289685|38319194
circRNA_008900	14:45619419|45619804	circRNA_007103	14:21988029|21996158	circRNA_009941	9:39970551|39982797	circRNA_014678	2:49882526|49912043
circRNA_011457	15:152395889|152396274	circRNA_014364	2:87964030|87972159	circRNA_020390	1:80320770|80333018	circRNA_001923	6:148040878|148070397
circRNA_016294	7:57602686|57603071	circRNA_020885	2:81112457|81120589	circRNA_014896	X:12339635|12351884	circRNA_011915	6:148040878|148070402
circRNA_017764	2:83801388|83801773	circRNA_011323	14:41725329|41733465	circRNA_013747	13:97302620|97314870	circRNA_020486	1:298098190|298127716
circRNA_001265	2:70193426|70193812	circRNA_014421	4:61279291|61287431	circRNA_016419	8:144739680|144751930	circRNA_018604	12:44004809|44034345
circRNA_013165	5:43365469|43365855	circRNA_018240	2:134953156|134961296	circRNA_014654	14:46217501|46229752	circRNA_005978	2:52235687|52265225
circRNA_015526	13:90886399|90886787	circRNA_001691	5:19105763|19113904	circRNA_008069	8:144739677|144751930	circRNA_010654	4:33431196|33460740
circRNA_004957	8:80945069|80945458	circRNA_019405	1:251442836|251450977	circRNA_006909	12:11470995|11483250	circRNA_018810	15:144725781|144755326
circRNA_011004	1:141115360|141115749	circRNA_020097	18:20228064|20236208	circRNA_011420	15:86607064|86619319	circRNA_008240	X:17758636|17788187
circRNA_012438	11:50756015|50756404	circRNA_019212	8:75542191|75550336	circRNA_011657	3:11507278|11519534	circRNA_002061	8:12379709|12409262
circRNA_001635	4:116017070|116017460	circRNA_003263	13:210042276|210050422	circRNA_019234	8:134712877|134725134	circRNA_017148	5:74569912|74599470

circRNA_002273	9:101923667|101924057	circRNA_014394	3:72456612|72464759	circRNA_008150	9:102919251|102931512	circRNA_015854	2:2992758|3022317
circRNA_005286	JH118489.1:81186|81576	circRNA_015110	X:122545608|122553757	circRNA_008822	13:141962644|141974905	circRNA_001172	18:18686159|18715721
circRNA_002932	11:68999509|68999900	circRNA_012244	1:121457027|121465177	circRNA_007763	5:74533338|74545601	circRNA_012036	9:6916447|6946013
circRNA_021110	6:85341893|85342284	circRNA_003898	2:36235515|36243672	circRNA_012212	1:55298663|55310926	circRNA_004462	5:70617986|70647555
circRNA_001728	5:63725719|63726112	circRNA_000938	15:73508752|73516910	circRNA_019044	4:111297330|111309596	circRNA_016431	9:11771734|11801305
circRNA_007078	13:170277812|170278205	circRNA_009299	2:128516665|128524824	circRNA_008513	10:17369643|17381911	circRNA_020028	14:474755|504329
circRNA_007717	5:6890151|6890544	circRNA_013424	8:142057485|142065646	circRNA_000487	12:39217481|39229750	circRNA_017492	1:231210010|231239599
circRNA_010788	7:40392366|40392759	circRNA_009175	18:10792104|10800270	circRNA_006843	10:54112639|54124909	circRNA_006210	5:61475230|61504828
circRNA_019334	MT:14238|14631	circRNA_015612	14:52802367|52810536	circRNA_018982	3:74760760|74773032	circRNA_005761	14:10633685|10663285
circRNA_002116	8:88543731|88544126	circRNA_001746	5:74537431|74545601	circRNA_014493	7:64736082|64748355	circRNA_005709	13:126270184|126299787
circRNA_008675	12:44687527|44687922	circRNA_002728	1:265189347|265197520	circRNA_000266	1:300139455|300151730	circRNA_004926	8:57375305|57404915
circRNA_016830	13:205198842|205199237	circRNA_015194	1:101861737|101869910	circRNA_003029	12:45421101|45433380	circRNA_018892	18:59666431|59696045
circRNA_017019	2:58424319|58424714	circRNA_005306	MT:3038|11212	circRNA_000402	11:22229047|22241327	circRNA_004371	4:129763283|129792899
circRNA_019357	1:14077764|14078159	circRNA_011549	18:36472459|36480633	circRNA_015601	14:34717481|34729763	circRNA_003403	14:83655944|83685562
circRNA_013082	4:38240989|38241385	circRNA_010431	14:33994382|34002557	circRNA_000088	1:116736713|116748998	circRNA_016768	12:47804289|47833910
circRNA_016004	3:98016245|98016641	circRNA_017621	14:10483485|10491663	circRNA_010679	4:96774978|96787266	circRNA_010370	13:53872565|53902188
circRNA_018397	X:67429473|67429869	circRNA_016315	7:102205505|102213687	circRNA_013356	7:120262186|120274480	circRNA_012635	14:34675990|34705614
circRNA_007117	14:42969793|42970190	circRNA_005077	9:22402298|22410483	circRNA_007566	3:81720523|81732824	circRNA_007181	14:122429376|122459005
circRNA_019266	9:49290024|49290421	circRNA_006872	11:21089411|21097597	circRNA_000701	13:200425058|200437361	circRNA_010049	GL893009.1:66223|95865
circRNA_019410	1:280648787|280649184	circRNA_019303	GL892813.1:30504|38690	circRNA_004487	5:88193297|88205602	circRNA_018362	9:3153451|3183098
circRNA_002350	GL894584.1:42613|43011	circRNA_010519	17:5409813|5418002	circRNA_019463	12:40721518|40733832	circRNA_002149	8:134379373|134409025
circRNA_018081	1:103512707|103513105	circRNA_010402	13:155644173|155652364	circRNA_018307	6:64478265|64490580	circRNA_009318	2:150630673|150660330
circRNA_018299	6:871859|872257	circRNA_011693	3:73352373|73360565	circRNA_020230	6:102021939|102034254	circRNA_006858	11:472454|502116
circRNA_021060	5:70045596|70045994	circRNA_002928	11:55451765|55459959	circRNA_009494	4:121741759|121754075	circRNA_012378	10:53346989|53376653
circRNA_000903	15:37995043|37995443	circRNA_008148	9:101484691|101492888	circRNA_009301	2:128783620|128795937	circRNA_015351	10:47064149|47093826
circRNA_002559	1:118608831|118609231	circRNA_016299	7:70033347|70041544	circRNA_011511	17:20636979|20649297	circRNA_012589	13:148879705|148909385
circRNA_011233	13:80698984|80699384	circRNA_020892	2:85683347|85691545	circRNA_001883	6:102021935|102034254	circRNA_012794	16:23698631|23728315
circRNA_015097	5:18745419|18745819	circRNA_010416	13:210039602|210047801	circRNA_017044	2:128516665|128528985	circRNA_004274	4:55349876|55379564
circRNA_019960	10:37465102|37465502	circRNA_014314	16:47809258|47817458	circRNA_010516	17:5159655|5171977	circRNA_014426	4:72446101|72475797
circRNA_005564	10:56100870|56101271	circRNA_009113	16:53279948|53288151	circRNA_018149	14:31064919|31077243	circRNA_016699	1:283916128|283945829
circRNA_008798	13:97393646|97394047	circRNA_011025	1:181161592|181169796	circRNA_010153	X:121922443|121934769	circRNA_019550	14:120758678|120788379
circRNA_010389	13:119602088|119602489	circRNA_011968	7:102712011|102720215	circRNA_012123	GL894597.1:33450|45776	circRNA_018101	10:17369643|17399345
circRNA_021376	X:97848584|97848985	circRNA_011980	7:125280803|125289007	circRNA_007750	5:50820506|50832839	circRNA_005951	18:36505709|36535413
circRNA_009622	6:26462255|26462658	circRNA_019914	1:84250378|84258582	circRNA_012507	13:3080251|3092588	circRNA_007801	6:32195144|32224859
circRNA_016602	X:101913526|101913929	circRNA_020619	13:122641855|122650059	circRNA_008133	9:79212011|79224350	circRNA_018811	15:146530961|146560684
circRNA_016724	10:45905499|45905902	circRNA_004803	7:73226911|73235116	circRNA_009874	8:103699742|103712082	circRNA_004151	3:101043292|101073016
circRNA_018226	2:65418293|65418696	circRNA_006010	2:87837872|87846078	circRNA_004468	5:73508181|73520522	circRNA_020706	14:119374779|119404503
circRNA_007364	17:51437349|51437753	circRNA_008035	8:91639395|91647603	circRNA_001597	4:79089337|79101679	circRNA_007186	14:124736001|124765738
circRNA_016771	12:50456375|50456779	circRNA_006779	1:280497623|280505832	circRNA_009432	4:16767207|16779552	circRNA_009884	8:110762091|110791848
circRNA_021061	5:70496529|70496933	circRNA_017724	17:47067112|47075323	circRNA_000671	13:144391256|144403607	circRNA_013521	GL893884.1:17986|47747
circRNA_017915	6:145342909|145343315	circRNA_014707	4:139715852|139724066	circRNA_006063	3:19040182|19052536	circRNA_019743	5:105247263|105277027
circRNA_018894	2:5806970|5807376	circRNA_005987	2:73893292|73901507	circRNA_020818	17:61188071|61200427	circRNA_011023	1:161752061|161781848
circRNA_013463	9:63133898|63134305	circRNA_015221	1:176386093|176394308	circRNA_020330	9:112777872|112790229	circRNA_006610	1:18496821|18526616
circRNA_000446	12:12368345|12368753	circRNA_007097	14:13477887|13486104	circRNA_004157	3:104182825|104195188	circRNA_020841	18:41987764|42017561
circRNA_013308	7:21868824|21869232	circRNA_003880	2:8286557|8294777	circRNA_000516	12:60937061|60949425	circRNA_010959	1:25510519|25540318
circRNA_019707	4:116757994|116758403	circRNA_001771	5:106601856|106610077	circRNA_012477	12:23393480|23405847	circRNA_017800	3:76077846|76107647
circRNA_002726	1:262203573|262203983	circRNA_010427	14:31364465|31372689	circRNA_005200	9:143299432|143311801	circRNA_019637	2:91899706|91929512
circRNA_006467	9:37038282|37038692	circRNA_012066	9:51467096|51475324	circRNA_018889	18:49466484|49478855	circRNA_005235	GL893811.1:6502|36310
circRNA_012759	15:116124302|116124713	circRNA_012998	3:29650912|29659144	circRNA_000530	13:12197595|12209968	circRNA_015332	10:30404229|30434042
circRNA_018050	X:55498696|55499107	circRNA_003101	13:35647658|35655895	circRNA_009179	18:11035272|11047645	circRNA_015622	14:61603585|61633400
circRNA_005973	2:29219247|29219660	circRNA_014522	8:32892981|32901219	circRNA_017010	2:20507747|20520121	circRNA_005719	13:142522109|142551946
circRNA_014517	8:20528052|20528465	circRNA_017754	2:53072052|53080290	circRNA_013166	5:46892191|46904567	circRNA_004464	5:70663866|70693708

circRNA_014848	6:40797702|40798115	circRNA_001290	2:86283747|86291987	circRNA_009214	2:455352|467729	circRNA_014909	1:283070491|283100341
circRNA_018310	6:79400081|79400494	circRNA_005117	9:50825051|50833295	circRNA_007287	15:133614874|133627254	circRNA_003135	13:72802675|72832527
circRNA_004864	7:130879957|130880371	circRNA_013880	2:50800152|50808396	circRNA_009970	9:51067117|51079497	circRNA_011063	1:270414452|270444314
circRNA_011210	13:34583991|34584405	circRNA_016996	2:450748|458994	circRNA_016781	13:34698244|34710627	circRNA_017173	6:62401029|62430900
circRNA_020677	14:43100107|43100522	circRNA_020822	18:814124|822370	circRNA_020387	1:63729107|63741490	circRNA_010726	5:106543058|106572931
circRNA_010203	1:127641289|127641705	circRNA_003141	13:77761973|77770223	circRNA_012749	15:106358782|106371170	circRNA_004699	6:148034940|148064823
circRNA_001339	2:123502993|123503411	circRNA_006813	10:17067380|17075630	circRNA_019114	6:92042985|92055373	circRNA_018521	1:282166095|282195982
circRNA_015107	JH118995.1:394|812	circRNA_016562	JH118545.1:30364|38614	circRNA_000682	13:158525065|158537462	circRNA_011524	17:45646799|45676687
circRNA_016879	14:131867981|131868399	circRNA_017100	4:37751367|37759621	circRNA_004422	5:33833674|33846071	circRNA_017667	15:71715508|71745408
circRNA_004756	7:37471724|37472143	circRNA_016048	4:15229913|15238168	circRNA_004434	5:42104599|42116997	circRNA_019171	7:73063995|73093908
circRNA_013778	1:181330431|181330850	circRNA_012906	2:17762810|17771066	circRNA_004436	5:47663197|47675595	circRNA_006532	GL894965.2:19005|48924
circRNA_017101	4:39143287|39143706	circRNA_014322	16:68960001|68968257	circRNA_007361	17:45685173|45697572	circRNA_012885	18:48576462|48606383
circRNA_010200	1:120220840|120221260	circRNA_008748	13:44064993|44073250	circRNA_017486	1:208222667|208235067	circRNA_008008	8:50626727|50656654
circRNA_011703	3:84971796|84972217	circRNA_001186	18:43744959|43753217	circRNA_005711	13:127421316|127433717	circRNA_014968	1:230910210|230940137
circRNA_016371	8:57909334|57909755	circRNA_001338	2:122626814|122635072	circRNA_012124	GL894729.2:5713|18116	circRNA_007349	17:36419116|36449044
circRNA_017816	3:135350241|135350662	circRNA_004206	3:123743295|123751557	circRNA_013199	5:91245793|91258197	circRNA_001910	6:139872924|139902854
circRNA_004772	7:58507983|58508405	circRNA_015140	1:16119378|16127640	circRNA_013525	GL894019.2:16864|29269	circRNA_012190	1:23213758|23243695
circRNA_006167	4:103267690|103268112	circRNA_019302	GL892789.1:86305|94569	circRNA_013503	9:138768104|138780511	circRNA_017294	8:134379373|134409317
circRNA_019870	GL894320.1:35579|36001	circRNA_000943	15:73910689|73918954	circRNA_019175	7:89005630|89018039	circRNA_017997	9:79880267|79910220
circRNA_001795	6:43177209|43177632	circRNA_013053	3:121830635|121838900	circRNA_016489	9:101480476|101492888	circRNA_014876	8:49949834|49979796
circRNA_008856	13:206958569|206958992	circRNA_006120	3:134703415|134711682	circRNA_001313	2:98820974|98833387	circRNA_015694	15:61862385|61892350
circRNA_020625	13:131374931|131375354	circRNA_010406	13:167174462|167182729	circRNA_005310	X:7258915|7271328	circRNA_008259	X:73939459|73969431
circRNA_015985	3:73882214|73882638	circRNA_014993	14:33395790|33404059	circRNA_003144	13:80690197|80702612	circRNA_011776	4:73920402|73950380
circRNA_018944	2:132404785|132405209	circRNA_014601	X:109113838|109122109	circRNA_010347	12:59907022|59919437	circRNA_012826	17:1345076|1375056
circRNA_008387	1:146912898|146913323	circRNA_013795	10:45915306|45923578	circRNA_012319	1:282216219|282228639	circRNA_002080	8:40321067|40351050
circRNA_015122	15:106502652|106503077	circRNA_020773	16:4411036|4419308	circRNA_014689	2:128512404|128524824	circRNA_008248	X:43159977|43189968
circRNA_018371	9:63133898|63134323	circRNA_008114	9:50848480|50856753	circRNA_020366	1:16327908|16340328	circRNA_005487	1:205444963|205474960
circRNA_006241	5:97653518|97653944	circRNA_014514	7:130875508|130883781	circRNA_002294	9:133331935|133344356	circRNA_012073	9:88630066|88660063
circRNA_018102	10:19736916|19737342	circRNA_018261	4:45007707|45015985	circRNA_001005	15:120872321|120884747	circRNA_007694	4:129256921|129286926
circRNA_006062	3:18074971|18075398	circRNA_020488	1:299970045|299978323	circRNA_007250	15:75681177|75693603	circRNA_012578	13:119184171|119214179
circRNA_019928	1:152083770|152084197	circRNA_018711	14:31064919|31073198	circRNA_008876	14:12682156|12694583	circRNA_020421	1:157849877|157879888
circRNA_002783	10:21700951|21701379	circRNA_009220	2:11070983|11079263	circRNA_018285	5:32956398|32968826	circRNA_001169	18:18671313|18701330
circRNA_010188	1:75139902|75140330	circRNA_000869	14:145123173|145131457	circRNA_019407	1:263633167|263645595	circRNA_017226	7:62884149|62914166
circRNA_020190	5:690244|690672	circRNA_006454	9:19215967|19224251	circRNA_012954	2:91113063|91125493	circRNA_010790	7:57257302|57287321
circRNA_011707	3:91092657|91093086	circRNA_000743	14:16173206|16181494	circRNA_014085	7:10291894|10304324	circRNA_014798	15:35514849|35544893
circRNA_001599	4:85304081|85304511	circRNA_019106	6:77945423|77953711	circRNA_006457	9:22384694|22397126	circRNA_014293	15:86579482|86609530
circRNA_008115	9:50883377|50883807	circRNA_003889	2:19750076|19758365	circRNA_016574	X:14657985|14670417	circRNA_020851	18:60636455|60666503
circRNA_014005	14:45702404|45702834	circRNA_006360	7:102696376|102704665	circRNA_004798	7:72903666|72916101	circRNA_006368	7:119763701|119793755
circRNA_018225	2:61929824|61930254	circRNA_014525	8:58297279|58305568	circRNA_016175	5:88016428|88028864	circRNA_010177	1:45734305|45764360
circRNA_003927	2:77288091|77288522	circRNA_018069	1:16870879|16879168	circRNA_011516	17:36729287|36741725	circRNA_012898	2:3009236|3039309
circRNA_016138	5:36462332|36462763	circRNA_010963	1:40737383|40745673	circRNA_014636	13:42346716|42359154	circRNA_014297	15:101654960|101685033
circRNA_019578	15:134204505|134204938	circRNA_020109	2:3368705|3376995	circRNA_011631	2:121549319|121561759	circRNA_007767	5:80902859|80932933
circRNA_005127	9:67438738|67439173	circRNA_003339	14:41753683|41761976	circRNA_003617	15:117690764|117703208	circRNA_009597	5:99032220|99062303
circRNA_012196	1:30979937|30980372	circRNA_011313	14:30050907|30059201	circRNA_010117	X:44667590|44680038	circRNA_011484	16:36889764|36919852
circRNA_004279	4:56565505|56565941	circRNA_020274	7:116999334|117007628	circRNA_012395	10:64310358|64322806	circRNA_007700	4:136071929|136102022
circRNA_019745	6:870262|870698	circRNA_001824	6:74972541|74980836	circRNA_005842	15:67009488|67021943	circRNA_014830	3:98087301|98117395
circRNA_005509	1:241935890|241936327	circRNA_012243	1:121374839|121383134	circRNA_016949	16:75044687|75057142	circRNA_007011	13:83142279|83172376
circRNA_010351	13:8103505|8103942	circRNA_007768	5:82659439|82667737	circRNA_015853	2:446538|458994	circRNA_020682	14:57864008|57894112
circRNA_017883	6:56005690|56006127	circRNA_017220	7:48098507|48106808	circRNA_019701	4:72916723|72929179	circRNA_011318	14:31937681|31967787
circRNA_001857	6:98763605|98764043	circRNA_020895	2:87542278|87550579	circRNA_011200	13:19755935|19768395	circRNA_011367	14:116995830|117025945
circRNA_016186	6:871815|872253	circRNA_005051	9:9303312|9311614	circRNA_006761	1:244821954|244834415	circRNA_014942	6:145384185|145414300
circRNA_000819	14:82889383|82889822	circRNA_002839	10:57355468|57363777	circRNA_014272	15:18327032|18339494	circRNA_009838	8:40313491|40343610

circRNA_005352	X:78096984|78097423	circRNA_004796	7:69874055|69882364	circRNA_000210	1:229233921|229246387	circRNA_008274	X:121793875|121823999
circRNA_015915	2:121571724|121572163	circRNA_002611	1:148284301|148292614	circRNA_011301	14:7185050|7197518	circRNA_020357	X:70818283|70848414
circRNA_004415	5:22747527|22747967	circRNA_007887	6:148414520|148422837	circRNA_018246	3:10365569|10378037	circRNA_010627	3:90691702|90721844
circRNA_014124	1:176855013|176855454	circRNA_018418	1:60931627|60939946	circRNA_010171	1:32080657|32093129	circRNA_006693	1:143396359|143426509
circRNA_014954	GL896327.1:295|737	circRNA_005466	1:175580337|175588657	circRNA_015096	4:96074696|96087170	circRNA_011125	10:59635863|59666014
circRNA_016257	6:143777569|143778011	circRNA_012664	14:77690784|77699105	circRNA_012959	2:104867971|104880447	circRNA_000042	1:40737383|40767556
circRNA_003162	13:86954020|86954463	circRNA_014235	14:30929985|30938306	circRNA_016203	6:38136166|38148642	circRNA_005974	2:39869318|39899493
circRNA_005480	1:200721530|200721973	circRNA_015094	3:134981592|134989914	circRNA_016240	6:98736019|98748495	circRNA_006762	1:245565427|245595602
circRNA_013007	3:55592917|55593361	circRNA_009784	7:89009716|89018039	circRNA_011377	14:132237197|132249674	circRNA_018022	GL893593.1:26429|56607
circRNA_020257	7:45100111|45100555	circRNA_010085	GL896447.1:76245|84568	circRNA_012454	12:289311|301791	circRNA_017009	2:17755529|17785714
circRNA_009025	15:79683656|79684102	circRNA_013286	6:147153527|147161851	circRNA_002127	8:109625320|109637802	circRNA_013883	2:52797534|52827726
circRNA_016657	1:145008366|145008813	circRNA_018283	5:24476594|24484918	circRNA_014544	9:13722986|13735468	circRNA_019163	7:61606817|61637010
circRNA_002515	1:64113130|64113579	circRNA_012594	13:156888783|156897108	circRNA_006464	9:36060450|36072933	circRNA_018563	11:314022|344218
circRNA_016836	14:27801989|27802438	circRNA_012884	18:46750544|46758871	circRNA_016000	3:84395797|84408282	circRNA_000485	12:38771647|38801846
circRNA_019814	8:32121299|32121749	circRNA_015961	3:29964563|29972893	circRNA_012777	15:133519080|133531566	circRNA_010432	14:34671930|34702143
circRNA_019326	GL896501.1:23267|23718	circRNA_016444	9:29836166|29844499	circRNA_009340	3:29625387|29637874	circRNA_004968	8:87993468|88023699
circRNA_000918	15:55506079|55506531	circRNA_006398	8:43018351|43026689	circRNA_009545	5:52582222|52594709	circRNA_018315	6:99514655|99544888
circRNA_004064	3:18698106|18698559	circRNA_018251	3:24612720|24621058	circRNA_003875	2:3097364|3109853	circRNA_019585	16:27060292|27090533
circRNA_015824	18:18696912|18697365	circRNA_011193	13:2727564|2735905	circRNA_005042	9:7122453|7134943	circRNA_016335	8:3318209|3348457
circRNA_021319	GL894597.1:44194|44647	circRNA_012167	X:113412803|113421145	circRNA_019311	GL893884.1:35252|47747	circRNA_009751	7:56641681|56671934
circRNA_012934	2:72147111|72147566	circRNA_009188	18:26401624|26409969	circRNA_012416	11:4057924|4070420	circRNA_009909	8:146911444|146941701
circRNA_014913	12:59837742|59838198	circRNA_019356	X:142774098|142782443	circRNA_015067	JH118611.1:2203|14701	circRNA_008802	13:103789411|103819693
circRNA_009529	5:32067783|32068240	circRNA_008231	JH118963.1:60762|69109	circRNA_002946	11:86942801|86955303	circRNA_013191	5:86829708|86859993
circRNA_016185	6:870262|870719	circRNA_012654	14:60127644|60135991	circRNA_013374	8:31474913|31487416	circRNA_016153	5:61457868|61488161
circRNA_017180	6:86652525|86652983	circRNA_001121	17:49163449|49171800	circRNA_007924	7:69670117|69682624	circRNA_003540	15:66933760|66964066
circRNA_018302	6:14354902|14355360	circRNA_002880	11:8728917|8737268	circRNA_007473	2:83206943|83219454	circRNA_006861	11:6772276|6802586
circRNA_006533	GL895121.2:32321|32780	circRNA_012382	10:54165734|54174085	circRNA_007829	6:73225179|73237691	circRNA_010532	17:59454683|59484993
circRNA_013141	4:139828383|139828842	circRNA_003117	13:43417778|43426130	circRNA_002553	1:114109375|114121891	circRNA_001902	6:132124985|132155299
circRNA_003025	12:44665244|44665704	circRNA_008915	14:60129291|60137643	circRNA_001424	3:55588291|55600809	circRNA_010715	5:83051935|83082256
circRNA_017287	8:103926820|103927281	circRNA_017023	2:80568943|80577295	circRNA_005917	17:20669421|20681939	circRNA_015543	13:127837474|127867797
circRNA_019470	13:3594619|3595080	circRNA_005154	9:115691139|115699492	circRNA_014116	1:132962462|132974980	circRNA_014164	10:66012330|66042667
circRNA_009122	16:74325935|74326397	circRNA_002584	1:134599618|134607972	circRNA_017607	13:138043750|138056269	circRNA_009561	5:70565540|70595881
circRNA_009316	2:148351052|148351514	circRNA_018317	6:125857136|125865492	circRNA_001954	7:27922038|27934559	circRNA_015063	9:102020372|102050715
circRNA_000785	14:59079242|59079705	circRNA_021203	8:71347911|71356267	circRNA_021204	8:71474268|71486789	circRNA_001611	4:93598992|93629339
circRNA_004328	4:103384379|103384842	circRNA_017146	5:73401184|73409542	circRNA_007084	13:205982161|205994684	circRNA_004390	4:139855414|139885766
circRNA_012712	15:45466934|45467397	circRNA_013205	5:94470561|94478927	circRNA_008187	GL892857.2:22367|34891	circRNA_008167	9:128160907|128191259
circRNA_006251	6:16370604|16371068	circRNA_018885	18:42821896|42830262	circRNA_018818	16:23637247|23649775	circRNA_002523	1:82801008|82831362
circRNA_012914	2:31134072|31134536	circRNA_017086	3:114077581|114085951	circRNA_011330	14:57689330|57701859	circRNA_020269	7:95436160|95466527
circRNA_014383	3:25745007|25745471	circRNA_001812	6:64511470|64519841	circRNA_008359	1:124814761|124827295	circRNA_019549	14:117630383|117660757
circRNA_018634	13:55507972|55508436	circRNA_008191	GL893394.1:46818|55189	circRNA_011449	15:133531311|133543845	circRNA_018682	13:172774839|172805226
circRNA_020731	15:55547709|55548173	circRNA_009627	6:39320647|39329022	circRNA_013000	3:38637515|38650049	circRNA_005728	13:144555700|144586089
circRNA_012647	14:53989825|53990290	circRNA_015724	15:117366653|117375028	circRNA_014885	9:32422597|32435131	circRNA_003402	14:82864427|82894828
circRNA_014734	8:89347874|89348339	circRNA_005476	1:199962719|199971098	circRNA_000188	1:205472022|205484559	circRNA_011109	10:47056133|47086534
circRNA_014236	14:32976357|32976823	circRNA_007452	2:65595365|65603744	circRNA_000519	12:62435246|62447784	circRNA_018578	11:25529781|25560191
circRNA_015032	3:106171453|106171919	circRNA_013673	13:28828413|28836794	circRNA_020439	1:186637728|186650267	circRNA_008540	10:49655220|49685631
circRNA_020461	1:242706609|242707075	circRNA_007115	14:41900365|41908747	circRNA_016016	3:109719710|109732253	circRNA_009098	16:36895597|36926011
circRNA_004379	4:135596014|135596481	circRNA_016685	1:242080071|242088453	circRNA_002602	1:143279275|143291819	circRNA_007553	3:55892340|55922761
circRNA_015315	1:299801167|299801634	circRNA_003086	13:28828409|28836794	circRNA_012115	GL893671.2:10789|23333	circRNA_005921	17:36698962|36729385
circRNA_018298	6:869787|870254	circRNA_014785	13:117698446|117706831	circRNA_017301	8:140242839|140255386	circRNA_016093	4:103237684|103268112
circRNA_006648	1:92286461|92286929	circRNA_009162	17:49628660|49637047	circRNA_014760	1:75089371|75101925	circRNA_003733	16:75043882|75074322
circRNA_007451	2:65119742|65120211	circRNA_011314	14:30064916|30073303	circRNA_000941	15:73900098|73912653	circRNA_010705	5:55357121|55387578
circRNA_015025	2:118371400|118371870	circRNA_013754	10:46134653|46143041	circRNA_000746	14:16177975|16190532	circRNA_009394	3:104169248|104199715

circRNA_010923	GL896447.1:54004|54475	circRNA_013761	X:109068407|109076797	circRNA_000924	15:66249470|66262027	circRNA_017245	7:124952565|124983041
circRNA_015759	16:38539919|38540390	circRNA_006089	3:92448986|92457377	circRNA_008013	8:58349146|58361703	circRNA_015531	13:90996579|91027069
circRNA_011655	3:10658314|10658786	circRNA_009234	2:42089530|42097921	circRNA_018707	14:30965816|30978374	circRNA_006691	1:143245293|143275786
circRNA_013146	5:3834616|3835088	circRNA_001117	17:46533185|46541578	circRNA_008425	1:200530333|200542898	circRNA_006684	1:134770024|134800523
circRNA_005078	9:22678235|22678709	circRNA_005690	13:81426923|81435317	circRNA_015665	14:127901235|127913800	circRNA_003082	13:28146354|28176864
circRNA_005230	GL893329.2:19554|20029	circRNA_020660	13:212940716|212949113	circRNA_010414	13:206272126|206284694	circRNA_016300	7:73081330|73111840
circRNA_000831	14:98578456|98578932	circRNA_006978	13:28828396|28836794	circRNA_009996	9:102416245|102428814	circRNA_009312	2:142522062|142552577
circRNA_004563	6:69449174|69449650	circRNA_018854	17:45377174|45385573	circRNA_009699	6:137387063|137399634	circRNA_008456	1:242706609|242737129
circRNA_007098	14:14027675|14028151	circRNA_005272	GL896250.1:7783|16183	circRNA_008307	1:37134829|37147403	circRNA_010262	10:16947860|16978389
circRNA_013076	4:19821065|19821541	circRNA_014130	1:202650920|202659321	circRNA_000688	13:167096198|167108777	circRNA_004776	7:62812462|62843001
circRNA_016208	6:40917242|40917718	circRNA_014139	1:233145796|233154197	circRNA_012895	2:67323|79903	circRNA_003655	15:157067652|157098198
circRNA_017505	1:299554717|299555194	circRNA_015376	11:18581458|18589861	circRNA_017553	11:72118477|72131057	circRNA_003008	12:37398861|37429409
circRNA_000898	15:34770888|34771366	circRNA_013858	15:144918068|144926472	circRNA_008175	9:138767928|138780511	circRNA_009038	15:91077981|91108565
circRNA_019211	8:57909334|57909812	circRNA_020840	18:41987764|41996168	circRNA_010303	11:5347897|5360480	circRNA_006929	12:27366725|27397310
circRNA_003791	17:53732886|53733365	circRNA_013681	13:58781354|58789759	circRNA_004800	7:73060730|73073314	circRNA_014955	1:2783966|2814551
circRNA_004988	8:104002975|104003454	circRNA_000894	15:22759841|22768247	circRNA_006434	8:140242798|140255386	circRNA_020658	13:210819581|210850177
circRNA_013489	9:116762218|116762697	circRNA_004821	7:91969534|91977940	circRNA_004607	6:89203607|89216201	circRNA_005409	1:21191798|21222409
circRNA_006551	MT:2986|3466	circRNA_007231	15:53791850|53800256	circRNA_002864	10:77696541|77709139	circRNA_020807	17:25115023|25145634
circRNA_001125	17:55247404|55247885	circRNA_000735	14:10662186|10670593	circRNA_001139	18:2767700|2780302	circRNA_016612	X:140031246|140061863
circRNA_003433	14:118004686|118005168	circRNA_011668	3:26988009|26996417	circRNA_018943	2:132227980|132240582	circRNA_008919	14:68165377|68195995
circRNA_004028	2:142510447|142510929	circRNA_005569	10:66171809|66180218	circRNA_013348	7:102964571|102977175	circRNA_011660	3:16566447|16597096
circRNA_013449	9:40553755|40554238	circRNA_011421	15:86614285|86622694	circRNA_016739	11:48208224|48220828	circRNA_011679	3:45685601|45716254
circRNA_007556	3:59520220|59520704	circRNA_020253	7:33603201|33611612	circRNA_015919	2:125498449|125511054	circRNA_013594	X:126492530|126523185
circRNA_002472	1:18633147|18633632	circRNA_005045	9:7134810|7143222	circRNA_006395	8:34341118|34353725	circRNA_005831	15:34820828|34851487
circRNA_012069	9:69159033|69159518	circRNA_016623	1:22126330|22134742	circRNA_007297	15:157411434|157424041	circRNA_020736	15:71690220|71720879
circRNA_021094	6:23732487|23732972	circRNA_021253	9:29811553|29819965	circRNA_003641	15:131280019|131292628	circRNA_010005	9:114841276|114871940
circRNA_003949	2:85973785|85974271	circRNA_014275	15:34947373|34955787	circRNA_000170	1:200269189|200281803	circRNA_001344	2:132404785|132435460
circRNA_007141	14:65584884|65585370	circRNA_019838	8:141400930|141409344	circRNA_002361	GL895293.1:36054|48671	circRNA_008044	8:109011842|109042519
circRNA_008458	1:243435941|243436427	circRNA_009233	2:35599194|35607609	circRNA_001903	6:132138607|132151229	circRNA_007261	15:87509872|87540556
circRNA_013994	10:48609499|48609985	circRNA_012422	11:11327362|11335777	circRNA_000534	13:18735166|18747789	circRNA_006575	X:85891093|85921778
circRNA_017873	6:869802|870288	circRNA_016546	GL895853.2:73107|81522	circRNA_007785	6:9731929|9744554	circRNA_010462	15:3761230|3791918
circRNA_020937	3:24310937|24311423	circRNA_017049	2:142510447|142518862	circRNA_013674	13:33736722|33749350	circRNA_012218	1:74429977|74460665
circRNA_018270	4:102864459|102864946	circRNA_012721	15:61777761|61786177	circRNA_006058	3:10479905|10492534	circRNA_016361	8:45754467|45785156
circRNA_019825	8:86299463|86299950	circRNA_014133	1:208307045|208315461	circRNA_020880	2:64746824|64759456	circRNA_007356	17:43030480|43061187
circRNA_004263	4:42849823|42850311	circRNA_004930	8:57901394|57909812	circRNA_002281	9:115691139|115703772	circRNA_006792	1:297336189|297366899
circRNA_004651	6:120012902|120013390	circRNA_012453	12:289311|297730	circRNA_000191	1:208103946|208116580	circRNA_009695	6:132138607|132169325
circRNA_019754	6:41561594|41562084	circRNA_007302	16:23719895|23728315	circRNA_013628	14:71903291|71915927	circRNA_004939	8:64483430|64514152
circRNA_004531	6:14475670|14476162	circRNA_008589	11:16104484|16112904	circRNA_003681	16:29438836|29451474	circRNA_002609	1:145408743|145439488
circRNA_018717	14:35157985|35158477	circRNA_004086	3:45768689|45777111	circRNA_006534	GL895293.1:36054|48692	circRNA_007389	18:20655219|20685978
circRNA_020879	2:61950218|61950710	circRNA_020559	12:37420987|37429409	circRNA_008698	12:63473957|63486597	circRNA_002582	1:132965230|132995990
circRNA_007909	7:48721997|48722490	circRNA_008778	13:83171324|83179748	circRNA_015378	11:20082238|20094879	circRNA_013330	7:65664182|65694943
circRNA_009417	3:125926943|125927436	circRNA_011376	14:132237197|132245621	circRNA_017370	GL894396.1:31788|44429	circRNA_003090	13:29719703|29750471
circRNA_007946	7:97833510|97834004	circRNA_013105	4:73759207|73767631	circRNA_018621	13:16461158|16473811	circRNA_004470	5:76327567|76358338
circRNA_011019	1:156020861|156021356	circRNA_017032	2:89583247|89591671	circRNA_003034	12:45484923|45497578	circRNA_008196	GL893725.2:2267|33040
circRNA_015410	11:87013576|87014071	circRNA_020927	2:148224335|148232759	circRNA_013619	1:208292449|208305105	circRNA_000352	10:58881715|58912493
circRNA_008508	10:11927976|11928472	circRNA_000301	10:27783758|27792184	circRNA_013717	5:213884|226541	circRNA_017684	15:106832344|106863127
circRNA_018874	18:35925585|35926081	circRNA_001400	3:29760501|29768928	circRNA_009036	15:86948961|86961619	circRNA_000835	14:98695887|98726678
circRNA_014029	7:106449290|106449787	circRNA_013426	8:143266951|143275381	circRNA_003028	12:45341102|45353762	circRNA_008177	9:138823270|138854073
circRNA_015861	2:5812713|5813210	circRNA_013142	4:142573710|142582143	circRNA_016458	9:44753740|44766400	circRNA_003826	18:18610834|18641648
circRNA_004593	6:85048146|85048644	circRNA_005043	9:7128595|7137030	circRNA_003678	16:27840671|27853332	circRNA_008522	10:33047631|33078465
circRNA_008947	14:115638815|115639313	circRNA_014757	X:121820813|121829249	circRNA_007990	8:31012141|31024807	circRNA_006642	1:74429977|74460814
circRNA_011111	10:47265021|47265519	circRNA_000169	1:200269189|200277626	circRNA_007722	5:13967635|13980303	circRNA_005697	13:90919603|90950445

circRNA_011388	14:146810850|146811348	circRNA_018949	2:142535263|142543701	circRNA_003531	15:61770154|61782824	circRNA_011047	1:227165198|227196042
circRNA_016397	8:110791016|110791514	circRNA_019747	6:13110937|13119375	circRNA_008090	9:19330947|19343618	circRNA_012872	18:25777975|25808837
circRNA_017644	14:95579373|95579872	circRNA_000036	1:37134829|37143270	circRNA_002661	1:202606369|202619041	circRNA_010478	15:73542910|73573786
circRNA_015548	13:131581844|131582344	circRNA_010901	9:132867376|132875818	circRNA_012059	9:40646005|40658685	circRNA_017113	4:56554006|56584883
circRNA_019330	JH118940.1:109810|110310	circRNA_006041	2:136420659|136429104	circRNA_016540	GL895238.2:49303|61985	circRNA_005874	15:128043602|128074514
circRNA_003646	15:142752193|142752694	circRNA_014073	16:51755379|51763824	circRNA_020926	2:146232192|146244874	circRNA_012189	1:23208792|23239705
circRNA_015491	13:34947591|34948092	circRNA_018127	13:81538597|81547042	circRNA_012428	11:16154643|16167326	circRNA_000848	14:117069303|117100223
circRNA_018979	3:67300428|67300929	circRNA_008682	12:48582940|48591386	circRNA_007855	6:108423850|108436534	circRNA_009827	8:29279800|29310723
circRNA_020618	13:122267966|122268467	circRNA_012308	1:244821954|244830400	circRNA_012658	14:68165377|68178062	circRNA_002035	7:116973936|117004866
circRNA_005884	16:19146666|19147169	circRNA_014965	1:225104208|225112654	circRNA_007139	14:63330103|63342789	circRNA_003093	13:33153278|33184216
circRNA_008654	12:25026657|25027161	circRNA_006685	1:134774498|134782945	circRNA_009799	7:102337721|102350409	circRNA_014445	5:37132179|37163131
circRNA_013498	9:132597635|132598141	circRNA_007954	7:103941332|103949780	circRNA_021051	5:33932196|33944886	circRNA_007663	4:72772834|72803788
circRNA_014306	15:142021254|142021761	circRNA_006088	3:90673158|90681607	circRNA_003036	12:48106851|48119542	circRNA_006031	2:119198668|119229632
circRNA_012174	X:142050065|142050573	circRNA_013632	17:39442884|39451333	circRNA_008982	15:18557378|18570071	circRNA_010424	14:30872166|30903132
circRNA_015050	7:124987073|124987581	circRNA_003643	15:133380261|133388711	circRNA_000492	12:44463879|44476577	circRNA_005082	9:24125972|24156940
circRNA_018755	14:115638804|115639313	circRNA_015667	14:133487770|133496220	circRNA_009119	16:71385817|71398515	circRNA_014852	6:119982422|120013390
circRNA_006264	6:55937345|55937855	circRNA_010368	13:43409382|43417833	circRNA_020297	8:135793313|135806012	circRNA_002683	1:211714940|211745915
circRNA_019666	3:27911181|27911691	circRNA_008545	10:51578404|51586857	circRNA_013676	13:43393874|43406575	circRNA_015873	2:30450151|30481157
circRNA_020626	13:131630312|131630823	circRNA_011824	5:65026933|65035386	circRNA_020999	3:135328996|135341697	circRNA_004481	5:83018001|83049008
circRNA_000708	13:206987445|206987957	circRNA_003798	17:65846664|65855120	circRNA_005757	14:3194644|3207347	circRNA_011821	5:56933127|56964141
circRNA_005031	8:144751418|144751930	circRNA_017770	2:113169612|113178069	circRNA_001974	7:57934614|57947318	circRNA_013955	8:124913148|124944168
circRNA_007405	18:44530678|44531192	circRNA_019181	7:94204724|94213181	circRNA_016737	11:22180829|22193534	circRNA_004427	5:37132109|37163131
circRNA_003524	15:55354683|55355198	circRNA_014222	13:148454748|148463208	circRNA_009762	7:65184011|65196717	circRNA_015274	1:230890866|230921889
circRNA_009626	6:39320647|39321162	circRNA_009611	6:12648433|12656894	circRNA_020911	2:122626814|122639520	circRNA_020454	1:215878079|215909111
circRNA_000586	13:49818677|49819194	circRNA_009366	3:61511667|61520129	circRNA_010724	5:93498712|93511420	circRNA_002833	10:51236326|51267368
circRNA_018720	14:41894075|41894592	circRNA_017207	6:145705905|145714368	circRNA_010931	X:19220867|19233575	circRNA_018456	1:145455671|145486713
circRNA_020092	17:43114806|43115323	circRNA_016756	12:20113464|20121928	circRNA_006998	13:57974186|57986896	circRNA_008459	1:244316290|244347335
circRNA_007520	2:147053805|147054323	circRNA_018571	11:14848030|14856494	circRNA_018916	2:79837584|79850295	circRNA_012774	15:128047839|128078889
circRNA_008405	1:178909297|178909815	circRNA_021308	GL893655.1:37492|45957	circRNA_016572	X:14166333|14179050	circRNA_001997	7:73042261|73073314
circRNA_021275	9:79962890|79963408	circRNA_004700	6:148116282|148124749	circRNA_004653	6:125658483|125671201	circRNA_005192	9:138853942|138885004
circRNA_007255	15:85045931|85046450	circRNA_001859	6:99075904|99084372	circRNA_020806	17:14009032|14021750	circRNA_019098	6:37870420|37901490
circRNA_001618	4:102984325|102984845	circRNA_013906	3:122011566|122020038	circRNA_018726	14:51495045|51507764	circRNA_009303	2:139087755|139118827
circRNA_007345	17:15902244|15902764	circRNA_021313	GL894163.1:11497|19969	circRNA_005736	13:155848724|155861447	circRNA_017551	11:56099855|56130928
circRNA_011856	6:871815|872335	circRNA_018404	1:16080529|16089003	circRNA_013930	5:92098435|92111160	circRNA_017653	14:136857332|136888438
circRNA_002825	10:47242802|47243323	circRNA_002324	GL892547.1:5870|14345	circRNA_004099	3:63158910|63171638	circRNA_019973	11:72290089|72321197
circRNA_014053	6:79983161|79983682	circRNA_003065	13:14159901|14168377	circRNA_006339	7:62835978|62848707	circRNA_009871	8:93287563|93318694
circRNA_008568	10:71361138|71361661	circRNA_014994	14:59996069|60004545	circRNA_010981	1:101733942|101746676	circRNA_002074	8:34341118|34372263
circRNA_019049	4:121586260|121586783	circRNA_007178	14:120779066|120787546	circRNA_016907	15:97974673|97987408	circRNA_011995	8:32861923|32893069
circRNA_015453	12:44476053|44476577	circRNA_005129	9:72670797|72679278	circRNA_014921	15:55357496|55370232	circRNA_005751	13:212955534|212986687
circRNA_020037	14:51674214|51674738	circRNA_008323	1:59744264|59752746	circRNA_005593	11:25511459|25524196	circRNA_012138	GL896504.1:24299|55460
circRNA_020600	13:80161907|80162433	circRNA_005276	GL896372.1:1040|9523	circRNA_007413	18:59628324|59641061	circRNA_013752	3:121362925|121394097
circRNA_007023	13:90954665|90955192	circRNA_007058	13:138069734|138078218	circRNA_003526	15:56525514|56538254	circRNA_000813	14:81008366|81039544
circRNA_010814	7:129912574|129913102	circRNA_016525	GL894350.2:21972|30458	circRNA_007110	14:33910035|33922776	circRNA_011829	5:80902859|80934041
circRNA_016843	14:36783522|36784050	circRNA_020271	7:102997168|103005656	circRNA_021223	8:111993346|112006091	circRNA_009541	5:49898645|49929837
circRNA_003300	14:13445402|13445931	circRNA_004484	5:86283657|86292147	circRNA_000440	12:151796|164543	circRNA_013634	4:39819040|39850235
circRNA_012227	1:97331478|97332007	circRNA_009603	6:204871|213363	circRNA_001653	4:129725177|129737926	circRNA_004827	7:97312228|97343426
circRNA_013216	6:3096151|3096680	circRNA_003132	13:67233177|67241670	circRNA_011010	1:143267765|143280515	circRNA_007191	14:136612215|136643431
circRNA_000973	15:97957279|97957809	circRNA_001919	6:147017881|147026376	circRNA_019547	14:116609237|116621990	circRNA_019143	7:129268|160485
circRNA_013188	5:82763395|82763925	circRNA_000979	15:104515243|104523739	circRNA_009885	8:116836002|116848756	circRNA_008398	1:169304335|169335560
circRNA_009904	8:141449845|141450376	circRNA_011054	1:256337526|256346024	circRNA_015714	15:94293320|94306076	circRNA_008228	JH118611.1:2203|33429
circRNA_017217	7:18159040|18159572	circRNA_005828	15:22575140|22583640	circRNA_013599	8:47773859|47786617	circRNA_002154	8:139497435|139528662
circRNA_014356	2:43344322|43344855	circRNA_021107	6:79324154|79332659	circRNA_020835	18:27396026|27408790	circRNA_001481	3:104168480|104199715

<table>
<tr><td>circRNA_019818</td><td>8:34729642|34730175</td><td>circRNA_001906</td><td>6:137394338|137402844</td><td>circRNA_007317</td><td>16:38185893|38198658</td><td>circRNA_014287</td><td>15:81180411|81211649</td></tr>
<tr><td>circRNA_000673</td><td>13:144779663|144780197</td><td>circRNA_003460</td><td>14:136289720|136298231</td><td>circRNA_017717</td><td>17:5203741|5216506</td><td>circRNA_020542</td><td>12:13034185|13065423</td></tr>
<tr><td>circRNA_011568</td><td>2:17189969|17190503</td><td>circRNA_007268</td><td>15:105475683|105484194</td><td>circRNA_010459</td><td>14:142837282|142850048</td><td>circRNA_011526</td><td>17:50274078|50305326</td></tr>
<tr><td>circRNA_004555</td><td>6:50083452|50083987</td><td>circRNA_005413</td><td>1:25396409|25404924</td><td>circRNA_011810</td><td>5:35782269|35795037</td><td>circRNA_005381</td><td>X:121910171|121941420</td></tr>
<tr><td>circRNA_009738</td><td>7:35115791|35116326</td><td>circRNA_012016</td><td>8:103930161|103938677</td><td>circRNA_005846</td><td>15:73580843|73593612</td><td>circRNA_015377</td><td>11:20075966|20107217</td></tr>
<tr><td>circRNA_018428</td><td>1:99237901|99238436</td><td>circRNA_001858</td><td>6:98796781|98805299</td><td>circRNA_001872</td><td>6:101573996|101586767</td><td>circRNA_016244</td><td>6:99516427|99547687</td></tr>
<tr><td>circRNA_018912</td><td>2:65365209|65365744</td><td>circRNA_008519</td><td>10:30396527|30405046</td><td>circRNA_008821</td><td>13:141952509|141965282</td><td>circRNA_020616</td><td>13:119205106|119236371</td></tr>
<tr><td>circRNA_006816</td><td>10:20398666|20399202</td><td>circRNA_001843</td><td>6:87841092|87849612</td><td>circRNA_019670</td><td>3:45549204|45561978</td><td>circRNA_002214</td><td>9:38389524|38420790</td></tr>
<tr><td>circRNA_009781</td><td>7:80416946|80417483</td><td>circRNA_000920</td><td>15:61425963|61434485</td><td>circRNA_005875</td><td>15:133558194|133570976</td><td>circRNA_012627</td><td>14:31262408|31293688</td></tr>
<tr><td>circRNA_012900</td><td>2:5812680|5813217</td><td>circRNA_003243</td><td>13:167096198|167104720</td><td>circRNA_009536</td><td>5:38567660|38580442</td><td>circRNA_003636</td><td>15:124662048|124693334</td></tr>
<tr><td>circRNA_000133</td><td>1:141993842|141994380</td><td>circRNA_007216</td><td>15:19243124|19251646</td><td>circRNA_002096</td><td>8:74346358|74359142</td><td>circRNA_012099</td><td>GL892397.1:109233|140520</td></tr>
<tr><td>circRNA_009879</td><td>8:104103005|104103543</td><td>circRNA_007160</td><td>14:95571347|95579872</td><td>circRNA_006017</td><td>2:91592359|91605143</td><td>circRNA_005820</td><td>14:145833236|145864533</td></tr>
<tr><td>circRNA_010786</td><td>7:21070538|21071076</td><td>circRNA_016440</td><td>9:25899676|25908203</td><td>circRNA_014895</td><td>JH118921.1:16802|29587</td><td>circRNA_011994</td><td>8:32728074|32759376</td></tr>
<tr><td>circRNA_004189</td><td>3:119833596|119834135</td><td>circRNA_019424</td><td>10:33049254|33057782</td><td>circRNA_009069</td><td>15:152385966|152398753</td><td>circRNA_003639</td><td>15:131248910|131280215</td></tr>
<tr><td>circRNA_016580</td><td>X:21815559|21816098</td><td>circRNA_001403</td><td>3:33369926|33378455</td><td>circRNA_006295</td><td>6:118035375|118048164</td><td>circRNA_011950</td><td>7:62090955|62122274</td></tr>
<tr><td>circRNA_007777</td><td>5:92125555|92126095</td><td>circRNA_018542</td><td>10:35254557|35263088</td><td>circRNA_019077</td><td>5:72095260|72108057</td><td>circRNA_004623</td><td>6:99212305|99243627</td></tr>
<tr><td>circRNA_016864</td><td>14:86998888|86999428</td><td>circRNA_018179</td><td>15:85018708|85027240</td><td>circRNA_008369</td><td>1:132344010|132356808</td><td>circRNA_020661</td><td>13:212963033|212994363</td></tr>
<tr><td>circRNA_006553</td><td>MT:3091|3632</td><td>circRNA_011831</td><td>5:82625769|82634302</td><td>circRNA_009314</td><td>2:142624803|142637604</td><td>circRNA_020240</td><td>6:139905274|139936605</td></tr>
<tr><td>circRNA_019363</td><td>1:48059061|48059602</td><td>circRNA_012338</td><td>10:11687662|11696196</td><td>circRNA_019269</td><td>9:52673623|52686429</td><td>circRNA_015243</td><td>1:204797064|204828419</td></tr>
<tr><td>circRNA_011575</td><td>2:30150022|30150564</td><td>circRNA_007696</td><td>4:129535947|129544482</td><td>circRNA_013014</td><td>3:61514538|61527346</td><td>circRNA_009841</td><td>8:42394633|42426011</td></tr>
<tr><td>circRNA_014025</td><td>7:35546228|35546770</td><td>circRNA_000144</td><td>1:145394702|145403239</td><td>circRNA_000359</td><td>10:71294817|71307626</td><td>circRNA_016676</td><td>1:205495469|205526853</td></tr>
<tr><td>circRNA_003917</td><td>2:54394540|54395084</td><td>circRNA_012412</td><td>11:3765581|3774119</td><td>circRNA_000358</td><td>10:69978850|69991661</td><td>circRNA_019408</td><td>1:266578997|266610386</td></tr>
<tr><td>circRNA_009531</td><td>5:35794493|35795037</td><td>circRNA_005681</td><td>13:72895321|72903860</td><td>circRNA_005203</td><td>9:143478477|143491288</td><td>circRNA_005644</td><td>13:15518257|15549655</td></tr>
<tr><td>circRNA_013015</td><td>3:62271197|62271741</td><td>circRNA_015590</td><td>14:6997771|7006310</td><td>circRNA_015374</td><td>11:16783166|16795977</td><td>circRNA_000658</td><td>13:138876327|138907728</td></tr>
<tr><td>circRNA_014247</td><td>14:51304643|51305187</td><td>circRNA_017575</td><td>12:45671206|45679747</td><td>circRNA_002823</td><td>10:47093752|47106567</td><td>circRNA_007881</td><td>6:147617242|147648651</td></tr>
<tr><td>circRNA_008612</td><td>11:54017786|54018331</td><td>circRNA_015292</td><td>1:257015250|257023798</td><td>circRNA_017066</td><td>3:43036166|43048981</td><td>circRNA_007310</td><td>16:36617706|36649117</td></tr>
<tr><td>circRNA_013138</td><td>4:137125707|137126252</td><td>circRNA_008702</td><td>13:3105140|3113691</td><td>circRNA_004139</td><td>3:90566719|90579536</td><td>circRNA_003954</td><td>2:86260574|86291987</td></tr>
<tr><td>circRNA_009050</td><td>15:118036732|118037278</td><td>circRNA_012847</td><td>17:57066927|57075478</td><td>circRNA_008887</td><td>14:32141473|32154291</td><td>circRNA_013989</td><td>1:269689085|269720498</td></tr>
<tr><td>circRNA_013577</td><td>X:46394977|46395523</td><td>circRNA_014983</td><td>13:66355056|66363609</td><td>circRNA_009942</td><td>9:40148558|40161378</td><td>circRNA_001180</td><td>18:25520260|25551680</td></tr>
<tr><td>circRNA_002947</td><td>12:1154730|1155277</td><td>circRNA_012377</td><td>10:53346989|53355543</td><td>circRNA_020204</td><td>5:91211194|91224020</td><td>circRNA_008601</td><td>11:24805989|24837421</td></tr>
<tr><td>circRNA_013583</td><td>X:67160527|67161074</td><td>circRNA_017751</td><td>2:47993416|48001971</td><td>circRNA_006780</td><td>1:280497623|280510450</td><td>circRNA_006635</td><td>1:60893530|60924967</td></tr>
<tr><td>circRNA_001803</td><td>6:50127662|50128210</td><td>circRNA_002412</td><td>X:17758636|17767192</td><td>circRNA_003083</td><td>13:28188392|28201220</td><td>circRNA_017666</td><td>15:66034545|66066010</td></tr>
<tr><td>circRNA_012329</td><td>1:302240165|302240713</td><td>circRNA_017600</td><td>13:90509630|90518188</td><td>circRNA_021351</td><td>X:7309036|7321870</td><td>circRNA_018165</td><td>14:114537837|114569311</td></tr>
<tr><td>circRNA_004145</td><td>3:98116845|98117395</td><td>circRNA_017991</td><td>9:52694374|52702938</td><td>circRNA_010363</td><td>13:37486493|37499329</td><td>circRNA_009187</td><td>18:26314382|26345863</td></tr>
<tr><td>circRNA_010050</td><td>GL893329.2:13085|13635</td><td>circRNA_001184</td><td>18:42650214|42658779</td><td>circRNA_003781</td><td>17:45663844|45676687</td><td>circRNA_017891</td><td>6:79877008|79908489</td></tr>
<tr><td>circRNA_002421</td><td>X:47899533|47900084</td><td>circRNA_012878</td><td>18:41980502|41989067</td><td>circRNA_006486</td><td>9:79206662|79219505</td><td>circRNA_018068</td><td>1:16839578|16871069</td></tr>
<tr><td>circRNA_007336</td><td>17:5332994|5333545</td><td>circRNA_004253</td><td>4:37761441|37770008</td><td>circRNA_004399</td><td>5:3468414|3481258</td><td>circRNA_006452</td><td>9:13703971|13735468</td></tr>
<tr><td>circRNA_013884</td><td>2:72357058|72357609</td><td>circRNA_008199</td><td>GL893884.1:31770|40337</td><td>circRNA_019686</td><td>3:114074966|114087812</td><td>circRNA_003780</td><td>17:45645182|45676687</td></tr>
<tr><td>circRNA_000372</td><td>11:266572|267124</td><td>circRNA_013384</td><td>8:57803396|57811963</td><td>circRNA_010374</td><td>13:73329729|73342587</td><td>circRNA_006945</td><td>12:45334013|45365521</td></tr>
<tr><td>circRNA_010102</td><td>MT:2926|3478</td><td>circRNA_021292</td><td>9:134235965|134244532</td><td>circRNA_010215</td><td>1:169133734|169146593</td><td>circRNA_010986</td><td>1:124864310|124895832</td></tr>
<tr><td>circRNA_016638</td><td>1:103475507|103476060</td><td>circRNA_015309</td><td>1:293355657|293364225</td><td>circRNA_013781</td><td>1:202644306|202657170</td><td>circRNA_017526</td><td>10:50892576|50924109</td></tr>
<tr><td>circRNA_017170</td><td>6:50127657|50128210</td><td>circRNA_005361</td><td>X:105400134|105408704</td><td>circRNA_005177</td><td>9:131740321|131753192</td><td>circRNA_003822</td><td>18:15020215|15051751</td></tr>
<tr><td>circRNA_014643</td><td>13:108395217|108395772</td><td>circRNA_010120</td><td>X:51844159|51852729</td><td>circRNA_017302</td><td>8:143262507|143275381</td><td>circRNA_020656</td><td>13:202622610|202654149</td></tr>
<tr><td>circRNA_019288</td><td>9:122734906|122735461</td><td>circRNA_015182</td><td>1:86333461|86342031</td><td>circRNA_010180</td><td>1:60892775|60905657</td><td>circRNA_003424</td><td>14:115712680|115744220</td></tr>
<tr><td>circRNA_021364</td><td>X:47899529|47900084</td><td>circRNA_000166</td><td>1:195169049|195177620</td><td>circRNA_003897</td><td>2:36230781|36243672</td><td>circRNA_018901</td><td>2:43632613|43664163</td></tr>
<tr><td>circRNA_010716</td><td>5:84032680|84033236</td><td>circRNA_020259</td><td>7:57960999|57969573</td><td>circRNA_011375</td><td>14:131878915|131891808</td><td>circRNA_008846</td><td>13:190906188|190937745</td></tr>
<tr><td>circRNA_013354</td><td>7:117841710|117842266</td><td>circRNA_012108</td><td>GL893259.1:18126|26701</td><td>circRNA_015314</td><td>1:297539041|297551935</td><td>circRNA_008864</td><td>13:216655489|216687050</td></tr>
<tr><td>circRNA_013975</td><td>JH118612.1:122418|122975</td><td>circRNA_015539</td><td>13:118617384|118625960</td><td>circRNA_003848</td><td>18:36467738|36480633</td><td>circRNA_020894</td><td>2:87139525|87171087</td></tr>
<tr><td>circRNA_001537</td><td>3:135341139|135341697</td><td>circRNA_001337</td><td>2:122033113|122041690</td><td>circRNA_014682</td><td>2:90660850|90673745</td><td>circRNA_020843</td><td>18:43963437|43995012</td></tr>
<tr><td>circRNA_003205</td><td>13:132656250|132656809</td><td>circRNA_003268</td><td>13:210784081|210792658</td><td>circRNA_003960</td><td>2:87164147|87177048</td><td>circRNA_008804</td><td>13:108339930|108371507</td></tr>
<tr><td>circRNA_018032</td><td>GL896097.1:760|1319</td><td>circRNA_012223</td><td>1:85597181|85605758</td><td>circRNA_011413</td><td>15:84975710|84988611</td><td>circRNA_011732</td><td>3:120255517|120287104</td></tr>
<tr><td>circRNA_018361</td><td>9:359578|360137</td><td>circRNA_013556</td><td>JH118963.1:56856|65433</td><td>circRNA_020137</td><td>3:45419321|45432223</td><td>circRNA_009601</td><td>5:108374735|108406326</td></tr>
</table>

<table>
<tr><td>circRNA_005279</td><td>GL896440.1:9766|10326</td><td>circRNA_005615</td><td>12:18731949|18740531</td><td>circRNA_004504</td><td>5:99086346|99099249</td><td>circRNA_000954</td><td>15:85315348|85346940</td></tr>
<tr><td>circRNA_017337</td><td>9:71393765|71394325</td><td>circRNA_005933</td><td>17:57170779|57179361</td><td>circRNA_005332</td><td>X:46141564|46154467</td><td>circRNA_009610</td><td>6:12077068|12108674</td></tr>
<tr><td>circRNA_019691</td><td>4:7909969|7910530</td><td>circRNA_019768</td><td>6:112903807|112912389</td><td>circRNA_002793</td><td>10:29339600|29352504</td><td>circRNA_005567</td><td>10:60092228|60123846</td></tr>
<tr><td>circRNA_005303</td><td>MT:2300|2862</td><td>circRNA_008897</td><td>14:43179929|43188512</td><td>circRNA_016333</td><td>7:134411388|134424293</td><td>circRNA_004053</td><td>3:11468929|11500560</td></tr>
<tr><td>circRNA_013843</td><td>14:132373518|132374080</td><td>circRNA_009818</td><td>8:1248425|1257008</td><td>circRNA_009148</td><td>17:43061013|43073920</td><td>circRNA_007900</td><td>7:21530071|21561703</td></tr>
<tr><td>circRNA_016575</td><td>X:15200594|15201156</td><td>circRNA_018403</td><td>1:14099143|14107726</td><td>circRNA_018614</td><td>12:52763999|52776906</td><td>circRNA_014503</td><td>7:91932958|91964590</td></tr>
<tr><td>circRNA_018911</td><td>2:61914801|61915363</td><td>circRNA_009687</td><td>6:108531838|108540422</td><td>circRNA_014458</td><td>5:77630305|77643213</td><td>circRNA_014019</td><td>4:137104657|137136291</td></tr>
<tr><td>circRNA_004261</td><td>4:42011532|42012095</td><td>circRNA_017972</td><td>8:140624179|140632763</td><td>circRNA_006184</td><td>4:130817704|130830613</td><td>circRNA_005599</td><td>11:52232763|52264401</td></tr>
<tr><td>circRNA_005333</td><td>X:47397169|47397732</td><td>circRNA_015804</td><td>18:814124|822709</td><td>circRNA_008109</td><td>9:44292300|44305209</td><td>circRNA_005579</td><td>11:6772276|6803923</td></tr>
<tr><td>circRNA_012063</td><td>9:49325141|49325706</td><td>circRNA_019765</td><td>6:96570946|96579531</td><td>circRNA_000838</td><td>14:113064543|113077453</td><td>circRNA_010908</td><td>9:143556231|143587888</td></tr>
<tr><td>circRNA_015321</td><td>10:177153|177718</td><td>circRNA_012219</td><td>1:84723988|84732574</td><td>circRNA_002447</td><td>X:130750807|130763718</td><td>circRNA_008645</td><td>12:16053861|16085519</td></tr>
<tr><td>circRNA_017966</td><td>8:112710597|112711162</td><td>circRNA_002697</td><td>1:233012606|233021193</td><td>circRNA_019675</td><td>3:76037041|76049955</td><td>circRNA_008064</td><td>8:141433436|141465095</td></tr>
<tr><td>circRNA_017728</td><td>17:55866818|55867387</td><td>circRNA_012844</td><td>17:47003535|47012122</td><td>circRNA_003888</td><td>2:16918297|16931216</td><td>circRNA_000259</td><td>1:283810273|283841933</td></tr>
<tr><td>circRNA_001706</td><td>5:38350856|38351426</td><td>circRNA_004871</td><td>8:1048082|1056670</td><td>circRNA_005451</td><td>1:134770024|134782945</td><td>circRNA_016362</td><td>8:55114181|55145843</td></tr>
<tr><td>circRNA_005372</td><td>X:121785129|121785699</td><td>circRNA_016287</td><td>7:45327224|45335812</td><td>circRNA_007595</td><td>3:109761586|109774507</td><td>circRNA_002853</td><td>10:62859080|62890750</td></tr>
<tr><td>circRNA_016559</td><td>JH118496.1:2119|2689</td><td>circRNA_017726</td><td>17:52888413|52897001</td><td>circRNA_008326</td><td>1:64724999|64737924</td><td>circRNA_016074</td><td>4:67861414|67893087</td></tr>
<tr><td>circRNA_017870</td><td>5:103839614|103840184</td><td>circRNA_006689</td><td>1:141600567|141609157</td><td>circRNA_004747</td><td>7:33183955|33196881</td><td>circRNA_012126</td><td>GL895028.1:12988|44671</td></tr>
<tr><td>circRNA_002403</td><td>MT:3024|3595</td><td>circRNA_013455</td><td>9:44395930|44404520</td><td>circRNA_005141</td><td>9:83350139|83363067</td><td>circRNA_004310</td><td>4:90629026|90660717</td></tr>
<tr><td>circRNA_003186</td><td>13:103725786|103726359</td><td>circRNA_007878</td><td>6:147348510|147357101</td><td>circRNA_019245</td><td>9:11209215|11222145</td><td>circRNA_017037</td><td>2:106236891|106268585</td></tr>
<tr><td>circRNA_006757</td><td>1:241247055|241247628</td><td>circRNA_006190</td><td>5:8992765|9001360</td><td>circRNA_011165</td><td>12:21025298|21038230</td><td>circRNA_019306</td><td>GL893011.1:27745|59476</td></tr>
<tr><td>circRNA_001566</td><td>4:41982139|41982713</td><td>circRNA_020150</td><td>3:102681763|102690358</td><td>circRNA_013935</td><td>6:78655585|78668523</td><td>circRNA_010030</td><td>9:144062171|144093904</td></tr>
<tr><td>circRNA_002739</td><td>1:275565268|275565842</td><td>circRNA_009801</td><td>7:104147580|104156180</td><td>circRNA_009890</td><td>8:124242821|124255766</td><td>circRNA_006417</td><td>8:85974968|86006710</td></tr>
<tr><td>circRNA_016608</td><td>X:126963525|126964099</td><td>circRNA_010119</td><td>X:46519648|46528248</td><td>circRNA_013710</td><td>3:46250087|46263034</td><td>circRNA_007341</td><td>17:10389674|10421420</td></tr>
<tr><td>circRNA_020928</td><td>2:150637205|150637779</td><td>circRNA_004548</td><td>6:37733939|37742540</td><td>circRNA_013856</td><td>15:133579638|133592588</td><td>circRNA_016919</td><td>15:133539228|133570976</td></tr>
<tr><td>circRNA_005513</td><td>1:264890623|264891198</td><td>circRNA_017102</td><td>4:43558505|43567106</td><td>circRNA_014453</td><td>5:61454150|61467100</td><td>circRNA_001588</td><td>4:67861336|67893087</td></tr>
<tr><td>circRNA_019048</td><td>4:118778275|118778851</td><td>circRNA_020793</td><td>16:51805936|51814537</td><td>circRNA_017039</td><td>2:106423456|106436407</td><td>circRNA_007049</td><td>13:131128156|131159913</td></tr>
<tr><td>circRNA_009267</td><td>2:87129523|87130100</td><td>circRNA_015654</td><td>14:98508745|98517347</td><td>circRNA_003315</td><td>14:29836044|29848997</td><td>circRNA_006825</td><td>10:33579419|33611200</td></tr>
<tr><td>circRNA_018514</td><td>1:264153454|264154031</td><td>circRNA_007195</td><td>14:136879835|136888438</td><td>circRNA_002312</td><td>9:146585271|146598229</td><td>circRNA_019146</td><td>7:10291894|10323693</td></tr>
<tr><td>circRNA_008565</td><td>10:65608769|65609347</td><td>circRNA_018204</td><td>18:9779444|9788048</td><td>circRNA_013724</td><td>7:91900007|91912965</td><td>circRNA_006131</td><td>4:34609769|34641574</td></tr>
<tr><td>circRNA_000102</td><td>1:125219032|125219611</td><td>circRNA_013220</td><td>6:14241727|14250339</td><td>circRNA_018386</td><td>GL894396.1:11490|24448</td><td>circRNA_016183</td><td>5:105265060|105296870</td></tr>
<tr><td>circRNA_002424</td><td>X:52208084|52208663</td><td>circRNA_011412</td><td>15:84413658|84422274</td><td>circRNA_003148</td><td>13:81424354|81437313</td><td>circRNA_009410</td><td>3:121688604|121720420</td></tr>
<tr><td>circRNA_005744</td><td>13:199173390|199173969</td><td>circRNA_000778</td><td>14:53600684|53609301</td><td>circRNA_001689</td><td>5:16675258|16688220</td><td>circRNA_018398</td><td>X:108947693|108979510</td></tr>
<tr><td>circRNA_008899</td><td>14:44562865|44563444</td><td>circRNA_003847</td><td>18:36185037|36193660</td><td>circRNA_012983</td><td>2:153879053|153892018</td><td>circRNA_016947</td><td>16:74740187|74772010</td></tr>
<tr><td>circRNA_016896</td><td>15:61704909|61705488</td><td>circRNA_006579</td><td>X:105384438|105393061</td><td>circRNA_008010</td><td>8:57803396|57816363</td><td>circRNA_018473</td><td>1:181368995|181400829</td></tr>
<tr><td>circRNA_021206</td><td>8:73510598|73511177</td><td>circRNA_005816</td><td>14:140551654|140560278</td><td>circRNA_013113</td><td>4:88783359|88796326</td><td>circRNA_017618</td><td>13:210787792|210819635</td></tr>
<tr><td>circRNA_005934</td><td>17:57269639|57270219</td><td>circRNA_007935</td><td>7:91917856|91926482</td><td>circRNA_002948</td><td>12:1676102|1689070</td><td>circRNA_005392</td><td>X:138428178|138460022</td></tr>
<tr><td>circRNA_006797</td><td>1:304822634|304823214</td><td>circRNA_020249</td><td>7:20540093|20548720</td><td>circRNA_000803</td><td>14:77573358|77586330</td><td>circRNA_003820</td><td>18:15002914|15034759</td></tr>
<tr><td>circRNA_013495</td><td>9:125506887|125507467</td><td>circRNA_011651</td><td>2:154284494|154293122</td><td>circRNA_006397</td><td>8:40321067|40334043</td><td>circRNA_019095</td><td>6:32413181|32445038</td></tr>
<tr><td>circRNA_013530</td><td>GL894597.1:91960|92540</td><td>circRNA_011513</td><td>17:30276183|30284814</td><td>circRNA_013228</td><td>6:32884132|32897111</td><td>circRNA_014984</td><td>13:75395663|75427529</td></tr>
<tr><td>circRNA_016732</td><td>11:8505176|8505757</td><td>circRNA_013518</td><td>GL893329.2:11397|20029</td><td>circRNA_006024</td><td>2:105846755|105859735</td><td>circRNA_005708</td><td>13:119154837|119186715</td></tr>
<tr><td>circRNA_016877</td><td>14:125193307|125193888</td><td>circRNA_012495</td><td>12:44467943|44476577</td><td>circRNA_015427</td><td>12:15262828|15275808</td><td>circRNA_002144</td><td>8:130101503|130133384</td></tr>
<tr><td>circRNA_003922</td><td>2:69812430|69813013</td><td>circRNA_010890</td><td>9:79212011|79220647</td><td>circRNA_006240</td><td>5:92492203|92505185</td><td>circRNA_000097</td><td>1:124982100|125013992</td></tr>
<tr><td>circRNA_008210</td><td>GL894597.1:91957|92540</td><td>circRNA_000862</td><td>14:136879801|136888438</td><td>circRNA_016630</td><td>1:59739762|59752746</td><td>circRNA_019072</td><td>5:63272510|63304404</td></tr>
<tr><td>circRNA_006563</td><td>X:47621822|47622406</td><td>circRNA_002526</td><td>1:83314425|83323066</td><td>circRNA_001577</td><td>4:51356571|51369556</td><td>circRNA_006450</td><td>9:13417189|13449103</td></tr>
<tr><td>circRNA_012346</td><td>10:17413837|17414423</td><td>circRNA_020250</td><td>7:24138185|24146826</td><td>circRNA_007288</td><td>15:133619734|133632719</td><td>circRNA_005188</td><td>9:137710304|137742223</td></tr>
<tr><td>circRNA_014065</td><td>JH118743.1:14904|15490</td><td>circRNA_008731</td><td>13:35123699|35132343</td><td>circRNA_018538</td><td>10:27770855|27783841</td><td>circRNA_019951</td><td>1:297507827|297539752</td></tr>
<tr><td>circRNA_016228</td><td>6:85284146|85284732</td><td>circRNA_003404</td><td>14:92407605|92416252</td><td>circRNA_007298</td><td>16:6173606|6186594</td><td>circRNA_007403</td><td>18:44427910|44459840</td></tr>
<tr><td>circRNA_018001</td><td>9:101235349|101235936</td><td>circRNA_016122</td><td>5:3468414|3477062</td><td>circRNA_004526</td><td>6:12643904|12656894</td><td>circRNA_019383</td><td>1:142523717|142555648</td></tr>
<tr><td>circRNA_007816</td><td>6:50103805|50104393</td><td>circRNA_010693</td><td>5:17117262|17125911</td><td>circRNA_011855</td><td>5:109528884|109541874</td><td>circRNA_005221</td><td>GL892789.1:62629|94569</td></tr>
<tr><td>circRNA_015440</td><td>12:26293118|26293706</td><td>circRNA_017288</td><td>8:112052219|112060868</td><td>circRNA_019207</td><td>8:41183979|41196969</td><td>circRNA_010543</td><td>18:16661859|16693800</td></tr>
<tr><td>circRNA_021088</td><td>6:7598387|7598975</td><td>circRNA_009588</td><td>5:88170079|88178730</td><td>circRNA_018486</td><td>1:200287462|200300454</td><td>circRNA_013678</td><td>13:44645509|44677450</td></tr>
<tr><td>circRNA_018784</td><td>15:67051474|67052063</td><td>circRNA_004935</td><td>8:58320243|58328895</td><td>circRNA_009839</td><td>8:40860107|40873101</td><td>circRNA_012348</td><td>10:18187787|18219757</td></tr>
</table>

circRNA_014304	15:133579638|133580228	circRNA_017917	6:146614097|146622749	circRNA_020742	15:79996923|80009919	circRNA_019722	5:38558578|38590557
circRNA_021247	9:21378716|21379306	circRNA_008060	8:140670588|140679241	circRNA_005776	14:33699276|33712273	circRNA_021169	7:87883201|87915181
circRNA_007447	2:59783769|59784360	circRNA_015166	1:48325934|48334589	circRNA_008346	1:103248559|103261556	circRNA_001620	4:103198177|103230166
circRNA_019155	7:47118803|47119394	circRNA_003349	14:50004138|50012794	circRNA_003977	2:97468296|97481294	circRNA_000183	1:202954152|202986161
circRNA_000822	14:83832559|83833151	circRNA_018867	18:15785406|15794062	circRNA_003985	2:104498556|104511554	circRNA_004693	6:147045770|147077787
circRNA_021086	6:869802|870394	circRNA_013474	9:96037782|96046441	circRNA_011098	10:29576396|29589396	circRNA_009501	4:132846171|132878212
circRNA_001826	6:77623182|77623775	circRNA_017263	8:32725226|32733886	circRNA_011277	13:158524462|158537462	circRNA_012414	11:4038375|4070420
circRNA_018390	GL896197.1:10777|11370	circRNA_011295	13:210831648|210840309	circRNA_016338	8:4053490|4066496	circRNA_014370	2:118308150|118340199
circRNA_006179	4:121581861|121582456	circRNA_009777	7:73453613|73462279	circRNA_003711	16:47827438|47840449	circRNA_007046	13:130233837|130265889
circRNA_009843	8:43026094|43026689	circRNA_014008	15:59683347|59692013	circRNA_015574	13:202372077|202385088	circRNA_016825	13:166282060|166314115
circRNA_017472	1:179129495|179130090	circRNA_014401	3:92528897|92537565	circRNA_016303	7:73436271|73449286	circRNA_019600	17:55230543|55262601
circRNA_004797	7:69878128|69878724	circRNA_007559	3:61090978|61099647	circRNA_010006	9:114848880|114861897	circRNA_013908	4:21605708|21637785
circRNA_012845	17:53738526|53739122	circRNA_013817	13:80748224|80756893	circRNA_016917	15:122866809|122879826	circRNA_013052	3:121806817|121838900
circRNA_014413	4:14956919|14957516	circRNA_014479	6:88789118|88797787	circRNA_005062	9:12986704|12999722	circRNA_021068	5:87163988|87196099
circRNA_020959	3:56417580|56418177	circRNA_009570	5:73766947|73775618	circRNA_013919	4:121522270|121535288	circRNA_008180	9:144503744|144535857
circRNA_015059	9:51522099|51522697	circRNA_011769	4:51360884|51369556	circRNA_019264	9:44506050|44519068	circRNA_004913	8:41183979|41216093
circRNA_001813	6:65670011|65670610	circRNA_005296	JH118921.1:27248|35922	circRNA_006410	8:76070362|76083381	circRNA_003107	13:38256179|38288294
circRNA_002510	1:61006639|61007238	circRNA_004539	6:26453983|26462658	circRNA_012253	1:132577490|132590511	circRNA_002972	12:16069709|16101827
circRNA_010600	2:146082058|146082657	circRNA_002322	GL892430.1:44064|52745	circRNA_017626	14:33699276|33712298	circRNA_012353	10:30396527|30428646
circRNA_003434	14:120292442|120293042	circRNA_000094	1:121383033|121391716	circRNA_015560	13:150098374|150111397	circRNA_019022	4:40748966|40781089
circRNA_004155	3:102834961|102835561	circRNA_013655	1:23262198|23270881	circRNA_007928	7:73073215|73086240	circRNA_019443	11:11029229|11061383
circRNA_003945	2:83908876|83909477	circRNA_014778	12:44738008|44746693	circRNA_019812	8:664322|677347	circRNA_016224	6:76192486|76224646
circRNA_011441	15:120890047|120890648	circRNA_000234	1:264034002|264042688	circRNA_011137	11:18571298|18584327	circRNA_018065	1:4653396|4685558
circRNA_004520	6:11926255|11926858	circRNA_002369	GL896270.1:12583|21269	circRNA_005671	13:42379269|42392300	circRNA_000308	10:30396527|30428690
circRNA_013606	13:50969982|50970585	circRNA_013153	5:23502183|23510869	circRNA_003309	14:21472380|21485412	circRNA_016266	7:112298|144469
circRNA_018245	3:9331022|9331625	circRNA_000757	14:30974161|30982848	circRNA_009749	7:53593445|53606479	circRNA_019684	3:114064239|114096420
circRNA_021362	X:43151112|43151715	circRNA_008803	13:106755384|106764071	circRNA_018239	2:123072233|123085268	circRNA_019366	1:60892775|60924967
circRNA_004183	3:114091253|114091857	circRNA_004815	7:91904277|91912965	circRNA_003359	14:59605873|59618911	circRNA_020191	5:5399612|5431806
circRNA_014198	13:50969985|50970589	circRNA_001827	6:78655585|78664274	circRNA_012675	14:98721243|98734281	circRNA_015546	13:131205684|131237882
circRNA_000168	1:195179688|195180293	circRNA_008407	1:180714491|180723180	circRNA_017968	8:120772216|120785256	circRNA_020423	1:161713076|161745277
circRNA_019977	12:21074484|21075089	circRNA_009144	17:40408920|40417612	circRNA_011324	14:44612154|44625196	circRNA_007326	16:54825523|54857726
circRNA_003920	2:61899588|61900194	circRNA_016659	1:152340211|152348903	circRNA_008641	12:16036543|16049593	circRNA_005860	15:111918691|111950899
circRNA_014424	4:71351183|71351789	circRNA_004655	6:125857136|125865829	circRNA_010906	9:141723625|141736681	circRNA_016283	7:43147749|43179962
circRNA_020965	3:72753221|72753827	circRNA_006829	10:37127268|37135961	circRNA_015872	2:29518272|29531330	circRNA_019476	13:43393874|43426130
circRNA_008752	13:50969982|50970589	circRNA_019109	6:85422078|85430771	circRNA_004686	6:147017881|147030946	circRNA_010784	7:18468861|18501121
circRNA_016845	14:50012187|50012794	circRNA_014972	10:44187868|44196565	circRNA_004264	4:43055085|43068151	circRNA_005376	X:121793875|121826148
circRNA_004180	3:114048665|114049273	circRNA_001349	2:139012954|139021653	circRNA_019017	4:19615523|19628591	circRNA_012302	1:229214112|229246387
circRNA_006193	5:13399178|13399786	circRNA_007863	6:125857136|125865835	circRNA_020332	9:115681075|115694143	circRNA_019778	6:148040878|148073160
circRNA_013056	3:125872548|125873156	circRNA_013526	GL894027.2:45296|53995	circRNA_002781	10:18351232|18364302	circRNA_006727	1:188147577|188179860
circRNA_016944	16:50163576|50164184	circRNA_014775	12:21029531|21038230	circRNA_005494	1:210247860|210260930	circRNA_020938	3:28741072|28773356
circRNA_016786	13:38216935|38217544	circRNA_021229	8:120776554|120785256	circRNA_010765	6:132248038|132261114	circRNA_010245	1:244418080|244450365
circRNA_020501	10:19923823|19924434	circRNA_010064	GL894608.1:7692|16395	circRNA_015229	1:186643400|186656477	circRNA_005148	9:102535923|102568218
circRNA_003961	2:87303357|87303969	circRNA_019623	2:51439801|51448507	circRNA_001456	3:84809485|84822563	circRNA_012421	11:7790491|7822810
circRNA_016292	7:55698679|55699291	circRNA_020666	14:6684769|6693475	circRNA_013335	7:73449201|73462279	circRNA_019494	13:135510188|135542508
circRNA_004200	3:120585899|120586512	circRNA_005767	14:21488596|21497303	circRNA_014674	2:3009236|3022317	circRNA_000109	1:127997793|128030115
circRNA_019808	7:104185334|104185948	circRNA_013077	4:19832625|19841332	circRNA_001930	6:150861612|150874694	circRNA_019059	5:9709010|9741366
circRNA_001603	4:90623622|90624237	circRNA_001899	6:125857136|125865844	circRNA_002867	11:444919|458003	circRNA_018948	2:140877263|140909621
circRNA_005637	12:55974574|55975189	circRNA_000585	13:44026155|44034867	circRNA_020245	6:156274394|156287480	circRNA_000394	11:19712163|19744523
circRNA_002516	1:64750118|64750734	circRNA_019802	7:91917856|91926568	circRNA_015030	3:76474842|76487931	circRNA_019925	1:143945707|143978082
circRNA_020418	1:154279576|154280192	circRNA_015806	18:5545554|5554267	circRNA_003058	13:919792|932884	circRNA_020756	15:105475683|105508062
circRNA_020970	3:80752145|80752763	circRNA_013456	9:46185278|46193994	circRNA_009381	3:84438631|84451723	circRNA_001052	16:36742926|36775306
circRNA_016298	7:65167966|65168587	circRNA_005065	9:13656502|13665222	circRNA_015952	3:11505775|11518869	circRNA_017452	1:127604961|127637341

circRNA_000163	1:181484717|181485339	circRNA_002906	11:24782336|24791057	circRNA_016537	GL895006.2:40857|53958	circRNA_016762	12:37687990|37720372
circRNA_004514	6:871815|872437	circRNA_004218	3:135358108|135366829	circRNA_001249	2:52238948|52252052	circRNA_003161	13:86952848|86985258
circRNA_006619	1:25360229|25360852	circRNA_011959	7:91884279|91893001	circRNA_014599	X:73917548|73930652	circRNA_010879	9:51124702|51157112
circRNA_007969	7:124956434|124957057	circRNA_001641	4:118832318|118841042	circRNA_018413	1:48506309|48519415	circRNA_020379	1:59477666|59510085
circRNA_017074	3:90583580|90584203	circRNA_004638	6:103686782|103695506	circRNA_020555	12:35849683|35862791	circRNA_003563	15:85397016|85429438
circRNA_019969	11:21623421|21624044	circRNA_000257	1:283102747|283111472	circRNA_007676	4:102437791|102450907	circRNA_007942	7:97294164|97326586
circRNA_020540	12:6035933|6036557	circRNA_016301	7:73267046|73275773	circRNA_006244	5:109487613|109500733	circRNA_006875	11:21787387|21819817
circRNA_020924	2:145752197|145752821	circRNA_005212	GL892332.2:315558|324287	circRNA_011834	5:86279023|86292147	circRNA_015441	12:27535585|27568015
circRNA_001585	4:56555763|56556388	circRNA_020606	13:87157309|87166038	circRNA_016043	3:135192228|135205352	circRNA_017899	6:92070354|92102784
circRNA_010739	6:45499829|45500454	circRNA_021176	7:102964571|102973300	circRNA_014049	4:43055085|43068213	circRNA_014264	14:116579352|116611789
circRNA_018654	13:101379004|101379630	circRNA_021270	9:77190326|77199055	circRNA_003807	18:6130457|6143586	circRNA_013470	9:81468461|81500918
circRNA_005225	GL892930.1:17127|17754	circRNA_007573	3:90583580|90592312	circRNA_004458	5:70582741|70595881	circRNA_010915	GL894196.2:24010|56480
circRNA_008040	8:103708394|103709021	circRNA_000190	1:205495469|205504202	circRNA_003717	16:51312835|51325978	circRNA_020726	15:18557378|18589857
circRNA_008910	14:59597135|59597762	circRNA_017971	8:139806671|139815404	circRNA_010079	GL895478.1:35935|49080	circRNA_000964	15:86639052|86671532
circRNA_014936	5:5270800|5271427	circRNA_012503	12:62407440|62416175	circRNA_003938	2:82153520|82166666	circRNA_020476	1:282163496|282195982
circRNA_001541	4:1995100|1995728	circRNA_015001	15:36338366|36347101	circRNA_000545	13:25108325|25121472	circRNA_002648	1:195220278|195252772
circRNA_003947	2:84264529|84265157	circRNA_005220	GL892708.2:37775|46511	circRNA_003754	17:19828222|19841369	circRNA_019271	9:57662614|57695111
circRNA_008159	9:120380266|120380894	circRNA_000262	1:293488318|293497055	circRNA_020001	13:58558555|58571704	circRNA_013603	1:300110070|300142568
circRNA_004323	4:98563829|98564458	circRNA_010848	8:112636025|112644764	circRNA_015533	13:91499963|91513114	circRNA_017420	1:14572861|14605368
circRNA_004566	6:69990483|69991112	circRNA_009849	8:57794720|57803460	circRNA_005988	2:74628226|74641379	circRNA_007963	7:119761232|119793755
circRNA_010319	11:86932930|86933561	circRNA_006132	4:36900162|36908903	circRNA_007434	2:43651009|43664163	circRNA_021345	JH118802.1:2189|34725
circRNA_017365	GL893818.1:81441|82072	circRNA_019285	9:115748259|115757000	circRNA_013903	3:109719098|109732253	circRNA_004611	6:92078907|92111445
circRNA_018963	3:18348051|18348684	circRNA_002970	12:16062482|16071225	circRNA_012772	15:124776531|124789687	circRNA_013180	5:72062877|72095416
circRNA_008198	GL893783.2:10469|11103	circRNA_004182	3:114087675|114096420	circRNA_004676	6:141484093|141497256	circRNA_011814	5:50595002|50627564
circRNA_017721	17:43098460|43099094	circRNA_010575	2:87312978|87321723	circRNA_011332	14:58594277|58607441	circRNA_013873	18:49455064|49487626
circRNA_003285	14:948863|949498	circRNA_014898	X:67062592|67071339	circRNA_021243	9:9534133|9547298	circRNA_008164	9:127864210|127896783
circRNA_016611	X:130722670|130723305	circRNA_011366	14:116881370|116890119	circRNA_014620	10:2342778|2355944	circRNA_004306	4:88603967|88636542
circRNA_018060	X:115981245|115981880	circRNA_006052	3:3687221|3695971	circRNA_015257	1:218536643|218549814	circRNA_014981	12:48558810|48591386
circRNA_000852	14:120554176|120554812	circRNA_007689	4:117897403|117906153	circRNA_013158	5:32061014|32074186	circRNA_018132	13:119184171|119216757
circRNA_008344	1:101760962|101761598	circRNA_018932	2:109014594|109023345	circRNA_003622	15:118153598|118166774	circRNA_012235	1:106896702|106929326
circRNA_011069	1:293362010|293362646	circRNA_009700	6:138157842|138166597	circRNA_015223	1:176833200|176846376	circRNA_002763	1:301502546|301535195
circRNA_008310	1:40759887|40760525	circRNA_012200	1:31419390|31428147	circRNA_021072	5:88027154|88040330	circRNA_003202	13:130082229|130114879
circRNA_010925	JH118921.1:35284|35922	circRNA_017395	X:78197644|78206401	circRNA_004834	7:100759932|100773109	circRNA_021078	5:94418719|94451370
circRNA_015931	2:139593394|139594032	circRNA_019519	14:10474853|10483610	circRNA_016912	15:115453171|115466348	circRNA_002445	X:127709214|127741869
circRNA_017520	10:37816804|37817442	circRNA_011316	14:31014235|31022994	circRNA_000895	15:26926656|26939836	circRNA_004816	7:91917856|91950519
circRNA_021282	9:112401629|112402267	circRNA_012403	11:320232|328992	circRNA_002750	1:284289001|284302182	circRNA_013323	7:57914632|57947318
circRNA_009736	7:26692139|26692778	circRNA_001633	4:113285108|113293869	circRNA_020066	15:116343384|116356565	circRNA_013440	9:21378716|21411409
circRNA_015091	3:62257312|62257951	circRNA_008532	10:36699515|36708276	circRNA_019604	18:9534503|9547689	circRNA_018181	15:92672038|92704735
circRNA_004354	4:123208673|123209313	circRNA_015711	15:87446213|87454975	circRNA_010877	9:50821415|50834603	circRNA_016331	7:129078033|129110737
circRNA_020063	15:104105668|104106308	circRNA_001580	4:55278022|55286785	circRNA_007502	2:105133377|105146567	circRNA_005845	15:73484194|73516910
circRNA_020227	6:89927175|89927815	circRNA_018269	4:89250254|89259021	circRNA_002810	10:37118200|37131391	circRNA_017538	11:8551724|8584446
circRNA_017937	7:81291705|81292346	circRNA_002423	X:51899829|51908597	circRNA_014842	5:38338234|38351426	circRNA_013210	5:105276876|105309621
circRNA_011600	2:73695861|73696503	circRNA_011214	13:35734591|35743359	circRNA_002878	11:7809616|7822810	circRNA_000450	12:16030982|16063733
circRNA_012350	10:18597983|18598626	circRNA_017719	17:15905146|15913917	circRNA_005707	13:117558744|117571938	circRNA_002010	7:91949364|91982115
circRNA_015010	16:51813894|51814537	circRNA_017511	10:13605364|13614140	circRNA_006647	1:89934335|89947529	circRNA_006016	2:91513977|91546743
circRNA_018900	2:43065603|43066248	circRNA_017256	8:21853691|21862470	circRNA_018904	2:43925422|43938619	circRNA_009193	18:40626773|40659540
circRNA_008680	12:46857007|46857655	circRNA_015507	13:47757221|47766001	circRNA_021098	6:37870420|37883618	circRNA_015563	13:155534233|155567005
circRNA_005236	GL893821.1:2029|2678	circRNA_002794	10:29342271|29351054	circRNA_019923	1:132817550|132830751	circRNA_006671	1:110527369|110560153
circRNA_007240	15:62067358|62068007	circRNA_005183	9:133335567|133344356	circRNA_005732	13:149657446|149670651	circRNA_011849	5:103268724|103301526
circRNA_018701	14:13558704|13559353	circRNA_012431	11:21811028|21819817	circRNA_018253	3:40912876|40926084	circRNA_018271	4:107924568|107957391
circRNA_000081	1:103946010|103946660	circRNA_006097	3:107058409|107067199	circRNA_015708	15:85364480|85377689	circRNA_006424	8:93287563|93320426
circRNA_007956	7:106535537|106536187	circRNA_009487	4:106615781|106624571	circRNA_012250	1:126936876|126950090	circRNA_007803	6:35439133|35472022

circRNA_006782	1:282195331|282195982	circRNA_015995	3:76466177|76474967	circRNA_019981	12:36120985|36134199	circRNA_000968	15:91077981|91110875
circRNA_002328	GL893057.1:4350|5003	circRNA_004170	3:109719098|109727889	circRNA_009461	4:55390283|55403504	circRNA_013918	4:117913001|117945925
circRNA_010018	9:137078424|137079077	circRNA_009481	4:93613223|93622015	circRNA_002288	9:127883559|127896783	circRNA_013060	3:130387896|130420826
circRNA_014102	1:50516496|50517149	circRNA_013891	2:125520281|125529074	circRNA_006654	1:96792819|96806044	circRNA_012727	15:73548305|73581238
circRNA_016711	10:11772748|11773401	circRNA_007430	2:28869234|28878034	circRNA_004278	4:55390276|55403504	circRNA_006030	2:119165841|119198795
circRNA_002656	1:200299009|200299664	circRNA_018255	3:72511167|72519969	circRNA_011291	13:207175610|207188840	circRNA_010204	1:132967048|133000011
circRNA_017851	4:141346800|141347456	circRNA_010067	GL894772.1:20848|29651	circRNA_020171	4:40740616|40753847	circRNA_012342	10:16968012|17000982
circRNA_020292	8:82927747|82928404	circRNA_018804	15:120865237|120874040	circRNA_007123	14:50021607|50034842	circRNA_008170	9:132262224|132295195
circRNA_017403	X:112705483|112706141	circRNA_014591	X:14171526|14180333	circRNA_002885	11:11212642|11225878	circRNA_003930	2:79837584|79870561
circRNA_019309	GL893689.1:63096|63754	circRNA_013845	15:55337963|55346771	circRNA_016901	15:85074728|85087964	circRNA_011611	2:87952595|87985584
circRNA_020547	12:19563984|19564642	circRNA_018622	13:20908175|20916983	circRNA_018488	1:202605803|202619041	circRNA_009563	5:70588009|70621000
circRNA_012891	18:59695386|59696045	circRNA_001355	2:142522062|142530871	circRNA_002117	8:91602564|91615803	circRNA_005604	11:75060899|75093907
circRNA_016772	12:52869369|52870028	circRNA_010091	JH118940.1:66614|75425	circRNA_007025	13:91026859|91040099	circRNA_003705	16:47778049|47811066
circRNA_009571	5:74574241|74574901	circRNA_002653	1:199991719|200000535	circRNA_008948	14:115744138|115757379	circRNA_009165	18:1942538|1975555
circRNA_012601	13:177703889|177704549	circRNA_003260	13:207007605|207016422	circRNA_005582	11:15979885|15993129	circRNA_001870	6:99514655|99547687
circRNA_014296	15:92420042|92420702	circRNA_014028	7:105387843|105396660	circRNA_014975	11:495509|508754	circRNA_001955	7:31316350|31349385
circRNA_004795	7:69495691|69496352	circRNA_015856	2:3011482|3020299	circRNA_007543	3:29713966|29727215	circRNA_014018	3:134635120|134668164
circRNA_006754	1:227185988|227186649	circRNA_012939	2:80398497|80407317	circRNA_007985	8:12704089|12717344	circRNA_018313	6:90434708|90467752
circRNA_004707	6:152903688|152904351	circRNA_009092	16:29638099|29646923	circRNA_001076	16:75043882|75057142	circRNA_009184	18:16660735|16693800
circRNA_004863	7:129818641|129819304	circRNA_010964	1:40758732|40767556	circRNA_004293	4:72908013|72921273	circRNA_002507	1:60908344|60941431
circRNA_018007	9:137085313|137085976	circRNA_016369	8:57810131|57818958	circRNA_012083	9:120349846|120363114	circRNA_003429	14:117074459|117107550
circRNA_001755	5:84016419|84017083	circRNA_014764	1:163944258|163953086	circRNA_001463	3:92468130|92481403	circRNA_017913	6:139878851|139911949
circRNA_013689	14:7176205|7176869	circRNA_008104	9:39970141|39978970	circRNA_003258	13:206119895|206133168	circRNA_019226	8:103678982|103712082
circRNA_008250	X:46343033|46343698	circRNA_005195	9:139653163|139661994	circRNA_007555	3:58975400|58988675	circRNA_014188	13:14307735|14340843
circRNA_001945	7:2977563|2978229	circRNA_015807	18:5554189|5563021	circRNA_009935	9:36653995|36667272	circRNA_016629	1:58484449|58517592
circRNA_002411	X:17758636|17759302	circRNA_011813	5:49566730|49575564	circRNA_009774	7:73270655|73283935	circRNA_011470	16:24960988|24994149
circRNA_005090	9:31829253|31829919	circRNA_015741	15:157089364|157098198	circRNA_010778	7:112298|125579	circRNA_017181	6:88257154|88290327
circRNA_021155	7:26984596|26985262	circRNA_009661	6:86657694|86666530	circRNA_018117	12:20097144|20110425	circRNA_010968	1:62694950|62728124
circRNA_005202	9:143423352|143424019	circRNA_006900	11:76193953|76202790	circRNA_015625	14:65183475|65196759	circRNA_007021	13:90919603|90952792
circRNA_008137	9:85105697|85106364	circRNA_010087	GL896549.1:14676|23514	circRNA_015932	2:140344565|140357849	circRNA_005716	13:138907625|138940815
circRNA_018860	18:8116985|8117652	circRNA_007019	13:90686409|90695250	circRNA_014449	5:54912755|54926044	circRNA_021309	GL893759.2:32870|66061
circRNA_007417	2:5185942|5186610	circRNA_001030	15:152336067|152344909	circRNA_009834	8:32882663|32895953	circRNA_012210	1:45701230|45734427
circRNA_006980	13:33174778|33175447	circRNA_010222	1:195020817|195029660	circRNA_016816	13:131013234|131026525	circRNA_013534	GL894982.1:12114|45312
circRNA_008921	14:69441478|69442147	circRNA_013122	4:116008617|116017460	circRNA_017371	GL894519.2:77713|91007	circRNA_016121	5:2734257|2767456
circRNA_016286	7:45097140|45097809	circRNA_017380	JH118669.1:196964|205808	circRNA_005130	9:73323019|73336315	circRNA_011694	3:73604709|73637911
circRNA_001897	6:125781000|125781670	circRNA_016100	4:115670774|115679624	circRNA_009106	16:47813571|47826868	circRNA_015128	5:76358127|76391339
circRNA_007480	2:87308480|87309150	circRNA_000550	13:28306363|28315215	circRNA_003497	15:18728205|18741503	circRNA_005116	9:49371098|49404311
circRNA_000991	15:115465676|115466348	circRNA_002517	1:72497312|72506164	circRNA_010072	GL894982.1:32013|45312	circRNA_017709	16:36595410|36628627
circRNA_005474	1:186657166|186657838	circRNA_006085	3:84254791|84263643	circRNA_002337	GL893841.1:32135|45436	circRNA_011644	2:146067464|146100698
circRNA_001893	6:113681718|113682391	circRNA_002650	1:195518425|195527278	circRNA_003134	13:67568901|67582203	circRNA_021117	6:92978958|93012200
circRNA_001534	3:134711008|134711682	circRNA_004760	7:43188490|43197345	circRNA_007035	13:103719305|103732607	circRNA_016194	6:16269873|16303134
circRNA_007656	4:63085081|63085755	circRNA_020795	16:53255847|53264702	circRNA_015092	3:72409632|72422934	circRNA_010895	9:114852775|114886040
circRNA_014109	1:103215127|103215801	circRNA_007976	7:133993467|134002323	circRNA_006835	10:49672328|49685631	circRNA_016241	6:98763605|98796872
circRNA_001234	2:30491227|30491902	circRNA_011089	10:26264515|26273371	circRNA_019592	16:53235594|53248903	circRNA_011221	13:49972583|50005869
circRNA_012095	9:141691661|141692338	circRNA_011135	11:15976293|15985150	circRNA_013716	4:129522757|129536068	circRNA_007574	3:90732525|90765820
circRNA_015777	16:56974369|56975046	circRNA_018596	12:27559138|27567997	circRNA_007080	13:192359764|192373082	circRNA_005857	15:105450884|105484194
circRNA_011565	2:5926645|5927323	circRNA_020512	10:53856831|53865690	circRNA_001356	2:142522062|142535381	circRNA_008430	1:202490137|202523454
circRNA_020479	1:283056939|283057617	circRNA_004377	4:134762147|134771008	circRNA_005517	1:270329795|270343116	circRNA_000548	13:28113282|28146602
circRNA_002533	1:89946850|89947529	circRNA_009998	9:102775845|102784706	circRNA_018992	3:92530938|92544259	circRNA_021133	6:134517053|134550382
circRNA_003011	12:37710316|37710996	circRNA_021035	4:135544493|135553354	circRNA_002500	1:56096587|56109914	circRNA_009313	2:142522062|142555400
circRNA_020448	1:202605803|202606483	circRNA_009039	15:91614843|91623705	circRNA_000171	1:200325282|200338610	circRNA_006437	8:144641851|144675196
circRNA_004395	4:143313443|143314129	circRNA_004156	3:103056866|103065729	circRNA_013479	9:101936355|101949686	circRNA_014077	8:147991677|148025041

<table>
<tr><td>circRNA_003731</td><td>16:70016115|70016802</td><td>circRNA_012153</td><td>X:51823235|51832102</td><td>circRNA_019257</td><td>9:37686040|37699385</td><td>circRNA_012494</td><td>12:44463879|44497245</td></tr>
<tr><td>circRNA_010271</td><td>10:19355988|19356675</td><td>circRNA_011839</td><td>5:87362392|87371260</td><td>circRNA_003193</td><td>13:117567899|117581247</td><td>circRNA_015845</td><td>18:53703979|53737345</td></tr>
<tr><td>circRNA_006979</td><td>13:29361328|29362016</td><td>circRNA_010025</td><td>9:142460849|142469718</td><td>circRNA_017110</td><td>4:55211975|55225326</td><td>circRNA_005518</td><td>1:270442985|270476354</td></tr>
<tr><td>circRNA_000160</td><td>1:180714491|180715180</td><td>circRNA_005134</td><td>9:78890360|78899230</td><td>circRNA_017157</td><td>5:109516321|109529675</td><td>circRNA_020055</td><td>15:35923327|35956714</td></tr>
<tr><td>circRNA_001804</td><td>6:50530897|50531586</td><td>circRNA_010889</td><td>9:78740877|78749748</td><td>circRNA_018554</td><td>10:54209843|54223208</td><td>circRNA_013281</td><td>6:139914643|139948032</td></tr>
<tr><td>circRNA_014332</td><td>17:44564987|44565676</td><td>circRNA_004389</td><td>4:139855414|139864288</td><td>circRNA_016036</td><td>3:128287822|128301190</td><td>circRNA_016157</td><td>5:68113554|68146959</td></tr>
<tr><td>circRNA_000901</td><td>15:36443208|36443898</td><td>circRNA_018661</td><td>13:131119326|131128200</td><td>circRNA_018294</td><td>5:93485749|93499119</td><td>circRNA_009686</td><td>6:103715572|103748981</td></tr>
<tr><td>circRNA_003878</td><td>2:6984578|6985268</td><td>circRNA_002991</td><td>12:27559138|27568015</td><td>circRNA_004185</td><td>3:117257014|117270385</td><td>circRNA_018738</td><td>14:66861565|66894997</td></tr>
<tr><td>circRNA_014266</td><td>14:136306844|136307534</td><td>circRNA_012871</td><td>18:25511592|25520470</td><td>circRNA_000881</td><td>15:3788416|3801791</td><td>circRNA_001333</td><td>2:121372041|121405489</td></tr>
<tr><td>circRNA_015201</td><td>1:115244034|115244724</td><td>circRNA_018474</td><td>1:181380655|181389533</td><td>circRNA_002320</td><td>GL892430.1:39370|52745</td><td>circRNA_019915</td><td>1:84258390|84291857</td></tr>
<tr><td>circRNA_013002</td><td>3:42066184|42066875</td><td>circRNA_004902</td><td>8:33308379|33317260</td><td>circRNA_012481</td><td>12:27599876|27613254</td><td>circRNA_005002</td><td>8:123981625|124015123</td></tr>
<tr><td>circRNA_015577</td><td>13:202714032|202714724</td><td>circRNA_002162</td><td>8:144739680|144748563</td><td>circRNA_014639</td><td>13:84710600|84723981</td><td>circRNA_000608</td><td>13:84626456|84659957</td></tr>
<tr><td>circRNA_017208</td><td>6:145878565|145879257</td><td>circRNA_007997</td><td>8:33308376|33317260</td><td>circRNA_008914</td><td>14:59874821|59888204</td><td>circRNA_019728</td><td>5:50627413|50660921</td></tr>
<tr><td>circRNA_012089</td><td>9:136557895|136558588</td><td>circRNA_001995</td><td>7:73042261|73051146</td><td>circRNA_003841</td><td>18:25751809|25765194</td><td>circRNA_009697</td><td>6:136000651|136034171</td></tr>
<tr><td>circRNA_018913</td><td>2:69854762|69855455</td><td>circRNA_004852</td><td>7:119957967|119966852</td><td>circRNA_012626</td><td>14:31063858|31077243</td><td>circRNA_018130</td><td>13:98113084|98146604</td></tr>
<tr><td>circRNA_015104</td><td>8:81877619|81878313</td><td>circRNA_005030</td><td>8:144739677|144748563</td><td>circRNA_013350</td><td>7:105383271|105396660</td><td>circRNA_002580</td><td>1:132962462|132995990</td></tr>
<tr><td>circRNA_003459</td><td>14:135630658|135631353</td><td>circRNA_019206</td><td>8:40334723|40343610</td><td>circRNA_000282</td><td>10:13600749|13614140</td><td>circRNA_019432</td><td>10:58589942|58623477</td></tr>
<tr><td>circRNA_006781</td><td>1:281729287|281729982</td><td>circRNA_000928</td><td>15:67051474|67060363</td><td>circRNA_016410</td><td>8:142120245|142133636</td><td>circRNA_006849</td><td>10:62722156|62755695</td></tr>
<tr><td>circRNA_017485</td><td>1:202815351|202816046</td><td>circRNA_007269</td><td>15:106836857|106845749</td><td>circRNA_000084</td><td>1:110525008|110538400</td><td>circRNA_013278</td><td>6:137828146|137861689</td></tr>
<tr><td>circRNA_007810</td><td>6:40811520|40812217</td><td>circRNA_014701</td><td>4:73580862|73589754</td><td>circRNA_010084</td><td>GL896372.1:1040|14432</td><td>circRNA_004639</td><td>6:103686782|103720331</td></tr>
<tr><td>circRNA_012021</td><td>8:120764216|120764913</td><td>circRNA_014477</td><td>6:86657694|86666587</td><td>circRNA_012623</td><td>14:30053619|30067011</td><td>circRNA_008345</td><td>1:103215127|103248684</td></tr>
<tr><td>circRNA_002593</td><td>1:141225456|141226154</td><td>circRNA_002429</td><td>X:73933282|73942176</td><td>circRNA_000796</td><td>14:65832702|65846096</td><td>circRNA_007915</td><td>7:57934614|57968188</td></tr>
<tr><td>circRNA_007419</td><td>2:9629379|9630077</td><td>circRNA_009836</td><td>8:34753681|34762575</td><td>circRNA_011795</td><td>4:136390669|136404063</td><td>circRNA_021198</td><td>8:44869888|44903479</td></tr>
<tr><td>circRNA_004474</td><td>5:78224359|78225058</td><td>circRNA_020324</td><td>9:79152745|79161639</td><td>circRNA_009574</td><td>5:79772211|79785608</td><td>circRNA_008841</td><td>13:167164236|167197828</td></tr>
<tr><td>circRNA_015550</td><td>13:142834061|142834760</td><td>circRNA_006506</td><td>9:127746262|127755157</td><td>circRNA_017017</td><td>2:52228455|52241853</td><td>circRNA_014593</td><td>X:15924705|15958311</td></tr>
<tr><td>circRNA_017399</td><td>X:103660428|103661127</td><td>circRNA_003924</td><td>2:71193761|71202659</td><td>circRNA_012644</td><td>14:52830738|52844139</td><td>circRNA_003168</td><td>13:90919603|90953214</td></tr>
<tr><td>circRNA_020038</td><td>14:51757375|51758074</td><td>circRNA_010289</td><td>10:56109733|56118632</td><td>circRNA_000984</td><td>15:106832344|106845749</td><td>circRNA_006313</td><td>6:150861612|150895226</td></tr>
<tr><td>circRNA_012041</td><td>9:10154556|10155256</td><td>circRNA_004598</td><td>6:85446257|85455157</td><td>circRNA_009095</td><td>16:36617706|36631111</td><td>circRNA_003575</td><td>15:86637917|86671532</td></tr>
<tr><td>circRNA_018879</td><td>18:41381384|41382084</td><td>circRNA_000575</td><td>13:42311419|42320321</td><td>circRNA_014878</td><td>8:71459662|71473071</td><td>circRNA_010708</td><td>5:63631299|63664917</td></tr>
<tr><td>circRNA_000202</td><td>1:226117819|226118520</td><td>circRNA_001032</td><td>16:19127489|19136391</td><td>circRNA_013427</td><td>8:144674980|144688396</td><td>circRNA_007245</td><td>15:72682970|72716592</td></tr>
<tr><td>circRNA_001354</td><td>2:142522062|142522763</td><td>circRNA_003219</td><td>13:144113033|144121936</td><td>circRNA_019947</td><td>1:282121073|282134489</td><td>circRNA_004837</td><td>7:102789030|102822653</td></tr>
<tr><td>circRNA_011292</td><td>13:209915557|209916258</td><td>circRNA_018615</td><td>12:62424103|62433008</td><td>circRNA_003455</td><td>14:133479278|133492695</td><td>circRNA_008834</td><td>13:149623912|149657541</td></tr>
<tr><td>circRNA_015881</td><td>2:50485447|50486148</td><td>circRNA_016900</td><td>15:84975710|84984616</td><td>circRNA_009505</td><td>4:136390669|136404088</td><td>circRNA_018024</td><td>GL893841.1:29395|63035</td></tr>
<tr><td>circRNA_016443</td><td>9:29704904|29705606</td><td>circRNA_013219</td><td>6:13097978|13106885</td><td>circRNA_008761</td><td>13:58776339|58789759</td><td>circRNA_018626</td><td>13:32176941|32210587</td></tr>
<tr><td>circRNA_004587</td><td>6:82348232|82348935</td><td>circRNA_014814</td><td>2:15925033|15933940</td><td>circRNA_008610</td><td>11:53841367|53854788</td><td>circRNA_016357</td><td>8:43550245|43583894</td></tr>
<tr><td>circRNA_016950</td><td>16:77439557|77440260</td><td>circRNA_004392</td><td>4:141572612|141581521</td><td>circRNA_018513</td><td>1:257023665|257037086</td><td>circRNA_016948</td><td>16:75043882|75077536</td></tr>
<tr><td>circRNA_011704</td><td>3:90555361|90556065</td><td>circRNA_004999</td><td>8:120759223|120768132</td><td>circRNA_010792</td><td>7:61661239|61674662</td><td>circRNA_002070</td><td>8:32587989|32621660</td></tr>
<tr><td>circRNA_003289</td><td>14:6906858|6907564</td><td>circRNA_017326</td><td>9:51065971|51074882</td><td>circRNA_008883</td><td>14:30922612|30936038</td><td>circRNA_021297</td><td>9:143484202|143517883</td></tr>
<tr><td>circRNA_017464</td><td>1:155380720|155381426</td><td>circRNA_008722</td><td>13:26014191|26023104</td><td>circRNA_013888</td><td>2:119185369|119198795</td><td>circRNA_005631</td><td>12:45463896|45497578</td></tr>
<tr><td>circRNA_004453</td><td>5:69495867|69496574</td><td>circRNA_002356</td><td>GL894965.2:40010|48924</td><td>circRNA_001360</td><td>2:142535263|142548691</td><td>circRNA_020096</td><td>18:8655702|8689385</td></tr>
<tr><td>circRNA_000663</td><td>13:141245887|141246595</td><td>circRNA_010872</td><td>9:39971915|39980829</td><td>circRNA_016993</td><td>18:49341237|49354669</td><td>circRNA_004018</td><td>2:136407725|136441429</td></tr>
<tr><td>circRNA_012797</td><td>16:27423986|27424694</td><td>circRNA_004134</td><td>3:84984397|84993312</td><td>circRNA_005715</td><td>13:132319701|132333134</td><td>circRNA_006881</td><td>11:26002135|26035840</td></tr>
<tr><td>circRNA_015650</td><td>14:94925010|94925718</td><td>circRNA_002379</td><td>GL896504.1:24299|33216</td><td>circRNA_013294</td><td>6:152878863|152892298</td><td>circRNA_004414</td><td>5:20667769|20701486</td></tr>
<tr><td>circRNA_009013</td><td>15:66048762|66049471</td><td>circRNA_003710</td><td>16:47791556|47800473</td><td>circRNA_007683</td><td>4:108244663|108258099</td><td>circRNA_013381</td><td>8:42394633|42428350</td></tr>
<tr><td>circRNA_001343</td><td>2:130854885|130855596</td><td>circRNA_020057</td><td>15:73548305|73557223</td><td>circRNA_001693</td><td>5:22719002|22732443</td><td>circRNA_007973</td><td>7:128927160|128960887</td></tr>
<tr><td>circRNA_009190</td><td>18:27446734|27447445</td><td>circRNA_007591</td><td>3:107119004|107127924</td><td>circRNA_005475</td><td>1:195020817|195034259</td><td>circRNA_003819</td><td>18:15001029|15034759</td></tr>
<tr><td>circRNA_004076</td><td>3:39564463|39565175</td><td>circRNA_016263</td><td>6:148044322|148053243</td><td>circRNA_016326</td><td>7:123602136|123615582</td><td>circRNA_011490</td><td>16:47791556|47825304</td></tr>
<tr><td>circRNA_016296</td><td>7:64693055|64693769</td><td>circRNA_017358</td><td>GL892755.1:93846|102767</td><td>circRNA_004133</td><td>3:84817941|84831391</td><td>circRNA_015742</td><td>16:4481513|4515273</td></tr>
<tr><td>circRNA_016690</td><td>1:256814563|256815277</td><td>circRNA_016532</td><td>GL894597.1:48506|57428</td><td>circRNA_002627</td><td>1:169119051|169132503</td><td>circRNA_021251</td><td>9:22937466|22971228</td></tr>
<tr><td>circRNA_016895</td><td>15:59810876|59811590</td><td>circRNA_019468</td><td>12:62124628|62133550</td><td>circRNA_009964</td><td>9:46861375|46874828</td><td>circRNA_020906</td><td>2:120705612|120739379</td></tr>
<tr><td>circRNA_007665</td><td>4:73577490|73578205</td><td>circRNA_007592</td><td>3:109401412|109410336</td><td>circRNA_017605</td><td>13:117326011|117339469</td><td>circRNA_012926</td><td>2:54339863|54373632</td></tr>
<tr><td>circRNA_012686</td><td>14:118363984|118364699</td><td>circRNA_020042</td><td>14:77624653|77633580</td><td>circRNA_019477</td><td>13:44073064|44086522</td><td>circRNA_008491</td><td>1:284289001|284322778</td></tr>
</table>

circRNA_014450	5:57064654|57065369	circRNA_008878	14:14027675|14036603	circRNA_009094	16:34274758|34288217	circRNA_020516	10:55751216|55784996
circRNA_007793	6:18420888|18421604	circRNA_001723	5:61467036|61475967	circRNA_012097	9:143268621|143282082	circRNA_014331	17:43359995|43393779
circRNA_011661	3:17103410|17104126	circRNA_006380	8:390769|399701	circRNA_007736	5:35903030|35916494	circRNA_003218	13:144052007|144085795
circRNA_016226	6:82735106|82735822	circRNA_010528	17:43615013|43623946	circRNA_005899	16:51775688|51789153	circRNA_020087	17:10387631|10421420
circRNA_009352	3:44877899|44878616	circRNA_010836	8:58689278|58698211	circRNA_001438	3:72580187|72593654	circRNA_001878	6:101779796|101813590
circRNA_015485	13:28828409|28829126	circRNA_013342	7:94524184|94533117	circRNA_003973	2:92576700|92590171	circRNA_005137	9:81468461|81502257
circRNA_019940	1:247699026|247699743	circRNA_009761	7:65184011|65192947	circRNA_004989	8:108424880|108438354	circRNA_000609	13:86459133|86492936
circRNA_002241	9:69056402|69057120	circRNA_002540	1:94568797|94577735	circRNA_001533	3:134654689|134668164	circRNA_019791	7:36438654|36472463
circRNA_002326	GL892789.1:93851|94569	circRNA_017682	15:106491919|106500857	circRNA_007189	14:132243465|132256940	circRNA_019987	12:62413971|62447784
circRNA_003381	14:77896241|77896962	circRNA_020069	15:144953199|144962138	circRNA_017926	7:33665914|33679390	circRNA_000366	10:76272294|76306111
circRNA_005145	9:101637290|101638011	circRNA_019451	11:53982386|53991328	circRNA_008981	15:18327032|18340515	circRNA_010596	2:139165773|139199596
circRNA_010100	MT:2757|3478	circRNA_013751	18:44787541|44796486	circRNA_013554	JH118963.1:42907|56390	circRNA_016025	3:115433791|115467615
circRNA_016579	X:21584133|21584854	circRNA_000945	15:79683656|79692603	circRNA_021134	6:138486476|138499959	circRNA_014280	15:66886362|66920187
circRNA_015722	15:115982270|115982993	circRNA_007053	13:132975613|132984562	circRNA_009152	17:44388746|44402233	circRNA_004291	4:72820300|72854126
circRNA_006596	1:4264348|4265072	circRNA_008424	1:200325282|200334231	circRNA_015700	15:67042882|67056369	circRNA_008829	13:145052451|145086280
circRNA_007770	5:83018001|83018725	circRNA_012768	15:117869607|117878557	circRNA_019552	14:122682984|122696472	circRNA_008143	9:98614769|98648601
circRNA_014714	6:871815|872539	circRNA_009219	2:11070312|11079263	circRNA_020832	18:16898135|16911623	circRNA_000427	11:70156594|70190434
circRNA_015142	1:16421005|16421729	circRNA_017063	3:42806686|42815639	circRNA_005076	9:22396993|22410483	circRNA_008366	1:127997793|128031634
circRNA_014159	10:48232668|48233393	circRNA_001449	3:77625137|77634093	circRNA_004501	5:94425846|94439340	circRNA_015553	13:144076090|144109931
circRNA_001807	6:57980044|57980770	circRNA_007092	14:7176205|7185161	circRNA_016924	16:19080932|19094426	circRNA_008627	11:76367637|76401491
circRNA_015512	13:57863679|57864406	circRNA_018693	13:210047704|210056662	circRNA_000124	1:134656768|134670263	circRNA_015466	12:59366760|59400625
circRNA_001361	2:143111063|143111792	circRNA_005132	9:78693459|78702418	circRNA_021159	7:33242142|33255637	circRNA_000556	13:33494610|33528483
circRNA_015327	10:16602454|16603183	circRNA_008130	9:78768002|78776962	circRNA_001753	5:82625769|82639265	circRNA_008393	1:161219334|161253222
circRNA_015662	14:118370410|118371139	circRNA_001250	2:54339863|54348827	circRNA_006166	4:103237684|103251183	circRNA_001660	4:134737116|134771008
circRNA_017977	9:9484401|9485130	circRNA_009917	9:9476165|9485130	circRNA_017123	4:115779111|115792611	circRNA_018209	18:42882852|42916749
circRNA_009739	7:36446703|36447433	circRNA_002903	11:22240397|22249363	circRNA_003411	14:98503845|98517347	circRNA_014104	1:83289162|83323066
circRNA_021076	5:92460533|92461263	circRNA_002997	12:36535677|36544644	circRNA_019948	1:282182480|282195982	circRNA_004271	4:45690143|45724049
circRNA_015745	16:23497337|23498068	circRNA_008632	12:6051284|6060253	circRNA_008036	8:93306923|93320426	circRNA_000470	12:26712527|26746441
circRNA_006948	12:46904604|46905337	circRNA_016459	9:44764905|44773874	circRNA_003513	15:51637238|51650743	circRNA_004712	7:4587208|4621123
circRNA_015800	17:57298388|57299121	circRNA_007682	4:107957216|107966186	circRNA_005817	14:141164858|141178363	circRNA_001165	18:18641519|18675443
circRNA_018962	3:18267529|18268263	circRNA_009859	8:75770134|75779107	circRNA_016705	1:300377341|300390846	circRNA_004813	7:89101045|89134969
circRNA_009073	15:156302559|156303295	circRNA_010259	10:15627096|15636069	circRNA_020203	5:77630305|77643810	circRNA_013784	1:222016017|222049942
circRNA_018308	6:69806635|69807371	circRNA_006977	13:28757838|28766813	circRNA_015886	2:64745949|64759456	circRNA_017965	8:112610833|112644764
circRNA_000654	13:131237145|131237882	circRNA_012680	14:115748404|115757379	circRNA_013567	X:17736713|17750223	circRNA_012690	14:124422938|124456895
circRNA_002243	9:71420669|71421406	circRNA_015329	10:18583351|18592326	circRNA_020492	10:9884743|9898253	circRNA_016813	13:119202413|119236371
circRNA_010572	2:73909891|73910628	circRNA_007031	13:97298407|97307385	circRNA_004941	8:69857712|69871223	circRNA_004410	5:16660983|16694950
circRNA_012145	JH118940.1:98091|98829	circRNA_017033	2:89987134|89996112	circRNA_010207	1:134656752|134670263	circRNA_021013	4:55199068|55233036
circRNA_017448	1:119998397|119999135	circRNA_015527	13:90896704|90905685	circRNA_018531	10:15244827|15258338	circRNA_003581	15:91589714|91623705
circRNA_018662	13:131136658|131137396	circRNA_011493	16:51783754|51792737	circRNA_000633	13:108357995|108371507	circRNA_010653	4:33426747|33460740
circRNA_001223	2:16545939|16546678	circRNA_004049	3:5155280|5164265	circRNA_015338	10:35288939|35302453	circRNA_017264	8:32861923|32895953
circRNA_005240	GL894083.2:5621|6360	circRNA_014262	14:115555579|115564564	circRNA_012101	GL892805.1:54588|68104	circRNA_014629	11:24202794|24236831
circRNA_016161	5:72530032|72530771	circRNA_015968	3:46262974|46271960	circRNA_011744	4:19827815|19841332	circRNA_003982	2:98306632|98340685
circRNA_000380	11:5454621|5455362	circRNA_017355	GL892680.2:154968|163954	circRNA_004110	3:75742883|75756405	circRNA_018210	18:52262210|52296298
circRNA_004269	4:45282363|45283104	circRNA_016838	14:31262408|31271395	circRNA_003829	18:18661914|18675443	circRNA_014673	16:70068372|70102462
circRNA_009785	7:89134396|89135137	circRNA_000107	1:127652319|127661307	circRNA_016526	GL894351.1:4122|17652	circRNA_010457	14:136012794|136046886
circRNA_010558	2:8449836|8450577	circRNA_008809	13:117594277|117603266	circRNA_003118	13:43762967|43776498	circRNA_014865	7:89101045|89135137
circRNA_002770	10:15048938|15049680	circRNA_017697	16:18574324|18583314	circRNA_015844	18:49341138|49354669	circRNA_020629	13:133911503|133945602
circRNA_005284	GL896549.1:14676|15418	circRNA_009083	16:24823085|24832076	circRNA_011643	2:145727555|145741087	circRNA_015307	1:284320474|284354583
circRNA_007898	7:14146603|14147345	circRNA_009882	8:109051187|109060178	circRNA_002554	1:115149639|115163177	circRNA_016481	9:79847931|79882043
circRNA_014020	5:15487161|15487903	circRNA_005870	15:120976533|120985525	circRNA_020708	14:120779066|120792605	circRNA_021065	5:80185715|80219831
circRNA_021101	6:40609993|40610735	circRNA_013943	7:60968354|60977347	circRNA_013951	8:58292028|58305568	circRNA_009347	3:42032747|42066875
circRNA_015053	8:57997327|57998070	circRNA_019148	7:14662608|14671601	circRNA_020887	2:82552204|82565744	circRNA_001140	18:5252251|5286395

circRNA_002376	GL896447.1:26049|26793	circRNA_010984	1:119589318|119598313	circRNA_002206	9:29830958|29844499	circRNA_015375	11:18571298|18605445
circRNA_013239	6:50556627|50557371	circRNA_004283	4:67884090|67893087	circRNA_001956	7:33100021|33113564	circRNA_004976	8:93207545|93241704
circRNA_001062	16:47329782|47330528	circRNA_011921	6:156310075|156319073	circRNA_006993	13:43454862|43468409	circRNA_011243	13:97270169|97304344
circRNA_007783	6:869929|870675	circRNA_002771	10:15627070|15636069	circRNA_012935	2:74366115|74379662	circRNA_004749	7:36377897|36412083
circRNA_009606	6:871815|872561	circRNA_004778	7:62827072|62836071	circRNA_007303	16:23761178|23774738	circRNA_014344	18:36472459|36506655
circRNA_009887	8:120826514|120827260	circRNA_003464	14:140974850|140983853	circRNA_016718	10:18250054|18263614	circRNA_006742	1:208314837|208349034
circRNA_015320	10:34341|35087	circRNA_006587	X:140031246|140040249	circRNA_003861	18:49341108|49354669	circRNA_007705	4:139822139|139856337
circRNA_019413	1:282666520|282667267	circRNA_012869	18:21609095|21618099	circRNA_015387	11:25049553|25063114	circRNA_009097	16:36664765|36698971
circRNA_001540	3:140437843|140438591	circRNA_001809	6:63443230|63452235	circRNA_006163	4:98120655|98134217	circRNA_007173	14:115712680|115746889
circRNA_012204	1:35232316|35233064	circRNA_010105	X:3681563|3690568	circRNA_002755	1:288073705|288087269	circRNA_007845	6:99120942|99155163
circRNA_015215	1:136789248|136789996	circRNA_004292	4:72832383|72841389	circRNA_007017	13:90496250|90509814	circRNA_013132	4:129268028|129302281
circRNA_002220	9:42882942|42883693	circRNA_020300	8:140242798|140251806	circRNA_016970	18:9836652|9850217	circRNA_001692	5:20667769|20702029
circRNA_004969	8:89343634|89344386	circRNA_016316	7:102425698|102434708	circRNA_019529	14:59040543|59054110	circRNA_006447	9:10202464|10236726
circRNA_003558	15:85325487|85326240	circRNA_018478	1:186650215|186659227	circRNA_003771	17:41831450|41845019	circRNA_007565	3:81716210|81750482
circRNA_016969	18:6259506|6260259	circRNA_005764	14:14019138|14028151	circRNA_014119	1:144470561|144484131	circRNA_018866	18:15020215|15054489
circRNA_005955	18:46693648|46694402	circRNA_020579	13:34173598|34182614	circRNA_002730	1:266592910|266606482	circRNA_003039	12:49350315|49384594
circRNA_017914	6:143706339|143707093	circRNA_012581	13:130256872|130265889	circRNA_011836	5:87106749|87120323	circRNA_020689	14:68177959|68212247
circRNA_018721	14:41932585|41933339	circRNA_004997	8:118376130|118385149	circRNA_016116	4:137112072|137125646	circRNA_021257	9:40209557|40243856
circRNA_007007	13:79420085|79420841	circRNA_008314	1:45652265|45661284	circRNA_011256	13:131093962|131107538	circRNA_012928	2:59237034|59271334
circRNA_010758	6:119320977|119321733	circRNA_003750	17:15472252|15481272	circRNA_009056	15:124765665|124779242	circRNA_009269	2:87176958|87211265
circRNA_011407	15:66043871|66044627	circRNA_003220	13:144272713|144281734	circRNA_017706	16:29718442|29732020	circRNA_016651	1:141763126|141797448
circRNA_010286	10:53520136|53520893	circRNA_018679	13:166795034|166804059	circRNA_007776	5:91482491|91496070	circRNA_003481	14:147004006|147038331
circRNA_016682	1:232792268|232793025	circRNA_007183	14:122832506|122841532	circRNA_016247	6:101256515|101270094	circRNA_003271	13:210819581|210853912
circRNA_005616	12:19366398|19367156	circRNA_009590	5:91474738|91483764	circRNA_008410	1:181145447|181159028	circRNA_018356	8:122154137|122188479
circRNA_007182	14:122455302|122456060	circRNA_019917	1:84583469|84592497	circRNA_008151	9:113153990|113167573	circRNA_004411	5:16670864|16705209
circRNA_020669	14:21508995|21509754	circRNA_013410	8:112417315|112426346	circRNA_006367	7:119676436|119690020	circRNA_014605	X:122513974|122548340
circRNA_001521	3:122517780|122518540	circRNA_020050	14:122682984|122692016	circRNA_005896	16:47267573|47281159	circRNA_015895	2:85455814|85490186
circRNA_013998	12:49749481|49750243	circRNA_017793	3:56415476|56424509	circRNA_005063	9:13245753|13259345	circRNA_008763	13:59859476|59893851
circRNA_011007	1:142617449|142618213	circRNA_002048	7:129078033|129087067	circRNA_013043	3:104199592|104213184	circRNA_006997	13:52502787|52537175
circRNA_020877	2:60731687|60732452	circRNA_015514	13:59664601|59673639	circRNA_020826	18:6035144|6048736	circRNA_003246	13:168499283|168533672
circRNA_000193	1:212177996|212178762	circRNA_006110	3:117261346|117270385	circRNA_012112	GL893511.2:215624|229217	circRNA_002872	11:5233207|5267612
circRNA_004440	5:50283218|50283984	circRNA_016111	4:131739473|131748512	circRNA_008672	12:40666195|40679789	circRNA_004384	4:137324329|137358737
circRNA_014379	3:8493981|8494747	circRNA_013190	5:86459917|86468959	circRNA_009899	8:134701991|134715585	circRNA_005175	9:129689965|129724374
circRNA_011401	15:59538969|59539737	circRNA_020865	2:30367889|30376931	circRNA_004495	5:92505001|92518596	circRNA_017678	15:97022874|97057285
circRNA_012975	2:142117527|142118295	circRNA_017099	4:36908818|36917862	circRNA_020044	14:96079948|96093544	circRNA_016598	X:85919732|85954145
circRNA_016829	13:202631408|202632177	circRNA_011473	16:25734595|25743640	circRNA_002409	X:14455714|14469311	circRNA_001808	6:62396478|62430900
circRNA_014121	1:154223045|154223816	circRNA_007731	5:26855478|26864524	circRNA_007603	3:119574324|119587923	circRNA_014211	13:91226436|91260862
circRNA_015619	14:59561150|59561921	circRNA_001852	6:92169737|92178784	circRNA_014246	14:49144364|49157965	circRNA_010991	1:127661208|127695635
circRNA_006302	6:127528135|127528907	circRNA_000147	1:145429099|145438147	circRNA_012969	2:125058624|125072226	circRNA_017799	3:74727498|74761931
circRNA_010483	15:93489036|93489809	circRNA_006435	8:143297969|143307020	circRNA_003808	18:9569859|9583462	circRNA_009667	6:91612729|91647168
circRNA_000965	15:89765459|89766233	circRNA_008226	GL896423.1:14083|23135	circRNA_019197	8:3343742|3357351	circRNA_013889	2:121372041|121406493
circRNA_003704	16:47777338|47778112	circRNA_010358	13:29260098|29269156	circRNA_003792	17:53883431|53897046	circRNA_004688	6:147017881|147052351
circRNA_016655	1:144031456|144032230	circRNA_014871	8:3351703|3360764	circRNA_018162	14:95239724|95253340	circRNA_005656	13:29719703|29754177
circRNA_000429	11:73511911|73512686	circRNA_011328	14:53906577|53915641	circRNA_006675	1:117474208|117487827	circRNA_004309	4:90623622|90658112
circRNA_003012	12:37741360|37742135	circRNA_016234	6:88784847|88793915	circRNA_005650	13:24118679|24132299	circRNA_004128	3:84394642|84429144
circRNA_006316	6:156179349|156180124	circRNA_008770	13:75395663|75404735	circRNA_007674	4:92246087|92259710	circRNA_002769	10:12062635|12097159
circRNA_017257	8:29238207|29238982	circRNA_013775	1:180819278|180828351	circRNA_000389	11:14292148|14305774	circRNA_007260	15:86948961|86983485
circRNA_021087	6:871815|872590	circRNA_018965	3:26984449|26993523	circRNA_016725	10:45923431|45937059	circRNA_016107	4:129302194|129336726
circRNA_007204	14:145557672|145558450	circRNA_012185	1:18724958|18734033	circRNA_019938	1:243103750|243117379	circRNA_010279	10:35254557|35289091
circRNA_015939	2:146232432|146233210	circRNA_000235	1:264148082|264157159	circRNA_020770	15:157060548|157074177	circRNA_006027	2:108122136|108156671
circRNA_002547	1:105950020|105950799	circRNA_014655	14:60727790|60736867	circRNA_020404	1:124861734|124875364	circRNA_005374	X:121785129|121819674
circRNA_007784	6:871815|872594	circRNA_010157	X:126961800|126970879	circRNA_003850	18:42193455|42207089	circRNA_008810	13:117594277|117628835

circRNA_004095	3:60093143|60093923	circRNA_013878	2:26467631|26476710	circRNA_003492	15:18325856|18339494	circRNA_018414	1:48636668|48671226
circRNA_013229	6:37882838|37883618	circRNA_008278	X:122539260|122548340	circRNA_015606	14:43480945|43494583	circRNA_008363	1:127113152|127147715
circRNA_019016	4:17011316|17012096	circRNA_009806	7:117016827|117025907	circRNA_004257	4:39929686|39943326	circRNA_008636	12:13030858|13065423
circRNA_018777	15:51637238|51638019	circRNA_000001	1:2805468|2814551	circRNA_009240	2:45007618|45021260	circRNA_016665	1:183813973|183848552
circRNA_006868	11:19157893|19158675	circRNA_001070	16:54816613|54825696	circRNA_018419	1:62685635|62699277	circRNA_000698	13:190906188|190940782
circRNA_007138	14:61599945|61600727	circRNA_017735	18:15081212|15090297	circRNA_004995	8:118371504|118385149	circRNA_007826	6:67384370|67418987
circRNA_000083	1:106025647|106026431	circRNA_020361	1:2761692|2770778	circRNA_000787	14:60123994|60137643	circRNA_016321	7:116973006|117007628
circRNA_016437	9:22612456|22613240	circRNA_003931	2:80454230|80463317	circRNA_010429	14:31736566|31750216	circRNA_015596	14:21462677|21497303
circRNA_019426	10:45782590|45783374	circRNA_021231	8:124944082|124953169	circRNA_011728	3:114349157|114362807	circRNA_004775	7:62801435|62836071
circRNA_020333	9:115704297|115705081	circRNA_010761	6:128400178|128409268	circRNA_012029	8:143369637|143383288	circRNA_000747	14:21462664|21497303
circRNA_008388	1:146914062|146914847	circRNA_014712	5:99073579|99082674	circRNA_002987	12:23736600|23750256	circRNA_004308	4:88636380|88671024
circRNA_000047	1:48670439|48671226	circRNA_018066	1:14415409|14424505	circRNA_020252	7:32783248|32796907	circRNA_005945	18:18610834|18645486
circRNA_002388	JH118940.1:100050|100837	circRNA_000399	11:21049875|21058972	circRNA_020701	14:98657157|98670820	circRNA_008454	1:242696922|242731574
circRNA_003684	16:31282270|31283057	circRNA_006885	11:26295949|26305046	circRNA_003474	14:143104359|143118023	circRNA_004649	6:113003755|113038410
circRNA_015022	2:30868291|30869078	circRNA_006278	6:83149369|83158468	circRNA_017654	14:142840673|142854338	circRNA_017080	3:102783623|102818283
circRNA_018341	7:130882992|130883781	circRNA_007517	2:142600487|142609586	circRNA_002151	8:134408464|134422131	circRNA_011453	15:146501208|146535869
circRNA_007759	5:70423698|70424489	circRNA_000392	11:18571298|18580399	circRNA_018150	14:33793844|33807512	circRNA_011716	3:102783623|102818293
circRNA_008890	14:33242003|33242794	circRNA_006321	7:12262726|12271827	circRNA_006763	1:245581931|245595602	circRNA_013513	GL892822.1:73952|108628
circRNA_011669	3:29719350|29720141	circRNA_001574	4:45282363|45291466	circRNA_005417	1:37113063|37126736	circRNA_015428	12:16036543|16071225
circRNA_003157	13:84695582|84696374	circRNA_004058	3:16571202|16580305	circRNA_015080	12:20108250|20121928	circRNA_015979	3:60805128|60839819
circRNA_014837	4:109041942|109042735	circRNA_006377	7:129884224|129893327	circRNA_004840	7:106678476|106692160	circRNA_019222	8:90710288|90744992
circRNA_021239	9:34787|35582	circRNA_016966	17:57290017|57299121	circRNA_010750	6:86671271|86684955	circRNA_011141	11:35045323|35080039
circRNA_016528	GL894406.2:12859|13655	circRNA_006359	7:100752533|100761638	circRNA_016151	5:57066828|57080513	circRNA_015419	12:13088210|13122950
circRNA_003777	17:44356053|44356851	circRNA_003638	15:131248910|131258016	circRNA_009118	16:71378365|71392051	circRNA_008399	1:176142565|176177307
circRNA_013476	9:100215682|100216480	circRNA_006723	1:181050309|181059423	circRNA_017527	10:50953078|50966766	circRNA_003204	13:131203137|131237882
circRNA_001427	3:55816207|55817006	circRNA_017639	14:78036663|78045777	circRNA_019127	6:132124985|132138673	circRNA_004137	3:88084866|88119620
circRNA_012549	13:73317888|73318687	circRNA_015599	14:31029980|31039099	circRNA_012976	2:142510447|142524139	circRNA_003273	13:211120471|211155228
circRNA_004062	3:17254499|17255299	circRNA_018690	13:202639457|202648576	circRNA_017528	10:54114522|54128217	circRNA_009150	17:43061013|43095775
circRNA_009782	7:83106599|83107400	circRNA_000167	1:195169049|195178170	circRNA_004884	8:16931177|16944881	circRNA_010773	6:147623208|147657973
circRNA_010834	8:57989763|57990564	circRNA_001359	2:142526258|142535381	circRNA_011741	4:16852370|16866075	circRNA_013240	6:52454181|52488957
circRNA_018991	3:90773974|90774775	circRNA_000489	12:44347013|44356138	circRNA_012187	1:21218218|21231923	circRNA_008371	1:132965230|133000011
circRNA_002375	GL896426.1:14159|14961	circRNA_012281	1:182114095|182123220	circRNA_015076	10:35240929|35254634	circRNA_000341	10:50688302|50723087
circRNA_004407	5:6219482|6220285	circRNA_002608	1:145408743|145417869	circRNA_016089	4:92634592|92648299	circRNA_020946	3:33343644|33378455
circRNA_015018	18:22100888|22101691	circRNA_003112	13:42383173|42392300	circRNA_000111	1:128682226|128695939	circRNA_015729	15:124685329|124720160
circRNA_009261	2:81180825|81181629	circRNA_008555	10:54146645|54155775	circRNA_005521	1:283917882|283931595	circRNA_003241	13:167069887|167104720
circRNA_019480	13:50969785|50970589	circRNA_010650	4:17243204|17252337	circRNA_000738	14:13472389|13486104	circRNA_020990	3:121691230|121726072
circRNA_017174	6:64014022|64014828	circRNA_017624	14:30889462|30898597	circRNA_011976	7:121210817|121224532	circRNA_001498	3:114318121|114352969
circRNA_001378	3:5151251|5152058	circRNA_013509	GL892486.1:13566|22703	circRNA_008085	9:13293149|13306866	circRNA_017848	4:127084424|127119281
circRNA_007267	15:97716153|97716961	circRNA_016892	15:36428546|36437683	circRNA_015137	1:11257426|11271143	circRNA_000149	1:146214623|146249488
circRNA_020933	3:19650867|19651675	circRNA_013597	X:139014173|139023311	circRNA_001402	3:32848231|32861950	circRNA_018790	15:85340631|85375504
circRNA_004417	5:23660897|23661707	circRNA_020335	9:127733875|127743013	circRNA_013334	7:73102328|73116048	circRNA_009643	6:67384093|67418987
circRNA_007142	14:65737468|65738278	circRNA_005790	14:77594174|77603313	circRNA_006894	11:55451765|55465491	circRNA_007919	7:62884149|62919050
circRNA_013174	5:54842500|54843310	circRNA_006221	5:79767220|79776359	circRNA_004412	5:16681220|16694950	circRNA_009444	4:39819040|39853942
circRNA_010305	11:12254847|12255658	circRNA_012258	1:135097123|135106264	circRNA_019263	9:40995704|41009434	circRNA_017886	6:73269305|73304224
circRNA_012544	13:55746337|55747148	circRNA_001721	5:56954996|56964141	circRNA_020254	7:35672296|35686026	circRNA_019963	10:50947369|50982289
circRNA_020214	6:47789462|47790273	circRNA_020972	3:84167983|84177128	circRNA_009232	2:35584886|35598620	circRNA_010417	13:210784706|210819635
circRNA_000630	13:106138563|106139375	circRNA_004664	6:132251968|132261114	circRNA_013276	6:135987041|136000777	circRNA_006638	1:60924835|60959765
circRNA_000646	13:120826360|120827173	circRNA_008113	9:49773373|49782522	circRNA_003333	14:33126223|33139960	circRNA_018996	3:102818114|102853047
circRNA_020853	2:2101494|2102307	circRNA_017710	16:47050901|47060055	circRNA_007214	15:18576118|18589857	circRNA_008918	14:67540897|67575848
circRNA_009151	17:44356053|44356867	circRNA_020020	13:156136719|156145873	circRNA_015579	13:205655759|205669502	circRNA_001973	7:57934610|57969569
circRNA_010081	GL896009.2:11444|12258	circRNA_002242	9:70890418|70899574	circRNA_009270	2:87830162|87843906	circRNA_006336	7:57934614|57969573
circRNA_002071	8:33236820|33237635	circRNA_009651	6:78476142|78485300	circRNA_010682	4:112798128|112811873	circRNA_001336	2:121998264|122033234

circRNA_008553	10:54059518|54060333	circRNA_001025	15:147145354|147154516	circRNA_006984	13:33954373|33968119	circRNA_009772	7:73073215|73108194
circRNA_010892	9:102968346|102969162	circRNA_019258	9:40608131|40617293	circRNA_016449	9:40081423|40095172	circRNA_005840	15:66048762|66083752
circRNA_010969	1:63933084|63933900	circRNA_009932	9:30676326|30685489	circRNA_009262	2:82502736|82516486	circRNA_012568	13:98146479|98181473
circRNA_007646	4:40049197|40050014	circRNA_007020	13:90919603|90928767	circRNA_007819	6:57230731|57244482	circRNA_001904	6:132138607|132173603
circRNA_008127	9:71453163|71453980	circRNA_007188	14:132243465|132252629	circRNA_000669	13:144052007|144065761	circRNA_009147	17:43054927|43089928
circRNA_008504	1:313777345|313778162	circRNA_006162	4:96740134|96749299	circRNA_006011	2:89577917|89591671	circRNA_012043	9:11747252|11782261
circRNA_015705	15:80808069|80808886	circRNA_019626	2:71204151|71213316	circRNA_018859	18:6034982|6048736	circRNA_015839	18:43942640|43977652
circRNA_000437	11:87003246|87004064	circRNA_004087	3:49898276|49907442	circRNA_000194	1:212991537|213005294	circRNA_012260	1:136466617|136501633
circRNA_006538	GL896223.1:1634|2452	circRNA_001452	3:81741315|81750482	circRNA_021116	6:92078907|92092664	circRNA_002298	9:139626975|139661994
circRNA_012798	16:29706506|29707324	circRNA_000847	14:115739339|115748510	circRNA_002626	1:168965413|168979171	circRNA_018635	13:58536677|58571704
circRNA_020949	3:40274004|40274823	circRNA_002703	1:242727958|242737129	circRNA_007532	3:11505775|11519534	circRNA_019783	7:82092|117119
circRNA_013340	7:93708009|93708829	circRNA_012876	18:31040615|31049786	circRNA_020208	6:4477977|4491739	circRNA_016740	11:52440550|52475583
circRNA_017496	1:245197179|245197999	circRNA_010630	3:100584279|100593451	circRNA_001332	2:121307436|121321204	circRNA_019633	2:86203222|86238256
circRNA_011598	2:69998260|69999081	circRNA_010779	7:1978363|1987535	circRNA_002119	8:91929412|91943182	circRNA_008103	9:38455021|38490059
circRNA_000468	12:24242431|24243253	circRNA_006720	1:180819175|180828351	circRNA_004321	4:98051660|98065430	circRNA_004669	6:138053763|138088819
circRNA_001470	3:99569226|99570048	circRNA_004600	6:88187287|88196465	circRNA_020764	15:144242335|144256105	circRNA_016264	6:148440633|148475711
circRNA_012465	12:14494109|14494931	circRNA_005664	13:34853744|34862922	circRNA_003536	15:66248256|66262027	circRNA_001271	2:75754177|75789266
circRNA_007037	13:108385469|108386292	circRNA_016304	7:73456776|73465955	circRNA_014606	1:16169680|16183454	circRNA_011715	3:101033374|101068474
circRNA_014719	6:82991958|82992781	circRNA_005504	1:230999159|231008340	circRNA_020549	12:21479256|21493030	circRNA_019978	12:22069389|22104498
circRNA_003087	13:29029712|29030539	circRNA_018031	GL895413.1:12566|21747	circRNA_017852	4:141816454|141830229	circRNA_005638	12:59331765|59366879
circRNA_004932	8:58262345|58263173	circRNA_004721	7:14279340|14288525	circRNA_005468	1:176738721|176752498	circRNA_017262	8:32603610|32638725
circRNA_021080	5:103774497|103775325	circRNA_020803	17:5330457|5339649	circRNA_007083	13:202634797|202648576	circRNA_014724	6:148405581|148440705
circRNA_020411	1:134774498|134775328	circRNA_008699	13:1119574|1128768	circRNA_009726	7:14657819|14671601	circRNA_019864	GL892946.2:83891|119019
circRNA_007513	2:137903629|137904461	circRNA_011212	13:35728481|35737676	circRNA_012574	13:106138563|106152345	circRNA_000407	11:25529781|25564920
circRNA_012436	11:24972445|24973277	circRNA_002930	11:56283465|56292661	circRNA_021034	4:129817308|129831092	circRNA_006668	1:110525008|110560153
circRNA_001760	5:89608596|89609429	circRNA_004404	5:4775102|4784298	circRNA_007375	17:65762779|65776565	circRNA_011550	18:36480482|36515630
circRNA_002912	11:26258633|26259466	circRNA_019290	9:126337275|126346473	circRNA_016398	8:112170059|112183845	circRNA_006455	9:19264985|19300140
circRNA_004809	7:81476768|81477601	circRNA_003533	15:61883151|61892350	circRNA_002079	8:40321067|40334860	circRNA_003789	17:52006963|52042134
circRNA_005168	9:127867416|127868250	circRNA_007648	4:45547502|45556702	circRNA_018375	9:101231884|101245683	circRNA_019822	8:73375718|73410894
circRNA_019702	4:101886085|101886919	circRNA_015880	2:47677026|47686227	circRNA_013389	8:71472988|71486789	circRNA_005611	12:15557534|15592721
circRNA_003228	13:147590503|147591338	circRNA_011708	3:92373579|92382786	circRNA_007835	6:79324154|79337961	circRNA_009140	17:33077647|33112840
circRNA_013189	5:83949409|83950244	circRNA_000337	10:47097358|47106567	circRNA_005107	9:42960097|42973905	circRNA_020351	X:19257387|19292596
circRNA_018525	1:284448584|284449419	circRNA_003479	14:146739636|146748848	circRNA_006478	9:52663750|52677558	circRNA_019502	13:167176711|167211925
circRNA_012224	1:87083864|87084700	circRNA_008689	12:59357665|59366879	circRNA_005364	X:114101454|114115263	circRNA_013986	1:200299009|200334231
circRNA_013559	MT:2994|3830	circRNA_018158	14:60778901|60788115	circRNA_000703	13:202685143|202698955	circRNA_000414	11:52209155|52244398
circRNA_018026	GL894329.1:51333|52170	circRNA_010920	GL895500.1:2619|11834	circRNA_000232	1:263633167|263646983	circRNA_018519	1:280627976|280663228
circRNA_016827	13:179740362|179741200	circRNA_016081	4:73640942|73650159	circRNA_000611	13:86952848|86966664	circRNA_010121	X:56809380|56844642
circRNA_008079	9:10817291|10818130	circRNA_020287	8:40860107|40869325	circRNA_005164	9:125506887|125520704	circRNA_020520	11:466843|502116
circRNA_017175	6:73334893|73335732	circRNA_006547	JH118963.1:42907|52126	circRNA_011586	2:47993416|48007233	circRNA_018620	13:15514377|15549655
circRNA_021085	6:293425|294267	circRNA_017498	1:248812431|248821652	circRNA_019035	4:73696909|73710726	circRNA_008762	13:58781354|58816633
circRNA_002449	X:138428178|138429023	circRNA_019195	8:1066184|1075406	circRNA_011622	2:105165760|105179579	circRNA_010294	10:60057311|60092594
circRNA_008160	9:120390256|120391101	circRNA_001334	2:121405391|121414614	circRNA_012058	9:40608131|40621954	circRNA_003637	15:128043602|128078889
circRNA_020872	2:51828107|51828952	circRNA_010871	9:36992574|37001797	circRNA_014804	16:53238470|53252293	circRNA_007101	14:21576022|21611312
circRNA_014315	16:50736809|50737656	circRNA_015566	13:160279354|160288577	circRNA_020583	13:38891253|38905076	circRNA_002179	9:10202464|10237775
circRNA_018678	13:158549370|158550217	circRNA_019678	3:98714752|98723978	circRNA_014685	2:98312972|98326796	circRNA_007509	2:120392795|120428110
circRNA_001780	6:4219100|4219948	circRNA_021240	9:7162160|7171386	circRNA_004449	5:61500662|61514489	circRNA_012523	13:35652573|35687894
circRNA_010841	8:82594780|82595629	circRNA_001497	3:114030015|114039242	circRNA_015480	13:20986223|21000051	circRNA_004675	6:140604563|140639893
circRNA_014258	14:82515861|82516710	circRNA_014635	13:12433863|12443091	circRNA_000773	14:50887772|50901603	circRNA_017700	16:24958808|24994149
circRNA_000873	14:145833236|145834086	circRNA_006674	1:114174958|114184187	circRNA_015047	7:104451564|104465395	circRNA_008296	1:17087947|17123292
circRNA_002954	12:8036910|8037760	circRNA_007753	5:61457868|61467100	circRNA_012862	18:9751490|9765325	circRNA_012390	10:59635863|59671222
circRNA_011684	3:58997708|58998559	circRNA_018896	2:15930326|15939558	circRNA_016197	6:18436857|18450692	circRNA_018946	2:139528791|139564154
circRNA_005334	X:51812778|51813630	circRNA_002001	7:73514061|73523294	circRNA_001303	2:90152679|90166516	circRNA_020553	12:27532648|27568015

circRNA_001565	4:41114627|41115480	circRNA_006503	9:125452979|125462212	circRNA_002801	10:33597363|33611200	circRNA_013771	1:103226188|103261556
circRNA_003103	13:35742506|35743359	circRNA_018492	1:210251697|210260930	circRNA_005070	9:19251282|19265120	circRNA_017193	6:113086834|113122204
circRNA_000125	1:134678492|134679346	circRNA_020327	9:99990027|99999261	circRNA_009920	9:19251279|19265120	circRNA_020679	14:48087694|48123076
circRNA_016587	X:48728238|48729093	circRNA_007965	7:120856218|120865459	circRNA_016863	14:83671721|83685562	circRNA_007079	13:190890857|190926244
circRNA_002134	8:120767276|120768132	circRNA_008595	11:21787387|21796628	circRNA_000473	12:27366725|27380569	circRNA_020994	3:122965496|123000888
circRNA_013242	6:64478265|64479121	circRNA_009355	3:46264384|46273625	circRNA_008386	1:145425643|145439488	circRNA_016322	7:116973006|117008421
circRNA_013542	GL895413.1:42635|43491	circRNA_014465	6:18418914|18428157	circRNA_019071	5:54894299|54908150	circRNA_016476	9:78667003|78702418
circRNA_013937	6:142419376|142420233	circRNA_009370	3:72580187|72589431	circRNA_018663	13:132970710|132984562	circRNA_007374	17:59454683|59490101
circRNA_017970	8:139708449|139709307	circRNA_013649	9:101637290|101646535	circRNA_017831	4:55310960|55324815	circRNA_009295	2:122006272|122041690
circRNA_006564	X:49434302|49435164	circRNA_017228	7:69684126|69693371	circRNA_007340	17:6303397|6317255	circRNA_005669	13:42298387|42333823
circRNA_010464	15:15300310|15301173	circRNA_018080	1:101847657|101856904	circRNA_015953	3:16566447|16580305	circRNA_004851	7:119789996|119825439
circRNA_012997	3:25766195|25767058	circRNA_003588	15:101702509|101711757	circRNA_004057	3:16566447|16580308	circRNA_013022	3:73613900|73649349
circRNA_019144	7:5046293|5047156	circRNA_006874	11:21787387|21796637	circRNA_020383	1:60945904|60959765	circRNA_009905	8:141457586|141493042
circRNA_004998	8:120759223|120760088	circRNA_011781	4:96748019|96757270	circRNA_011103	10:37657908|37671770	circRNA_017552	11:68634181|68669641
circRNA_011230	13:75857204|75858069	circRNA_003912	2:50799143|50808396	circRNA_017152	5:88191739|88205602	circRNA_011603	2:78515947|78551415
circRNA_018280	5:6094295|6095160	circRNA_001119	17:49147794|49157057	circRNA_007511	2:124221453|124235318	circRNA_000466	12:23370369|23405847
circRNA_008561	10:63270507|63271373	circRNA_002003	7:86329719|86338985	circRNA_012093	9:139613232|139627098	circRNA_016090	4:93607963|93643448
circRNA_013976	JH118886.1:85067|85933	circRNA_003601	15:111901622|111910889	circRNA_004266	4:43558505|43572379	circRNA_012629	14:31361690|31397196
circRNA_003744	17:9404071|9404941	circRNA_005486	1:204819151|204828419	circRNA_005799	14:108754195|108768069	circRNA_010538	18:9534503|9570018
circRNA_019415	1:283172516|283173386	circRNA_002226	9:44916087|44925357	circRNA_015004	15:61774735|61788609	circRNA_019614	18:60674419|60709940
circRNA_019801	7:89045124|89045994	circRNA_003169	13:90954665|90963935	circRNA_006421	8:90731115|90744992	circRNA_015357	10:57994109|58029635
circRNA_007590	3:106723747|106724618	circRNA_020595	13:67295475|67304745	circRNA_007936	7:91938950|91952828	circRNA_011820	5:56928608|56964141
circRNA_019797	7:64702403|64703275	circRNA_000665	13:142925649|142934920	circRNA_007299	16:18583255|18597138	circRNA_013728	8:20657917|20693450
circRNA_000092	1:118377839|118378712	circRNA_001110	17:43359995|43369268	circRNA_012832	17:19717427|19731311	circRNA_009837	8:39363973|39399517
circRNA_003098	13:34918254|34919128	circRNA_010147	X:105847668|105856941	circRNA_004027	2:140877263|140891153	circRNA_002715	1:251516590|251552157
circRNA_015383	11:22243068|22243942	circRNA_007847	6:99212305|99221579	circRNA_007014	13:84797022|84810916	circRNA_018277	4:137107382|137142949
circRNA_020152	3:113264835|113265711	circRNA_006314	6:150870199|150879474	circRNA_002658	1:200314643|200328539	circRNA_001127	17:57540295|57575864
circRNA_014444	5:33927679|33928557	circRNA_018732	14:60123994|60133269	circRNA_004450	5:63272510|63286407	circRNA_018078	1:86577009|86612583
circRNA_015646	14:83544233|83545111	circRNA_019525	14:42341566|42350841	circRNA_005791	14:78234225|78248123	circRNA_003706	16:47778049|47813627
circRNA_004755	7:37462945|37463824	circRNA_013432	9:1498095|1507371	circRNA_018028	GL894376.2:15367|29269	circRNA_005505	1:233012606|233048185
circRNA_005285	GL896549.1:20035|20915	circRNA_011343	14:77586163|77595440	circRNA_009580	5:83018001|83031907	circRNA_019418	10:17000906|17036486
circRNA_009218	2:8665085|8665965	circRNA_018348	8:50040512|50049790	circRNA_014340	18:10874962|10888869	circRNA_005226	GL893006.1:10357|45943
circRNA_013661	1:242065637|242066517	circRNA_001154	18:10894089|10903368	circRNA_010453	14:117646846|117660757	circRNA_009289	2:119260427|119296023
circRNA_016858	14:71925280|71926160	circRNA_003013	12:38622947|38632226	circRNA_007492	2:97457124|97471036	circRNA_016783	13:35652295|35687894
circRNA_018262	4:51505866|51506747	circRNA_004986	8:103699742|103709021	circRNA_002289	9:129664193|129678106	circRNA_017332	9:57695008|57730623
circRNA_019317	GL895057.2:44013|44894	circRNA_010201	1:124967959|124977239	circRNA_019594	17:720308|734222	circRNA_018985	3:81726931|81762554
circRNA_001921	6:147362290|147363172	circRNA_014050	5:35894045|35903325	circRNA_014126	1:182110736|182124651	circRNA_002344	GL894261.2:55392|91044
circRNA_008462	1:245197116|245197999	circRNA_003183	13:102343369|102352650	circRNA_011862	6:13057542|13071460	circRNA_011180	12:45461922|45497578
circRNA_008932	14:81408044|81408927	circRNA_003866	18:56233382|56242663	circRNA_018389	GL895718.2:7630|21550	circRNA_008469	1:254898556|254934216
circRNA_010736	6:40006360|40007244	circRNA_001415	3:48109613|48118895	circRNA_003430	14:117093628|117107550	circRNA_020083	16:71573274|71608938
circRNA_016192	6:15064374|15065259	circRNA_003024	12:44476053|44485338	circRNA_015954	3:16580139|16594063	circRNA_006557	X:17758636|17794304
circRNA_017331	9:57666601|57667486	circRNA_009484	4:98044696|98053981	circRNA_012085	9:128116996|128130921	circRNA_009710	6:147612968|147648651
circRNA_009860	8:76896146|76897033	circRNA_002676	1:205472022|205481309	circRNA_006692	1:143361257|143375185	circRNA_005581	11:14263348|14299032
circRNA_019832	8:139729579|139730466	circRNA_009862	8:80044192|80053480	circRNA_018377	9:139707694|139721622	circRNA_003610	15:116124302|116159993
circRNA_001586	4:61279291|61280179	circRNA_007241	15:63836845|63846137	circRNA_009940	9:37910194|37924125	circRNA_007165	14:108151041|108186735
circRNA_007680	4:107020560|107021448	circRNA_004223	4:1995100|2004393	circRNA_020890	2:85375458|85389392	circRNA_015454	12:45428281|45463975
circRNA_000225	1:250053841|250054732	circRNA_011288	13:206275400|206284694	circRNA_001006	15:120976533|120990471	circRNA_019280	9:88659895|88695591
circRNA_001389	3:18139502|18140393	circRNA_001137	18:2736819|2746115	circRNA_004184	3:116959659|116973597	circRNA_020668	14:10634894|10670593
circRNA_017158	6:389812|390703	circRNA_004898	8:32701671|32710967	circRNA_010214	1:165270115|165284055	circRNA_006851	10:62803459|62839160
circRNA_017638	14:78014861|78015752	circRNA_014215	13:126801546|126810842	circRNA_020750	15:92702601|92716543	circRNA_016393	8:108959474|108995183
circRNA_010439	14:64137426|64138318	circRNA_006429	8:112417048|112426346	circRNA_014113	1:127627761|127641705	circRNA_018139	13:146850620|146886361
circRNA_006340	7:65184011|65184904	circRNA_012419	11:7430020|7439320	circRNA_002323	GL892541.1:17420|31367	circRNA_013304	7:12262726|12298480

circRNA_016535	GL894817.1:3997|4890	circRNA_021310	GL893818.1:51450|60750	circRNA_007831	6:74096530|74110479	circRNA_002016	7:95430732|95466527
circRNA_002399	MT:2285|3179	circRNA_014850	6:69145548|69154853	circRNA_020099	18:26303145|26317097	circRNA_015889	2:79837584|79873381
circRNA_000099	1:125013097|125013992	circRNA_017388	X:40375709|40385014	circRNA_012806	16:40878799|40892757	circRNA_010378	13:84409983|84445793
circRNA_007047	13:130256872|130257767	circRNA_003551	15:85065639|85074945	circRNA_017502	1:268060776|268074734	circRNA_000286	10:16256296|16292113
circRNA_012511	13:16934387|16935282	circRNA_003958	2:87139525|87148831	circRNA_007584	3:104169248|104183209	circRNA_011481	16:34036278|34072097
circRNA_017007	2:16119305|16120200	circRNA_008071	8:145146940|145156246	circRNA_010037	GL892397.1:109233|123194	circRNA_000845	14:115712680|115748510
circRNA_005515	1:269211207|269212103	circRNA_019696	4:48085300|48094608	circRNA_017885	6:69145548|69159515	circRNA_016060	4:39835278|39871109
circRNA_012017	8:108906979|108907876	circRNA_013608	13:202682827|202692136	circRNA_018501	1:231595599|231609566	circRNA_011227	13:59840070|59875903
circRNA_017307	9:8134806|8135704	circRNA_000774	14:51295877|51305187	circRNA_006206	5:50724811|50738780	circRNA_007988	8:20498411|20534245
circRNA_002095	8:74346358|74347258	circRNA_005551	10:37486613|37495924	circRNA_010815	7:134309016|134322985	circRNA_015583	13:210819581|210855436
circRNA_009433	4:16854916|16855816	circRNA_016215	6:64286222|64295533	circRNA_002998	12:36535677|36549647	circRNA_006613	1:21874055|21909914
circRNA_016702	1:297371340|297372240	circRNA_003441	14:120779066|120788379	circRNA_003612	15:116138978|116152953	circRNA_007732	5:31770471|31806332
circRNA_009155	17:45693163|45694064	circRNA_014847	6:31447413|31456726	circRNA_020798	16:78186357|78200333	circRNA_009342	3:33342594|33378455
circRNA_001467	3:98680367|98681269	circRNA_005073	9:22123762|22133076	circRNA_001978	7:62798589|62812567	circRNA_004227	4:10801067|10836946
circRNA_018870	18:20920611|20921513	circRNA_009201	18:48346421|48355735	circRNA_002634	1:181368995|181382973	circRNA_015391	11:26265782|26301669
circRNA_012305	1:242087550|242088453	circRNA_016042	3:135154829|135164143	circRNA_001232	2:30450151|30464133	circRNA_017179	6:85419249|85455157
circRNA_018899	2:30490999|30491902	circRNA_006700	1:146236293|146245609	circRNA_014172	11:26291064|26305046	circRNA_013013	3:58911870|58947781
circRNA_014373	2:120871938|120872842	circRNA_008267	X:106978782|106988101	circRNA_006383	8:9011621|9025604	circRNA_019662	3:16325774|16361690
circRNA_015384	11:22243068|22243973	circRNA_011727	3:114039950|114049273	circRNA_007699	4:134737116|134751099	circRNA_008676	12:44782366|44818283
circRNA_008485	1:282205950|282206858	circRNA_005488	1:205465636|205474960	circRNA_002642	1:188165875|188179860	circRNA_018599	12:37126733|37162666
circRNA_020223	6:77327659|77328568	circRNA_006374	7:123615110|123624436	circRNA_019950	1:293191126|293205111	circRNA_007382	18:10776134|10812072
circRNA_019322	GL896331.1:1142|2052	circRNA_007468	2:80452934|80462260	circRNA_013617	1:293191126|293205115	circRNA_005326	X:22030699|22066648
circRNA_001381	3:7183714|7184628	circRNA_009062	15:134218261|134227587	circRNA_014494	7:65666367|65680360	circRNA_012720	15:61398533|61434485
circRNA_011975	7:120293509|120294423	circRNA_010692	5:14001255|14010581	circRNA_016958	17:33108518|33122517	circRNA_004005	2:128479853|128515807
circRNA_018558	10:71200227|71201141	circRNA_016384	8:88534800|88544126	circRNA_010574	2:86089976|86103979	circRNA_016793	13:59859476|59895449
circRNA_004602	6:88289412|88290327	circRNA_004166	3:107577150|107586477	circRNA_005630	12:44779271|44793275	circRNA_007968	7:124683310|124719293
circRNA_009605	6:869802|870717	circRNA_017579	12:62406847|62416175	circRNA_019067	5:47553612|47567620	circRNA_017297	8:135417176|135453163
circRNA_020002	13:81442395|81443311	circRNA_021256	9:36999899|37009229	circRNA_007322	16:47942252|47956261	circRNA_000216	1:242750212|242786202
circRNA_004965	8:86174179|86175096	circRNA_004694	6:147058599|147067936	circRNA_014190	13:28294413|28308425	circRNA_008912	14:59632979|59668970
circRNA_003280	13:212947616|212948534	circRNA_020678	14:45650613|45659950	circRNA_005810	14:136289720|136303736	circRNA_006499	9:116106122|116142114
circRNA_009328	3:13640058|13640976	circRNA_002382	JH118465.1:159385|168723	circRNA_012359	10:37117371|37131391	circRNA_019312	GL894284.2:84270|120275
circRNA_019331	JH118940.1:113715|114633	circRNA_006936	12:37420069|37429409	circRNA_007618	3:134697661|134711682	circRNA_004444	5:50595002|50631015
circRNA_011592	2:52058462|52059381	circRNA_006219	5:72090687|72100028	circRNA_006103	3:109890287|109904319	circRNA_002764	1:303998762|304034811
circRNA_008447	1:229343179|229344099	circRNA_008625	11:75129058|75138399	circRNA_011113	10:51413904|51427936	circRNA_002756	1:297336189|297372240
circRNA_009028	15:85065639|85066559	circRNA_017459	1:137635283|137644627	circRNA_008048	8:111948599|111962633	circRNA_020235	6:136710334|136746386
circRNA_005725	13:143902275|143903196	circRNA_011606	2:82633420|82642765	circRNA_017792	3:56414602|56428637	circRNA_019650	2:138829293|138865359
circRNA_015557	13:149428084|149429006	circRNA_008112	9:46890981|46900327	circRNA_005916	17:18920220|18934256	circRNA_008318	1:56060605|56096675
circRNA_017242	7:120164114|120165036	circRNA_005680	13:72888860|72898208	circRNA_007429	2:28863997|28878034	circRNA_006258	6:32551152|32587225
circRNA_004388	4:139855414|139856337	circRNA_000951	15:85065639|85074988	circRNA_001578	4:55272747|55286785	circRNA_010080	GL895718.2:7630|43709
circRNA_008730	13:35008849|35009772	circRNA_012566	13:90984047|90993396	circRNA_000714	13:210781573|210795613	circRNA_013620	1:245490347|245526429
circRNA_019804	7:99254891|99255814	circRNA_020844	18:44415387|44424736	circRNA_020698	14:82393345|82407388	circRNA_016164	5:79946906|79983003
circRNA_011932	7:25228359|25229283	circRNA_008211	GL894772.1:8659|18009	circRNA_012134	GL896346.2:42579|56623	circRNA_000496	12:45475763|45511876
circRNA_001559	4:36062015|36062940	circRNA_014932	4:2233274|2242624	circRNA_018666	13:141951238|141965282	circRNA_015677	15:3755802|3791918
circRNA_003630	15:122703583|122704508	circRNA_017325	9:46271320|46280670	circRNA_010390	13:119794850|119808896	circRNA_019033	4:72410011|72446129
circRNA_017249	7:129341000|129341925	circRNA_002655	1:200290313|200299664	circRNA_011335	14:65209645|65223693	circRNA_018104	10:31473583|31509707
circRNA_011175	12:37690214|37691140	circRNA_014547	9:31820568|31829919	circRNA_000340	10:50688302|50702358	circRNA_016832	13:216386615|216422750
circRNA_015499	13:36921381|36922307	circRNA_018527	1:293108779|293118131	circRNA_001153	18:10795154|10809210	circRNA_014205	13:90659114|90695250
circRNA_021263	9:49218349|49219276	circRNA_003465	14:141169010|141178363	circRNA_020238	6:139700927|139714992	circRNA_011131	11:477553|513694
circRNA_004473	5:77642285|77643213	circRNA_016295	7:59732994|59742347	circRNA_007721	5:13498409|13512479	circRNA_011469	16:23613621|23649775
circRNA_013549	GL896435.1:8471|9399	circRNA_007818	6:57230731|57240085	circRNA_005344	X:70495253|70509324	circRNA_014846	5:106543058|106579236
circRNA_000477	12:37179801|37180730	circRNA_015877	2:44841341|44850697	circRNA_014519	8:30554693|30568764	circRNA_014359	2:50769530|50805720
circRNA_016280	7:38843625|38844554	circRNA_006724	1:181057330|181066687	circRNA_015393	11:26411733|26425805	circRNA_003767	17:36480170|36516369

circRNA_004671	6:138087889|138088819	circRNA_020968	3:76361902|76371260	circRNA_015796	17:43088897|43102970	circRNA_012610	13:211120471|211156675
circRNA_005401	1:9397801|9398731	circRNA_005061	9:12986704|12996064	circRNA_017252	8:16931177|16945250	circRNA_002836	10:55772042|55808258
circRNA_009663	6:88389666|88390596	circRNA_011840	5:88158511|88167872	circRNA_004650	6:113681718|113695798	circRNA_018137	13:144085719|144121936
circRNA_012556	13:81194156|81195086	circRNA_010987	1:124875218|124884580	circRNA_011219	13:43454328|43468409	circRNA_012406	11:477466|513694
circRNA_014326	17:6316325|6317255	circRNA_002160	8:144326770|144336134	circRNA_002788	10:25141041|25155126	circRNA_015048	7:116819453|116855702
circRNA_016242	6:98804369|98805299	circRNA_007262	15:87920068|87929434	circRNA_004703	6:148440633|148454719	circRNA_001408	3:39564463|39600713
circRNA_016997	2:5807006|5807936	circRNA_006038	2:128446839|128456208	circRNA_006230	5:87252274|87266366	circRNA_002615	1:151769349|151805605
circRNA_011967	7:102164942|102165873	circRNA_020450	1:205270709|205280079	circRNA_004071	3:29625387|29639487	circRNA_009778	7:76473147|76509406
circRNA_001685	5:6774342|6775274	circRNA_012334	10:29315|38689	circRNA_009445	4:39859616|39873716	circRNA_004985	8:103675822|103712082
circRNA_004452	5:65447461|65448393	circRNA_014592	X:14292770|14302145	circRNA_006453	9:14187707|14201808	circRNA_009396	3:105838607|105874870
circRNA_015626	14:65213318|65214250	circRNA_000464	12:20310297|20319673	circRNA_000192	1:210246828|210260930	circRNA_017265	8:39536968|39573240
circRNA_019272	9:73250503|73251435	circRNA_021174	7:95007160|95016538	circRNA_019031	4:68353630|68367737	circRNA_018061	X:127299998|127336284
circRNA_014507	7:97414024|97414957	circRNA_010169	1:31493349|31502732	circRNA_016142	5:38567660|38581769	circRNA_001850	6:92090650|92126940
circRNA_017563	12:27168205|27169138	circRNA_007373	17:59454683|59464069	circRNA_001907	6:137394338|137408448	circRNA_002870	11:472454|508754
circRNA_002663	1:202611390|202612325	circRNA_008660	12:27597385|27606771	circRNA_012316	1:266578997|266593107	circRNA_016765	12:44463879|44500183
circRNA_002910	11:25559256|25560191	circRNA_000741	14:16172107|16181494	circRNA_019975	12:7954962|7969072	circRNA_010334	12:34601226|34637547
circRNA_003629	15:122630724|122631659	circRNA_020032	14:44792421|44801809	circRNA_014347	18:49455074|49469189	circRNA_017417	1:12198334|12234659
circRNA_010559	2:12145085|12146020	circRNA_015951	3:10507175|10516566	circRNA_004387	4:139842217|139856337	circRNA_020878	2:60806762|60843100
circRNA_018577	11:22574569|22575504	circRNA_014823	2:141885830|141895222	circRNA_007295	15:157067652|157081773	circRNA_002158	8:143239037|143275381
circRNA_002208	9:30657642|30658578	circRNA_013101	4:72834902|72844296	circRNA_010498	15:130325057|130339180	circRNA_003180	13:98155627|98191973
circRNA_014992	14:30895315|30896251	circRNA_014709	5:29938324|29947722	circRNA_000751	14:30050907|30065031	circRNA_005997	2:82502736|82539084
circRNA_001374	2:157822105|157823042	circRNA_012825	16:81588960|81598359	circRNA_001190	18:49455064|49469189	circRNA_013520	GL893841.1:50026|86375
circRNA_008548	10:53682211|53683148	circRNA_008236	X:14292770|14302172	circRNA_007981	8:8643830|8657955	circRNA_019172	7:73412932|73449286
circRNA_015017	18:12704703|12705640	circRNA_021193	8:40120114|40129519	circRNA_009526	5:23502183|23516308	circRNA_011819	5:56918746|56955103
circRNA_011555	18:49340344|49341282	circRNA_020430	1:181043371|181052777	circRNA_005242	GL894261.2:76917|91044	circRNA_018282	5:23502183|23538552
circRNA_012457	12:745794|746732	circRNA_005649	13:24109347|24118754	circRNA_003658	16:365213|379345	circRNA_004316	4:96740134|96776508
circRNA_011075	1:305174205|305175146	circRNA_012014	8:103811005|103820414	circRNA_004977	8:93220585|93234721	circRNA_020453	1:211709522|211745915
circRNA_000860	14:132329649|132330593	circRNA_020981	3:109919944|109929356	circRNA_020460	1:242153420|242167556	circRNA_014792	14:60101240|60137643
circRNA_007621	4:218062|219006	circRNA_001722	5:61337118|61346531	circRNA_006641	1:71041497|71055642	circRNA_018526	1:287936886|287973294
circRNA_012631	14:32135166|32136110	circRNA_016082	4:73740572|73749987	circRNA_018767	14:140551654|140565800	circRNA_003248	13:187748589|187785003
circRNA_004060	3:16739096|16740041	circRNA_006180	4:121744658|121754075	circRNA_013823	13:119253884|119268032	circRNA_010385	13:102287901|102324338
circRNA_001717	5:51954308|51955255	circRNA_007758	5:70411450|70420868	circRNA_011779	4:90643957|90658112	circRNA_004734	7:24685941|24722398
circRNA_013723	6:146461781|146462728	circRNA_017699	16:23698631|23708050	circRNA_017477	1:186636110|186650267	circRNA_017046	2:140344565|140381030
circRNA_007332	16:78165123|78166071	circRNA_020922	2:142615574|142624996	circRNA_021288	9:125480644|125494801	circRNA_017137	5:22689593|22726068
circRNA_013719	5:23508237|23509186	circRNA_012050	9:22545191|22554614	circRNA_005493	1:209510139|209524297	circRNA_020971	3:82691426|82727943
circRNA_003541	15:67009488|67010438	circRNA_004040	2:148210061|148219487	circRNA_011576	2:30353850|30368009	circRNA_017841	4:98105501|98142047
circRNA_012488	12:37865153|37866104	circRNA_012868	18:20346552|20355980	circRNA_005646	13:20996725|21010885	circRNA_012546	13:55776452|55813021
circRNA_017629	14:54077106|54078058	circRNA_001592	4:73740572|73750002	circRNA_011405	15:62961047|62975208	circRNA_018851	17:43066397|43102970
circRNA_000768	14:41118204|41119157	circRNA_005112	9:44916087|44925517	circRNA_013834	14:31008830|31022994	circRNA_021017	4:73577490|73614069
circRNA_009319	2:153868366|153869319	circRNA_004758	7:39245483|39254915	circRNA_014821	2:128516665|128530836	circRNA_015986	3:74727498|74764088
circRNA_011777	4:74192262|74193215	circRNA_016120	5:470060|479492	circRNA_008433	1:204783996|204798169	circRNA_016834	14:13658285|13694878
circRNA_011161	12:14536599|14537553	circRNA_000055	1:59709107|59718542	circRNA_013465	9:72064217|72078392	circRNA_018618	13:2389104|2425710
circRNA_012572	13:104606592|104607547	circRNA_017343	9:96806488|96815924	circRNA_002240	9:61794758|61808937	circRNA_006962	13:4737528|4774160
circRNA_014120	1:144705007|144705962	circRNA_006430	8:120817823|120827260	circRNA_010227	1:202793118|202807301	circRNA_014056	6:150809988|150846624
circRNA_020727	15:18583901|18584857	circRNA_000555	13:33174778|33184216	circRNA_011296	13:210833657|210847843	circRNA_000576	13:42355650|42392300
circRNA_017286	8:103822499|103823456	circRNA_003712	16:47892129|47901568	circRNA_003124	13:51772131|51786322	circRNA_006629	1:45701230|45737883
circRNA_011930	7:20819678|20820636	circRNA_018479	1:195011671|195021110	circRNA_014325	17:5203741|5217932	circRNA_012375	10:53339997|53376653
circRNA_002455	X:144003929|144004888	circRNA_009771	7:73061859|73071300	circRNA_000563	13:34853744|34867936	circRNA_004345	4:117913001|117949658
circRNA_004213	3:135116468|135117428	circRNA_005118	9:51067117|51076560	circRNA_010041	GL892430.1:44064|58262	circRNA_005499	1:227159382|227196042
circRNA_013815	13:55544176|55545136	circRNA_000187	1:205465515|205474960	circRNA_002692	1:227152127|227166326	circRNA_017782	3:10479905|10516566
circRNA_018076	1:83300056|83301016	circRNA_013068	4:8482700|8492146	circRNA_003811	18:9603361|9617560	circRNA_004125	3:84254791|84291468
circRNA_008692	12:60096868|60097830	circRNA_013198	5:91211194|91220640	circRNA_008616	11:56292568|56306768	circRNA_003164	13:86966559|87003242

circRNA_016132	5:23683162|23684124	circRNA_019582	16:18564948|18574394	circRNA_015965	3:43037182|43051386	circRNA_017595	13:75857804|75894492
circRNA_005275	GL896343.1:11885|12848	circRNA_004648	6:111925403|111934854	circRNA_000811	14:78371743|78385948	circRNA_006468	9:39942278|39978970
circRNA_009910	8:146956379|146957342	circRNA_018051	X:64714690|64724142	circRNA_008615	11:56292560|56306768	circRNA_010181	1:60903252|60939946
circRNA_010273	10:19934535|19935498	circRNA_010312	11:22552052|22561505	circRNA_017361	GL893374.2:642|14850	circRNA_015855	2:3002611|3039309
circRNA_005906	16:68923545|68924509	circRNA_011536	18:10879415|10888869	circRNA_008394	1:161598234|161612444	circRNA_011583	2:45021103|45057809
circRNA_012122	GL894584.1:42047|43011	circRNA_004153	3:102653727|102663182	circRNA_008916	14:64347610|64361820	circRNA_008468	1:254897497|254934216
circRNA_017206	6:145323643|145324607	circRNA_013287	6:148034940|148044395	circRNA_012177	1:2775861|2790071	circRNA_011904	6:122000800|122037550
circRNA_009454	4:45572098|45573063	circRNA_019773	6:141363206|141372662	circRNA_017906	6:125473732|125487944	circRNA_001247	2:52228455|52265225
circRNA_001696	5:23683162|23684128	circRNA_021191	8:33463616|33473072	circRNA_016270	7:14125735|14139948	circRNA_000989	15:115236095|115272874
circRNA_008663	12:36509145|36510111	circRNA_010883	9:70888223|70897682	circRNA_016959	17:36766711|36780924	circRNA_019270	9:54409993|54446776
circRNA_001531	3:133957110|133958077	circRNA_012297	1:218566263|218575722	circRNA_003970	2:91159097|91173312	circRNA_021291	9:133828146|133864933
circRNA_012148	X:12408788|12409755	circRNA_020146	3:75746945|75756405	circRNA_015011	16:65140674|65154889	circRNA_008253	X:51899829|51936618
circRNA_017898	6:88473452|88474419	circRNA_012893	18:60636455|60645918	circRNA_015305	1:282130411|282144628	circRNA_017239	7:116818909|116855702
circRNA_010833	8:49959084|49960052	circRNA_016558	GL896571.2:125511|134975	circRNA_018449	1:136476985|136491204	circRNA_018555	10:58875694|58912493
circRNA_007729	5:24316172|24317142	circRNA_019615	2:3008083|3017547	circRNA_018853	17:44398277|44412496	circRNA_000931	15:71690220|71727032
circRNA_009527	5:23515337|23516308	circRNA_014213	13:106221532|106230999	circRNA_014711	5:87252145|87266366	circRNA_002551	1:110525008|110561827
circRNA_015863	2:8323515|8324486	circRNA_017585	13:28146354|28155821	circRNA_010671	4:73577490|73591712	circRNA_011542	18:14997940|15034759
circRNA_015412	12:5479393|5480365	circRNA_005091	9:31851489|31860957	circRNA_012045	9:11795661|11809883	circRNA_018512	1:256978708|257015532
circRNA_004096	3:60663337|60664311	circRNA_016276	7:18292136|18301606	circRNA_019307	GL893259.1:21819|36041	circRNA_009102	16:40878799|40915626
circRNA_004705	6:151228861|151229835	circRNA_009278	2:98823915|98833387	circRNA_012166	X:112398998|112413221	circRNA_013601	14:58561833|58598669
circRNA_020861	2:26467631|26468605	circRNA_014274	15:28884310|28893785	circRNA_008647	12:18731949|18746173	circRNA_004380	4:135625308|135662148
circRNA_017796	3:61505088|61506064	circRNA_017517	10:30411251|30420726	circRNA_015584	13:210833614|210847843	circRNA_004197	3:120260142|120296994
circRNA_010423	14:28527296|28528274	circRNA_004343	4:116997042|117006520	circRNA_006284	6:92163364|92177594	circRNA_006105	3:113383687|113420547
circRNA_000651	13:131013234|131014214	circRNA_011506	17:3489528|3499007	circRNA_012019	8:109055311|109069549	circRNA_009613	6:16266271|16303134
circRNA_018306	6:47202211|47203192	circRNA_017636	14:71913303|71922782	circRNA_003393	14:79594018|79608261	circRNA_005438	1:110525008|110561873
circRNA_020655	13:202560796|202561777	circRNA_018460	1:161765502|161774983	circRNA_008531	10:36694024|36708276	circRNA_017341	9:88644143|88681009
circRNA_012434	11:23622033|23623015	circRNA_011258	13:132234613|132244096	circRNA_011970	7:105394327|105408581	circRNA_017400	X:105384438|105421313
circRNA_019709	4:127182099|127183082	circRNA_009582	5:84825887|84835371	circRNA_020010	13:117188877|117203135	circRNA_012278	1:181050309|181087185
circRNA_005981	2:59040088|59041072	circRNA_012584	13:132979954|132989440	circRNA_000374	11:3530481|3544747	circRNA_017608	13:142945732|142982616
circRNA_017168	6:46160893|46161877	circRNA_009016	15:66954578|66964066	circRNA_012608	13:210781573|210795839	circRNA_012368	10:47069682|47106567
circRNA_010695	5:23811204|23812189	circRNA_009108	16:49030403|49039896	circRNA_012575	13:114754401|114768669	circRNA_013497	9:129664193|129701100
circRNA_002995	12:34001990|34002976	circRNA_012047	9:13299241|13308739	circRNA_002749	1:283810273|283824543	circRNA_000734	14:10633685|10670593
circRNA_001252	2:59040088|59041075	circRNA_011677	3:45419321|45428821	circRNA_014066	X:142927488|142941758	circRNA_017662	15:53400417|53437329
circRNA_010311	11:22552052|22553039	circRNA_006509	9:136550559|136560060	circRNA_013312	7:36377897|36392170	circRNA_015736	15:133558194|133595116
circRNA_010143	X:90783693|90784682	circRNA_016342	8:10588237|10597739	circRNA_003364	14:60159735|60174009	circRNA_020604	13:84596887|84633809
circRNA_005592	11:22552052|22553042	circRNA_016805	13:91499963|91509466	circRNA_020544	12:15123853|15138127	circRNA_011385	14:142803973|142840902
circRNA_007813	6:48285309|48286299	circRNA_010536	18:6250183|6259688	circRNA_013167	5:49634014|49648291	circRNA_017659	15:35514849|35551787
circRNA_008232	MT:1514|2504	circRNA_014953	GL896320.1:10753|20259	circRNA_018629	13:38819847|38834125	circRNA_012256	1:134599618|134636559
circRNA_008974	14:143525551|143526543	circRNA_014214	13:117698446|117707954	circRNA_010935	X:70818283|70832562	circRNA_011579	2:31756186|31793144
circRNA_020548	12:20429896|20430888	circRNA_007095	14:8945938|8955447	circRNA_009787	7:91912202|91926482	circRNA_019351	X:114935600|114972573
circRNA_010601	2:153884125|153885118	circRNA_009596	5:99032220|99041729	circRNA_004752	7:37118868|37133150	circRNA_013672	12:48418687|48455661
circRNA_014377	2:148139473|148140466	circRNA_017550	11:54008348|54017857	circRNA_013054	3:122035667|122049951	circRNA_005219	GL892553.2:66194|103174
circRNA_000495	12:44885590|44886584	circRNA_018611	12:48641265|48650775	circRNA_014440	4:143299843|143314129	circRNA_018739	14:68175267|68212247
circRNA_011482	16:35579543|35580537	circRNA_020737	15:73509995|73519506	circRNA_018757	14:115744138|115758424	circRNA_002029	7:102785672|102822653
circRNA_017169	6:47421800|47422794	circRNA_006346	7:73102328|73111840	circRNA_006306	6:145356549|145370836	circRNA_010452	14:117614090|117651080
circRNA_010741	6:50628650|50629645	circRNA_001839	6:85027838|85037351	circRNA_008418	1:188443660|188457952	circRNA_001888	6:108165403|108202404
circRNA_015238	1:202522458|202523454	circRNA_003587	15:101683537|101693050	circRNA_009976	9:70877652|70891949	circRNA_013831	13:210782621|210819635
circRNA_015205	1:127112257|127113254	circRNA_003664	16:22548129|22557643	circRNA_014677	2:17353231|17367528	circRNA_000155	1:169095484|169132503
circRNA_018085	1:134119280|134120277	circRNA_017178	6:85419249|85428764	circRNA_007581	3:100750155|100764455	circRNA_011724	3:113383528|113420547
circRNA_012846	17:55870974|55871972	circRNA_020396	1:103206286|103215801	circRNA_015234	1:200276116|200290416	circRNA_006487	9:85842426|85879451
circRNA_018543	10:37117371|37118369	circRNA_000771	14:43945222|43954743	circRNA_018517	1:270602784|270617085	circRNA_013737	9:79907204|79944229
circRNA_009222	2:17104712|17105711	circRNA_006719	1:176806314|176815836	circRNA_013810	13:28822491|28836794	circRNA_001457	3:88082590|88119620

circRNA_007051	13:131374931|131375931	circRNA_001639	4:117913001|117922524	circRNA_000754	14:30058998|30073303	circRNA_003724	16:54820692|54857726
circRNA_011093	10:29324824|29325824	circRNA_004216	3:135341139|135350662	circRNA_006631	1:49609705|49624011	circRNA_020831	18:14695794|14732829
circRNA_016976	18:10902368|10903368	circRNA_000067	1:80356327|80365853	circRNA_012639	14:42125619|42139925	circRNA_014170	11:19712163|19749205
circRNA_014826	3:34715827|34716828	circRNA_003726	16:55475544|55485070	circRNA_000136	1:143460231|143474541	circRNA_019965	10:53393187|53430254
circRNA_020416	1:143292928|143293929	circRNA_015000	15:18576118|18585645	circRNA_007617	3:130629324|130643634	circRNA_021266	9:52649358|52686429
circRNA_002863	10:77000880|77001883	circRNA_001988	7:69620773|69630302	circRNA_002393	MT:1409|15721	circRNA_000200	1:225771871|225808956
circRNA_015341	10:37423768|37424772	circRNA_003549	15:80705053|80714582	circRNA_007247	15:73542910|73557223	circRNA_005120	9:57693529|57730623
circRNA_019970	11:53966891|53967896	circRNA_003373	14:72309798|72319329	circRNA_015423	12:13897339|13911659	circRNA_013115	4:90623622|90660717
circRNA_019876	GL896111.1:9971|10977	circRNA_005793	14:82894668|82904199	circRNA_013512	GL892789.1:80248|94569	circRNA_011971	7:109814818|109851924
circRNA_000914	15:55390302|55391310	circRNA_005266	GL895413.1:33959|43491	circRNA_005957	18:49340344|49354669	circRNA_007470	2:82120327|82157445
circRNA_020819	17:65770875|65771883	circRNA_012118	GL893884.1:31770|41304	circRNA_004944	8:72408807|72423138	circRNA_003323	14:31361690|31398817
circRNA_006351	7:86337976|86338985	circRNA_008741	13:42349619|42359154	circRNA_012613	13:216655489|216669820	circRNA_019103	6:73267093|73304224
circRNA_011179	12:45335759|45336769	circRNA_018468	1:176855013|176864548	circRNA_018052	X:70705510|70719841	circRNA_001818	6:69416233|69453376
circRNA_008192	GL893420.1:5623|6635	circRNA_008280	X:127326747|127336284	circRNA_002155	8:140917583|140931918	circRNA_005201	9:143299432|143336580
circRNA_013720	5:32956398|32957411	circRNA_015830	18:27447277|27456814	circRNA_007002	13:72865581|72879916	circRNA_009568	5:72062877|72100028
circRNA_014834	4:37582966|37583979	circRNA_002047	7:128024097|128033636	circRNA_007995	8:32717281|32731617	circRNA_002482	1:25357026|25394185
circRNA_014849	6:56973895|56974908	circRNA_002224	9:44284359|44293899	circRNA_005133	9:78740877|78755215	circRNA_000836	14:98695887|98733049
circRNA_020539	11:76642102|76643115	circRNA_019895	X:122505346|122514886	circRNA_020212	6:44350220|44364560	circRNA_019790	7:33676048|33713210
circRNA_006259	6:37918847|37919861	circRNA_018884	18:42820720|42830262	circRNA_015241	1:202657120|202671463	circRNA_008847	13:199380000|199417167
circRNA_014415	4:40153546|40154560	circRNA_018683	13:179687719|179697262	circRNA_003196	13:119202413|119216757	circRNA_007905	7:31345217|31382413
circRNA_004553	6:43227952|43228967	circRNA_001853	6:92170081|92179625	circRNA_000679	13:156006479|156020824	circRNA_020007	13:114731451|114768669
circRNA_018657	13:127884001|127885016	circRNA_003321	14:31263526|31273074	circRNA_020564	12:48588061|48602409	circRNA_014700	4:67855860|67893087
circRNA_002287	9:127853331|127854347	circRNA_007150	14:77624031|77633580	circRNA_009709	6:147038000|147052351	circRNA_018537	10:25684845|25722081
circRNA_005484	1:202655424|202656440	circRNA_006687	1:136780445|136789996	circRNA_010853	8:131295327|131309682	circRNA_008789	13:90919603|90956856
circRNA_011307	14:13485087|13486104	circRNA_011012	1:143396359|143405910	circRNA_014506	7:97312228|97326586	circRNA_011806	5:31898374|31935633
circRNA_012816	16:51828982|51829999	circRNA_006000	2:82549217|82558770	circRNA_015057	8:140241026|140255386	circRNA_001943	7:1978363|2015624
circRNA_016213	6:56973891|56974908	circRNA_007569	3:87852194|87861750	circRNA_011425	15:92710907|92725270	circRNA_017587	13:33146950|33184216
circRNA_008757	13:55503062|55504080	circRNA_020814	17:44402940|44412496	circRNA_013636	4:93598992|93613358	circRNA_007313	16:36895597|36932871
circRNA_006333	7:44026778|44027797	circRNA_005312	X:10418106|10427663	circRNA_020502	10:20972708|20987074	circRNA_015740	15:157059180|157096463
circRNA_020815	17:49550949|49551968	circRNA_012576	13:117571688|117581247	circRNA_017773	2:139597065|139611434	circRNA_000222	1:249459092|249496380
circRNA_001149	18:10157853|10158873	circRNA_001752	5:79973442|79983003	circRNA_001188	18:44782112|44796486	circRNA_016894	15:54527316|54564609
circRNA_012277	1:180736416|180737438	circRNA_018573	11:18580300|18589861	circRNA_011431	15:105469817|105484194	circRNA_011110	10:47056522|47093826
circRNA_018364	9:8237835|8238857	circRNA_019893	X:116236283|116245845	circRNA_002045	7:124977818|124992196	circRNA_003209	13:142562710|142600017
circRNA_018124	13:28753936|28754959	circRNA_018758	14:116560364|116569928	circRNA_010502	16:27077927|27092307	circRNA_019008	3:121691230|121728555
circRNA_018824	16:29596103|29597127	circRNA_020973	3:90658682|90668246	circRNA_013570	X:30975313|30989693	circRNA_012671	14:97469111|97506440
circRNA_008683	12:48674648|48675673	circRNA_015922	2:128795701|128805266	circRNA_016399	8:117196718|117211108	circRNA_012343	10:17019078|17056412
circRNA_009259	2:80461235|80462260	circRNA_012128	GL895413.1:33959|43526	circRNA_008322	1:59728051|59742442	circRNA_016134	5:31785851|31823209
circRNA_002247	9:72762799|72763825	circRNA_019507	13:202622610|202632177	circRNA_007281	15:121417853|121432247	circRNA_002258	9:82272680|82310044
circRNA_002815	10:37631619|37632645	circRNA_003652	15:147691480|147701049	circRNA_002093	8:71582612|71597007	circRNA_002332	GL893394.1:17824|55189
circRNA_012912	2:30417767|30418794	circRNA_006385	8:9816450|9826019	circRNA_002944	11:84950637|84965034	circRNA_006389	8:29273357|29310723
circRNA_019052	4:129543453|129544482	circRNA_014650	13:210654025|210663594	circRNA_020060	15:85107065|85121462	circRNA_019261	9:40767096|40804462
circRNA_001258	2:60037167|60038197	circRNA_000791	14:61623825|61633400	circRNA_004831	7:99226381|99240779	circRNA_003110	13:42298387|42335756
circRNA_017078	3:92462859|92463890	circRNA_012771	15:120884639|120894217	circRNA_011181	12:45497472|45511876	circRNA_010001	9:107575529|107612903
circRNA_019293	9:127864210|127865241	circRNA_016775	13:14126215|14135795	circRNA_003738	17:1345076|1359481	circRNA_000062	1:63702370|63739758
circRNA_006356	7:97854902|97855934	circRNA_007282	15:122904851|122914434	circRNA_007314	16:36914607|36929016	circRNA_019853	9:116104719|116142114
circRNA_017830	4:51211139|51212171	circRNA_000745	14:16177975|16187559	circRNA_007670	4:88593582|88607994	circRNA_013291	6:150842053|150879474
circRNA_020787	16:37504640|37505672	circRNA_020058	15:80714358|80723942	circRNA_003692	16:37500291|37514704	circRNA_015126	3:72556230|72593654
circRNA_021073	5:88166840|88167872	circRNA_009271	2:87962568|87972159	circRNA_007638	4:37688694|37703107	circRNA_016734	11:14837406|14874847
circRNA_002334	GL893684.1:19920|20953	circRNA_014390	3:61088430|61098022	circRNA_013688	13:202682827|202697241	circRNA_018174	14:139582115|139619567
circRNA_007301	16:23404831|23405864	circRNA_018027	GL894351.1:8060|17652	circRNA_009907	8:143380055|143394475	circRNA_001255	2:59237034|59274491
circRNA_020399	1:108448292|108449328	circRNA_010529	17:55226220|55235813	circRNA_015569	13:167103118|167117540	circRNA_008842	13:167174462|167211925
circRNA_008221	GL896286.1:26895|27932	circRNA_013207	5:101608532|101618125	circRNA_005052	9:9411674|9426099	circRNA_000606	13:83142279|83179748

<table>
<tr><td>circRNA_012192</td><td>1:28530068|28531105</td><td>circRNA_014957</td><td>1:32509604|32519201</td><td>circRNA_001233</td><td>2:30477465|30491902</td><td>circRNA_003846</td><td>18:33305187|33342656</td></tr>
<tr><td>circRNA_003412</td><td>14:98553323|98554363</td><td>circRNA_018664</td><td>13:133914431|133924028</td><td>circRNA_000254</td><td>1:280648787|280663228</td><td>circRNA_007980</td><td>8:6756356|6793833</td></tr>
<tr><td>circRNA_010745</td><td>6:69689581|69690621</td><td>circRNA_009208</td><td>18:59551551|59561149</td><td>circRNA_004535</td><td>6:18449174|18463615</td><td>circRNA_019223</td><td>8:90731115|90768604</td></tr>
<tr><td>circRNA_002452</td><td>X:143993560|143994602</td><td>circRNA_018582</td><td>11:26365584|26375183</td><td>circRNA_005590</td><td>11:19712163|19726605</td><td>circRNA_002612</td><td>1:148459942|148497434</td></tr>
<tr><td>circRNA_007158</td><td>14:94814058|94815100</td><td>circRNA_007426</td><td>2:17211909|17221511</td><td>circRNA_000580</td><td>13:42377857|42392300</td><td>circRNA_009522</td><td>5:14231995|14269491</td></tr>
<tr><td>circRNA_003099</td><td>13:34925731|34926774</td><td>circRNA_007167</td><td>14:108758466|108768069</td><td>circRNA_016497</td><td>9:115691139|115705584</td><td>circRNA_002796</td><td>10:30396527|30434042</td></tr>
<tr><td>circRNA_001145</td><td>18:6593916|6594961</td><td>circRNA_006901</td><td>11:86869925|86879529</td><td>circRNA_010192</td><td>1:86215836|86230282</td><td>circRNA_011024</td><td>1:176704621|176742136</td></tr>
<tr><td>circRNA_010094</td><td>JH118963.1:56275|57321</td><td>circRNA_019448</td><td>11:21429243|21438847</td><td>circRNA_008229</td><td>JH118611.1:25240|39687</td><td>circRNA_019294</td><td>9:128129181|128166700</td></tr>
<tr><td>circRNA_008659</td><td>12:27543194|27544241</td><td>circRNA_003189</td><td>13:103789411|103799017</td><td>circRNA_016396</td><td>8:110665254|110679703</td><td>circRNA_008370</td><td>1:132962462|133000011</td></tr>
<tr><td>circRNA_019409</td><td>1:271967864|271968912</td><td>circRNA_016468</td><td>9:58357792|58367398</td><td>circRNA_016406</td><td>8:140267217|140281666</td><td>circRNA_006895</td><td>11:56099855|56137406</td></tr>
<tr><td>circRNA_004891</td><td>8:29309674|29310723</td><td>circRNA_012252</td><td>1:130207561|130217168</td><td>circRNA_001536</td><td>3:135336211|135350662</td><td>circRNA_000620</td><td>13:91211618|91249175</td></tr>
<tr><td>circRNA_009916</td><td>9:9332253|9333302</td><td>circRNA_000832</td><td>14:98657157|98666777</td><td>circRNA_014092</td><td>1:14096258|14110711</td><td>circRNA_017537</td><td>11:3736554|3774119</td></tr>
<tr><td>circRNA_019042</td><td>4:107439574|107440623</td><td>circRNA_019039</td><td>4:89192965|89202585</td><td>circRNA_006992</td><td>13:42377846|42392300</td><td>circRNA_019276</td><td>9:78933668|78971257</td></tr>
<tr><td>circRNA_019752</td><td>6:18909020|18910069</td><td>circRNA_006796</td><td>1:304797554|304807179</td><td>circRNA_007972</td><td>7:128873317|128887774</td><td>circRNA_018812</td><td>15:147145354|147182944</td></tr>
<tr><td>circRNA_004967</td><td>8:87952704|87953756</td><td>circRNA_011811</td><td>5:35948460|35958087</td><td>circRNA_017115</td><td>4:73730788|73745247</td><td>circRNA_009174</td><td>18:10774476|10812072</td></tr>
<tr><td>circRNA_018367</td><td>9:29693288|29694340</td><td>circRNA_013982</td><td>1:101190051|101199679</td><td>circRNA_005560</td><td>10:51173272|51187732</td><td>circRNA_011447</td><td>15:124628985|124666583</td></tr>
<tr><td>circRNA_007846</td><td>6:99170339|99171392</td><td>circRNA_015562</td><td>13:154715370|154725000</td><td>circRNA_012314</td><td>1:264950262|264964723</td><td>circRNA_016731</td><td>11:4032787|4070420</td></tr>
<tr><td>circRNA_010689</td><td>5:4607780|4608833</td><td>circRNA_004433</td><td>5:38472417|38482048</td><td>circRNA_017322</td><td>9:40753526|40767990</td><td>circRNA_014240</td><td>14:37732669|37770325</td></tr>
<tr><td>circRNA_001773</td><td>5:108177093|108178148</td><td>circRNA_007916</td><td>7:57959941|57969573</td><td>circRNA_016923</td><td>16:18596005|18610477</td><td>circRNA_010420</td><td>14:10493793|10531452</td></tr>
<tr><td>circRNA_002616</td><td>1:152023403|152024459</td><td>circRNA_002448</td><td>X:138419390|138429023</td><td>circRNA_010918</td><td>GL894855.1:14720|29194</td><td>circRNA_008898</td><td>14:43179929|43217590</td></tr>
<tr><td>circRNA_012399</td><td>10:77676098|77677154</td><td>circRNA_014611</td><td>1:127661208|127670842</td><td>circRNA_002525</td><td>1:83300056|83314537</td><td>circRNA_004859</td><td>7:125239034|125276702</td></tr>
<tr><td>circRNA_001741</td><td>5:71944819|71945876</td><td>circRNA_007856</td><td>6:112319400|112329035</td><td>circRNA_001972</td><td>7:57914632|57929116</td><td>circRNA_000375</td><td>11:3736449|3774119</td></tr>
<tr><td>circRNA_013147</td><td>5:13377059|13378117</td><td>circRNA_008406</td><td>1:180705543|180715180</td><td>circRNA_005048</td><td>9:8935966|8950452</td><td>circRNA_010643</td><td>3:125885513|125923207</td></tr>
<tr><td>circRNA_003031</td><td>12:45448728|45449787</td><td>circRNA_008872</td><td>14:10435358|10444996</td><td>circRNA_011196</td><td>13:8012521|8027008</td><td>circRNA_010794</td><td>7:62881349|62919050</td></tr>
<tr><td>circRNA_013367</td><td>8:1116774|1117835</td><td>circRNA_005627</td><td>12:39702231|39711870</td><td>circRNA_014645</td><td>13:130358254|130372748</td><td>circRNA_010722</td><td>5:90584373|90622092</td></tr>
<tr><td>circRNA_006709</td><td>1:161192141|161193203</td><td>circRNA_009361</td><td>3:57414714|57424357</td><td>circRNA_014227</td><td>13:167788266|167802761</td><td>circRNA_002441</td><td>X:122510620|122548340</td></tr>
<tr><td>circRNA_008495</td><td>1:287936886|287937948</td><td>circRNA_011399</td><td>15:53790613|53800256</td><td>circRNA_004013</td><td>2:132404785|132419281</td><td>circRNA_014365</td><td>2:91169756|91207479</td></tr>
<tr><td>circRNA_019385</td><td>1:144963274|144964336</td><td>circRNA_020891</td><td>2:85403503|85413146</td><td>circRNA_002907</td><td>11:25426691|25441196</td><td>circRNA_017964</td><td>8:112004918|112042666</td></tr>
<tr><td>circRNA_018632</td><td>13:46230493|46231556</td><td>circRNA_020772</td><td>15:157401884|157411528</td><td>circRNA_006203</td><td>5:49631609|49646121</td><td>circRNA_001739</td><td>5:70693548|70731302</td></tr>
<tr><td>circRNA_019881</td><td>JH118669.1:204745|205808</td><td>circRNA_019724</td><td>5:42174836|42184481</td><td>circRNA_016484</td><td>9:85842426|85856941</td><td>circRNA_011646</td><td>2:148139473|148177235</td></tr>
<tr><td>circRNA_001703</td><td>5:38335076|38336140</td><td>circRNA_004047</td><td>2:157813396|157823042</td><td>circRNA_019714</td><td>5:2254887|2269403</td><td>circRNA_017412</td><td>X:140015694|140053459</td></tr>
<tr><td>circRNA_001254</td><td>2:59237034|59238099</td><td>circRNA_020202</td><td>5:68003279|68012925</td><td>circRNA_003238</td><td>13:166284117|166298634</td><td>circRNA_009114</td><td>16:54819953|54857726</td></tr>
<tr><td>circRNA_004916</td><td>8:43597516|43598582</td><td>circRNA_005815</td><td>14:140519939|140529589</td><td>circRNA_005250</td><td>GL894597.1:33450|47967</td><td>circRNA_004002</td><td>2:121372041|121409820</td></tr>
<tr><td>circRNA_015538</td><td>13:117331417|117332484</td><td>circRNA_006680</td><td>1:132819046|132828697</td><td>circRNA_011508</td><td>17:12515432|12529955</td><td>circRNA_017543</td><td>11:21764322|21802145</td></tr>
<tr><td>circRNA_021050</td><td>5:32065178|32066245</td><td>circRNA_015929</td><td>2:134971674|134981326</td><td>circRNA_020951</td><td>3:42985753|43000276</td><td>circRNA_018311</td><td>6:81196700|81234534</td></tr>
<tr><td>circRNA_021267</td><td>9:53856375|53857442</td><td>circRNA_017267</td><td>8:39804896|39814548</td><td>circRNA_013767</td><td>1:40749926|40764451</td><td>circRNA_012155</td><td>X:66688064|66725904</td></tr>
<tr><td>circRNA_008106</td><td>9:40614116|40615184</td><td>circRNA_014257</td><td>14:77896241|77905894</td><td>circRNA_010893</td><td>9:113288178|113302707</td><td>circRNA_001189</td><td>18:46871196|46909041</td></tr>
<tr><td>circRNA_015132</td><td>8:109636734|109637802</td><td>circRNA_004240</td><td>4:31387169|31396823</td><td>circRNA_017038</td><td>2:106243351|106257881</td><td>circRNA_015921</td><td>2:128486979|128524824</td></tr>
<tr><td>circRNA_018496</td><td>1:224447360|224448429</td><td>circRNA_014087</td><td>9:250376|260033</td><td>circRNA_018275</td><td>4:132208769|132223299</td><td>circRNA_001294</td><td>2:87085725|87123585</td></tr>
<tr><td>circRNA_008886</td><td>14:31405966|31407036</td><td>circRNA_004901</td><td>8:32749718|32759376</td><td>circRNA_014978</td><td>11:19722198|19736732</td><td>circRNA_017308</td><td>9:8159257|8197119</td></tr>
<tr><td>circRNA_007625</td><td>4:16798423|16799495</td><td>circRNA_008988</td><td>15:22575140|22584801</td><td>circRNA_004173</td><td>3:110292158|110306694</td><td>circRNA_002019</td><td>7:97118845|97156735</td></tr>
<tr><td>circRNA_013237</td><td>6:41839122|41840194</td><td>circRNA_012054</td><td>9:29830958|29840619</td><td>circRNA_019376</td><td>1:128898945|128913483</td><td>circRNA_015930</td><td>2:139080932|139118827</td></tr>
<tr><td>circRNA_021103</td><td>6:48445911|48446983</td><td>circRNA_013127</td><td>4:117893543|117903206</td><td>circRNA_020321</td><td>9:54432237|54446776</td><td>circRNA_018510</td><td>1:256829848|256867750</td></tr>
<tr><td>circRNA_005843</td><td>15:67048310|67049383</td><td>circRNA_006069</td><td>3:43037182|43046846</td><td>circRNA_013862</td><td>16:27088302|27102842</td><td>circRNA_006466</td><td>9:36812992|36850896</td></tr>
<tr><td>circRNA_008942</td><td>14:107499623|107500697</td><td>circRNA_009502</td><td>4:134115899|134125567</td><td>circRNA_013807</td><td>13:1123404|1137946</td><td>circRNA_001710</td><td>5:48589782|48627688</td></tr>
<tr><td>circRNA_014328</td><td>17:42861078|42862152</td><td>circRNA_011523</td><td>17:44398277|44407946</td><td>circRNA_001161</td><td>18:15020215|15034759</td><td>circRNA_002073</td><td>8:33317026|33354941</td></tr>
<tr><td>circRNA_020690</td><td>14:68447475|68448550</td><td>circRNA_020301</td><td>8:144581170|144590839</td><td>circRNA_013073</td><td>4:15257895|15272443</td><td>circRNA_000371</td><td>11:250979|288907</td></tr>
<tr><td>circRNA_000014</td><td>1:16603165|16604241</td><td>circRNA_006275</td><td>6:82339265|82348935</td><td>circRNA_009791</td><td>7:92231943|92246494</td><td>circRNA_016350</td><td>8:39330225|39368167</td></tr>
<tr><td>circRNA_002645</td><td>1:195113989|195115065</td><td>circRNA_014483</td><td>6:132108169|132117840</td><td>circRNA_008129</td><td>9:77414305|77428858</td><td>circRNA_011146</td><td>11:70152479|70190434</td></tr>
<tr><td>circRNA_009982</td><td>9:78757286|78758362</td><td>circRNA_004577</td><td>6:78642664|78652337</td><td>circRNA_016809</td><td>13:106755691|106770245</td><td>circRNA_017481</td><td>1:200296262|200334231</td></tr>
<tr><td>circRNA_006265</td><td>6:56191827|56192904</td><td>circRNA_007147</td><td>14:71895250|71904925</td><td>circRNA_008971</td><td>14:141219820|141234375</td><td>circRNA_013820</td><td>13:100697258|100735239</td></tr>
<tr><td>circRNA_013990</td><td>1:276574512|276575589</td><td>circRNA_020962</td><td>3:71982481|71992156</td><td>circRNA_007719</td><td>5:12719651|12734207</td><td>circRNA_017031</td><td>2:87947590|87985584</td></tr>
</table>

circRNA_017975	9:681605|682682	circRNA_018705	14:30926362|30936038	circRNA_015136	1:10921743|10936300	circRNA_000640	13:117567899|117605927
circRNA_009130	17:9436690|9437768	circRNA_007410	18:57721198|57730875	circRNA_004946	8:74346358|74360916	circRNA_000715	13:210781573|210819635
circRNA_007681	4:107670743|107671822	circRNA_014822	2:140357692|140367372	circRNA_001841	6:85446257|85460816	circRNA_010354	13:18138045|18176107
circRNA_010465	15:15497567|15498646	circRNA_006721	1:181031656|181041341	circRNA_000347	10:58751706|58766266	circRNA_002780	10:18149844|18187909
circRNA_019574	15:111936218|111937298	circRNA_021059	5:67971975|67981660	circRNA_007641	4:39655510|39670072	circRNA_000729	14:7176205|7214283
circRNA_002918	11:49580965|49582048	circRNA_005872	15:122394669|122404355	circRNA_013685	13:136696267|136710833	circRNA_007184	14:122832506|122870587
circRNA_006326	7:23948984|23950067	circRNA_012653	14:60119758|60129445	circRNA_018094	1:242073887|242088453	circRNA_019487	13:86952848|86990937
circRNA_006393	8:32694464|32695547	circRNA_018436	1:117679965|117689652	circRNA_007342	17:13115098|13129665	circRNA_009719	6:156145870|156183966
circRNA_017843	4:106633693|106634776	circRNA_016530	GL894466.1:12549|22237	circRNA_006133	4:37554385|37568955	circRNA_004730	7:21551602|21589708
circRNA_019472	13:35109871|35110954	circRNA_013668	11:4038375|4048067	circRNA_011861	6:11926255|11940825	circRNA_013667	10:55649419|55687530
circRNA_019904	1:28725278|28726362	circRNA_012276	1:180703756|180713449	circRNA_015317	1:305142256|305156826	circRNA_008162	9:120426043|120464158
circRNA_001150	18:10581955|10583041	circRNA_006740	1:205261252|205270946	circRNA_005135	9:79123706|79138277	circRNA_006299	6:125633079|125671201
circRNA_004631	6:101028601|101029687	circRNA_008082	9:13249651|13259345	circRNA_002174	9:7122453|7137030	circRNA_004140	3:90790793|90828943
circRNA_011880	6:73364629|73365715	circRNA_004262	4:42104548|42114243	circRNA_016348	8:32353099|32367679	circRNA_012313	1:256943805|256981965
circRNA_011167	12:23727465|23728553	circRNA_005809	14:131878915|131888610	circRNA_007133	14:60093255|60107836	circRNA_016571	X:14042434|14080611
circRNA_015176	1:72066427|72067515	circRNA_007940	7:92415871|92425567	circRNA_014727	7:121217521|121232103	circRNA_010287	10:54023860|54062038
circRNA_002977	12:17084827|17085916	circRNA_011567	2:15933482|15943182	circRNA_002168	8:147991677|148006263	circRNA_021079	5:94418719|94456902
circRNA_018292	5:88183253|88184342	circRNA_013926	5:79656879|79666579	circRNA_000896	15:28808655|28823242	circRNA_005416	1:31389962|31428147
circRNA_008964	14:137734747|137735837	circRNA_015224	1:176836675|176846376	circRNA_003854	18:44310484|44325072	circRNA_006262	6:48351537|48389725
circRNA_015865	2:16479731|16480821	circRNA_013537	GL895092.1:7358|17061	circRNA_007348	17:33077647|33092235	circRNA_016555	GL896504.1:17264|55460
circRNA_006768	1:264195140|264196231	circRNA_011265	13:144076090|144085795	circRNA_007329	16:57092936|57107527	circRNA_001348	2:135775904|135814103
circRNA_007820	6:62370730|62371821	circRNA_006912	12:14551135|14560842	circRNA_014212	13:97300278|97314870	circRNA_011237	13:87134378|87172589
circRNA_003463	14:140345853|140346945	circRNA_004019	2:136702391|136712100	circRNA_014740	9:63604082|63618674	circRNA_003589	15:103623363|103661579
circRNA_005157	9:115778548|115779641	circRNA_016717	10:18140266|18149975	circRNA_011168	12:23727465|23742062	circRNA_005330	X:45299771|45337993
circRNA_009894	8:130190190|130191283	circRNA_013587	X:78087713|78097423	circRNA_018296	5:99080391|99094992	circRNA_000244	1:270602784|270641008
circRNA_014789	13:212111311|212112404	circRNA_017916	6:145869546|145879257	circRNA_016323	7:120182606|120197216	circRNA_002941	11:75120545|75158789
circRNA_015947	3:6041551|6042644	circRNA_006106	3:113967495|113977210	circRNA_012079	9:113946338|113960949	circRNA_003428	14:117069303|117107550
circRNA_013309	7:28018900|28019995	circRNA_013790	1:245565427|245575146	circRNA_009711	6:148062427|148077039	circRNA_004298	4:74151645|74189892
circRNA_004754	7:37136921|37138017	circRNA_010077	GL895215.1:4533|14253	circRNA_017042	2:120408899|120423513	circRNA_020341	GL894502.2:9089|47350
circRNA_019075	5:69418785|69419881	circRNA_014887	9:72025883|72035603	circRNA_017547	11:25540028|25554646	circRNA_001305	2:91159097|91197361
circRNA_013871	18:27283361|27284461	circRNA_019291	9:127749197|127758917	circRNA_007525	2:153874707|153889326	circRNA_020459	1:241244047|241282312
circRNA_001231	2:30149463|30150564	circRNA_020446	1:200644823|200654543	circRNA_008877	14:12692228|12706847	circRNA_012191	1:23213758|23252035
circRNA_004949	8:75572650|75573751	circRNA_004209	3:128263778|128273499	circRNA_011629	2:119198668|119213291	circRNA_002022	7:98966300|99004583
circRNA_010436	14:57738151|57739252	circRNA_000115	1:133338956|133348678	circRNA_005741	13:190926158|190940782	circRNA_004830	7:98966300|99004586
circRNA_009512	4:143304255|143305357	circRNA_009215	2:3029587|3039309	circRNA_013010	3:57772902|57787529	circRNA_000684	13:158525065|158563363
circRNA_002673	1:204797064|204798169	circRNA_015779	16:65096671|65106394	circRNA_011537	18:10888740|10903368	circRNA_013080	4:33422438|33460740
circRNA_017354	9:138816200|138817306	circRNA_015258	1:218540056|218549781	circRNA_002293	9:129718279|129732908	circRNA_017776	2:145419860|145458183
circRNA_003392	14:78520142|78521249	circRNA_011390	15:19253545|19263276	circRNA_003301	14:13680246|13694878	circRNA_018491	1:208292449|208330783
circRNA_004512	6:869802|870909	circRNA_008428	1:200779615|200789350	circRNA_003805	18:6034100|6048736	circRNA_002072	8:33316588|33354941
circRNA_012651	14:58836943|58838050	circRNA_017846	4:116725354|116735096	circRNA_018751	14:112937729|112952367	circRNA_008025	8:77450887|77489259
circRNA_019522	14:23963072|23964180	circRNA_010717	5:86850247|86859993	circRNA_002274	9:102309677|102324317	circRNA_012766	15:116646273|116684653
circRNA_009315	2:146092829|146093938	circRNA_012695	14:135311335|135321083	circRNA_011115	10:53115416|53130056	circRNA_019475	13:37949748|37988133
circRNA_012248	1:125146816|125147925	circRNA_017656	14:150965603|150975351	circRNA_021182	7:124774765|124789407	circRNA_002183	9:11302178|11340580
circRNA_015407	11:74294032|74295142	circRNA_007388	18:18610834|18620583	circRNA_018530	1:306171665|306186312	circRNA_010599	2:145702685|145741087
circRNA_001916	6:145356549|145357660	circRNA_002581	1:132965230|132974980	circRNA_002092	8:69668235|69682886	circRNA_013085	4:39835278|39873716
circRNA_003885	2:16092401|16093512	circRNA_004238	4:20042957|20052708	circRNA_015828	18:26590275|26604927	circRNA_015913	2:119227889|119266346
circRNA_011671	3:39449044|39450156	circRNA_001557	4:33450988|33460740	circRNA_007201	14:141222593|141237251	circRNA_006832	10:46681560|46720028
circRNA_007631	4:31688033|31689146	circRNA_018409	1:40737383|40747135	circRNA_018773	15:18325856|18340515	circRNA_011618	2:93830099|93868582
circRNA_003125	13:54028102|54029216	circRNA_004030	2:142624803|142634560	circRNA_008190	GL893077.1:35756|50416	circRNA_004356	4:127084424|127122933
circRNA_010623	3:76043022|76044136	circRNA_006968	13:16560693|16570452	circRNA_017564	12:27547982|27562642	circRNA_003570	15:86571013|86609530
circRNA_017789	3:43227450|43228565	circRNA_002044	7:124977818|124987581	circRNA_011735	3:125854763|125869425	circRNA_005601	11:56107956|56146474
circRNA_019695	4:42554036|42555151	circRNA_004900	8:32728074|32737837	circRNA_005215	GL892421.2:63000|77664	circRNA_000226	1:254898556|254937077

<table>
<tr><td>circRNA_001015</td><td>15:124719044|124720160</td><td>circRNA_009397</td><td>3:106700866|106710635</td><td>circRNA_011807</td><td>5:34057373|34072037</td><td>circRNA_012175</td><td>X:143966364|144004888</td></tr>
<tr><td>circRNA_008638</td><td>12:13955863|13956979</td><td>circRNA_013869</td><td>17:56998501|57008272</td><td>circRNA_016749</td><td>12:11463660|11478325</td><td>circRNA_019220</td><td>8:85966163|86004688</td></tr>
<tr><td>circRNA_015685</td><td>15:20364898|20366014</td><td>circRNA_000444</td><td>12:5475167|5484941</td><td>circRNA_020728</td><td>15:19216968|19231633</td><td>circRNA_013770</td><td>1:103176719|103215268</td></tr>
<tr><td>circRNA_020597</td><td>13:73048357|73049473</td><td>circRNA_000828</td><td>14:96757362|96767138</td><td>circRNA_012933</td><td>2:67201163|67215829</td><td>circRNA_010114</td><td>X:31558043|31596601</td></tr>
<tr><td>circRNA_017980</td><td>9:19219890|19221007</td><td>circRNA_015293</td><td>1:261821325|261831103</td><td>circRNA_000769</td><td>14:41894075|41908747</td><td>circRNA_012444</td><td>11:56099855|56138417</td></tr>
<tr><td>circRNA_019826</td><td>8:89345260|89346377</td><td>circRNA_016123</td><td>5:4599055|4608833</td><td>circRNA_018100</td><td>1:299882725|299897398</td><td>circRNA_008966</td><td>14:138412943|138451513</td></tr>
<tr><td>circRNA_013265</td><td>6:99789194|99790312</td><td>circRNA_014772</td><td>10:60057311|60067090</td><td>circRNA_019192</td><td>7:120224746|120239421</td><td>circRNA_019182</td><td>7:94511177|94549747</td></tr>
<tr><td>circRNA_000217</td><td>1:243617932|243619051</td><td>circRNA_002000</td><td>7:73258086|73267871</td><td>circRNA_014399</td><td>3:77337843|77352520</td><td>circRNA_008131</td><td>9:78959290|78997862</td></tr>
<tr><td>circRNA_021260</td><td>9:40859458|40860578</td><td>circRNA_008128</td><td>9:72670797|72680582</td><td>circRNA_006751</td><td>1:218647971|218662649</td><td>circRNA_009684</td><td>6:102238879|102277455</td></tr>
<tr><td>circRNA_001401</td><td>3:29971772|29972893</td><td>circRNA_017171</td><td>6:57276795|57286584</td><td>circRNA_018883</td><td>18:42797967|42812647</td><td>circRNA_017969</td><td>8:123840406|123878982</td></tr>
<tr><td>circRNA_007547</td><td>3:41521905|41523026</td><td>circRNA_020531</td><td>11:21071635|21081424</td><td>circRNA_011134</td><td>11:5274155|5288837</td><td>circRNA_008931</td><td>14:81355508|81394088</td></tr>
<tr><td>circRNA_007901</td><td>7:24168721|24169843</td><td>circRNA_003997</td><td>2:119219842|119229632</td><td>circRNA_000379</td><td>11:5274152|5288837</td><td>circRNA_007490</td><td>2:91881042|91919629</td></tr>
<tr><td>circRNA_001673</td><td>5:3457119|3458242</td><td>circRNA_005585</td><td>11:16102041|16111834</td><td>circRNA_017862</td><td>5:70796224|70810909</td><td>circRNA_017087</td><td>3:116947785|116986374</td></tr>
<tr><td>circRNA_008588</td><td>11:15545905|15547028</td><td>circRNA_000909</td><td>15:52179536|52189330</td><td>circRNA_020552</td><td>12:25978620|25993306</td><td>circRNA_007701</td><td>4:136363463|136402068</td></tr>
<tr><td>circRNA_003475</td><td>14:143705043|143706167</td><td>circRNA_006303</td><td>6:127534127|127543923</td><td>circRNA_016429</td><td>9:11757301|11771988</td><td>circRNA_005228</td><td>GL893259.1:18126|56752</td></tr>
<tr><td>circRNA_017094</td><td>4:16723581|16724706</td><td>circRNA_008088</td><td>9:19259967|19269763</td><td>circRNA_005714</td><td>13:132234613|132249301</td><td>circRNA_006475</td><td>9:40761242|40799868</td></tr>
<tr><td>circRNA_021099</td><td>6:37998707|37999832</td><td>circRNA_009992</td><td>9:91540811|91550607</td><td>circRNA_015876</td><td>2:31299699|31314387</td><td>circRNA_020354</td><td>X:35465814|35504448</td></tr>
<tr><td>circRNA_009978</td><td>9:71092540|71093666</td><td>circRNA_003872</td><td>2:449197|458994</td><td>circRNA_003953</td><td>2:86260574|86275268</td><td>circRNA_007466</td><td>2:80139481|80178118</td></tr>
<tr><td>circRNA_017384</td><td>MT:1617|2743</td><td>circRNA_005189</td><td>9:138393537|138403334</td><td>circRNA_008039</td><td>8:103694327|103709021</td><td>circRNA_011271</td><td>13:148316595|148355241</td></tr>
<tr><td>circRNA_009055</td><td>15:122266857|122267984</td><td>circRNA_018692</td><td>13:202689156|202698955</td><td>circRNA_019023</td><td>4:44883063|44897757</td><td>circRNA_020362</td><td>1:2775861|2814551</td></tr>
<tr><td>circRNA_009971</td><td>9:51279143|51280270</td><td>circRNA_017222</td><td>7:57318122|57327922</td><td>circRNA_005772</td><td>14:31384107|31398817</td><td>circRNA_017925</td><td>7:30110338|30149040</td></tr>
<tr><td>circRNA_010703</td><td>5:53268273|53269400</td><td>circRNA_015446</td><td>12:37370893|37380695</td><td>circRNA_009042</td><td>15:97943095|97957809</td><td>circRNA_005540</td><td>10:18101700|18140410</td></tr>
<tr><td>circRNA_003882</td><td>2:9163330|9164458</td><td>circRNA_015894</td><td>2:83908876|83918678</td><td>circRNA_015766</td><td>16:47892129|47906845</td><td>circRNA_019924</td><td>1:134628972|134667697</td></tr>
<tr><td>circRNA_005095</td><td>9:37674853|37675981</td><td>circRNA_008337</td><td>1:87048426|87058229</td><td>circRNA_018458</td><td>1:154299284|154314006</td><td>circRNA_001143</td><td>18:5384923|5423652</td></tr>
<tr><td>circRNA_018498</td><td>1:227165198|227166326</td><td>circRNA_020640</td><td>13:155932248|155942051</td><td>circRNA_007616</td><td>3:130406103|130420826</td><td>circRNA_007730</td><td>5:24800153|24838893</td></tr>
<tr><td>circRNA_005199</td><td>9:141729679|141730808</td><td>circRNA_011760</td><td>4:39632783|39642587</td><td>circRNA_015688</td><td>15:51611391|51626114</td><td>circRNA_004592</td><td>6:85026828|85065591</td></tr>
<tr><td>circRNA_011958</td><td>7:89101045|89102174</td><td>circRNA_002981</td><td>12:23240658|23250463</td><td>circRNA_000252</td><td>1:280482957|280497683</td><td>circRNA_007986</td><td>8:15379042|15417819</td></tr>
<tr><td>circRNA_021350</td><td>X:3964749|3965878</td><td>circRNA_016377</td><td>8:75769301|75779107</td><td>circRNA_009393</td><td>3:104168480|104183209</td><td>circRNA_017292</td><td>8:134340773|134379557</td></tr>
<tr><td>circRNA_000141</td><td>1:144705007|144706137</td><td>circRNA_014051</td><td>6:11749560|11759367</td><td>circRNA_016687</td><td>1:247405365|247420095</td><td>circRNA_013572</td><td>X:35377743|35416528</td></tr>
<tr><td>circRNA_014218</td><td>13:144391256|144392386</td><td>circRNA_012075</td><td>9:101849358|101859166</td><td>circRNA_011016</td><td>1:148277881|148292614</td><td>circRNA_008384</td><td>1:143939294|143978082</td></tr>
<tr><td>circRNA_014320</td><td>16:56974369|56975499</td><td>circRNA_005050</td><td>9:9101193|9111003</td><td>circRNA_013244</td><td>6:69151420|69166153</td><td>circRNA_005731</td><td>13:149631857|149670651</td></tr>
<tr><td>circRNA_019618</td><td>2:17315584|17316714</td><td>circRNA_018625</td><td>13:24361766|24371577</td><td>circRNA_007966</td><td>7:123615110|123629846</td><td>circRNA_012028</td><td>8:141440630|141479430</td></tr>
<tr><td>circRNA_003184</td><td>13:102377698|102378830</td><td>circRNA_013832</td><td>14:21462664|21472477</td><td>circRNA_011910</td><td>6:141370929|141385665</td><td>circRNA_015717</td><td>15:105540482|105579286</td></tr>
<tr><td>circRNA_008534</td><td>10:37404530|37405662</td><td>circRNA_008475</td><td>1:264881379|264891198</td><td>circRNA_010173</td><td>1:37339107|37353844</td><td>circRNA_012937</td><td>2:75754177|75793005</td></tr>
<tr><td>circRNA_013841</td><td>14:120519820|120520952</td><td>circRNA_014603</td><td>X:113348505|113358326</td><td>circRNA_011664</td><td>3:23062363|23077100</td><td>circRNA_019235</td><td>8:140242798|140281666</td></tr>
<tr><td>circRNA_012327</td><td>1:293515410|293516543</td><td>circRNA_007132</td><td>14:59642266|59652089</td><td>circRNA_008655</td><td>12:25978568|25993306</td><td>circRNA_006289</td><td>6:99506005|99544888</td></tr>
<tr><td>circRNA_016922</td><td>16:18596005|18597138</td><td>circRNA_011020</td><td>1:159826499|159836323</td><td>circRNA_014472</td><td>6:78476142|78490884</td><td>circRNA_016006</td><td>3:102273355|102312238</td></tr>
<tr><td>circRNA_007792</td><td>6:16370604|16371738</td><td>circRNA_015949</td><td>3:10470262|10480087</td><td>circRNA_009052</td><td>15:118162219|118176962</td><td>circRNA_000686</td><td>13:167069887|167108777</td></tr>
<tr><td>circRNA_011427</td><td>15:97956675|97957809</td><td>circRNA_006741</td><td>1:208295276|208305105</td><td>circRNA_018373</td><td>9:78874925|78889668</td><td>circRNA_000681</td><td>13:158524462|158563363</td></tr>
<tr><td>circRNA_013225</td><td>6:25895725|25896859</td><td>circRNA_016414</td><td>8:143311067|143320896</td><td>circRNA_010539</td><td>18:14986350|15001094</td><td>circRNA_011609</td><td>2:87176958|87215862</td></tr>
<tr><td>circRNA_004406</td><td>5:5192560|5193695</td><td>circRNA_005984</td><td>2:61866062|61875892</td><td>circRNA_014649</td><td>13:206362190|206376938</td><td>circRNA_003427</td><td>14:116594582|116633507</td></tr>
<tr><td>circRNA_002879</td><td>11:8604783|8605920</td><td>circRNA_006916</td><td>12:16882081|16891912</td><td>circRNA_020178</td><td>4:83680009|83694757</td><td>circRNA_003550</td><td>15:81369920|81408848</td></tr>
<tr><td>circRNA_006809</td><td>10:14306330|14307467</td><td>circRNA_018960</td><td>3:11496127|11505958</td><td>circRNA_000695</td><td>13:170277812|170292562</td><td>circRNA_013925</td><td>5:56916166|56955103</td></tr>
<tr><td>circRNA_010927</td><td>X:11614674|11615811</td><td>circRNA_002443</td><td>X:127132748|127142583</td><td>circRNA_001020</td><td>15:133558194|133572944</td><td>circRNA_003809</td><td>18:9569859|9608802</td></tr>
<tr><td>circRNA_008325</td><td>1:60958627|60959765</td><td>circRNA_019593</td><td>16:80304477|80314312</td><td>circRNA_013341</td><td>7:94511177|94525927</td><td>circRNA_010810</td><td>7:109744938|109783904</td></tr>
<tr><td>circRNA_011652</td><td>2:157845326|157846464</td><td>circRNA_017476</td><td>1:182114095|182123942</td><td>circRNA_009096</td><td>16:36634366|36649117</td><td>circRNA_010337</td><td>12:37228113|37267082</td></tr>
<tr><td>circRNA_010208</td><td>1:134756874|134758013</td><td>circRNA_009126</td><td>16:78211482|78221332</td><td>circRNA_020790</td><td>16:47809258|47824009</td><td>circRNA_011062</td><td>1:270329811|270368801</td></tr>
<tr><td>circRNA_002883</td><td>11:9912569|9913709</td><td>circRNA_001677</td><td>5:3524350|3534201</td><td>circRNA_002414</td><td>X:17765641|17780393</td><td>circRNA_004056</td><td>3:16286906|16325910</td></tr>
<tr><td>circRNA_004994</td><td>8:113070673|113071813</td><td>circRNA_020299</td><td>8:138513823|138523674</td><td>circRNA_005398</td><td>X:144149375|144164127</td><td>circRNA_002735</td><td>1:270329795|270368801</td></tr>
<tr><td>circRNA_007860</td><td>6:125476805|125477945</td><td>circRNA_005024</td><td>8:143175337|143185192</td><td>circRNA_004892</td><td>8:30550155|30564908</td><td>circRNA_014127</td><td>1:188140834|188179860</td></tr>
<tr><td>circRNA_005231</td><td>GL893374.2:3775|4916</td><td>circRNA_003447</td><td>14:122449149|122459005</td><td>circRNA_007996</td><td>8:32878313|32893069</td><td>circRNA_012469</td><td>12:16046492|16085519</td></tr>
<tr><td>circRNA_011216</td><td>13:38021930|38023071</td><td>circRNA_013163</td><td>5:35813835|35823693</td><td>circRNA_014706</td><td>4:135605447|135620204</td><td>circRNA_017255</td><td>8:20703886|20742913</td></tr>
</table>

circRNA_007207	14:145729515|145730657	circRNA_001779	6:2770529|2780391	circRNA_000400	11:21787387|21802145	circRNA_004277	4:55364472|55403504
circRNA_005304	MT:2306|3449	circRNA_019345	X:68698170|68708032	circRNA_011066	1:280487401|280502159	circRNA_012855	17:65846664|65885696
circRNA_013546	GL896100.1:953|2096	circRNA_013562	X:6738743|6748606	circRNA_010332	12:27154978|27169739	circRNA_020168	4:39849335|39888370
circRNA_009764	7:67736164|67737308	circRNA_019868	GL894163.1:13796|23659	circRNA_021128	6:113747874|113762635	circRNA_000449	12:16024692|16063733
circRNA_003552	15:85133152|85134297	circRNA_010499	15:133563077|133572944	circRNA_011199	13:19744143|19758906	circRNA_009178	18:10888740|10927784
circRNA_018541	10:31910594|31911739	circRNA_008782	13:87162721|87172589	circRNA_006925	12:23741928|23756693	circRNA_020348	X:8578423|8617484
circRNA_020647	13:160337459|160338604	circRNA_012619	14:15467893|15477763	circRNA_011613	2:89767470|89782236	circRNA_006494	9:113184559|113223640
circRNA_007263	15:92433859|92435005	circRNA_005612	12:16053861|16063733	circRNA_013812	13:29739409|29754177	circRNA_004007	2:128786909|128826032
circRNA_001028	15:152173932|152175079	circRNA_012696	14:135597237|135607109	circRNA_004079	3:42052106|42066875	circRNA_005768	14:21564778|21603902
circRNA_007090	14:3079943|3081090	circRNA_004394	4:143304255|143314129	circRNA_001584	4:55349876|55364656	circRNA_015792	17:20669421|20708552
circRNA_007335	17:1345076|1346223	circRNA_019440	11:3257618|3267493	circRNA_021293	9:137710304|137725089	circRNA_019063	5:33833674|33872838
circRNA_011985	8:1529169|1530316	circRNA_015303	1:280564252|280574129	circRNA_010665	4:45690143|45704929	circRNA_003870	18:59518595|59557766
circRNA_011721	3:108119511|108120659	circRNA_018148	14:24107383|24117261	circRNA_001644	4:122939000|122953787	circRNA_020759	15:115254429|115293605
circRNA_014173	11:56140966|56142114	circRNA_008440	1:215885411|215895290	circRNA_008903	14:51290393|51305187	circRNA_003338	14:34693433|34732614
circRNA_014660	14:141236102|141237251	circRNA_009277	2:98306659|98316542	circRNA_009376	3:77625137|77639933	circRNA_020303	9:1142823|1182009
circRNA_013223	6:24231624|24232774	circRNA_005800	14:113064543|113074428	circRNA_006545	JH118669.1:191011|205808	circRNA_018518	1:275780524|275819743
circRNA_017859	5:42893267|42894417	circRNA_017994	9:63604082|63613967	circRNA_020617	13:119243439|119258239	circRNA_007402	18:42650214|42689446
circRNA_002599	1:142976243|142977395	circRNA_008101	9:37679653|37689540	circRNA_014524	8:43583780|43598582	circRNA_011185	12:52232056|52271288
circRNA_002008	7:91949364|91950519	circRNA_012478	12:23729226|23739114	circRNA_003895	2:35582601|35597404	circRNA_011501	16:57288506|57327749
circRNA_014194	13:38021930|38023085	circRNA_004618	6:96922152|96932041	circRNA_001829	6:81320444|81335249	circRNA_004092	3:57385109|57424357
circRNA_017950	8:31739628|31740784	circRNA_012056	9:40148558|40158450	circRNA_000859	14:132243465|132258271	circRNA_017035	2:103773335|103812585
circRNA_008435	1:207016864|207018021	circRNA_002075	8:34670670|34680565	circRNA_003815	18:10792104|10806911	circRNA_011580	2:34839877|34879139
circRNA_018862	18:9728802|9729959	circRNA_016583	X:28964683|28974581	circRNA_014767	1:256967158|256981965	circRNA_017085	3:113981573|114020837
circRNA_003877	2:6014021|6015179	circRNA_002458	1:4648182|4658081	circRNA_004584	6:81320444|81335252	circRNA_001959	7:33470627|33509906
circRNA_008991	15:36264244|36265404	circRNA_005786	14:59597135|59607034	circRNA_017026	2:85446695|85461503	circRNA_016379	8:77396214|77435516
circRNA_013378	8:39572078|39573240	circRNA_010129	X:68698170|68708070	circRNA_000560	13:34006058|34020867	circRNA_015009	16:31971561|32010873
circRNA_015398	11:53900017|53901180	circRNA_007005	13:73331002|73340904	circRNA_003522	15:54564541|54579350	circRNA_009737	7:33676048|33715361
circRNA_016566	JH118861.1:198513|199677	circRNA_007746	5:48617786|48627688	circRNA_015656	14:98646675|98661484	circRNA_011953	7:69814148|69853461
circRNA_016181	5:93861788|93862953	circRNA_001947	7:10555431|10565336	circRNA_020913	2:122626987|122641798	circRNA_005508	1:241901877|241941205
circRNA_017983	9:29740807|29741972	circRNA_004176	3:113380479|113390384	circRNA_015331	10:30396527|30411339	circRNA_000870	14:145123173|145162509
circRNA_013212	5:106542022|106543188	circRNA_003759	17:30274908|30284814	circRNA_018250	3:19080276|19095088	circRNA_011152	11:76391099|76430435
circRNA_006658	1:101693272|101694439	circRNA_007961	7:119670239|119680145	circRNA_005733	13:149670465|149685278	circRNA_008646	12:16062482|16101827
circRNA_015556	13:148412255|148413422	circRNA_009819	8:1256857|1266763	circRNA_014455	5:70372892|70387711	circRNA_018697	14:4040395|4079742
circRNA_019516	14:3642153|3643320	circRNA_015079	11:15983222|15993129	circRNA_018258	3:98006243|98021062	circRNA_000561	13:34615712|34655067
circRNA_014336	17:52398129|52399297	circRNA_020534	11:23622033|23631943	circRNA_019248	9:13650403|13665222	circRNA_000562	13:34615712|34655070
circRNA_005153	9:115691139|115692308	circRNA_006252	6:16564214|16574125	circRNA_001701	5:35894045|35908869	circRNA_015397	11:52436220|52475583
circRNA_004494	5:92100111|92101281	circRNA_005666	13:35739632|35749546	circRNA_013326	7:61979807|61994632	circRNA_000233	1:263840380|263879756
circRNA_015324	10:11695025|11696196	circRNA_006307	6:145916377|145926291	circRNA_019890	X:85946186|85961011	circRNA_007519	2:146476770|146516158
circRNA_016547	GL895934.1:1492|2663	circRNA_011276	13:156013911|156023826	circRNA_018816	16:23448740|23463567	circRNA_020646	13:160212018|160251406
circRNA_017070	3:74760760|74761931	circRNA_013914	4:72844211|72854126	circRNA_003627	15:122446655|122461484	circRNA_005779	14:41894075|41933480
circRNA_008096	9:29836166|29837338	circRNA_013357	7:120858510|120868427	circRNA_007892	6:157482052|157496882	circRNA_011489	16:47778049|47817458
circRNA_011992	8:32205992|32207164	circRNA_013078	4:19886763|19896681	circRNA_013099	4:61932160|61946991	circRNA_014000	13:50980036|51019448
circRNA_008252	X:51806302|51807475	circRNA_013387	8:58474545|58484463	circRNA_005837	15:61718934|61733766	circRNA_014255	14:67517948|67557363
circRNA_010412	13:177534237|177535411	circRNA_009743	7:43147749|43157669	circRNA_019834	8:141086866|141101699	circRNA_003830	18:18661914|18701330
circRNA_013642	8:40333684|40334860	circRNA_018574	11:18975972|18985892	circRNA_005366	X:116015532|116030368	circRNA_007192	14:136612215|136651646
circRNA_010233	1:208348828|208350005	circRNA_001183	18:36505709|36515630	circRNA_017346	9:101934847|101949686	circRNA_001789	6:32344546|32383981
circRNA_011934	7:36410905|36412083	circRNA_010448	14:114527991|114537913	circRNA_003728	16:55481570|55496410	circRNA_008497	1:297500311|297539752
circRNA_020703	14:111700151|111701329	circRNA_019685	3:114067813|114077736	circRNA_012538	13:47552389|47567229	circRNA_015361	10:60057311|60096783
circRNA_002719	1:254981227|254982407	circRNA_017284	8:82961738|82971662	circRNA_000261	1:287973103|287987945	circRNA_007660	4:67947307|67986787
circRNA_003152	13:83142279|83143459	circRNA_020075	16:23639847|23649775	circRNA_000307	10:30396527|30411370	circRNA_006144	4:49039859|49079360
circRNA_008667	12:37398861|37400041	circRNA_010870	9:29836166|29846096	circRNA_011717	3:102818114|102832958	circRNA_012796	16:24954648|24994149
circRNA_020014	13:143959226|143960406	circRNA_012579	13:126886356|126896290	circRNA_005274	GL896294.1:1265|16115	circRNA_002519	1:74429977|74469483

circRNA_017043	2:122655951|122657134	circRNA_018742	14:71905993|71915927	circRNA_019046	4:116008617|116023472	circRNA_005747	13:210819581|210859107
circRNA_000310	10:33080414|33081598	circRNA_020261	7:60539545|60549480	circRNA_004527	6:12648433|12663291	circRNA_014097	1:32129558|32169111
circRNA_001372	2:148349328|148350512	circRNA_013714	4:70732636|70742572	circRNA_011225	13:55530278|55545136	circRNA_019681	3:107111748|107151303
circRNA_003623	15:120243210|120244394	circRNA_003874	2:2992758|3002697	circRNA_004952	8:76896142|76911001	circRNA_002592	1:136590855|136630414
circRNA_011359	14:114667671|114668857	circRNA_011686	3:61098540|61108481	circRNA_006411	8:76896146|76911005	circRNA_013793	10:29576396|29615958
circRNA_008552	10:54057934|54059121	circRNA_016256	6:138078878|138088819	circRNA_008934	14:82894668|82909527	circRNA_013978	X:77203534|77243106
circRNA_009383	3:92480216|92481403	circRNA_019005	3:121254917|121264860	circRNA_009449	4:44538833|44553694	circRNA_002717	1:254897497|254937077
circRNA_000818	14:82613081|82614269	circRNA_008996	15:44368123|44378067	circRNA_012607	13:210047704|210062567	circRNA_019007	3:121657951|121697538
circRNA_004571	6:73931072|73932260	circRNA_009368	3:63149385|63159329	circRNA_010266	10:17594928|17609793	circRNA_014252	14:60123994|60163585
circRNA_019762	6:85292782|85293970	circRNA_013625	13:37977080|37987024	circRNA_012061	9:44922267|44937132	circRNA_016464	9:54476924|54516526
circRNA_008801	13:102385990|102387179	circRNA_011299	14:936878|946829	circRNA_019930	1:176374364|176389232	circRNA_006037	2:122079203|122118806
circRNA_002331	GL893171.1:21567|22757	circRNA_005160	9:118423385|118433337	circRNA_002306	9:143476417|143491288	circRNA_005834	15:36556955|36596563
circRNA_016155	5:65025640|65026831	circRNA_021039	4:137173178|137183130	circRNA_017675	15:86948961|86963832	circRNA_013985	1:127637224|127676835
circRNA_001626	4:107477472|107478664	circRNA_009129	17:6306458|6316411	circRNA_018735	14:64137426|64152302	circRNA_000971	15:94327571|94367189
circRNA_016177	5:91479925|91481117	circRNA_016502	9:133498591|133508551	circRNA_002563	1:124801758|124816635	circRNA_013039	3:101033374|101073016
circRNA_020576	13:20961472|20962666	circRNA_000944	15:79290856|79300817	circRNA_005371	X:121770822|121785699	circRNA_005264	GL895238.2:37239|76883
circRNA_010118	X:46489086|46490282	circRNA_012724	15:65950210|65960174	circRNA_015494	13:35728481|35743359	circRNA_006665	1:106924970|106964621
circRNA_012711	15:44376870|44378067	circRNA_007060	13:142931343|142941310	circRNA_003159	13:86459133|86474012	circRNA_011602	2:77001100|77040765
circRNA_002952	12:5476779|5477978	circRNA_013186	5:77633245|77643213	circRNA_002508	1:60917934|60932819	circRNA_009104	16:47310150|47349817
circRNA_020518	10:77263318|77264517	circRNA_016810	13:108091694|108101662	circRNA_016596	X:73915767|73930652	circRNA_004405	5:5041256|5080928
circRNA_008738	13:37986933|37988133	circRNA_020824	18:2770334|2780302	circRNA_008995	15:37944142|37959030	circRNA_013227	6:27301156|27340831
circRNA_014234	14:30886263|30887464	circRNA_001845	6:88344356|88354325	circRNA_011787	4:110119370|110134258	circRNA_002737	1:270414452|270454132
circRNA_011561	2:2833539|2834743	circRNA_008202	GL894027.2:44024|53995	circRNA_015027	2:119213114|119228003	circRNA_018448	1:136271279|136310959
circRNA_006918	12:19868684|19869889	circRNA_016033	3:122014186|122024159	circRNA_018392	JH118921.1:16802|31692	circRNA_016079	4:72772834|72812537
circRNA_006942	12:40678584|40679789	circRNA_018847	17:36439071|36449044	circRNA_014670	16:27222088|27236981	circRNA_010564	2:28405915|28445623
circRNA_020686	14:60172804|60174009	circRNA_009863	8:87965563|87975537	circRNA_018327	7:5287879|5302773	circRNA_017319	9:36062179|36101918
circRNA_007148	14:71902174|71903380	circRNA_010415	13:206274720|206284694	circRNA_009888	8:123878847|123893742	circRNA_020237	6:139675240|139714992
circRNA_010096	JH118990.1:108156|109362	circRNA_012861	18:9578283|9588258	circRNA_018895	2:15928285|15943182	circRNA_000510	12:53698948|53738722
circRNA_014294	15:86637917|86639123	circRNA_013365	7:134013493|134023468	circRNA_003188	13:103784117|103799017	circRNA_010254	1:293285640|293325414
circRNA_003632	15:122869162|122870369	circRNA_012090	9:137431410|137441390	circRNA_011102	10:37131324|37146225	circRNA_016954	17:19691533|19731311
circRNA_011231	13:76733101|76734308	circRNA_015086	14:31888094|31898074	circRNA_016720	10:19767992|19782893	circRNA_019162	7:61582111|61621899
circRNA_018775	15:19268260|19269467	circRNA_001946	7:4587208|4597189	circRNA_013501	9:136564645|136579549	circRNA_013452	9:41177328|41217130
circRNA_007822	6:63745679|63746888	circRNA_009683	6:102230923|102240904	circRNA_021023	4:79081305|79096209	circRNA_010281	10:43878947|43918754
circRNA_017469	1:161265185|161266394	circRNA_007009	13:81528678|81538660	circRNA_003773	17:42008715|42023624	circRNA_001986	7:67547452|67587294
circRNA_007913	7:57608750|57609962	circRNA_007752	5:56918746|56928732	circRNA_010680	4:98128291|98143200	circRNA_013983	1:107795204|107835060
circRNA_001363	2:147049942|147051156	circRNA_006822	10:29350935|29360924	circRNA_016840	14:31729382|31744293	circRNA_006922	12:23370369|23410227
circRNA_007344	17:15481218|15482432	circRNA_019073	5:64073970|64083959	circRNA_005455	1:141486242|141501160	circRNA_006836	10:50688302|50728163
circRNA_012475	12:17864605|17865819	circRNA_003462	14:136612215|136622206	circRNA_003120	13:47722780|47737699	circRNA_015852	18:60606054|60645918
circRNA_010620	3:57677007|57678222	circRNA_007537	3:22555416|22565409	circRNA_012270	1:157816758|157831683	circRNA_009914	9:6833607|6873499
circRNA_018344	8:32181173|32182390	circRNA_009064	15:145090622|145100618	circRNA_018075	1:80332936|80347867	circRNA_012830	17:14843150|14883055
circRNA_000104	1:126578348|126579567	circRNA_009639	6:64377223|64387219	circRNA_010043	GL892486.1:4394|19327	circRNA_016282	7:43121208|43161125
circRNA_003299	14:11695240|11696461	circRNA_018359	8:144757649|144767647	circRNA_018092	1:231247867|231262805	circRNA_012364	10:37797097|37837047
circRNA_004426	5:36617422|36618643	circRNA_000829	14:96757362|96767362	circRNA_009085	16:25177146|25192087	circRNA_018230	2:79596775|79636728
circRNA_007535	3:17185508|17186729	circRNA_005324	X:19233457|19243457	circRNA_005000	8:122227452|122242394	circRNA_012809	16:46572727|46612699
circRNA_012929	2:61773531|61774752	circRNA_011941	7:53603281|53613285	circRNA_019937	1:215848635|215863577	circRNA_005778	14:41753683|41793663
circRNA_011268	13:145892746|145893968	circRNA_017613	13:167807289|167817294	circRNA_013305	7:20297317|20312260	circRNA_013120	4:103198177|103238159
circRNA_002549	1:107104356|107105579	circRNA_019014	3:134701675|134711682	circRNA_011596	2:60840258|60855204	circRNA_007607	3:122008499|122048486
circRNA_003458	14:135611130|135612353	circRNA_008701	13:3082580|3092588	circRNA_001790	6:32369034|32383981	circRNA_014404	3:104155199|104195188
circRNA_018086	1:144913639|144914862	circRNA_011595	2:60028188|60038197	circRNA_000761	14:31362268|31377217	circRNA_002689	1:225771871|225811866
circRNA_019273	9:73284487|73285710	circRNA_012840	17:42867884|42877893	circRNA_015029	3:5922033|5936986	circRNA_015762	16:42904912|42944920
circRNA_006164	4:102928595|102929819	circRNA_003466	14:141227241|141237251	circRNA_014924	16:23583623|23598577	circRNA_004340	4:113501945|113541958
circRNA_001676	5:3524350|3525576	circRNA_017353	9:138383629|138393642	circRNA_010657	4:39835278|39850235	circRNA_006431	8:124913148|124953169

circRNA_006228	5:86798389|86799615	circRNA_008823	13:143042309|143052323	circRNA_010818	8:3345807|3360764	circRNA_008389	1:154299284|154339318
circRNA_006745	1:212196049|212197275	circRNA_015747	16:23582249|23592263	circRNA_002933	11:70164239|70179198	circRNA_019909	1:60892775|60932819
circRNA_014088	9:46809241|46810467	circRNA_018918	2:80558990|80569004	circRNA_013836	14:57987810|58002776	circRNA_008648	12:19466581|19506627
circRNA_006392	8:32345748|32346975	circRNA_006353	7:91890126|91900141	circRNA_021346	JH118872.1:1770|16736	circRNA_002923	11:52733686|52773771
circRNA_019177	7:91925254|91926482	circRNA_019902	1:25396409|25406424	circRNA_013598	13:56459055|56474023	circRNA_016430	9:11761218|11801305
circRNA_013746	12:23307742|23308971	circRNA_015786	16:80472060|80482076	circRNA_017375	GL895293.1:80547|95518	circRNA_001179	18:25511592|25551680
circRNA_017825	4:37097959|37099189	circRNA_016745	11:86717931|86727947	circRNA_011557	18:52592734|52607709	circRNA_016967	17:59012610|59052702
circRNA_019746	6:12122273|12123504	circRNA_012842	17:43051169|43061187	circRNA_020229	6:101204164|101219140	circRNA_007099	14:21492868|21532972
circRNA_012637	14:35979092|35980324	circRNA_001842	6:85450794|85460816	circRNA_020246	7:5453011|5467989	circRNA_010583	2:104498556|104538670
circRNA_017982	9:22403235|22404468	circRNA_002049	7:134694063|134704085	circRNA_020786	16:36683993|36698971	circRNA_010397	13:145683350|145723470
circRNA_002838	10:56116139|56117374	circRNA_010446	14:108816383|108826406	circRNA_013303	7:12247873|12262858	circRNA_015568	13:166273794|166313915
circRNA_005550	10:35106382|35107617	circRNA_018768	14:141212719|141222744	circRNA_015659	14:116331768|116346753	circRNA_016905	15:91070747|91110875
circRNA_011164	12:20847738|20848973	circRNA_001087	17:14563617|14573644	circRNA_019013	3:134248342|134263327	circRNA_002624	1:161725478|161765610
circRNA_013119	4:102166423|102167659	circRNA_005014	8:139785453|139795481	circRNA_013789	1:241901877|241916869	circRNA_016364	8:57375305|57415474
circRNA_001203	2:329766|331003	circRNA_011874	6:64282430|64292459	circRNA_011477	16:27896184|27911180	circRNA_008089	9:19259967|19300140
circRNA_010846	8:93279365|93280602	circRNA_014523	8:32908502|32918532	circRNA_000763	14:32126531|32141530	circRNA_003995	2:119165841|119206026
circRNA_018695	13:210818398|210819635	circRNA_011984	7:134694054|134704085	circRNA_015206	1:127622340|127637341	circRNA_015715	15:103542857|103583049
circRNA_004324	4:98616995|98618234	circRNA_008120	9:52667526|52677558	circRNA_012505	13:2391632|2406634	circRNA_007570	3:88079394|88119620
circRNA_012127	GL895413.1:33959|35198	circRNA_013006	3:48109613|48119645	circRNA_010318	11:75001213|75016218	circRNA_016448	9:40028498|40068725
circRNA_001686	5:12442511|12443752	circRNA_012483	12:37128365|37138400	circRNA_013757	3:45701245|45716254	circRNA_010696	5:31935410|31975642
circRNA_003716	16:51293826|51295067	circRNA_000249	1:278917340|278927376	circRNA_004551	6:41561594|41576604	circRNA_014289	15:83594678|83634913
circRNA_015124	2:59821233|59822475	circRNA_020788	16:44262809|44272845	circRNA_020201	5:62763024|62778037	circRNA_018986	3:83038013|83078248
circRNA_001029	15:152228373|152229616	circRNA_003266	13:210782621|210792658	circRNA_000179	1:202644306|202659321	circRNA_006985	13:35647658|35687894
circRNA_009317	2:148351052|148352296	circRNA_011480	16:29641797|29651840	circRNA_000289	10:17555075|17570093	circRNA_003365	14:63330103|63370343
circRNA_002051	8:1513679|1514924	circRNA_003974	2:93539380|93549427	circRNA_013385	8:58274497|58289515	circRNA_019458	12:16030982|16071225
circRNA_003255	13:202715441|202716686	circRNA_000086	1:115163045|115173093	circRNA_010059	GL893953.2:79067|94086	circRNA_003505	15:22746712|22786965
circRNA_003335	14:34001311|34002557	circRNA_010440	14:68165377|68175427	circRNA_004751	7:36591623|36606643	circRNA_004568	6:73263962|73304224
circRNA_004402	5:4583340|4584587	circRNA_008375	1:134611516|134621568	circRNA_002339	GL893953.2:79064|94086	circRNA_018426	1:87778133|87818404
circRNA_005251	GL894597.1:48506|49753	circRNA_000482	12:37710316|37720372	circRNA_009881	8:109045156|109060178	circRNA_010186	1:74429977|74470252
circRNA_001830	6:81334003|81335252	circRNA_003284	14:936878|946936	circRNA_006224	5:82625769|82640792	circRNA_003129	13:58776339|58816633
circRNA_003286	14:1158578|1159827	circRNA_009159	17:49153503|49163564	circRNA_008620	11:72290089|72305113	circRNA_005808	14:124044677|124084974
circRNA_020557	12:37151269|37152519	circRNA_015252	1:212196049|212206112	circRNA_011224	13:50643382|50658407	circRNA_010624	3:77312222|77352520
circRNA_019277	9:79283908|79285160	circRNA_004065	3:19058493|19068557	circRNA_011204	13:28131575|28146602	circRNA_018459	1:157816758|157857097
circRNA_018958	3:10255278|10256531	circRNA_016937	16:37504640|37514704	circRNA_018825	16:29666100|29681127	circRNA_004505	5:105210066|105250420
circRNA_002006	7:91917856|91919110	circRNA_011186	12:52771100|52781167	circRNA_015284	1:245197179|245212209	circRNA_001171	18:18675366|18715721
circRNA_011331	14:57846855|57848109	circRNA_011638	2:140871498|140881566	circRNA_006657	1:101247404|101262436	circRNA_019861	9:144197766|144238134
circRNA_018703	14:28594322|28595577	circRNA_016605	X:114448830|114458899	circRNA_002284	9:125451231|125466266	circRNA_015163	1:45632972|45673355
circRNA_013544	GL895865.1:39639|40895	circRNA_019184	7:99403176|99413246	circRNA_018659	13:130118894|130133929	circRNA_006175	4:112851286|112891675
circRNA_005718	13:141245887|141247145	circRNA_009072	15:155078849|155088924	circRNA_010544	18:18671313|18686351	circRNA_013405	8:108954786|108995183
circRNA_011919	6:152329597|152330857	circRNA_002269	9:101476729|101486805	circRNA_002555	1:116733953|116748998	circRNA_013136	4:136390669|136431078
circRNA_004491	5:91755963|91757224	circRNA_000320	10:37117371|37127451	circRNA_007401	18:41987764|42002812	circRNA_002993	12:31875147|31915569
circRNA_013316	7:50067167|50068429	circRNA_016552	GL896387.1:21398|31479	circRNA_011766	4:43494812|43509860	circRNA_018619	13:9577093|9617516
circRNA_020995	3:125926174|125927436	circRNA_017740	18:36573605|36583692	circRNA_019028	4:55318260|55333311	circRNA_015172	1:60905557|60945988
circRNA_020588	13:43391092|43392355	circRNA_000542	13:24101792|24111883	circRNA_021224	8:112004918|112019970	circRNA_014096	1:32128667|32169111
circRNA_001938	6:156189695|156190959	circRNA_005634	12:49654708|49664799	circRNA_000553	13:33160394|33175447	circRNA_011272	13:148316595|148357047
circRNA_002639	1:186650215|186651479	circRNA_000856	14:132237197|132247290	circRNA_006694	1:143998940|144013993	circRNA_007239	15:62005102|62045562
circRNA_010854	8:140977483|140978747	circRNA_008328	1:80283905|80293999	circRNA_010503	16:31977938|31992995	circRNA_010026	9:143198081|143238551
circRNA_015372	11:11196732|11197996	circRNA_006764	1:256829848|256839944	circRNA_013782	1:202656404|202671463	circRNA_014057	8:86001857|86042328
circRNA_006683	1:134659825|134661090	circRNA_015385	11:22548061|22558158	circRNA_019009	3:121711013|121726072	circRNA_005136	9:79907204|79947699
circRNA_016529	GL894448.1:11597|12862	circRNA_015598	14:30895315|30905412	circRNA_013024	3:74763811|74778873	circRNA_015733	15:131239708|131280215
circRNA_017004	2:11364507|11365773	circRNA_003528	15:61398533|61408631	circRNA_018834	16:47889036|47904101	circRNA_020495	10:14306330|14346850
circRNA_004235	4:16777417|16778684	circRNA_005277	GL896372.1:6239|16337	circRNA_000666	13:143037257|143052323	circRNA_012033	8:146890664|146931194

circRNA_017203	6:136908153|136909420	circRNA_002892	11:18595342|18605445	circRNA_016139	5:37185163|37200230	circRNA_014528	8:85966163|86006710
circRNA_018969	3:39173709|39174976	circRNA_011471	16:25321505|25331612	circRNA_006006	2:86689997|86705066	circRNA_004167	3:108496197|108536751
circRNA_008790	13:90946479|90947748	circRNA_001971	7:57270687|57280795	circRNA_009875	8:103767633|103782702	circRNA_002882	11:9467314|9507889
circRNA_016747	12:2272008|2273277	circRNA_004745	7:33103456|33113564	circRNA_002920	11:52209155|52224225	circRNA_009543	5:50996616|51037210
circRNA_020401	1:110714914|110716183	circRNA_016581	X:22244241|22254352	circRNA_008442	1:216048759|216063829	circRNA_019128	6:132127016|132167622
circRNA_009519	5:6771300|6772570	circRNA_020530	11:19712163|19722275	circRNA_005092	9:31863848|31878919	circRNA_012215	1:63702370|63742985
circRNA_004950	8:75577793|75579064	circRNA_013414	8:120527591|120537704	circRNA_004805	7:73405929|73421006	circRNA_003450	14:127833432|127874052
circRNA_008831	13:147427561|147428832	circRNA_006153	4:87535228|87545344	circRNA_010379	13:84704237|84719315	circRNA_006520	GL893394.1:14561|55189
circRNA_020582	13:35734591|35735863	circRNA_016672	1:204814021|204824137	circRNA_010302	11:5252528|5267612	circRNA_003521	15:54542107|54582748
circRNA_000137	1:143473268|143474541	circRNA_011656	3:11490443|11500560	circRNA_009339	3:29618701|29633787	circRNA_004933	8:58262345|58302990
circRNA_018696	14:3112936|3114209	circRNA_007552	3:49791512|49801631	circRNA_011169	12:23736596|23751682	circRNA_018651	13:97274219|97314870
circRNA_011275	13:155952678|155953952	circRNA_017052	2:146067464|146077583	circRNA_008602	11:25517563|25532653	circRNA_003354	14:52830738|52871390
circRNA_005489	1:206978880|206980155	circRNA_005482	1:202513334|202523454	circRNA_015157	1:32082085|32097176	circRNA_012199	1:31387491|31428147
circRNA_015133	9:71422895|71424170	circRNA_017890	6:78432080|78442200	circRNA_004275	4:55364472|55379564	circRNA_018643	13:85098937|85139605
circRNA_019515	14:2406287|2407562	circRNA_008694	12:62435246|62445367	circRNA_001975	7:57954480|57969573	circRNA_020434	1:181340021|181380713
circRNA_019221	8:89423118|89424394	circRNA_000228	1:256805155|256815277	circRNA_014929	3:10513862|10528960	circRNA_000737	14:13445402|13486104
circRNA_006676	1:121440741|121442018	circRNA_008753	13:50969982|50980104	circRNA_013026	3:81216721|81231820	circRNA_015363	10:62681670|62722387
circRNA_015870	2:20619672|20620949	circRNA_014012	15:133037228|133047350	circRNA_008474	1:259286768|259301868	circRNA_019526	14:52206598|52247318
circRNA_020268	7:86998410|86999687	circRNA_006840	10:54031496|54041621	circRNA_008742	13:43300658|43315761	circRNA_008707	13:4752268|4792993
circRNA_011868	6:39418245|39419523	circRNA_019858	9:137075851|137085976	circRNA_005904	16:65139785|65154889	circRNA_007632	4:32658673|32699400
circRNA_000949	15:84437803|84439082	circRNA_011730	3:118475492|118485618	circRNA_020623	13:130293484|130308588	circRNA_016504	9:138813342|138854073
circRNA_000997	15:117585297|117586576	circRNA_009061	15:133617126|133627254	circRNA_003066	13:14159901|14175018	circRNA_011725	3:113999359|114040093
circRNA_016254	6:125574790|125576069	circRNA_018908	2:52847733|52857861	circRNA_014454	5:68106969|68122090	circRNA_006136	4:39819040|39859777
circRNA_004521	6:11928924|11930204	circRNA_002308	9:144503744|144513873	circRNA_002462	1:14114964|14130086	circRNA_011931	7:21442338|21483087
circRNA_012542	13:53493999|53495279	circRNA_000596	13:73039342|73049473	circRNA_020950	3:41183098|41198220	circRNA_001887	6:108161644|108202404
circRNA_015541	13:119193445|119194725	circRNA_013332	7:69660094|69670225	circRNA_012821	16:71603991|71619115	circRNA_009959	9:44922267|44963046
circRNA_014024	6:156189678|156190959	circRNA_020482	1:284490232|284500363	circRNA_006213	5:68106961|68122090	circRNA_001749	5:77331534|77372323
circRNA_015648	14:88636329|88637610	circRNA_020976	3:102842916|102853047	circRNA_009679	6:99529757|99544888	circRNA_013184	5:77331517|77372323
circRNA_008767	13:74656936|74658218	circRNA_011748	4:30233514|30243647	circRNA_006964	13:14159901|14175033	circRNA_014770	10:13908855|13949677
circRNA_020590	13:47280277|47281559	circRNA_003506	15:22758111|22768247	circRNA_007702	4:136805197|136820329	circRNA_013773	1:137504490|137545318
circRNA_007795	6:18426873|18428157	circRNA_014114	1:128019978|128030115	circRNA_000766	14:34717481|34732614	circRNA_003814	18:10776134|10816967
circRNA_014302	15:133388618|133389902	circRNA_013319	7:53488006|53498145	circRNA_008691	12:59907022|59922158	circRNA_016045	3:140577049|140617907
circRNA_017732	18:12704703|12705987	circRNA_010019	9:137085313|137095457	circRNA_014739	9:29830958|29846096	circRNA_017383	JH118940.1:34566|75425
circRNA_007156	14:83858791|83860077	circRNA_001047	16:31971561|31981707	circRNA_004000	2:119280884|119296023	circRNA_016675	1:205493987|205534853
circRNA_009604	6:396370|397656	circRNA_007300	16:22458483|22468631	circRNA_006572	X:68882913|68898055	circRNA_016501	9:133467663|133508551
circRNA_003768	17:39462650|39463937	circRNA_002799	10:33047631|33057782	circRNA_001133	17:69010071|69025218	circRNA_013331	7:65666367|65707257
circRNA_006348	7:80406932|80408219	circRNA_012381	10:54048970|54059121	circRNA_012119	GL894386.1:27340|42489	circRNA_020896	2:87802970|87843906
circRNA_016056	4:37567667|37568955	circRNA_007368	17:57066927|57077079	circRNA_013515	GL892920.1:11453|26602	circRNA_020358	X:110131584|110172524
circRNA_018119	12:24050240|24051528	circRNA_003883	2:11355620|11365773	circRNA_009105	16:47362889|47378040	circRNA_012008	8:78476244|78517185
circRNA_005908	16:77826021|77827311	circRNA_006257	6:31808231|31818387	circRNA_011247	13:102280273|102295425	circRNA_011582	2:41825697|41866646
circRNA_009363	3:59005137|59006428	circRNA_007134	14:60097680|60107836	circRNA_006196	5:32056661|32071815	circRNA_012057	9:40532481|40573450
circRNA_005954	18:44795192|44796486	circRNA_011726	3:114039113|114049273	circRNA_008704	13:3122794|3137949	circRNA_008525	10:33278776|33319767
circRNA_020713	14:134309343|134310638	circRNA_001977	7:60967186|60977347	circRNA_014820	2:128486979|128502134	circRNA_000712	13:210778638|210819635
circRNA_010336	12:37180182|37181479	circRNA_001104	17:43030480|43040642	circRNA_004845	7:117038131|117053289	circRNA_005802	14:115716359|115757379
circRNA_001572	4:44812584|44813882	circRNA_016856	14:71889478|71899641	circRNA_018173	14:136733521|136748686	circRNA_011867	6:25100513|25141559
circRNA_012923	2:50802485|50803783	circRNA_019229	8:108985018|108995183	circRNA_014339	18:814124|829291	circRNA_000877	14:146997281|147038331
circRNA_008208	GL894576.1:22441|23740	circRNA_011875	6:64311271|64321438	circRNA_001547	4:15915883|15931051	circRNA_012730	15:81367769|81408848
circRNA_013808	13:15758220|15759519	circRNA_020206	5:101881542|101891709	circRNA_002229	9:46795297|46810467	circRNA_005402	1:15907346|15948427
circRNA_009581	5:83080956|83082256	circRNA_011363	14:115787502|115797670	circRNA_003833	18:18686159|18701330	circRNA_011545	18:18645265|18686351
circRNA_019816	8:33035209|33036509	circRNA_021324	GL894982.1:29571|39739	circRNA_009054	15:120976533|120991704	circRNA_013640	6:92070354|92111445
circRNA_012700	14:139617959|139619260	circRNA_018961	3:11511922|11522092	circRNA_013502	9:137433503|137448674	circRNA_004537	6:25100513|25141613
circRNA_014136	1:226086404|226087705	circRNA_008455	1:242706609|242716780	circRNA_003477	14:145557672|145572844	circRNA_008605	11:25838572|25879679

circRNA_019169	7:69842801|69844102	circRNA_009070	15:153807566|153817738	circRNA_007187	14:124850854|124866027	circRNA_007791	6:16284471|16325581
circRNA_017778	2:146451204|146452506	circRNA_020528	11:19534518|19544691	circRNA_015657	14:110539454|110554627	circRNA_013671	12:38622947|38664057
circRNA_008217	GL895994.1:37368|38671	circRNA_003688	16:36688797|36698971	circRNA_016887	15:18325856|18341032	circRNA_011733	3:121671913|121713024
circRNA_010728	5:110696947|110698250	circRNA_020794	16:53242115|53252293	circRNA_018569	11:11212642|11227818	circRNA_001071	16:54816613|54857726
circRNA_009708	6:146754276|146755580	circRNA_014526	8:71459662|71469841	circRNA_005238	GL893947.1:6948|22125	circRNA_014398	3:77245218|77286335
circRNA_017164	6:26462255|26463559	circRNA_018194	16:51837857|51848039	circRNA_007422	2:12792393|12807575	circRNA_010057	GL893841.1:45245|86375
circRNA_002773	10:16612452|16613757	circRNA_012060	9:40653401|40663587	circRNA_009409	3:121378915|121394097	circRNA_015996	3:77245203|77286335
circRNA_010477	15:73508752|73510057	circRNA_004541	6:32171146|32181333	circRNA_020024	13:203616934|203632117	circRNA_018400	X:139925204|139966347
circRNA_001802	6:50096098|50097404	circRNA_005591	11:19858193|19868382	circRNA_003472	14:143014790|143029976	circRNA_010953	1:15907346|15948494
circRNA_005453	1:136475794|136477100	circRNA_007864	6:125923718|125933908	circRNA_013865	16:57312562|57327749	circRNA_005465	1:154082449|154123607
circRNA_013462	9:63000517|63001823	circRNA_012393	10:62738557|62748747	circRNA_008342	1:101021270|101036464	circRNA_013496	9:128129294|128170463
circRNA_006246	6:1219989|1221297	circRNA_002668	1:202805859|202816050	circRNA_002950	12:1735134|1750329	circRNA_000011	1:15907346|15948518
circRNA_012105	GL893090.2:37521|38829	circRNA_003772	17:42008715|42018906	circRNA_015472	13:1425667|1440862	circRNA_005734	13:154054001|154095196
circRNA_020059	15:85069175|85070483	circRNA_003230	13:148398444|148408637	circRNA_009524	5:16572550|16587746	circRNA_007583	3:104168480|104209684
circRNA_008051	8:122176417|122177726	circRNA_021227	8:117488167|117498361	circRNA_010800	7:73102342|73117539	circRNA_017716	16:77372488|77413708
circRNA_013662	1:267361614|267362923	circRNA_001040	16:24983954|24994149	circRNA_020093	17:55247404|55262601	circRNA_010046	GL892628.1:7171|48395
circRNA_001274	2:77616722|77618032	circRNA_003095	13:33957923|33968119	circRNA_017568	12:37401810|37417012	circRNA_000373	11:472454|513694
circRNA_003473	14:143089269|143090580	circRNA_001109	17:43088897|43099094	circRNA_020801	17:1345076|1360278	circRNA_001952	7:24644089|24685330
circRNA_003143	13:79284414|79285728	circRNA_008161	9:120426043|120436242	circRNA_000453	12:16048530|16063733	circRNA_020632	13:144080695|144121936
circRNA_008233	MT:2447|3761	circRNA_002522	1:80356327|80366530	circRNA_015060	9:52673623|52688827	circRNA_017920	6:148422752|148464020
circRNA_008710	13:9489303|9490617	circRNA_002530	1:86885065|86895269	circRNA_001447	3:76474842|76490049	circRNA_012388	10:56167668|56208948
circRNA_018036	GL896381.1:13171|14485	circRNA_015529	13:90946479|90956685	circRNA_014910	10:63282823|63298033	circRNA_010898	9:128129181|128170463
circRNA_010255	1:299811725|299813040	circRNA_017300	8:139958736|139968942	circRNA_018263	4:55199068|55214278	circRNA_020072	16:3586420|3627702
circRNA_003198	13:120826360|120827676	circRNA_010348	12:63565756|63575963	circRNA_019952	1:297536723|297551935	circRNA_016693	1:257001531|257042814
circRNA_011402	15:61425963|61427279	circRNA_013957	9:9307763|9317971	circRNA_019325	GL896447.1:19752|34966	circRNA_019711	4:137107382|137148667
circRNA_016239	6:92792577|92793893	circRNA_020504	10:29905494|29915703	circRNA_012968	2:125057008|125072226	circRNA_008026	8:77606790|77648079
circRNA_019995	13:37663759|37665075	circRNA_003501	15:19259257|19269467	circRNA_013461	9:52649358|52664576	circRNA_012745	15:101669077|101710376
circRNA_020732	15:63229414|63230730	circRNA_009196	18:44449630|44459840	circRNA_006540	GL896549.1:8294|23514	circRNA_002506	1:60900123|60941431
circRNA_002368	GL896111.1:2464|3782	circRNA_011392	15:28820398|28830613	circRNA_015964	3:43036166|43051386	circRNA_020554	12:27532648|27573979
circRNA_010524	17:36201364|36202682	circRNA_003969	2:90148542|90158760	circRNA_019299	9:141099533|141114754	circRNA_017314	9:14160467|14201808
circRNA_002571	1:127652255|127653574	circRNA_004770	7:57924473|57934691	circRNA_002620	1:154901709|154916931	circRNA_015413	12:11441895|11483250
circRNA_005532	1:304797554|304798876	circRNA_005844	15:67050145|67060363	circRNA_011078	10:2286849|2302077	circRNA_020525	11:11344497|11385864
circRNA_013206	5:99155769|99157091	circRNA_010832	8:49949834|49960052	circRNA_001996	7:73042261|73057496	circRNA_001648	4:129295358|129336726
circRNA_004499	5:94024345|94025668	circRNA_013025	3:76334887|76345105	circRNA_005419	1:44454221|44469456	circRNA_010032	9:148485115|148526509
circRNA_006625	1:35488669|35489992	circRNA_016913	15:116962020|116972239	circRNA_010496	15:117892875|117908111	circRNA_012948	2:86089976|86131375
circRNA_007316	16:38184718|38186041	circRNA_018235	2:85446695|85456918	circRNA_021168	7:86329719|86344966	circRNA_017422	1:18679112|18720511
circRNA_011262	13:140998202|140999525	circRNA_009284	2:108995245|109005470	circRNA_014799	15:53434689|53449943	circRNA_009117	16:57277154|57318578
circRNA_013001	3:40912876|40914200	circRNA_001567	4:42001869|42012095	circRNA_017437	1:83307812|83323066	circRNA_004459	5:70588009|70629435
circRNA_002926	11:53945586|53946911	circRNA_011442	15:121117559|121127785	circRNA_011789	4:112796616|112811873	circRNA_000009	1:13668197|13709627
circRNA_005682	13:72922671|72923996	circRNA_000565	13:34916547|34926774	circRNA_017904	6:119998132|120013390	circRNA_004014	2:134822800|134864238
circRNA_001418	3:49800303|49801631	circRNA_007639	4:39301775|39312003	circRNA_017610	13:167182568|167197828	circRNA_010053	GL893514.2:12087|53528
circRNA_012370	10:47450264|47451592	circRNA_018974	3:46278413|46288641	circRNA_019339	X:46489086|46504346	circRNA_020157	3:122008499|122049951
circRNA_002278	9:102612803|102614132	circRNA_006821	10:29342271|29352504	circRNA_002302	9:142454453|142469718	circRNA_006168	4:106677979|106719435
circRNA_000937	15:73337972|73339303	circRNA_018737	14:66861565|66871801	circRNA_006366	7:119674750|119690020	circRNA_004161	3:106269291|106310785
circRNA_014223	13:149286710|149288041	circRNA_006039	2:132404785|132415023	circRNA_019837	8:141385727|141401000	circRNA_013008	3:55775496|55817006
circRNA_003409	14:94772383|94773715	circRNA_006913	12:14691671|14701912	circRNA_009075	16:3984456|3999731	circRNA_005946	18:18610834|18652345
circRNA_006169	4:106746218|106747551	circRNA_020473	1:268025872|268036113	circRNA_018649	13:91207372|91222648	circRNA_007073	13:155623439|155664955
circRNA_015127	5:70382336|70383669	circRNA_003904	2:43974053|43984295	circRNA_017106	4:46386225|46401504	circRNA_007788	6:16261615|16303134
circRNA_019590	16:51794590|51795923	circRNA_005807	14:124044677|124054928	circRNA_000615	13:87157309|87172589	circRNA_010136	X:70664081|70705622
circRNA_000241	1:270471815|270473150	circRNA_020199	5:54866869|54877125	circRNA_012413	11:4032787|4048067	circRNA_008470	1:255707317|255748862
circRNA_003802	18:3240831|3242166	circRNA_005243	GL894350.2:20200|30458	circRNA_004386	4:139605677|139620959	circRNA_001177	18:25510131|25551680
circRNA_014456	5:73046399|73047734	circRNA_016941	16:47505528|47515788	circRNA_021298	GL892397.1:99226|114511	circRNA_011619	2:93851810|93893360

circRNA_000173	1:201460678|201462014	circRNA_003757	17:30263142|30273404	circRNA_007814	6:48351537|48366827	circRNA_014641	13:98121118|98162682
circRNA_019321	GL896265.2:92611|93947	circRNA_013729	8:93144154|93154416	circRNA_002427	X:70638360|70653651	circRNA_002990	12:27366725|27408291
circRNA_001319	2:108987945|108989283	circRNA_019138	6:148809541|148819803	circRNA_000116	1:134284841|134300133	circRNA_001840	6:85419249|85460816
circRNA_009081	16:23686581|23687919	circRNA_019855	9:127886521|127896783	circRNA_002373	GL896372.1:1040|16337	circRNA_020019	13:149837473|149879040
circRNA_016991	18:41323076|41324414	circRNA_018671	13:148331555|148341820	circRNA_010998	1:134659825|134675125	circRNA_002700	1:241875289|241916869
circRNA_012294	1:212171161|212172500	circRNA_009497	4:122107165|122117431	circRNA_005822	14:147023028|147038331	circRNA_001089	17:14844243|14885851
circRNA_017138	5:28995563|28996902	circRNA_014176	12:16852309|16862575	circRNA_018636	13:61562235|61577543	circRNA_004691	6:147026312|147067936
circRNA_014291	15:85220075|85221415	circRNA_008958	14:130791469|130801736	circRNA_020897	2:88187232|88202545	circRNA_012778	15:133531311|133572944
circRNA_019411	1:281712176|281713517	circRNA_001613	4:98051660|98061929	circRNA_007522	2:148177179|148192512	circRNA_009532	5:37132179|37173822
circRNA_001299	2:87624115|87625457	circRNA_005223	GL892805.1:48659|58930	circRNA_007704	4:139605626|139620959	circRNA_001869	6:99506005|99547687
circRNA_012474	12:17112901|17114245	circRNA_017056	2:153879053|153889326	circRNA_015653	14:95245306|95260651	circRNA_004428	5:37132109|37173822
circRNA_013457	9:46458100|46459444	circRNA_016030	3:121245045|121255319	circRNA_013416	8:123981625|123996974	circRNA_004695	6:147425793|147467514
circRNA_002383	JH118611.1:2203|3548	circRNA_006946	12:45343485|45353762	circRNA_010823	8:12701991|12717344	circRNA_002171	9:639963|681692
circRNA_007362	17:49147794|49149139	circRNA_012784	15:145119846|145130125	circRNA_017959	8:75763752|75779107	circRNA_018267	4:75991837|76033589
circRNA_009145	17:41092440|41093786	circRNA_000253	1:280487401|280497683	circRNA_004702	6:148390283|148405640	circRNA_019478	13:50616643|50658407
circRNA_012865	18:12311411|12312757	circRNA_006576	X:90730226|90740510	circRNA_012330	1:305377281|305392639	circRNA_019597	17:44564987|44606764
circRNA_014881	8:140973217|140974563	circRNA_014193	13:35122058|35132343	circRNA_002765	1:305377277|305392639	circRNA_003173	13:91207372|91249175
circRNA_017795	3:60832497|60833843	circRNA_001855	6:92745097|92755383	circRNA_012220	1:84754696|84770058	circRNA_003554	15:85315348|85357154
circRNA_013176	5:62806332|62807679	circRNA_010838	8:75358318|75368606	circRNA_016126	5:14231995|14247357	circRNA_021064	5:76330931|76372755
circRNA_015790	17:15890788|15892137	circRNA_000342	10:51236326|51246615	circRNA_018071	1:45654654|45670023	circRNA_008489	1:283800107|283841933
circRNA_016849	14:60181934|60183283	circRNA_009592	5:92502063|92512352	circRNA_015517	13:74658164|74673534	circRNA_005423	1:60917934|60959765
circRNA_017695	15:157407747|157409096	circRNA_018648	13:91012120|91022409	circRNA_018774	15:19247885|19263276	circRNA_012966	2:121253565|121295422
circRNA_014551	9:49298595|49299945	circRNA_004765	7:54561290|54571581	circRNA_014476	6:85269339|85284732	circRNA_010476	15:71703524|71745408
circRNA_001634	4:116008617|116009969	circRNA_010028	9:143383683|143393975	circRNA_000790	14:60727790|60743184	circRNA_019037	4:74095428|74137333
circRNA_003256	13:205664917|205666269	circRNA_020053	14:142262564|142272856	circRNA_014260	14:112006650|112022045	circRNA_014135	1:215715817|215757725
circRNA_018813	15:157407747|157409099	circRNA_021175	7:100793553|100803845	circRNA_011925	7:9022291|9037692	circRNA_010727	5:106543058|106585027
circRNA_020527	11:15548881|15550235	circRNA_012433	11:22231034|22241327	circRNA_017278	8:76057138|76072541	circRNA_019070	5:50696792|50738780
circRNA_003020	12:40897655|40899010	circRNA_020562	12:44776560|44786854	circRNA_003527	15:61383234|61398639	circRNA_013351	7:109783554|109825592
circRNA_010228	1:204835343|204836699	circRNA_021226	8:116836002|116846298	circRNA_011960	7:91966709|91982115	circRNA_013328	7:62884149|62926208
circRNA_011240	13:89625322|89626678	circRNA_021259	9:40775540|40785836	circRNA_000861	14:132329649|132345057	circRNA_011604	2:79837584|79879663
circRNA_009344	3:39643788|39645147	circRNA_012389	10:59554486|59564783	circRNA_006502	9:120426043|120441453	circRNA_018568	11:6761843|6803923
circRNA_021115	6:90570313|90571672	circRNA_019935	1:200790738|200801042	circRNA_008030	8:85966163|85981573	circRNA_014690	2:130813510|130855596
circRNA_005093	9:31869060|31870420	circRNA_011157	12:5278151|5288457	circRNA_006405	8:71459662|71475073	circRNA_002957	12:14242194|14284284
circRNA_006290	6:99769671|99771031	circRNA_020541	12:11456306|11466613	circRNA_006965	13:14230737|14246148	circRNA_017918	6:147575304|147617411
circRNA_014354	2:30462773|30464133	circRNA_009068	15:152385966|152396274	circRNA_012824	16:79063373|79078790	circRNA_001697	5:24783952|24826063
circRNA_002121	8:93220585|93221946	circRNA_002145	8:130481169|130491478	circRNA_011502	16:57338445|57353864	circRNA_000182	1:202944045|202986161
circRNA_004942	8:71458415|71459779	circRNA_015901	2:93851810|93862119	circRNA_012170	X:125365423|125380842	circRNA_017082	3:106268651|106310785
circRNA_017779	2:149811276|149812640	circRNA_005673	13:44098037|44108347	circRNA_018009	9:139606828|139622249	circRNA_005385	X:122506163|122548340
circRNA_002216	9:40572085|40573450	circRNA_011762	4:40343834|40354146	circRNA_015306	1:282213213|282228639	circRNA_013337	7:91884279|91926482
circRNA_010699	5:38432829|38434196	circRNA_016165	5:80416757|80427069	circRNA_003079	13:24061768|24077196	circRNA_003223	13:144543873|144586089
circRNA_018447	1:134653309|134654676	circRNA_019328	JH118743.1:5178|15490	circRNA_003453	14:132237197|132252629	circRNA_017756	2:65132488|65174711
circRNA_014078	1:13745741|13747109	circRNA_020634	13:144264028|144274341	circRNA_015603	14:37536658|37552090	circRNA_008138	9:85879365|85921589
circRNA_016432	9:19235063|19236432	circRNA_009205	18:53769001|53779316	circRNA_017988	9:50817859|50833295	circRNA_002573	1:127997793|128040020
circRNA_017711	16:47823935|47825304	circRNA_000322	10:37135909|37146225	circRNA_002699	1:240937441|240952881	circRNA_014460	5:87266266|87308493
circRNA_003865	18:53834407|53835777	circRNA_002594	1:141278731|141289047	circRNA_008715	13:18138045|18153485	circRNA_009415	3:123000541|123042772
circRNA_000459	12:16978073|16979444	circRNA_005801	14:114868474|114878790	circRNA_017416	1:10141811|10157251	circRNA_008558	10:58881715|58923959
circRNA_007762	5:72572851|72574222	circRNA_020797	16:78185199|78195515	circRNA_001821	6:69714724|69730166	circRNA_002843	10:58870241|58912493
circRNA_009915	9:7447947|7449318	circRNA_003562	15:85397016|85407334	circRNA_006866	11:15977687|15993129	circRNA_008243	X:22018872|22061151
circRNA_007196	14:140504669|140506041	circRNA_011281	13:167124287|167134609	circRNA_015773	16:52464865|52480307	circRNA_008854	13:205921704|205963984
circRNA_011722	3:110373462|110374835	circRNA_014533	8:112183571|112193894	circRNA_003787	17:49672394|49687839	circRNA_000031	1:30684925|30727210
circRNA_015940	2:146580999|146582372	circRNA_017488	1:215969220|215979543	circRNA_016857	14:71889478|71904925	circRNA_008450	1:232091999|232134297
circRNA_013215	5:111001237|111002612	circRNA_016736	11:21081332|21091663	circRNA_000742	14:16172107|16187559	circRNA_021180	7:121010890|121053188

circRNA_015150	1:24166856|24168231	circRNA_006014	2:90967315|90977647	circRNA_002058	8:10582286|10597739	circRNA_002724	1:259286768|259329084
circRNA_013249	6:82408604|82409980	circRNA_021361	X:41447025|41457363	circRNA_005740	13:190890857|190906311	circRNA_012835	17:24465226|24507544
circRNA_001454	3:84178465|84179842	circRNA_015007	15:144877669|144888008	circRNA_013964	9:79015624|79031079	circRNA_008630	12:996270|1038595
circRNA_008201	GL893953.2:92709|94086	circRNA_017018	2:52241712|52252052	circRNA_014688	2:125069347|125084807	circRNA_016692	1:256994751|257037086
circRNA_010350	13:8094772|8096149	circRNA_010282	10:44196320|44206662	circRNA_004146	3:98116845|98132309	circRNA_000304	10:29321805|29364146
circRNA_010888	9:77370451|77371829	circRNA_010698	5:33923729|33934071	circRNA_013677	13:43394660|43410124	circRNA_015735	15:133542340|133584711
circRNA_016096	4:109111891|109113269	circRNA_002934	11:70429764|70440107	circRNA_015794	17:36439071|36454536	circRNA_017275	8:57362540|57404915
circRNA_004737	7:26926482|26927861	circRNA_016232	6:88302844|88313189	circRNA_003233	13:151514055|151529521	circRNA_015542	13:126404165|126446550
circRNA_020523	11:5621437|5622816	circRNA_008937	14:83655944|83666291	circRNA_007484	2:90151050|90166516	circRNA_014807	17:43037337|43079731
circRNA_012669	14:82449633|82451014	circRNA_005982	2:60043010|60053359	circRNA_017923	7:10297937|10313405	circRNA_011132	11:508651|551063
circRNA_018935	2:112634093|112635475	circRNA_008524	10:33268605|33278954	circRNA_008471	1:256330546|256346024	circRNA_012367	10:47064149|47106567
circRNA_005282	GL896504.1:54077|55460	circRNA_008356	1:114173836|114184187	circRNA_013857	15:133579638|133595116	circRNA_003743	17:5895188|5937612
circRNA_019196	8:1269897|1271280	circRNA_002485	1:25529966|25540318	circRNA_005094	9:36992574|37008057	circRNA_013581	X:61016381|61058809
circRNA_000143	1:144946985|144948371	circRNA_008173	9:134281948|134292300	circRNA_002299	9:139646501|139661994	circRNA_012886	18:55380452|55422882
circRNA_008452	1:233195418|233196805	circRNA_014607	1:30979937|30990296	circRNA_005920	17:36500876|36516369	circRNA_014244	14:46157361|46199793
circRNA_002371	GL896282.2:21447|22835	circRNA_007325	16:51753464|51763824	circRNA_007695	4:129286788|129302281	circRNA_001038	16:24951713|24994149
circRNA_019823	8:78338051|78339439	circRNA_014103	1:64727564|64737924	circRNA_010936	X:73939459|73954955	circRNA_014949	9:24125972|24168454
circRNA_006975	13:26336118|26337507	circRNA_007971	7:124987073|124997437	circRNA_008946	14:114857540|114873040	circRNA_010070	GL894982.1:2829|45312
circRNA_009972	9:52649358|52650747	circRNA_020018	13:146730228|146740593	circRNA_006025	2:106257761|106273263	circRNA_019842	9:19269563|19312056
circRNA_004739	7:31306372|31307763	circRNA_004368	4:129602895|129613263	circRNA_004801	7:73081330|73096838	circRNA_002956	12:13573401|13615897
circRNA_017421	1:16333438|16334830	circRNA_008015	8:58742622|58752992	circRNA_001314	2:101141685|101157198	circRNA_000779	14:55592489|55634995
circRNA_005152	9:107358595|107359989	circRNA_006974	13:24879745|24890118	circRNA_021217	8:90653634|90669148	circRNA_013050	3:119701312|119743831
circRNA_015697	15:66248256|66249650	circRNA_007290	15:139201406|139211780	circRNA_019523	14:31361690|31377217	circRNA_011559	18:59442183|59484705
circRNA_017466	1:159904576|159905970	circRNA_021225	8:112535762|112546138	circRNA_010095	JH118963.1:68976|84505	circRNA_016752	12:15464790|15507313
circRNA_021320	GL894597.1:72864|74259	circRNA_011841	5:92232853|92243231	circRNA_018321	6:141363206|141378736	circRNA_020577	13:28113282|28155821
circRNA_001211	2:4157505|4158901	circRNA_008166	9:128120540|128130921	circRNA_018054	X:78796256|78811788	circRNA_020580	13:34615712|34658266
circRNA_011574	2:30041081|30042478	circRNA_018917	2:80452934|80463317	circRNA_017672	15:86591592|86607126	circRNA_006130	4:33418180|33460740
circRNA_006001	2:83234666|83236064	circRNA_012836	17:28213601|28223986	circRNA_001226	2:17755529|17771066	circRNA_021000	4:10801067|10843628
circRNA_008352	1:108448292|108449692	circRNA_008554	10:54114522|54124909	circRNA_013571	X:31558043|31573582	circRNA_010589	2:121372041|121414614
circRNA_017596	13:76858807|76860207	circRNA_002194	9:13308635|13319023	circRNA_015516	13:73319538|73335080	circRNA_020898	2:89767470|89810056
circRNA_019569	15:101522761|101524162	circRNA_004617	6:94778502|94788895	circRNA_000656	13:132927553|132943099	circRNA_008032	8:88824075|88866692
circRNA_017544	11:23609011|23610413	circRNA_012911	2:30034059|30044454	circRNA_021055	5:50586128|50601679	circRNA_011244	13:98113084|98155714
circRNA_002027	7:102246054|102247457	circRNA_016561	JH118500.1:21110|31505	circRNA_001560	4:36253818|36269370	circRNA_016712	10:13315914|13358546
circRNA_006932	12:35282347|35283750	circRNA_007756	5:68117667|68128063	circRNA_020114	2:26857203|26872755	circRNA_017172	6:62119447|62162089
circRNA_009673	6:98714001|98715404	circRNA_011713	3:98006243|98016641	circRNA_012587	13:144152728|144168281	circRNA_013664	10:17127705|17170362
circRNA_020963	3:72256382|72257786	circRNA_007679	4:106709033|106719435	circRNA_006334	7:46989796|47005350	circRNA_012251	1:127661208|127703879
circRNA_008041	8:103925876|103927281	circRNA_016506	9:144090434|144100836	circRNA_018937	2:120022510|120038064	circRNA_015559	13:149836368|149879040
circRNA_017747	2:16112934|16114339	circRNA_008613	11:56136069|56146474	circRNA_005379	X:121808444|121823999	circRNA_004620	6:99060571|99103249
circRNA_016916	15:118292366|118293772	circRNA_007521	2:147434535|147444947	circRNA_004714	7:10297937|10313493	circRNA_001088	17:14843150|14885851
circRNA_021336	GL896381.1:14361|15767	circRNA_006663	1:103173037|103183452	circRNA_018142	13:160288515|160304071	circRNA_011962	7:98966300|99009001
circRNA_010324	12:18731949|18733356	circRNA_014482	6:124817728|124828144	circRNA_019849	9:84199222|84214781	circRNA_001556	4:32672829|32715543
circRNA_013897	3:45602185|45603593	circRNA_020285	8:39588373|39598789	circRNA_021200	8:57803396|57818958	circRNA_011239	13:87172364|87215095
circRNA_019024	4:45555294|45556702	circRNA_014400	3:83018888|83029307	circRNA_001652	4:129634550|129650114	circRNA_013179	5:70576776|70619507
circRNA_002434	X:113348505|113349914	circRNA_001099	17:36148045|36158466	circRNA_012561	13:90755537|90771102	circRNA_011896	6:99216722|99259476
circRNA_005773	14:31408084|31409493	circRNA_003324	14:31362268|31372689	circRNA_019102	6:65265172|65280737	circRNA_005012	8:134379373|134422131
circRNA_007140	14:64064267|64065676	circRNA_020090	17:32253733|32264156	circRNA_010225	1:199984968|200000535	circRNA_014569	9:133465775|133508551
circRNA_018297	5:106346589|106347998	circRNA_004330	4:106709011|106719435	circRNA_016469	9:61784105|61799680	circRNA_020904	2:119223549|119266346
circRNA_000514	12:58063185|58064595	circRNA_006229	5:86815194|86825619	circRNA_008339	1:87802828|87818404	circRNA_018351	8:73274904|73317737
circRNA_001595	4:79034573|79035983	circRNA_020613	13:107007182|107017607	circRNA_002679	1:205511273|205526853	circRNA_008300	1:21206251|21249088
circRNA_003138	13:72865581|72866992	circRNA_018719	14:36220033|36230465	circRNA_010382	13:90869873|90885454	circRNA_001762	5:92200375|92243231
circRNA_017061	3:24725453|24726864	circRNA_008349	1:105756428|105766861	circRNA_017002	2:9129018|9144599	circRNA_001148	18:9961637|10004494
circRNA_008795	13:97366509|97367921	circRNA_014318	16:53238470|53248903	circRNA_020845	18:44790011|44805592	circRNA_019905	1:32069210|32112092

circRNA_015691	15:53848182|53849595	circRNA_017627	14:34702864|34713298	circRNA_021129	6:118010983|118026564	circRNA_012005	8:73387627|73430512
circRNA_002422	X:51830688|51832102	circRNA_006726	1:188117293|188127728	circRNA_001571	4:43558505|43574090	circRNA_006342	7:67504695|67547581
circRNA_012125	GL894826.2:43158|44572	circRNA_000461	12:18731949|18742385	circRNA_003984	2:103773335|103788922	circRNA_006507	9:129689965|129732908
circRNA_015783	16:75043882|75045297	circRNA_019242	9:34787|45223	circRNA_017282	8:82515546|82531135	circRNA_006156	4:88593582|88636542
circRNA_007076	13:166915927|166917343	circRNA_005450	1:134659825|134670263	circRNA_003737	17:717212|732802	circRNA_017512	10:16958022|17000982
circRNA_020941	3:29695496|29696912	circRNA_017947	8:3341344|3351783	circRNA_012046	9:13293149|13308739	circRNA_001623	4:106690698|106733675
circRNA_005507	1:241901877|241903294	circRNA_001555	4:31452929|31463369	circRNA_006199	5:38471187|38486778	circRNA_020833	18:18602505|18645486
circRNA_007931	7:73282517|73283935	circRNA_016218	6:64570542|64580982	circRNA_003901	2:41439642|41455239	circRNA_009103	16:47081487|47124469
circRNA_011163	12:15975437|15976855	circRNA_002891	11:16154643|16165084	circRNA_002031	7:105392983|105408581	circRNA_003302	14:14036518|14079510
circRNA_017588	13:37836480|37837898	circRNA_008783	13:87165815|87176256	circRNA_004023	2:139097672|139113270	circRNA_016312	7:97058335|97101356
circRNA_005384	X:121940001|121941420	circRNA_005648	13:24066753|24077196	circRNA_016317	7:102704610|102720215	circRNA_009694	6:132108169|132151229
circRNA_006352	7:87588710|87590129	circRNA_008678	12:45351778|45362223	circRNA_016750	12:14530678|14546286	circRNA_010797	7:65666367|65709436
circRNA_009631	6:45106338|45107759	circRNA_019134	6:139079394|139089841	circRNA_001796	6:44991359|45006970	circRNA_009763	7:65664182|65707257
circRNA_014393	3:72442656|72444077	circRNA_006525	GL893725.2:22588|33040	circRNA_015036	5:45551752|45567366	circRNA_017141	5:48837142|48880238
circRNA_011825	5:65466667|65468089	circRNA_004601	6:88253944|88264397	circRNA_004773	7:59801505|59817124	circRNA_017814	3:134654689|134697787
circRNA_016235	6:90240971|90242393	circRNA_014125	1:179091950|179102403	circRNA_009657	6:82980243|82995863	circRNA_011028	1:188136744|188179860
circRNA_015818	18:11525883|11527306	circRNA_004743	7:33100021|33110476	circRNA_016367	8:57800743|57816363	circRNA_004463	5:70620860|70663997
circRNA_009823	8:8825493|8826917	circRNA_016366	8:57790382|57800838	circRNA_006950	12:52765546|52781167	circRNA_002430	X:77199964|77243106
circRNA_012369	10:47105143|47106567	circRNA_009197	18:44782112|44792573	circRNA_015013	17:44564987|44580609	circRNA_010995	1:134611516|134654676
circRNA_012757	15:115949281|115950705	circRNA_007528	3:9324579|9335045	circRNA_006960	13:3092387|3108013	circRNA_002007	7:91938950|91982115
circRNA_004090	3:55892340|55893766	circRNA_008901	14:49377982|49388449	circRNA_018442	1:127661208|127676835	circRNA_001846	6:90571500|90614667
circRNA_013446	9:37007803|37009229	circRNA_015573	13:190993013|191003480	circRNA_016888	15:18570017|18585645	circRNA_013609	18:36472459|36515630
circRNA_008298	1:18701399|18702826	circRNA_009418	3:128253444|128263914	circRNA_009031	15:86607064|86622694	circRNA_010783	7:17209021|17252211
circRNA_012735	15:86509217|86510646	circRNA_007778	5:92237091|92247562	circRNA_012740	15:88619780|88635410	circRNA_013887	2:119165841|119209060
circRNA_009490	4:107748690|107750120	circRNA_019122	6:112318559|112329035	circRNA_017409	X:130737537|130753169	circRNA_019359	1:23208792|23252035
circRNA_016519	GL893852.2:66110|67540	circRNA_014386	3:45760384|45770861	circRNA_000823	14:83832559|83848192	circRNA_021003	4:31005099|31048345
circRNA_021378	X:107355777|107357207	circRNA_016086	4:81096531|81107008	circRNA_021042	5:4827605|4843238	circRNA_009880	8:108342978|108386252
circRNA_019865	GL893077.1:22716|24148	circRNA_020982	3:110306538|110317020	circRNA_005970	2:19824959|19840596	circRNA_007605	3:120307356|120350634
circRNA_007798	6:26111121|26112554	circRNA_006686	1:136466617|136477100	circRNA_012728	15:73900098|73915735	circRNA_013066	4:2565801|2609139
circRNA_000069	1:84047500|84048935	circRNA_007176	14:120768685|120779168	circRNA_016238	6:92111298|92126940	circRNA_012679	14:114857540|114900889
circRNA_018793	15:92420042|92421478	circRNA_005099	9:40522304|40532789	circRNA_000726	14:933854|949498	circRNA_008204	GL894261.2:47688|91044
circRNA_000064	1:64530710|64532147	circRNA_009459	4:55324656|55335142	circRNA_006732	1:197294373|197310017	circRNA_004312	4:90661612|90704987
circRNA_011416	15:85345503|85346940	circRNA_010009	9:116131627|116142114	circRNA_016662	1:176846274|176861919	circRNA_010711	5:70588009|70631439
circRNA_001227	2:18698775|18700214	circRNA_004644	6:108191915|108202404	circRNA_010997	1:134656768|134672419	circRNA_003774	17:43030480|43073920
circRNA_006406	8:71508448|71509887	circRNA_008873	14:10483485|10493976	circRNA_000505	12:51832595|51848255	circRNA_019891	X:86450626|86494087
circRNA_014561	9:101485366|101486805	circRNA_014761	1:97745504|97755995	circRNA_004204	3:122008499|122024159	circRNA_019777	6:148034940|148078417
circRNA_010275	10:29268486|29269926	circRNA_011223	13:50625935|50636427	circRNA_003211	13:142925649|142941310	circRNA_003800	18:2736819|2780302
circRNA_014793	14:65222253|65223693	circRNA_016507	9:153032605|153043101	circRNA_014879	8:86231350|86247013	circRNA_020791	16:48659817|48703328
circRNA_004860	7:128689753|128691194	circRNA_009936	9:36725705|36736202	circRNA_015634	14:77094824|77110489	circRNA_019379	1:134599618|134643130
circRNA_009091	16:29444659|29446101	circRNA_015178	1:80866821|80877325	circRNA_015212	1:134656752|134672419	circRNA_001196	18:59518595|59562118
circRNA_005206	9:144381172|144382617	circRNA_016911	15:115443614|115454120	circRNA_001411	3:40969228|40984897	circRNA_009045	15:116124302|116167830
circRNA_019933	1:200299009|200300454	circRNA_011742	4:16883656|16894163	circRNA_011128	10:71361138|71376807	circRNA_013922	4:139842217|139885766
circRNA_014765	1:215947923|215949369	circRNA_019099	6:41561594|41572102	circRNA_012990	3:4946357|4962030	circRNA_010966	1:45713269|45756836
circRNA_001266	2:70193426|70194875	circRNA_011373	14:131863897|131874408	circRNA_007690	4:117897403|117913079	circRNA_016909	15:111184182|111227757
circRNA_018462	1:175587208|175588657	circRNA_020194	5:38348311|38358827	circRNA_001715	5:50645243|50660921	circRNA_005350	X:73967201|74010804
circRNA_008097	9:30345906|30347357	circRNA_004979	8:93231187|93241704	circRNA_012919	2:46096292|46111970	circRNA_007223	15:36517174|36560784
circRNA_002802	10:34445346|34446798	circRNA_018698	14:7192057|7202575	circRNA_018059	X:109061119|109076797	circRNA_014618	1:282152370|282195982
circRNA_014612	1:176836675|176838127	circRNA_008275	X:121813479|121823999	circRNA_008141	9:96037782|96053461	circRNA_014773	10:66473871|66517483
circRNA_011045	1:227124801|227126254	circRNA_015920	2:127248841|127259362	circRNA_006806	10:13342866|13358546	circRNA_019568	15:97943737|97987408
circRNA_015379	11:20547894|20549347	circRNA_007839	6:85026828|85037351	circRNA_002539	1:94559522|94575209	circRNA_001731	5:69911961|69955639
circRNA_002999	12:36567831|36569285	circRNA_014781	13:50625935|50636458	circRNA_007275	15:116470717|116486409	circRNA_017812	3:130349256|130392936
circRNA_008443	1:216062375|216063829	circRNA_019677	3:98670746|98681269	circRNA_009305	2:139112346|139128038	circRNA_019646	2:130872680|130916366

circRNA_009566	5:72061590|72063044	circRNA_012012	8:86231350|86241874	circRNA_014916	13:130087801|130103494	circRNA_000377	11:5189667|5233354
circRNA_009010	15:60961638|60963094	circRNA_013851	15:117869607|117880131	circRNA_018463	1:175780781|175796474	circRNA_010749	6:79809424|79853129
circRNA_013433	9:2616908|2618364	circRNA_016539	GL895152.1:27183|37712	circRNA_003633	15:122898738|122914434	circRNA_001018	15:131248910|131292628
circRNA_010221	1:186656379|186657838	circRNA_013658	1:176805305|176815836	circRNA_011499	16:55636224|55651926	circRNA_020741	15:75475656|75519381
circRNA_000733	14:10633685|10635146	circRNA_020671	14:31293176|31303710	circRNA_012385	10:56077005|56092707	circRNA_017932	7:60758058|60801794
circRNA_002392	MT:1401|2862	circRNA_009595	5:94439224|94449760	circRNA_007233	15:54635062|54650765	circRNA_000135	1:143396359|143440106
circRNA_005963	2:203723|205184	circRNA_002766	1:305559423|305569960	circRNA_002973	12:16852309|16868014	circRNA_003981	2:98283043|98326796
circRNA_018273	4:114796951|114798413	circRNA_020128	3:11444069|11454606	circRNA_008544	10:50877061|50892767	circRNA_020011	13:131405105|131448858
circRNA_003794	17:55856695|55858158	circRNA_017781	3:5269524|5280062	circRNA_012663	14:77617872|77633580	circRNA_014883	9:14158052|14201808
circRNA_005879	15:147194816|147196279	circRNA_004559	6:64468581|64479121	circRNA_006896	11:56283465|56299174	circRNA_010326	12:20387114|20430888
circRNA_016751	12:15319056|15320519	circRNA_003235	13:155848724|155859269	circRNA_019066	5:38581675|38597389	circRNA_018443	1:128292382|128336163
circRNA_018799	15:112175034|112176497	circRNA_019982	12:36535677|36546223	circRNA_006672	1:110532620|110548336	circRNA_020426	1:176789554|176833339
circRNA_000720	13:212995887|212997351	circRNA_013694	15:97747096|97757643	circRNA_016815	13:130288798|130304514	circRNA_002748	1:283798123|283841933
circRNA_010951	1:14083797|14085261	circRNA_018424	1:80660555|80671102	circRNA_019168	7:65666367|65682090	circRNA_012284	1:188136037|188179860
circRNA_004420	5:32070350|32071815	circRNA_000987	15:112138368|112148920	circRNA_003298	14:11428131|11443856	circRNA_018432	1:106884369|106928193
circRNA_004966	8:86231350|86232815	circRNA_015056	8:127787109|127797662	circRNA_015834	18:36452161|36467886	circRNA_009923	9:19264985|19308814
circRNA_009472	4:73648054|73649519	circRNA_012380	10:54023860|54034416	circRNA_019202	8:32701671|32717396	circRNA_000593	13:59840070|59883903
circRNA_019784	7:4044467|4045934	circRNA_018153	14:36418799|36429355	circRNA_018551	10:50698422|50714152	circRNA_018005	9:125482616|125526453
circRNA_002984	12:23265330|23266798	circRNA_005314	X:14262458|14273016	circRNA_001785	6:18405869|18421604	circRNA_000957	15:85340631|85384479
circRNA_007636	4:37102480|37103948	circRNA_005328	X:43366558|43377117	circRNA_004616	6:94773159|94788895	circRNA_009826	8:20703886|20747741
circRNA_014925	16:51805936|51807404	circRNA_006986	13:35732800|35743359	circRNA_010639	3:114478505|114494241	circRNA_019584	16:25416913|25460777
circRNA_010542	18:16038715|16040184	circRNA_005255	GL894864.1:45296|55859	circRNA_018557	10:66158459|66174196	circRNA_015319	1:311023437|311067320
circRNA_000913	15:55256994|55258465	circRNA_013680	13:51132717|51143281	circRNA_001106	17:43037337|43053075	circRNA_012051	9:25341606|25385497
circRNA_016184	5:110487947|110489418	circRNA_016171	5:87157448|87168012	circRNA_018562	11:266572|282312	circRNA_001810	6:63452187|63496102
circRNA_011048	1:231032079|231033551	circRNA_013264	6:99506005|99516571	circRNA_013535	GL894982.1:29571|45312	circRNA_015354	10:50877061|50920985
circRNA_014782	13:75148128|75149600	circRNA_001197	18:59551551|59562118	circRNA_016034	3:126256536|126272280	circRNA_013296	6:153769525|153813460
circRNA_016782	13:34979462|34980935	circRNA_016841	14:33833203|33843770	circRNA_000448	12:14679564|14695314	circRNA_003048	12:54621571|54665529
circRNA_008380	1:142576039|142577513	circRNA_004403	5:4583340|4593908	circRNA_011964	7:100745888|100761638	circRNA_017645	14:100158947|100202909
circRNA_018018	GL892550.1:15704|17178	circRNA_007567	3:85096895|85107464	circRNA_007479	2:87133080|87148831	circRNA_017483	1:200808601|200852579
circRNA_005302	MT:1403|2878	circRNA_012396	10:65664902|65675473	circRNA_016415	8:143338022|143353774	circRNA_020008	13:114754401|114798392
circRNA_000090	1:117496938|117498416	circRNA_015475	13:12199397|12209968	circRNA_021009	4:41064761|41080514	circRNA_006793	1:297365575|297409578
circRNA_008816	13:131631736|131633215	circRNA_012331	1:305382060|305392639	circRNA_014534	8:117204489|117220244	circRNA_020371	1:30921618|30965623
circRNA_016979	18:15806186|15807668	circRNA_001511	3:120151930|120162510	circRNA_009474	4:81043876|81059633	circRNA_005209	9:150354490|150398500
circRNA_014060	GL893171.1:5494|6977	circRNA_009766	7:69660094|69670674	circRNA_014671	16:37498946|37514704	circRNA_018633	13:50980036|51024060
circRNA_000311	10:33080414|33081898	circRNA_007874	6:137387063|137397644	circRNA_001293	2:87085725|87101485	circRNA_005173	9:129664193|129708229
circRNA_011464	16:21136449|21137933	circRNA_014741	9:73323019|73333601	circRNA_004936	8:58350412|58366177	circRNA_016661	1:176142565|176186618
circRNA_009337	3:24354996|24356481	circRNA_005549	10:31143161|31153744	circRNA_014333	17:49147794|49163564	circRNA_010735	6:35124281|35168344
circRNA_015424	12:14528146|14529631	circRNA_006463	9:31044963|31055546	circRNA_010996	1:134654492|134670263	circRNA_012569	13:102280273|102324338
circRNA_013164	5:38348311|38349797	circRNA_019085	5:92512231|92522814	circRNA_019362	1:38719885|38735658	circRNA_006057	3:8995124|9039219
circRNA_002395	MT:1661|3149	circRNA_008727	13:33146950|33157534	circRNA_008254	X:67430148|67445922	circRNA_003443	14:122257724|122301834
circRNA_016814	13:126563388|126564876	circRNA_018360	8:146931116|146941701	circRNA_018484	1:195114912|195130686	circRNA_009272	2:88675453|88719584
circRNA_007505	2:112591635|112593124	circRNA_003054	12:62513102|62523689	circRNA_019101	6:64468581|64484356	circRNA_020106	18:55378722|55422882
circRNA_010107	X:10418106|10419595	circRNA_000508	12:52753646|52764234	circRNA_019215	8:77419741|77435516	circRNA_019012	3:130877269|130921437
circRNA_013663	10:12274354|12275844	circRNA_008777	13:82870481|82881069	circRNA_020417	1:146847996|146863771	circRNA_000977	15:103617399|103661579
circRNA_002169	9:39472|40964	circRNA_007274	15:116425424|116436013	circRNA_021096	6:25845230|25861005	circRNA_013230	6:38250913|38295097
circRNA_006404	8:58349146|58350639	circRNA_010408	13:167914163|167924754	circRNA_001178	18:25511592|25527369	circRNA_016162	5:76314134|76358338
circRNA_014070	15:117371369|117372862	circRNA_006702	1:154139505|154150097	circRNA_005341	X:67430148|67445926	circRNA_008637	12:13078736|13122950
circRNA_014216	13:130538051|130539544	circRNA_008193	GL893520.1:9921|20513	circRNA_016166	5:82659439|82675225	circRNA_003853	18:43942640|43986861
circRNA_010756	6:105518975|105520469	circRNA_018968	3:32848231|32858823	circRNA_013564	X:8565836|8581627	circRNA_002260	9:83318844|83363067
circRNA_004437	5:49604698|49606193	circRNA_017350	9:127873164|127883759	circRNA_013702	18:59319799|59335593	circRNA_012873	18:26272854|26317097
circRNA_020307	9:21749350|21750846	circRNA_003677	16:25535497|25546093	circRNA_013979	X:85919732|85935529	circRNA_017583	13:21030220|21074468
circRNA_005750	13:212947616|212949113	circRNA_010885	9:72061438|72072037	circRNA_019230	8:109055955|109071752	circRNA_002831	10:50698422|50742685

circRNA_006247	6:5097896|5099394	circRNA_012894	18:60697210|60707810	circRNA_000458	12:16876107|16891912	circRNA_003014	12:38757575|38801846
circRNA_001702	5:36442698|36444197	circRNA_017590	13:44645509|44656109	circRNA_017815	3:135176536|135192341	circRNA_004439	5:49928478|49972756
circRNA_006790	1:293488318|293489817	circRNA_019609	18:41970023|41980627	circRNA_017407	X:127123795|127139604	circRNA_017218	7:21545430|21589708
circRNA_003769	17:40996159|40997659	circRNA_006884	11:26291064|26301669	circRNA_015987	3:74737135|74752948	circRNA_003611	15:116124302|116168588
circRNA_003804	18:5627894|5629396	circRNA_015256	1:215947923|215958529	circRNA_005026	8:144223745|144239562	circRNA_005007	8:128735200|128779487
circRNA_014974	10:76304609|76306111	circRNA_000462	12:19128236|19138843	circRNA_004311	4:90657864|90673688	circRNA_019608	18:36461492|36505790
circRNA_011083	10:16947860|16949363	circRNA_003425	14:115746772|115757379	circRNA_010159	X:127320459|127336284	circRNA_004346	4:117913001|117957301
circRNA_008539	10:48232668|48234172	circRNA_003136	13:72821916|72832527	circRNA_000205	1:227181487|227197323	circRNA_019473	13:36383856|36428161
circRNA_017376	GL895584.1:6134|7638	circRNA_002396	MT:1661|12274	circRNA_012683	14:116563564|116579401	circRNA_013654	1:18676193|18720511
circRNA_003941	2:83234559|83236064	circRNA_011771	4:62999230|63009847	circRNA_013061	3:130877269|130893130	circRNA_006032	2:119198668|119242988
circRNA_007285	15:133542340|133543845	circRNA_014809	17:59429157|59439777	circRNA_015958	3:24707804|24723668	circRNA_001281	2:81260228|81304602
circRNA_011249	13:103091330|103092835	circRNA_017861	5:50689038|50699658	circRNA_016785	13:37597575|37613439	circRNA_003535	15:63801735|63846137
circRNA_017958	8:71569151|71570656	circRNA_012940	2:80548536|80559157	circRNA_006940	12:39707486|39723351	circRNA_013380	8:40299206|40343610
circRNA_015119	13:146208594|146210100	circRNA_016114	4:135669086|135679707	circRNA_012632	14:32258589|32274456	circRNA_018766	14:136731189|136775595
circRNA_020857	2:7660552|7662058	circRNA_016318	7:102966554|102977175	circRNA_003739	17:1359188|1375056	circRNA_001170	18:18671313|18715721
circRNA_002967	12:16030982|16032491	circRNA_019688	3:120296840|120307461	circRNA_011254	13:126563388|126579257	circRNA_002496	1:45735708|45780118
circRNA_009902	8:139868072|139869581	circRNA_008436	1:208304839|208315461	circRNA_002493	1:40936826|40952696	circRNA_015448	12:38628145|38672576
circRNA_015416	12:11467637|11469146	circRNA_021262	9:46486141|46496763	circRNA_021230	8:123965840|123981710	circRNA_007614	3:125854763|125899205
circRNA_019344	X:68373379|68374889	circRNA_005406	1:18451548|18462171	circRNA_009386	3:98684730|98700602	circRNA_016697	1:280655936|280700379
circRNA_021358	X:31453580|31455092	circRNA_006956	13:1187021|1197644	circRNA_000101	1:125143608|125159485	circRNA_007222	15:36377634|36422115
circRNA_018120	12:44742567|44744081	circRNA_021045	5:17115286|17125911	circRNA_013277	6:136724832|136740709	circRNA_019669	3:42032747|42077233
circRNA_003778	17:44543886|44545401	circRNA_004480	5:82625769|82636395	circRNA_008967	14:138435635|138451513	circRNA_021218	8:90653634|90698129
circRNA_007563	3:74777358|74778873	circRNA_019274	9:78691792|78702418	circRNA_010223	1:195169049|195184929	circRNA_012705	15:9456353|9500850
circRNA_007367	17:57066927|57068443	circRNA_005701	13:100697258|100707887	circRNA_007673	4:90642228|90658112	circRNA_009391	3:104155199|104199715
circRNA_010640	3:121673683|121675200	circRNA_012461	12:7990373|8001002	circRNA_002702	1:242717985|242733870	circRNA_013411	8:112636025|112680543
circRNA_014387	3:48850375|48851892	circRNA_008125	9:70877652|70888285	circRNA_012927	2:54834985|54850871	circRNA_011101	10:35244566|35289091
circRNA_021093	6:18449174|18450692	circRNA_015904	2:100941232|100951865	circRNA_013900	3:104155199|104171085	circRNA_008156	9:115774313|115818856
circRNA_000339	10:49672328|49673847	circRNA_006845	10:54210961|54221596	circRNA_013126	4:117099716|117115606	circRNA_018581	11:25890872|25935442
circRNA_010403	13:155796342|155797862	circRNA_013172	5:52089724|52100360	circRNA_000139	1:143955137|143971028	circRNA_005543	10:18123982|18168555
circRNA_016233	6:88343002|88344522	circRNA_004295	4:73577490|73588127	circRNA_012437	11:35087651|35103547	circRNA_004219	3:135358108|135402694
circRNA_002209	9:32147065|32148586	circRNA_020816	17:51654188|51664826	circRNA_013480	9:102587388|102603287	circRNA_008100	9:36691555|36736202
circRNA_009227	2:28883702|28885224	circRNA_009146	17:41820950|41831590	circRNA_017803	3:109904183|109920084	circRNA_011011	1:143361257|143405910
circRNA_012987	3:161499|163021	circRNA_013382	8:44136959|44147599	circRNA_001173	18:18699816|18715721	circRNA_001144	18:5395091|5439747
circRNA_005735	13:155796339|155797862	circRNA_013954	8:109627162|109637802	circRNA_011878	6:69714260|69730166	circRNA_004888	8:20662920|20707578
circRNA_008749	13:44084999|44086522	circRNA_016622	1:16463108|16473752	circRNA_021121	6:99060571|99076477	circRNA_002767	1:311422004|311466666
circRNA_016253	6:119247435|119248958	circRNA_006371	7:121213886|121224532	circRNA_004031	2:145725178|145741087	circRNA_013169	5:50654972|50699658
circRNA_020723	14:148348272|148349795	circRNA_013780	1:200287462|200298109	circRNA_017030	2:87936746|87952657	circRNA_014263	14:115712680|115757379
circRNA_016573	X:14300648|14302172	circRNA_016193	6:16261615|16272264	circRNA_012864	18:10894089|10910003	circRNA_002966	12:15573276|15617981
circRNA_013417	8:126730108|126731633	circRNA_013898	3:45690728|45701378	circRNA_014300	15:116138978|116154892	circRNA_002964	12:15231041|15275808
circRNA_001712	5:48775578|48777104	circRNA_006839	10:50863858|50874511	circRNA_005027	8:144223745|144239660	circRNA_020672	14:31362268|31407036
circRNA_014435	4:121606785|121608311	circRNA_012400	10:77698486|77709139	circRNA_018676	13:156888783|156904698	circRNA_003837	18:25506902|25551680
circRNA_018747	14:82922376|82923904	circRNA_005256	GL894906.1:15964|26620	circRNA_021141	6:147566043|147581958	circRNA_006697	1:145394702|145439488
circRNA_001625	4:106997229|106998758	circRNA_015346	10:45772717|45783374	circRNA_006423	8:93220585|93236501	circRNA_014744	9:79899441|79944229
circRNA_011782	4:96774978|96776508	circRNA_016536	GL894943.1:25517|36174	circRNA_013704	2:87830162|87846078	circRNA_006256	6:27317005|27361798
circRNA_014360	2:52057851|52059381	circRNA_002569	1:126926296|126936954	circRNA_005143	9:88644143|88660063	circRNA_013359	7:124952565|124997437
circRNA_003517	15:53111670|53113201	circRNA_011475	16:27101321|27111983	circRNA_008985	15:19253545|19269467	circRNA_014890	9:124435848|124480730
circRNA_000719	13:211155143|211156675	circRNA_012952	2:89782086|89792748	circRNA_019003	3:115418067|115433989	circRNA_008781	13:87095883|87140810
circRNA_010507	16:51837857|51839390	circRNA_003569	15:86498639|86509303	circRNA_019606	18:15086907|15102829	circRNA_010939	X:85916081|85961011
circRNA_011264	13:142814626|142816160	circRNA_020400	1:110340582|110351249	circRNA_009154	17:45675110|45691034	circRNA_021285	9:117795916|117840855
circRNA_020334	9:116237046|116238582	circRNA_016538	GL895152.1:20630|31299	circRNA_004839	7:103045223|103061148	circRNA_012982	2:150742471|150787411
circRNA_005678	13:72554931|72556469	circRNA_013760	GL893077.1:39746|50416	circRNA_000333	10:46597762|46613689	circRNA_000034	1:32124162|32169111
circRNA_008713	13:15492585|15494125	circRNA_015683	15:19240976|19251646	circRNA_002498	1:56080746|56096675	circRNA_011009	1:143245293|143290256

circRNA_017021	2:71759932|71761473	circRNA_005270	GL896097.1:1221|11892	circRNA_010793	7:62827072|62843001	circRNA_005485	1:202941193|202986161
circRNA_001380	3:6704057|6705599	circRNA_015015	17:58803349|58814020	circRNA_011705	3:90576383|90592312	circRNA_018266	4:73737881|73782852
circRNA_015831	18:30295932|30297474	circRNA_016621	1:16112729|16123400	circRNA_005784	14:49996854|50012794	circRNA_001122	17:51997158|52042134
circRNA_006445	9:3017504|3019047	circRNA_003431	14:117272265|117282937	circRNA_006614	1:23046965|23062905	circRNA_008735	13:37934642|37979622
circRNA_007833	6:76037160|76038703	circRNA_016517	GL893704.1:26687|37359	circRNA_018605	12:44007881|44023821	circRNA_020376	1:45632972|45677973
circRNA_012843	17:44579066|44580609	circRNA_021321	GL894608.1:5723|16395	circRNA_012195	1:30921618|30937560	circRNA_010296	10:62722156|62767160
circRNA_018063	1:1175729|1177272	circRNA_003215	13:143962683|143973357	circRNA_000724	14:418144|434092	circRNA_010772	6:147612968|147657973
circRNA_000758	14:31136362|31137906	circRNA_006451	9:13693438|13704113	circRNA_001228	2:20100182|20116135	circRNA_013829	13:167788266|167833274
circRNA_007461	2:72186915|72188459	circRNA_003072	13:19726699|19737377	circRNA_008502	1:306181496|306197451	circRNA_013696	15:124584175|124629189
circRNA_016407	8:140627896|140629440	circRNA_017312	9:11801107|11811785	circRNA_007174	14:115739339|115755297	circRNA_007554	3:55906033|55951059
circRNA_007198	14:140971850|140973395	circRNA_004081	3:43036166|43046846	circRNA_014549	9:32419173|32435131	circRNA_000043	1:40891902|40936935
circRNA_009336	3:24160303|24161848	circRNA_020026	13:213173113|213183793	circRNA_001663	4:136143187|136159147	circRNA_008034	8:91602564|91647603
circRNA_020674	14:38276003|38277548	circRNA_011460	15:157085779|157096463	circRNA_012013	8:93207545|93223505	circRNA_011084	10:16955933|17000982
circRNA_008390	1:155827295|155828841	circRNA_004285	4:68353630|68364318	circRNA_002338	GL893884.1:31770|47747	circRNA_004543	6:32179801|32224859
circRNA_017231	7:80293549|80295095	circRNA_008587	11:14837406|14848094	circRNA_018405	1:17245871|17261849	circRNA_000063	1:63827196|63872257
circRNA_002282	9:115697945|115699492	circRNA_018938	2:120412824|120423513	circRNA_014855	6:141369686|141385665	circRNA_020295	8:91961908|92006984
circRNA_009868	8:92674063|92675610	circRNA_020136	3:45419321|45430012	circRNA_002670	1:202954152|202970134	circRNA_000023	1:18675426|18720511
circRNA_003713	16:47900020|47901568	circRNA_002372	GL896286.1:17238|27932	circRNA_004583	6:81306905|81322888	circRNA_021102	6:48344625|48389725
circRNA_021162	7:43617655|43619203	circRNA_011863	6:13088932|13099628	circRNA_005126	9:63116301|63132287	circRNA_017693	15:145557642|145602786
circRNA_004396	5:477943|479492	circRNA_001716	5:50900169|50910867	circRNA_001230	2:28869234|28885224	circRNA_015365	10:76272294|76317440
circRNA_011497	16:52931560|52933109	circRNA_018047	X:43179268|43189968	circRNA_012214	1:59739762|59755752	circRNA_008559	10:59564631|59609785
circRNA_003206	13:135146950|135148500	circRNA_012074	9:96042760|96053461	circRNA_012781	15:142005769|142021761	circRNA_015914	2:120950442|120995610
circRNA_010568	2:52058462|52060012	circRNA_005492	1:208503114|208513821	circRNA_009048	15:117892875|117908868	circRNA_015642	14:80442333|80487543
circRNA_020991	3:121950526|121952077	circRNA_016486	9:100588876|100599583	circRNA_012564	13:90919603|90935596	circRNA_012874	18:26334427|26379639
circRNA_001462	3:92460203|92461755	circRNA_019882	JH118944.1:245127|255840	circRNA_009691	6:125479400|125495394	circRNA_013988	1:255662209|255707430
circRNA_017122	4:109009100|109010652	circRNA_014872	8:32878313|32889029	circRNA_017761	2:79837584|79853579	circRNA_000549	13:28131575|28176864
circRNA_016549	GL896286.1:17238|18791	circRNA_008247	X:40497155|40507872	circRNA_013084	4:39622405|39638405	circRNA_018015	GL892397.1:95227|140520
circRNA_020649	13:166909686|166911239	circRNA_000326	10:43538376|43549096	circRNA_011108	10:46597688|46613689	circRNA_018353	8:110767547|110812866
circRNA_011711	3:92457168|92458722	circRNA_019847	9:50822572|50833295	circRNA_006645	1:86322010|86338016	circRNA_015455	12:46564506|46609836
circRNA_017058	3:11517980|11519534	circRNA_006667	1:108805541|108816265	circRNA_001419	3:51111489|51127498	circRNA_010426	14:31361690|31407036
circRNA_006677	1:123053053|123054608	circRNA_008076	9:8935966|8946694	circRNA_015727	15:117892858|117908868	circRNA_000054	1:58472238|58517592
circRNA_007284	15:131291073|131292628	circRNA_015611	14:51737786|51748514	circRNA_003675	16:24978133|24994149	circRNA_005464	1:151979105|152024459
circRNA_019885	X:19232020|19233575	circRNA_000839	14:113066724|113077453	circRNA_006415	8:82528463|82544481	circRNA_001615	4:98245901|98291288
circRNA_008056	8:129831168|129832724	circRNA_008812	13:130079549|130090279	circRNA_016386	8:89359060|89375078	circRNA_006209	5:56918746|56964141
circRNA_010109	X:14271460|14273016	circRNA_014058	8:141080303|141091035	circRNA_013905	3:120136072|120152093	circRNA_005928	17:43208017|43253419
circRNA_013582	X:64632081|64633637	circRNA_015145	1:18440912|18451652	circRNA_020984	3:114476157|114492181	circRNA_017411	X:138587662|138633095
circRNA_005985	2:61870482|61872040	circRNA_000354	10:62681670|62692414	circRNA_000330	10:46597662|46613689	circRNA_016683	1:233002746|233048185
circRNA_018610	12:48232839|48234400	circRNA_004677	6:143048817|143059562	circRNA_006743	1:209443302|209459339	circRNA_011763	4:40343834|40389291
circRNA_007369	17:57268654|57270219	circRNA_002985	12:23395100|23405847	circRNA_015240	1:202655424|202671463	circRNA_019076	5:70039585|70085045
circRNA_007052	13:131630312|131631880	circRNA_007733	5:31795585|31806332	circRNA_013980	X:113339294|113355334	circRNA_011942	7:57241844|57287321
circRNA_019422	10:29350935|29352504	circRNA_008598	11:22587236|22597989	circRNA_020413	1:136476985|136493027	circRNA_000624	13:98146479|98191973
circRNA_019616	2:8385039|8386609	circRNA_009831	8:32700211|32710967	circRNA_015020	18:36567649|36583692	circRNA_012160	X:77116736|77162241
circRNA_005010	8:133765118|133766690	circRNA_021342	JH118611.1:14573|25329	circRNA_016942	16:47809258|47825304	circRNA_019730	5:53733923|53779433
circRNA_008403	1:176740564|176742136	circRNA_020017	13:144384384|144395141	circRNA_005924	17:39442884|39458931	circRNA_002098	8:74357360|74402890
circRNA_017028	2:85980166|85981738	circRNA_015668	14:134764457|134775215	circRNA_009488	4:106717627|106733675	circRNA_004107	3:74727498|74773032
circRNA_017562	12:23750110|23751682	circRNA_020035	14:48561479|48572244	circRNA_002942	11:76626393|76642442	circRNA_016658	1:151769349|151814884
circRNA_017660	15:51628512|51630084	circRNA_012984	2:154506379|154517146	circRNA_006722	1:181043371|181059423	circRNA_005572	10:76594070|76639629
circRNA_005876	15:133817235|133818808	circRNA_017576	12:47646614|47657382	circRNA_001162	18:15051566|15067619	circRNA_015561	13:154679441|154725000
circRNA_013343	7:94854568|94856141	circRNA_004842	7:109814818|109825592	circRNA_018314	6:99178161|99194221	circRNA_010954	1:15907346|15952912
circRNA_014412	3:137691737|137693310	circRNA_017438	1:85692377|85703152	circRNA_019130	6:136018110|136034171	circRNA_013813	13:42346716|42392300
circRNA_000418	11:53756939|53758513	circRNA_017802	3:109696599|109707374	circRNA_009475	4:88783359|88799421	circRNA_007857	6:113121990|113167577
circRNA_013266	6:101007031|101008605	circRNA_007743	5:45994543|46005319	circRNA_005657	13:31063981|31080046	circRNA_005852	15:93542518|93588108

circRNA_004111	3:75950705|75952280	circRNA_012761	15:116361198|116371977	circRNA_008621	11:72298174|72314239	circRNA_014277	15:50827984|50873588
circRNA_006337	7:57967998|57969573	circRNA_013129	4:121532347|121543129	circRNA_015101	6:146042987|146059055	circRNA_015984	3:73603742|73649349
circRNA_018357	8:133765114|133766690	circRNA_014210	13:90953148|90963935	circRNA_016124	5:4775102|4791147	circRNA_008086	9:13336157|13381768
circRNA_019000	3:107441881|107443457	circRNA_006458	9:22396993|22407781	circRNA_016373	8:69855152|69871223	circRNA_015811	18:9563174|9608802
circRNA_003357	14:59078127|59079705	circRNA_020408	1:129010279|129021068	circRNA_008478	1:267674313|267690386	circRNA_001733	5:70039585|70085224
circRNA_007560	3:61587710|61589291	circRNA_005347	X:70970340|70981131	circRNA_014930	3:57812353|57828426	circRNA_007338	17:5933495|5979140
circRNA_009727	7:16766073|16767654	circRNA_000435	11:86951625|86962417	circRNA_004008	2:128789190|128805266	circRNA_014307	15:156396928|156442590
circRNA_002351	GL894597.1:44194|45776	circRNA_003154	13:83588162|83598954	circRNA_017577	12:51473563|51489639	circRNA_013944	7:76463695|76509406
circRNA_018216	2:5809514|5811096	circRNA_006970	13:20319983|20330776	circRNA_019051	4:129522757|129538840	circRNA_008871	14:3160095|3205830
circRNA_009968	9:50855170|50856753	circRNA_009442	4:39611818|39622613	circRNA_004940	8:69688714|69704803	circRNA_014044	14:115712680|115758424
circRNA_003597	15:111197129|111198713	circRNA_010425	14:31000287|31011082	circRNA_020988	3:121362925|121379020	circRNA_001395	3:24931501|24977247
circRNA_012748	15:105380326|105381910	circRNA_002147	8:133864713|133875510	circRNA_007964	7:119774053|119790150	circRNA_015826	18:24457721|24503485
circRNA_018430	1:101889031|101890615	circRNA_004172	3:109787455|109798252	circRNA_001035	16:22541545|22557643	circRNA_009440	4:35844936|35890743
circRNA_017468	1:161202388|161203973	circRNA_003386	14:78015574|78026373	circRNA_007906	7:33100021|33116120	circRNA_019203	8:32865811|32911622
circRNA_013248	6:79558469|79560055	circRNA_001553	4:19615523|19626323	circRNA_000752	14:30050907|30067011	circRNA_001473	3:101033374|101079220
circRNA_017758	2:68900596|68902182	circRNA_020432	1:181161592|181172394	circRNA_007034	13:102287901|102304005	circRNA_005424	1:63683313|63729178
circRNA_005287	JH118545.1:40523|42110	circRNA_014730	8:32345748|32356554	circRNA_015343	10:37661671|37677784	circRNA_004767	7:57234885|57280795
circRNA_012502	12:58063004|58064591	circRNA_007751	5:50990032|51000839	circRNA_009913	9:71135|87249	circRNA_011753	4:35844936|35890859
circRNA_018425	1:85791534|85793122	circRNA_002110	8:83269731|83280539	circRNA_017839	4:93613223|93629339	circRNA_004961	8:82960858|83006817
circRNA_001914	6:141384076|141385665	circRNA_002929	11:55451765|55462575	circRNA_002191	9:11795661|11811785	circRNA_003707	16:47778049|47824009
circRNA_006005	2:86283747|86285336	circRNA_018323	6:145402515|145413325	circRNA_004744	7:33100021|33116145	circRNA_011719	3:103056866|103102853
circRNA_006363	7:103748153|103749742	circRNA_006920	12:21021053|21031864	circRNA_006973	13:23714244|23730368	circRNA_020724	15:3755802|3801791
circRNA_003626	15:120985046|120986636	circRNA_009260	2:80549531|80560343	circRNA_009242	2:48826973|48843100	circRNA_014284	15:73524173|73570167
circRNA_000513	12:58063004|58064595	circRNA_003421	14:113299285|113310103	circRNA_014787	13:202682827|202698955	circRNA_018628	13:36984576|37030576
circRNA_009565	5:71944285|71945876	circRNA_002727	1:264034002|264044821	circRNA_000185	1:202970028|202986161	circRNA_019571	15:105438181|105484194
circRNA_006961	13:3130975|3132568	circRNA_012279	1:181161592|181172411	circRNA_016032	3:122011566|122027701	circRNA_011479	16:29069843|29115875
circRNA_013253	6:86728252|86729846	circRNA_014239	14:36577519|36588339	circRNA_021119	6:98732359|98748495	circRNA_005891	16:29069843|29115878
circRNA_014385	3:45554717|45556311	circRNA_003032	12:45475763|45486586	circRNA_000410	11:26291064|26307201	circRNA_002313	9:150621076|150667131
circRNA_019665	3:27857852|27859446	circRNA_011191	13:1141433|1152257	circRNA_011899	6:108611407|108627548	circRNA_003470	14:142803973|142850048
circRNA_012849	17:59261552|59263147	circRNA_019629	2:80566467|80577295	circRNA_011593	2:52990682|53006824	circRNA_005936	18:5206226|5252339
circRNA_013882	2:52263629|52265225	circRNA_009455	4:46390670|46401504	circRNA_003687	16:36676971|36693116	circRNA_005642	12:62904084|62950205
circRNA_019107	6:82409864|82411462	circRNA_018388	GL895413.1:42635|53469	circRNA_004657	6:125923718|125939863	circRNA_012562	13:90910708|90956856
circRNA_005110	9:44292300|44293899	circRNA_010552	2:3011482|3022317	circRNA_010299	11:250979|267124	circRNA_006036	2:122033113|122079281
circRNA_019347	X:73873598|73875197	circRNA_003741	17:5299125|5309965	circRNA_020510	10:49571586|49587733	circRNA_014717	6:32195144|32241328
circRNA_001409	3:39621256|39622856	circRNA_011908	6:137394338|137405178	circRNA_016019	3:113310620|113326774	circRNA_011505	16:79953195|79999401
circRNA_000547	13:26315726|26317327	circRNA_016914	15:117366653|117377494	circRNA_003502	15:20231068|20247223	circRNA_006151	4:77591055|77637270
circRNA_002861	10:75687658|75689259	circRNA_003980	2:98275617|98286459	circRNA_017834	4:73597912|73614069	circRNA_016594	X:70452530|70498760
circRNA_005461	1:145436546|145438147	circRNA_015552	13:144054919|144065761	circRNA_001245	2:50789562|50805720	circRNA_021278	9:98602367|98648601
circRNA_010330	12:24242434|24244035	circRNA_013252	6:85054748|85065591	circRNA_008299	1:21206251|21222409	circRNA_002184	9:11302178|11348456
circRNA_017957	8:69709859|69711460	circRNA_008121	9:62074009|62084854	circRNA_002270	9:101476729|101492888	circRNA_006218	5:70647420|70693708
circRNA_014226	13:167103118|167104720	circRNA_009379	3:83765306|83776152	circRNA_020177	4:83670194|83686353	circRNA_018402	1:14083797|14130086
circRNA_014500	7:73125807|73127409	circRNA_003532	15:61777761|61788609	circRNA_019216	8:78083303|78099468	circRNA_010027	9:143299432|143345739
circRNA_008653	12:24242431|24244035	circRNA_011791	4:121321317|121332168	circRNA_003761	17:32994293|33010459	circRNA_016869	14:113263792|113310103
circRNA_015464	12:55278702|55280306	circRNA_001474	3:101068367|101079220	circRNA_018955	2:156366504|156382672	circRNA_008169	9:129678049|129724374
circRNA_013471	9:81814473|81816078	circRNA_019796	7:43204085|43214938	circRNA_009724	7:14106190|14122364	circRNA_010185	1:63702370|63748701
circRNA_007870	6:132259508|132261114	circRNA_016412	8:143239037|143249892	circRNA_001985	7:65664182|65680360	circRNA_004841	7:109722597|109768951
circRNA_016372	8:69709859|69711465	circRNA_002386	JH118626.1:27964|38821	circRNA_015225	1:180721255|180737438	circRNA_011636	2:133350705|133397081
circRNA_001251	2:58635553|58637160	circRNA_018583	11:35123337|35134195	circRNA_005787	14:59632979|59649165	circRNA_012398	10:75648814|75695212
circRNA_006567	X:56868918|56870525	circRNA_012354	10:33046923|33057782	circRNA_020778	16:24967931|24984117	circRNA_016754	12:17826560|17872966
circRNA_009630	6:44511352|44512959	circRNA_016217	6:64546727|64557586	circRNA_015613	14:52842304|52858491	circRNA_017688	15:117690764|117737204
circRNA_013734	9:49634791|49636399	circRNA_007739	5:38471187|38482048	circRNA_008661	12:27608857|27625046	circRNA_014562	9:108475048|108521489
circRNA_021305	GL893095.2:34178|35786	circRNA_002695	1:230999159|231010023	circRNA_013231	6:39403334|39419523	circRNA_008021	8:73399062|73445514

circRNA_011070	1:300377341|300378950	circRNA_019180	7:91955977|91966849	circRNA_008401	1:176170427|176186618	circRNA_018673	13:155618482|155664955
circRNA_003595	15:106423132|106424743	circRNA_003653	15:147704190|147715063	circRNA_002090	8:58682018|58698211	circRNA_014081	10:62692304|62738784
circRNA_020185	4:118778275|118779886	circRNA_009451	4:45545828|45556702	circRNA_016077	4:70355456|70371656	circRNA_015976	3:58975400|59021942
circRNA_002881	11:8770514|8772126	circRNA_001769	5:104202017|104212893	circRNA_021268	9:63116301|63132504	circRNA_019186	7:101881462|101928005
circRNA_015469	12:62746153|62747766	circRNA_018594	12:25811579|25822455	circRNA_002560	1:119456367|119472571	circRNA_003005	12:37228113|37274657
circRNA_000978	15:104091311|104092926	circRNA_015719	15:111538635|111549512	circRNA_014961	1:103173037|103189241	circRNA_006459	9:23115633|23162184
circRNA_005280	GL896504.1:29972|31588	circRNA_009655	6:81570022|81580900	circRNA_020234	6:111972100|111988305	circRNA_009895	8:131059540|131106102
circRNA_006125	4:16777068|16778684	circRNA_018945	2:137124137|137135015	circRNA_001507	3:119574324|119590533	circRNA_001155	18:11088489|11135070
circRNA_019087	5:106543058|106544674	circRNA_011432	15:111071683|111082562	circRNA_012512	13:20785157|20801366	circRNA_017775	2:144483954|144530535
circRNA_006369	7:120856218|120857836	circRNA_020149	3:90179967|90190849	circRNA_010398	13:145987809|146004021	circRNA_014484	6:132127016|132173603
circRNA_007811	6:43367979|43369597	circRNA_005514	1:269120143|269131026	circRNA_002291	9:129708161|129724374	circRNA_017160	6:10299594|10346209
circRNA_008572	11:114927|116545	circRNA_015003	15:61718934|61729817	circRNA_004284	4:67947307|67963521	circRNA_002897	11:19805267|19851901
circRNA_006202	5:46901571|46903191	circRNA_003126	13:55544176|55555064	circRNA_001526	3:128253444|128269660	circRNA_014579	GL893006.1:10357|57002
circRNA_010170	1:32080657|32082277	circRNA_006535	GL895423.1:3844|14733	circRNA_016148	5:51000714|51016933	circRNA_018718	14:35979092|36025785
circRNA_013117	4:91312147|91313767	circRNA_012100	GL892486.1:4394|15283	circRNA_015289	1:256829848|256846070	circRNA_004165	3:107539747|107586477
circRNA_014022	6:145705905|145707525	circRNA_014155	10:37120502|37131391	circRNA_011178	12:39209207|39225437	circRNA_008994	15:36536596|36583358
circRNA_000619	13:90962314|90963935	circRNA_009999	9:105494102|105504992	circRNA_010068	GL894897.1:77056|93287	circRNA_015188	1:97331478|97378276
circRNA_002426	X:70638360|70639982	circRNA_020369	1:23232804|23243695	circRNA_017731	18:9562088|9578319	circRNA_000019	1:17087947|17134778
circRNA_004022	2:139012954|139014576	circRNA_000953	15:85315348|85326240	circRNA_005258	GL894943.1:12044|28276	circRNA_014687	2:121986386|122033234
circRNA_004808	7:80517394|80519017	circRNA_008145	9:101227171|101238063	circRNA_008411	1:181145447|181161684	circRNA_001487	3:107539626|107586477
circRNA_019355	X:131493485|131495109	circRNA_016115	4:137107382|137118276	circRNA_010047	GL892789.1:78327|94569	circRNA_002869	11:466843|513694
circRNA_009080	16:23421971|23423596	circRNA_008066	8:143287249|143298146	circRNA_010742	6:52482329|52498571	circRNA_015630	14:68165377|68212247
circRNA_011872	6:48546856|48548481	circRNA_015236	1:200800827|200811725	circRNA_000649	13:130356500|130372748	circRNA_016275	7:18129930|18176836
circRNA_012424	11:12763669|12765294	circRNA_018875	18:36567649|36578547	circRNA_000494	12:44802030|44818283	circRNA_017053	2:148139473|148186397
circRNA_010218	1:179188515|179190143	circRNA_001201	18:59685146|59696045	circRNA_006522	GL893560.1:9170|25425	circRNA_015019	18:36505709|36552661
circRNA_011285	13:202652521|202654149	circRNA_005047	9:8935966|8946866	circRNA_000199	1:218683998|218700255	circRNA_007391	18:26163899|26210863
circRNA_015233	1:195112533|195114161	circRNA_004779	7:62835978|62846879	circRNA_008409	1:181051819|181068076	circRNA_019117	6:99489396|99536373
circRNA_005179	9:132682311|132683940	circRNA_012303	1:230929236|230940137	circRNA_006357	7:98966300|98982558	circRNA_018293	5:91211194|91258197
circRNA_020779	16:27088904|27090533	circRNA_005855	15:104127158|104138064	circRNA_001851	6:92163364|92179625	circRNA_004920	8:50518546|50565554
circRNA_007469	2:80454230|80455860	circRNA_008052	8:123634095|123645002	circRNA_012565	13:90934181|90950445	circRNA_014224	13:159929266|159976282
circRNA_014071	15:137967576|137969206	circRNA_004894	8:31876392|31887300	circRNA_008000	8:39732043|39748311	circRNA_013565	X:14612680|14659709
circRNA_019375	1:128410321|128411951	circRNA_011740	3:137588757|137599665	circRNA_015617	14:58596411|58612679	circRNA_017847	4:117080615|117127644
circRNA_015684	15:19438164|19439795	circRNA_000493	12:44782366|44793275	circRNA_011955	7:73057336|73073609	circRNA_019338	X:43235344|43282373
circRNA_018274	4:119561275|119562906	circRNA_018211	18:52285386|52296298	circRNA_013555	JH118963.1:42907|59182	circRNA_012787	15:155226057|155273098
circRNA_007572	3:90576383|90578016	circRNA_002968	12:16030982|16041895	circRNA_000161	1:180982417|180998696	circRNA_000999	15:117863548|117910594
circRNA_014537	8:123643369|123645002	circRNA_014228	13:186799180|186810093	circRNA_004280	4:56568602|56584883	circRNA_015355	10:50877061|50924109
circRNA_017091	3:133944844|133946477	circRNA_009963	9:46271320|46282234	circRNA_005040	9:364340|380624	circRNA_011920	6:156274394|156321453
circRNA_017320	9:37874005|37875638	circRNA_012394	10:63270507|63281422	circRNA_006428	8:112042572|112058856	circRNA_002199	9:19264985|19312056
circRNA_017845	4:108991730|108993363	circRNA_016268	7:10297937|10308853	circRNA_021194	8:40327326|40343610	circRNA_020365	1:16080529|16127640
circRNA_010280	10:36282980|36284614	circRNA_018207	18:27477887|27488803	circRNA_008110	9:44920845|44937132	circRNA_001606	4:90657864|90704987
circRNA_010691	5:8926856|8928490	circRNA_004318	4:98044696|98055613	circRNA_019844	9:29689315|29705606	circRNA_018876	18:36567649|36614810
circRNA_017676	15:89607067|89608701	circRNA_018358	8:140529111|140540031	circRNA_015008	15:157407747|157424041	circRNA_002784	10:21720567|21767729
circRNA_007943	7:97618884|97620519	circRNA_005380	X:121815226|121826148	circRNA_006562	X:44667590|44683888	circRNA_004624	6:99212305|99259476
circRNA_013299	7:1739806|1741441	circRNA_014510	7:105823028|105833951	circRNA_003151	13:82883346|82899646	circRNA_016258	6:145355398|145402576
circRNA_006733	1:200298028|200299664	circRNA_015639	14:78009211|78020135	circRNA_011140	11:22574569|22590869	circRNA_016542	GL895308.2:193805|240987
circRNA_007536	3:18698106|18699742	circRNA_017274	8:48428156|48439081	circRNA_014285	15:74266319|74282623	circRNA_014361	2:52869263|52916460
circRNA_009857	8:73381003|73382639	circRNA_011528	17:55271174|55282100	circRNA_015801	17:59429157|59445461	circRNA_018108	10:48033677|48080891
circRNA_013934	6:50816284|50817920	circRNA_016167	5:82756597|82767524	circRNA_016707	1:304806907|304823214	circRNA_020650	13:167100656|167147884
circRNA_014281	15:67012075|67013711	circRNA_000123	1:134656768|134667697	circRNA_020560	12:40270485|40286799	circRNA_003708	16:47778049|47825304
circRNA_003695	16:38265568|38267205	circRNA_018947	2:140344565|140355494	circRNA_019116	6:99439123|99455441	circRNA_017453	1:127648623|127695880
circRNA_006714	1:170767319|170768956	circRNA_001675	5:3470325|3481258	circRNA_009713	6:148401539|148417858	circRNA_003762	17:33077647|33124910
circRNA_012713	15:51596415|51598055	circRNA_016960	17:37038769|37049702	circRNA_006138	4:40343834|40360156	circRNA_019488	13:87162721|87209984

circRNA_005009	8:133765047|133766690	circRNA_015755	16:29438836|29449771	circRNA_016404	8:131198862|131215185	circRNA_011120	10:58751706|58798980
circRNA_009389	3:102653727|102655370	circRNA_003779	17:45374637|45385573	circRNA_008584	11:13221205|13237532	circRNA_019021	4:34570313|34617596
circRNA_000907	15:51596415|51598059	circRNA_005606	11:76688002|76698938	circRNA_016446	9:37673213|37689540	circRNA_013154	5:24694618|24741903
circRNA_019558	14:144106612|144108257	circRNA_008073	9:250376|261322	circRNA_002503	1:59728051|59744379	circRNA_003003	12:37227369|37274657
circRNA_016995	18:59920080|59921726	circRNA_008756	13:55492221|55503168	circRNA_018898	2:16918297|16934633	circRNA_003864	18:53737307|53784629
circRNA_016678	1:208530596|208532243	circRNA_018857	17:57168414|57179361	circRNA_007624	4:16778493|16794831	circRNA_012827	17:3451680|3499007
circRNA_002826	10:47266666|47268314	circRNA_021071	5:88016428|88027375	circRNA_013805	12:51831916|51848255	circRNA_007802	6:32195144|32242475
circRNA_005383	X:121926744|121928392	circRNA_009430	4:15227218|15238168	circRNA_015348	10:45936930|45953269	circRNA_016251	6:108959030|109006369
circRNA_010092	JH118951.1:27732|29381	circRNA_010798	7:69682421|69693371	circRNA_005679	13:72865581|72881923	circRNA_006753	1:225790958|225838303
circRNA_011348	14:83710581|83712230	circRNA_008811	13:117695880|117706831	circRNA_011303	14:10462923|10479267	circRNA_004542	6:32177486|32224859
circRNA_014900	X:90715380|90717029	circRNA_013095	4:45254094|45265047	circRNA_008396	1:161765502|161781848	circRNA_010641	3:122008499|122055914
circRNA_013218	6:13097978|13099628	circRNA_014006	14:50023889|50034842	circRNA_002444	X:127709214|127725565	circRNA_008533	10:37402533|37449960
circRNA_009043	15:112464250|112465901	circRNA_001783	6:16298300|16309258	circRNA_015210	1:133418897|133435248	circRNA_001326	2:119165841|119213291
circRNA_006263	6:50391945|50393597	circRNA_015417	12:11474767|11485727	circRNA_004352	4:122750450|122766803	circRNA_015680	15:9456353|9503812
circRNA_003344	14:42894015|42895668	circRNA_016195	6:16298296|16309258	circRNA_003130	13:58781354|58797708	circRNA_011928	7:17964098|18011584
circRNA_018689	13:202614762|202616415	circRNA_015519	13:81424354|81435317	circRNA_013657	1:133418894|133435248	circRNA_006856	10:77576677|77624209
circRNA_011154	11:84395032|84396686	circRNA_000807	14:77652076|77663041	circRNA_004475	5:79173601|79189959	circRNA_015408	11:76193953|76241487
circRNA_018999	3:107151144|107152798	circRNA_019622	2:43632613|43643579	circRNA_002789	10:29321805|29338167	circRNA_003538	15:66886362|66933912
circRNA_012586	13:144152728|144154384	circRNA_013490	9:117829885|117840855	circRNA_010579	2:91214492|91230855	circRNA_016722	10:30386471|30434042
circRNA_014270	14:148062722|148064378	circRNA_007488	2:91206107|91217079	circRNA_012780	15:140024509|140040872	circRNA_002257	9:82271887|82319494
circRNA_017584	13:23393002|23394658	circRNA_000174	1:201664903|201675876	circRNA_020873	2:52235687|52252052	circRNA_007471	2:82153520|82201141
circRNA_004334	4:107943047|107944705	circRNA_020497	10:17402178|17413151	circRNA_007206	14:145714289|145730657	circRNA_007789	6:16261615|16309258
circRNA_013072	4:15236510|15238168	circRNA_020785	16:36074651|36085624	circRNA_013487	9:116165727|116182100	circRNA_000687	13:167069887|167117540
circRNA_004119	3:81748823|81750482	circRNA_005125	9:63000517|63011493	circRNA_005391	X:127725495|127741869	circRNA_021124	6:101697609|101745288
circRNA_002474	1:18661830|18663491	circRNA_012890	18:59685146|59696123	circRNA_013488	9:116176878|116193255	circRNA_013748	13:167164236|167211925
circRNA_003091	13:29765349|29767010	circRNA_000756	14:30062323|30073303	circRNA_000798	14:68195867|68212247	circRNA_009171	18:9569859|9617560
circRNA_004929	8:57832594|57834255	circRNA_019643	2:120392795|120403776	circRNA_004435	5:46888183|46904567	circRNA_013574	X:35456706|35504448
circRNA_018451	1:142578061|142579723	circRNA_005197	9:140018765|140029754	circRNA_021143	6:155089542|155105930	circRNA_000360	10:71835595|71883350
circRNA_017081	3:104116267|104117930	circRNA_003105	13:37602449|37613439	circRNA_003763	17:33108518|33124910	circRNA_008295	1:17075533|17123292
circRNA_008656	12:27128054|27129718	circRNA_013137	4:136401988|136412978	circRNA_012088	9:136023233|136039626	circRNA_013766	1:32054762|32102563
circRNA_015585	13:212906970|212908635	circRNA_008845	13:187763595|187774586	circRNA_018907	2:52248831|52265225	circRNA_002343	GL894261.2:43206|91044
circRNA_016051	4:16819910|16821577	circRNA_012206	1:35479001|35489992	circRNA_019113	6:91918116|91934520	circRNA_016466	9:55420796|55468658
circRNA_018971	3:42052106|42053774	circRNA_006860	11:5263855|5274850	circRNA_006608	1:17075533|17091938	circRNA_002938	11:74714006|74761877
circRNA_018000	9:89212443|89214112	circRNA_013155	5:29870633|29881628	circRNA_000691	13:167195512|167211925	circRNA_018236	2:91905341|91953227
circRNA_006473	9:40642170|40643840	circRNA_006235	5:88156032|88167029	circRNA_002976	12:17069503|17085916	circRNA_020935	3:21774890|21822807
circRNA_009257	2:70273439|70275110	circRNA_015819	18:12091537|12102535	circRNA_013211	5:105296613|105313031	circRNA_020735	15:66916145|66964066
circRNA_016527	GL894396.1:2308|3981	circRNA_000759	14:31361690|31372689	circRNA_009640	6:64478265|64494687	circRNA_007180	14:121761204|121809144
circRNA_019343	X:67444249|67445922	circRNA_007213	15:18325856|18336856	circRNA_004843	7:109976001|109992425	circRNA_004446	5:56916166|56964141
circRNA_006537	GL896152.2:40782|42458	circRNA_000424	11:56292568|56303570	circRNA_011608	2:83895486|83911912	circRNA_005547	10:29567935|29615958
circRNA_009872	8:93306923|93308599	circRNA_004524	6:12077068|12088070	circRNA_021359	X:37925332|37941759	circRNA_020438	1:183362402|183410425
circRNA_017025	2:83014283|83015959	circRNA_005068	9:18937518|18948522	circRNA_015725	15:117562197|117578630	circRNA_019553	14:124068568|124116596
circRNA_004538	6:26048730|26050407	circRNA_002835	10:54125708|54136715	circRNA_016417	8:143484196|143500629	circRNA_004612	6:92078907|92126940
circRNA_013584	X:67444249|67445926	circRNA_003279	13:212937526|212948534	circRNA_011698	3:76466177|76482619	circRNA_014637	13:74688185|74736227
circRNA_017894	6:83159243|83160920	circRNA_007131	14:58594277|58605289	circRNA_013106	4:79564780|79581223	circRNA_019854	9:119281410|119329461
circRNA_019460	12:37179801|37181479	circRNA_000142	1:144729933|144740947	circRNA_007283	15:122942696|122959141	circRNA_016854	14:68452039|68500095
circRNA_010447	14:113550575|113552254	circRNA_003272	13:211120471|211131487	circRNA_017889	6:76019958|76036406	circRNA_002488	1:30684925|30732984
circRNA_001964	7:43350888|43352570	circRNA_007541	3:29618701|29629718	circRNA_000073	1:93126380|93142829	circRNA_009575	5:79818808|79866896
circRNA_016733	11:8770444|8772126	circRNA_011334	14:62469263|62480280	circRNA_000264	1:300126112|300142568	circRNA_020313	9:36667204|36715305
circRNA_018749	14:95251657|95253340	circRNA_017430	1:56918248|56929266	circRNA_006135	4:39819040|39835499	circRNA_020535	11:53943226|53991328
circRNA_001126	17:57250248|57251932	circRNA_014045	15:51611391|51622410	circRNA_000622	13:97298407|97314870	circRNA_018783	15:66313353|66361485
circRNA_010855	8:140978507|140980191	circRNA_010903	9:133538054|133549074	circRNA_002693	1:227165198|227181662	circRNA_019150	7:17537616|17585757
circRNA_007926	7:73042261|73043946	circRNA_010468	15:28540273|28551296	circRNA_019835	8:141308029|141324497	circRNA_018365	9:8935966|8984112

circRNA_010126	X:64980606|64982291	circRNA_007418	2:7629760|7640784	circRNA_000454	12:16085352|16101827	circRNA_006150	4:77589119|77637270
circRNA_017887	6:75411758|75413444	circRNA_014237	14:33212834|33223859	circRNA_003613	15:116425424|116441903	circRNA_020209	6:16261615|16309767
circRNA_018534	10:19926197|19927883	circRNA_018750	14:111708320|111719345	circRNA_004493	5:92098435|92114918	circRNA_011996	8:33447628|33495808
circRNA_010199	1:116747311|116748998	circRNA_004629	6:99525347|99536373	circRNA_001350	2:139227898|139244383	circRNA_020305	9:19263852|19312056
circRNA_019188	7:103798237|103799924	circRNA_007545	3:33359977|33371003	circRNA_019047	4:117906038|117922524	circRNA_016277	7:18474412|18522624
circRNA_002813	10:37486613|37488301	circRNA_008355	1:110527369|110538400	circRNA_010392	13:124264105|124280592	circRNA_020375	1:37382758|37430976
circRNA_015892	2:81045248|81046937	circRNA_007952	7:102425698|102436732	circRNA_004106	3:73358334|73374822	circRNA_018684	13:190918409|190966639
circRNA_005821	14:145874264|145875955	circRNA_020156	3:121673683|121684717	circRNA_005358	X:102745424|102761917	circRNA_005698	13:91200922|91249175
circRNA_018905	2:43984992|43986683	circRNA_011605	2:80451220|80462260	circRNA_012169	X:116399661|116416155	circRNA_016482	9:79899441|79947699
circRNA_014941	6:143739937|143741629	circRNA_011973	7:119769147|119780189	circRNA_009886	8:120025934|120042430	circRNA_019848	9:78959290|79007550
circRNA_004540	6:31447413|31449106	circRNA_002437	X:116341278|116352321	circRNA_006394	8:32694464|32710967	circRNA_000292	10:18101700|18149975
circRNA_011504	16:68923545|68925239	circRNA_012049	9:22399439|22410483	circRNA_007496	2:98310292|98326796	circRNA_015148	1:22086461|22134742
circRNA_006921	12:23311439|23313135	circRNA_015156	1:32082085|32093129	circRNA_008213	GL895028.1:36544|53050	circRNA_005193	9:139451974|139500268
circRNA_014886	9:46280538|46282234	circRNA_004814	7:91884279|91895327	circRNA_019065	5:37252743|37269250	circRNA_015263	1:226070210|226118520
circRNA_000145	1:145416172|145417869	circRNA_004080	3:42066184|42077233	circRNA_012811	16:47823935|47840449	circRNA_017428	1:45756711|45805070
circRNA_012131	GL895825.2:21435|23132	circRNA_017858	5:35903030|35914080	circRNA_006786	1:285642342|285658859	circRNA_015147	1:21200690|21249088
circRNA_003142	13:77865050|77866748	circRNA_015835	18:36461492|36472543	circRNA_003996	2:119213114|119229632	circRNA_001159	18:14986350|15034759
circRNA_018586	12:5569270|5570968	circRNA_013644	8:78366796|78377848	circRNA_007071	13:154567698|154584216	circRNA_014317	16:52884700|52933109
circRNA_014950	9:102929813|102931512	circRNA_005667	13:37977080|37988133	circRNA_016982	18:19933479|19949999	circRNA_006847	10:56160513|56208948
circRNA_009168	18:5561321|5563021	circRNA_007668	4:74298616|74309669	circRNA_005848	15:85340631|85357154	circRNA_015775	16:52884649|52933109
circRNA_009624	6:27509418|27511118	circRNA_008720	13:25467656|25478709	circRNA_014752	X:10411139|10427663	circRNA_013111	4:83637884|83686353
circRNA_013283	6:145359462|145361162	circRNA_014585	GL894855.1:18136|29194	circRNA_001283	2:82549217|82565744	circRNA_004847	7:117061597|117110099
circRNA_005301	MT:1398|3099	circRNA_021337	GL896405.1:30785|41850	circRNA_007044	13:126877865|126894393	circRNA_020407	1:127622340|127670842
circRNA_016206	6:39470582|39472285	circRNA_011737	3:134657095|134668164	circRNA_003023	12:44468807|44485338	circRNA_010743	6:52544925|52593444
circRNA_013345	7:100759932|100761638	circRNA_018822	16:25405911|25416980	circRNA_011844	5:92502063|92518596	circRNA_013128	4:117897403|117945925
circRNA_020511	10:50998506|51000212	circRNA_013271	6:113140338|113151408	circRNA_020860	2:12791042|12807575	circRNA_010751	6:88258181|88306712
circRNA_020621	13:128120359|128122066	circRNA_017674	15:86948961|86960031	circRNA_020467	1:249045889|249062426	circRNA_016801	13:84810799|84859332
circRNA_010748	6:77618016|77619724	circRNA_019401	1:227044102|227055172	circRNA_016859	14:77567920|77584462	circRNA_010862	9:11747252|11795797
circRNA_005018	8:141043790|141045499	circRNA_005587	11:18665770|18676841	circRNA_017670	15:85364480|85381023	circRNA_005207	9:149654250|149702797
circRNA_015190	1:101178705|101180415	circRNA_013069	4:10825873|10836946	circRNA_000867	14:142840673|142857217	circRNA_019479	13:50931553|50980104
circRNA_009870	8:93221794|93223505	circRNA_020004	13:82875609|82886686	circRNA_003030	12:45421101|45437647	circRNA_014425	4:72410011|72458585
circRNA_016780	13:33720938|33722650	circRNA_016821	13:145370141|145381220	circRNA_015788	17:5298184|5314733	circRNA_019161	7:60967186|61015769
circRNA_019236	8:141043787|141045499	circRNA_001632	4:113244529|113255612	circRNA_007482	2:87947590|87964141	circRNA_016471	9:63863814|63912406
circRNA_003957	2:87128383|87130100	circRNA_020752	15:97976325|97987408	circRNA_005563	10:54381760|54398312	circRNA_004662	6:132124985|132173603
circRNA_006068	3:39633512|39635229	circRNA_002546	1:103542032|103553116	circRNA_001448	3:76474842|76491398	circRNA_021366	X:52835294|52883964
circRNA_007782	5:106793019|106794736	circRNA_000713	13:210781573|210792658	circRNA_017741	18:42001004|42017561	circRNA_017130	4:135658543|135707221
circRNA_020270	7:100802127|100803845	circRNA_010948	1:2750752|2761837	circRNA_005142	9:85990640|86007198	circRNA_016023	3:114304286|114352969
circRNA_005989	2:76398453|76400172	circRNA_016897	15:63894587|63905675	circRNA_020665	14:4043079|4059637	circRNA_007786	6:10251024|10299712
circRNA_009629	6:41369771|41371490	circRNA_005795	14:84848647|84859738	circRNA_017205	6:141363206|141379767	circRNA_000398	11:19803203|19851901
circRNA_011440	15:120872321|120874040	circRNA_015242	1:204787077|204798169	circRNA_010411	13:172707520|172724087	circRNA_000675	13:148857550|148906255
circRNA_002367	GL895834.1:10169|11889	circRNA_015483	13:22797970|22809064	circRNA_001351	2:139496126|139512697	circRNA_020310	9:29427479|29476186
circRNA_011756	4:36916142|36917862	circRNA_008926	14:77573358|77584462	circRNA_005849	15:85384383|85400955	circRNA_006933	12:35338611|35387319
circRNA_016851	14:62522480|62524201	circRNA_009170	18:9558914|9570018	circRNA_012597	13:166282060|166298634	circRNA_009297	2:126285440|126334183
circRNA_010917	GL894608.1:15485|17207	circRNA_007726	5:16683843|16694950	circRNA_009248	2:53080124|53096699	circRNA_005574	11:464949|513694
circRNA_010952	1:14101396|14103118	circRNA_000364	10:76033749|76044859	circRNA_019305	GL892843.1:64025|80602	circRNA_015040	6:73334893|73383647
circRNA_017788	3:38764978|38766700	circRNA_012657	14:65938181|65949291	circRNA_017539	11:10321813|10338393	circRNA_013763	1:8795511|8844270
circRNA_018431	1:103214079|103215801	circRNA_000739	14:14068398|14079510	circRNA_011228	13:67530900|67547482	circRNA_005316	X:14455714|14504493
circRNA_001732	5:69943782|69945505	circRNA_012332	1:306460466|306471579	circRNA_003619	15:117863548|117880131	circRNA_017211	6:150846440|150895226
circRNA_013516	GL893190.1:4212|5937	circRNA_004144	3:92450641|92461755	circRNA_001538	3:135350241|135366829	circRNA_009422	3:134654689|134703504
circRNA_016214	6:58244799|58246524	circRNA_021332	GL896000.1:906|12021	circRNA_009195	18:42190501|42207089	circRNA_015476	13:14126215|14175033
circRNA_016309	7:80358694|80360419	circRNA_010013	9:125455145|125466266	circRNA_012484	12:37398861|37415450	circRNA_003709	16:47778049|47826868
circRNA_020481	1:283089950|283091675	circRNA_011126	10:59663316|59674438	circRNA_003078	13:23704328|23720920	circRNA_001933	6:153309526|153358353

circRNA_011673	3:41493305|41495031	circRNA_009404	3:118544288|118555411	circRNA_005847	15:85074728|85091324	circRNA_008249	X:44568029|44616861
circRNA_013466	9:72569978|72571704	circRNA_010980	1:96782128|96793252	circRNA_003776	17:43615013|43631612	circRNA_009183	18:15002914|15051751
circRNA_011263	13:142627386|142629113	circRNA_009653	6:81212689|81223815	circRNA_016564	JH118701.1:16917|33520	circRNA_020987	3:121345252|121394097
circRNA_014152	10:31870846|31872573	circRNA_009207	18:59324466|59335593	circRNA_004116	3:81716210|81732824	circRNA_009921	9:19251279|19300140
circRNA_021067	5:86842699|86844426	circRNA_015702	15:67049233|67060363	circRNA_001158	18:14986350|15002971	circRNA_015465	12:59331765|59380626
circRNA_011909	6:137802513|137804242	circRNA_001980	7:62801435|62812567	circRNA_004822	7:94509306|94525927	circRNA_001181	18:31018819|31067701
circRNA_010344	12:47450863|47452593	circRNA_016101	4:116731304|116742436	circRNA_006637	1:60908344|60924967	circRNA_003574	15:86622619|86671532
circRNA_003952	2:86248439|86250170	circRNA_012151	X:17739090|17750223	circRNA_003001	12:37146039|37162666	circRNA_020979	3:103798859|103847787
circRNA_007215	15:18583913|18585645	circRNA_021014	4:55214192|55225326	circRNA_008740	13:38271667|38288294	circRNA_007414	18:59639958|59688893
circRNA_007458	2:71200925|71202659	circRNA_016761	12:37135028|37146163	circRNA_008594	11:21089411|21106042	circRNA_020392	1:84560283|84609236
circRNA_019112	6:88638325|88640059	circRNA_008640	12:14679564|14690700	circRNA_016898	15:66037290|66053922	circRNA_010540	18:15020215|15069170
circRNA_009523	5:16400508|16402243	circRNA_003240	13:167069887|167081024	circRNA_004768	7:57270687|57287321	circRNA_000012	1:15907346|15956308
circRNA_017844	4:108045619|108047354	circRNA_006413	8:78481950|78493089	circRNA_006094	3:104183075|104199715	circRNA_011278	13:160239598|160288577
circRNA_001545	4:15260718|15262454	circRNA_017290	8:117196718|117207859	circRNA_006365	7:116904577|116921217	circRNA_015077	10:43878947|43927930
circRNA_015087	14:60226981|60228717	circRNA_004084	3:45602185|45613329	circRNA_013436	9:11779155|11795797	circRNA_005351	X:77194121|77243106
circRNA_017506	1:299797832|299799568	circRNA_000239	1:270442985|270454132	circRNA_021196	8:40572430|40589072	circRNA_007666	4:73577490|73626478
circRNA_006544	JH118622.1:6975|8712	circRNA_013850	15:97032573|97043722	circRNA_018560	10:77690752|77707396	circRNA_009721	7:57008|105997
circRNA_010824	8:19208010|19209747	circRNA_019763	6:88268086|88279235	circRNA_010167	1:30710564|30727210	circRNA_004817	7:91917856|91966849
circRNA_016639	1:103509407|103511144	circRNA_019989	13:7747417|7758566	circRNA_011837	5:87157448|87174095	circRNA_021171	7:91890126|91939138
circRNA_001627	4:108045619|108047358	circRNA_015191	1:101205442|101216594	circRNA_005265	GL895413.1:26842|43491	circRNA_021112	6:86818335|86867375
circRNA_007427	2:17234650|17236390	circRNA_008022	8:74346358|74357512	circRNA_018415	1:56093263|56109914	circRNA_006109	3:114326822|114375867
circRNA_013318	7:52135012|52136752	circRNA_008923	14:71904773|71915927	circRNA_005766	14:14656977|14673631	circRNA_014209	13:90903716|90952792
circRNA_013984	1:113957632|113959372	circRNA_016727	10:58742098|58753252	circRNA_004397	5:3454359|3471014	circRNA_008629	12:358832|407916
circRNA_006801	10:177153|178895	circRNA_013645	9:44742751|44753907	circRNA_014030	9:9531641|9548296	circRNA_003573	15:86622429|86671532
circRNA_006907	12:6449639|6451382	circRNA_008772	13:75597790|75608947	circRNA_015600	14:31736566|31753221	circRNA_013721	5:37004818|37053948
circRNA_013247	6:78476142|78477885	circRNA_015492	13:35647684|35658848	circRNA_012149	X:14294763|14311425	circRNA_004093	3:58116769|58165903
circRNA_014505	7:94524184|94525927	circRNA_008796	13:97366509|97377675	circRNA_021237	8:143258710|143275381	circRNA_009714	6:148405581|148454719
circRNA_001806	6:57230731|57232475	circRNA_006425	8:102882998|102894165	circRNA_008723	13:26014191|26030866	circRNA_018328	7:12814018|12863160
circRNA_009124	16:78203963|78205707	circRNA_015693	15:61353356|61364525	circRNA_017580	13:7975063|7991744	circRNA_017204	6:137755080|137804242
circRNA_009001	15:53153877|53155622	circRNA_000597	13:73329729|73340904	circRNA_010442	14:78009691|78026373	circRNA_001225	2:17721874|17771066
circRNA_000445	12:12135663|12137409	circRNA_010272	10:19595506|19606682	circRNA_001525	3:126269305|126285991	circRNA_003547	15:75644402|75693603
circRNA_012593	13:156525512|156527258	circRNA_014202	13:56459055|56470231	circRNA_015633	14:77060624|77077310	circRNA_014966	1:225691245|225740450
circRNA_011877	6:69352429|69354176	circRNA_002666	1:202644306|202655484	circRNA_011080	10:14913686|14930373	circRNA_014535	8:118450593|118499802
circRNA_013735	9:50831548|50833295	circRNA_011090	10:28516698|28527877	circRNA_017050	2:142518692|142535381	circRNA_003555	15:85315348|85364566
circRNA_013123	4:116719316|116721064	circRNA_009692	6:125764845|125776025	circRNA_002286	9:127761598|127778289	circRNA_016804	13:91211618|91260862
circRNA_016882	14:141164858|141166607	circRNA_010492	15:115158384|115169564	circRNA_003910	2:48859588|48876280	circRNA_002020	7:97294164|97343426
circRNA_003293	14:9504665|9506415	circRNA_014422	4:70410831|70422011	circRNA_019770	6:132138607|132155299	circRNA_017491	1:230890866|230940137
circRNA_004873	8:1522764|1524515	circRNA_008751	13:49869090|49880272	circRNA_002874	11:5263855|5280553	circRNA_000236	1:270188319|270237597
circRNA_004530	6:14474409|14476162	circRNA_004945	8:73375718|73386901	circRNA_004877	8:9011621|9028319	circRNA_010130	X:70426183|70475483
circRNA_013616	8:82557066|82558820	circRNA_003287	14:3194644|3205830	circRNA_014755	X:68449374|68466076	circRNA_011148	11:74618777|74668079
circRNA_000053	1:50514833|50516588	circRNA_011351	14:96756176|96767362	circRNA_001671	5:200465|217179	circRNA_002734	1:270188281|270237597
circRNA_000996	15:117531248|117533003	circRNA_005628	12:40259565|40270754	circRNA_008933	14:82320897|82337611	circRNA_007684	4:113244529|113293869
circRNA_017006	2:12965934|12967689	circRNA_003401	14:82403576|82414766	circRNA_004710	6:156189695|156206411	circRNA_013618	1:58468249|58517592
circRNA_000050	1:50514830|50516588	circRNA_006869	11:19712163|19723353	circRNA_008449	1:229828505|229845228	circRNA_019141	6:153309526|153358915
circRNA_002862	10:76033749|76035507	circRNA_006987	13:36937742|36948936	circRNA_018973	3:45760384|45777111	circRNA_002204	9:23112748|23162184
circRNA_011624	2:108990313|108992071	circRNA_003676	16:24983954|24995151	circRNA_004575	6:78476142|78492871	circRNA_013589	X:109580418|109629854
circRNA_010010	9:120377550|120379309	circRNA_005672	13:44062045|44073250	circRNA_006810	10:16939299|16956030	circRNA_010944	X:109580414|109629854
circRNA_016278	7:23933083|23934842	circRNA_009725	7:14136139|14147345	circRNA_006581	X:109068407|109085139	circRNA_013679	13:50969982|51019448
circRNA_006854	10:71317683|71319443	circRNA_002895	11:19715398|19726605	circRNA_008422	1:200281377|200298109	circRNA_018464	1:176185364|176234880
circRNA_013833	14:24811227|24812988	circRNA_015676	15:17725|28932	circRNA_007018	13:90496250|90512987	circRNA_001966	7:47785227|47834772
circRNA_014576	GL892628.1:24048|25809	circRNA_020279	8:18153753|18164960	circRNA_005436	1:103209533|103226272	circRNA_007253	15:79683656|79733212
circRNA_010163	X:144147876|144149638	circRNA_014824	3:11496127|11507336	circRNA_009967	9:50817859|50834603	circRNA_019943	1:256829848|256879435

circRNA_014382	3:19043905|19045667	circRNA_020866	2:43662771|43673981	circRNA_011474	16:27092137|27108891	circRNA_003179	13:98113084|98162682
circRNA_015456	12:46869425|46871187	circRNA_000408	11:25548977|25560191	circRNA_018420	1:62694950|62711704	circRNA_004921	8:50518546|50568148
circRNA_007456	2:70959436|70961199	circRNA_015088	17:5330457|5341673	circRNA_006908	12:11466494|11483250	circRNA_000368	10:77572815|77622440
circRNA_021127	6:112391424|112393187	circRNA_015661	14:117639860|117651080	circRNA_017714	16:55475544|55492301	circRNA_013802	12:38622947|38672576
circRNA_011435	15:112522894|112524658	circRNA_019821	8:57800743|57811963	circRNA_019281	9:101235349|101252107	circRNA_009602	5:109492237|109541874
circRNA_017467	1:161056362|161058128	circRNA_002349	GL894485.1:10252|21473	circRNA_016628	1:49655991|49672750	circRNA_006611	1:18675426|18725069
circRNA_012689	14:122899944|122901711	circRNA_002187	9:11337234|11348456	circRNA_009974	9:58415508|58432271	circRNA_016344	8:20657917|20707578
circRNA_011205	13:30860472|30862241	circRNA_005780	14:42341566|42352788	circRNA_003388	14:78234225|78250994	circRNA_018412	1:45737787|45787500
circRNA_002039	7:121179727|121181497	circRNA_006568	X:61016381|61027603	circRNA_011349	14:95061065|95077834	circRNA_019920	1:97325990|97375710
circRNA_006905	12:4804161|4805931	circRNA_012249	1:126092089|126103317	circRNA_002068	8:29293951|29310723	circRNA_019167	7:65664182|65713909
circRNA_006280	6:85042122|85043893	circRNA_005378	X:121808444|121819674	circRNA_003975	2:93851810|93868582	circRNA_009986	9:78997745|79047485
circRNA_012452	12:162772|164543	circRNA_001764	5:93584635|93595868	circRNA_015005	15:73557013|73573786	circRNA_009136	17:24523664|24573448
circRNA_007586	3:104197942|104199715	circRNA_004971	8:89353525|89364758	circRNA_011064	1:278506590|278523369	circRNA_014627	11:19108869|19158675
circRNA_013079	4:20119669|20121443	circRNA_010852	8:125017791|125029024	circRNA_020140	3:55783850|55800633	circRNA_019647	2:130883647|130933457
circRNA_016156	5:65307976|65309751	circRNA_000936	15:73328068|73339303	circRNA_016281	7:42438417|42455203	circRNA_013395	8:82980802|83030613
circRNA_018844	17:28241701|28243476	circRNA_006315	6:154541185|154552424	circRNA_001758	5:88153461|88170249	circRNA_007387	18:18602505|18652345
circRNA_001517	3:122048175|122049951	circRNA_014182	12:40668549|40679789	circRNA_008174	9:138536002|138552791	circRNA_008863	13:210819581|210869426
circRNA_007951	7:102242791|102244568	circRNA_019457	12:14684074|14695314	circRNA_005608	11:86869925|86886717	circRNA_001792	6:35124281|35174142
circRNA_009465	4:70410831|70412608	circRNA_007197	14:140924796|140936039	circRNA_008938	14:95061035|95077834	circRNA_007319	16:43232224|43282123
circRNA_013274	6:125774248|125776025	circRNA_017075	3:90690141|90701391	circRNA_004131	3:84814587|84831391	circRNA_000156	1:169424632|169474563
circRNA_007594	3:109726110|109727889	circRNA_019962	10:48598735|48609985	circRNA_007991	8:31730675|31747487	circRNA_009498	4:129286788|129336726
circRNA_005969	2:17755529|17757310	circRNA_016518	GL893784.1:6605|17856	circRNA_000420	11:53974514|53991328	circRNA_003620	15:117863548|117913489
circRNA_010449	14:114567530|114569311	circRNA_020098	18:25797586|25808837	circRNA_006387	8:29185746|29202562	circRNA_021184	7:125239034|125289007
circRNA_006116	3:125921425|125923207	circRNA_011445	15:122873048|122879826	circRNA_018221	2:32306523|32323341	circRNA_000215	1:241875289|241925272
circRNA_009696	6:132167543|132169325	circRNA_008220	GL896209.1:14829|21612	circRNA_010137	X:70818283|70835105	circRNA_004289	4:72746838|72796823
circRNA_012851	17:61297296|61299078	circRNA_019392	1:202448313|202455106	circRNA_008769	13:75148128|75164951	circRNA_002475	1:21187807|21237803
circRNA_015785	16:78142000|78143787	circRNA_004826	7:97294164|97300958	circRNA_003444	14:122297273|122314098	circRNA_006679	1:127604961|127654960
circRNA_002364	GL895707.1:5758|7546	circRNA_018694	13:210785863|210792658	circRNA_002263	9:90282096|90298922	circRNA_011627	2:113641401|113691406
circRNA_004120	3:81815281|81817069	circRNA_005084	9:26047339|26054137	circRNA_007431	2:30709277|30726103	circRNA_007672	4:90623622|90673688
circRNA_003042	12:50480103|50481892	circRNA_007876	6:145705905|145712703	circRNA_004126	3:84274641|84291468	circRNA_001747	5:76330931|76381045
circRNA_016475	9:78650899|78652688	circRNA_011483	16:36595410|36602208	circRNA_001908	6:137844860|137861689	circRNA_011053	1:250313006|250363168
circRNA_006201	5:44957305|44959096	circRNA_014784	13:97274219|97281017	circRNA_014800	15:79283986|79300817	circRNA_007312	16:36742926|36793124
circRNA_015074	1:242716644|242718436	circRNA_001077	16:78165123|78171923	circRNA_005635	12:52254456|52271288	circRNA_011554	18:45536718|45586937
circRNA_011589	2:50771335|50773128	circRNA_014582	GL893884.1:31770|38570	circRNA_016169	5:86229845|86246678	circRNA_013346	7:101076419|101126663
circRNA_000634	13:108413402|108415199	circRNA_002577	1:132315007|132321809	circRNA_013799	11:15976293|15993129	circRNA_012612	13:212950253|213000535
circRNA_009628	6:39451629|39453427	circRNA_010445	14:99899814|99906616	circRNA_013993	1:299933010|299949846	circRNA_001495	3:113380479|113430806
circRNA_013969	GL893077.1:7680|9479	circRNA_015591	14:9434993|9441795	circRNA_004483	5:86229841|86246678	circRNA_014067	1:17072954|17123292
circRNA_017635	14:67517948|67519747	circRNA_019819	8:40472433|40479235	circRNA_004258	4:40283338|40300180	circRNA_003471	14:142803973|142854338
circRNA_006577	X:101666930|101668730	circRNA_013602	1:132962462|132969266	circRNA_004953	8:78476244|78493089	circRNA_000208	1:227644106|227694491
circRNA_004573	6:75060283|75062084	circRNA_000806	14:77596507|77603313	circRNA_012234	1:103929815|103946660	circRNA_019528	14:55819182|55869595
circRNA_015620	14:60127644|60129445	circRNA_004823	7:94882187|94888995	circRNA_018188	15:117886587|117903433	circRNA_008557	10:56267463|56317956
circRNA_002951	12:5475167|5476969	circRNA_005512	1:251428283|251435091	circRNA_014001	13:190949788|190966639	circRNA_012349	10:18199660|18250156
circRNA_003824	18:16346291|16348093	circRNA_008347	1:103254748|103261556	circRNA_001002	15:118280880|118297732	circRNA_019176	7:91900007|91950519
circRNA_006226	5:83975898|83977701	circRNA_002464	1:16112729|16119538	circRNA_005481	1:202012272|202029125	circRNA_020367	1:16340188|16390764
circRNA_007125	14:51721706|51723510	circRNA_006820	10:29339600|29346409	circRNA_011235	13:85435308|85452163	circRNA_004716	7:12247873|12298480
circRNA_003933	2:80458726|80460531	circRNA_013927	5:79946906|79953716	circRNA_016769	12:48633920|48650775	circRNA_018500	1:230871275|230921889
circRNA_007157	14:85796577|85798382	circRNA_005338	X:61016381|61023192	circRNA_002849	10:61727666|61744522	circRNA_017622	14:10612603|10663285
circRNA_013103	4:73759207|73761012	circRNA_006560	X:40486596|40493407	circRNA_009572	5:76314134|76330992	circRNA_020161	4:20378229|20428914
circRNA_002390	MT:1293|3099	circRNA_007028	13:91219961|91226772	circRNA_001901	6:128736988|128753848	circRNA_016062	4:41064761|41115480
circRNA_021327	GL895293.1:46865|48671	circRNA_012198	1:31387491|31394302	circRNA_006004	2:86275127|86291987	circRNA_013764	1:17075533|17126273
circRNA_006350	7:83017250|83019059	circRNA_010603	2:157388513|157395325	circRNA_009266	2:86275122|86291987	circRNA_006176	4:113204856|113255612
circRNA_002005	7:86337175|86338985	circRNA_012828	17:12529639|12536452	circRNA_020291	8:78476244|78493111	circRNA_015143	1:16870879|16921652

circRNA_011551	18:42042599|42044409	circRNA_006767	1:263638779|263645595	circRNA_011543	18:15081212|15098086	circRNA_014532	8:110762091|110812866
circRNA_013999	12:62443557|62445367	circRNA_004454	5:70372892|70379709	circRNA_007983	8:9011621|9028499	circRNA_015477	13:15498867|15549655
circRNA_019716	5:6774342|6776152	circRNA_008695	12:62446432|62453250	circRNA_021012	4:48139391|48156272	circRNA_012970	2:130933380|130984187
circRNA_002394	MT:1551|3362	circRNA_016029	3:120579693|120586512	circRNA_001581	4:55318260|55335142	circRNA_010578	2:89007457|89058265
circRNA_003437	14:120545819|120547630	circRNA_009018	15:67043433|67050253	circRNA_020944	3:33326822|33343705	circRNA_001019	15:131633789|131684607
circRNA_011183	12:49750004|49751815	circRNA_018379	GL892269.1:38712|45532	circRNA_014988	13:167100656|167117540	circRNA_018091	1:202935303|202986161
circRNA_003608	15:115291793|115293605	circRNA_003187	13:103725786|103732607	circRNA_020319	9:46857944|46874828	circRNA_017490	1:225740193|225791062
circRNA_012236	1:110560015|110561827	circRNA_016284	7:43185658|43192480	circRNA_013682	13:79310756|79327641	circRNA_019792	7:38858509|38909427
circRNA_007448	2:60627746|60629559	circRNA_014208	13:90760724|90767547	circRNA_014342	18:24405599|24422485	circRNA_012785	15:147145354|147196279
circRNA_013776	1:181157215|181159028	circRNA_015075	10:9667997|9674820	circRNA_017344	9:100409103|100425990	circRNA_009989	9:88630066|88681009
circRNA_015130	6:134072885|134074698	circRNA_009633	6:50766988|50773813	circRNA_000049	1:49655861|49672750	circRNA_003748	17:13616684|13667649
circRNA_016640	1:105950020|105951833	circRNA_001823	6:73679331|73686158	circRNA_001482	3:104182825|104199715	circRNA_003600	15:111788024|111838997
circRNA_014843	5:45996382|45998196	circRNA_010702	5:51910032|51916859	circRNA_020329	9:108154906|108171796	circRNA_010491	15:111788024|111838999
circRNA_004882	8:12441715|12443530	circRNA_021382	X:113348505|113355334	circRNA_010144	X:101651839|101668730	circRNA_010235	1:225120006|225170992
circRNA_009321	2:154312784|154314599	circRNA_003990	2:112705863|112712693	circRNA_006505	9:125610011|125626904	circRNA_015768	16:48831766|48882768
circRNA_019453	11:75092092|75093907	circRNA_002723	1:257030254|257037086	circRNA_009226	2:27347578|27364474	circRNA_005035	8:146890664|146941701
circRNA_020567	12:56026618|56028433	circRNA_006819	10:29332925|29339758	circRNA_002357	GL894982.1:22842|39739	circRNA_010522	17:21974306|22025354
circRNA_011801	5:6490156|6491973	circRNA_011642	2:142524037|142530871	circRNA_010771	6:146111177|146128074	circRNA_015396	11:48358890|48409967
circRNA_012856	17:68993482|68995300	circRNA_018107	10:47444758|47451592	circRNA_014055	6:148469443|148486340	circRNA_005425	1:72497312|72548400
circRNA_014973	10:57994109|57995928	circRNA_003166	13:89609751|89616586	circRNA_015488	13:33815459|33832357	circRNA_009758	7:61582111|61633213
circRNA_016766	12:45354531|45356350	circRNA_014388	3:49908793|49915628	circRNA_015841	18:44432877|44449775	circRNA_012293	1:208422437|208473568
circRNA_013162	5:33031868|33033688	circRNA_014541	9:7316552|7323391	circRNA_002046	7:124977818|124994717	circRNA_000087	1:115244034|115295202
circRNA_020302	9:851539|853359	circRNA_009955	9:44250044|44256884	circRNA_002217	9:40746631|40763538	circRNA_006020	2:98275617|98326796
circRNA_005620	12:23727465|23729286	circRNA_000313	10:33604359|33611200	circRNA_002915	11:48285840|48302743	circRNA_019532	14:60727790|60779011
circRNA_008353	1:108480412|108482233	circRNA_016442	9:29693288|29700129	circRNA_003342	14:42204283|42221186	circRNA_005318	X:17297479|17348709
circRNA_017581	13:8025187|8027008	circRNA_020657	13:206140809|206147650	circRNA_006347	7:73254173|73271089	circRNA_007346	17:19717427|19768679
circRNA_018370	9:50832782|50834603	circRNA_003935	2:80553499|80560343	circRNA_003817	18:10795154|10812072	circRNA_017698	16:18596005|18647267
circRNA_012233	1:103551293|103553116	circRNA_012267	1:146847996|146854843	circRNA_001367	2:147428027|147444947	circRNA_012040	9:9466096|9517372
circRNA_017534	10:76119639|76121463	circRNA_012515	13:28308365|28315215	circRNA_000229	1:256850826|256867750	circRNA_018728	14:58547368|58598669
circRNA_016556	GL896549.1:21689|23514	circRNA_012031	8:144739677|144746528	circRNA_017339	9:78890360|78907286	circRNA_020469	1:257198606|257249935
circRNA_017826	4:39358395|39360221	circRNA_000309	10:33071613|33078465	circRNA_008784	13:89609751|89626678	circRNA_020051	14:136683518|136734852
circRNA_010082	GL896137.1:64521|66349	circRNA_004032	2:146232432|146239284	circRNA_002436	X:116235048|116251977	circRNA_015134	9:140018765|140070106
circRNA_020077	16:36895597|36897425	circRNA_019813	8:18869389|18876241	circRNA_017589	13:37977080|37994009	circRNA_020256	7:39845466|39896839
circRNA_013152	5:23289255|23291084	circRNA_005777	14:33995704|34002557	circRNA_015266	1:227193880|227210811	circRNA_001439	3:74727498|74778873
circRNA_019857	9:136580216|136582045	circRNA_000990	15:115438915|115445769	circRNA_017072	3:80493595|80510528	circRNA_006969	13:18089043|18140422
circRNA_007004	13:73190426|73192256	circRNA_012752	15:110692104|110698959	circRNA_020286	8:39593744|39610679	circRNA_000369	10:77572815|77624209
circRNA_020186	4:121453705|121455535	circRNA_006759	1:242160698|242167556	circRNA_006653	1:96785770|96802706	circRNA_009203	18:52556214|52607709
circRNA_005858	15:105482363|105484194	circRNA_007486	2:90673619|90680477	circRNA_007922	7:65167966|65184904	circRNA_016265	6:156602111|156653633
circRNA_007627	4:17245368|17247200	circRNA_010922	GL896372.1:9479|16337	circRNA_012520	13:34925731|34942673	circRNA_013493	9:124826954|124878480
circRNA_002965	12:15273975|15275808	circRNA_014555	9:78892371|78899230	circRNA_019676	3:84446033|84462977	circRNA_015874	2:30477465|30528998
circRNA_013322	7:57838059|57839892	circRNA_015139	1:14096258|14103118	circRNA_000098	1:124997045|125013992	circRNA_004198	3:120260142|120311693
circRNA_014420	4:51531332|51533165	circRNA_020471	1:266455035|266461897	circRNA_018288	5:71665675|71682624	circRNA_013928	5:87112433|87164024
circRNA_015703	15:67058529|67060363	circRNA_020123	2:120394337|120401200	circRNA_017990	9:51507092|51524042	circRNA_008484	1:280648787|280700379
circRNA_011290	13:206964722|206966557	circRNA_002238	9:58396899|58403763	circRNA_005434	1:101009473|101026424	circRNA_011540	18:14762483|14814105
circRNA_012703	14:148266744|148268579	circRNA_012659	14:68168562|68175427	circRNA_001387	3:16580139|16597096	circRNA_008020	8:71895225|71946859
circRNA_020116	2:59040088|59041924	circRNA_001767	5:99055437|99062303	circRNA_013301	7:10291894|10308853	circRNA_007927	7:73042261|73093908
circRNA_007959	7:110044513|110046350	circRNA_008415	1:186643400|186650267	circRNA_012729	15:74445099|74462062	circRNA_013406	8:109011842|109063493
circRNA_017522	10:46289771|46291609	circRNA_009012	15:62342057|62348924	circRNA_010196	1:96789076|96806044	circRNA_013140	4:137358677|137410340
circRNA_006165	4:103228326|103230166	circRNA_008838	13:159976238|159983106	circRNA_003016	12:39200635|39217604	circRNA_018832	16:47778049|47829752
circRNA_009730	7:21551602|21553443	circRNA_008904	14:51298319|51305187	circRNA_008746	13:43406481|43423452	circRNA_002852	10:62839029|62890750
circRNA_006255	6:26465042|26466884	circRNA_001596	4:79089337|79096209	circRNA_009300	2:128769962|128786933	circRNA_004375	4:134354742|134406551
circRNA_015716	15:105377422|105379265	circRNA_006651	1:94535554|94542426	circRNA_015085	13:126801546|126818519	circRNA_004848	7:117120845|117172676

circRNA_012501	12:48453817|48455661	circRNA_007068	13:146879487|146886361	circRNA_003310	14:21515998|21532972	circRNA_014220	13:144555700|144607544
circRNA_009919	9:9546451|9548296	circRNA_019232	8:127513966|127520840	circRNA_017163	6:25859998|25876972	circRNA_004792	7:67535445|67587294
circRNA_017692	15:145002737|145004583	circRNA_002961	12:14530678|14537553	circRNA_015277	1:230986044|231003021	circRNA_001929	6:148414520|148466426
circRNA_001847	6:91918116|91919964	circRNA_000256	1:281712176|281719052	circRNA_010701	5:41367549|41384528	circRNA_003742	17:5885678|5937612
circRNA_012476	12:21154589|21156437	circRNA_014608	1:60893530|60900407	circRNA_020651	13:170769226|170786207	circRNA_019679	3:102783623|102835561
circRNA_021386	X:142022925|142024774	circRNA_002170	9:400676|407555	circRNA_002233	9:51507059|51524042	circRNA_014186	13:7975063|8027008
circRNA_005994	2:81755819|81757669	circRNA_004855	7:123615110|123621989	circRNA_018540	10:29514408|29531391	circRNA_001406	3:39426206|39478158
circRNA_007094	14:8570680|8572530	circRNA_005914	17:9703518|9710397	circRNA_009733	7:23933083|23950067	circRNA_021311	GL893841.1:17187|69147
circRNA_018524	1:283916128|283917980	circRNA_007598	3:113401335|113408214	circRNA_016049	4:15914060|15931051	circRNA_008451	1:233128640|233180602
circRNA_009464	4:68358494|68360347	circRNA_019050	4:129522757|129529638	circRNA_009500	4:129522757|129539754	circRNA_012971	2:130933380|130985342
circRNA_011804	5:29892331|29894186	circRNA_015510	13:55485637|55492521	circRNA_003950	2:86094825|86111828	circRNA_009009	15:59683347|59735328
circRNA_009852	8:57889006|57890863	circRNA_018595	12:27154978|27161862	circRNA_019559	15:11929|28932	circRNA_005639	12:59331765|59383777
circRNA_014845	5:92098435|92100292	circRNA_002176	9:7316506|7323391	circRNA_003955	2:86274979|86291987	circRNA_006774	1:270185577|270237597
circRNA_001754	5:82860913|82862771	circRNA_009139	17:30266519|30273404	circRNA_017133	4:137164662|137181671	circRNA_017029	2:86079351|86131375
circRNA_004636	6:101811732|101813590	circRNA_005782	14:43487689|43494583	circRNA_005911	17:717212|734222	circRNA_002733	1:270185572|270237597
circRNA_007873	6:137193371|137195229	circRNA_000474	12:27599876|27606771	circRNA_001289	2:86274975|86291987	circRNA_013160	5:32835002|32887030
circRNA_008432	1:202686258|202688116	circRNA_002908	11:25434300|25441196	circRNA_003216	13:143973209|143990223	circRNA_004621	6:99103127|99155163
circRNA_003881	2:8371841|8373701	circRNA_007711	5:988588|995484	circRNA_009101	16:39410526|39427540	circRNA_013338	7:91930063|91982115
circRNA_005796	14:86962587|86964447	circRNA_008913	14:59642266|59649165	circRNA_018055	X:84998268|85015283	circRNA_010865	9:19259967|19312056
circRNA_009084	16:24992289|24994149	circRNA_005410	1:23232804|23239705	circRNA_005640	12:59366760|59383777	circRNA_020505	10:33597363|33649475
circRNA_010674	4:85302651|85304511	circRNA_005625	12:37178410|37185311	circRNA_006690	1:143245293|143262310	circRNA_004984	8:103659931|103712082
circRNA_005526	1:297466978|297468840	circRNA_009310	2:142522062|142528964	circRNA_002636	1:183393407|183410425	circRNA_002521	1:80241844|80293999
circRNA_012471	12:16977581|16979444	circRNA_001479	3:102415331|102422235	circRNA_011927	7:14136139|14153157	circRNA_007974	7:130799674|130851846
circRNA_018040	GL896571.2:133111|134975	circRNA_013121	4:108617797|108624702	circRNA_016400	8:120683094|120700114	circRNA_019642	2:113516538|113568728
circRNA_020931	2:157284602|157286466	circRNA_014313	16:47323622|47330528	circRNA_016337	8:3343742|3360764	circRNA_018545	10:37747971|37800179
circRNA_008329	1:80327077|80328942	circRNA_001405	3:38675766|38682674	circRNA_021384	X:126970727|126987749	circRNA_017571	12:40541328|40593559
circRNA_008582	11:11405097|11406962	circRNA_001681	5:4405930|4412839	circRNA_008014	8:58349146|58366177	circRNA_020747	15:86619273|86671532
circRNA_010165	1:16356085|16357950	circRNA_017807	3:118510677|118517586	circRNA_008885	14:30965816|30982848	circRNA_009023	15:75641326|75693603
circRNA_019404	1:248990243|248992113	circRNA_011969	7:105383271|105390181	circRNA_017680	15:101663406|101680438	circRNA_000075	1:97325990|97378276
circRNA_001391	3:18519549|18521420	circRNA_003446	14:122449149|122456060	circRNA_003068	13:15532621|15549655	circRNA_017424	1:21231837|21284136
circRNA_019043	4:108622830|108624702	circRNA_013828	13:156013911|156020824	circRNA_018670	13:145987809|146004845	circRNA_019439	11:498720|551063
circRNA_014963	1:103475507|103477380	circRNA_011147	11:72371861|72378775	circRNA_021179	7:118961846|118978888	circRNA_014499	7:73050148|73102497
circRNA_019372	1:124814761|124816635	circRNA_000939	15:73509995|73516910	circRNA_009107	16:47887052|47904101	circRNA_009181	18:14762483|14814873
circRNA_016645	1:126461855|126463730	circRNA_017565	12:27561099|27568015	circRNA_008818	13:138069734|138086786	circRNA_005556	10:46726604|46779066
circRNA_005739	13:167147677|167149553	circRNA_004011	2:128813680|128820597	circRNA_003369	14:66870396|66887449	circRNA_019136	6:145356549|145409067
circRNA_008223	GL896374.1:28001|29877	circRNA_002477	1:23236777|23243695	circRNA_002266	9:98631547|98648601	circRNA_001157	18:14762483|14815003
circRNA_011081	10:14933620|14935496	circRNA_014875	8:44980268|44987187	circRNA_008879	14:21576022|21593078	circRNA_006128	4:32646874|32699400
circRNA_014691	3:31095867|31097743	circRNA_001093	17:19828222|19835142	circRNA_012702	14:142837282|142854338	circRNA_002623	1:161713076|161765610
circRNA_013783	1:210259053|210260930	circRNA_006841	10:54046151|54053071	circRNA_021325	GL894993.1:13224|30287	circRNA_008956	14:124404348|124456895
circRNA_003314	14:24860954|24862833	circRNA_002094	8:73375718|73382639	circRNA_021109	6:85026828|85043893	circRNA_005990	2:78479725|78532281
circRNA_014391	3:61097768|61099647	circRNA_003884	2:11358850|11365773	circRNA_001212	2:4157505|4174576	circRNA_020916	2:132404785|132457350
circRNA_019217	8:78187072|78188951	circRNA_004904	8:34670670|34677594	circRNA_008881	14:30441264|30458336	circRNA_020419	1:154901709|154954283
circRNA_021307	GL893374.2:16729|18609	circRNA_018191	16:23643977|23650903	circRNA_019450	11:26287974|26305046	circRNA_002690	1:227144723|227197323
circRNA_006902	12:2288179|2290061	circRNA_016012	3:109429044|109435971	circRNA_004281	4:56568602|56585675	circRNA_005571	10:76253466|76306111
circRNA_006250	6:13369364|13371247	circRNA_008505	10:154814|161742	circRNA_014158	10:47097358|47114448	circRNA_020076	16:36443209|36495862
circRNA_009044	15:115718457|115720341	circRNA_011570	2:19835406|19842334	circRNA_016723	10:35025752|35042842	circRNA_007760	5:70576776|70629435
circRNA_010670	4:72919389|72921273	circRNA_012965	2:119213114|119220044	circRNA_004286	4:68353630|68370721	circRNA_012854	17:65833013|65885696
circRNA_013727	7:122522392|122524276	circRNA_016694	1:263633167|263640097	circRNA_004643	6:105486591|105503682	circRNA_007489	2:91601752|91654443
circRNA_011184	12:51830838|51832723	circRNA_000916	15:55433780|55440711	circRNA_017500	1:254965315|254982407	circRNA_014683	2:91601749|91654443
circRNA_020917	2:134981210|134983095	circRNA_018278	5:576306|583237	circRNA_012738	15:87215202|87232295	circRNA_009172	18:9569859|9622572
circRNA_012829	17:13090591|13092479	circRNA_006104	3:109913152|109920084	circRNA_015822	18:15034652|15051751	circRNA_015304	1:280648787|280701515
circRNA_011922	6:157208869|157210758	circRNA_007354	17:40446021|40452955	circRNA_001064	16:47800356|47817458	circRNA_002304	9:143198081|143250812

circRNA_014726	7:87834158|87836047	circRNA_003856	18:44782112|44789048	circRNA_011280	13:167117507|167134609	circRNA_005964	2:723533|776266
circRNA_020980	3:107580168|107582057	circRNA_012230	1:101627933|101634870	circRNA_006309	6:147612968|147630071	circRNA_006019	2:97529618|97582396
circRNA_009514	5:1112631|1114522	circRNA_012132	GL895853.2:76998|83937	circRNA_017188	6:99242373|99259476	circRNA_013393	8:82954008|83006817
circRNA_013422	8:141448485|141450376	circRNA_002722	1:256994751|257001691	circRNA_003383	14:78009269|78026373	circRNA_021083	5:106543058|106595873
circRNA_001007	15:122459592|122461484	circRNA_004101	3:72464696|72471636	circRNA_001151	18:10792104|10809210	circRNA_013697	16:36722483|36775306
circRNA_014351	2:9163330|9165222	circRNA_010283	10:44197866|44204806	circRNA_010407	13:167802687|167819794	circRNA_005993	2:81251743|81304602
circRNA_002649	1:195242228|195244121	circRNA_015992	3:75950705|75957646	circRNA_020045	14:112935251|112952367	circRNA_005661	13:33967997|34020867
circRNA_013011	3:57863218|57865111	circRNA_008404	1:176857604|176864548	circRNA_003507	15:22768074|22785191	circRNA_007821	6:63443230|63496102
circRNA_000573	13:38024064|38025958	circRNA_012298	1:227159382|227166326	circRNA_005109	9:43033759|43050877	circRNA_017768	2:103773335|103826215
circRNA_003182	13:102343369|102345263	circRNA_020336	9:135417867|135424815	circRNA_020855	2:3022188|3039309	circRNA_017643	14:87294200|87347081
circRNA_017757	2:66709085|66710979	circRNA_003203	13:130233837|130240788	circRNA_003939	2:82153520|82170645	circRNA_003070	13:16473712|16526599
circRNA_000312	10:33271895|33273790	circRNA_018522	1:283089950|283096903	circRNA_001941	7:82092|99219	circRNA_010021	9:138832114|138885004
circRNA_006474	9:40661692|40663587	circRNA_014853	6:122000800|122007755	circRNA_018128	13:91057347|91074476	circRNA_015937	2:143080815|143133707
circRNA_015690	15:53781864|53783759	circRNA_000146	1:145429099|145436055	circRNA_008286	X:144149375|144166508	circRNA_002248	9:74154092|74206987
circRNA_017368	GL894350.2:28562|30458	circRNA_001609	4:90929684|90936640	circRNA_018744	14:79054583|79071718	circRNA_007395	18:27396026|27448992
circRNA_006519	GL893366.1:14717|16614	circRNA_001388	3:17136409|17143367	circRNA_009483	4:96740134|96757270	circRNA_021008	4:39885800|39938780
circRNA_012226	1:97175019|97176916	circRNA_008207	GL894386.1:47835|54793	circRNA_020673	14:33695132|33712273	circRNA_002143	8:128726506|128779487
circRNA_008538	10:47245359|47247257	circRNA_012025	8:139050881|139057840	circRNA_002909	11:25511459|25528603	circRNA_003207	13:138907625|138960618
circRNA_004861	7:128851816|128853715	circRNA_012790	16:21326993|21333952	circRNA_014786	13:130101830|130118975	circRNA_005866	15:116154400|116207401
circRNA_007130	14:53590430|53592330	circRNA_016108	4:129535947|129542909	circRNA_001719	5:56916166|56933313	circRNA_002017	7:97005367|97058377
circRNA_010209	1:145751517|145753417	circRNA_003175	13:91488790|91495754	circRNA_007884	6:148118708|148135855	circRNA_017054	2:148139473|148192512
circRNA_010776	6:157208869|157210769	circRNA_005836	15:55345812|55352780	circRNA_019292	9:127848094|127865241	circRNA_001598	4:83633287|83686353
circRNA_004142	3:92448986|92450888	circRNA_001309	2:93467543|93474512	circRNA_010801	7:73165386|73182534	circRNA_013447	9:37817622|37870695
circRNA_006021	2:100724472|100726374	circRNA_003925	2:71195690|71202659	circRNA_007937	7:91938950|91956101	circRNA_011761	4:39835278|39888370
circRNA_021316	GL894386.1:27340|29242	circRNA_008264	X:85954041|85961011	circRNA_013096	4:45690143|45707294	circRNA_016883	14:144925981|144979077
circRNA_007440	2:48076528|48078432	circRNA_000298	10:24486122|24493093	circRNA_003439	14:120762011|120779168	circRNA_010249	1:270602784|270655882
circRNA_009251	2:61866062|61867966	circRNA_008705	13:3130975|3137949	circRNA_012802	16:37494597|37511757	circRNA_020869	2:49858927|49912043
circRNA_002038	7:119977886|119979791	circRNA_007398	18:28200384|28207359	circRNA_007153	14:78009211|78026373	circRNA_000750	14:21564778|21617912
circRNA_001107	17:43051169|43053075	circRNA_015966	3:45768689|45775664	circRNA_018926	2:88552220|88569384	circRNA_017470	1:169304335|169357476
circRNA_007069	13:148444729|148446635	circRNA_020722	14:146924554|146931529	circRNA_013467	9:72790436|72807602	circRNA_015299	1:276352633|276405786
circRNA_015177	1:76213543|76215451	circRNA_015038	5:83970725|83977701	circRNA_005636	12:52763999|52781167	circRNA_013941	6:150842053|150895226
circRNA_002085	8:43586936|43588846	circRNA_007538	3:24141255|24148233	circRNA_008849	13:202631408|202648576	circRNA_007886	6:148401539|148454719
circRNA_017311	9:11740106|11742016	circRNA_018070	1:24157618|24164598	circRNA_011846	5:94428393|94445562	circRNA_014996	14:115712680|115765873
circRNA_007163	14:98553323|98555234	circRNA_018322	6:145323643|145330625	circRNA_020761	15:117869607|117886776	circRNA_014962	1:103173037|103226272
circRNA_012904	2:17314802|17316714	circRNA_007800	6:31456596|31463579	circRNA_019238	8:141440630|141457805	circRNA_000865	14:142803973|142857217
circRNA_013124	4:116733183|116735096	circRNA_016455	9:40778851|40785836	circRNA_001667	4:142854258|142871435	circRNA_017291	8:123840476|123893742
circRNA_018383	GL893076.2:43811|45724	circRNA_012289	1:202809063|202816050	circRNA_020247	7:5791171|5808349	circRNA_017827	4:39835077|39888370
circRNA_000885	15:18728205|18730119	circRNA_020699	14:95425043|95432030	circRNA_002860	10:75674306|75691485	circRNA_020594	13:66451168|66504485
circRNA_015524	13:86739488|86741402	circRNA_019851	9:102920383|102927371	circRNA_020993	3:122078615|122095795	circRNA_002463	1:14699637|14752959
circRNA_006355	7:91976025|91977940	circRNA_004742	7:31326956|31333945	circRNA_000243	1:270602784|270619968	circRNA_009530	5:32833666|32887030
circRNA_009421	3:134295908|134297823	circRNA_017092	3:135140353|135147343	circRNA_020193	5:35802271|35819455	circRNA_018098	1:280627976|280681396
circRNA_008734	13:35779334|35781251	circRNA_008460	1:244830879|244837872	circRNA_013272	6:125658483|125675668	circRNA_013827	13:149631857|149685278
circRNA_008080	9:11209215|11211136	circRNA_011071	1:303004139|303011132	circRNA_015604	14:37732669|37749858	circRNA_012648	14:58534019|58587459
circRNA_009173	18:10157853|10159774	circRNA_015012	17:43030480|43037474	circRNA_018852	17:43352074|43369268	circRNA_015713	15:91537307|91590776
circRNA_018972	3:43107742|43109664	circRNA_014196	13:43455004|43461999	circRNA_004668	6:138053763|138070968	circRNA_019079	5:76327567|76381045
circRNA_020184	4:114447040|114448962	circRNA_011076	1:306800281|306807277	circRNA_007175	14:116594582|116611789	circRNA_009608	6:9691062|9744554
circRNA_015957	3:23075177|23077100	circRNA_009856	8:71459662|71466659	circRNA_010428	14:31381610|31398817	circRNA_019231	8:123961626|124015123
circRNA_014431	4:91138990|91140914	circRNA_007725	5:16681220|16688220	circRNA_019596	17:44055387|44072594	circRNA_002736	1:270329795|270383304
circRNA_008805	13:108410122|108412047	circRNA_002937	11:74714006|74721007	circRNA_012630	14:32126531|32143744	circRNA_007891	6:154511955|154565489
circRNA_004425	5:36458959|36460887	circRNA_010645	3:128289492|128296495	circRNA_007278	15:120884639|120901856	circRNA_020039	14:52205470|52259009
circRNA_009198	18:44805458|44807387	circRNA_015035	5:17115286|17122289	circRNA_021002	4:19824111|19841332	circRNA_005910	16:78913434|78966976
circRNA_011436	15:115478519|115480450	circRNA_015285	1:248423400|248430404	circRNA_011571	2:26772771|26789993	circRNA_008467	1:249858161|249911706

circRNA_013157	5:31973711|31975642	circRNA_002629	1:176227875|176234880	circRNA_002672	1:204790935|204808158	circRNA_011013	1:143396359|143449923
circRNA_013879	2:45070552|45072483	circRNA_007843	6:92090650|92097655	circRNA_014376	2:132435226|132452449	circRNA_010595	2:139112346|139165912
circRNA_013706	2:104844828|104846760	circRNA_016753	12:16187562|16194567	circRNA_004319	4:98044696|98061929	circRNA_008908	14:57935077|57988669
circRNA_007027	13:91074284|91076217	circRNA_017329	9:53368934|53375941	circRNA_010003	9:109715920|109733157	circRNA_010733	6:30708117|30761713
circRNA_007050	13:131235949|131237882	circRNA_001426	3:55777082|55784090	circRNA_011496	16:51922170|51939408	circRNA_011764	4:41190969|41244579
circRNA_008780	13:86952848|86954781	circRNA_002030	7:104156049|104163058	circRNA_008643	12:16046492|16063733	circRNA_017210	6:149163669|149217315
circRNA_009356	3:46271692|46273625	circRNA_009851	8:57803396|57810406	circRNA_003341	14:42111193|42128438	circRNA_000706	13:205921704|205975398
circRNA_014669	16:23465932|23467865	circRNA_014841	5:23685402|23692412	circRNA_002211	9:36850715|36867961	circRNA_008787	13:90896704|90950445
circRNA_016001	3:84698909|84700842	circRNA_019859	9:143389445|143396455	circRNA_002556	1:116900620|116917868	circRNA_019286	9:117856547|117910301
circRNA_018642	13:81545108|81547042	circRNA_008536	10:44171899|44178913	circRNA_007135	14:60127644|60144892	circRNA_012649	14:58537186|58590955
circRNA_019732	5:61141150|61143085	circRNA_016767	12:45504861|45511876	circRNA_012725	15:66048762|66066010	circRNA_008892	14:34675990|34729763
circRNA_017808	3:118555217|118557154	circRNA_016946	16:59908414|59915429	circRNA_017809	3:121657951|121675200	circRNA_011448	15:124666386|124720160
circRNA_001384	3:10227479|10229417	circRNA_000465	12:21068070|21075089	circRNA_000027	1:21231837|21249088	circRNA_013816	13:59840070|59893851
circRNA_008935	14:82913063|82915001	circRNA_005600	11:53841367|53848387	circRNA_006076	3:57385109|57402361	circRNA_001168	18:18661914|18715721
circRNA_011988	8:5815605|5817543	circRNA_019284	9:115602126|115609146	circRNA_001928	6:148405581|148422837	circRNA_020228	6:99205646|99259476
circRNA_011238	13:87138870|87140810	circRNA_006701	1:152319829|152326850	circRNA_012467	12:14819804|14837065	circRNA_016865	14:94716575|94770407
circRNA_017872	6:22706|24646	circRNA_013623	10:21667651|21674674	circRNA_002329	GL893171.1:5494|22757	circRNA_005115	9:49350469|49404311
circRNA_001219	2:7628883|7630825	circRNA_018380	GL892334.1:10746|17769	circRNA_021100	6:38035735|38052998	circRNA_005011	8:134355181|134409025
circRNA_006288	6:98799538|98801480	circRNA_014511	7:116360508|116367534	circRNA_001308	2:92702721|92719989	circRNA_002188	9:11337234|11391169
circRNA_002234	9:51522099|51524042	circRNA_003946	2:83908876|83915903	circRNA_005327	X:40486596|40503864	circRNA_013638	5:53477815|53531813
circRNA_010475	15:67048310|67050253	circRNA_014489	7:37126123|37133150	circRNA_000546	13:25244218|25261487	circRNA_014459	5:87157448|87211468
circRNA_003104	13:37111091|37113036	circRNA_007562	3:71000484|71007512	circRNA_017829	4:42114119|42131389	circRNA_017134	4:139682020|139736055
circRNA_015034	3:130866933|130868878	circRNA_018523	1:283467788|283474817	circRNA_009829	8:31947681|31964962	circRNA_007164	14:104732283|104786323
circRNA_003786	17:49600368|49602315	circRNA_002565	1:124967959|124974989	circRNA_019903	1:28708099|28725381	circRNA_002189	9:11747252|11801305
circRNA_006061	3:17103410|17105358	circRNA_018814	16:3605285|3612320	circRNA_001370	2:148139473|148156756	circRNA_021170	7:89101045|89155100
circRNA_011079	10:14863084|14865032	circRNA_011000	1:134790067|134797103	circRNA_000721	13:212995887|213013171	circRNA_014854	6:138904496|138958631
circRNA_007655	4:60858276|60860227	circRNA_012513	13:20961472|20968509	circRNA_019125	6:125615895|125633179	circRNA_014138	1:230674897|230729058
circRNA_011690	3:71000484|71002436	circRNA_019879	GL896447.1:19752|26793	circRNA_001811	6:63478817|63496102	circRNA_009446	4:40299977|40354146
circRNA_017979	9:13306787|13308739	circRNA_003876	2:4157505|4164547	circRNA_008301	1:21231837|21249125	circRNA_006075	3:54706870|54761061
circRNA_013196	5:88193297|88195251	circRNA_008945	14:114549878|114556920	circRNA_014017	3:75420602|75437890	circRNA_009949	9:40955217|41009434
circRNA_006211	5:63603521|63605477	circRNA_007558	3:61090978|61098022	circRNA_003937	2:82120327|82137621	circRNA_001670	5:162936|217179
circRNA_016998	2:8292820|8294777	circRNA_013232	6:39500462|39507506	circRNA_011099	10:33568182|33585476	circRNA_014437	4:135627272|135681519
circRNA_020273	7:116855649|116857606	circRNA_014737	9:11010683|11017729	circRNA_017685	15:107370970|107388267	circRNA_004448	5:61142992|61197241
circRNA_013324	7:60799836|60801794	circRNA_021140	6:147451159|147458207	circRNA_008732	13:35641550|35658848	circRNA_004510	5:109487613|109541874
circRNA_016009	3:104155199|104157157	circRNA_014221	13:147773671|147780722	circRNA_017884	6:63478817|63496116	circRNA_012995	3:24575402|24629671
circRNA_012026	8:141399041|141401000	circRNA_009436	4:20378229|20385281	circRNA_009510	4:141816454|141833754	circRNA_018536	10:20077658|20131932
circRNA_009925	9:21947403|21949364	circRNA_000456	12:16845381|16852434	circRNA_015401	11:56099855|56117160	circRNA_011815	5:50595002|50649277
circRNA_014197	13:49818677|49820638	circRNA_005081	9:24090254|24097307	circRNA_015487	13:33722489|33739802	circRNA_006967	13:16472252|16526599
circRNA_006254	6:26460696|26462658	circRNA_003185	13:103719305|103726359	circRNA_007393	18:26590275|26607594	circRNA_019444	11:11281429|11335777
circRNA_016505	9:143556231|143558193	circRNA_019469	13:2382115|2389171	circRNA_012769	15:118068018|118085341	circRNA_008542	10:50688302|50742685
circRNA_009554	5:61457868|61459831	circRNA_004347	4:117915467|117922524	circRNA_013946	7:100611865|100629188	circRNA_014928	2:119947335|120001743
circRNA_006807	10:13453971|13455936	circRNA_004129	3:84423735|84430797	circRNA_013203	5:93363144|93380473	circRNA_019399	1:225754533|225808956
circRNA_010993	1:132451642|132453607	circRNA_004867	7:134300825|134307888	circRNA_011298	13:216669717|216687050	circRNA_017117	4:87209598|87264024
circRNA_014759	1:32054762|32056727	circRNA_012534	13:44001591|44008655	circRNA_008155	9:115761298|115778635	circRNA_004191	3:120081729|120136187
circRNA_013245	6:75922421|75924387	circRNA_013718	5:22719002|22726068	circRNA_009468	4:72458457|72475797	circRNA_013578	X:46489086|46543552
circRNA_013550	GL896489.1:3791|5757	circRNA_008793	13:91506047|91513114	circRNA_008081	9:11210975|11228318	circRNA_010634	3:104155199|104209684
circRNA_018021	GL893520.1:14693|16659	circRNA_014732	8:71505036|71512104	circRNA_014084	6:157496770|157514113	circRNA_005443	1:127622340|127676835
circRNA_000539	13:21228133|21230101	circRNA_009845	8:43039144|43046213	circRNA_005737	13:156006479|156023826	circRNA_006119	3:130866933|130921437
circRNA_004680	6:145341347|145343315	circRNA_012084	9:127871138|127878207	circRNA_019798	7:73243547|73260894	circRNA_017574	12:45457349|45511876
circRNA_013779	1:195020817|195022785	circRNA_005085	9:26053628|26060698	circRNA_015970	3:49898276|49915628	circRNA_010110	X:14511526|14566099
circRNA_005503	1:230084757|230086726	circRNA_013295	6:153769525|153776595	circRNA_013847	15:71703524|71720879	circRNA_009423	3:134657095|134711682
circRNA_004522	6:12077068|12079038	circRNA_019566	15:73290746|73297816	circRNA_006194	5:16670864|16688220	circRNA_008817	13:135093898|135148500

circRNA_013997	12:22572340|22574310	circRNA_001552	4:19562816|19569887	circRNA_009654	6:81212689|81230045	circRNA_011338	14:68445457|68500095
circRNA_013035	3:97836618|97838589	circRNA_015458	12:48149958|48157029	circRNA_013837	14:60127644|60145000	circRNA_015754	16:27423986|27478630
circRNA_015864	2:8548411|8550383	circRNA_012944	2:82558670|82565744	circRNA_002377	GL896447.1:37117|54475	circRNA_018655	13:113412584|113467257
circRNA_004097	3:62337027|62339000	circRNA_009973	9:53368861|53375941	circRNA_010971	1:74443452|74460814	circRNA_004256	4:39819040|39873716
circRNA_011074	1:304816140|304818115	circRNA_020131	3:34672088|34679168	circRNA_005613	12:16053861|16071225	circRNA_013909	4:39819040|39873757
circRNA_004561	6:65913845|65915822	circRNA_016522	GL893884.1:52517|59599	circRNA_000328	10:46273271|46290641	circRNA_003987	2:105082209|105136942
circRNA_009820	8:1727412|1729389	circRNA_002059	8:10590656|10597739	circRNA_015463	12:53721335|53738722	circRNA_009685	6:103686782|103741524
circRNA_013611	3:57484017|57485994	circRNA_002251	9:78882583|78889668	circRNA_016452	9:40552034|40569423	circRNA_003277	13:211155143|211209889
circRNA_011419	15:85480849|85482828	circRNA_014203	13:75857204|75864289	circRNA_008098	9:36271212|36288607	circRNA_017253	8:19208010|19262804
circRNA_005335	X:51863815|51865795	circRNA_005204	9:143484202|143491288	circRNA_017497	1:247788345|247805744	circRNA_009407	3:120085410|120140221
circRNA_018184	15:115173583|115175563	circRNA_002219	9:41145064|41152151	circRNA_018819	16:23710913|23728315	circRNA_015245	1:205472022|205526853
circRNA_017167	6:44401007|44402988	circRNA_011319	14:33642903|33649990	circRNA_009281	2:108973031|108990435	circRNA_003991	2:118253397|118308249
circRNA_009783	7:83196831|83198816	circRNA_011422	15:88381955|88389043	circRNA_018613	12:51972452|51989856	circRNA_014040	12:12749163|12804024
circRNA_020095	18:1843187|1845173	circRNA_019845	9:29833529|29840619	circRNA_020538	11:74603718|74621124	circRNA_008788	13:90897875|90952792
circRNA_008662	12:34001990|34003977	circRNA_005552	10:37689024|37696115	circRNA_012121	GL894522.1:31987|49397	circRNA_004376	4:134354742|134409672
circRNA_014378	2:148348524|148350512	circRNA_006695	1:144006899|144013993	circRNA_016889	15:18690562|18707974	circRNA_007400	18:36480482|36535413
circRNA_012225	1:93363329|93365318	circRNA_015994	3:76037041|76044136	circRNA_000717	13:210819581|210836997	circRNA_012704	15:3755802|3810739
circRNA_002040	7:121258976|121260967	circRNA_020961	3:60086828|60093923	circRNA_004423	5:33938784|33956200	circRNA_007914	7:57914632|57969573
circRNA_017938	7:83521807|83523799	circRNA_017338	9:78882583|78889679	circRNA_015461	12:51830838|51848255	circRNA_017254	8:20670183|20725146
circRNA_018155	14:44212456|44214448	circRNA_012974	2:140765849|140772947	circRNA_006852	10:71290208|71307626	circRNA_016360	8:44980268|45035240
circRNA_020659	13:210873571|210875564	circRNA_007296	15:157089364|157096463	circRNA_017978	9:11795661|11813081	circRNA_020224	6:79846144|79901166
circRNA_007437	2:43993047|43995042	circRNA_012362	10:37747971|37755070	circRNA_020969	3:77312222|77329644	circRNA_020989	3:121657951|121713024
circRNA_009040	15:92433859|92435854	circRNA_015062	9:78882583|78889684	circRNA_016930	16:23703191|23720625	circRNA_004506	5:105210066|105265163
circRNA_003662	16:22466635|22468631	circRNA_017443	1:101579255|101586356	circRNA_010659	4:39870935|39888370	circRNA_001651	4:129426261|129481379
circRNA_006379	7:130889078|130891074	circRNA_008961	14:132245527|132252629	circRNA_012877	18:33873424|33890862	circRNA_001489	3:108496197|108551339
circRNA_016798	13:75597790|75599787	circRNA_020463	1:245588500|245595602	circRNA_002848	10:61381029|61398475	circRNA_012956	2:93539380|93594522
circRNA_007945	7:97744029|97746028	circRNA_021363	X:47803644|47810750	circRNA_011718	3:102818114|102835561	circRNA_009399	3:108496197|108551342
circRNA_014403	3:98684730|98686730	circRNA_009939	9:37910194|37917301	circRNA_015789	17:6298959|6316411	circRNA_009589	5:89469540|89524689
circRNA_016890	15:18728119|18730119	circRNA_017541	11:18532986|18540093	circRNA_002904	11:22573414|22590869	circRNA_011409	15:71690220|71745408
circRNA_001446	3:76334887|76336888	circRNA_003840	18:25520260|25527369	circRNA_020451	1:205509398|205526853	circRNA_018475	1:183355206|183410425
circRNA_016972	18:10560854|10562856	circRNA_014035	1:39747136|39754248	circRNA_002359	GL895028.1:36544|54000	circRNA_011594	2:54339863|54395084
circRNA_013116	4:90702984|90704987	circRNA_002054	8:3341344|3348457	circRNA_003362	14:60090378|60107836	circRNA_017797	3:72464696|72519969
circRNA_014680	2:80575287|80577295	circRNA_011952	7:69663561|69670674	circRNA_009230	2:30366335|30383793	circRNA_017237	7:100880481|100935783
circRNA_020611	13:91499963|91501971	circRNA_020151	3:107665453|107672566	circRNA_017573	12:45433318|45450776	circRNA_009294	2:121986386|122041690
circRNA_020810	17:42877581|42879589	circRNA_003423	14:114891766|114898881	circRNA_013270	6:111908180|111925644	circRNA_008644	12:16046492|16101827
circRNA_021178	7:116973936|116975944	circRNA_007714	5:4603986|4611101	circRNA_005765	14:14019138|14036603	circRNA_013193	5:87266266|87321630
circRNA_003438	14:120564864|120566873	circRNA_004050	3:5209263|5216379	circRNA_012841	17:43035603|43053075	circRNA_017850	4:135613831|135669202
circRNA_018702	14:13991235|13993244	circRNA_001221	2:15679097|15686214	circRNA_010894	9:113302666|113320145	circRNA_003140	13:76451613|76506992
circRNA_010616	3:45773654|45775664	circRNA_003693	16:37504640|37511757	circRNA_007938	7:91949364|91966849	circRNA_015926	2:130878078|130933457
circRNA_020130	3:33376445|33378455	circRNA_017739	18:27206105|27213224	circRNA_010054	GL893689.1:3069|20555	circRNA_012609	13:210781573|210836997
circRNA_007200	14:141212719|141214730	circRNA_001017	15:131201187|131208307	circRNA_019916	1:84274370|84291857	circRNA_020521	11:3544607|3600033
circRNA_019755	6:41841160|41843171	circRNA_019329	JH118841.1:116817|123937	circRNA_008844	13:184042251|184059739	circRNA_018779	15:54527316|54582748
circRNA_016719	10:19710729|19712741	circRNA_005283	GL896549.1:8294|15418	circRNA_016105	4:122094480|122111974	circRNA_008016	8:63842223|63897657
circRNA_016500	9:127711202|127713216	circRNA_007720	5:13204634|13211758	circRNA_002453	X:143993560|144011055	circRNA_011194	13:4737528|4792993
circRNA_018925	2:85972257|85974271	circRNA_014446	5:38344302|38351426	circRNA_018849	17:42001407|42018906	circRNA_006783	1:283819938|283875429
circRNA_006386	8:15406606|15408621	circRNA_010884	9:71446854|71453980	circRNA_015933	2:140344565|140362065	circRNA_018214	2:738000|793493
circRNA_005835	15:51648726|51650743	circRNA_017413	X:144003929|144011055	circRNA_006327	7:24626858|24644364	circRNA_001204	2:736962|792458
circRNA_013507	9:146508769|146510787	circRNA_018840	17:727089|734222	circRNA_019639	2:104885954|104903460	circRNA_007124	14:51693016|51748514
circRNA_002621	1:155379406|155381426	circRNA_000529	13:4737528|4744662	circRNA_014251	14:59116631|59134144	circRNA_004824	7:97101219|97156735
circRNA_012697	14:135990542|135992563	circRNA_001682	5:4601699|4608833	circRNA_007967	7:123615110|123632624	circRNA_005566	10:58856976|58912493
circRNA_015227	1:181328829|181330850	circRNA_013705	2:87910831|87917965	circRNA_003291	14:7185050|7202575	circRNA_017548	11:25838572|25894094
circRNA_018141	13:148469447|148471470	circRNA_017691	15:144880874|144888008	circRNA_019045	4:112851286|112868812	circRNA_012733	15:85325487|85381023

circRNA_012065	9:50988737|50990762	circRNA_018408	1:36687165|36694300	circRNA_009228	2:29207475|29225004	circRNA_009192	18:36583500|36639039
circRNA_005196	9:139980918|139982945	circRNA_019644	2:121549319|121556455	circRNA_000526	13:2389104|2406634	circRNA_009552	5:61142992|61198578
circRNA_006934	12:36052760|36054787	circRNA_008420	1:195022523|195029660	circRNA_015111	1:21222171|21239703	circRNA_017616	13:199361578|199417167
circRNA_009457	4:51227350|51229377	circRNA_011127	10:63281102|63288239	circRNA_012340	10:15497485|15515022	circRNA_010000	9:106588886|106644487
circRNA_013803	12:40309716|40311743	circRNA_015503	13:43454862|43461999	circRNA_005457	1:143267765|143285303	circRNA_009033	15:86615927|86671532
circRNA_012213	1:59384708|59386736	circRNA_018763	14:131867268|131874408	circRNA_008684	12:48983286|49000824	circRNA_009980	9:77414305|77469954
circRNA_003656	15:157072147|157074177	circRNA_016616	1:7408056|7415197	circRNA_015308	1:292555388|292572926	circRNA_016152	5:61141150|61196828
circRNA_009641	6:65141439|65143469	circRNA_002354	GL894855.1:11155|18297	circRNA_008457	1:242707926|242725465	circRNA_004632	6:101724150|101779902
circRNA_010076	GL895215.1:4533|6563	circRNA_018624	13:24111612|24118754	circRNA_017076	3:90750338|90767877	circRNA_005859	15:107266814|107322566
circRNA_010341	12:44352663|44354694	circRNA_000581	13:42385157|42392300	circRNA_016015	3:109717662|109735207	circRNA_008057	8:133985141|134040905
circRNA_005749	13:212098133|212100168	circRNA_006446	9:8134806|8141949	circRNA_015002	15:51612536|51630084	circRNA_020802	17:5122742|5178557
circRNA_016426	9:8944659|8946694	circRNA_009452	4:45549559|45556702	circRNA_020543	12:13978846|13996403	circRNA_018427	1:93309386|93365318
circRNA_011322	14:34693427|34695463	circRNA_002688	1:224441285|224448429	circRNA_004421	5:33828511|33846071	circRNA_005349	X:73954851|74010804
circRNA_013325	7:61907054|61909090	circRNA_003916	2:53089550|53096699	circRNA_018123	13:24091897|24109460	circRNA_007472	2:82502736|82558770
circRNA_011653	3:4984727|4986764	circRNA_021136	6:145083774|145090923	circRNA_008550	10:54031496|54049060	circRNA_017089	3:119696509|119752566
circRNA_019218	8:82191182|82193219	circRNA_008445	1:227048022|227055172	circRNA_004947	8:74346358|74363926	circRNA_009345	3:40002957|40059018
circRNA_007774	5:89608596|89610635	circRNA_015146	1:18676193|18683344	circRNA_005830	15:28533728|28551296	circRNA_001199	18:59639958|59696045
circRNA_011142	11:52232763|52234803	circRNA_018724	14:46222600|46229752	circRNA_006661	1:101744027|101761598	circRNA_015973	3:57429897|57485994
circRNA_016037	3:133957110|133959150	circRNA_008639	12:14508500|14515653	circRNA_007615	3:126274387|126291958	circRNA_015710	15:87308175|87364293
circRNA_003669	16:23596532|23598577	circRNA_004078	3:40918928|40926084	circRNA_004159	3:104192112|104209684	circRNA_015061	9:61508896|61565044
circRNA_010537	18:6267079|6269124	circRNA_017769	2:107023759|107030915	circRNA_012539	13:50636313|50653895	circRNA_009231	2:34556446|34612597
circRNA_015347	10:45903856|45905902	circRNA_001147	18:9637460|9644617	circRNA_016339	8:8742743|8760325	circRNA_017542	11:21049875|21106042
circRNA_016038	3:133994994|133997040	circRNA_007210	15:14442|21599	circRNA_012536	13:44090759|44108347	circRNA_021271	9:77586916|77643132
circRNA_000627	13:102350602|102352650	circRNA_015169	1:57003197|57010355	circRNA_015481	13:20990706|21008294	circRNA_009584	5:87252274|87308493
circRNA_010710	5:70043709|70045757	circRNA_000048	1:49510303|49517462	circRNA_002416	X:19334301|19351890	circRNA_014288	15:83538630|83594895
circRNA_011414	15:85220075|85222123	circRNA_009002	15:53153877|53161036	circRNA_010248	1:257023665|257041255	circRNA_015470	12:62862828|62919117
circRNA_018349	8:55441039|55443089	circRNA_013899	3:98018368|98025528	circRNA_018143	13:166909686|166927276	circRNA_008074	9:8186647|8242946
circRNA_009594	5:93527399|93529450	circRNA_004332	4:107641905|107649067	circRNA_001787	6:31800795|31818387	circRNA_014795	14:135990542|136046886
circRNA_012902	2:16118149|16120200	circRNA_003668	16:23398700|23405864	circRNA_011647	2:148145804|148163399	circRNA_016394	8:108964837|109021186
circRNA_003374	14:77540655|77542707	circRNA_003694	16:37507535|37514704	circRNA_011685	3:60805128|60822724	circRNA_017631	14:57946422|58002776
circRNA_010123	X:61016381|61018433	circRNA_012925	2:52968058|52975227	circRNA_021328	GL895308.2:181738|199336	circRNA_008395	1:161725478|161781848
circRNA_007734	5:32064191|32066245	circRNA_000792	14:62473110|62480280	circRNA_004595	6:85261799|85279398	circRNA_014023	6:148924023|148980488
circRNA_008218	GL896111.1:2464|4518	circRNA_004567	6:73230520|73237691	circRNA_009160	17:49153503|49171103	circRNA_008920	14:68600483|68656990
circRNA_012450	11:76239432|76241487	circRNA_010955	1:16109508|16116681	circRNA_006718	1:176376707|176394308	circRNA_002097	8:74346358|74402890
circRNA_019872	GL894906.1:24565|26620	circRNA_014999	14:140512869|140520043	circRNA_015823	18:15051566|15069170	circRNA_016491	9:108409166|108465721
circRNA_011640	2:141893166|141895222	circRNA_012300	1:228011592|228018767	circRNA_020424	1:176376707|176394312	circRNA_008950	14:117050975|117107550
circRNA_019466	12:51821064|51823121	circRNA_008332	1:84362746|84369923	circRNA_002840	10:57994109|58011719	circRNA_009668	6:92070354|92126940
circRNA_021058	5:66479882|66481939	circRNA_007256	15:85067809|85074988	circRNA_017310	9:11302178|11319790	circRNA_002502	1:58460995|58517592
circRNA_015353	10:47448975|47451033	circRNA_014475	6:85048146|85055325	circRNA_009473	4:73730788|73748405	circRNA_010621	3:58307813|58364568
circRNA_009780	7:80295592|80297651	circRNA_014543	9:13674798|13681977	circRNA_012723	15:63775222|63792841	circRNA_004606	6:89159443|89216201
circRNA_013514	GL892916.1:18814|20873	circRNA_021118	6:96504635|96511819	circRNA_014554	9:78882583|78900203	circRNA_009803	7:109768787|109825592
circRNA_004350	4:122107165|122109225	circRNA_003577	15:87922246|87929434	circRNA_018709	14:31005372|31022994	circRNA_017962	8:90653634|90710444
circRNA_001023	15:142019700|142021761	circRNA_005433	1:94570546|94577735	circRNA_015623	14:61615777|61633400	circRNA_010607	3:24575402|24632228
circRNA_010907	9:143540238|143542300	circRNA_005892	16:29638099|29645288	circRNA_018410	1:40749926|40767556	circRNA_004605	6:88792955|88849787
circRNA_011086	10:17402178|17404240	circRNA_010387	13:117332280|117339469	circRNA_001465	3:97935477|97953112	circRNA_019705	4:106690698|106747551
circRNA_007806	6:38146993|38149056	circRNA_012943	2:82454061|82461250	circRNA_011136	11:15977687|15995322	circRNA_003851	18:42650214|42707088
circRNA_020183	4:112631923|112633988	circRNA_016143	5:43308647|43315838	circRNA_006297	6:125470307|125487944	circRNA_014864	7:89078260|89135137
circRNA_001365	2:147085019|147087085	circRNA_003976	2:97457124|97464316	circRNA_009833	8:32878313|32895953	circRNA_018346	8:32486094|32542971
circRNA_010635	3:108192042|108194109	circRNA_014497	7:69682421|69689614	circRNA_020119	2:108995245|109012889	circRNA_018340	7:117053211|117110099
circRNA_010290	10:56118543|56120611	circRNA_006171	4:107641867|107649067	circRNA_015400	11:54000210|54017857	circRNA_013907	3:134023451|134080373
circRNA_011411	15:81273346|81275415	circRNA_010413	13:184052539|184059739	circRNA_014581	GL893655.1:37492|55141	circRNA_012788	16:9153185|9210113
circRNA_012068	9:58396899|58398969	circRNA_021348	JH118921.1:28722|35922	circRNA_001573	4:45252469|45270119	circRNA_000378	11:5210653|5267612

circRNA_008983	15:19216968|19219039	circRNA_014587	GL896344.1:8222|15423	circRNA_012077	9:105389643|105407294	circRNA_017069	3:66536815|66593786
circRNA_005387	X:126567527|126569599	circRNA_020889	2:83911477|83918678	circRNA_019298	9:138753548|138771200	circRNA_002086	8:44511824|44568805
circRNA_013545	GL895950.1:2867|4939	circRNA_003004	12:37228113|37235315	circRNA_002063	8:19208010|19225663	circRNA_003683	16:29621190|29678179
circRNA_005473	1:181330431|181332504	circRNA_013529	GL894377.1:8456|15659	circRNA_008719	13:24109347|24127002	circRNA_010820	8:9814040|9871034
circRNA_003785	17:49169026|49171103	circRNA_009798	7:101389795|101396999	circRNA_010806	7:91964457|91982115	circRNA_017554	11:76373396|76430435
circRNA_006146	4:55333062|55335142	circRNA_018602	12:37670958|37678162	circRNA_019929	1:161036936|161054605	circRNA_014162	10:62681670|62738784
circRNA_013311	7:35516579|35518659	circRNA_014021	5:63625257|63632464	circRNA_000329	10:46596019|46613689	circRNA_011998	8:40126184|40183332
circRNA_014816	2:80461235|80463317	circRNA_021142	6:148463817|148471024	circRNA_012533	13:43783286|43800956	circRNA_020125	2:137511855|137569028
circRNA_004252	4:37581896|37583979	circRNA_003440	14:120779066|120786274	circRNA_000683	13:158525065|158542736	circRNA_004545	6:32326772|32383981
circRNA_016646	1:126926296|126928380	circRNA_010321	11:86955208|86962417	circRNA_010754	6:103836621|103854303	circRNA_008697	12:62856482|62913707
circRNA_017048	2:142117527|142119611	circRNA_008158	9:117761925|117769135	circRNA_004177	3:113891379|113909062	circRNA_000963	15:86614285|86671532
circRNA_017801	3:102643211|102645295	circRNA_019297	9:138719819|138727029	circRNA_019692	4:8452625|8470308	circRNA_020705	14:115700129|115757379
circRNA_005619	12:22321255|22323341	circRNA_005248	GL894597.1:26840|34052	circRNA_011273	13:149836368|149854053	circRNA_011790	4:118832318|118889603
circRNA_008094	9:26051645|26053731	circRNA_014074	17:59438248|59445461	circRNA_017794	3:57369131|57386816	circRNA_009390	3:102669512|102726837
circRNA_010035	GL892332.2:322201|324287	circRNA_015778	16:59176203|59183416	circRNA_016112	4:135354311|135371998	circRNA_008739	13:38230967|38288294
circRNA_013835	14:36797468|36799554	circRNA_002618	1:152340211|152347427	circRNA_004828	7:97321612|97339300	circRNA_015670	14:136831008|136888438
circRNA_015707	15:85089238|85091324	circRNA_008685	12:52264072|52271288	circRNA_021274	9:79206662|79224350	circRNA_010395	13:132284548|132342011
circRNA_002374	GL896372.1:14249|16337	circRNA_014662	15:61682878|61690094	circRNA_010365	13:42298387|42316078	circRNA_007644	4:39885800|39943326
circRNA_020350	X:16953133|16955222	circRNA_013315	7:43188490|43195707	circRNA_013758	3:104182022|104199715	circRNA_004519	6:9977425|10034973
circRNA_007185	14:124044677|124046767	circRNA_006043	2:138829293|138836513	circRNA_004249	4:36900162|36917862	circRNA_021187	8:12659793|12717344
circRNA_020533	11:22573414|22575504	circRNA_003978	2:97497783|97505006	circRNA_009548	5:53612899|53630601	circRNA_003056	12:62856130|62913707
circRNA_019753	6:37996707|37998798	circRNA_016135	5:32061014|32068240	circRNA_015039	6:32224765|32242475	circRNA_013607	13:155607360|155664955
circRNA_007462	2:77617731|77619823	circRNA_019883	JH118963.1:42907|50133	circRNA_008005	8:42317790|42335501	circRNA_001134	18:735742|793351
circRNA_010265	10:17379819|17381911	circRNA_005759	14:9503215|9510442	circRNA_019803	7:95448814|95466527	circRNA_004948	8:74346358|74404030
circRNA_007453	2:66861252|66863345	circRNA_009476	4:88823008|88830238	circRNA_006582	X:113348505|113366223	circRNA_009046	15:116149729|116207401
circRNA_000258	1:283798123|283800217	circRNA_004229	4:15227218|15234449	circRNA_011170	12:23738975|23756693	circRNA_014646	13:145362490|145420162
circRNA_004717	7:12919796|12921890	circRNA_014971	10:31870846|31878079	circRNA_011051	1:245648519|245666238	circRNA_010929	X:14612680|14670417
circRNA_011895	6:92170081|92172175	circRNA_016531	GL894597.1:35377|42611	circRNA_020188	4:135544493|135562220	circRNA_014405	3:114318121|114375867
circRNA_002686	1:218683998|218686093	circRNA_004063	3:17254499|17261734	circRNA_021302	GL892789.1:62629|80358	circRNA_013317	7:52078950|52136752
circRNA_003722	16:51837295|51839390	circRNA_012268	1:152095176|152102411	circRNA_018995	3:102800563|102818293	circRNA_008824	13:144064121|144121936
circRNA_014002	13:207192306|207194401	circRNA_004557	6:64282430|64289666	circRNA_008037	8:103120812|103138543	circRNA_000764	14:34671930|34729763
circRNA_009889	8:123891646|123893742	circRNA_015197	1:110456936|110464173	circRNA_009480	4:91294507|91312241	circRNA_013787	1:226060684|226118520
circRNA_016920	15:157055344|157057440	circRNA_009035	15:86907264|86914503	circRNA_010202	1:127637224|127654960	circRNA_003482	14:147294220|147352065
circRNA_020470	1:264034002|264036098	circRNA_004706	6:152329597|152336837	circRNA_014245	14:48121030|48138766	circRNA_010339	12:38713970|38771817
circRNA_003701	16:43251568|43253665	circRNA_005357	X:97828399|97835641	circRNA_006249	6:13071374|13089115	circRNA_002776	10:17146793|17204667
circRNA_007740	5:42901737|42903838	circRNA_014478	6:88257154|88264397	circRNA_009756	7:60959606|60977347	circRNA_020638	13:149599667|149657541
circRNA_006539	GL896294.1:76434|78537	circRNA_000922	15:61722573|61729817	circRNA_003405	14:92407605|92425347	circRNA_016886	15:8512819|8570725
circRNA_008152	9:113942755|113944858	circRNA_013064	4:1198975|1206219	circRNA_008423	1:200310792|200328539	circRNA_020758	15:112090894|112148920
circRNA_013092	4:42104548|42106651	circRNA_020139	3:47576898|47584144	circRNA_000056	1:59726631|59744379	circRNA_004708	6:153334935|153393008
circRNA_016576	X:15890942|15893046	circRNA_003040	12:49362946|49370193	circRNA_002126	8:103694327|103712082	circRNA_013586	X:73952719|74010804
circRNA_013360	7:124952570|124954675	circRNA_005323	X:17788131|17795378	circRNA_003399	14:82393345|82411103	circRNA_008312	1:41655344|41713431
circRNA_011494	16:51795728|51797837	circRNA_021248	9:22403235|22410483	circRNA_000344	10:54023860|54041621	circRNA_015208	1:127972025|128030115
circRNA_002041	7:124952565|124954675	circRNA_011982	7:134300825|134308074	circRNA_008608	11:49487460|49505222	circRNA_007503	2:107044276|107102386
circRNA_003224	13:145222118|145224229	circRNA_001476	3:102273355|102280606	circRNA_006556	X:17547484|17565248	circRNA_001013	15:124662048|124720160
circRNA_003686	16:34274758|34276870	circRNA_010461	14:151205900|151213151	circRNA_007678	4:103237684|103255448	circRNA_018936	2:118250134|118308249
circRNA_012458	12:1401703|1403815	circRNA_008453	1:233195418|233202671	circRNA_004838	7:103043381|103061148	circRNA_013788	1:233128640|233186782
circRNA_016833	14:10542537|10544649	circRNA_010604	2:157463348|157470602	circRNA_010474	15:66916145|66933912	circRNA_013713	4:6908428|6966587
circRNA_010494	15:116402448|116404561	circRNA_002490	1:36705595|36712852	circRNA_017014	2:50315707|50333474	circRNA_006888	11:35045323|35103547
circRNA_012451	11:77282244|77284357	circRNA_007144	14:66224279|66231536	circRNA_013963	9:78889517|78907286	circRNA_001444	3:75724949|75783176
circRNA_002271	9:101484691|101486805	circRNA_010672	4:73714673|73721931	circRNA_014406	3:121345252|121363022	circRNA_002505	1:60892775|60951054
circRNA_004736	7:26727292|26729406	circRNA_012634	14:33300809|33308068	circRNA_015746	16:23568811|23586581	circRNA_001868	6:99489396|99547687
circRNA_017929	7:43370412|43372526	circRNA_015905	2:105108619|105115879	circRNA_015805	18:2762532|2780302	circRNA_001529	3:130810562|130868878

<table>
<tr><td>circRNA_008820</td><td>13:140998202|141000317</td><td>circRNA_010328</td><td>12:23736596|23743858</td><td>circRNA_004160</td><td>3:106269291|106287062</td><td>circRNA_005395</td><td>X:141567562|141625879</td></tr>
<tr><td>circRNA_000441</td><td>12:1401699|1403815</td><td>circRNA_003127</td><td>13:55776452|55783715</td><td>circRNA_012179</td><td>1:4653396|4671167</td><td>circRNA_006285</td><td>6:92745097|92803482</td></tr>
<tr><td>circRNA_003544</td><td>15:67058246|67060363</td><td>circRNA_002246</td><td>9:72672014|72679278</td><td>circRNA_012113</td><td>GL893609.2:60344|78120</td><td>circRNA_001475</td><td>3:101319636|101378022</td></tr>
<tr><td>circRNA_005292</td><td>JH118815.1:27051|29169</td><td>circRNA_020569</td><td>12:62428106|62435370</td><td>circRNA_018506</td><td>1:245490347|245508123</td><td>circRNA_006067</td><td>3:39564463|39622856</td></tr>
<tr><td>circRNA_009520</td><td>5:6939900|6942018</td><td>circRNA_021032</td><td>4:121753788|121761052</td><td>circRNA_012810</td><td>16:47791556|47809336</td><td>circRNA_016614</td><td>1:6417991|6476403</td></tr>
<tr><td>circRNA_000680</td><td>13:157354809|157356928</td><td>circRNA_001222</td><td>2:16112934|16120200</td><td>circRNA_006325</td><td>7:21571926|21589708</td><td>circRNA_000700</td><td>13:199361578|199420003</td></tr>
<tr><td>circRNA_016819</td><td>13:144096992|144099112</td><td>circRNA_008018</td><td>8:69704199|69711465</td><td>circRNA_020318</td><td>9:44480031|44497813</td><td>circRNA_019545</td><td>14:116563564|116621990</td></tr>
<tr><td>circRNA_009847</td><td>8:46503924|46506045</td><td>circRNA_014914</td><td>12:62428106|62435373</td><td>circRNA_014090</td><td>1:4618742|4636526</td><td>circRNA_018382</td><td>GL892628.1:25750|84201</td></tr>
<tr><td>circRNA_012102</td><td>GL892843.1:78481|80602</td><td>circRNA_002401</td><td>MT:2513|9782</td><td>circRNA_007493</td><td>2:97457124|97474912</td><td>circRNA_004192</td><td>3:120081729|120140221</td></tr>
<tr><td>circRNA_013660</td><td>1:188119162|188121283</td><td>circRNA_017876</td><td>6:18420888|18428157</td><td>circRNA_000893</td><td>15:22750457|22768247</td><td>circRNA_010636</td><td>3:113981573|114040093</td></tr>
<tr><td>circRNA_009485</td><td>4:98078591|98080713</td><td>circRNA_018193</td><td>16:51809302|51816571</td><td>circRNA_014128</td><td>1:200644823|200662616</td><td>circRNA_002467</td><td>1:16415160|16473752</td></tr>
<tr><td>circRNA_011979</td><td>7:124952553|124954675</td><td>circRNA_009540</td><td>5:49617794|49625064</td><td>circRNA_008402</td><td>1:176374364|176392159</td><td>circRNA_016445</td><td>9:31869060|31927654</td></tr>
<tr><td>circRNA_020920</td><td>2:142403818|142405941</td><td>circRNA_012967</td><td>2:122649864|122657134</td><td>circRNA_003699</td><td>16:38539919|38557717</td><td>circRNA_000024</td><td>1:18675426|18734033</td></tr>
<tr><td>circRNA_001345</td><td>2:132433336|132435460</td><td>circRNA_000026</td><td>1:18877993|18885264</td><td>circRNA_015442</td><td>12:27543412|27561213</td><td>circRNA_018013</td><td>9:148485115|148543751</td></tr>
<tr><td>circRNA_013853</td><td>15:124787561|124789687</td><td>circRNA_012339</td><td>10:15032847|15040118</td><td>circRNA_000033</td><td>1:32054762|32072573</td><td>circRNA_020288</td><td>8:63838998|63897657</td></tr>
<tr><td>circRNA_003250</td><td>13:189554196|189556323</td><td>circRNA_017558</td><td>12:12187479|12194750</td><td>circRNA_010830</td><td>8:44980268|44998089</td><td>circRNA_010229</td><td>1:204885769|204944457</td></tr>
<tr><td>circRNA_007309</td><td>16:34095143|34097270</td><td>circRNA_021192</td><td>8:36910098|36917369</td><td>circRNA_008745</td><td>13:43392302|43410124</td><td>circRNA_007194</td><td>14:136879801|136938518</td></tr>
<tr><td>circRNA_020144</td><td>3:72122816|72124943</td><td>circRNA_010356</td><td>13:25368582|25375855</td><td>circRNA_011042</td><td>1:224436134|224453958</td><td>circRNA_015196</td><td>1:110340582|110399307</td></tr>
<tr><td>circRNA_021365</td><td>X:51708716|51710844</td><td>circRNA_012216</td><td>1:64125099|64132372</td><td>circRNA_009268</td><td>2:87139525|87157353</td><td>circRNA_016577</td><td>X:19429833|19488721</td></tr>
<tr><td>circRNA_005122</td><td>9:58393955|58396087</td><td>circRNA_017649</td><td>14:122682984|122690258</td><td>circRNA_011450</td><td>15:138663710|138681538</td><td>circRNA_007999</td><td>8:39689414|39748311</td></tr>
<tr><td>circRNA_000833</td><td>14:98659351|98661484</td><td>circRNA_016777</td><td>13:26007017|26014292</td><td>circRNA_009285</td><td>2:110549603|110567432</td><td>circRNA_004646</td><td>6:109735016|109793974</td></tr>
<tr><td>circRNA_019080</td><td>5:79676712|79678845</td><td>circRNA_009689</td><td>6:112321758|112329035</td><td>circRNA_001488</td><td>3:108496197|108514028</td><td>circRNA_007628</td><td>4:19782369|19841332</td></tr>
<tr><td>circRNA_002899</td><td>11:21429243|21431377</td><td>circRNA_017129</td><td>4:134785876|134793154</td><td>circRNA_007667</td><td>4:74172060|74189892</td><td>circRNA_007012</td><td>13:83142279|83201253</td></tr>
<tr><td>circRNA_006826</td><td>10:35382114|35384249</td><td>circRNA_001646</td><td>4:128903356|128910638</td><td>circRNA_000617</td><td>13:90062337|90080170</td><td>circRNA_006899</td><td>11:75029922|75088925</td></tr>
<tr><td>circRNA_015753</td><td>16:27088302|27090437</td><td>circRNA_005713</td><td>13:131006932|131014214</td><td>circRNA_017519</td><td>10:36252996|36270829</td><td>circRNA_005390</td><td>X:127682865|127741869</td></tr>
<tr><td>circRNA_012156</td><td>X:67385337|67387473</td><td>circRNA_017995</td><td>9:72672014|72679297</td><td>circRNA_005158</td><td>9:116165727|116183562</td><td>circRNA_002936</td><td>11:74618777|74677794</td></tr>
<tr><td>circRNA_006915</td><td>12:16876107|16878244</td><td>circRNA_016250</td><td>6:108048133|108055419</td><td>circRNA_011833</td><td>5:86274310|86292147</td><td>circRNA_008714</td><td>13:16467579|16526599</td></tr>
<tr><td>circRNA_009620</td><td>6:25979327|25981467</td><td>circRNA_006897</td><td>11:69873672|69880966</td><td>circRNA_021294</td><td>9:138385495|138403334</td><td>circRNA_011628</td><td>2:118506457|118565484</td></tr>
<tr><td>circRNA_014564</td><td>9:112333151|112335291</td><td>circRNA_009030</td><td>15:86564756|86572050</td><td>circRNA_007376</td><td>18:1445459|1463300</td><td>circRNA_021296</td><td>9:140018765|140077848</td></tr>
<tr><td>circRNA_017357</td><td>GL892728.1:12123|14264</td><td>circRNA_010183</td><td>1:60938694|60945988</td><td>circRNA_013150</td><td>5:20837257|20855099</td><td>circRNA_000209</td><td>1:227644106|227703212</td></tr>
<tr><td>circRNA_014769</td><td>10:11694054|11696196</td><td>circRNA_016634</td><td>1:89808237|89815531</td><td>circRNA_006081</td><td>3:81201375|81219219</td><td>circRNA_016099</td><td>4:110255147|110314311</td></tr>
<tr><td>circRNA_010560</td><td>2:17103567|17105711</td><td>circRNA_019165</td><td>7:62888401|62895695</td><td>circRNA_018920</td><td>2:83015788|83033639</td><td>circRNA_010770</td><td>6:137802513|137861689</td></tr>
<tr><td>circRNA_015799</td><td>17:57006128|57008272</td><td>circRNA_016779</td><td>13:32203291|32210587</td><td>circRNA_017974</td><td>8:143273296|143291155</td><td>circRNA_017241</td><td>7:117050907|117110099</td></tr>
<tr><td>circRNA_018674</td><td>13:155932248|155934393</td><td>circRNA_002438</td><td>X:116389266|116396564</td><td>circRNA_004704</td><td>6:150861612|150879474</td><td>circRNA_019427</td><td>10:46719867|46779066</td></tr>
<tr><td>circRNA_019166</td><td>7:65201906|65204052</td><td>circRNA_004039</td><td>2:148156100|148163399</td><td>circRNA_009292</td><td>2:121534782|121552644</td><td>circRNA_000017</td><td>1:17075533|17134778</td></tr>
<tr><td>circRNA_020046</td><td>14:114876644|114878790</td><td>circRNA_021333</td><td>GL896221.1:30399|37699</td><td>circRNA_005029</td><td>8:144641851|144659719</td><td>circRNA_005660</td><td>13:33961610|34020867</td></tr>
<tr><td>circRNA_004918</td><td>8:45932892|45935039</td><td>circRNA_004418</td><td>5:23676827|23684128</td><td>circRNA_010845</td><td>8:89424301|89442169</td><td>circRNA_009682</td><td>6:101745126|101804423</td></tr>
<tr><td>circRNA_013596</td><td>X:127334137|127336284</td><td>circRNA_004215</td><td>3:135328996|135336298</td><td>circRNA_021370</td><td>X:67430148|67448018</td><td>circRNA_003604</td><td>15:112201333|112260631</td></tr>
<tr><td>circRNA_020776</td><td>16:21419644|21421793</td><td>circRNA_007205</td><td>14:145686684|145693987</td><td>circRNA_021138</td><td>6:146105698|146123569</td><td>circRNA_021153</td><td>7:24626858|24686216</td></tr>
<tr><td>circRNA_004042</td><td>2:149779440|149781590</td><td>circRNA_001340</td><td>2:128479853|128487157</td><td>circRNA_006704</td><td>1:154649289|154667163</td><td>circRNA_009734</td><td>7:24627308|24686711</td></tr>
<tr><td>circRNA_009395</td><td>3:104207532|104209684</td><td>circRNA_019018</td><td>4:19659157|19666461</td><td>circRNA_016053</td><td>4:20397252|20415126</td><td>circRNA_015663</td><td>14:120786139|120845549</td></tr>
<tr><td>circRNA_013519</td><td>GL893738.1:40878|43030</td><td>circRNA_010352</td><td>13:14238842|14246148</td><td>circRNA_007564</td><td>3:76306490|76324375</td><td>circRNA_003902</td><td>2:41825697|41885122</td></tr>
<tr><td>circRNA_015138</td><td>1:11290049|11292201</td><td>circRNA_005977</td><td>2:43993047|44000355</td><td>circRNA_018731</td><td>14:60119758|60137643</td><td>circRNA_011352</td><td>14:105105415|105164869</td></tr>
<tr><td>circRNA_016704</td><td>1:299799482|299801634</td><td>circRNA_020429</td><td>1:179189037|179196345</td><td>circRNA_020457</td><td>1:227108368|227126254</td><td>circRNA_009804</td><td>7:109814818|109874274</td></tr>
<tr><td>circRNA_017285</td><td>8:91929412|91931565</td><td>circRNA_018978</td><td>3:61512818|61520129</td><td>circRNA_009702</td><td>6:143689203|143707093</td><td>circRNA_004460</td><td>5:70588009|70647555</td></tr>
<tr><td>circRNA_019228</td><td>8:108964837|108966990</td><td>circRNA_000929</td><td>15:67053051|67060363</td><td>circRNA_016744</td><td>11:84629870|84647764</td><td>circRNA_007108</td><td>14:32917255|32976823</td></tr>
<tr><td>circRNA_011577</td><td>2:30381639|30383793</td><td>circRNA_002514</td><td>1:63741389|63748701</td><td>circRNA_013522</td><td>GL893884.1:23405|41304</td><td>circRNA_009438</td><td>4:31337248|31396823</td></tr>
<tr><td>circRNA_012924</td><td>2:52017822|52019977</td><td>circRNA_003571</td><td>15:86607064|86614377</td><td>circRNA_009600</td><td>5:105247263|105265163</td><td>circRNA_015846</td><td>18:57690667|57750266</td></tr>
<tr><td>circRNA_000857</td><td>14:132243465|132245621</td><td>circRNA_016327</td><td>7:124815434|124822749</td><td>circRNA_002342</td><td>GL894055.2:52153|70058</td><td>circRNA_017707</td><td>16:33999307|34058931</td></tr>
<tr><td>circRNA_004025</td><td>2:140344565|140346722</td><td>circRNA_020683</td><td>14:58796714|58804030</td><td>circRNA_018994</td><td>3:97935477|97953389</td><td>circRNA_015993</td><td>3:75984449|76044136</td></tr>
<tr><td>circRNA_015699</td><td>15:66961909|66964066</td><td>circRNA_005269</td><td>GL895957.1:33683|41005</td><td>circRNA_010913</td><td>GL893259.1:18126|36041</td><td>circRNA_005856</td><td>15:105424453|105484194</td></tr>
<tr><td>circRNA_012360</td><td>10:37675626|37677784</td><td>circRNA_016311</td><td>7:94525795|94533117</td><td>circRNA_007220</td><td>15:36329183|36347101</td><td>circRNA_020360</td><td>X:131493485|131553229</td></tr>
</table>

<table>
<tr><td>circRNA_014958</td><td>1:34824222|34826380</td><td>circRNA_002681</td><td>1:208264642|208271965</td><td>circRNA_002705</td><td>1:244832039|244849964</td><td>circRNA_015269</td><td>1:229186577|229246387</td></tr>
<tr><td>circRNA_020385</td><td>1:62688792|62690950</td><td>circRNA_010725</td><td>5:99055437|99062760</td><td>circRNA_012003</td><td>8:58335316|58353243</td><td>circRNA_012750</td><td>15:107266814|107326659</td></tr>
<tr><td>circRNA_001055</td><td>16:36914607|36916766</td><td>circRNA_008429</td><td>1:200790738|200798067</td><td>circRNA_010766</td><td>6:136000651|136018579</td><td>circRNA_010419</td><td>14:8637132|8696994</td></tr>
<tr><td>circRNA_008848</td><td>13:199473946|199476105</td><td>circRNA_008634</td><td>12:11470995|11478325</td><td>circRNA_001871</td><td>6:99529757|99547687</td><td>circRNA_004073</td><td>3:30119792|30179656</td></tr>
<tr><td>circRNA_019718</td><td>5:23083310|23085469</td><td>circRNA_014469</td><td>6:52342736|52350067</td><td>circRNA_018754</td><td>14:114872934|114890868</td><td>circRNA_007698</td><td>4:133935391|133995278</td></tr>
<tr><td>circRNA_003530</td><td>15:61682878|61685041</td><td>circRNA_008038</td><td>8:103514407|103521741</td><td>circRNA_011092</td><td>10:29321805|29339758</td><td>circRNA_001638</td><td>4:117897403|117957301</td></tr>
<tr><td>circRNA_021220</td><td>8:103914218|103916383</td><td>circRNA_012519</td><td>13:34909325|34916661</td><td>circRNA_006142</td><td>4:48122277|48140232</td><td>circRNA_019579</td><td>15:146201175|146261100</td></tr>
<tr><td>circRNA_018082</td><td>1:105927964|105930130</td><td>circRNA_020710</td><td>14:123441978|123449315</td><td>circRNA_018393</td><td>JH118963.1:42907|60866</td><td>circRNA_012042</td><td>9:10296782|10356709</td></tr>
<tr><td>circRNA_005190</td><td>9:138815139|138817306</td><td>circRNA_002803</td><td>10:35025752|35033090</td><td>circRNA_015739</td><td>15:157056216|157074177</td><td>circRNA_000271</td><td>1:311287304|311347236</td></tr>
<tr><td>circRNA_015925</td><td>2:130854885|130857053</td><td>circRNA_018399</td><td>X:130554947|130562285</td><td>circRNA_004006</td><td>2:128768970|128786933</td><td>circRNA_001046</td><td>16:29621190|29681127</td></tr>
<tr><td>circRNA_011799</td><td>5:3337672|3339841</td><td>circRNA_002024</td><td>7:99001662|99009001</td><td>circRNA_017900</td><td>6:92994234|93012200</td><td>circRNA_000660</td><td>13:140920980|140980962</td></tr>
<tr><td>circRNA_015042</td><td>6:125758894|125761063</td><td>circRNA_010240</td><td>1:233126038|233133377</td><td>circRNA_015281</td><td>1:242142862|242160834</td><td>circRNA_014079</td><td>1:17075533|17135524</td></tr>
<tr><td>circRNA_005329</td><td>X:43423641|43425811</td><td>circRNA_006617</td><td>1:23213758|23221098</td><td>circRNA_009273</td><td>2:90148542|90166516</td><td>circRNA_005534</td><td>10:2811299|2871377</td></tr>
<tr><td>circRNA_009926</td><td>9:22402298|22404468</td><td>circRNA_020373</td><td>1:32082085|32089431</td><td>circRNA_020272</td><td>7:116372672|116390648</td><td>circRNA_020160</td><td>4:18415654|18475736</td></tr>
<tr><td>circRNA_017327</td><td>9:51379089|51381260</td><td>circRNA_021190</td><td>8:32602740|32610089</td><td>circRNA_008258</td><td>X:70646172|70664153</td><td>circRNA_018745</td><td>14:81355508|81415598</td></tr>
<tr><td>circRNA_013102</td><td>4:73730788|73732960</td><td>circRNA_000558</td><td>13:33954373|33961723</td><td>circRNA_018480</td><td>1:195011671|195029660</td><td>circRNA_017191</td><td>6:103828536|103888664</td></tr>
<tr><td>circRNA_014768</td><td>1:303196524|303198696</td><td>circRNA_003156</td><td>13:84626456|84633809</td><td>circRNA_008617</td><td>11:69821718|69839712</td><td>circRNA_007100</td><td>14:21515998|21576174</td></tr>
<tr><td>circRNA_021355</td><td>X:21719134|21721310</td><td>circRNA_016609</td><td>X:126963525|126970879</td><td>circRNA_010042</td><td>GL892430.1:44064|62058</td><td>circRNA_005174</td><td>9:129664193|129724374</td></tr>
<tr><td>circRNA_006578</td><td>X:103700117|103702294</td><td>circRNA_010210</td><td>1:146238254|146245609</td><td>circRNA_000397</td><td>11:19726528|19744523</td><td>circRNA_000365</td><td>10:76245922|76306111</td></tr>
<tr><td>circRNA_002461</td><td>1:13630780|13632958</td><td>circRNA_011585</td><td>2:47678872|47686227</td><td>circRNA_013288</td><td>6:148040878|148058873</td><td>circRNA_006112</td><td>3:120081729|120141921</td></tr>
<tr><td>circRNA_005712</td><td>13:130133705|130135883</td><td>circRNA_020071</td><td>16:3548202|3555557</td><td>circRNA_010456</td><td>14:131390060|131408058</td><td>circRNA_010719</td><td>5:87192177|87252405</td></tr>
<tr><td>circRNA_014132</td><td>1:207015843|207018021</td><td>circRNA_004832</td><td>7:100228877|100236236</td><td>circRNA_004372</td><td>4:132005794|132023793</td><td>circRNA_018335</td><td>7:73042261|73102497</td></tr>
<tr><td>circRNA_017689</td><td>15:129637141|129639319</td><td>circRNA_013750</td><td>15:124782328|124789687</td><td>circRNA_019089</td><td>5:109523873|109541874</td><td>circRNA_016688</td><td>1:249858161|249918475</td></tr>
<tr><td>circRNA_009578</td><td>5:82634216|82636395</td><td>circRNA_001379</td><td>3:5184976|5192337</td><td>circRNA_005322</td><td>X:17732218|17750223</td><td>circRNA_015515</td><td>13:66516811|66577148</td></tr>
<tr><td>circRNA_012006</td><td>8:76070362|76072541</td><td>circRNA_010520</td><td>17:15485334|15488101</td><td>circRNA_008227</td><td>JH118602.1:45202|63209</td><td>circRNA_014204</td><td>13:83142279|83202617</td></tr>
<tr><td>circRNA_020213</td><td>6:47018476|47020655</td><td>circRNA_013257</td><td>6:88477642|88480409</td><td>circRNA_017202</td><td>6:132195677|132213693</td><td>circRNA_014181</td><td>12:34577207|34637547</td></tr>
<tr><td>circRNA_010369</td><td>13:43483204|43485384</td><td>circRNA_019314</td><td>GL894584.1:40244|43011</td><td>circRNA_002280</td><td>9:114868023|114886040</td><td>circRNA_009224</td><td>2:20100182|20160570</td></tr>
<tr><td>circRNA_014667</td><td>15:104092863|104095043</td><td>circRNA_009680</td><td>6:101010009|101012777</td><td>circRNA_004478</td><td>5:81375491|81393509</td><td>circRNA_004472</td><td>5:76330931|76391339</td></tr>
<tr><td>circRNA_011085</td><td>10:17369643|17371825</td><td>circRNA_013960</td><td>9:37673213|37675981</td><td>circRNA_005522</td><td>1:287919930|287937948</td><td>circRNA_020455</td><td>1:224984471|225044886</td></tr>
<tr><td>circRNA_013504</td><td>9:138815124|138817306</td><td>circRNA_009402</td><td>3:113906292|113909062</td><td>circRNA_010690</td><td>5:4771043|4789070</td><td>circRNA_012157</td><td>X:70438343|70498760</td></tr>
<tr><td>circRNA_018708</td><td>14:30976192|30978374</td><td>circRNA_002729</td><td>1:265966523|265969297</td><td>circRNA_014750</td><td>GL894855.1:11155|29194</td><td>circRNA_012344</td><td>10:17067380|17127798</td></tr>
<tr><td>circRNA_005838</td><td>15:61883151|61885334</td><td>circRNA_007703</td><td>4:137355963|137358737</td><td>circRNA_017790</td><td>3:45880026|45898065</td><td>circRNA_018281</td><td>5:13361763|13422184</td></tr>
<tr><td>circRNA_006361</td><td>7:103037110|103039293</td><td>circRNA_016962</td><td>17:49169026|49171800</td><td>circRNA_007016</td><td>13:86985202|87003242</td><td>circRNA_003397</td><td>14:81920776|81981214</td></tr>
<tr><td>circRNA_016642</td><td>1:123403065|123405248</td><td>circRNA_010505</td><td>16:45812938|45815714</td><td>circRNA_018756</td><td>14:115739339|115757379</td><td>circRNA_013627</td><td>13:190906188|190966639</td></tr>
<tr><td>circRNA_017824</td><td>4:22450256|22452440</td><td>circRNA_005607</td><td>11:84664584|84667363</td><td>circRNA_010721</td><td>5:87303583|87321627</td><td>circRNA_003035</td><td>12:47600223|47660700</td></tr>
<tr><td>circRNA_020825</td><td>18:5402373|5404557</td><td>circRNA_011174</td><td>12:37175753|37178532</td><td>circRNA_001742</td><td>5:73490736|73508782</td><td>circRNA_019756</td><td>6:43045717|43106238</td></tr>
<tr><td>circRNA_019659</td><td>2:146108635|146110820</td><td>circRNA_019397</td><td>1:212203332|212206112</td><td>circRNA_006232</td><td>5:87303583|87321630</td><td>circRNA_010975</td><td>1:84560341|84620883</td></tr>
<tr><td>circRNA_004492</td><td>5:91755963|91758149</td><td>circRNA_010658</td><td>4:39870935|39873716</td><td>circRNA_004896</td><td>8:32603610|32621660</td><td>circRNA_019934</td><td>1:200363897|200424446</td></tr>
<tr><td>circRNA_014064</td><td>JH118404.1:116644|118833</td><td>circRNA_015249</td><td>1:208269182|208271965</td><td>circRNA_020415</td><td>1:143231694|143249745</td><td>circRNA_001866</td><td>6:99256999|99317549</td></tr>
<tr><td>circRNA_018234</td><td>2:84213514|84215703</td><td>circRNA_006148</td><td>4:61932160|61934946</td><td>circRNA_003094</td><td>13:33157395|33175447</td><td>circRNA_015851</td><td>18:59628324|59688893</td></tr>
<tr><td>circRNA_006606</td><td>1:16492760|16494951</td><td>circRNA_014788</td><td>13:209723825|209726611</td><td>circRNA_015335</td><td>10:33579419|33597479</td><td>circRNA_018201</td><td>17:55180059|55240628</td></tr>
<tr><td>circRNA_014578</td><td>GL892916.1:18682|20873</td><td>circRNA_013745</td><td>1:267591642|267594430</td><td>circRNA_006882</td><td>11:26247793|26265854</td><td>circRNA_010166</td><td>1:16779138|16839716</td></tr>
<tr><td>circRNA_001640</td><td>4:117915467|117917659</td><td>circRNA_013009</td><td>3:56863189|56865978</td><td>circRNA_003936</td><td>2:81132642|81150704</td><td>circRNA_019183</td><td>7:97058335|97118937</td></tr>
<tr><td>circRNA_010016</td><td>9:135963364|135965556</td><td>circRNA_015173</td><td>1:62793096|62795885</td><td>circRNA_011061</td><td>1:270329811|270347874</td><td>circRNA_013911</td><td>4:40283338|40343945</td></tr>
<tr><td>circRNA_018156</td><td>14:53891911|53894103</td><td>circRNA_002142</td><td>8:125015138|125017928</td><td>circRNA_016726</td><td>10:58011567|58029635</td><td>circRNA_009664</td><td>6:88789118|88849787</td></tr>
<tr><td>circRNA_003451</td><td>14:131872215|131874408</td><td>circRNA_007757</td><td>5:70411450|70414240</td><td>circRNA_005651</td><td>13:24353502|24371577</td><td>circRNA_014335</td><td>17:49887473|49948154</td></tr>
<tr><td>circRNA_020740</td><td>15:74445099|74447292</td><td>circRNA_016671</td><td>1:202612264|202615054</td><td>circRNA_003825</td><td>18:18602505|18620583</td><td>circRNA_019901</td><td>1:17075533|17136233</td></tr>
<tr><td>circRNA_021022</td><td>4:75065079|75067272</td><td>circRNA_011663</td><td>3:19866529|19869320</td><td>circRNA_000237</td><td>1:270329795|270347874</td><td>circRNA_015959</td><td>3:29276416|29337133</td></tr>
<tr><td>circRNA_011355</td><td>14:110567233|110569427</td><td>circRNA_014617</td><td>1:282147236|282150027</td><td>circRNA_004525</td><td>6:12077068|12095147</td><td>circRNA_009922</td><td>9:19259967|19320699</td></tr>
<tr><td>circRNA_002056</td><td>8:4258670|4260865</td><td>circRNA_015593</td><td>14:10462923|10465714</td><td>circRNA_011546</td><td>18:21597334|21615413</td><td>circRNA_012915</td><td>2:31907368|31968100</td></tr>
<tr><td>circRNA_018951</td><td>2:143695204|143697399</td><td>circRNA_009253</td><td>2:61882308|61885100</td><td>circRNA_001503</td><td>3:114476160|114494241</td><td>circRNA_008251</td><td>X:46443566|46504346</td></tr>
<tr><td>circRNA_019377</td><td>1:129156159|129158355</td><td>circRNA_004594</td><td>6:85062798|85065591</td><td>circRNA_000091</td><td>1:118360630|118378712</td><td>circRNA_016874</td><td>14:116642719|116703512</td></tr>
</table>

<table>
<tr><td>circRNA_011358</td><td>14:114543856|114546053</td><td>circRNA_007067</td><td>13:145987809|145990602</td><td>circRNA_007428</td><td>2:20519951|20538034</td><td>circRNA_002492</td><td>1:40891902|40952696</td></tr>
<tr><td>circRNA_000113</td><td>1:132828553|132830751</td><td>circRNA_009579</td><td>5:82860913|82863706</td><td>circRNA_001502</td><td>3:114476157|114494241</td><td>circRNA_000224</td><td>1:249850891|249911706</td></tr>
<tr><td>circRNA_000566</td><td>13:34942344|34944542</td><td>circRNA_002632</td><td>1:181133477|181136271</td><td>circRNA_018827</td><td>16:36889845|36907929</td><td>circRNA_018957</td><td>3:9267608|9328457</td></tr>
<tr><td>circRNA_018096</td><td>1:257754306|257756506</td><td>circRNA_007823</td><td>6:64288025|64290820</td><td>circRNA_010553</td><td>2:3016339|3034430</td><td>circRNA_017495</td><td>1:242725331|242786202</td></tr>
<tr><td>circRNA_004533</td><td>6:16609864|16612066</td><td>circRNA_020688</td><td>14:68175267|68178062</td><td>circRNA_018939</td><td>2:120983371|121001462</td><td>circRNA_008493</td><td>1:284448584|284509515</td></tr>
<tr><td>circRNA_007576</td><td>3:92528897|92531099</td><td>circRNA_010534</td><td>18:1417740|1420536</td><td>circRNA_020681</td><td>14:51638310|51656404</td><td>circRNA_017981</td><td>9:22123762|22184702</td></tr>
<tr><td>circRNA_017077</td><td>3:92382600|92384802</td><td>circRNA_006584</td><td>X:115970997|115973795</td><td>circRNA_016374</td><td>8:71582612|71600707</td><td>circRNA_013168</td><td>5:49928478|49989511</td></tr>
<tr><td>circRNA_006953</td><td>12:62413971|62416175</td><td>circRNA_003115</td><td>13:43392088|43394888</td><td>circRNA_013175</td><td>5:61457868|61475967</td><td>circRNA_001505</td><td>3:118042427|118103463</td></tr>
<tr><td>circRNA_010515</td><td>16:78183056|78185260</td><td>circRNA_008350</td><td>1:105785018|105787819</td><td>circRNA_019421</td><td>10:29342271|29360371</td><td>circRNA_008907</td><td>14:52810347|52871390</td></tr>
<tr><td>circRNA_020316</td><td>9:40615089|40617293</td><td>circRNA_001364</td><td>2:147049942|147052745</td><td>circRNA_011077</td><td>1:310030060|310048161</td><td>circRNA_015405</td><td>11:70907464|70968532</td></tr>
<tr><td>circRNA_000004</td><td>1:10817302|10819507</td><td>circRNA_002319</td><td>GL892407.1:24079|26882</td><td>circRNA_007372</td><td>17:59438248|59456350</td><td>circRNA_016582</td><td>X:23586368|23647447</td></tr>
<tr><td>circRNA_005426</td><td>1:75046188|75048394</td><td>circRNA_003735</td><td>16:78113134|78115937</td><td>circRNA_000837</td><td>14:112935251|112953354</td><td>circRNA_006015</td><td>2:91169756|91230855</td></tr>
<tr><td>circRNA_008077</td><td>9:8944659|8946866</td><td>circRNA_012274</td><td>1:176789554|176792357</td><td>circRNA_015437</td><td>12:23732147|23750252</td><td>circRNA_007749</td><td>5:49928478|49989587</td></tr>
<tr><td>circRNA_013639</td><td>6:39464092|39466299</td><td>circRNA_015608</td><td>14:48619065|48621868</td><td>circRNA_009690</td><td>6:118035375|118053482</td><td>circRNA_018243</td><td>2:145758608|145819737</td></tr>
<tr><td>circRNA_014276</td><td>15:37967726|37969934</td><td>circRNA_001999</td><td>7:73120980|73123784</td><td>circRNA_002084</td><td>8:43550245|43568354</td><td>circRNA_006054</td><td>3:4820089|4881241</td></tr>
<tr><td>circRNA_002596</td><td>1:142576039|142578248</td><td>circRNA_007077</td><td>13:167100656|167103460</td><td>circRNA_006524</td><td>GL893689.1:63096|81205</td><td>circRNA_018058</td><td>X:86132408|86193572</td></tr>
<tr><td>circRNA_016800</td><td>13:76189587|76191796</td><td>circRNA_002380</td><td>GL896504.1:52653|55460</td><td>circRNA_020236</td><td>6:137379532|137397644</td><td>circRNA_010430</td><td>14:31918604|31979781</td></tr>
<tr><td>circRNA_013441</td><td>9:22334946|22337156</td><td>circRNA_008674</td><td>12:44665244|44668051</td><td>circRNA_015990</td><td>3:74760760|74778873</td><td>circRNA_007337</td><td>17:5356805|5418002</td></tr>
<tr><td>circRNA_000297</td><td>10:24484010|24486223</td><td>circRNA_010381</td><td>13:90760724|90763531</td><td>circRNA_017912</td><td>6:137387063|137405178</td><td>circRNA_015906</td><td>2:105156230|105217494</td></tr>
<tr><td>circRNA_010194</td><td>1:93294017|93296230</td><td>circRNA_004804</td><td>7:73258086|73260894</td><td>circRNA_001413</td><td>3:45698129|45716254</td><td>circRNA_012818</td><td>16:59176203|59237476</td></tr>
<tr><td>circRNA_014337</td><td>17:53885029|53887242</td><td>circRNA_009258</td><td>2:72278982|72281790</td><td>circRNA_001969</td><td>7:54511993|54530118</td><td>circRNA_017838</td><td>4:89318648|89379922</td></tr>
<tr><td>circRNA_017120</td><td>4:103264898|103267113</td><td>circRNA_017867</td><td>5:91490970|91493778</td><td>circRNA_006047</td><td>2:142526258|142544386</td><td>circRNA_008989</td><td>15:34770888|34832166</td></tr>
<tr><td>circRNA_005224</td><td>GL892841.1:31705|33921</td><td>circRNA_008132</td><td>9:79138144|79140954</td><td>circRNA_007685</td><td>4:113275739|113293869</td><td>circRNA_014868</td><td>7:109722597|109783904</td></tr>
<tr><td>circRNA_018160</td><td>14:71918903|71921119</td><td>circRNA_000443</td><td>12:5475167|5477978</td><td>circRNA_003499</td><td>15:19245136|19263276</td><td>circRNA_015340</td><td>10:37411679|37473029</td></tr>
<tr><td>circRNA_001082</td><td>17:5339456|5341673</td><td>circRNA_001404</td><td>3:34704652|34707464</td><td>circRNA_009634</td><td>6:52578943|52597083</td><td>circRNA_020189</td><td>4:142025103|142086476</td></tr>
<tr><td>circRNA_005446</td><td>1:132967048|132969266</td><td>circRNA_003760</td><td>17:31723719|31726533</td><td>circRNA_013579</td><td>X:52869732|52887877</td><td>circRNA_015151</td><td>1:24730806|24792185</td></tr>
<tr><td>circRNA_016352</td><td>8:41671944|41674162</td><td>circRNA_003345</td><td>14:44201888|44204705</td><td>circRNA_007893</td><td>7:112298|130444</td><td>circRNA_019198</td><td>8:29249330|29310723</td></tr>
<tr><td>circRNA_000626</td><td>13:102346239|102348459</td><td>circRNA_000781</td><td>14:58489341|58492159</td><td>circRNA_011030</td><td>1:195112533|195130686</td><td>circRNA_015908</td><td>2:107040975|107102386</td></tr>
<tr><td>circRNA_005044</td><td>9:7134810|7137030</td><td>circRNA_013071</td><td>4:15227218|15230037</td><td>circRNA_001982</td><td>7:63538957|63557113</td><td>circRNA_020219</td><td>6:52531995|52593444</td></tr>
<tr><td>circRNA_006073</td><td>3:49146846|49149067</td><td>circRNA_021047</td><td>5:29853467|29856286</td><td>circRNA_008687</td><td>12:52763999|52782155</td><td>circRNA_008696</td><td>12:62856130|62917605</td></tr>
<tr><td>circRNA_013610</td><td>2:154056528|154058749</td><td>circRNA_002245</td><td>9:72672014|72674834</td><td>circRNA_005854</td><td>15:94327571|94345728</td><td>circRNA_009204</td><td>18:53737307|53798829</td></tr>
<tr><td>circRNA_021252</td><td>9:29682317|29684539</td><td>circRNA_011035</td><td>1:202813229|202816050</td><td>circRNA_004713</td><td>7:10291894|10310057</td><td>circRNA_013033</td><td>3:97544470|97605999</td></tr>
<tr><td>circRNA_010562</td><td>2:27609102|27611325</td><td>circRNA_021249</td><td>9:22568662|22571483</td><td>circRNA_001491</td><td>3:109787455|109805619</td><td>circRNA_010493</td><td>15:115994299|116055840</td></tr>
<tr><td>circRNA_013949</td><td>8:46584125|46586348</td><td>circRNA_004325</td><td>4:101880026|101882848</td><td>circRNA_002819</td><td>10:46597662|46615828</td><td>circRNA_009426</td><td>3:135341139|135402694</td></tr>
<tr><td>circRNA_018593</td><td>12:25022937|25025161</td><td>circRNA_000780</td><td>14:58489334|58492159</td><td>circRNA_008333</td><td>1:85792894|85811061</td><td>circRNA_000343</td><td>10:53682211|53743854</td></tr>
<tr><td>circRNA_006204</td><td>5:50053136|50055362</td><td>circRNA_019129</td><td>6:135987041|135989866</td><td>circRNA_009004</td><td>15:53419160|53437329</td><td>circRNA_015231</td><td>1:191515566|191577209</td></tr>
<tr><td>circRNA_012466</td><td>12:14691671|14693897</td><td>circRNA_008949</td><td>14:116999005|117001831</td><td>circRNA_009760</td><td>7:63538939|63557113</td><td>circRNA_007038</td><td>13:108990403|109052118</td></tr>
<tr><td>circRNA_001027</td><td>15:147864600|147866827</td><td>circRNA_016269</td><td>7:10562510|10565336</td><td>circRNA_015214</td><td>1:136483458|136501633</td><td>circRNA_020565</td><td>12:51731271|51793096</td></tr>
<tr><td>circRNA_005298</td><td>JH118940.1:100050|102277</td><td>circRNA_020757</td><td>15:106899116|106901942</td><td>circRNA_018665</td><td>13:141239521|141257696</td><td>circRNA_000875</td><td>14:145928322|145990152</td></tr>
<tr><td>circRNA_008987</td><td>15:22575140|22577367</td><td>circRNA_012791</td><td>16:21366358|21369187</td><td>circRNA_003326</td><td>14:31377807|31395983</td><td>circRNA_005170</td><td>9:128129294|128191259</td></tr>
<tr><td>circRNA_006644</td><td>1:83291219|83293447</td><td>circRNA_017945</td><td>7:128846360|128849189</td><td>circRNA_010774</td><td>6:154523123|154541301</td><td>circRNA_014906</td><td>1:21222171|21284136</td></tr>
<tr><td>circRNA_006755</td><td>1:231528885|231531113</td><td>circRNA_014536</td><td>8:122432854|122435684</td><td>circRNA_011556</td><td>18:49455064|49473243</td><td>circRNA_008123</td><td>9:62346713|62408681</td></tr>
<tr><td>circRNA_003928</td><td>2:79596775|79599004</td><td>circRNA_006042</td><td>2:136451195|136454027</td><td>circRNA_016742</td><td>11:72345491|72363670</td><td>circRNA_003225</td><td>13:145860014|145922001</td></tr>
<tr><td>circRNA_014640</td><td>13:91072247|91074476</td><td>circRNA_005623</td><td>12:31697144|31699978</td><td>circRNA_000662</td><td>13:141239521|141257701</td><td>circRNA_012055</td><td>9:36072628|36134637</td></tr>
<tr><td>circRNA_015323</td><td>10:8244815|8247044</td><td>circRNA_019538</td><td>14:78243180|78246015</td><td>circRNA_014111</td><td>1:123275194|123293376</td><td>circRNA_003208</td><td>13:142522109|142584123</td></tr>
<tr><td>circRNA_017501</td><td>1:266604253|266606482</td><td>circRNA_002978</td><td>12:18788148|18790985</td><td>circRNA_015660</td><td>14:116579352|116597536</td><td>circRNA_005971</td><td>2:27347578|27409648</td></tr>
<tr><td>circRNA_002605</td><td>1:144729933|144732163</td><td>circRNA_004774</td><td>7:60959606|60962443</td><td>circRNA_010300</td><td>11:495509|513694</td><td>circRNA_005169</td><td>9:128129181|128191259</td></tr>
<tr><td>circRNA_016597</td><td>X:73967201|73969431</td><td>circRNA_021181</td><td>7:121229266|121232103</td><td>circRNA_020207</td><td>6:293425|311611</td><td>circRNA_019862</td><td>9:145637577|145699665</td></tr>
<tr><td>circRNA_000357</td><td>10:63281102|63283334</td><td>circRNA_011294</td><td>13:209982741|209985579</td><td>circRNA_000396</td><td>11:19718544|19736732</td><td>circRNA_001766</td><td>5:97801784|97863905</td></tr>
<tr><td>circRNA_012111</td><td>GL893425.2:39777|42010</td><td>circRNA_013971</td><td>GL893841.1:29395|32233</td><td>circRNA_003689</td><td>16:36907823|36926011</td><td>circRNA_014839</td><td>4:133935391|133997583</td></tr>
<tr><td>circRNA_012848</td><td>17:57269639|57271872</td><td>circRNA_014242</td><td>14:42338806|42341646</td><td>circRNA_018921</td><td>2:83206943|83225141</td><td>circRNA_001656</td><td>4:133935391|133997589</td></tr>
<tr><td>circRNA_013557</td><td>MT:1398|3632</td><td>circRNA_004856</td><td>7:123629783|123632624</td><td>circRNA_015833</td><td>18:32288970|32307169</td><td>circRNA_019541</td><td>14:100476021|100538254</td></tr>
</table>

circRNA_006989	13:42295832|42298067	circRNA_015867	2:17221418|17224259	circRNA_016963	17:49169026|49187227	circRNA_013048	3:113976993|114039242
circRNA_009492	4:112825591|112827827	circRNA_009250	2:60840258|60843100	circRNA_020639	13:155923849|155942051	circRNA_019388	1:176679869|176742136
circRNA_009867	8:90494255|90496491	circRNA_018325	6:148098228|148101071	circRNA_008427	1:200703767|200721973	circRNA_010593	2:130871164|130933457
circRNA_012628	14:31286863|31289099	circRNA_000059	1:62359384|62362228	circRNA_012717	15:54564541|54582748	circRNA_018933	2:112456647|112518956
circRNA_016803	13:90144187|90146423	circRNA_003508	15:28820398|28823242	circRNA_020948	3:36002268|36020475	circRNA_005155	9:115756518|115818856
circRNA_003919	2:60064686|60066924	circRNA_012555	13:79310756|79313600	circRNA_013741	GL893259.1:8490|26701	circRNA_008414	1:183773091|183835431
circRNA_013443	9:30486127|30488366	circRNA_000770	14:43179929|43182775	circRNA_005432	1:94559522|94577735	circRNA_008626	11:76339151|76401491
circRNA_008954	14:120786139|120788379	circRNA_001761	5:92098435|92101281	circRNA_005097	9:37678422|37696637	circRNA_012736	15:86609169|86671532
circRNA_009728	7:16831959|16834199	circRNA_008416	1:186656379|186659227	circRNA_014813	2:3011482|3029700	circRNA_000294	10:18187787|18250156
circRNA_012217	1:64737843|64740083	circRNA_019581	15:157399187|157402037	circRNA_014089	X:61016381|61034602	circRNA_017922	7:82092|144469
circRNA_019968	11:2275318|2277558	circRNA_008492	1:284448584|284451435	circRNA_015972	3:57275578|57293800	circRNA_019949	1:284447133|284509515
circRNA_014308	16:23641967|23644209	circRNA_003316	14:30050907|30053760	circRNA_009811	7:128395106|128413331	circRNA_005501	1:229233921|229296319
circRNA_003666	16:23393877|23396120	circRNA_019511	13:209990827|209993681	circRNA_018752	14:114803451|114821678	circRNA_017810	3:122016272|122078774
circRNA_004726	7:16831954|16834199	circRNA_001793	6:39337935|39340790	circRNA_015555	13:146898748|146916978	circRNA_020251	7:24642345|24704878
circRNA_009672	6:96859272|96861518	circRNA_003097	13:34824430|34827287	circRNA_013195	5:88187371|88205602	circRNA_013134	4:135644679|135707221
circRNA_017433	1:62630181|62632427	circRNA_009786	7:91900007|91902868	circRNA_003045	12:52150744|52168979	circRNA_013753	1:188117293|188179860
circRNA_004326	4:102814251|102816499	circRNA_004854	7:120244208|120247070	circRNA_014191	13:34824430|34842668	circRNA_018800	15:115173583|115236155
circRNA_000915	15:55433780|55436029	circRNA_014052	6:26460696|26463559	circRNA_004825	7:97232754|97250995	circRNA_002846	10:59564631|59627241
circRNA_004585	6:81644326|81646576	circRNA_006409	8:75358318|75361182	circRNA_012708	15:44246446|44264697	circRNA_017150	5:85618048|85680662
circRNA_005245	GL894351.1:20089|22339	circRNA_018190	15:152395889|152398753	circRNA_008357	1:116730739|116748998	circRNA_001464	3:97846059|97908682
circRNA_003813	18:9751490|9753741	circRNA_008354	1:109686040|109688905	circRNA_000213	1:240860923|240879186	circRNA_000523	12:62856482|62919117
circRNA_014945	7:100613625|100615876	circRNA_016926	16:20340270|20343136	circRNA_014311	16:36914607|36932871	circRNA_012616	14:10442544|10505232
circRNA_009755	7:60544561|60546813	circRNA_007121	14:44784269|44787136	circRNA_006511	9:138768104|138786372	circRNA_013133	4:133935658|133998356
circRNA_016075	4:67949008|67951260	circRNA_003229	13:148316595|148319463	circRNA_012521	13:35107180|35125451	circRNA_006772	1:269912309|269975028
circRNA_016291	7:53606391|53608643	circRNA_011289	13:206281824|206284694	circRNA_003236	13:158524462|158542736	circRNA_018668	13:142537295|142600017
circRNA_020804	17:9442844|9445097	circRNA_016408	8:140676371|140679241	circRNA_020654	13:191928098|191946373	circRNA_011150	11:76367637|76430435
circRNA_013839	14:71901126|71903380	circRNA_016523	GL893953.2:79067|81938	circRNA_011778	4:87306270|87324546	circRNA_019041	4:101173329|101236174
circRNA_015955	3:18191836|18194090	circRNA_000483	12:37781159|37784033	circRNA_015675	14:142244393|142262670	circRNA_001376	2:158058831|158121727
circRNA_005882	16:6441497|6443753	circRNA_003624	15:120872321|120875195	circRNA_012751	15:107383202|107401486	circRNA_013418	8:128735200|128798141
circRNA_019481	13:51772131|51774387	circRNA_008200	GL893953.2:79064|81938	circRNA_014428	4:73714673|73732960	circRNA_014345	18:36472459|36535413
circRNA_010554	2:3018042|3020299	circRNA_011933	7:33633384|33636258	circRNA_001926	6:148118708|148136997	circRNA_020080	16:47267573|47330528
circRNA_014105	1:86183677|86185934	circRNA_012263	1:143460231|143463105	circRNA_018016	GL892421.2:51224|69514	circRNA_003131	13:67147906|67210865
circRNA_020796	16:74259804|74262061	circRNA_000231	1:256943805|256946680	circRNA_004077	3:40257421|40275714	circRNA_001657	4:133935391|133998356
circRNA_010545	18:27446734|27448992	circRNA_003010	12:37668544|37671420	circRNA_011201	13:19755935|19774230	circRNA_014168	11:5189667|5252643
circRNA_019786	7:4875692|4877950	circRNA_011495	16:51827122|51829999	circRNA_006432	8:134712877|134731173	circRNA_011159	12:13030858|13093840
circRNA_006698	1:145435888|145438147	circRNA_015918	2:125069347|125072226	circRNA_013409	8:112042572|112060868	circRNA_000522	12:62856130|62919117
circRNA_016563	JH118583.1:29083|31342	circRNA_020767	15:152359161|152362040	circRNA_019599	17:49153503|49171800	circRNA_004806	7:74641881|74704888
circRNA_006065	3:38730108|38732368	circRNA_016620	1:16036042|16038922	circRNA_021235	8:140660940|140679241	circRNA_005998	2:82502736|82565744
circRNA_011160	12:13120690|13122950	circRNA_015207	1:127661208|127664089	circRNA_017531	10:56191251|56209553	circRNA_000349	10:58798085|58861115
circRNA_010075	GL895152.1:24396|26657	circRNA_015772	16:51845158|51848039	circRNA_004698	6:148034940|148053243	circRNA_015450	12:40543538|40606568
circRNA_008924	14:77058514|77060776	circRNA_004906	8:39412477|39415359	circRNA_020602	13:82870481|82888788	circRNA_017840	4:96740134|96803170
circRNA_009703	6:145355398|145357660	circRNA_008434	1:206990528|206993410	circRNA_003484	15:3788416|3806724	circRNA_020986	3:116938632|117001691
circRNA_003770	17:41092440|41094707	circRNA_007882	6:147864742|147867625	circRNA_010044	GL892486.1:4394|22703	circRNA_000812	14:78371743|78434807
circRNA_016236	6:91498124|91500391	circRNA_014167	11:3766438|3769323	circRNA_011509	17:15902244|15920554	circRNA_000607	13:84596887|84659957
circRNA_019105	6:75428214|75430481	circRNA_004187	3:118705451|118708337	circRNA_017279	8:76070362|76088673	circRNA_008571	10:77576677|77639775
circRNA_021001	4:15215395|15217662	circRNA_013932	5:106783200|106786086	circRNA_005008	8:130625685|130644003	circRNA_000154	1:157816758|157879888
circRNA_019509	13:202715644|202717912	circRNA_003106	13:37934642|37937529	circRNA_020065	15:110788555|110806875	circRNA_014100	1:45701230|45764360
circRNA_008065	8:141512465|141514736	circRNA_006682	1:134612798|134615687	circRNA_007039	13:118617384|118635708	circRNA_011743	4:19778172|19841332
circRNA_016994	18:49352398|49354669	circRNA_006893	11:53888439|53891329	circRNA_006588	X:140031246|140049571	circRNA_004374	4:133935391|133998585
circRNA_001591	4:73577490|73579762	circRNA_000455	12:16098936|16101827	circRNA_014131	1:202797721|202816050	circRNA_017734	18:14971565|15034759
circRNA_003391	14:78245851|78248123	circRNA_000711	13:210044909|210047801	circRNA_015962	3:40257421|40275754	circRNA_020593	13:55847657|55910893
circRNA_009153	17:44606585|44608858	circRNA_014436	4:129535947|129538840	circRNA_005531	1:304016474|304034811	circRNA_019843	9:23052452|23115725

circRNA_012866	18:14764051|14766324	circRNA_013592	X:121910171|121913065	circRNA_012641	14:51638062|51656404	circRNA_018893	2:3034318|3097595
circRNA_019237	8:141375566|141377839	circRNA_014538	8:140621348|140624242	circRNA_018823	16:25408511|25426853	circRNA_008045	8:109467554|109530854
circRNA_015645	14:82077154|82079428	circRNA_019733	5:63650386|63653282	circRNA_004684	6:146105224|146123569	circRNA_011371	14:122395679|122459005
circRNA_000657	13:132982287|132984562	circRNA_003698	16:38539919|38542819	circRNA_011535	18:9569911|9588258	circRNA_017442	1:101227154|101290518
circRNA_015969	3:49039216|49041491	circRNA_008673	12:44347013|44349914	circRNA_020692	14:71889478|71907827	circRNA_013070	4:12580211|12643641
circRNA_007211	15:7838580|7840856	circRNA_007875	6:145340412|145343315	circRNA_017601	13:97282134|97300487	circRNA_006152	4:85304081|85367540
circRNA_010479	15:85065639|85067916	circRNA_001248	2:52238948|52241853	circRNA_001342	2:128786909|128805266	circRNA_003057	12:62895314|62958836
circRNA_014684	2:95754776|95757053	circRNA_018970	3:40271918|40274823	circRNA_005448	1:134656768|134675125	circRNA_005704	13:103830057|103893618
circRNA_006943	12:44687527|44689805	circRNA_005101	9:40529883|40532789	circRNA_006649	1:93396709|93415072	circRNA_011203	13:28113282|28176864
circRNA_017313	9:13348736|13351015	circRNA_001010	15:122876918|122879826	circRNA_003009	12:37401810|37420176	circRNA_005393	X:138587662|138651247
circRNA_002597	1:142893340|142895620	circRNA_004123	3:84176934|84179842	circRNA_014095	1:23646297|23664663	circRNA_004199	3:120287019|120350634
circRNA_000579	13:42377857|42380138	circRNA_015118	13:28317262|28320170	circRNA_020073	16:18564948|18583314	circRNA_011913	6:147362290|147425956
circRNA_013629	14:108973811|108976092	circRNA_004369	4:129639341|129642250	circRNA_009388	3:102403868|102422235	circRNA_016988	18:26147189|26210863
circRNA_010306	11:14463282|14465565	circRNA_011005	1:141727584|141730493	circRNA_013059	3:128289492|128307860	circRNA_017463	1:154934355|154998031
circRNA_018511	1:256877152|256879435	circRNA_016647	1:128888081|128890990	circRNA_010622	3:72585580|72603949	circRNA_004674	6:139872924|139936605
circRNA_019081	5:80682993|80685276	circRNA_006471	9:40572085|40574995	circRNA_016158	5:70372892|70391261	circRNA_014150	10:21606482|21670197
circRNA_019748	6:17199325|17201608	circRNA_001091	17:14882939|14885851	circRNA_018616	13:1146627|1164999	circRNA_018422	1:80241844|80305582
circRNA_015521	13:82212210|82214494	circRNA_005967	2:15903735|15906647	circRNA_017153	5:92502063|92520442	circRNA_014702	4:117893543|117957301
circRNA_003932	2:80456480|80458765	circRNA_006688	1:137635283|137638195	circRNA_004203	3:121950526|121968907	circRNA_009158	17:47003535|47067299
circRNA_010002	9:108522658|108524946	circRNA_021250	9:22689056|22691968	circRNA_001654	4:129774517|129792899	circRNA_004471	5:76327567|76391339
circRNA_015673	14:140558898|140561186	circRNA_005389	X:127123810|127126725	circRNA_016521	GL893884.1:41214|59599	circRNA_001327	2:119165841|119229632
circRNA_002002	7:83110904|83113193	circRNA_020529	11:19622389|19625304	circRNA_004477	5:79767220|79785608	circRNA_008446	1:229131344|229195144
circRNA_017555	11:76652368|76654657	circRNA_006173	4:109817051|109819967	circRNA_008246	X:36800638|36819029	circRNA_014859	7:82092|145901
circRNA_018565	11:1725475|1727765	circRNA_014321	16:65103478|65106394	circRNA_011912	6:145384185|145402576	circRNA_017390	X:44568029|44631871
circRNA_019199	8:32179201|32181491	circRNA_011037	1:208292449|208295366	circRNA_001523	3:125854763|125873156	circRNA_016590	X:60851321|60915164
circRNA_002166	8:146890664|146892955	circRNA_012527	13:38469198|38472116	circRNA_012237	1:114031439|114049837	circRNA_000324	10:37409164|37473029
circRNA_018976	3:60071252|60073543	circRNA_011234	13:81528678|81531597	circRNA_001146	18:9569859|9588258	circRNA_005025	8:143175337|143239247
circRNA_015435	12:23687724|23690018	circRNA_002974	12:16861242|16864162	circRNA_003160	13:86663679|86682078	circRNA_008516	10:18123982|18187909
circRNA_017238	7:106583472|106585766	circRNA_009079	16:21326993|21329914	circRNA_000727	14:3642153|3660559	circRNA_013585	X:70790266|70854213
circRNA_004615	6:92177330|92179625	circRNA_003456	14:135311335|135314257	circRNA_012247	1:125143608|125162014	circRNA_006785	1:284448584|284512537
circRNA_019912	1:84042003|84044299	circRNA_009776	7:73296506|73299428	circRNA_015632	14:77056392|77074798	circRNA_018450	1:137504490|137568451
circRNA_018770	14:145719891|145722189	circRNA_016418	8:144739680|144742602	circRNA_010364	13:37968615|37987024	circRNA_018097	1:271527196|271591171
circRNA_013866	17:13112865|13115164	circRNA_010458	14:141219820|141222744	circRNA_011309	14:14230991|14249401	circRNA_010581	2:91605054|91669034
circRNA_002212	9:36992574|36994874	circRNA_011654	3:5155280|5158204	circRNA_014831	3:106268651|106287062	circRNA_005079	9:23052452|23116450
circRNA_002389	JH118940.1:100050|102350	circRNA_009607	6:2770529|2773454	circRNA_008365	1:127715924|127734337	circRNA_005165	9:127376687|127440700
circRNA_003435	14:120298194|120300496	circRNA_019533	14:63605183|63608108	circRNA_021284	9:115681075|115699492	circRNA_018011	9:140018765|140082783
circRNA_013298	7:103694|105997	circRNA_014796	14:144888359|144891286	circRNA_015276	1:230921715|230940137	circRNA_015821	18:14986350|15050446
circRNA_016319	7:102970997|102973300	circRNA_015767	16:47903918|47906845	circRNA_003306	14:16172107|16190532	circRNA_016700	1:284448584|284512682
circRNA_002751	1:284320474|284322778	circRNA_002363	GL895346.1:6767|9695	circRNA_005559	10:49667206|49685631	circRNA_005317	X:17284573|17348709
circRNA_021306	GL893374.2:3775|6080	circRNA_007850	6:99544759|99547687	circRNA_007045	13:126877865|126896290	circRNA_018564	11:486920|551063
circRNA_003145	13:80754587|80756893	circRNA_000527	13:3592151|3595080	circRNA_019726	5:42899527|42917954	circRNA_006185	4:136816726|136880888
circRNA_020414	1:142525118|142527425	circRNA_009693	6:125784145|125787074	circRNA_007062	13:143415401|143433833	circRNA_012418	11:5210653|5274850
circRNA_001297	2:87512163|87514471	circRNA_012646	14:52868461|52871390	circRNA_011553	18:44530678|44549111	circRNA_010787	7:24687028|24751226
circRNA_016209	6:46487333|46489641	circRNA_002011	7:91966709|91969641	circRNA_017475	1:181143247|181161684	circRNA_000316	10:35096153|35160373
circRNA_010941	X:86132408|86134717	circRNA_002691	1:227152127|227155059	circRNA_007869	6:132199188|132217626	circRNA_018881	18:42650214|42714461
circRNA_012044	9:11749319|11751629	circRNA_016150	5:53731099|53734031	circRNA_010876	9:46856389|46874828	circRNA_003971	2:91206107|91270409
circRNA_001324	2:119165841|119168152	circRNA_016943	16:47823935|47826868	circRNA_017943	7:109851808|109870247	circRNA_021154	7:24684178|24748519
circRNA_014341	18:19294616|19296928	circRNA_009495	4:121744658|121747592	circRNA_007978	8:1710939|1729389	circRNA_003635	15:124628985|124693334
circRNA_021207	8:75577793|75580105	circRNA_010231	1:205472022|205474960	circRNA_015795	17:36698962|36717412	circRNA_006023	2:105082209|105146567
circRNA_000983	15:106500764|106503077	circRNA_014185	12:60084897|60087835	circRNA_005686	13:75395663|75414115	circRNA_019542	14:105105415|105169801
circRNA_007321	16:47827438|47829752	circRNA_017154	5:98831114|98834052	circRNA_011403	15:61770154|61788609	circRNA_018502	1:232983793|233048185
circRNA_004781	7:63319985|63322300	circRNA_007491	2:93830099|93833038	circRNA_013861	16:27072071|27090533	circRNA_000962	15:86607064|86671532

circRNA_001600	4:87543028|87545344	circRNA_015692	15:55354683|55357622	circRNA_015500	13:37934642|37953108	circRNA_008839	13:160239598|160304071
circRNA_002497	1:50514833|50517149	circRNA_019053	4:129731405|129734347	circRNA_001757	5:86522733|86541201	circRNA_013268	6:109717487|109781967
circRNA_019767	6:101584451|101586767	circRNA_004836	7:102246054|102248998	circRNA_011044	1:225509264|225527736	circRNA_003564	15:85397016|85461499
circRNA_016795	13:67268107|67270424	circRNA_017956	8:57887917|57890863	circRNA_003843	18:27396026|27414502	circRNA_000659	13:138876327|138940815
circRNA_003294	14:9504665|9506983	circRNA_001130	17:61049424|61052372	circRNA_007237	15:61335011|61353487	circRNA_016290	7:53408460|53472958
circRNA_021329	GL895413.1:4477|6795	circRNA_003908	2:47973024|47975973	circRNA_004074	3:33359977|33378455	circRNA_008670	12:38622947|38687508
circRNA_000051	1:50514830|50517149	circRNA_010325	12:18787369|18790319	circRNA_012143	JH118724.1:73816|92294	circRNA_020491	10:2342778|2407376
circRNA_000760	14:31362268|31364588	circRNA_017216	7:7763903|7766853	circRNA_014086	7:105390102|105408581	circRNA_012867	18:18610834|18675443
circRNA_003498	15:19240976|19243296	circRNA_005851	15:93334714|93337665	circRNA_015198	1:110529857|110548336	circRNA_009896	8:134344412|134409025
circRNA_019628	2:80454230|80456550	circRNA_009291	2:121380856|121383808	circRNA_015390	11:26262333|26280812	circRNA_018865	18:14986350|15051001
circRNA_001612	4:98051660|98053981	circRNA_014250	14:58819704|58822656	circRNA_020614	13:115377550|115396031	circRNA_015185	1:87753751|87818404
circRNA_010850	8:120815672|120817996	circRNA_020829	18:10809119|10812072	circRNA_019521	14:17053787|17072269	circRNA_003821	18:15002914|15067619
circRNA_010251	1:282142302|282144628	circRNA_001768	5:99165946|99168902	circRNA_015950	3:10470262|10488747	circRNA_010957	1:21083206|21147916
circRNA_013675	13:38942704|38945031	circRNA_017730	18:5567767|5570723	circRNA_002901	11:21795724|21814210	circRNA_012407	11:486346|551063
circRNA_010649	4:16798423|16800751	circRNA_008063	8:141415791|141418749	circRNA_017139	5:35776549|35795037	circRNA_005738	13:167069887|167134609
circRNA_010571	2:70498613|70500945	circRNA_014803	16:23583623|23586581	circRNA_007529	3:10470262|10488752	circRNA_019252	9:25276912|25341666
circRNA_000138	1:143756831|143759164	circRNA_015113	1:72012306|72015264	circRNA_012559	13:84829078|84847569	circRNA_003190	13:103830057|103894828
circRNA_008650	12:21029531|21031864	circRNA_020954	3:49146892|49149851	circRNA_000540	13:23702427|23720920	circRNA_000879	14:152764272|152829057
circRNA_002646	1:195169049|195171383	circRNA_003177	13:97278057|97281017	circRNA_003245	13:167164236|167182729	circRNA_018638	13:67266325|67331110
circRNA_004622	6:99190172|99192507	circRNA_015944	2:148215117|148218077	circRNA_011305	14:10542537|10561030	circRNA_006489	9:85856790|85921589
circRNA_007815	6:48922011|48924347	circRNA_006983	13:33494610|33497574	circRNA_018769	14:141884764|141903257	circRNA_012193	1:30877430|30942245
circRNA_011599	2:72404917|72407253	circRNA_020052	14:141234287|141237251	circRNA_011031	1:200269189|200287684	circRNA_006954	12:62458832|62523689
circRNA_012945	2:84231850|84234187	circRNA_003049	12:54662564|54665529	circRNA_020643	13:158525065|158543560	circRNA_014355	2:41825697|41890569
circRNA_017382	JH118921.1:27248|29587	circRNA_003053	12:62443557|62446522	circRNA_015108	X:70638360|70656856	circRNA_015261	1:222016017|222080892
circRNA_004082	3:43107742|43110083	circRNA_004178	3:113974245|113977210	circRNA_015168	1:56918248|56936748	circRNA_014937	5:32968798|33033688
circRNA_009625	6:32619094|32621437	circRNA_005354	X:78878616|78881582	circRNA_012814	16:51689555|51708056	circRNA_003396	14:81297022|81361983
circRNA_001116	17:45690868|45693212	circRNA_017248	7:128850749|128853715	circRNA_011989	8:8825493|8843997	circRNA_004732	7:24656119|24721087
circRNA_014334	17:49184883|49187227	circRNA_011626	2:112705863|112708830	circRNA_002267	9:101227171|101245683	circRNA_003703	16:47050901|47115880
circRNA_004637	6:102944006|102946352	circRNA_011383	14:141234283|141237251	circRNA_000891	15:22746712|22765227	circRNA_015935	2:140683938|140748941
circRNA_010731	6:16298300|16300646	circRNA_009027	15:84437803|84440772	circRNA_001504	3:118042427|118060942	circRNA_008973	14:142775864|142840902
circRNA_008118	9:51475077|51477424	circRNA_017746	2:12139164|12142133	circRNA_002805	10:35244566|35263088	circRNA_004273	4:53450096|53515135
circRNA_008214	GL895204.1:2317|4664	circRNA_012001	8:57375305|57378275	circRNA_016514	GL893095.2:93598|112122	circRNA_020352	X:35351485|35416528
circRNA_010317	11:74618777|74621124	circRNA_009670	6:92433833|92436806	circRNA_000498	12:45493347|45511876	circRNA_000388	11:14212237|14277417
circRNA_011940	7:45327224|45329571	circRNA_007436	2:43970357|43973332	circRNA_020124	2:122627161|122645691	circRNA_007392	18:26314382|26379639
circRNA_007836	6:81578551|81580900	circRNA_019776	6:146111177|146114152	circRNA_018585	12:2107547|2126079	circRNA_012978	2:145758608|145823933
circRNA_001976	7:60540874|60543228	circRNA_003044	12:52150744|52153720	circRNA_014912	11:49690381|49708915	circRNA_020275	7:117841710|117907064
circRNA_000980	15:104539261|104541616	circRNA_002203	9:23112748|23115725	circRNA_018931	2:104884924|104903460	circRNA_007459	2:72132914|72198308
circRNA_006831	10:43538376|43540731	circRNA_002790	10:29336781|29339758	circRNA_021281	9:102762643|102781181	circRNA_017200	6:132108169|132173603
circRNA_012401	10:77706784|77709139	circRNA_008276	X:121823171|121826148	circRNA_006877	11:22539619|22558158	circRNA_011884	6:79809424|79874911
circRNA_015974	3:57699119|57701474	circRNA_004496	5:93402344|93405323	circRNA_009223	2:17348985|17367528	circRNA_019503	13:168397816|168463336
circRNA_015909	2:107639457|107641813	circRNA_018959	3:11060821|11063801	circRNA_019275	9:78881650|78900203	circRNA_020691	14:71312025|71377671
circRNA_006542	JH118478.1:124826|127183	circRNA_016008	3:102803374|102806357	circRNA_006040	2:134953156|134971715	circRNA_006605	1:16415160|16480830
circRNA_007029	13:91490811|91493168	circRNA_016850	14:60790049|60793033	circRNA_002082	8:41183979|41202540	circRNA_018871	18:24457721|24523395
circRNA_010031	9:146397908|146400265	circRNA_002647	1:195208521|195211514	circRNA_003796	17:57160790|57179361	circRNA_009469	4:72746838|72812537
circRNA_014165	10:71359304|71361661	circRNA_016607	X:116392501|116395496	circRNA_010244	1:242750212|242768784	circRNA_010490	15:111557295|111623007
circRNA_015975	3:58965760|58968119	circRNA_018116	12:16187562|16190557	circRNA_019734	5:64164266|64182839	circRNA_019004	3:117902722|117968462
circRNA_019846	9:40608131|40610490	circRNA_012292	1:208312465|208315461	circRNA_001238	2:41866547|41885122	circRNA_013913	4:72410011|72475797
circRNA_004509	5:106607717|106610077	circRNA_005184	9:133364153|133367150	circRNA_016906	15:94327571|94346146	circRNA_006837	10:50688302|50754092
circRNA_003457	14:135597237|135599599	circRNA_009513	5:477943|480940	circRNA_003929	2:79837584|79856164	circRNA_002480	1:24792111|24857916
circRNA_010212	1:161192141|161194504	circRNA_002004	7:86335987|86338985	circRNA_010444	14:82894668|82913253	circRNA_007630	4:31387169|31452986
circRNA_017931	7:57682410|57684773	circRNA_003343	14:42660884|42663883	circRNA_020506	10:37117371|37135961	circRNA_006712	1:161699776|161765610
circRNA_014728	8:18856400|18858765	circRNA_000767	14:34729613|34732614	circRNA_010831	8:46471841|46490433	circRNA_011981	7:129912574|129978456

<table>
<tr><td>circRNA_016932</td><td>16:25736184|25738549</td><td>circRNA_010618</td><td>3:48632532|48635534</td><td>circRNA_012882</td><td>18:44770456|44789048</td><td>circRNA_010241</td><td>1:241875289|241941205</td></tr>
<tr><td>circRNA_011050</td><td>1:245505757|245508123</td><td>circRNA_014248</td><td>14:52162032|52165034</td><td>circRNA_006770</td><td>1:267671789|267690386</td><td>circRNA_011816</td><td>5:50595002|50660921</td></tr>
<tr><td>circRNA_013297</td><td>6:156410973|156413339</td><td>circRNA_017389</td><td>X:41048022|41051024</td><td>circRNA_019069</td><td>5:49631609|49650207</td><td>circRNA_001607</td><td>4:90657864|90723810</td></tr>
<tr><td>circRNA_010361</td><td>13:34856835|34859202</td><td>circRNA_016496</td><td>9:115691139|115694143</td><td>circRNA_012509</td><td>13:15326169|15344773</td><td>circRNA_001429</td><td>3:57418121|57484136</td></tr>
<tr><td>circRNA_014565</td><td>9:112775690|112778058</td><td>circRNA_019700</td><td>4:71284022|71287026</td><td>circRNA_002561</td><td>1:120607344|120625949</td><td>circRNA_016065</td><td>4:48056407|48122458</td></tr>
<tr><td>circRNA_003015</td><td>12:39195360|39197730</td><td>circRNA_001416</td><td>3:49146846|49149851</td><td>circRNA_006390</td><td>8:30550155|30568764</td><td>circRNA_001558</td><td>4:35844936|35911000</td></tr>
<tr><td>circRNA_010775</td><td>6:156410969|156413339</td><td>circRNA_003303</td><td>14:14118550|14121555</td><td>circRNA_020015</td><td>13:144152728|144171342</td><td>circRNA_014960</td><td>1:60892775|60958863</td></tr>
<tr><td>circRNA_004970</td><td>8:89353525|89355896</td><td>circRNA_016625</td><td>1:24166856|24169861</td><td>circRNA_001318</td><td>2:107412680|107431295</td><td>circRNA_004908</td><td>8:39666147|39732243</td></tr>
<tr><td>circRNA_013769</td><td>1:49498577|49500948</td><td>circRNA_017955</td><td>8:55114181|55117186</td><td>circRNA_019393</td><td>1:204985026|205003641</td><td>circRNA_021052</td><td>5:34134710|34200867</td></tr>
<tr><td>circRNA_015760</td><td>16:40649530|40651901</td><td>circRNA_001286</td><td>2:85973785|85976791</td><td>circRNA_004179</td><td>3:114020620|114039242</td><td>circRNA_016411</td><td>8:142157274|142223491</td></tr>
<tr><td>circRNA_010555</td><td>2:4157505|4159877</td><td>circRNA_011697</td><td>3:76361902|76364908</td><td>circRNA_018385</td><td>GL893852.2:48917|67540</td><td>circRNA_017632</td><td>14:58521239|58587459</td></tr>
<tr><td>circRNA_011252</td><td>13:108917686|108920058</td><td>circRNA_012282</td><td>1:185283215|185286221</td><td>circRNA_019551</td><td>14:122681530|122700154</td><td>circRNA_002979</td><td>12:19072621|19138843</td></tr>
<tr><td>circRNA_003906</td><td>2:45065086|45067459</td><td>circRNA_017012</td><td>2:45059358|45062364</td><td>circRNA_009245</td><td>2:50787095|50805720</td><td>circRNA_016743</td><td>11:74310647|74376902</td></tr>
<tr><td>circRNA_007166</td><td>14:108184362|108186735</td><td>circRNA_016325</td><td>7:122497132|122500139</td><td>circRNA_019769</td><td>6:125557440|125576069</td><td>circRNA_019605</td><td>18:15002914|15069170</td></tr>
<tr><td>circRNA_000355</td><td>10:62803459|62805834</td><td>circRNA_004112</td><td>3:76361899|76364908</td><td>circRNA_000958</td><td>15:85397016|85415647</td><td>circRNA_010868</td><td>9:28646434|28712693</td></tr>
<tr><td>circRNA_014348</td><td>18:52492322|52494698</td><td>circRNA_010982</td><td>1:101847657|101850666</td><td>circRNA_007948</td><td>7:99001662|99020293</td><td>circRNA_016229</td><td>6:85411301|85477563</td></tr>
<tr><td>circRNA_017712</td><td>16:51317149|51319525</td><td>circRNA_004777</td><td>7:62827072|62830084</td><td>circRNA_012106</td><td>GL893095.2:17152|35786</td><td>circRNA_001480</td><td>3:103781514|103847787</td></tr>
<tr><td>circRNA_006999</td><td>13:60155936|60158313</td><td>circRNA_011939</td><td>7:43870109|43873122</td><td>circRNA_010626</td><td>3:84430711|84449348</td><td>circRNA_013370</td><td>8:29244364|29310723</td></tr>
<tr><td>circRNA_018780</td><td>15:61777761|61780138</td><td>circRNA_006083</td><td>3:81864480|81867496</td><td>circRNA_002325</td><td>GL892628.1:7171|25809</td><td>circRNA_006650</td><td>1:94529065|94595460</td></tr>
<tr><td>circRNA_020575</td><td>13:18138045|18140422</td><td>circRNA_011091</td><td>10:29270850|29273866</td><td>circRNA_015302</td><td>1:278920982|278939621</td><td>circRNA_005720</td><td>13:142562710|142629113</td></tr>
<tr><td>circRNA_003290</td><td>14:7103656|7106034</td><td>circRNA_002824</td><td>10:47148477|47151494</td><td>circRNA_000060</td><td>1:62359384|62378026</td><td>circRNA_014368</td><td>2:104437584|104503990</td></tr>
<tr><td>circRNA_016974</td><td>18:10883227|10885605</td><td>circRNA_003489</td><td>15:8286403|8289420</td><td>circRNA_000118</td><td>1:134518071|134536714</td><td>circRNA_016884</td><td>14:145096063|145162509</td></tr>
<tr><td>circRNA_008149</td><td>9:101875176|101877556</td><td>circRNA_008668</td><td>12:37398861|37401878</td><td>circRNA_000117</td><td>1:134518067|134536714</td><td>circRNA_012202</td><td>1:32143461|32209973</td></tr>
<tr><td>circRNA_017651</td><td>14:124000124|124002504</td><td>circRNA_016474</td><td>9:72069020|72072037</td><td>circRNA_020792</td><td>16:51757091|51775742</td><td>circRNA_015362</td><td>10:60057311|60123846</td></tr>
<tr><td>circRNA_020821</td><td>17:66416059|66418439</td><td>circRNA_000263</td><td>1:299796549|299799568</td><td>circRNA_011094</td><td>10:29342271|29360924</td><td>circRNA_009499</td><td>4:129426261|129492839</td></tr>
<tr><td>circRNA_011902</td><td>6:119548772|119551153</td><td>circRNA_008473</td><td>1:257015250|257018270</td><td>circRNA_005949</td><td>18:20373134|20391792</td><td>circRNA_018499</td><td>1:229296125|229362710</td></tr>
<tr><td>circRNA_007580</td><td>3:98698220|98700602</td><td>circRNA_004432</td><td>5:38471187|38474208</td><td>circRNA_020141</td><td>3:62785202|62803860</td><td>circRNA_001412</td><td>3:42764109|42830696</td></tr>
<tr><td>circRNA_009462</td><td>4:56554006|56556388</td><td>circRNA_012402</td><td>11:113524|116545</td><td>circRNA_017348</td><td>9:115742803|115761463</td><td>circRNA_004364</td><td>4:129426261|129492850</td></tr>
<tr><td>circRNA_015041</td><td>6:125581476|125583858</td><td>circRNA_000524</td><td>13:1121459|1124482</td><td>circRNA_013183</td><td>5:76372676|76391339</td><td>circRNA_010036</td><td>GL892397.1:56539|123194</td></tr>
<tr><td>circRNA_019731</td><td>5:57064654|57067037</td><td>circRNA_006044</td><td>2:140676197|140679220</td><td>circRNA_014464</td><td>6:16284471|16303134</td><td>circRNA_000006</td><td>1:11849149|11915854</td></tr>
<tr><td>circRNA_003382</td><td>14:77904652|77907036</td><td>circRNA_012614</td><td>14:6690452|6693475</td><td>circRNA_006523</td><td>GL893689.1:13178|31842</td><td>circRNA_012262</td><td>1:143396359|143463105</td></tr>
<tr><td>circRNA_005905</td><td>16:68472703|68475090</td><td>circRNA_009463</td><td>4:68324097|68327121</td><td>circRNA_006158</td><td>4:90623622|90642287</td><td>circRNA_014699</td><td>4:31396526|31463369</td></tr>
<tr><td>circRNA_019435</td><td>10:66171809|66174196</td><td>circRNA_005622</td><td>12:25819429|25822455</td><td>circRNA_015997</td><td>3:77312222|77330887</td><td>circRNA_002779</td><td>10:18101700|18168555</td></tr>
<tr><td>circRNA_003346</td><td>14:44341630|44344018</td><td>circRNA_013415</td><td>8:120770163|120773190</td><td>circRNA_020524</td><td>11:11297213|11315888</td><td>circRNA_009731</td><td>7:21551602|21618500</td></tr>
<tr><td>circRNA_009237</td><td>2:43943609|43945997</td><td>circRNA_005865</td><td>15:115740395|115743423</td><td>circRNA_013739</td><td>9:116123437|116142114</td><td>circRNA_020847</td><td>18:52318955|52385888</td></tr>
<tr><td>circRNA_007065</td><td>13:144686339|144688729</td><td>circRNA_007933</td><td>7:86330961|86333989</td><td>circRNA_009435</td><td>4:19647782|19666461</td><td>circRNA_007858</td><td>6:113695691|113762635</td></tr>
<tr><td>circRNA_008936</td><td>14:82958111|82960502</td><td>circRNA_014882</td><td>8:141404602|141407630</td><td>circRNA_014836</td><td>4:80758319|80776999</td><td>circRNA_008058</td><td>8:134355181|134422131</td></tr>
<tr><td>circRNA_004820</td><td>7:91964457|91966849</td><td>circRNA_005527</td><td>1:297536723|297539752</td><td>circRNA_007877</td><td>6:146044580|146063275</td><td>circRNA_009599</td><td>5:105210066|105277027</td></tr>
<tr><td>circRNA_005932</td><td>17:55857987|55860379</td><td>circRNA_003585</td><td>15:97747096|97750126</td><td>circRNA_000612</td><td>13:86966559|86985258</td><td>circRNA_008075</td><td>9:8196997|8263976</td></tr>
<tr><td>circRNA_014926</td><td>18:44782112|44784505</td><td>circRNA_020550</td><td>12:23729226|23732256</td><td>circRNA_015028</td><td>2:145725178|145743881</td><td>circRNA_008993</td><td>15:36377634|36444626</td></tr>
<tr><td>circRNA_002106</td><td>8:82954008|82956402</td><td>circRNA_017665</td><td>15:65951210|65954241</td><td>circRNA_000318</td><td>10:37017761|37036465</td><td>circRNA_008560</td><td>10:62722156|62789150</td></tr>
<tr><td>circRNA_012839</td><td>17:32967593|32969987</td><td>circRNA_021289</td><td>9:129651936|129654968</td><td>circRNA_006186</td><td>4:141562814|141581521</td><td>circRNA_012755</td><td>15:112090894|112157900</td></tr>
<tr><td>circRNA_017805</td><td>3:114071758|114074153</td><td>circRNA_013613</td><td>4:107618726|107621759</td><td>circRNA_015328</td><td>10:18149844|18168555</td><td>circRNA_012800</td><td>16:35350655|35417661</td></tr>
<tr><td>circRNA_009748</td><td>7:50155587|50157983</td><td>circRNA_009110</td><td>16:51834324|51837358</td><td>circRNA_016439</td><td>9:25341606|25360320</td><td>circRNA_005054</td><td>9:11252773|11319790</td></tr>
<tr><td>circRNA_015294</td><td>1:264888802|264891198</td><td>circRNA_004069</td><td>3:24318465|24321501</td><td>circRNA_010525</td><td>17:43061013|43079731</td><td>circRNA_013256</td><td>6:88281456|88348522</td></tr>
<tr><td>circRNA_017418</td><td>1:13626498|13628894</td><td>circRNA_007476</td><td>2:83908876|83911912</td><td>circRNA_003150</td><td>13:82880925|82899646</td><td>circRNA_005960</td><td>18:59484562|59551671</td></tr>
<tr><td>circRNA_014877</td><td>8:57805777|57808175</td><td>circRNA_019304</td><td>GL892841.1:101878|104914</td><td>circRNA_018345</td><td>8:32353099|32371824</td><td>circRNA_018549</td><td>10:46711922|46779066</td></tr>
<tr><td>circRNA_019749</td><td>6:18394863|18397261</td><td>circRNA_020513</td><td>10:54054021|54057057</td><td>circRNA_009951</td><td>9:42955177|42973905</td><td>circRNA_000335</td><td>10:46711919|46779066</td></tr>
<tr><td>circRNA_015897</td><td>2:87542278|87544679</td><td>circRNA_018129</td><td>13:97883427|97886464</td><td>circRNA_013040</td><td>3:102653727|102672455</td><td>circRNA_011100</td><td>10:35240929|35308084</td></tr>
<tr><td>circRNA_011116</td><td>10:54381760|54384166</td><td>circRNA_018786</td><td>15:73940399|73943437</td><td>circRNA_002384</td><td>JH118611.1:2203|20940</td><td>circRNA_006237</td><td>5:90554905|90622092</td></tr>
<tr><td>circRNA_005149</td><td>9:102582514|102584921</td><td>circRNA_008686</td><td>12:52750817|52753856</td><td>circRNA_016146</td><td>5:49623445|49642185</td><td>circRNA_004017</td><td>2:135507455|135574724</td></tr>
<tr><td>circRNA_008855</td><td>13:206374530|206376938</td><td>circRNA_011391</td><td>15:20147715|20150754</td><td>circRNA_021258</td><td>9:40761242|40779984</td><td>circRNA_013885</td><td>2:91601752|91669034</td></tr>
</table>

<table>
<tr><td>circRNA_009403</td><td>3:114030015|114032423</td><td>circRNA_019187</td><td>7:102244418|102247457</td><td>circRNA_015610</td><td>14:51502334|51521077</td><td>circRNA_016383</td><td>8:85974968|86042328</td></tr>
<tr><td>circRNA_010338</td><td>12:37378287|37380695</td><td>circRNA_019414</td><td>1:283056939|283059979</td><td>circRNA_002544</td><td>1:101191388|101210132</td><td>circRNA_011456</td><td>15:150299956|150367390</td></tr>
<tr><td>circRNA_020086</td><td>17:6175245|6177653</td><td>circRNA_008055</td><td>8:127051429|127054470</td><td>circRNA_007659</td><td>4:67874342|67893087</td><td>circRNA_001383</td><td>3:9267608|9335045</td></tr>
<tr><td>circRNA_020983</td><td>3:113971998|113974406</td><td>circRNA_009931</td><td>9:29012165|29015206</td><td>circRNA_001848</td><td>6:92078907|92097655</td><td>circRNA_018520</td><td>1:282128502|282195982</td></tr>
<tr><td>circRNA_000105</td><td>1:126947681|126950090</td><td>circRNA_003682</td><td>16:29443058|29446101</td><td>circRNA_008518</td><td>10:30367820|30386570</td><td>circRNA_008479</td><td>1:270170108|270237597</td></tr>
<tr><td>circRNA_005233</td><td>GL893592.2:34539|36948</td><td>circRNA_008766</td><td>13:73046428|73049473</td><td>circRNA_008537</td><td>10:47086473|47105223</td><td>circRNA_020608</td><td>13:90885295|90952792</td></tr>
<tr><td>circRNA_016477</td><td>9:78746589|78749000</td><td>circRNA_018592</td><td>12:25019975|25023025</td><td>circRNA_020556</td><td>12:35858810|35877564</td><td>circRNA_002713</td><td>1:249850891|249918475</td></tr>
<tr><td>circRNA_020884</td><td>2:79596775|79599186</td><td>circRNA_004068</td><td>3:23526296|23529350</td><td>circRNA_014490</td><td>7:54178247|54197003</td><td>circRNA_004923</td><td>8:50589064|50656654</td></tr>
<tr><td>circRNA_012415</td><td>11:4045654|4048067</td><td>circRNA_007315</td><td>16:37504640|37507694</td><td>circRNA_009496</td><td>4:121911465|121930222</td><td>circRNA_001331</td><td>2:121253565|121321204</td></tr>
<tr><td>circRNA_017628</td><td>14:49377982|49380395</td><td>circRNA_014256</td><td>14:71904773|71907827</td><td>circRNA_007651</td><td>4:53450096|53468858</td><td>circRNA_016660</td><td>1:154082449|154150097</td></tr>
<tr><td>circRNA_018639</td><td>13:67535162|67537576</td><td>circRNA_002231</td><td>9:50831548|50834603</td><td>circRNA_008279</td><td>X:127123810|127142583</td><td>circRNA_002256</td><td>9:82058623|82126273</td></tr>
<tr><td>circRNA_020089</td><td>17:21445615|21448029</td><td>circRNA_020342</td><td>GL894597.1:41592|44647</td><td>circRNA_004251</td><td>4:37565201|37583979</td><td>circRNA_001198</td><td>18:59628324|59696045</td></tr>
<tr><td>circRNA_020934</td><td>3:19854335|19856749</td><td>circRNA_013538</td><td>GL895152.1:20035|23092</td><td>circRNA_018590</td><td>12:16845381|16864162</td><td>circRNA_002479</td><td>1:24724428|24792185</td></tr>
<tr><td>circRNA_005247</td><td>GL894597.1:26840|29257</td><td>circRNA_014672</td><td>16:47942252|47945309</td><td>circRNA_018685</td><td>13:190976278|190995066</td><td>circRNA_001430</td><td>3:57418121|57485994</td></tr>
<tr><td>circRNA_003537</td><td>15:66308639|66311057</td><td>circRNA_013428</td><td>9:34787|37845</td><td>circRNA_014004</td><td>14:21462664|21481453</td><td>circRNA_006773</td><td>1:270169722|270237597</td></tr>
<tr><td>circRNA_015956</td><td>3:19609348|19611768</td><td>circRNA_006477</td><td>9:51076438|51079497</td><td>circRNA_000709</td><td>13:207175610|207194401</td><td>circRNA_017493</td><td>1:232091999|232159897</td></tr>
<tr><td>circRNA_001604</td><td>4:90623622|90626043</td><td>circRNA_010316</td><td>11:56114607|56117666</td><td>circRNA_000076</td><td>1:97905979|97924771</td><td>circRNA_018433</td><td>1:106896702|106964621</td></tr>
<tr><td>circRNA_004304</td><td>4:87330247|87332668</td><td>circRNA_007208</td><td>14:145861468|145864533</td><td>circRNA_008348</td><td>1:103346987|103365781</td><td>circRNA_000696</td><td>13:170324267|170392219</td></tr>
<tr><td>circRNA_013774</td><td>1:176853033|176855454</td><td>circRNA_006149</td><td>4:70407823|70410889</td><td>circRNA_001048</td><td>16:33999307|34018107</td><td>circRNA_020437</td><td>1:183342472|183410425</td></tr>
<tr><td>circRNA_002674</td><td>1:205265275|205267698</td><td>circRNA_006591</td><td>1:985624|988691</td><td>circRNA_018493</td><td>1:216008681|216027485</td><td>circRNA_017992</td><td>9:57662614|57730623</td></tr>
<tr><td>circRNA_008765</td><td>13:67634648|67637071</td><td>circRNA_013236</td><td>6:41229622|41232689</td><td>circRNA_016424</td><td>9:7103733|7122545</td><td>circRNA_013892</td><td>2:139496126|139564154</td></tr>
<tr><td>circRNA_016557</td><td>GL896553.1:668|3094</td><td>circRNA_005652</td><td>13:25498737|25501805</td><td>circRNA_004152</td><td>3:102211044|102229861</td><td>circRNA_001253</td><td>2:59187624|59255661</td></tr>
<tr><td>circRNA_018791</td><td>15:85375263|85377689</td><td>circRNA_019510</td><td>13:207522489|207525558</td><td>circRNA_007081</td><td>13:202372077|202390898</td><td>circRNA_009026</td><td>15:83526823|83594895</td></tr>
<tr><td>circRNA_015967</td><td>3:46253973|46256400</td><td>circRNA_003921</td><td>2:61950218|61953288</td><td>circRNA_004866</td><td>7:134061631|134080464</td><td>circRNA_015518</td><td>13:77798662|77866748</td></tr>
<tr><td>circRNA_015300</td><td>1:276527636|276530064</td><td>circRNA_019833</td><td>8:140260091|140263164</td><td>circRNA_007441</td><td>2:50789562|50808396</td><td>circRNA_008771</td><td>13:75540795|75608947</td></tr>
<tr><td>circRNA_007162</td><td>14:97974694|97977123</td><td>circRNA_014438</td><td>4:142642590|142645664</td><td>circRNA_009236</td><td>2:43761227|43780067</td><td>circRNA_002173</td><td>9:3416060|3484227</td></tr>
<tr><td>circRNA_009131</td><td>17:13118846|13121277</td><td>circRNA_001131</td><td>17:61049424|61052499</td><td>circRNA_007057</td><td>13:136691991|136710833</td><td>circRNA_013130</td><td>4:122811569|122879761</td></tr>
<tr><td>circRNA_013314</td><td>7:43147749|43150180</td><td>circRNA_010687</td><td>4:142642590|142645666</td><td>circRNA_007652</td><td>4:55214192|55233036</td><td>circRNA_010284</td><td>10:46613551|46681760</td></tr>
<tr><td>circRNA_016024</td><td>3:114476157|114478588</td><td>circRNA_012994</td><td>3:17185508|17188584</td><td>circRNA_000387</td><td>11:13305769|13324616</td><td>circRNA_020264</td><td>7:69814148|69882364</td></tr>
<tr><td>circRNA_001968</td><td>7:52958469|52960901</td><td>circRNA_011209</td><td>13:33736722|33739802</td><td>circRNA_011326</td><td>14:51693016|51711869</td><td>circRNA_019966</td><td>10:53393187|53461410</td></tr>
<tr><td>circRNA_020173</td><td>4:55222894|55225326</td><td>circRNA_014568</td><td>9:117749747|117752827</td><td>circRNA_000942</td><td>15:73900098|73918954</td><td>circRNA_012161</td><td>X:78770431|78838672</td></tr>
<tr><td>circRNA_010882</td><td>9:67375854|67378287</td><td>circRNA_004812</td><td>7:86745779|86748861</td><td>circRNA_004572</td><td>6:74096530|74115386</td><td>circRNA_009558</td><td>5:70006732|70074979</td></tr>
<tr><td>circRNA_018248</td><td>3:14261710|14264143</td><td>circRNA_005553</td><td>10:37797097|37800179</td><td>circRNA_000853</td><td>14:120768685|120787546</td><td>circRNA_017293</td><td>8:134340773|134409025</td></tr>
<tr><td>circRNA_019878</td><td>GL896423.1:20702|23135</td><td>circRNA_003274</td><td>13:211128404|211131487</td><td>circRNA_013019</td><td>3:63038185|63057047</td><td>circRNA_020585</td><td>13:41555863|41624117</td></tr>
<tr><td>circRNA_008794</td><td>13:97312436|97314870</td><td>circRNA_019589</td><td>16:51710534|51713618</td><td>circRNA_018681</td><td>13:167798432|167817294</td><td>circRNA_007040</td><td>13:119145908|119214179</td></tr>
<tr><td>circRNA_000577</td><td>13:42362493|42364929</td><td>circRNA_021188</td><td>8:15379042|15382126</td><td>circRNA_003328</td><td>14:32119110|32137975</td><td>circRNA_000003</td><td>1:6417991|6486326</td></tr>
<tr><td>circRNA_011008</td><td>1:142633280|142635716</td><td>circRNA_006601</td><td>1:9911040|9914125</td><td>circRNA_003356</td><td>14:53590430|53609301</td><td>circRNA_001797</td><td>6:45070836|45139175</td></tr>
<tr><td>circRNA_011250</td><td>13:108395217|108397654</td><td>circRNA_008852</td><td>13:205655759|205658845</td><td>circRNA_007600</td><td>3:116947785|116966656</td><td>circRNA_009447</td><td>4:40712731|40781089</td></tr>
<tr><td>circRNA_003642</td><td>15:133380261|133382701</td><td>circRNA_000912</td><td>15:55069306|55072396</td><td>circRNA_017429</td><td>1:50085656|50104536</td><td>circRNA_011245</td><td>13:98113084|98181473</td></tr>
<tr><td>circRNA_007235</td><td>15:59538969|59541410</td><td>circRNA_004083</td><td>3:43187440|43190530</td><td>circRNA_003782</td><td>17:45672152|45691034</td><td>circRNA_000995</td><td>15:116138978|116207401</td></tr>
<tr><td>circRNA_010768</td><td>6:137198133|137200574</td><td>circRNA_009957</td><td>9:44922267|44925357</td><td>circRNA_000930</td><td>15:71690220|71709108</td><td>circRNA_017340</td><td>9:83040537|83108986</td></tr>
<tr><td>circRNA_012387</td><td>10:56160513|56162954</td><td>circRNA_016259</td><td>6:145359462|145362552</td><td>circRNA_011073</td><td>1:303998762|304017650</td><td>circRNA_001875</td><td>6:101745126|101813590</td></tr>
<tr><td>circRNA_002847</td><td>10:59607342|59609785</td><td>circRNA_007531</td><td>3:10587049|10590140</td><td>circRNA_000947</td><td>15:80705053|80723942</td><td>circRNA_012979</td><td>2:145758608|145827131</td></tr>
<tr><td>circRNA_004003</td><td>2:122626814|122629257</td><td>circRNA_004143</td><td>3:92450105|92453197</td><td>circRNA_006117</td><td>3:126274387|126293277</td><td>circRNA_018254</td><td>3:57418121|57486702</td></tr>
<tr><td>circRNA_017556</td><td>11:86751850|86754293</td><td>circRNA_003452</td><td>14:131878915|131882008</td><td>circRNA_010246</td><td>1:251424046|251442945</td><td>circRNA_002959</td><td>12:14259841|14328470</td></tr>
<tr><td>circRNA_006273</td><td>6:81320444|81322888</td><td>circRNA_015161</td><td>1:41032896|41035989</td><td>circRNA_003740</td><td>17:5159655|5178557</td><td>circRNA_004242</td><td>4:32646874|32715543</td></tr>
<tr><td>circRNA_009821</td><td>8:4467677|4470121</td><td>circRNA_017510</td><td>10:11891726|11894819</td><td>circRNA_003442</td><td>14:122238908|122257813</td><td>circRNA_007446</td><td>2:52847764|52916460</td></tr>
<tr><td>circRNA_000177</td><td>1:202609214|202611659</td><td>circRNA_006382</td><td>8:4394321|4397416</td><td>circRNA_003353</td><td>14:52830738|52849650</td><td>circRNA_010015</td><td>9:129664193|129732908</td></tr>
<tr><td>circRNA_001940</td><td>6:157681701|157684147</td><td>circRNA_010945</td><td>X:113644886|113647981</td><td>circRNA_012528</td><td>13:42301408|42320321</td><td>circRNA_018792</td><td>15:86637917|86706652</td></tr>
<tr><td>circRNA_003350</td><td>14:50021607|50024055</td><td>circRNA_014316</td><td>16:51707557|51710653</td><td>circRNA_000122</td><td>1:134640969|134659892</td><td>circRNA_000007</td><td>1:11849149|11917891</td></tr>
<tr><td>circRNA_009850</td><td>8:57803396|57805849</td><td>circRNA_018587</td><td>12:7872827|7875923</td><td>circRNA_014656</td><td>14:60727790|60746720</td><td>circRNA_004307</td><td>4:88607905|88676666</td></tr>
<tr><td>circRNA_016119</td><td>4:142643211|142645664</td><td>circRNA_009537</td><td>5:38575947|38579044</td><td>circRNA_001880</td><td>6:101794657|101813590</td><td>circRNA_000072</td><td>1:86117059|86185934</td></tr>
</table>

circRNA_005415	1:28662025|28664480	circRNA_005889	16:25405911|25409009	circRNA_001743	5:73490736|73509675	circRNA_007202	14:142735102|142804005
circRNA_007707	4:142643211|142645666	circRNA_009573	5:77630305|77633404	circRNA_009238	2:43970357|43989297	circRNA_020134	3:42764109|42833022
circRNA_019246	9:13613834|13616289	circRNA_019751	6:18502173|18505277	circRNA_010847	8:103801474|103820414	circRNA_010840	8:81615914|81684836
circRNA_008941	14:98670665|98673121	circRNA_017359	GL893190.1:7005|10110	circRNA_009241	2:45026964|45045907	circRNA_006910	12:13030858|13099785
circRNA_005693	13:82688894|82691352	circRNA_007865	6:126803667|126806775	circRNA_012691	14:124814867|124833813	circRNA_016021	3:113980293|114049273
circRNA_005861	15:112461241|112463699	circRNA_001242	2:45975766|45978876	circRNA_004790	7:65666367|65685321	circRNA_011021	1:161699776|161768784
circRNA_014286	15:80705053|80707512	circRNA_007830	6:73234581|73237691	circRNA_010594	2:138829293|138848248	circRNA_019889	X:73939459|74008493
circRNA_013275	6:132199188|132201648	circRNA_012209	1:44466346|44469456	circRNA_002811	10:37127268|37146225	circRNA_003062	13:3656432|3725467
circRNA_005041	9:386764|389227	circRNA_013063	3:135328996|135332107	circRNA_021283	9:112750007|112768966	circRNA_006286	6:94766483|94835552
circRNA_009387	3:102170857|102173320	circRNA_003799	17:66503566|66506678	circRNA_001865	6:99240509|99259476	circRNA_015248	1:206804869|206873967
circRNA_012907	2:28405915|28408378	circRNA_013517	GL893195.1:14364|17476	circRNA_016173	5:87303583|87322552	circRNA_018922	2:83801388|83870489
circRNA_006059	3:11449859|11452323	circRNA_016274	7:17582645|17585757	circRNA_005382	X:121922443|121941420	circRNA_005429	1:93126380|93195489
circRNA_016918	15:122984115|122986579	circRNA_000265	1:300139455|300142568	circRNA_019495	13:140973312|140992290	circRNA_003556	15:85315348|85384479
circRNA_000961	15:86607064|86609530	circRNA_001132	17:66503563|66506678	circRNA_020754	15:98191119|98210102	circRNA_011926	7:12202504|12271827
circRNA_018461	1:163301980|163304446	circRNA_001705	5:38348311|38351426	circRNA_003495	15:18646758|18665742	circRNA_006137	4:39819040|39888370
circRNA_004041	2:148224335|148226802	circRNA_012239	1:117505463|117508579	circRNA_002279	9:108773188|108792173	circRNA_011838	5:87252274|87321630
circRNA_011731	3:118558304|118560771	circRNA_018858	17:57891339|57894455	circRNA_003831	18:18671313|18690307	circRNA_005210	9:150354490|150423880
circRNA_018532	10:17411955|17414423	circRNA_019800	7:83107205|83110322	circRNA_019132	6:138603449|138622445	circRNA_000716	13:210806110|210875564
circRNA_011003	1:136466617|136469087	circRNA_007848	6:99240509|99243627	circRNA_011566	2:15667215|15686214	circRNA_004051	3:11271739|11341198
circRNA_010495	15:116470717|116473189	circRNA_014580	GL893509.1:53470|56588	circRNA_010700	5:38561440|38580442	circRNA_008281	X:130647394|130716858
circRNA_012404	11:447879|450351	circRNA_015288	1:254933957|254937077	circRNA_015983	3:72511167|72530169	circRNA_011916	6:148401539|148471024
circRNA_018354	8:112720112|112722584	circRNA_009848	8:57779695|57782816	circRNA_005290	JH118724.1:73289|92294	circRNA_002152	8:134408464|134477954
circRNA_004299	4:74315685|74318160	circRNA_011437	15:117371369|117374490	circRNA_004044	2:150641324|150660330	circRNA_010977	1:86885065|86954593
circRNA_019340	X:51863815|51866290	circRNA_006496	9:115606023|115609146	circRNA_009135	17:15485334|15504340	circRNA_010849	8:117488167|117557776
circRNA_001422	3:51823390|51825866	circRNA_015696	15:66037290|66040413	circRNA_013403	8:103641047|103660054	circRNA_017615	13:187715366|187785003
circRNA_010340	12:40530928|40533404	circRNA_013548	GL896381.1:11360|14485	circRNA_001217	2:7344070|7363078	circRNA_006938	12:38732180|38801846
circRNA_013251	6:85038301|85040777	circRNA_005469	1:180819278|180822404	circRNA_001608	4:90704802|90723810	circRNA_013854	15:131248910|131318599
circRNA_004628	6:99256999|99259476	circRNA_007718	5:8899087|8902213	circRNA_006223	5:81374500|81393509	circRNA_006064	3:29760501|29830248
circRNA_013062	3:133992658|133995136	circRNA_005260	GL894963.1:4897|8025	circRNA_012493	12:44004809|44023821	circRNA_016297	7:65162015|65231776
circRNA_015301	1:278520891|278523369	circRNA_001060	16:37511575|37514704	circRNA_012666	14:78488895|78507923	circRNA_006272	6:74992305|75062084
circRNA_006053	3:4507183|4509663	circRNA_004661	6:132029330|132032459	circRNA_007832	6:74096530|74115559	circRNA_021295	9:138815139|138885004
circRNA_012776	15:133037228|133039708	circRNA_007861	6:125615895|125619025	circRNA_009189	18:27278420|27297449	circRNA_011452	15:145717007|145786873
circRNA_005331	X:46125649|46128130	circRNA_018343	8:30866481|30869611	circRNA_010664	4:45576146|45595180	circRNA_018954	2:147137211|147207083
circRNA_019967	10:76668485|76670966	circRNA_010359	13:33157395|33160528	circRNA_013859	15:155078849|155097886	circRNA_014571	9:138815124|138885004
circRNA_010737	6:40867633|40870115	circRNA_003018	12:40342412|40345546	circRNA_018796	15:101683537|101702576	circRNA_015368	11:5210653|5280553
circRNA_012341	10:16565804|16568287	circRNA_002277	9:102587388|102590523	circRNA_005618	12:20308389|20327437	circRNA_018056	X:85891093|85961011
circRNA_000970	15:93776942|93779427	circRNA_011041	1:224111540|224114675	circRNA_004010	2:128801546|128820597	circRNA_001045	16:29550281|29620216
circRNA_001729	5:65277754|65280239	circRNA_018218	2:6984578|6987713	circRNA_018025	GL894261.2:71993|91044	circRNA_004764	7:53408460|53478403
circRNA_002786	10:24473176|24475663	circRNA_011990	8:12575856|12578994	circRNA_017186	6:99084196|99103249	circRNA_018570	11:11234151|11304180
circRNA_004562	6:69244120|69246608	circRNA_003322	14:31282576|31285715	circRNA_003074	13:19793909|19812967	circRNA_001516	3:122008499|122078774
circRNA_005340	X:64982112|64984600	circRNA_019567	15:85074728|85077868	circRNA_002276	9:102535923|102554986	circRNA_014233	14:21462664|21532972
circRNA_006422	8:91914715|91917203	circRNA_012617	14:13295667|13298808	circRNA_019750	6:18421477|18440543	circRNA_004193	3:120081729|120152093
circRNA_010106	X:6528823|6531311	circRNA_009247	2:52846487|52849629	circRNA_021144	6:157477813|157496882	circRNA_004237	4:16999382|17069753
circRNA_019268	9:52667526|52670014	circRNA_012962	2:112531676|112534818	circRNA_007994	8:32694464|32713541	circRNA_021151	7:18129930|18200347
circRNA_005890	16:29026347|29028836	circRNA_002754	1:288073705|288076848	circRNA_005803	14:115739339|115758424	circRNA_015444	12:35849683|35920127
circRNA_007129	14:53336077|53338566	circRNA_014735	8:89363712|89366855	circRNA_013995	11:14837406|14856494	circRNA_003828	18:18645265|18715721
circRNA_009071	15:155076578|155079067	circRNA_008959	14:131871264|131874408	circRNA_003580	15:91091785|91110875	circRNA_020088	17:19717427|19787907
circRNA_016018	3:109917595|109920084	circRNA_006048	2:142575657|142578802	circRNA_003361	14:59874821|59893912	circRNA_003679	16:27840671|27911180
circRNA_007987	8:19247066|19249558	circRNA_011888	6:83025822|83028967	circRNA_004231	4:15253352|15272443	circRNA_007397	18:27447277|27517802
circRNA_009650	6:77621283|77623775	circRNA_014577	GL892789.1:63318|66463	circRNA_003379	14:77581776|77600868	circRNA_006620	1:28460560|28531105
circRNA_013161	5:32884538|32887030	circRNA_006271	6:74096530|74099676	circRNA_020642	13:158524462|158543560	circRNA_020837	18:30363095|30433649
circRNA_006846	10:56116139|56118632	circRNA_008867	14:933854|937000	circRNA_010956	1:16870879|16889979	circRNA_006291	6:101724150|101794738

circRNA_018785	15:73545877|73548370	circRNA_017533	10:61847984|61851131	circRNA_002181	9:11209215|11228318	circRNA_006296	6:122037337|122107954
circRNA_017597	13:77761973|77764468	circRNA_007571	3:90414201|90417350	circRNA_001737	5:70576776|70595881	circRNA_008381	1:143403895|143474541
circRNA_006082	3:81216721|81219219	circRNA_013454	9:43193719|43196869	circRNA_001519	3:122048175|122067285	circRNA_002111	8:83358407|83429055
circRNA_011809	5:34152199|34154697	circRNA_020648	13:160485672|160488823	circRNA_021317	GL894444.1:55142|74257	circRNA_011581	2:36255938|36326660
circRNA_008884	14:30954355|30956855	circRNA_013030	3:90576383|90579536	circRNA_014099	1:34740831|34759948	circRNA_018046	X:30958644|31029412
circRNA_012299	1:227185988|227188489	circRNA_008172	9:133341202|133344356	circRNA_005295	JH118921.1:16802|35922	circRNA_009562	5:70576776|70647555
circRNA_012524	13:35906752|35909253	circRNA_020581	13:34853744|34856898	circRNA_001786	6:25895725|25914846	circRNA_019500	13:158492539|158563363
circRNA_015052	8:43043712|43046213	circRNA_017853	4:142854258|142857414	circRNA_016305	7:73495104|73514226	circRNA_004127	3:84311601|84382433
circRNA_002782	10:19923823|19926325	circRNA_003476	14:144072146|144075305	circRNA_012718	15:54610049|54629173	circRNA_010580	2:91264177|91335022
circRNA_007033	13:99024292|99026795	circRNA_010628	3:91035889|91039048	circRNA_010349	13:8021216|8040351	circRNA_000993	15:115960373|116031233
circRNA_000863	14:141202621|141205125	circRNA_011396	15:36504524|36507684	circRNA_015836	18:36461492|36480633	circRNA_019156	7:53408460|53479321
circRNA_010912	GL892407.1:19130|21635	circRNA_020813	17:43359995|43363155	circRNA_005502	1:230022107|230041250	circRNA_006495	9:114262312|114333183
circRNA_011765	4:42128884|42131389	circRNA_006627	1:44537106|44540267	circRNA_017494	1:242717985|242737129	circRNA_019208	8:41708900|41779848
circRNA_009698	6:137301309|137303815	circRNA_018609	12:46904604|46907766	circRNA_004753	7:37118868|37138017	circRNA_012590	13:149599667|149670651
circRNA_019954	1:306221512|306224018	circRNA_005829	15:22765084|22768247	circRNA_016353	8:43018351|43037500	circRNA_002105	8:81613815|81684836
circRNA_009521	5:12442511|12445019	circRNA_007390	18:20672465|20675630	circRNA_018407	1:32091199|32110361	circRNA_016855	14:71155789|71226837
circRNA_002391	MT:1293|3804	circRNA_005880	15:154834827|154837993	circRNA_015180	1:86114517|86133686	circRNA_002804	10:35240929|35312084
circRNA_003119	13:43773987|43776498	circRNA_014622	10:49568556|49571722	circRNA_008813	13:130118894|130138064	circRNA_013412	8:113000638|113071813
circRNA_005211	GL892277.2:80612|83123	circRNA_003043	12:51824669|51827837	circRNA_018966	3:29618701|29637874	circRNA_000078	1:101482924|101554111
circRNA_014192	13:35035493|35038004	circRNA_010746	6:73120660|73123828	circRNA_000824	14:83832559|83851733	circRNA_002484	1:25518797|25589991
circRNA_015991	3:75715967|75718478	circRNA_015431	12:21028696|21031864	circRNA_013536	GL895038.1:60566|79742	circRNA_021006	4:34570313|34641574
circRNA_004844	7:110403366|110405878	circRNA_005583	11:15992153|15995322	circRNA_002606	1:145031571|145050748	circRNA_005348	X:73939459|74010804
circRNA_004503	5:98840510|98843023	circRNA_017020	2:66709085|66712255	circRNA_005558	10:49568556|49587733	circRNA_000070	1:86114517|86185934
circRNA_004554	6:50082427|50084940	circRNA_019371	1:115149639|115152811	circRNA_021104	6:69143298|69162481	circRNA_007333	16:78913434|78984905
circRNA_004724	7:14669088|14671601	circRNA_009450	4:44988069|44991243	circRNA_007929	7:73092645|73111840	circRNA_004895	8:31876392|31947867
circRNA_019068	5:48837142|48839655	circRNA_019900	1:16162076|16165251	circRNA_001805	6:57075630|57094838	circRNA_005811	14:136612215|136683701
circRNA_000079	1:101704036|101706552	circRNA_013181	5:72243881|72247057	circRNA_003812	18:9603361|9622572	circRNA_002186	9:11319664|11391169
circRNA_000872	14:145831570|145834086	circRNA_014231	14:6687380|6690556	circRNA_011774	4:73730788|73750002	circRNA_002575	1:128685283|128756792
circRNA_017397	X:85958495|85961011	circRNA_005103	9:40614116|40617293	circRNA_019820	8:41196878|41216093	circRNA_004958	8:81615914|81687472
circRNA_007449	2:60996624|60999141	circRNA_007366	17:55864210|55867387	circRNA_001509	3:120066344|120085560	circRNA_006643	1:80241844|80313405
circRNA_009587	5:88167732|88170249	circRNA_020440	1:188147577|188150754	circRNA_007960	7:117047645|117066861	circRNA_019449	11:26002135|26073707
circRNA_009635	6:53765096|53767613	circRNA_001967	7:50154804|50157983	circRNA_010187	1:75082709|75101925	circRNA_003515	15:52972603|53044224
circRNA_020532	11:21431232|21433749	circRNA_009217	2:8382933|8386112	circRNA_004849	7:119245301|119264520	circRNA_004670	6:138078891|138150517
circRNA_014548	9:32326743|32329262	circRNA_016014	3:109717662|109720842	circRNA_017105	4:45564608|45583827	circRNA_003749	17:14861638|14933267
circRNA_000089	1:116898188|116900708	circRNA_003651	15:147281324|147284505	circRNA_002124	8:103659931|103679154	circRNA_005498	1:225740193|225811866
circRNA_001616	4:102444968|102447488	circRNA_017144	5:70079561|70082742	circRNA_014917	13:144384384|144403607	circRNA_003640	15:131248910|131320591
circRNA_002567	1:126147153|126149673	circRNA_006060	3:11517980|11521162	circRNA_006883	11:26287974|26307201	circRNA_001669	5:145469|217179
circRNA_020694	14:81225874|81228394	circRNA_008596	11:21811028|21814210	circRNA_015149	1:23232804|23252035	circRNA_012716	15:52972502|53044224
circRNA_016198	6:18442659|18445181	circRNA_012287	1:201811150|201814335	circRNA_020901	2:104867971|104887207	circRNA_001090	17:14861638|14933362
circRNA_001563	4:40283338|40285863	circRNA_015083	12:47117039|47120224	circRNA_017213	6:157483705|157502942	circRNA_011455	15:148789557|148861405
circRNA_001924	6:148116282|148118812	circRNA_013730	8:93237744|93240931	circRNA_020036	14:49717331|49736569	circRNA_020484	1:285609337|285681202
circRNA_009283	2:108992874|108995405	circRNA_016899	15:80704325|80707512	circRNA_005098	9:40513547|40532789	circRNA_012676	14:104997908|105069802
circRNA_002159	8:143316894|143319426	circRNA_018196	16:71443428|71446616	circRNA_011300	14:4040395|4059637	circRNA_010172	1:32097053|32169111
circRNA_002644	1:195112533|195115065	circRNA_003239	13:166927031|166930220	circRNA_007276	15:118051711|118070954	circRNA_007579	3:97836618|97908682
circRNA_017051	2:145725178|145727710	circRNA_011215	13:37921511|37924700	circRNA_015414	12:11459082|11478325	circRNA_005703	13:103822708|103894828
circRNA_011560	2:2764259|2766792	circRNA_014201	13:56459055|56462244	circRNA_020447	1:202454865|202474119	circRNA_019683	3:112992563|113064811
circRNA_011387	14:145831552|145834086	circRNA_015832	18:31996976|32000165	circRNA_013500	9:133325099|133344356	circRNA_016721	10:30361792|30434042
circRNA_021090	6:12648433|12650967	circRNA_011382	14:140527878|140531069	circRNA_005729	13:148398444|148417703	circRNA_018395	X:35444248|35516528
circRNA_009239	2:43987819|43990356	circRNA_013434	9:10296782|10299973	circRNA_015484	13:24072755|24092025	circRNA_010501	15:154434946|154507230
circRNA_002310	9:144921409|144923948	circRNA_019091	6:18059965|18063156	circRNA_004121	3:83018888|83038160	circRNA_008483	1:280627976|280700379
circRNA_006926	12:23797987|23800527	circRNA_005691	13:81437153|81440345	circRNA_015638	14:78007100|78026373	circRNA_016189	6:10251024|10323446
circRNA_017083	3:109681872|109684412	circRNA_017393	X:57677653|57680846	circRNA_021371	X:70438343|70457620	circRNA_002905	11:24688121|24760594

circRNA_003793	17:55279559|55282100	circRNA_001437	3:72424177|72427371	circRNA_016738	11:25529781|25549061	circRNA_005430	1:94522915|94595460
circRNA_014805	16:78211482|78214023	circRNA_010509	16:58937245|58940439	circRNA_005213	GL892397.1:95227|114511	circRNA_006573	X:70426183|70498760
circRNA_019438	11:317743|320284	circRNA_005232	GL893560.1:9170|12365	circRNA_013036	3:98006243|98025528	circRNA_001856	6:97024076|97096660
circRNA_012004	8:71458415|71460958	circRNA_020135	3:43045786|43048981	circRNA_009583	5:87192177|87211468	circRNA_007072	13:155592365|155664955
circRNA_016986	18:22609842|22612385	circRNA_015129	5:93759998|93763194	circRNA_019827	8:89355787|89375078	circRNA_013648	9:97012313|97084931
circRNA_015540	13:119184171|119186715	circRNA_007911	7:53603281|53606479	circRNA_012600	13:170769226|170788519	circRNA_009991	9:88659895|88732539
circRNA_018330	7:14669056|14671601	circRNA_015802	17:64076770|64079968	circRNA_005519	1:278917340|278936634	circRNA_016084	4:74131965|74204622
circRNA_013666	10:54055539|54058085	circRNA_004059	3:16593897|16597096	circRNA_000905	15:43026464|43045759	circRNA_003894	2:35512252|35584957
circRNA_014374	2:121585729|121588275	circRNA_020384	1:61006639|61009838	circRNA_007514	2:139493399|139512697	circRNA_008929	14:81015098|81087819
circRNA_001441	3:74763811|74766358	circRNA_001704	5:38338234|38341434	circRNA_017752	2:49882526|49901833	circRNA_013369	8:20670183|20742913
circRNA_019474	13:36913102|36915650	circRNA_017694	15:157092775|157095975	circRNA_008706	13:4566107|4585416	circRNA_019434	10:59554486|59627241
circRNA_017414	X:144163959|144166508	circRNA_017655	14:146937329|146940530	circRNA_008179	9:144296741|144316052	circRNA_010373	13:66504318|66577148
circRNA_019496	13:142624887|142627436	circRNA_005431	1:94529065|94532267	circRNA_017419	1:13690314|13709627	circRNA_009908	8:146890664|146963502
circRNA_019137	6:147348510|147351061	circRNA_014651	14:16204288|16207491	circRNA_020744	15:85027126|85046450	circRNA_006217	5:70620860|70693708
circRNA_019897	X:143187223|143189775	circRNA_005311	X:8578423|8581627	circRNA_019620	2:30749214|30768544	circRNA_019336	X:14019906|14092759
circRNA_007744	5:46831512|46834065	circRNA_009256	2:69777995|69781199	circRNA_016078	4:72772834|72792173	circRNA_010238	1:229173474|229246387
circRNA_011738	3:135114875|135117428	circRNA_009486	4:102849982|102853187	circRNA_016007	3:102792837|102812180	circRNA_014634	12:59331765|59404683
circRNA_002855	10:63295479|63298033	circRNA_001390	3:18229680|18232887	circRNA_003109	13:42298387|42317731	circRNA_005944	18:18602505|18675443
circRNA_012411	11:3585232|3587787	circRNA_008219	GL896192.1:22918|26125	circRNA_008543	10:50698422|50717769	circRNA_016005	3:101136145|101209091
circRNA_001461	3:92450641|92453197	circRNA_011805	5:29908838|29912048	circRNA_010217	1:176827026|176846376	circRNA_014508	7:101076419|101149395
circRNA_013347	7:102945619|102948176	circRNA_012543	13:55531842|55535053	circRNA_014713	5:106590727|106610077	circRNA_000707	13:205921704|205994684
circRNA_001021	15:136774577|136777135	circRNA_008072	9:37750|40964	circRNA_001778	6:1576368|1595723	circRNA_010795	7:65150234|65223310
circRNA_013094	4:44538833|44541391	circRNA_004560	6:64542087|64545302	circRNA_004973	8:90954785|90974141	circRNA_015859	2:3036679|3109853
circRNA_019152	7:21302926|21305484	circRNA_000013	1:16124424|16127640	circRNA_000705	13:205921704|205941062	circRNA_008963	14:136757920|136831101
circRNA_017668	15:85026832|85029391	circRNA_012665	14:78244907|78248123	circRNA_014959	1:45632972|45652333	circRNA_018891	18:59484562|59557766
circRNA_011255	13:130358254|130360814	circRNA_017057	2:153881899|153885118	circRNA_000106	1:127622340|127641705	circRNA_000447	12:13899562|13972797
circRNA_017333	9:58368415|58370975	circRNA_007396	18:27408621|27411842	circRNA_012602	13:184211055|184230427	circRNA_019279	9:82189438|82262760
circRNA_005441	1:119482605|119485168	circRNA_012779	15:133589366|133592588	circRNA_015352	10:47067162|47086534	circRNA_015095	4:67947307|68020671
circRNA_013192	5:87161461|87164024	circRNA_017749	2:29213178|29216402	circRNA_017125	4:117903152|117922524	circRNA_002757	1:297336189|297409578
circRNA_010276	10:31875514|31878079	circRNA_010017	9:137075851|137079077	circRNA_014160	10:54112639|54132013	circRNA_018350	8:71895225|71968747
circRNA_004835	7:100815934|100818500	circRNA_015066	GL895864.2:56902|60128	circRNA_018736	14:65777770|65797146	circRNA_018093	1:241875289|241948825
circRNA_011887	6:83025822|83028390	circRNA_016698	1:283088447|283091675	circRNA_006055	3:5408827|5428207	circRNA_004574	6:77344573|77418217
circRNA_005121	9:57728054|57730623	circRNA_019324	GL896381.1:8217|11445	circRNA_001285	2:83782392|83801773	circRNA_003102	13:35647658|35721305
circRNA_008506	10:11709199|11711768	circRNA_006952	12:59263043|59266273	circRNA_000246	1:275800353|275819743	circRNA_018997	3:103102738|103176394
circRNA_005059	9:11798735|11801305	circRNA_008336	1:86577009|86580240	circRNA_015757	16:36664765|36684156	circRNA_019824	8:81613815|81687472
circRNA_007643	4:39885800|39888370	circRNA_008501	1:303289220|303292451	circRNA_010191	1:86210890|86230282	circRNA_007797	6:25841148|25914846
circRNA_008157	9:116180992|116183562	circRNA_003648	15:145610977|145614211	circRNA_015403	11:64858709|64878101	circRNA_003133	13:67530900|67604724
circRNA_010785	7:21040004|21042577	circRNA_002955	12:12135663|12138898	circRNA_006991	13:42358748|42378144	circRNA_018656	13:119205106|119278934
circRNA_019420	10:18748058|18750631	circRNA_011591	2:50802485|50805720	circRNA_001074	16:57101626|57121028	circRNA_018795	15:94293320|94367189
circRNA_004536	6:18464132|18466706	circRNA_019378	1:130427075|130430314	circRNA_008889	14:33035185|33054591	circRNA_012321	1:282666520|282740392
circRNA_013902	3:107583903|107586477	circRNA_004024	2:139112346|139115586	circRNA_006636	1:60905557|60924967	circRNA_013700	18:36461492|36535413
circRNA_001386	3:11510637|11513212	circRNA_020684	14:59244754|59247994	circRNA_000385	11:12807920|12827332	circRNA_011670	3:31879020|31952945
circRNA_000295	10:18361726|18364302	circRNA_013407	8:109429079|109432321	circRNA_016589	X:51889184|51908597	circRNA_015893	2:83796563|83870489
circRNA_008657	12:27541665|27544241	circRNA_011650	2:153844478|153847722	circRNA_000386	11:12807920|12827335	circRNA_011002	1:135303493|135377427
circRNA_011029	1:188418093|188420669	circRNA_008431	1:202520209|202523454	circRNA_003389	14:78234225|78253641	circRNA_020596	13:68061202|68135137
circRNA_018437	1:124986333|124988911	circRNA_000323	10:37142979|37146225	circRNA_004876	8:3341344|3360764	circRNA_017036	2:104437584|104511554
circRNA_019654	2:140902693|140905271	circRNA_014298	15:101665956|101669202	circRNA_018598	12:37126733|37146163	circRNA_011750	4:31389361|31463369
circRNA_001468	3:98680367|98682946	circRNA_010752	6:88641987|88645235	circRNA_017546	11:25509167|25528603	circRNA_007501	2:105082209|105156268
circRNA_006828	10:37118200|37120779	circRNA_018454	1:143932187|143935435	circRNA_001192	18:53765188|53784629	circRNA_011384	14:142775864|142850048
circRNA_018033	GL896111.1:1202|3782	circRNA_005365	X:115944520|115947769	circRNA_013801	12:15573276|15592721	circRNA_010720	5:87192177|87266366
circRNA_000564	13:34916547|34919128	circRNA_006331	7:36615306|36618555	circRNA_008977	14:148266744|148286190	circRNA_001166	18:18641519|18715721
circRNA_001791	6:32419351|32421933	circRNA_005114	9:44922267|44925517	circRNA_020545	12:15256360|15275808	circRNA_006976	13:28146354|28220691

circRNA_006889	11:49599584|49602166	circRNA_006399	8:43023439|43026689	circRNA_009593	5:93481383|93500837	circRNA_010807	7:97005367|97079790
circRNA_015689	15:53400417|53403000	circRNA_004135	3:85291843|85295094	circRNA_007589	3:106700866|106720325	circRNA_011947	7:61558755|61633213
circRNA_018171	14:136308649|136311233	circRNA_015106	9:51507059|51510311	circRNA_014995	14:101914851|101934314	circRNA_003855	18:44530678|44605192
circRNA_020049	14:122681530|122684116	circRNA_017165	6:37790246|37793498	circRNA_012373	10:49576020|49595487	circRNA_015468	12:62458832|62533382
circRNA_007582	3:102273355|102275943	circRNA_019386	1:152630400|152633652	circRNA_019936	1:202793869|202813336	circRNA_008391	1:157816758|157891426
circRNA_005643	13:3080251|3082840	circRNA_008341	1:98110332|98113585	circRNA_009385	3:97986911|98006379	circRNA_018376	9:132220492|132295195
circRNA_000559	13:33955466|33958057	circRNA_018374	9:81541641|81544896	circRNA_013306	7:20297317|20316788	circRNA_016154	5:64081960|64156704
circRNA_009225	2:20684777|20687369	circRNA_016965	17:55271174|55274430	circRNA_003336	14:34675990|34695463	circRNA_019446	11:19661652|19736422
circRNA_020475	1:278556233|278558826	circRNA_012231	1:101717714|101720971	circRNA_018259	3:109447083|109466558	circRNA_003568	15:85415559|85490341
circRNA_000432	11:81632780|81635374	circRNA_013621	1:264890623|264893881	circRNA_011341	14:77056392|77075869	circRNA_015743	16:5181134|5255951
circRNA_005645	13:18707840|18710434	circRNA_018003	9:115700511|115703772	circRNA_008622	11:72326151|72345641	circRNA_000544	13:24296721|24371577
circRNA_005106	9:40859458|40862053	circRNA_004248	4:36469354|36472616	circRNA_007270	15:106836857|106856355	circRNA_001366	2:147137211|147212170
circRNA_009431	4:15262358|15264955	circRNA_013003	3:42973499|42976762	circRNA_004012	2:131561222|131580729	circRNA_002442	X:126771940|126846915
circRNA_011527	17:52808443|52811041	circRNA_014177	12:20316409|20319673	circRNA_009320	2:154041920|154061428	circRNA_015803	17:67404760|67479794
circRNA_014708	5:3468414|3471014	circRNA_001614	4:98125120|98128385	circRNA_010247	1:254962897|254982407	circRNA_017109	4:53440076|53515135
circRNA_016479	9:79218044|79220647	circRNA_014919	14:30841817|30845082	circRNA_015764	16:47791556|47811066	circRNA_002894	11:19661652|19736732
circRNA_001330	2:120420909|120423513	circRNA_019390	1:182105237|182108503	circRNA_017869	5:97667033|97686545	circRNA_010142	X:89912449|89987569
circRNA_009392	3:104168480|104171085	circRNA_015875	2:31206266|31209534	circRNA_018095	1:254769627|254789145	circRNA_016592	X:67430148|67505417
circRNA_003259	13:206985351|206987957	circRNA_016480	9:79221081|79224350	circRNA_004233	4:15915883|15935405	circRNA_019241	8:144771186|144846497
circRNA_001100	17:39442884|39445491	circRNA_004868	8:413584|416854	circRNA_000664	13:142609589|142629113	circRNA_020200	5:59878144|59953531
circRNA_011917	6:148463817|148466426	circRNA_006928	12:25021891|25025161	circRNA_000431	11:81567206|81586734	circRNA_018238	2:108558988|108634398
circRNA_014790	14:30933428|30936038	circRNA_016746	12:151796|155066	circRNA_002552	1:114030308|114049837	circRNA_001210	2:3034318|3109853
circRNA_021063	5:72572851|72575462	circRNA_014860	7:9900963|9904235	circRNA_002694	1:229343179|229362710	circRNA_016207	6:39642248|39717788
circRNA_008925	14:77540095|77542707	circRNA_017567	12:36542951|36546223	circRNA_003603	15:112138368|112157900	circRNA_004974	8:91436782|91512346
circRNA_019133	6:138823936|138826548	circRNA_015816	18:11035272|11038545	circRNA_010799	7:73073215|73092750	circRNA_006111	3:120066344|120141921
circRNA_019491	13:126893678|126896290	circRNA_017650	14:123111964|123115238	circRNA_007849	6:99525347|99544888	circRNA_007880	6:147355095|147430771
circRNA_011745	4:20188602|20191216	circRNA_018157	14:58823773|58827050	circRNA_019121	6:108611407|108630949	circRNA_014840	4:136805197|136880888
circRNA_020483	1:284708509|284711123	circRNA_014039	11:75129058|75132336	circRNA_016868	14:110126707|110146252	circRNA_007460	2:72132914|72208618
circRNA_017606	13:117695880|117698496	circRNA_018200	17:46270727|46274006	circRNA_014613	1:188117293|188136843	circRNA_006216	5:70617986|70693708
circRNA_010277	10:33075848|33078465	circRNA_008830	13:146916891|146920171	circRNA_014874	8:44840811|44860365	circRNA_006646	1:86878851|86954593
circRNA_011112	10:47448975|47451592	circRNA_009918	9:9545016|9548296	circRNA_017098	4:36253818|36273373	circRNA_020195	5:42104599|42180342
circRNA_017439	1:86114517|86117134	circRNA_015547	13:131341476|131344756	circRNA_017482	1:200784183|200803738	circRNA_016980	18:18602505|18678275
circRNA_020749	15:89945975|89948592	circRNA_013961	9:52649358|52652640	circRNA_019625	2:71193761|71213316	circRNA_011666	3:24280654|24356481
circRNA_009425	3:135290999|135293617	circRNA_001083	17:9339772|9343055	circRNA_002524	1:83281460|83301016	circRNA_000044	1:41655344|41731269
circRNA_009350	3:43044227|43046846	circRNA_010598	2:142541102|142544386	circRNA_003297	14:10615590|10635146	circRNA_001112	17:45173339|45249269
circRNA_018942	2:125526455|125529074	circRNA_009362	3:57699119|57702404	circRNA_016728	10:63455784|63475346	circRNA_003567	15:85406874|85482828
circRNA_004188	3:119657721|119660341	circRNA_014705	4:129946021|129949307	circRNA_009866	8:89404830|89424394	circRNA_009564	5:70588009|70663997
circRNA_015907	2:106892933|106895553	circRNA_008580	11:8863156|8866443	circRNA_019634	2:87952595|87972159	circRNA_002494	1:41655233|41731269
circRNA_016664	1:179464490|179467111	circRNA_018284	5:26807163|26810451	circRNA_011533	18:5614948|5634518	circRNA_000030	1:28454987|28531105
circRNA_017377	GL896000.1:906|3528	circRNA_016285	7:43282966|43286255	circRNA_002138	8:123995550|124015123	circRNA_004241	4:31387169|31463369
circRNA_012257	1:134653309|134655932	circRNA_003165	13:86999951|87003242	circRNA_004701	6:148116282|148135855	circRNA_012860	18:8579557|8655777
circRNA_019704	4:102442529|102445154	circRNA_011758	4:37756328|37759621	circRNA_006498	9:115686007|115705584	circRNA_017545	11:24202794|24279091
circRNA_009199	18:46756245|46758871	circRNA_001890	6:108611407|108614701	circRNA_019911	1:62517228|62536805	circRNA_016826	13:170769226|170845597
circRNA_015275	1:230910210|230912836	circRNA_013690	14:24809694|24812988	circRNA_012852	17:65833013|65852595	circRNA_011286	13:205444308|205520681
circRNA_016003	3:84990686|84993312	circRNA_015582	13:210782621|210785915	circRNA_017854	5:14001255|14020839	circRNA_010661	4:41114627|41191106
circRNA_006239	5:91493443|91496070	circRNA_021360	X:40486596|40489890	circRNA_006865	11:12807920|12827511	circRNA_009688	6:109717487|109793974
circRNA_011488	16:47050901|47053530	circRNA_003251	13:190890857|190894152	circRNA_003867	18:57730674|57750266	circRNA_003842	18:26303145|26379639
circRNA_002435	X:114370598|114373230	circRNA_012910	2:30034059|30037356	circRNA_013824	13:131006932|131026525	circRNA_014350	18:59442183|59518686
circRNA_006195	5:23508237|23510869	circRNA_014409	3:122027590|122030888	circRNA_014609	1:63729107|63748701	circRNA_020615	13:119202413|119278934
circRNA_004138	3:90361781|90364414	circRNA_010588	2:119280884|119284184	circRNA_018023	GL893784.1:6605|26203	circRNA_019398	1:215651154|215727733
circRNA_003139	13:73300581|73303215	circRNA_000826	14:92380920|92384221	circRNA_016860	14:81920776|81940377	circRNA_018993	3:97836618|97913205
circRNA_006298	6:125478593|125481232	circRNA_007955	7:104451564|104454866	circRNA_013650	9:137075851|137095457	circRNA_013394	8:82954008|83030613

circRNA_014835	4:39929686|39932326	circRNA_011325	14:48121030|48124333	circRNA_015220	1:161752061|161771667	circRNA_008303	1:28454469|28531105
circRNA_019563	15:53434689|53437329	circRNA_003586	15:101520858|101524162	circRNA_010940	X:85941401|85961011	circRNA_012615	14:8620295|8696994
circRNA_009211	18:60717576|60720217	circRNA_012053	9:28144692|28147997	circRNA_000434	11:86942801|86962417	circRNA_013327	7:62014264|62091118
circRNA_011893	6:90323632|90326273	circRNA_019492	13:131521588|131524895	circRNA_009163	17:49628660|49648277	circRNA_010896	9:115606023|115682911
circRNA_015082	12:23264157|23266798	circRNA_017461	1:142955184|142958492	circRNA_006375	7:124977818|124997437	circRNA_012345	10:17127705|17204667
circRNA_017935	7:65449058|65451703	circRNA_021164	7:60361336|60364644	circRNA_003546	15:75475656|75495281	circRNA_016483	9:82185790|82262760
circRNA_005147	9:102426168|102428814	circRNA_013423	8:141476121|141479430	circRNA_008261	X:77199964|77219590	circRNA_008828	13:144478832|144555825
circRNA_011082	10:15897937|15900583	circRNA_009790	7:91959137|91962447	circRNA_011961	7:97852317|97871951	circRNA_012620	14:21515998|21593078
circRNA_015498	13:36792576|36795222	circRNA_010400	13:149875730|149879040	circRNA_015226	1:181145447|181165093	circRNA_008153	9:115608962|115686087
circRNA_008049	8:112784240|112786887	circRNA_014870	7:119686710|119690020	circRNA_006113	3:120136072|120155720	circRNA_001713	5:48802052|48879189
circRNA_012753	15:111620359|111623007	circRNA_007992	8:32687757|32691071	circRNA_008747	13:43406481|43426130	circRNA_007508	2:119165841|119242988
circRNA_003923	2:70193426|70196075	circRNA_018771	14:146683070|146686384	circRNA_006154	4:88588344|88607994	circRNA_010811	7:109744938|109822191
circRNA_005400	1:6438466|6441115	circRNA_016368	8:57807091|57810406	circRNA_011832	5:86068811|86088461	circRNA_018168	14:117030293|117107550
circRNA_014851	6:98763605|98766255	circRNA_006876	11:22243068|22246384	circRNA_002828	10:49647635|49667287	circRNA_013566	X:16989229|17066502
circRNA_012326	1:293355657|293358309	circRNA_007764	5:77654672|77657988	circRNA_015125	3:46253973|46273625	circRNA_001698	5:32956398|33033688
circRNA_020113	2:15683561|15686214	circRNA_009279	2:105082209|105085525	circRNA_006225	5:83062597|83082256	circRNA_010900	9:132314930|132392257
circRNA_006808	10:13624315|13626969	circRNA_013254	6:88253944|88257261	circRNA_012719	15:55337963|55357622	circRNA_010910	9:146221304|146298732
circRNA_010938	X:78203745|78206401	circRNA_018645	13:87162721|87166038	circRNA_005293	JH118921.1:9922|29587	circRNA_010193	1:89739015|89816565
circRNA_014630	12:161887|164543	circRNA_004034	2:146512839|146516158	circRNA_008285	X:142762776|142782443	circRNA_001195	18:59484562|59562118
circRNA_014697	3:125883715|125886371	circRNA_007209	14:148286084|148289403	circRNA_010405	13:160284404|160304071	circRNA_002066	8:20670183|20747741
circRNA_005259	GL894963.1:306|2964	circRNA_019082	5:83967614|83970933	circRNA_002091	8:58763894|58783562	circRNA_012222	1:84973176|85050736
circRNA_006127	4:31712187|31714845	circRNA_018074	1:64731985|64735305	circRNA_019744	5:106543058|106562733	circRNA_014758	X:126803212|126880812
circRNA_019437	11:250979|253640	circRNA_007715	5:4607780|4611101	circRNA_003504	15:22565123|22584801	circRNA_001275	2:78493987|78571606
circRNA_020561	12:40294951|40297612	circRNA_013768	1:44439811|44443134	circRNA_011188	12:62428106|62447784	circRNA_019919	1:85731441|85809105
circRNA_013373	8:30952024|30954686	circRNA_007841	6:87965642|87968966	circRNA_014033	X:16245778|16265456	circRNA_009157	17:46989621|47067299
circRNA_002265	9:96050798|96053461	circRNA_008003	8:40606599|40609923	circRNA_003660	16:19127489|19147169	circRNA_003539	15:66886362|66964066
circRNA_015941	2:147048493|147051156	circRNA_015942	2:147935256|147938580	circRNA_000507	12:52161469|52181151	circRNA_011022	1:161704139|161781848
circRNA_017765	2:87316153|87318816	circRNA_005442	1:125216286|125219611	circRNA_015195	1:105706329|105726014	circRNA_001548	4:16999382|17077103
circRNA_001970	7:54527454|54530118	circRNA_006191	5:8998035|9001360	circRNA_011206	13:32176941|32196629	circRNA_011997	8:39412477|39490335
circRNA_005511	1:249496076|249498741	circRNA_007307	16:29112553|29115878	circRNA_017783	3:11581965|11601653	circRNA_011858	6:10493945|10571820
circRNA_007687	4:116732431|116735096	circRNA_009489	4:107406864|107410192	circRNA_021111	6:85391733|85411421	circRNA_011059	1:270324830|270402708
circRNA_019493	13:132319701|132322366	circRNA_014396	3:74760760|74764088	circRNA_002585	1:134599618|134619308	circRNA_001666	4:141826350|141904247
circRNA_003217	13:143981035|143983701	circRNA_020105	18:55311557|55314885	circRNA_012870	18:24402794|24422485	circRNA_016818	13:142522109|142600017
circRNA_014448	5:52435212|52437879	circRNA_002125	8:103675822|103679154	circRNA_001690	5:16675258|16694950	circRNA_015709	15:85404895|85482828
circRNA_012921	2:49909375|49912043	circRNA_005689	13:81257901|81261233	circRNA_005806	14:120768685|120788379	circRNA_017609	13:145609695|145687658
circRNA_016392	8:108430091|108432759	circRNA_003852	18:43942640|43945974	circRNA_005573	10:77181859|77201554	circRNA_005362	X:108901528|108979510
circRNA_019908	1:59750078|59752746	circRNA_012951	2:87312978|87316313	circRNA_001325	2:119165841|119185540	circRNA_013864	16:49629972|49708018
circRNA_005986	2:65359607|65362276	circRNA_001049	16:34224789|34228126	circRNA_017199	6:125479400|125499100	circRNA_014267	14:142778961|142857217
circRNA_008068	8:144719451|144722121	circRNA_009732	7:23927744|23931081	circRNA_007155	14:83665860|83685562	circRNA_005086	9:29210583|29288870
circRNA_006330	7:33113472|33116145	circRNA_003665	16:22554303|22557643	circRNA_011946	7:60322802|60342508	circRNA_006328	7:24644089|24722398
circRNA_006771	1:269065940|269068614	circRNA_016246	6:100723128|100726469	circRNA_011406	15:62969256|62988963	circRNA_015869	2:20082252|20160570
circRNA_010808	7:103742240|103744915	circRNA_011798	5:3231074|3234416	circRNA_018641	13:81528678|81548387	circRNA_004761	7:46279250|46357590
circRNA_003756	17:30003927|30006603	circRNA_009405	3:119587849|119591192	circRNA_014492	7:60322802|60342512	circRNA_014715	6:10267853|10346209
circRNA_015155	1:31500055|31502732	circRNA_010638	3:114071752|114075099	circRNA_013749	14:110515633|110535349	circRNA_009938	9:37845752|37924125
circRNA_014884	9:25673758|25676436	circRNA_004476	5:79715412|79718761	circRNA_002886	11:11217082|11236804	circRNA_006769	1:265773697|265852140
circRNA_017720	17:36484536|36487214	circRNA_015297	1:267405743|267409093	circRNA_007686	4:113522229|113541958	circRNA_009722	7:2977563|3056024
circRNA_008084	9:13256666|13259345	circRNA_018192	16:51615488|51618839	circRNA_015272	1:230871275|230891012	circRNA_007796	6:25836379|25914846
circRNA_016878	14:127952549|127955228	circRNA_019447	11:19712163|19715514	circRNA_012449	11:75102199|75121939	circRNA_019555	14:142775864|142854338
circRNA_018579	11:25551967|25554646	circRNA_016017	3:109787455|109790807	circRNA_018133	13:119216629|119236371	circRNA_010796	7:65153291|65231776
circRNA_015218	1:146236293|146238974	circRNA_019504	13:183909656|183913008	circRNA_013630	14:132237197|132256940	circRNA_011297	13:211131404|211209889
circRNA_000157	1:175574069|175576751	circRNA_014681	2:90657589|90660943	circRNA_008944	14:112932617|112952367	circRNA_000196	1:216220855|216299386
circRNA_006541	GL896549.1:20832|23514	circRNA_017696	16:18564948|18568302	circRNA_009769	7:73042261|73062014	circRNA_019738	5:79048194|79126790

circRNA_006747	1:215715817|215718501	circRNA_003278	13:212937526|212940882	circRNA_021290	9:133488798|133508551	circRNA_012405	11:472454|551063
circRNA_019119	6:102734062|102736746	circRNA_008840	13:167164236|167167593	circRNA_020652	13:186835561|186855321	circRNA_001628	4:110255147|110333830
circRNA_016619	1:10220083|10222768	circRNA_017015	2:50771335|50774694	circRNA_019871	GL894654.1:16138|35901	circRNA_006329	7:24644590|24723289
circRNA_000804	14:77581776|77584462	circRNA_005617	12:20113464|20116824	circRNA_017535	10:76256259|76276024	circRNA_001781	6:7616959|7695730
circRNA_020768	15:154968541|154971228	circRNA_008736	13:37949748|37953108	circRNA_006501	9:120349846|120369612	circRNA_010155	X:124200974|124279826
circRNA_015478	13:20950127|20952815	circRNA_001272	2:76183437|76186799	circRNA_018909	2:53089550|53109320	circRNA_017673	15:86607064|86685940
circRNA_002541	1:94575046|94577735	circRNA_012880	18:43096881|43100243	circRNA_013114	4:90439905|90459676	circRNA_007032	13:98113084|98191973
circRNA_004523	6:12077068|12079757	circRNA_018111	11:18532986|18536348	circRNA_013261	6:92050674|92070446	circRNA_006174	4:110254845|110333830
circRNA_003764	17:33122220|33124910	circRNA_010960	1:32069210|32072573	circRNA_021286	9:120349846|120369627	circRNA_017874	6:10267201|10346209
circRNA_001073	16:55612240|55614932	circRNA_011492	16:51185829|51189192	circRNA_002193	9:13299241|13319023	circRNA_003395	14:81282853|81361983
circRNA_020006	13:104406588|104409282	circRNA_015154	1:30945677|30949042	circRNA_006013	2:90927901|90947683	circRNA_001044	16:29541066|29620216
circRNA_003605	15:112540074|112542770	circRNA_002797	10:31246385|31249752	circRNA_015522	13:84699527|84719315	circRNA_006488	9:85842426|85921589
circRNA_016457	9:44395930|44398626	circRNA_000537	13:20327408|20330776	circRNA_001734	5:70319577|70339367	circRNA_013692	14:136879801|136959046
circRNA_005005	8:126790169|126792866	circRNA_006570	X:67157335|67160703	circRNA_018048	X:43179268|43199058	circRNA_008768	13:74656936|74736227
circRNA_005962	2:61301|63998	circRNA_009924	9:19308688|19312056	circRNA_016903	15:85355707|85375504	circRNA_020277	7:128927160|129006467
circRNA_004447	5:60370375|60373073	circRNA_014987	13:119829725|119833093	circRNA_001861	6:99220825|99240623	circRNA_007671	4:88607838|88687228
circRNA_013945	7:97412257|97414957	circRNA_017003	2:11319383|11322751	circRNA_005670	13:42372497|42392300	circRNA_003845	18:32741428|32820842
circRNA_003088	13:29169188|29171889	circRNA_003445	14:122307403|122310772	circRNA_021062	5:72221358|72241163	circRNA_005929	17:44465973|44545401
circRNA_009202	18:49466488|49469189	circRNA_007343	17:14563617|14566987	circRNA_007910	7:49725403|49745213	circRNA_008992	15:36264244|36343681
circRNA_011683	3:56415476|56418177	circRNA_000284	10:14994890|14998261	circRNA_011386	14:145714289|145734102	circRNA_001799	6:47257975|47337425
circRNA_016776	13:24089323|24092025	circRNA_000476	12:37135028|37138400	circRNA_016952	17:5196690|5216506	circRNA_004469	5:74124973|74204433
circRNA_009384	3:97986911|97989615	circRNA_017871	5:104209520|104212893	circRNA_013531	GL894654.1:35739|55560	circRNA_000029	1:25510519|25589991
circRNA_015887	2:70028276|70030981	circRNA_003123	13:50655026|50658407	circRNA_015443	12:32752030|32771852	circRNA_013038	3:100513972|100593451
circRNA_008197	GL893762.1:62476|65182	circRNA_000314	10:33646093|33649475	circRNA_010145	X:105384438|105404261	circRNA_006630	1:48408106|48487606
circRNA_012611	13:212947616|212950322	circRNA_010343	12:44859641|44863025	circRNA_020047	14:116328948|116348772	circRNA_009619	6:25836379|25915917
circRNA_000851	14:118365299|118368007	circRNA_013742	X:4900819|4904203	circRNA_002495	1:45701230|45721056	circRNA_019419	10:17067380|17146941
circRNA_006815	10:19943997|19946705	circRNA_003320	14:31262408|31265793	circRNA_010396	13:132977618|132997449	circRNA_007015	13:86459133|86538699
circRNA_008733	13:35728481|35731189	circRNA_010482	15:91107490|91110875	circRNA_012775	15:128059052|128078889	circRNA_019964	10:53350655|53430254
circRNA_010611	3:29694202|29696912	circRNA_016050	4:16774119|16777504	circRNA_011269	13:146886105|146905943	circRNA_012087	9:135242282|135321917
circRNA_018807	15:124776531|124779242	circRNA_017536	11:3596648|3600033	circRNA_000536	13:19726699|19746539	circRNA_013420	8:134040804|134120482
circRNA_007061	13:143414634|143417347	circRNA_010828	8:43018351|43021737	circRNA_009088	16:27092137|27111983	circRNA_018504	1:244253941|244333662
circRNA_019335	X:13417556|13420269	circRNA_008397	1:169138678|169142065	circRNA_013715	4:81206146|81225995	circRNA_013221	6:14579358|14659216
circRNA_006238	5:90619378|90622092	circRNA_012420	11:7652076|7655463	circRNA_015425	12:14691671|14711533	circRNA_000960	15:86591592|86671532
circRNA_019892	X:101629366|101632080	circRNA_014145	1:282203470|282206858	circRNA_016837	14:29836044|29855910	circRNA_011569	2:17691075|17771066
circRNA_011607	2:83183717|83186432	circRNA_009560	5:70335978|70339367	circRNA_001750	5:79698894|79718761	circRNA_003407	14:92425152|92505193
circRNA_014628	11:21001197|21003912	circRNA_000952	15:85315348|85318738	circRNA_014905	1:15928649|15948518	circRNA_020936	3:24272672|24352716
circRNA_016071	4:66627423|66630139	circRNA_001174	18:25506902|25510292	circRNA_004818	7:91932958|91952828	circRNA_006215	5:70515829|70595881
circRNA_020231	6:104484726|104487442	circRNA_003572	15:86615927|86619319	circRNA_020164	4:30354679|30374549	circRNA_008205	GL894284.2:40217|120275
circRNA_001111	17:44400340|44403057	circRNA_004445	5:54839918|54843310	circRNA_006269	6:73284406|73304281	circRNA_013485	9:115606023|115686087
circRNA_004043	2:149809923|149812640	circRNA_000567	13:35122058|35125451	circRNA_006830	10:37657908|37677784	circRNA_004609	6:91839899|91919964
circRNA_008860	13:210047704|210050422	circRNA_011461	16:6456843|6460236	circRNA_003038	12:49350315|49370193	circRNA_006198	5:36925045|37005111
circRNA_014175	12:2289946|2292664	circRNA_020102	18:35925585|35928978	circRNA_018455	1:145416172|145436055	circRNA_012207	1:38479516|38559582
circRNA_011898	6:104484723|104487442	circRNA_000827	14:94811706|94815100	circRNA_014117	1:134754832|134774718	circRNA_000521	12:62443557|62523689
circRNA_020905	2:119260427|119263146	circRNA_017394	X:68882913|68886307	circRNA_003089	13:29719703|29739591	circRNA_001209	2:3029587|3109853
circRNA_014112	1:124861734|124864455	circRNA_006734	1:200703767|200707164	circRNA_001321	2:112811828|112831718	circRNA_010533	17:67399523|67479794
circRNA_020056	15:73492950|73495671	circRNA_019423	10:31988490|31991887	circRNA_020974	3:92448986|92468880	circRNA_007852	6:101724150|101804423
circRNA_007608	3:122011566|122014288	circRNA_004272	4:51268524|51271923	circRNA_019583	16:23576710|23596614	circRNA_012259	1:135829826|135910108
circRNA_012482	12:35930283|35933005	circRNA_018727	14:51693016|51696415	circRNA_009182	18:15002914|15022819	circRNA_018686	13:191340935|191421236
circRNA_008095	9:29830958|29833681	circRNA_020670	14:31063858|31067257	circRNA_002762	1:301441603|301461510	circRNA_007879	6:147350407|147430771
circRNA_011274	13:150216320|150219043	circRNA_014586	GL895238.2:49303|52703	circRNA_017878	6:35452113|35472022	circRNA_017963	8:104001015|104081397
circRNA_009408	3:121254917|121257642	circRNA_018187	15:116990412|116993815	circRNA_002696	1:231032079|231051989	circRNA_006633	1:57195566|57275978
circRNA_008850	13:202668926|202671653	circRNA_006678	1:125143608|125147013	circRNA_021303	GL892789.1:74645|94569	circRNA_001961	7:35879122|35959559

circRNA_008906	14:51755345|51758074	circRNA_013528	GL894359.1:13198|16603	circRNA_000866	14:142837282|142857217	circRNA_017863	5:71388114|71468597
circRNA_017386	X:17707882|17710613	circRNA_005370	X:121770822|121774229	circRNA_012272	1:165572669|165592604	circRNA_010759	6:125859359|125939863
circRNA_003959	2:87154621|87157353	circRNA_015776	16:56571245|56574653	circRNA_004924	8:55114181|55134117	circRNA_007308	16:29539650|29620216
circRNA_013593	X:122545608|122548340	circRNA_001759	5:88166840|88170249	circRNA_006795	1:301515256|301535195	circRNA_010510	16:59797585|59878165
circRNA_000195	1:215885411|215888149	circRNA_000423	11:56283465|56286876	circRNA_007371	17:58794081|58814020	circRNA_001864	6:99236964|99317549
circRNA_020932	3:11029573|11032311	circRNA_010597	2:139584754|139588165	circRNA_021028	4:98108443|98128385	circRNA_019719	5:23083310|23163902
circRNA_003046	12:52161469|52164213	circRNA_003803	18:5545554|5548966	circRNA_014539	8:141381057|141401000	circRNA_019561	15:34770888|34851487
circRNA_006982	13:33302594|33305339	circRNA_002818	10:46139628|46143041	circRNA_004934	8:58274497|58294450	circRNA_003899	2:36251001|36331608
circRNA_004719	7:14125735|14128481	circRNA_003545	15:74304127|74307541	circRNA_014521	8:32691009|32710967	circRNA_004591	6:82938912|83019536
circRNA_010569	2:52206018|52208765	circRNA_010566	2:42388199|42391615	circRNA_020340	GL894502.2:9089|29047	circRNA_002296	9:139383664|139464306
circRNA_014918	14:29911026|29913773	circRNA_005554	10:44196320|44199737	circRNA_021005	4:31694887|31714845	circRNA_021177	7:109744938|109825592
circRNA_005088	9:29740807|29743556	circRNA_007243	15:66307639|66311057	circRNA_012007	8:77435427|77455389	circRNA_004590	6:82938849|83019536
circRNA_000010	1:14083797|14086548	circRNA_010909	9:144296741|144300159	circRNA_001152	18:10792104|10812072	circRNA_009897	8:134477803|134558516
circRNA_020630	13:133974871|133977625	circRNA_014488	7:33583169|33586587	circRNA_016934	16:37498946|37518916	circRNA_004194	3:120081729|120162510
circRNA_012681	14:115758353|115761108	circRNA_017455	1:128375717|128379137	circRNA_001410	3:40257421|40277398	circRNA_010744	6:61842290|61923073
circRNA_008338	1:87749486|87752243	circRNA_019038	4:81056213|81059633	circRNA_011027	1:183342472|183362450	circRNA_013484	9:115605241|115686087
circRNA_000068	1:82801008|82803766	circRNA_005273	GL896291.1:17360|20781	circRNA_008308	1:38715675|38735658	circRNA_021374	X:77162204|77243106
circRNA_010388	13:117603168|117605927	circRNA_014161	10:54455236|54458657	circRNA_011512	17:22552288|22572272	circRNA_010301	11:5193847|5274850
circRNA_010466	15:18995367|18998126	circRNA_017008	2:17314802|17318223	circRNA_010197	1:103206286|103226272	circRNA_013197	5:88358137|88439154
circRNA_000830	14:98514587|98517347	circRNA_020929	2:154162744|154166165	circRNA_011808	5:34134710|34154697	circRNA_015635	14:77094824|77175956
circRNA_014265	14:122838772|122841532	circRNA_019896	X:124212398|124215820	circRNA_015592	14:10459275|10479267	circRNA_013151	5:23083733|23164933
circRNA_020027	13:217078919|217081679	circRNA_005222	GL892789.1:71280|74703	circRNA_001784	6:16653258|16673251	circRNA_012317	1:270156380|270237597
circRNA_004365	4:129474350|129477112	circRNA_021339	GL896549.1:11995|15418	circRNA_012324	1:292685679|292705673	circRNA_011541	18:14986350|15067619
circRNA_017221	7:53605879|53608643	circRNA_011018	1:154139505|154142929	circRNA_019153	7:33196756|33216752	circRNA_011817	5:50724811|50806131
circRNA_003197	13:119794850|119797615	circRNA_011117	10:54455236|54458661	circRNA_004230	4:15252446|15272443	circRNA_006159	4:90623646|90704987
circRNA_006141	4:43558505|43561270	circRNA_007217	15:19245136|19248562	circRNA_015658	14:114543856|114563854	circRNA_009649	6:74980742|75062084
circRNA_016199	6:18444401|18447166	circRNA_000074	1:96802617|96806044	circRNA_016904	15:85364480|85384479	circRNA_000864	14:142775864|142857217
circRNA_003191	13:104482994|104485760	circRNA_008928	14:78510020|78513447	circRNA_000632	13:108339930|108359939	circRNA_001605	4:90623622|90704987
circRNA_004517	6:7491143|7493909	circRNA_018761	14:124862600|124866027	circRNA_012159	X:70834204|70854213	circRNA_010704	5:53531603|53612985
circRNA_003081	13:26034153|26040794	circRNA_003478	14:146703126|146706554	circRNA_010164	1:3240473|3260484	circRNA_002487	1:28449591|28531105
circRNA_006834	10:49667206|49673847	circRNA_006716	1:175580337|175583766	circRNA_014964	1:224091690|224111707	circRNA_001983	7:65150234|65231776
circRNA_004383	4:136451155|136457797	circRNA_016811	13:108410122|108413554	circRNA_017309	9:9101193|9121211	circRNA_004589	6:82937671|83019237
circRNA_000899	15:34890619|34897264	circRNA_017784	3:22239814|22243246	circRNA_005428	1:86322010|86342031	circRNA_001833	6:82937667|83019237
circRNA_008717	13:19730728|19737377	circRNA_006267	6:69151420|69154853	circRNA_015099	6:132124985|132145007	circRNA_017398	X:86412505|86494087
circRNA_004697	6:147575304|147581958	circRNA_010821	8:10597524|10600957	circRNA_015763	16:47329782|47349804	circRNA_019400	1:225915881|225997487
circRNA_007588	3:104206529|104213184	circRNA_019617	2:12143766|12147199	circRNA_012109	GL893329.2:4247|24274	circRNA_020196	5:42104599|42186208
circRNA_010061	GL894396.1:17793|24448	circRNA_009576	5:79946906|79950340	circRNA_018363	9:8159257|8179287	circRNA_001694	5:23083310|23164933
circRNA_009556	5:63658260|63664917	circRNA_009712	6:148135805|148139239	circRNA_013576	X:45938682|45958714	circRNA_018929	2:91601752|91683387
circRNA_017369	GL894396.1:17793|24451	circRNA_015339	10:37120502|37123936	circRNA_013404	8:104061364|104081397	circRNA_017738	18:26093833|26175513
circRNA_019318	GL895152.1:20630|27290	circRNA_020493	10:11883981|11887415	circRNA_016248	6:101779796|101799831	circRNA_005918	17:24785296|24866991
circRNA_016204	6:38271273|38277935	circRNA_005020	8:141405909|141409344	circRNA_018882	18:42654420|42674455	circRNA_017022	2:78489911|78571606
circRNA_017833	4:71280364|71287026	circRNA_006253	6:18424722|18428157	circRNA_021053	5:35894045|35914080	circRNA_019741	5:89495676|89577371
circRNA_021020	4:73965115|73971777	circRNA_004169	3:109625600|109629036	circRNA_000110	1:128019978|128040020	circRNA_010608	3:25012488|25094251
circRNA_001863	6:99236964|99243627	circRNA_008521	10:33046923|33050359	circRNA_020290	8:75542191|75562233	circRNA_014402	3:97544470|97626276
circRNA_001844	6:88344356|88351020	circRNA_003227	13:147207247|147210684	circRNA_009503	4:134750965|134771008	circRNA_013595	X:126803212|126885048
circRNA_017881	6:48389581|48396245	circRNA_001655	4:129789461|129792899	circRNA_000652	13:131013234|131033279	circRNA_018180	15:85325487|85407334
circRNA_008635	12:11476585|11483250	circRNA_009093	16:34249808|34253246	circRNA_006118	3:128253444|128273499	circRNA_001836	6:82937679|83019536
circRNA_017817	4:8469596|8476262	circRNA_016130	5:17174541|17177982	circRNA_000697	13:190906188|190926244	circRNA_001835	6:82937671|83019536
circRNA_020745	15:85110060|85116726	circRNA_016334	8:413584|417026	circRNA_018603	12:39197540|39217604	circRNA_001834	6:82937667|83019536
circRNA_021246	9:19336952|19343618	circRNA_010237	1:227193880|227197323	circRNA_001900	6:128733782|128753848	circRNA_006018	2:93467543|93549427
circRNA_001034	16:22541545|22548212	circRNA_012485	12:37674719|37678162	circRNA_007258	15:85380889|85400955	circRNA_017147	5:74566418|74648385
circRNA_007292	15:142753686|142760356	circRNA_013104	4:73759207|73762650	circRNA_021007	4:38917845|38937913	circRNA_019772	6:138876634|138958631

circRNA_008764	13:67530900|67537576	circRNA_016076	4:68358494|68361938	circRNA_010866	9:21929295|21949364	circRNA_004456	5:70565540|70647555
circRNA_010040	GL892407.1:103229|109905	circRNA_013590	X:113600156|113603602	circRNA_010919	GL894982.1:12114|32184	circRNA_009109	16:51341325|51423416
circRNA_002675	1:205465515|205472192	circRNA_009005	15:53796806|53800256	circRNA_017767	2:98306721|98326796	circRNA_021172	7:91900007|91982115
circRNA_004442	5:50595002|50601679	circRNA_009212	2:49283|52733	circRNA_016354	8:43018351|43038428	circRNA_012211	1:49106605|49188719
circRNA_015313	1:297438493|297445171	circRNA_008316	1:48754989|48758440	circRNA_013953	8:76063302|76083381	circRNA_005570	10:66473871|66556005
circRNA_008551	10:54048970|54055651	circRNA_009063	15:142756903|142760356	circRNA_000557	13:33508401|33528483	circRNA_006396	8:39666147|39748311
circRNA_014567	9:116176878|116183562	circRNA_009623	6:26463430|26466884	circRNA_002708	1:249459092|249479177	circRNA_007236	15:59997322|60079510
circRNA_007610	3:122022811|122029496	circRNA_017126	4:118064156|118067610	circRNA_017111	4:55278022|55298110	circRNA_005500	1:229214112|229296319
circRNA_017895	6:85037207|85043893	circRNA_008400	1:176156294|176159751	circRNA_012464	12:13911452|13931543	circRNA_010753	6:101697609|101779902
circRNA_014010	15:101703689|101710376	circRNA_019001	3:107569717|107573174	circRNA_002822	10:47086473|47106567	circRNA_013477	9:101476729|101559108
circRNA_011691	3:72556230|72562918	circRNA_018868	18:18617125|18620583	circRNA_006086	3:84410703|84430797	circRNA_001922	6:147753296|147835689
circRNA_014414	4:37095316|37102004	circRNA_020711	14:131867981|131871439	circRNA_020452	1:208292449|208312544	circRNA_017999	9:88650139|88732539
circRNA_015637	14:77594174|77600868	circRNA_004769	7:57914632|57918091	circRNA_017911	6:137379532|137399634	circRNA_000469	12:26640168|26722750
circRNA_021139	6:147350407|147357101	circRNA_009674	6:98799538|98803000	circRNA_002931	11:56283465|56303570	circRNA_012558	13:83142279|83224873
circRNA_010527	17:43416619|43423315	circRNA_005774	14:33388048|33391511	circRNA_012803	16:37494597|37514704	circRNA_004791	7:67504695|67587294
circRNA_004301	4:79094980|79101679	circRNA_002009	7:91949364|91952828	circRNA_011544	18:16011861|16031971	circRNA_020315	9:37910304|37993012
circRNA_021347	JH118921.1:16802|23501	circRNA_013624	12:35282347|35285813	circRNA_021161	7:43147749|43167861	circRNA_014169	11:9798241|9881009
circRNA_015192	1:101209894|101216594	circRNA_013867	17:19831674|19835142	circRNA_000189	1:205472022|205492135	circRNA_004246	4:35980125|36062940
circRNA_019531	14:60129291|60135991	circRNA_002307	9:144090434|144093904	circRNA_002062	8:19189621|19209747	circRNA_005832	15:36264244|36347101
circRNA_018762	14:131861698|131868399	circRNA_003021	12:44352663|44356138	circRNA_010999	1:134770024|134790152	circRNA_000218	1:243617932|243700790
circRNA_011797	4:139822139|139828842	circRNA_007602	3:118692673|118696148	circRNA_001296	2:87312978|87333107	circRNA_004245	4:35980007|36062940
circRNA_000531	13:14168314|14175018	circRNA_013371	8:30554693|30558168	circRNA_021301	GL892680.2:158928|179057	circRNA_010320	11:86933456|87016420
circRNA_003783	17:45690868|45697572	circRNA_017323	9:40775540|40779015	circRNA_003983	2:98306664|98326796	circRNA_011665	3:24272672|24355652
circRNA_017306	9:6833607|6840311	circRNA_018915	2:76183437|76186912	circRNA_013329	7:65320364|65340497	circRNA_014107	1:86871594|86954593
circRNA_017648	14:118528151|118534855	circRNA_002089	8:58299514|58302990	circRNA_001312	2:98306659|98326796	circRNA_011118	10:56592594|56675627
circRNA_021375	X:90723658|90730364	circRNA_015342	10:37446481|37449957	circRNA_012823	16:78165123|78185260	circRNA_017515	10:18167052|18250156
circRNA_011144	11:55455868|55462575	circRNA_016212	6:56726193|56729671	circRNA_002746	1:280648787|280668925	circRNA_006155	4:88593557|88676666
circRNA_005444	1:132819046|132825754	circRNA_002381	GL896549.1:20035|23514	circRNA_011466	16:23558435|23578577	circRNA_016320	7:109768787|109851924
circRNA_009636	6:57237774|57244482	circRNA_013173	5:53299514|53302996	circRNA_015989	3:74752890|74773032	circRNA_010131	X:70426183|70509324
circRNA_010962	1:37509689|37516397	circRNA_010083	GL896223.1:1634|5118	circRNA_013722	6:39320647|39340790	circRNA_000631	13:106138563|106221705
circRNA_020868	2:47536181|47542889	circRNA_008421	1:200269189|200272677	circRNA_012942	2:82157336|82177480	circRNA_011153	11:76391099|76474363
circRNA_012391	10:61737813|61744522	circRNA_015433	12:21825027|21828516	circRNA_012916	2:32306523|32326668	circRNA_016494	9:115608962|115692308
circRNA_000772	14:44169277|44175988	circRNA_015980	3:61070254|61073743	circRNA_020464	1:247785597|247805744	circRNA_005839	15:65858191|65941541
circRNA_002917	11:49487460|49494171	circRNA_014323	16:70013311|70016802	circRNA_003994	2:118941644|118961796	circRNA_004642	6:103828536|103911899
circRNA_000511	12:53721335|53728048	circRNA_006758	1:242084960|242088453	circRNA_011793	4:129522757|129542909	circRNA_005878	15:137963814|138047261
circRNA_006681	1:134520214|134526927	circRNA_009419	3:133942984|133946477	circRNA_005297	JH118940.1:55268|75425	circRNA_001934	6:153309526|153393008
circRNA_006659	1:101704036|101710750	circRNA_006281	6:85419249|85422743	circRNA_019084	5:92498438|92518596	circRNA_019863	GL892822.1:1893|85405
circRNA_002898	11:20075966|20082683	circRNA_013494	9:125452979|125456473	circRNA_012052	9:25908017|25928177	circRNA_006508	9:129821401|129904943
circRNA_012194	1:30921618|30928335	circRNA_005087	9:29693288|29696783	circRNA_016871	14:114007796|114027959	circRNA_017681	15:105424453|105508062
circRNA_013100	4:68353630|68360347	circRNA_010357	13:26518542|26522037	circRNA_002034	7:116973936|116994106	circRNA_009994	9:96806488|96890119
circRNA_000532	13:14168314|14175033	circRNA_012304	1:231466521|231470019	circRNA_008108	9:42973775|42993947	circRNA_010410	13:170308586|170392219
circRNA_014829	3:74760760|74767479	circRNA_003727	16:55481570|55485070	circRNA_013481	9:106476062|106496241	circRNA_010632	3:103476557|103560207
circRNA_017936	7:73254173|73260894	circRNA_020314	9:37686040|37689540	circRNA_016058	4:39622405|39642587	circRNA_020169	4:39859616|39943326
circRNA_000269	1:306165050|306171772	circRNA_003593	15:105424453|105427954	circRNA_019395	1:208295276|208315461	circRNA_010488	15:98126307|98210091
circRNA_012642	14:51693016|51699738	circRNA_013777	1:181161592|181165093	circRNA_003714	16:47936074|47956261	circRNA_000975	15:98126307|98210102
circRNA_011369	14:120762011|120768734	circRNA_017701	16:25402510|25406011	circRNA_019140	6:150870199|150890389	circRNA_016147	5:50654972|50738780
circRNA_012548	13:72821916|72828640	circRNA_018772	15:3788416|3791918	circRNA_001347	2:133350705|133370908	circRNA_001394	3:24272672|24356481
circRNA_020631	13:140914401|140921126	circRNA_019831	8:120592400|120595902	circRNA_020729	15:45574567|45594770	circRNA_013912	4:48056407|48140232
circRNA_011729	3:114487512|114494241	circRNA_005724	13:142937807|142941310	circRNA_004124	3:84254791|84274997	circRNA_003371	14:70841885|70925732
circRNA_004335	4:107957216|107963947	circRNA_020799	16:79273754|79277257	circRNA_017135	5:16572550|16592759	circRNA_000334	10:46597762|46681760
circRNA_018008	9:138542044|138548775	circRNA_010822	8:11361730|11365234	circRNA_017540	11:12807920|12828134	circRNA_010675	4:85304081|85388111
circRNA_000842	14:115712680|115719413	circRNA_015501	13:42355650|42359154	circRNA_014038	1:202793118|202813336	circRNA_011948	7:61558755|61642845

circRNA_001069	16:51757091|51763824	circRNA_007172	14:115645035|115648541	circRNA_007512	2:132227980|132248199	circRNA_000331	10:46597662|46681760
circRNA_019512	13:210830263|210836997	circRNA_007953	7:103749665|103753171	circRNA_020070	16:3507681|3527903	circRNA_011451	15:145529864|145614211
circRNA_011241	13:90946479|90953214	circRNA_005343	X:70495253|70498760	circRNA_002268	9:101231884|101252107	circRNA_007889	6:149027711|149112188
circRNA_011633	2:128435275|128442010	circRNA_010631	3:102842916|102846423	circRNA_015609	14:49983998|50004223	circRNA_020370	1:30747601|30832135
circRNA_017705	16:27973793|27980528	circRNA_010857	8:144326770|144330278	circRNA_020142	3:62785202|62805429	circRNA_011788	4:110330609|110415274
circRNA_002407	X:3677199|3683935	circRNA_005036	9:24239|27748	circRNA_004954	8:80328399|80348627	circRNA_019513	13:211155143|211239824
circRNA_014504	7:91949364|91956101	circRNA_006886	11:26301535|26305044	circRNA_007306	16:27072071|27092307	circRNA_006744	1:210044043|210128769
circRNA_001445	3:75950908|75957646	circRNA_009969	9:50907011|50910521	circRNA_015536	13:102283761|102304005	circRNA_007515	2:140676197|140760940
circRNA_008975	14:145324412|145331151	circRNA_014384	3:26002107|26005617	circRNA_009412	3:122035667|122055914	circRNA_011389	14:152764272|152849049
circRNA_000159	1:180708440|180715180	circRNA_006887	11:26301535|26305046	circRNA_006178	4:117897403|117917659	circRNA_017570	12:38779681|38864471
circRNA_007228	15:51629686|51636427	circRNA_012605	13:205285357|205288868	circRNA_010261	10:16947860|16968117	circRNA_010676	4:85304081|85388962
circRNA_020298	8:135799269|135806012	circRNA_005722	13:142834061|142837573	circRNA_014312	16:42288019|42308283	circRNA_020918	2:137521318|137606246
circRNA_010867	9:25669692|25676436	circRNA_017136	5:19125001|19128513	circRNA_002385	JH118611.1:2203|22469	circRNA_014254	14:66692910|66777839
circRNA_016098	4:110134177|110140922	circRNA_014638	13:81371686|81375200	circRNA_018741	14:71887546|71907827	circRNA_005123	9:62261845|62346821
circRNA_017011	2:30457263|30464010	circRNA_007904	7:29348907|29352423	circRNA_007066	13:145224128|145244412	circRNA_002261	9:85462848|85547840
circRNA_002622	1:159899221|159905970	circRNA_016223	6:73663166|73666682	circRNA_015102	6:146042987|146063275	circRNA_009825	8:20657917|20742913
circRNA_021010	4:41305796|41312545	circRNA_010992	1:132344010|132347527	circRNA_009509	4:139682020|139702320	circRNA_010141	X:86132408|86217418
circRNA_007305	16:25730174|25736924	circRNA_000481	12:37538488|37542007	circRNA_016709	1:311023437|311043740	circRNA_017566	12:35849683|35934746
circRNA_010139	X:70994646|71001396	circRNA_000744	14:16177975|16181494	circRNA_008579	11:7424395|7444699	circRNA_012599	13:168102462|168187615
circRNA_016510	GL892486.1:4394|11147	circRNA_007225	15:37958867|37962386	circRNA_004322	4:98051660|98071965	circRNA_008092	9:25256510|25341666
circRNA_014810	18:2773545|2780302	circRNA_009137	17:28264912|28268431	circRNA_012439	11:52224093|52244398	circRNA_009717	6:150809988|150895226
circRNA_009293	2:121555001|121561759	circRNA_014047	15:130331479|130335001	circRNA_018244	2:154284494|154304800	circRNA_001582	4:55318260|55403504
circRNA_014509	7:103142949|103149709	circRNA_015451	12:40720302|40723824	circRNA_001819	6:69433065|69453376	circRNA_000430	11:75498858|75584138
circRNA_009644	6:69159389|69166153	circRNA_014633	12:53724525|53728048	circRNA_002202	9:22526568|22546881	circRNA_012442	11:53900017|53985349
circRNA_010322	12:12797259|12804024	circRNA_013547	GL896250.1:12657|16183	circRNA_005797	14:104690448|104710775	circRNA_006730	1:195029560|195115065
circRNA_009032	15:86615927|86622694	circRNA_014574	9:150280681|150284207	circRNA_016141	5:38561440|38581769	circRNA_001551	4:17146601|17232117
circRNA_015179	1:84364418|84371187	circRNA_008815	13:131630312|131633839	circRNA_011637	2:140494862|140515197	circRNA_019907	1:45652265|45737883
circRNA_019554	14:132251502|132258271	circRNA_006362	7:103746214|103749742	circRNA_014189	13:20785157|20805497	circRNA_015115	10:46596019|46681760
circRNA_019946	1:271369643|271376413	circRNA_013987	1:231595599|231599127	circRNA_014771	10:37797097|37817442	circRNA_005897	16:47362889|47448671
circRNA_002975	12:16861242|16868014	circRNA_012120	GL894386.1:51262|54793	circRNA_001962	7:38858509|38878866	circRNA_012734	15:85397016|85482828
circRNA_002922	11:52436220|52442994	circRNA_005216	GL892447.1:26353|29885	circRNA_008716	13:18323062|18343422	circRNA_013029	3:84302257|84388089
circRNA_000178	1:202612264|202619041	circRNA_010905	9:139615762|139619294	circRNA_012922	2:50783412|50803783	circRNA_020155	3:121371409|121457355
circRNA_004037	2:147428027|147434804	circRNA_017177	6:85225143|85228676	circRNA_014310	16:24768066|24788444	circRNA_016797	13:73450817|73536800
circRNA_006300	6:125923718|125930495	circRNA_012246	1:124973704|124977239	circRNA_015761	16:42621232|42641610	circRNA_020197	5:42104599|42190657
circRNA_011938	7:40829298|40835755	circRNA_015283	1:244830879|244834415	circRNA_008277	X:122527958|122548340	circRNA_017260	8:31861798|31947867
circRNA_011315	14:30066845|30073303	circRNA_003261	13:209949211|209952749	circRNA_009191	18:36452161|36472543	circRNA_004163	3:107521846|107608026
circRNA_007494	2:97474834|97481294	circRNA_010401	13:150611009|150614548	circRNA_019341	X:52869732|52890114	circRNA_020187	4:134296416|134382599
circRNA_007998	8:34750879|34757340	circRNA_002473	1:18659951|18663491	circRNA_010213	1:161745226|161765610	circRNA_008515	10:18101700|18187909
circRNA_010965	1:44462994|44469456	circRNA_016652	1:141822796|141826336	circRNA_001094	17:24573209|24593597	circRNA_015164	1:45701230|45787500
circRNA_011370	14:120786139|120792605	circRNA_011659	3:14916894|14920435	circRNA_017774	2:142522073|142542463	circRNA_002958	12:14242194|14328470
circRNA_010051	GL893329.2:13562|20029	circRNA_003926	2:71199117|71202659	circRNA_008957	14:130772962|130793353	circRNA_011709	3:92382600|92468880
circRNA_009817	7:134434018|134440490	circRNA_000403	11:22240397|22243942	circRNA_003237	13:163011611|163032003	circRNA_020394	1:89620463|89706800
circRNA_000814	14:81355508|81361983	circRNA_006735	1:201668388|201671933	circRNA_001043	16:25440380|25460777	circRNA_014664	15:91537307|91623705
circRNA_016427	9:9311496|9317971	circRNA_016872	14:114509960|114513508	circRNA_006046	2:142522062|142542463	circRNA_001156	18:14523462|14609873
circRNA_018888	18:44790011|44796486	circRNA_016163	5:78609792|78613341	circRNA_020289	8:70949394|70969796	circRNA_016375	8:72694531|72781012
circRNA_018984	3:76334887|76341362	circRNA_010860	9:7105929|7109479	circRNA_004268	4:45173708|45194119	circRNA_007706	4:141995014|142081537
circRNA_014747	9:139983624|139990100	circRNA_012133	GL896294.1:101912|105462	circRNA_017737	18:20655219|20675630	circRNA_006187	4:141995014|142081554
circRNA_020859	2:11825781|11832258	circRNA_011860	6:11749560|11753113	circRNA_002542	1:94575046|94595460	circRNA_000600	13:73561709|73648259
circRNA_004098	3:63025940|63032418	circRNA_000506	12:51844701|51848255	circRNA_003944	2:83895486|83915903	circRNA_013472	9:85222175|85308751
circRNA_015246	1:205474830|205481309	circRNA_014818	2:89865397|89868951	circRNA_012490	12:40331141|40351560	circRNA_018312	6:82942371|83028967
circRNA_005053	9:9541816|9548296	circRNA_000419	11:53854619|53858174	circRNA_018981	3:74752613|74773032	circRNA_012312	1:255662209|255748862
circRNA_018600	12:37146039|37152519	circRNA_018906	2:49898278|49901833	circRNA_012285	1:188159439|188179860	circRNA_011911	6:142972882|143059562

circRNA_009304	2:139112346|139118827	circRNA_003858	18:44787541|44791097	circRNA_017641	14:82666033|82686458	circRNA_011658	3:12559589|12646295
circRNA_011848	5:99117971|99124452	circRNA_011615	2:91169756|91173312	circRNA_009115	16:54837291|54857726	circRNA_017426	1:38708636|38795389
circRNA_000800	14:71918903|71925385	circRNA_011293	13:209969995|209973552	circRNA_012309	1:247961314|247981752	circRNA_012963	2:118253397|118340199
circRNA_006305	6:135412453|135418935	circRNA_007085	13:209966625|209970183	circRNA_009149	17:43061013|43081454	circRNA_011851	5:105210066|105296870
circRNA_012909	2:29213178|29219660	circRNA_003631	15:122866809|122870369	circRNA_017197	6:121974470|121994919	circRNA_012429	11:19071828|19158675
circRNA_014419	4:51210255|51216737	circRNA_001267	2:70868417|70871979	circRNA_012698	14:138392575|138413025	circRNA_002115	8:88098100|88185045
circRNA_000574	13:42295832|42302315	circRNA_010162	X:140034602|140038164	circRNA_004534	6:18424722|18445181	circRNA_019992	13:32775505|32862490
circRNA_017363	GL893671.2:10789|17272	circRNA_005019	8:141374276|141377839	circRNA_016087	4:90623622|90644081	circRNA_001984	7:65150234|65237271
circRNA_017905	6:124821661|124828144	circRNA_010288	10:54728782|54732345	circRNA_019576	15:115994299|116014762	circRNA_001284	2:82549217|82636266
circRNA_007619	3:135157659|135164143	circRNA_011001	1:134796959|134800523	circRNA_001208	2:3009236|3029700	circRNA_020574	13:18089043|18176107
circRNA_005586	11:18598960|18605445	circRNA_008413	1:182110736|182114301	circRNA_012048	9:13714998|13735468	circRNA_007773	5:87371066|87458131
circRNA_014952	9:137088972|137095457	circRNA_008708	13:4770593|4774160	circRNA_013569	X:29493890|29514363	circRNA_015587	14:418144|505329
circRNA_015447	12:37419007|37425492	circRNA_003654	15:155094318|155097886	circRNA_015927	2:132414983|132435460	circRNA_010686	4:136071929|136159147
circRNA_016799	13:75857804|75864289	circRNA_016881	14:136406123|136409692	circRNA_014241	14:42332310|42352788	circRNA_000255	1:281586888|281674110
circRNA_017374	GL895092.1:7358|13844	circRNA_014474	6:85037207|85040777	circRNA_005510	1:242716644|242737129	circRNA_015291	1:256867612|256954834
circRNA_004061	3:17185508|17191996	circRNA_004786	7:65162015|65165588	circRNA_016875	14:120758678|120779168	circRNA_021213	8:87952704|88039934
circRNA_003073	13:19755935|19762426	circRNA_017530	10:56117038|56120611	circRNA_014518	8:20672958|20693450	circRNA_008042	8:108342978|108430220
circRNA_021069	5:87376748|87383239	circRNA_018789	15:85087751|85091324	circRNA_014259	14:98626249|98646746	circRNA_018556	10:62803459|62890750
circRNA_010673	4:73743510|73750002	circRNA_006268	6:69231033|69234607	circRNA_017236	7:97192526|97213027	circRNA_011353	14:108151041|108238352
circRNA_006664	1:105944306|105950799	circRNA_006752	1:225691245|225694820	circRNA_007596	3:109890287|109910797	circRNA_014931	3:107521846|107609161
circRNA_013552	JH118602.1:45202|51695	circRNA_009360	3:56414602|56418177	circRNA_004956	8:80764338|80784852	circRNA_007599	3:113747715|113835066
circRNA_021322	GL894772.1:8659|15154	circRNA_003330	14:32142333|32145909	circRNA_003650	15:147175763|147196279	circRNA_014283	15:73203467|73290946
circRNA_018038	GL896531.2:31501|37998	circRNA_008597	11:22240397|22243973	circRNA_015687	15:36536596|36557118	circRNA_016273	7:16923592|17011103
circRNA_018848	17:37032362|37038862	circRNA_010625	3:82691426|82695002	circRNA_015135	X:126967224|126987749	circRNA_004516	6:3409239|3496769
circRNA_000927	15:67042882|67049383	circRNA_014043	13:142931343|142934920	circRNA_015934	2:140514638|140535165	circRNA_005580	11:11662613|11750175
circRNA_001602	4:90453173|90459676	circRNA_005922	17:37108292|37111872	circRNA_011395	15:36377634|36398162	circRNA_020372	1:30921618|31009204
circRNA_005980	2:52847733|52854237	circRNA_014290	15:85065639|85069219	circRNA_000127	1:136271279|136291811	circRNA_019498	13:150156559|150244152
circRNA_002511	1:62569661|62576166	circRNA_003422	14:114875209|114878790	circRNA_021158	7:33165424|33185960	circRNA_016493	9:113369836|113457461
circRNA_019247	9:13613834|13620342	circRNA_013204	5:94469233|94472814	circRNA_021038	4:137128127|137148667	circRNA_012325	1:293212383|293300073
circRNA_003096	13:33961610|33968119	circRNA_015280	1:241244047|241247628	circRNA_017690	15:131253281|131273823	circRNA_007867	6:128742430|128830137
circRNA_004341	4:116008617|116015126	circRNA_003868	18:57746684|57750266	circRNA_000486	12:39209207|39229750	circRNA_012516	13:32774767|32862490
circRNA_019727	5:47577974|47584483	circRNA_017836	4:87318496|87322078	circRNA_017484	1:202650920|202671463	circRNA_018228	2:78483819|78571606
circRNA_000886	15:19245136|19251646	circRNA_020874	2:52346942|52350524	circRNA_020322	9:57693529|57714073	circRNA_009511	4:141816454|141904247
circRNA_012545	13:55748558|55755068	circRNA_001483	3:106723747|106727330	circRNA_015903	2:98306246|98326796	circRNA_006412	8:78332378|78420195
circRNA_010052	GL893425.2:35498|42010	circRNA_019537	14:77586163|77589748	circRNA_011347	14:82393345|82413898	circRNA_013814	13:50931553|51019448
circRNA_003410	14:95245306|95251819	circRNA_002953	12:5476779|5480365	circRNA_006598	1:4687898|4708453	circRNA_006593	1:2150622|2238552
circRNA_007741	5:42911441|42917954	circRNA_014550	9:46280538|46284126	circRNA_019358	1:18451548|18472105	circRNA_001539	3:137511691|137599665
circRNA_016929	16:23487328|23493841	circRNA_015445	12:36634508|36638096	circRNA_019352	X:116341278|116361851	circRNA_019630	2:83782392|83870489
circRNA_017704	16:27105469|27111983	circRNA_008486	1:282274683|282278272	circRNA_017016	2:51311848|51332424	circRNA_001485	3:107521846|107609954
circRNA_003325	14:31377807|31384322	circRNA_014588	GL896504.1:27999|31588	circRNA_008603	11:25528481|25549061	circRNA_015810	18:9534503|9622620
circRNA_002635	1:182114095|182120613	circRNA_000162	1:181051819|181055410	circRNA_019178	7:91938950|91959537	circRNA_002742	1:280567722|280656034
circRNA_004424	5:35948460|35954978	circRNA_012892	18:59916697|59920288	circRNA_006870	11:19712163|19732756	circRNA_015649	14:92407605|92495988
circRNA_007380	18:6253741|6260259	circRNA_007920	7:63538957|63542549	circRNA_000775	14:52810347|52830945	circRNA_008237	X:14581992|14670417
circRNA_012030	8:144223745|144230263	circRNA_019164	7:62801435|62805027	circRNA_014530	8:89346255|89366855	circRNA_001659	4:134294064|134382599
circRNA_000300	10:27281192|27287711	circRNA_019983	12:37179801|37183393	circRNA_001000	15:117892875|117913489	circRNA_017281	8:78332378|78420954
circRNA_004746	7:33109626|33116145	circRNA_016534	GL894729.2:8549|12142	circRNA_004500	5:94418719|94439340	circRNA_020412	1:136203228|136291811
circRNA_009947	9:40797941|40804462	circRNA_000391	11:16104484|16108078	circRNA_013483	9:114841276|114861897	circRNA_013391	8:78332378|78420991
circRNA_013637	4:129731405|129737926	circRNA_003963	2:88675453|88679052	circRNA_010738	6:41162846|41183469	circRNA_010662	4:41114627|41203285
circRNA_000176	1:202605803|202612325	circRNA_012592	13:156206347|156209946	circRNA_008305	1:30921618|30942245	circRNA_018725	14:49924111|50012794
circRNA_010983	1:101861737|101868259	circRNA_007790	6:16262816|16266416	circRNA_015616	14:58512296|58532933	circRNA_013108	4:83597540|83686353
circRNA_016227	6:85042122|85048644	circRNA_009213	2:67323|70923	circRNA_016788	13:42295832|42316470	circRNA_013693	15:12078417|12167330
circRNA_003037	12:48232839|48239362	circRNA_007203	14:145335356|145338958	circRNA_005108	9:43030233|43050877	circRNA_018147	14:7193887|7282852

circRNA_003419	14:110126707|110133230	circRNA_009506	4:136816726|136820329	circRNA_012071	9:78724734|78745378	circRNA_005055	9:11302178|11391169
circRNA_015479	13:20950127|20956650	circRNA_013540	GL895238.2:35727|39331	circRNA_016603	X:109076556|109097201	circRNA_012920	2:48754061|48843100
circRNA_013965	9:101245582|101252107	circRNA_014041	12:52267683|52271288	circRNA_018829	16:38265568|38286217	circRNA_011104	10:37747971|37837047
circRNA_020592	13:55562358|55568883	circRNA_006028	2:112631869|112635475	circRNA_020838	18:31047048|31067701	circRNA_001486	3:107521846|107610973
circRNA_007510	2:121377282|121383808	circRNA_009235	2:43651009|43654615	circRNA_008426	1:200644823|200665488	circRNA_004164	3:107521846|107611131
circRNA_008677	12:44786749|44793275	circRNA_012958	2:98554166|98557773	circRNA_010651	4:19761756|19782422	circRNA_011372	14:123534286|123623664
circRNA_017055	2:148217982|148224509	circRNA_003214	13:143421891|143425499	circRNA_012487	12:37716002|37736668	circRNA_001874	6:101724150|101813590
circRNA_019320	GL896223.1:1634|8162	circRNA_000786	14:59598742|59602351	circRNA_017973	8:141444429|141465095	circRNA_007075	13:166056959|166146411
circRNA_008800	13:102338729|102345263	circRNA_011876	6:65200370|65203979	circRNA_019534	14:70775779|70796446	circRNA_009076	16:9120620|9210113
circRNA_010837	8:71582612|71589148	circRNA_009516	5:4783980|4787591	circRNA_003370	14:70775779|70796449	circRNA_021241	9:8237835|8327340
circRNA_016490	9:101486703|101493242	circRNA_005996	2:82197529|82201141	circRNA_002707	1:245505757|245526429	circRNA_008244	X:30820948|30910575
circRNA_017708	16:34270329|34276870	circRNA_008968	14:140515705|140519318	circRNA_007152	14:77678428|77699105	circRNA_002362	GL895308.2:109700|199336
circRNA_012535	13:44026155|44032697	circRNA_017479	1:195026047|195029660	circRNA_004429	5:37153142|37173822	circRNA_016541	GL895308.2:109697|199336
circRNA_003167	13:90496250|90502795	circRNA_007424	2:15930326|15933940	circRNA_016409	8:141514558|141535238	circRNA_013145	5:3372611|3462302
circRNA_008374	1:134611516|134618061	circRNA_007467	2:80452934|80456550	circRNA_011202	13:24111612|24132299	circRNA_017525	10:50877061|50966766
circRNA_011242	13:94325217|94331764	circRNA_009243	2:50329858|50333474	circRNA_021131	6:125923718|125944413	circRNA_013948	8:20657917|20747741
circRNA_016330	7:128607180|128613730	circRNA_014894	GL894386.1:44564|48180	circRNA_003432	14:117630383|117651080	circRNA_002543	1:100401141|100491014
circRNA_003467	14:141331113|141337664	circRNA_007708	4:143285894|143289512	circRNA_006898	11:70156594|70177292	circRNA_002501	1:57105762|57195641
circRNA_016188	6:9579141|9585692	circRNA_011106	10:44203044|44206662	circRNA_008216	GL895829.1:12507|33213	circRNA_020871	2:50895879|50985835
circRNA_013938	6:145402515|145409067	circRNA_011514	17:36470199|36473817	circRNA_019210	8:49959084|49979796	circRNA_001854	6:92373542|92463528
circRNA_000568	13:35652295|35658848	circRNA_016550	GL896292.1:8833|12451	circRNA_004373	4:132825827|132846542	circRNA_002486	1:28422430|28512553
circRNA_010666	4:55318260|55324815	circRNA_002557	1:117494797|117498416	circRNA_013289	6:148116282|148136997	circRNA_003360	14:59632979|59723112
circRNA_009141	17:36148045|36154601	circRNA_004853	7:120243451|120247070	circRNA_014439	4:142854258|142874981	circRNA_017185	6:91829575|91919964
circRNA_009221	2:16112934|16119490	circRNA_008895	14:37703812|37707431	circRNA_004576	6:78476142|78496868	circRNA_017184	6:91829524|91919964
circRNA_013450	9:40808237|40814793	circRNA_005263	GL895152.1:23036|26657	circRNA_006310	6:148034940|148055667	circRNA_001513	3:120260142|120350634
circRNA_009308	2:140765849|140772407	circRNA_013838	14:66858975|66862596	circRNA_003723	16:54816613|54837341	circRNA_016027	3:117370067|117460581
circRNA_000153	1:157816758|157823318	circRNA_016170	5:86850247|86853868	circRNA_005746	13:210819581|210840309	circRNA_004569	6:73446116|73536700
circRNA_003860	18:47708686|47715249	circRNA_011612	2:88742854|88746479	circRNA_008238	X:15937583|15958311	circRNA_013895	3:28871129|28961725
circRNA_002809	10:37117371|37123936	circRNA_012799	16:34014482|34018107	circRNA_000816	14:81960481|81981214	circRNA_001668	5:126533|217179
circRNA_014892	9:138768104|138774670	circRNA_018333	7:48164559|48168184	circRNA_007675	4:98044696|98065430	circRNA_003092	13:32774767|32865436
circRNA_015430	12:21025298|21031864	circRNA_012541	13:51703978|51707605	circRNA_001341	2:128479853|128500588	circRNA_010268	10:18033408|18124093
circRNA_001949	7:21583140|21589708	circRNA_019957	10:16955933|16959560	circRNA_019282	9:108475048|108495784	circRNA_006947	12:45421101|45511876
circRNA_009873	8:103131974|103138543	circRNA_003913	2:50800152|50803783	circRNA_006364	7:105387843|105408581	circRNA_018438	1:126859245|126950090
circRNA_011443	15:122266857|122273427	circRNA_001630	4:110972981|110976614	circRNA_015621	14:60763680|60784419	circRNA_007498	2:104272233|104363086
circRNA_014997	14:132237197|132243767	circRNA_012678	14:114534280|114537913	circRNA_020601	13:82682289|82703029	circRNA_018528	1:298098190|298189181
circRNA_014243	14:43482274|43488845	circRNA_015820	18:14554016|14557650	circRNA_018902	2:43643416|43664163	circRNA_003516	15:53022089|53113201
circRNA_017162	6:25505838|25512409	circRNA_020605	13:87137176|87140810	circRNA_001763	5:92502063|92522814	circRNA_009930	9:25250548|25341666
circRNA_012899	2:4164369|4170941	circRNA_018017	GL892421.2:74028|77664	circRNA_013379	8:40108767|40129519	circRNA_020839	18:36461492|36552661
circRNA_021163	7:57954480|57961055	circRNA_007896	7:5801349|5804986	circRNA_001477	3:102273355|102294111	circRNA_001012	15:124628985|124720160
circRNA_010718	5:87157448|87164024	circRNA_008067	8:144596236|144599873	circRNA_010656	4:39272528|39293284	circRNA_004393	4:142701348|142792594
circRNA_009246	2:50799143|50805720	circRNA_020159	3:135247772|135251409	circRNA_002112	8:83452129|83472898	circRNA_012443	11:53900017|53991328
circRNA_017258	8:31471156|31477735	circRNA_018956	2:157390156|157393794	circRNA_002175	9:7122453|7143222	circRNA_000467	12:23985199|24076622
circRNA_003806	18:6042153|6048736	circRNA_011354	14:110126707|110130346	circRNA_019233	8:130625685|130646459	circRNA_016137	5:32968798|33060293
circRNA_005621	12:23750110|23756693	circRNA_013336	7:80329454|80333094	circRNA_009616	6:23732487|23753262	circRNA_012472	12:16978073|17069607
circRNA_006320	7:4590605|4597189	circRNA_007370	17:57295480|57299121	circRNA_017387	X:28987140|29007918	circRNA_011616	2:92063632|92155200
circRNA_004115	3:81212631|81219219	circRNA_011459	15:157059180|157062821	circRNA_003234	13:155644173|155664955	circRNA_013631	16:4004000|4095662
circRNA_008215	GL895413.1:36903|43491	circRNA_014207	13:90759890|90763531	circRNA_017516	10:25684845|25705628	circRNA_021148	7:16831959|16923676
circRNA_016388	8:103131974|103138562	circRNA_011232	13:79322864|79326506	circRNA_015960	3:29618701|29639487	circRNA_017214	7:52748|144469
circRNA_014529	8:86035739|86042328	circRNA_000583	13:43406481|43410124	circRNA_008330	1:80327077|80347867	circRNA_004727	7:16831954|16923676
circRNA_012130	GL895729.1:902|7492	circRNA_006863	11:9504246|9507889	circRNA_020410	1:134656768|134677558	circRNA_016272	7:16831904|16923676
circRNA_019333	JH118993.1:13905|20498	circRNA_020323	9:58367332|58370975	circRNA_021264	9:49350469|49371259	circRNA_015165	1:45713269|45805070
circRNA_010734	6:33100577|33107171	circRNA_006569	X:64683360|64687004	circRNA_001714	5:49566730|49587521	circRNA_016807	13:102212196|102304005

<table>
<tr><td>circRNA_020985</td><td>3:114501069|114507663</td><td>circRNA_000551</td><td>13:29358370|29362016</td><td>circRNA_001849</td><td>6:92090650|92111445</td><td>circRNA_017478</td><td>1:195020817|195112631</td></tr>
<tr><td>circRNA_010179</td><td>1:59726631|59733226</td><td>circRNA_000795</td><td>14:65796621|65800267</td><td>circRNA_014462</td><td>5:93485749|93506549</td><td>circRNA_018748</td><td>14:93524865|93616690</td></tr>
<tr><td>circRNA_016467</td><td>9:57688516|57695111</td><td>circRNA_011374</td><td>14:131878361|131882008</td><td>circRNA_019108</td><td>6:85027838|85048644</td><td>circRNA_007463</td><td>2:78479725|78571606</td></tr>
<tr><td>circRNA_015366</td><td>11:320232|326828</td><td>circRNA_020805</td><td>17:12820325|12823972</td><td>circRNA_015144</td><td>1:17224292|17245110</td><td>circRNA_016689</td><td>1:250436030|250527929</td></tr>
<tr><td>circRNA_019530</td><td>14:60101240|60107836</td><td>circRNA_013083</td><td>4:39272528|39276176</td><td>circRNA_007145</td><td>14:68023837|68044656</td><td>circRNA_000015</td><td>1:16779138|16871069</td></tr>
<tr><td>circRNA_016031</td><td>3:121951940|121958538</td><td>circRNA_001175</td><td>18:25506902|25510551</td><td>circRNA_015988</td><td>3:74743268|74764088</td><td>circRNA_001302</td><td>2:89904144|89996112</td></tr>
<tr><td>circRNA_017875</td><td>6:10339611|10346209</td><td>circRNA_007254</td><td>15:85042800|85046450</td><td>circRNA_009598</td><td>5:99041477|99062303</td><td>circRNA_000642</td><td>13:117695880|117787888</td></tr>
<tr><td>circRNA_019126</td><td>6:127534127|127540725</td><td>circRNA_011900</td><td>6:113246297|113249947</td><td>circRNA_014059</td><td>9:96032630|96053461</td><td>circRNA_020205</td><td>5:98949655|99041729</td></tr>
<tr><td>circRNA_014032</td><td>GL896250.1:9584|16183</td><td>circRNA_012333</td><td>1:306809216|306812867</td><td>circRNA_005346</td><td>X:70833378|70854213</td><td>circRNA_020402</td><td>1:113957632|114049837</td></tr>
<tr><td>circRNA_005699</td><td>13:94225019|94231619</td><td>circRNA_017176</td><td>6:78576591|78580242</td><td>circRNA_020162</td><td>4:30318040|30338888</td><td>circRNA_004733</td><td>7:24658976|24751226</td></tr>
<tr><td>circRNA_015160</td><td>1:40782839|40789439</td><td>circRNA_018338</td><td>7:99573866|99577517</td><td>circRNA_000613</td><td>13:87119945|87140810</td><td>circRNA_016846</td><td>14:57689330|57781734</td></tr>
<tr><td>circRNA_004181</td><td>3:114079349|114085951</td><td>circRNA_001709</td><td>5:45994543|45998196</td><td>circRNA_001072</td><td>16:55475544|55496410</td><td>circRNA_002643</td><td>1:195022523|195115065</td></tr>
<tr><td>circRNA_009264</td><td>2:84231850|84238452</td><td>circRNA_019267</td><td>9:50892209|50895862</td><td>circRNA_017212</td><td>6:157482052|157502922</td><td>circRNA_016002</td><td>3:84723879|84816461</td></tr>
<tr><td>circRNA_015866</td><td>2:17216582|17223184</td><td>circRNA_005632</td><td>12:46120613|46124267</td><td>circRNA_017718</td><td>17:9733262|9754135</td><td>circRNA_012448</td><td>11:75001213|75093907</td></tr>
<tr><td>circRNA_007008</td><td>13:81194156|81200759</td><td>circRNA_010780</td><td>7:4863419|4867074</td><td>circRNA_015595</td><td>14:17053787|17074664</td><td>circRNA_020115</td><td>2:30057825|30150564</td></tr>
<tr><td>circRNA_017321</td><td>9:39192459|39199062</td><td>circRNA_012241</td><td>1:119472303|119475959</td><td>circRNA_003509</td><td>15:36556955|36577833</td><td>circRNA_000674</td><td>13:146205337|146298143</td></tr>
<tr><td>circRNA_000636</td><td>13:117331417|117338022</td><td>circRNA_014527</td><td>8:71508448|71512104</td><td>circRNA_016450</td><td>9:40133812|40154700</td><td>circRNA_020153</td><td>3:117367743|117460581</td></tr>
<tr><td>circRNA_011458</td><td>15:157056216|157062821</td><td>circRNA_004296</td><td>4:73714673|73718330</td><td>circRNA_009952</td><td>9:42973775|42994664</td><td>circRNA_017686</td><td>15:114075376|114168230</td></tr>
<tr><td>circRNA_016382</td><td>8:85974968|85981573</td><td>circRNA_019990</td><td>13:25072824|25076481</td><td>circRNA_002114</td><td>8:88019043|88039934</td><td>circRNA_018272</td><td>4:110314109|110406982</td></tr>
<tr><td>circRNA_010469</td><td>15:28834560|28841166</td><td>circRNA_003514</td><td>15:51647084|51650743</td><td>circRNA_001187</td><td>18:43942640|43963532</td><td>circRNA_018406</td><td>1:21156208|21249088</td></tr>
<tr><td>circRNA_008909</td><td>14:59110245|59116853</td><td>circRNA_003616</td><td>15:117371369|117375028</td><td>circRNA_020428</td><td>1:179091950|179112850</td><td>circRNA_007444</td><td>2:52823538|52916460</td></tr>
<tr><td>circRNA_010730</td><td>6:13110937|13117545</td><td>circRNA_010512</td><td>16:67411434|67415093</td><td>circRNA_000201</td><td>1:225790958|225811866</td><td>circRNA_012603</td><td>13:190108609|190201577</td></tr>
<tr><td>circRNA_018010</td><td>9:139959242|139965850</td><td>circRNA_010826</td><td>8:34753681|34757340</td><td>circRNA_002185</td><td>9:11319664|11340580</td><td>circRNA_013149</td><td>5:17246417|17339454</td></tr>
<tr><td>circRNA_011119</td><td>10:58746643|58753252</td><td>circRNA_012918</td><td>2:45046699|45050358</td><td>circRNA_018417</td><td>1:60422840|60443757</td><td>circRNA_010763</td><td>6:128736988|128830137</td></tr>
<tr><td>circRNA_012000</td><td>8:40654294|40660904</td><td>circRNA_021304</td><td>GL892805.1:67991|71652</td><td>circRNA_003964</td><td>2:88698666|88719584</td><td>circRNA_001892</td><td>6:112567763|112660921</td></tr>
<tr><td>circRNA_009074</td><td>15:157089364|157095975</td><td>circRNA_010308</td><td>11:15976293|15979955</td><td>circRNA_018788</td><td>15:85070396|85091324</td><td>circRNA_018222</td><td>2:50007088|50100415</td></tr>
<tr><td>circRNA_002603</td><td>1:143285207|143291819</td><td>circRNA_020021</td><td>13:156215035|156218697</td><td>circRNA_006988</td><td>13:38819847|38840777</td><td>circRNA_020442</td><td>1:195020817|195114161</td></tr>
<tr><td>circRNA_016695</td><td>1:268073269|268079882</td><td>circRNA_016347</td><td>8:32345748|32349411</td><td>circRNA_012661</td><td>14:68636056|68656990</td><td>circRNA_012361</td><td>10:37732934|37826342</td></tr>
<tr><td>circRNA_005161</td><td>9:120362998|120369612</td><td>circRNA_012178</td><td>1:3119705|3123369</td><td>circRNA_005449</td><td>1:134656768|134677703</td><td>circRNA_005753</td><td>13:217685619|217779051</td></tr>
<tr><td>circRNA_011155</td><td>11:84417144|84423761</td><td>circRNA_009194</td><td>18:41985402|41989067</td><td>circRNA_010764</td><td>6:132117736|132138673</td><td>circRNA_018988</td><td>3:84302257|84395892</td></tr>
<tr><td>circRNA_004683</td><td>6:145916377|145922995</td><td>circRNA_004430</td><td>5:37185163|37188829</td><td>circRNA_015349</td><td>10:45979341|46000278</td><td>circRNA_017837</td><td>4:88593582|88687228</td></tr>
<tr><td>circRNA_004113</td><td>3:80510313|80516932</td><td>circRNA_016202</td><td>6:32884132|32887804</td><td>circRNA_004302</td><td>4:79094980|79115918</td><td>circRNA_007629</td><td>4:31369704|31463369</td></tr>
<tr><td>circRNA_011229</td><td>13:72897241|72903860</td><td>circRNA_016792</td><td>13:44123948|44127620</td><td>circRNA_000095</td><td>1:124822521|124843460</td><td>circRNA_017342</td><td>9:94148684|94242425</td></tr>
<tr><td>circRNA_005034</td><td>8:144846439|144853059</td><td>circRNA_007593</td><td>3:109680739|109684412</td><td>circRNA_015069</td><td>X:45938682|45959622</td><td>circRNA_019361</td><td>1:29491795|29585537</td></tr>
<tr><td>circRNA_017379</td><td>JH118551.1:32085|38706</td><td>circRNA_015371</td><td>11:6800250|6803923</td><td>circRNA_019708</td><td>4:121726651|121747592</td><td>circRNA_014745</td><td>9:94148684|94242462</td></tr>
<tr><td>circRNA_003380</td><td>14:77656419|77663041</td><td>circRNA_015296</td><td>1:266459360|266463034</td><td>circRNA_000267</td><td>1:300377341|300398288</td><td>circRNA_010156</td><td>X:125734578|125828524</td></tr>
<tr><td>circRNA_011095</td><td>10:29344432|29351054</td><td>circRNA_004547</td><td>6:33790614|33794291</td><td>circRNA_002450</td><td>X:139925204|139946155</td><td>circRNA_005812</td><td>14:136612215|136706295</td></tr>
<tr><td>circRNA_003111</td><td>13:42379269|42385893</td><td>circRNA_017942</td><td>7:105392983|105396660</td><td>circRNA_003663</td><td>16:22536691|22557643</td><td>circRNA_009434</td><td>4:17136598|17230779</td></tr>
<tr><td>circRNA_007379</td><td>18:5627894|5634518</td><td>circRNA_008628</td><td>11:86951625|86955303</td><td>circRNA_000589</td><td>13:51765369|51786322</td><td>circRNA_016668</td><td>1:195020817|195115065</td></tr>
<tr><td>circRNA_019976</td><td>12:14684074|14690700</td><td>circRNA_014888</td><td>9:101847443|101851121</td><td>circRNA_000021</td><td>1:17240890|17261849</td><td>circRNA_003172</td><td>13:91154925|91249175</td></tr>
<tr><td>circRNA_020215</td><td>6:50127662|50134288</td><td>circRNA_001918</td><td>6:146619070|146622749</td><td>circRNA_013991</td><td>1:280679418|280700379</td><td>circRNA_006414</td><td>8:80884288|80978568</td></tr>
<tr><td>circRNA_006091</td><td>3:92530938|92537565</td><td>circRNA_010377</td><td>13:81543363|81547042</td><td>circRNA_008607</td><td>11:26280706|26301669</td><td>circRNA_009378</td><td>3:82434774|82529118</td></tr>
<tr><td>circRNA_010158</td><td>X:126981120|126987749</td><td>circRNA_012714</td><td>15:51607781|51611461</td><td>circRNA_020900</td><td>2:98305833|98326796</td><td>circRNA_010113</td><td>X:30816160|30910575</td></tr>
<tr><td>circRNA_000406</td><td>11:25517563|25524196</td><td>circRNA_006731</td><td>1:195277533|195281214</td><td>circRNA_015170</td><td>1:59510004|59530977</td><td>circRNA_018928</td><td>2:91169756|91264363</td></tr>
<tr><td>circRNA_011158</td><td>12:6057442|6064077</td><td>circRNA_016405</td><td>8:139958736|139962418</td><td>circRNA_012229</td><td>1:101178705|101199679</td><td>circRNA_001191</td><td>18:52262210|52356954</td></tr>
<tr><td>circRNA_014721</td><td>6:99190172|99196807</td><td>circRNA_006583</td><td>X:114567036|114570719</td><td>circRNA_009164</td><td>17:59464011|59484993</td><td>circRNA_018843</td><td>17:28173576|28268431</td></tr>
<tr><td>circRNA_002207</td><td>9:30651942|30658578</td><td>circRNA_014595</td><td>X:21586693|21590376</td><td>circRNA_006812</td><td>10:17046557|17067546</td><td>circRNA_002118</td><td>8:91902449|91997361</td></tr>
<tr><td>circRNA_021221</td><td>8:109416257|109422893</td><td>circRNA_005940</td><td>18:10898787|10902471</td><td>circRNA_018855</td><td>17:45655690|45676687</td><td>circRNA_015367</td><td>11:3679167|3774119</td></tr>
<tr><td>circRNA_002829</td><td>10:49667206|49673843</td><td>circRNA_007264</td><td>15:92653934|92657618</td><td>circRNA_004689</td><td>6:147024893|147045896</td><td>circRNA_014844</td><td>5:87157448|87252405</td></tr>
<tr><td>circRNA_005261</td><td>GL895037.1:19231|25870</td><td>circRNA_012518</td><td>13:33954373|33958057</td><td>circRNA_010012</td><td>9:125451231|125472242</td><td>circRNA_013398</td><td>8:83358407|83453422</td></tr>
<tr><td>circRNA_012374</td><td>10:49621863|49628502</td><td>circRNA_021202</td><td>8:58301883|58305568</td><td>circRNA_002628</td><td>1:176156294|176177307</td><td>circRNA_019433</td><td>10:58766088|58861115</td></tr>
<tr><td>circRNA_012813</td><td>16:51257587|51264227</td><td>circRNA_019955</td><td>1:313827246|313830932</td><td>circRNA_014123</td><td>1:176794813|176815836</td><td>circRNA_013097</td><td>4:46363383|46458535</td></tr>
</table>

circRNA_020774	16:6434889|6441073	circRNA_014632	12:44489965|44493652	circRNA_011675	3:42032747|42053774	circRNA_010642	3:122008499|122103795
circRNA_004413	5:17119726|17125911	circRNA_012240	1:118180632|118184320	circRNA_020514	10:54125708|54146738	circRNA_002133	8:120604793|120700114
circRNA_013478	9:101486703|101492888	circRNA_014531	8:103708394|103712082	circRNA_003625	15:120969439|120990471	circRNA_020359	X:126789683|126885048
circRNA_006403	8:57805777|57811963	circRNA_007527	3:7183714|7187406	circRNA_002122	8:93287563|93308596	circRNA_013135	4:135651116|135746525
circRNA_009017	15:67009488|67015674	circRNA_012913	2:30477465|30481157	circRNA_002123	8:93287563|93308599	circRNA_004909	8:39666147|39761604
circRNA_006526	GL893953.2:87898|94086	circRNA_017939	7:86333710|86337402	circRNA_001815	6:67397934|67418987	circRNA_001822	6:73334893|73430424
circRNA_011584	2:45045796|45051984	circRNA_010250	1:280458972|280462665	circRNA_011156	11:86983006|87004064	circRNA_000517	12:62428106|62523689
circRNA_013699	18:10879415|10885605	circRNA_019710	4:129473418|129477112	circRNA_001647	4:128903356|128924416	circRNA_002182	9:11252773|11348456
circRNA_020267	7:82511993|82518183	circRNA_004564	6:69686926|69690621	circRNA_019027	4:55211975|55233036	circRNA_020296	8:120604362|120700114
circRNA_020714	14:136297545|136303736	circRNA_005915	17:15481868|15485565	circRNA_010184	1:60938694|60959765	circRNA_021228	8:120604334|120700114
circRNA_017280	8:77402399|77408591	circRNA_015578	13:202715441|202719139	circRNA_007585	3:104192112|104213184	circRNA_000280	10:12274354|12370142
circRNA_005516	1:270137406|270143605	circRNA_004735	7:26692139|26695838	circRNA_003454	14:132237197|132258271	circRNA_000722	13:217683188|217779051
circRNA_006338	7:61615699|61621899	circRNA_005080	9:23112748|23116450	circRNA_019239	8:144218585|144239660	circRNA_009427	3:137503688|137599665
circRNA_012981	2:148186310|148192512	circRNA_012688	14:122455302|122459005	circRNA_019308	GL893689.1:3069|24146	circRNA_002018	7:97005367|97101356
circRNA_009746	7:45323367|45329571	circRNA_005544	10:24586809|24590513	circRNA_014756	X:70664081|70685159	circRNA_005752	13:217683038|217779051
circRNA_011700	3:77052394|77058602	circRNA_001756	5:86263829|86267536	circRNA_006710	1:161199573|161220652	circRNA_001798	6:46472705|46568764
circRNA_013408	8:109429079|109435287	circRNA_006084	3:84139238|84142945	circRNA_020522	11:5274152|5295233	circRNA_006892	11:52691991|52788105
circRNA_018764	14:132243465|132249674	circRNA_008379	1:142523717|142527425	circRNA_017246	7:124952570|124973655	circRNA_020909	2:122395490|122491649
circRNA_020760	15:117366653|117372862	circRNA_004578	6:78655585|78659294	circRNA_004667	6:136013083|136034171	circRNA_009406	3:120066344|120162510
circRNA_006170	4:107403982|107410192	circRNA_008306	1:36715010|36718719	circRNA_006214	5:70319577|70340673	circRNA_018205	18:24326227|24422485
circRNA_001128	17:59478778|59484993	circRNA_010777	6:157496770|157500479	circRNA_007755	5:68106961|68128063	circRNA_010127	X:67430148|67526460
circRNA_007377	18:1942538|1948753	circRNA_001458	3:90751781|90755491	circRNA_019454	11:84964946|84986054	circRNA_005756	14:434044|530434
circRNA_007412	18:59551551|59557766	circRNA_020260	7:60157993|60161704	circRNA_017396	X:80967681|80988790	circRNA_003838	18:25511592|25608146
circRNA_006632	1:49668384|49674605	circRNA_001068	16:51704344|51708056	circRNA_009990	9:88659895|88681009	circRNA_014468	6:46472705|46569279
circRNA_008466	1:249782241|249788463	circRNA_019640	2:106413082|106416794	circRNA_017902	6:96831302|96852417	circRNA_020870	2:50889260|50985835
circRNA_020320	9:46890981|46897203	circRNA_003752	17:19087158|19090873	circRNA_004978	8:93220585|93241704	circRNA_018136	13:142503400|142600017
circRNA_014573	9:143537305|143543529	circRNA_003903	2:43779976|43783691	circRNA_006220	5:77097846|77118965	circRNA_013486	9:115608962|115705584
circRNA_004226	4:2625060|2631285	circRNA_011687	3:61510938|61514653	circRNA_002213	9:37819879|37841000	circRNA_018482	1:195029560|195126252
circRNA_013875	2:449197|455423	circRNA_013226	6:26380052|26383767	circRNA_009555	5:61467036|61488161	circRNA_001862	6:99220825|99317549
circRNA_002682	1:210254703|210260930	circRNA_018355	8:118372513|118376228	circRNA_002752	1:284320474|284341604	circRNA_013491	9:119329224|119425986
circRNA_020138	3:45423785|45430012	circRNA_018914	2:73202230|73205946	circRNA_004907	8:39516211|39537344	circRNA_004925	8:57362540|57459321
circRNA_017614	13:170385990|170392219	circRNA_006261	6:41347054|41350773	circRNA_015235	1:200385534|200406670	circRNA_002128	8:110582904|110679703
circRNA_014604	X:116389266|116395496	circRNA_011548	18:31018819|31022538	circRNA_017903	6:101724150|101745288	circRNA_012392	10:62692304|62789150
circRNA_003069	13:16467579|16473811	circRNA_006050	2:150783691|150787411	circRNA_004789	7:65664182|65685321	circRNA_009961	9:44936884|45033735
circRNA_010485	15:93770939|93777171	circRNA_008070	8:144846439|144850159	circRNA_006935	12:37117255|37138400	circRNA_013246	6:77308449|77405378
circRNA_004094	3:58982442|58988675	circRNA_001554	4:20215577|20219298	circRNA_002871	11:5189667|5210814	circRNA_020222	6:77308449|77405410
circRNA_018841	17:15481868|15488101	circRNA_013966	9:101934847|101938570	circRNA_018759	14:117272265|117293415	circRNA_006401	8:44222917|44319884
circRNA_016346	8:32185792|32192028	circRNA_003790	17:52395572|52399297	circRNA_014597	X:61016381|61037536	circRNA_002820	10:46681560|46779066
circRNA_009638	6:64286222|64292459	circRNA_012960	2:106416227|106419952	circRNA_019652	2:139097672|139118827	circRNA_017819	4:15522028|15619539
circRNA_010973	1:83293024|83299261	circRNA_008126	9:70888223|70891949	circRNA_003999	2:119263024|119284184	circRNA_008313	1:45640355|45737883
circRNA_017328	9:52667526|52673763	circRNA_000570	13:35739632|35743359	circRNA_002538	1:94556573|94577735	circRNA_003406	14:92407605|92505193
circRNA_019056	4:139879528|139885766	circRNA_016648	1:134614334|134618061	circRNA_005594	11:25887731|25908902	circRNA_015199	1:113952119|114049837
circRNA_020812	17:43318344|43324582	circRNA_008265	X:90713301|90717029	circRNA_018797	15:101687076|101708250	circRNA_001414	3:47943112|48040842
circRNA_012140	GL896549.1:14676|20915	circRNA_020356	X:63287516|63291247	circRNA_010146	X:105400134|105421313	circRNA_013018	3:62982993|63080743
circRNA_003548	15:79683656|79689898	circRNA_018873	18:35542631|35546365	circRNA_017128	4:128674738|128695923	circRNA_001433	3:62982989|63080743
circRNA_007897	7:14122239|14128481	circRNA_009100	16:37565228|37568964	circRNA_000810	14:78015574|78036782	circRNA_017998	9:88634775|88732539
circRNA_012306	1:242725331|242731574	circRNA_014867	7:97990721|97994457	circRNA_016584	X:35584870|35606086	circRNA_017432	1:61815052|61912985
circRNA_016653	1:142965376|142971620	circRNA_011197	13:8094772|8098509	circRNA_016873	14:114872934|114894153	circRNA_019190	7:109722597|109820542
circRNA_017713	16:53246049|53252293	circRNA_019485	13:76119001|76122738	circRNA_019452	11:56125252|56146474	circRNA_010591	2:130566550|130664547
circRNA_007872	6:136802506|136808752	circRNA_002195	9:13315285|13319023	circRNA_018151	14:34675990|34697216	circRNA_003561	15:85375263|85473265
circRNA_009491	4:110128012|110134258	circRNA_007425	2:16092401|16096142	circRNA_001229	2:28863997|28885224	circRNA_010270	10:18101700|18199788
circRNA_016935	16:37501728|37507976	circRNA_008383	1:143634011|143637753	circRNA_002654	1:200269189|200290416	circRNA_001562	4:40262034|40360156

circRNA_014292	15:86498639|86504888	circRNA_009325	3:10658314|10662056	circRNA_009948	9:40955217|40976445	circRNA_013725	7:97058335|97156735
circRNA_020947	3:34655376|34661627	circRNA_018760	14:124833649|124837391	circRNA_016328	7:124961813|124983041	circRNA_012707	15:36264244|36362665
circRNA_009019	15:71720780|71727032	circRNA_008576	11:2098052|2101796	circRNA_018878	18:40626773|40648001	circRNA_006444	9:2388577|2487067
circRNA_017521	10:46284389|46290641	circRNA_008861	13:210659850|210663594	circRNA_005119	9:52650662|52671892	circRNA_002150	8:134379373|134477954
circRNA_014108	1:93269648|93275902	circRNA_001200	18:59685146|59688893	circRNA_010518	17:5196690|5217932	circRNA_000982	15:106056959|106155553
circRNA_003584	15:93750378|93756633	circRNA_018601	12:37228113|37231860	circRNA_001891	6:111913606|111934854	circRNA_014463	6:12077068|12175731
circRNA_004270	4:45558506|45564762	circRNA_010681	4:99420312|99424060	circRNA_010707	5:63605306|63626555	circRNA_006087	3:84723879|84822563
circRNA_015120	14:32570962|32577219	circRNA_015264	1:227185988|227189737	circRNA_007169	14:114857540|114878790	circRNA_000417	11:52691991|52790682
circRNA_008549	10:53856831|53863094	circRNA_002418	X:21717559|21721310	circRNA_019850	9:98323811|98345068	circRNA_015916	2:124232728|124331446
circRNA_004054	3:11499694|11505958	circRNA_010253	1:293084019|293087770	circRNA_013377	8:32689706|32710967	circRNA_020363	1:6387477|6486326
circRNA_014873	8:40452798|40459063	circRNA_017941	7:94511177|94514929	circRNA_000647	13:130082229|130103494	circRNA_001004	15:119988764|120087629
circRNA_000499	12:47119831|47126097	circRNA_009398	3:107578304|107582057	circRNA_013876	2:3018042|3039309	circRNA_007634	4:34617495|34716368
circRNA_014166	10:77676098|77682364	circRNA_018485	1:199987183|199990936	circRNA_014027	7:97118845|97140112	circRNA_002528	1:86117059|86215967
circRNA_013894	3:9520792|9527059	circRNA_019742	5:92748226|92751979	circRNA_019839	9:7115760|7137030	circRNA_016149	5:53531603|53630601
circRNA_014806	17:20669421|20675688	circRNA_007637	4:37565201|37568955	circRNA_013034	3:97807254|97828527	circRNA_016179	5:93227769|93326810
circRNA_016893	15:51622337|51628605	circRNA_013947	8:5755686|5759442	circRNA_013686	13:140978250|140999525	circRNA_021146	7:13056968|13156103
circRNA_020041	14:67475936|67482204	circRNA_018481	1:195022523|195026279	circRNA_014502	7:91917856|91939138	circRNA_009793	7:97005367|97104509
circRNA_008799	13:100593724|100599993	circRNA_020192	5:23672122|23675878	circRNA_007738	5:37032661|37053948	circRNA_006443	9:2387852|2487067
circRNA_010646	3:133715005|133721279	circRNA_002873	11:5263855|5267612	circRNA_012746	15:103640291|103661579	circRNA_019540	14:83196332|83295682
circRNA_000569	13:35652573|35658848	circRNA_014880	8:115198972|115202729	circRNA_020033	14:45824505|45845794	circRNA_008315	1:48636668|48736020
circRNA_018319	6:132217493|132223770	circRNA_006949	12:47122339|47126097	circRNA_009646	6:73314439|73335732	circRNA_005610	12:12862834|12962192
circRNA_010048	GL892805.1:67991|74269	circRNA_014429	4:74554441|74558200	circRNA_017434	1:63721691|63742985	circRNA_011621	2:104942961|105042335
circRNA_001059	16:37504640|37510922	circRNA_011645	2:148134331|148138091	circRNA_001927	6:148401539|148422837	circRNA_005404	1:16771664|16871069
circRNA_011360	14:115642256|115648541	circRNA_008984	15:19247885|19251646	circRNA_003307	14:21462664|21483971	circRNA_011547	18:25508672|25608146
circRNA_000840	14:113577307|113583593	circRNA_019436	10:76099308|76103069	circRNA_010988	1:125298219|125319526	circRNA_003052	12:62424103|62523689
circRNA_019874	GL895152.1:21004|27290	circRNA_015655	14:98554274|98558037	circRNA_015203	1:123202006|123223316	circRNA_017277	8:71256451|71356267
circRNA_008556	10:56077005|56083293	circRNA_013046	3:109654649|109658413	circRNA_016293	7:57259483|57280795	circRNA_004222	4:592530|692379
circRNA_006994	13:43756823|43763114	circRNA_006093	3:98128544|98132309	circRNA_000478	12:37398861|37420176	circRNA_016066	4:48056407|48156272
circRNA_008195	GL893669.1:73398|79691	circRNA_015411	12:4645549|4649314	circRNA_007106	14:30058998|30080315	circRNA_001506	3:118060748|118160655
circRNA_009757	7:60962171|60968464	circRNA_019887	X:67157335|67161100	circRNA_010226	1:200542290|200563613	circRNA_018110	11:16913376|17013395
circRNA_000645	13:118650380|118656674	circRNA_002545	1:101199341|101203107	circRNA_002036	7:117016827|117038151	circRNA_010856	8:143175337|143275381
circRNA_011139	11:22243068|22249363	circRNA_017832	4:55329545|55333311	circRNA_020503	10:29339600|29360924	circRNA_019988	13:7144546|7244591
circRNA_003332	14:32735062|32741361	circRNA_010930	X:16108943|16112710	circRNA_018249	3:16572731|16594063	circRNA_014911	11:24690709|24791057
circRNA_004136	3:88079394|88085693	circRNA_002459	1:9393570|9397339	circRNA_000395	11:19715398|19736732	circRNA_008512	10:17046557|17146941
circRNA_020025	13:205663203|205669502	circRNA_003313	14:24811227|24814996	circRNA_008520	10:31342999|31364339	circRNA_015026	2:119165841|119266346
circRNA_004358	4:128233869|128240169	circRNA_015068	X:30975313|30979082	circRNA_003244	13:167096198|167117540	circRNA_000959	15:86571013|86671532
circRNA_008378	1:135371126|135377427	circRNA_005252	GL894597.1:103377|107147	circRNA_013444	9:31854903|31876246	circRNA_011562	2:3009236|3109853
circRNA_011162	12:15586419|15592721	circRNA_013222	6:17385723|17389493	circRNA_018019	GL893006.1:1409|22753	circRNA_008028	8:82828282|82928907
circRNA_009420	3:134248342|134254646	circRNA_011957	7:73287698|73291470	circRNA_008320	1:56870737|56892084	circRNA_020040	14:52602464|52703108
circRNA_013915	4:90704802|90711107	circRNA_002352	GL894597.1:44194|47967	circRNA_015513	13:58128469|58149818	circRNA_020374	1:32129558|32230252
circRNA_008134	9:79218044|79224350	circRNA_009569	5:72248349|72252123	circRNA_017192	6:112427242|112448596	circRNA_016928	16:23393877|23494582
circRNA_009020	15:73545877|73552183	circRNA_015153	1:28660706|28664480	circRNA_012709	15:44253492|44274857	circRNA_002721	1:256878727|256979460
circRNA_011639	2:140902693|140908999	circRNA_016599	X:90783693|90787468	circRNA_013972	GL893884.1:31770|53137	circRNA_009677	6:99216722|99317549
circRNA_016968	18:5458415|5464721	circRNA_004162	3:106902038|106905816	circRNA_000592	13:58776339|58797708	circRNA_004117	3:81716210|81817069
circRNA_020444	1:200281377|200287684	circRNA_012782	15:143217390|143221168	circRNA_019205	8:40313491|40334860	circRNA_016243	6:99446721|99547687
circRNA_009411	3:121691230|121697538	circRNA_006554	X:14262458|14266237	circRNA_018861	18:9562088|9583462	circRNA_018553	10:53519107|53620073
circRNA_013959	9:37001749|37008057	circRNA_010278	10:35240929|35244709	circRNA_004314	4:93607963|93629339	circRNA_018114	11:76373396|76474363
circRNA_003155	13:84435864|84442173	circRNA_003657	15:157407747|157411528	circRNA_017742	18:43157938|43179317	circRNA_011828	5:79090794|79191789
circRNA_003559	15:85340631|85346940	circRNA_019996	13:37680293|37684074	circRNA_021074	5:89555989|89577371	circRNA_016358	8:44339341|44440365
circRNA_006099	3:107580168|107586477	circRNA_006012	2:90128254|90132036	circRNA_021236	8:141080303|141101699	circRNA_015567	13:160345835|160446925
circRNA_012763	15:116365668|116371977	circRNA_007096	14:10459275|10463058	circRNA_013981	X:139963482|139984891	circRNA_009156	17:45773841|45874955
circRNA_013821	13:106763935|106770245	circRNA_008893	14:34693433|34697216	circRNA_010304	11:7817381|7838798	circRNA_012150	X:14511526|14612741

circRNA_009125	16:78211482|78217793	circRNA_009659	6:85269339|85273122	circRNA_006485	9:79015624|79037043	circRNA_000598	13:73547020|73648259
circRNA_011217	13:42311419|42317731	circRNA_001707	5:38557794|38561578	circRNA_000817	14:82393345|82414766	circRNA_001176	18:25506902|25608146
circRNA_021335	GL896381.1:6954|13266	circRNA_004802	7:73108056|73111840	circRNA_007807	6:38256508|38277935	circRNA_016059	4:39819040|39920373
circRNA_008791	13:90946479|90952792	circRNA_003788	17:51655197|51658984	circRNA_021156	7:30110338|30131766	circRNA_012182	1:16769724|16871069
circRNA_002774	10:17067380|17073695	circRNA_003337	14:34693427|34697216	circRNA_001065	16:47827438|47848867	circRNA_013430	9:234843|336270
circRNA_009759	7:62829756|62836071	circRNA_018440	1:127648623|127652412	circRNA_003685	16:31971561|31992995	circRNA_019668	3:29618701|29720141
circRNA_013826	13:136691991|136698308	circRNA_020551	12:25819429|25823218	circRNA_014733	8:71567714|71589148	circRNA_005427	1:86114517|86215967
circRNA_015228	1:181479020|181485339	circRNA_009621	6:26367274|26371064	circRNA_005788	14:65324436|65345871	circRNA_015159	1:38479516|38581068
circRNA_008351	1:105923807|105930130	circRNA_016249	6:103690733|103694523	circRNA_012462	12:11461813|11483250	circRNA_015344	10:41844753|41946308
circRNA_011060	1:270329795|270336118	circRNA_004342	4:116731304|116735096	circRNA_001061	16:43232224|43253665	circRNA_004931	8:57919106|58020691
circRNA_013255	6:88268086|88274409	circRNA_005254	GL894772.1:8659|12452	circRNA_001649	4:129315285|129336726	circRNA_017822	4:16634675|16736515
circRNA_003529	15:61402305|61408631	circRNA_014452	5:61221069|61224862	circRNA_013307	7:21551602|21573043	circRNA_005458	1:143361257|143463105
circRNA_012264	1:145031571|145037897	circRNA_017457	1:134774498|134778291	circRNA_009538	5:38575947|38597389	circRNA_018743	14:71960117|72062013
circRNA_015963	3:40978570|40984897	circRNA_016656	1:145004014|145007808	circRNA_000203	1:227165198|227186649	circRNA_009892	8:125231090|125333086
circRNA_008147	9:101480476|101486805	circRNA_008744	13:43391092|43394888	circRNA_001913	6:140633018|140654469	circRNA_004419	5:24427778|24529952
circRNA_013508	GL892407.1:21562|27892	circRNA_004783	7:64448638|64452436	circRNA_019690	3:135358108|135379562	circRNA_010694	5:23414033|23516308
circRNA_002398	MT:1680|8012	circRNA_017316	9:21848969|21852767	circRNA_006599	1:6464869|6486326	circRNA_005869	15:118153598|118255877
circRNA_011623	2:107642782|107649114	circRNA_012062	9:46182553|46186353	circRNA_002518	1:72497312|72518770	circRNA_012731	15:82802125|82904514
circRNA_008858	13:209969995|209976329	circRNA_014915	13:33957923|33961723	circRNA_002638	1:183813973|183835431	circRNA_000655	13:132473582|132575980
circRNA_002087	8:45605339|45611674	circRNA_016460	9:50821415|50825216	circRNA_010024	9:140247518|140268980	circRNA_008142	9:97084813|97187229
circRNA_011847	5:94439224|94445562	circRNA_008499	1:299797832|299801634	circRNA_016308	7:77877261|77898724	circRNA_004784	7:65150234|65252697
circRNA_006100	3:107854455|107860794	circRNA_017142	5:60354907|60358709	circRNA_000277	10:9709752|9731216	circRNA_020325	9:88630066|88732539
circRNA_014626	11:12312481|12318821	circRNA_014946	8:40455259|40459063	circRNA_010472	15:55414565|55436029	circRNA_004660	6:130939289|131041882
circRNA_001931	6:150888884|150895226	circRNA_016190	6:13097978|13101783	circRNA_003730	16:65133423|65154889	circRNA_019788	7:17209021|17311662
circRNA_002698	1:233180440|233186782	circRNA_012441	11:53900017|53903824	circRNA_016729	10:69791009|69812475	circRNA_003598	15:111234765|111337441
circRNA_008111	9:46187651|46193994	circRNA_017901	6:96441461|96445268	circRNA_016463	9:52671499|52692967	circRNA_006707	1:160169128|160271923
circRNA_013112	4:83680009|83686353	circRNA_005730	13:148479934|148483742	circRNA_017753	2:52346942|52368417	circRNA_005881	16:4095540|4198345
circRNA_008940	14:96079948|96086298	circRNA_013996	11:69705397|69709208	circRNA_018753	14:114857540|114879015	circRNA_000045	1:44437423|44540267
circRNA_015254	1:212203332|212209687	circRNA_018815	16:22498896|22502707	circRNA_001496	3:113999359|114020837	circRNA_012180	1:16080529|16183454
circRNA_003077	13:21193239|21199595	circRNA_001500	3:114349157|114352969	circRNA_010684	4:128674738|128696217	circRNA_012435	11:24688121|24791057
circRNA_006669	1:110526474|110532832	circRNA_005792	14:82403576|82407388	circRNA_002432	X:101588344|101609826	circRNA_014148	10:17067380|17170362
circRNA_012567	13:97366509|97372869	circRNA_015896	2:86246358|86250170	circRNA_001958	7:33242142|33263635	circRNA_007958	7:109722597|109825592
circRNA_001469	3:98680367|98686730	circRNA_018256	3:74730617|74734433	circRNA_020720	14:140198967|140220475	circRNA_002889	11:14710083|14813304
circRNA_005180	9:132895736|132902099	circRNA_003153	13:83142279|83146096	circRNA_003849	18:41996051|42017561	circRNA_008472	1:256878727|256981965
circRNA_018580	11:25887731|25894094	circRNA_004937	8:58659366|58663183	circRNA_012932	2:66875163|66896675	circRNA_016131	5:23004492|23107838
circRNA_019353	X:116396306|116402669	circRNA_005037	9:31241|35059	circRNA_003461	14:136289720|136311233	circRNA_007737	5:36340841|36444197
circRNA_002021	7:97739664|97746028	circRNA_011578	2:30460314|30464133	circRNA_016686	1:242087550|242109063	circRNA_002108	8:82960858|83064254
circRNA_006791	1:293511501|293517866	circRNA_020955	3:49903623|49907442	circRNA_018808	15:131242980|131264493	circRNA_007984	8:9767470|9871034
circRNA_019396	1:211714940|211721306	circRNA_009282	2:108991584|108995405	circRNA_003918	2:58219608|58241123	circRNA_004175	3:110373462|110477188
circRNA_002327	GL892843.1:74235|80602	circRNA_004865	7:130879957|130883781	circRNA_001407	3:39449044|39470560	circRNA_018483	1:195029560|195133308
circRNA_011802	5:15638101|15644469	circRNA_006370	7:121213886|121217710	circRNA_009003	15:53415811|53437329	circRNA_000421	11:54369518|54473280
circRNA_008503	1:310128560|310134929	circRNA_000858	14:132243465|132247290	circRNA_011408	15:67038844|67060363	circRNA_003565	15:85397016|85500787
circRNA_016370	8:57881908|57888277	circRNA_003670	16:23676740|23680565	circRNA_018940	2:121273902|121295422	circRNA_014369	2:118253397|118357183
circRNA_014034	1:16395773|16402143	circRNA_019319	GL895413.1:777|4602	circRNA_002483	1:25518797|25540318	circRNA_018257	3:84712662|84816461
circRNA_020751	15:96557829|96564200	circRNA_002481	1:25357026|25360852	circRNA_015364	10:63625465|63646997	circRNA_019251	9:25256510|25360320
circRNA_019866	GL893735.2:18429|24801	circRNA_005440	1:118441847|118445673	circRNA_000892	15:22746712|22768247	circRNA_020377	1:45701230|45805070
circRNA_009953	9:42980485|42986859	circRNA_010563	2:27611157|27614983	circRNA_021341	JH118545.1:38409|59944	circRNA_002710	1:249759669|249863571
circRNA_008209	GL894597.1:41592|47967	circRNA_000363	10:75687658|75691485	circRNA_000805	14:77581776|77603313	circRNA_006124	4:14476990|14580940
circRNA_003951	2:86243794|86250170	circRNA_012887	18:55610823|55614651	circRNA_011267	13:145395420|145416967	circRNA_000472	12:27207179|27311177
circRNA_002333	GL893413.1:37808|44185	circRNA_016178	5:92145446|92149275	circRNA_021214	8:89353525|89375078	circRNA_014362	2:61533613|61637637
circRNA_013860	16:23465932|23472310	circRNA_010548	18:41970023|41973853	circRNA_007885	6:148401539|148423094	circRNA_006805	10:12825482|12929568
circRNA_006462	9:29830958|29837338	circRNA_003267	13:210784081|210787912	circRNA_018647	13:90903716|90925273	circRNA_015268	1:229131344|229235510

circRNA_000490	12:44349757|44356138	circRNA_004190	3:120081729|120085560	circRNA_003795	17:55956945|55978505	circRNA_019764	6:93011929|93116232
circRNA_014171	11:21001197|21007581	circRNA_004016	2:135210890|135214723	circRNA_013310	7:33242142|33263702	circRNA_005975	2:41825697|41930028
circRNA_016853	14:65879938|65886323	circRNA_008230	JH118792.1:77193|81026	circRNA_011521	17:43061013|43082579	circRNA_004785	7:65150234|65254661
circRNA_004810	7:86329719|86336105	circRNA_020697	14:82228502|82232335	circRNA_019939	1:245635102|245656673	circRNA_006756	1:240848249|240952881
circRNA_015044	7:10297937|10304324	circRNA_001832	6:82345100|82348935	circRNA_005178	9:132273623|132295195	circRNA_009542	5:50595002|50699658
circRNA_004075	3:38637515|38643903	circRNA_013458	9:46890981|46894816	circRNA_005015	8:140260091|140281666	circRNA_000910	15:53008500|53113201
circRNA_013090	4:41735670|41742058	circRNA_017857	5:32070350|32074186	circRNA_003232	13:150105423|150126999	circRNA_006673	1:113945018|114049837
circRNA_003775	17:43362875|43369268	circRNA_012529	13:42346716|42350553	circRNA_003500	15:19247885|19269467	circRNA_020943	3:29760501|29865377
circRNA_000251	1:278920982|278927376	circRNA_003494	15:18646758|18650596	circRNA_003420	14:110126707|110148292	circRNA_003827	18:18610834|18715721
circRNA_019607	18:36461492|36467886	circRNA_006276	6:82345096|82348935	circRNA_015370	11:6780998|6802586	circRNA_011500	16:56296022|56401078
circRNA_016666	1:186651443|186657838	circRNA_013563	X:7258915|7262755	circRNA_015765	16:47826796|47848392	circRNA_006590	X:144028110|144133203
circRNA_006460	9:29012165|29018562	circRNA_011780	4:90657864|90661705	circRNA_018680	13:166795034|166816631	circRNA_019402	1:229141254|229246387
circRNA_009549	5:54725893|54732290	circRNA_003784	17:46488750|46492592	circRNA_020064	15:104516540|104538139	circRNA_004225	4:2602892|2708122
circRNA_008577	11:5274152|5280553	circRNA_005760	14:10533567|10537409	circRNA_001575	4:45690143|45711744	circRNA_009675	6:99212305|99317549
circRNA_007381	18:9779444|9785846	circRNA_009652	6:81212689|81216532	circRNA_010243	1:242728749|242750357	circRNA_007635	4:36253818|36359128
circRNA_014072	15:147708660|147715063	circRNA_018987	3:83801277|83805120	circRNA_020978	3:103781514|103803130	circRNA_013800	11:54369518|54474857
circRNA_021044	5:12506860|12513267	circRNA_007304	16:25321505|25325352	circRNA_007385	18:15081212|15102829	circRNA_017755	2:61532136|61637637
circRNA_010060	GL894350.2:20200|26609	circRNA_011510	17:15999829|16003676	circRNA_013901	3:107410294|107431917	circRNA_019094	6:32239126|32344661
circRNA_006799	1:311038381|311044794	circRNA_014154	10:31874232|31878079	circRNA_016262	6:148040878|148062504	circRNA_011043	1:225007114|225112654
circRNA_007626	4:17240787|17247200	circRNA_018301	6:7145257|7149104	circRNA_020355	X:45937992|45959622	circRNA_004461	5:70588009|70693708
circRNA_010974	1:83316653|83323066	circRNA_002925	11:53822474|53826322	circRNA_009286	2:112531676|112553307	circRNA_014616	1:269689085|269794789
circRNA_015549	13:141191882|141198299	circRNA_013349	7:104138883|104142731	circRNA_020709	14:122678521|122700154	circRNA_003879	2:7615361|7721209
circRNA_006139	4:40353738|40360156	circRNA_014473	6:81212689|81216538	circRNA_011951	7:67547452|67569094	circRNA_012307	1:244316290|244422185
circRNA_007324	16:51185829|51192247	circRNA_003700	16:42904912|42908762	circRNA_018612	12:49362946|49384594	circRNA_010899	9:132262224|132368236
circRNA_000668	13:144052007|144058426	circRNA_014147	10:140757|144607	circRNA_010904	9:136557895|136579549	circRNA_008494	1:284643223|284749270
circRNA_001235	2:31786725|31793144	circRNA_021189	8:31876392|31880242	circRNA_012184	1:17224292|17245947	circRNA_000599	13:73547020|73653108
circRNA_020091	17:41840202|41846621	circRNA_008078	9:9307763|9311614	circRNA_016586	X:45937966|45959622	circRNA_007902	7:24642345|24748519
circRNA_008681	12:46857007|46863427	circRNA_011692	3:72585580|72589431	circRNA_011426	15:97011015|97032673	circRNA_001218	2:7615361|7721555
circRNA_002347	GL894320.1:28024|34445	circRNA_011520	17:43051169|43055022	circRNA_015827	18:24501734|24523395	circRNA_009735	7:24642526|24748730
circRNA_002900	11:21795724|21802145	circRNA_014076	6:78517520|78521374	circRNA_007530	3:10492396|10514059	circRNA_009742	7:39021067|39127275
circRNA_006706	1:159929664|159936086	circRNA_002067	8:29293951|29297807	circRNA_014698	3:135328996|135350662	circRNA_020509	10:46613551|46720028
circRNA_013643	8:55110764|55117186	circRNA_003823	18:15785406|15789262	circRNA_018559	10:71355140|71376807	circRNA_015255	1:215651154|215757725
circRNA_005562	10:54085312|54091735	circRNA_007692	4:121743736|121747592	circRNA_003891	2:28405915|28427583	circRNA_021105	6:73314439|73421034
circRNA_011999	8:40321067|40327490	circRNA_012506	13:3078983|3082840	circRNA_014967	1:229173474|229195144	circRNA_016983	18:20362353|20468996
circRNA_017634	14:65329599|65336023	circRNA_003989	2:107642782|107646640	circRNA_010961	1:35479001|35500672	circRNA_019517	14:7176205|7282852
circRNA_004038	2:148139473|148145898	circRNA_017736	18:15785403|15789262	circRNA_008574	11:245450|267124	circRNA_017671	15:86564756|86671532
circRNA_013842	14:131867981|131874408	circRNA_002992	12:31696118|31699978	circRNA_012585	13:144064121|144085795	circRNA_009681	6:101697609|101804423
circRNA_005325	X:21215703|21222133	circRNA_003890	2:20494547|20498407	circRNA_018423	1:80283905|80305582	circRNA_020765	15:146428834|146535869
circRNA_000614	13:87134378|87140810	circRNA_011357	14:113577307|113581167	circRNA_007352	17:37028021|37049702	circRNA_014668	15:156203226|156310381
circRNA_009617	6:23746830|23753262	circRNA_014461	5:92111058|92114918	circRNA_005927	17:43088897|43110584	circRNA_020854	2:3002611|3109853
circRNA_019287	9:118222774|118229206	circRNA_000211	1:230999159|231003021	circRNA_001748	5:76369649|76391339	circRNA_021075	5:90514781|90622092
circRNA_019570	15:103655146|103661579	circRNA_013707	2:118336336|118340199	circRNA_001220	2:12191539|12213233	circRNA_003084	13:28201059|28308425
circRNA_011192	13:1164933|1171370	circRNA_005850	15:91619841|91623705	circRNA_016068	4:55272747|55294453	circRNA_006848	10:62681670|62789150
circRNA_007664	4:72806099|72812537	circRNA_008083	9:13255481|13259345	circRNA_008007	8:44869888|44891596	circRNA_016902	15:85340631|85448116
circRNA_012573	13:104777134|104783572	circRNA_010260	10:15988400|15992267	circRNA_010637	3:114064239|114085951	circRNA_005386	X:124531921|124639457
circRNA_003295	14:10459275|10465714	circRNA_008760	13:56340265|56344133	circRNA_001423	3:54939544|54961257	circRNA_020074	16:20241801|20349408
circRNA_013390	8:75763752|75770191	circRNA_012822	16:77782286|77786154	circRNA_000022	1:17240890|17262608	circRNA_001392	3:19817687|19925311
circRNA_006958	13:2387443|2393883	circRNA_014558	9:79218044|79221912	circRNA_013765	1:21200690|21222409	circRNA_004141	3:92379321|92486956
circRNA_012459	12:1651399|1657844	circRNA_004148	3:99288249|99292119	circRNA_005647	13:24055472|24077196	circRNA_017509	1:306809216|306916943
circRNA_010924	JH118638.1:314870|321317	circRNA_009324	3:10258449|10262321	circRNA_021011	4:48134546|48156272	circRNA_012473	12:16978073|17085916
circRNA_019185	7:100823334|100829781	circRNA_004604	6:88476536|88480409	circRNA_010570	2:52847733|52869467	circRNA_020143	3:62972768|63080743
circRNA_017156	5:106572787|106579236	circRNA_009343	3:38788790|38792663	circRNA_007661	4:71185799|71207535	circRNA_013924	5:50724811|50832839

<table>
<tr><td>circRNA_006903</td><td>12:4631315|4637765</td><td>circRNA_015316</td><td>1:304617890|304621764</td><td>circRNA_009470</td><td>4:72832383|72854126</td><td>circRNA_016061</td><td>4:39835278|39943326</td></tr>
<tr><td>circRNA_020730</td><td>15:52290912|52297362</td><td>circRNA_008176</td><td>9:138776634|138780511</td><td>circRNA_011699</td><td>3:76466177|76487931</td><td>circRNA_014269</td><td>14:146937329|147045391</td></tr>
<tr><td>circRNA_012988</td><td>3:359600|366053</td><td>circRNA_011662</td><td>3:19058493|19062370</td><td>circRNA_002413</td><td>X:17758636|17780393</td><td>circRNA_019342</td><td>X:60915080|61023192</td></tr>
<tr><td>circRNA_015636</td><td>14:77581776|77588230</td><td>circRNA_013016</td><td>3:62315178|62319055</td><td>circRNA_006470</td><td>9:40532481|40554238</td><td>circRNA_001678</td><td>5:3524350|3632541</td></tr>
<tr><td>circRNA_007454</td><td>2:69322500|69328955</td><td>circRNA_020343</td><td>GL894711.1:25655|29532</td><td>circRNA_018552</td><td>10:50701320|50723087</td><td>circRNA_007712</td><td>5:3524337|3632541</td></tr>
<tr><td>circRNA_020809</td><td>17:33077647|33084103</td><td>circRNA_007252</td><td>15:79516524|79520402</td><td>circRNA_007246</td><td>15:73524173|73545942</td><td>circRNA_004922</td><td>8:50518546|50626858</td></tr>
<tr><td>circRNA_017349</td><td>9:126535284|126540927</td><td>circRNA_009515</td><td>5:4515920|4519798</td><td>circRNA_011017</td><td>1:152305080|152326850</td><td>circRNA_010911</td><td>9:146291801|146400265</td></tr>
<tr><td>circRNA_002190</td><td>9:11795661|11801305</td><td>circRNA_003372</td><td>14:71918903|71922782</td><td>circRNA_012144</td><td>JH118921.1:9922|31692</td><td>circRNA_001396</td><td>3:29618701|29727215</td></tr>
<tr><td>circRNA_014753</td><td>X:21174276|21179921</td><td>circRNA_003732</td><td>16:71385817|71389696</td><td>circRNA_017041</td><td>2:112497186|112518956</td><td>circRNA_013431</td><td>9:1073464|1182009</td></tr>
<tr><td>circRNA_010367</td><td>13:42372497|42378144</td><td>circRNA_003746</td><td>17:13088600|13092479</td><td>circRNA_003226</td><td>13:147189066|147210843</td><td>circRNA_020241</td><td>6:143706339|143814929</td></tr>
<tr><td>circRNA_015330</td><td>10:29336781|29342429</td><td>circRNA_005520</td><td>1:283782371|283786252</td><td>circRNA_011945</td><td>7:57967998|57989778</td><td>circRNA_005568</td><td>10:63257104|63365702</td></tr>
<tr><td>circRNA_016524</td><td>GL893953.2:109824|115475</td><td>circRNA_011438</td><td>15:118051711|118055593</td><td>circRNA_020609</td><td>13:90928664|90950445</td><td>circRNA_009701</td><td>6:141868567|141977240</td></tr>
<tr><td>circRNA_001163</td><td>18:16661859|16667511</td><td>circRNA_012146</td><td>JH118963.1:65227|69109</td><td>circRNA_005239</td><td>GL894022.1:9043|30826</td><td>circRNA_015174</td><td>1:63620358|63729178</td></tr>
<tr><td>circRNA_011478</td><td>16:27950319|27955971</td><td>circRNA_014417</td><td>4:43514475|43518358</td><td>circRNA_005069</td><td>9:19215967|19237756</td><td>circRNA_019151</td><td>7:18392249|18501121</td></tr>
<tr><td>circRNA_013539</td><td>GL895152.1:21004|26657</td><td>circRNA_014441</td><td>5:3454359|3458242</td><td>circRNA_013051</td><td>3:121691230|121713024</td><td>circRNA_007334</td><td>17:1250517|1359481</td></tr>
<tr><td>circRNA_003582</td><td>15:92905984|92911641</td><td>circRNA_002100</td><td>8:76896146|76900030</td><td>circRNA_015838</td><td>18:40637737|40659540</td><td>circRNA_015312</td><td>1:297336189|297445171</td></tr>
<tr><td>circRNA_005319</td><td>X:17526788|17532445</td><td>circRNA_003221</td><td>13:144391256|144395141</td><td>circRNA_005420</td><td>1:45632972|45654779</td><td>circRNA_020003</td><td>13:82197957|82306978</td></tr>
<tr><td>circRNA_019610</td><td>18:44784420|44790077</td><td>circRNA_014075</td><td>5:90618207|90622092</td><td>circRNA_003033</td><td>12:45475763|45497578</td><td>circRNA_003839</td><td>18:25511592|25620626</td></tr>
<tr><td>circRNA_015891</td><td>2:80553499|80559157</td><td>circRNA_002264</td><td>9:91546721|91550607</td><td>circRNA_002037</td><td>7:119957967|119979791</td><td>circRNA_012552</td><td>13:76738802|76847836</td></tr>
<tr><td>circRNA_000164</td><td>1:182854630|182860290</td><td>circRNA_007351</td><td>17:36500876|36504762</td><td>circRNA_015247</td><td>1:206446036|206467864</td><td>circRNA_000198</td><td>1:216338622|216447657</td></tr>
<tr><td>circRNA_018658</td><td>13:130082229|130087890</td><td>circRNA_010531</td><td>17:59227820|59231706</td><td>circRNA_020635</td><td>13:148316595|148338426</td><td>circRNA_004400</td><td>5:3524350|3633443</td></tr>
<tr><td>circRNA_005151</td><td>9:102608469|102614132</td><td>circRNA_011190</td><td>13:1123404|1127291</td><td>circRNA_015473</td><td>13:4870091|4891923</td><td>circRNA_007330</td><td>16:57092936|57202262</td></tr>
<tr><td>circRNA_017233</td><td>7:87588710|87594373</td><td>circRNA_011866</td><td>6:23746830|23750717</td><td>circRNA_013573</td><td>X:35456706|35478544</td><td>circRNA_017406</td><td>X:121503771|121613124</td></tr>
<tr><td>circRNA_003137</td><td>13:72822976|72828640</td><td>circRNA_006343</td><td>7:72233626|72237514</td><td>circRNA_005023</td><td>8:141457586|141479430</td><td>circRNA_020126</td><td>2:137522550|137631960</td></tr>
<tr><td>circRNA_008194</td><td>GL893560.1:9170|14835</td><td>circRNA_017952</td><td>8:46580336|46584225</td><td>circRNA_018183</td><td>15:106841281|106863127</td><td>circRNA_012347</td><td>10:18101700|18211136</td></tr>
<tr><td>circRNA_020427</td><td>1:176840711|176846376</td><td>circRNA_011317</td><td>14:31377807|31381697</td><td>circRNA_017771</td><td>2:119262333|119284184</td><td>circRNA_006312</td><td>6:148924023|149033483</td></tr>
<tr><td>circRNA_009559</td><td>5:70074785|70080451</td><td>circRNA_007291</td><td>15:139207889|139211780</td><td>circRNA_017060</td><td>3:19058493|19080345</td><td>circRNA_000723</td><td>13:218430260|218539731</td></tr>
<tr><td>circRNA_012417</td><td>11:4500847|4506513</td><td>circRNA_016802</td><td>13:87172364|87176256</td><td>circRNA_017976</td><td>9:8928600|8950452</td><td>circRNA_005959</td><td>18:59442183|59551671</td></tr>
<tr><td>circRNA_013575</td><td>X:43149391|43155057</td><td>circRNA_014762</td><td>1:125491718|125495611</td><td>circRNA_020893</td><td>2:86089976|86111828</td><td>circRNA_001911</td><td>6:139872924|139982486</td></tr>
<tr><td>circRNA_008711</td><td>13:14154446|14160113</td><td>circRNA_005937</td><td>18:5554189|5558083</td><td>circRNA_017149</td><td>5:76369482|76391339</td><td>circRNA_009929</td><td>9:23052452|23162184</td></tr>
<tr><td>circRNA_012563</td><td>13:90919603|90925273</td><td>circRNA_016438</td><td>9:24373378|24377275</td><td>circRNA_013388</td><td>8:69661028|69682886</td><td>circRNA_015270</td><td>1:229186577|229296319</td></tr>
<tr><td>circRNA_012172</td><td>X:127709214|127714885</td><td>circRNA_020449</td><td>1:202655424|202659321</td><td>circRNA_017441</td><td>1:101205442|101227302</td><td>circRNA_018366</td><td>9:25250548|25360320</td></tr>
<tr><td>circRNA_015244</td><td>1:205265275|205270946</td><td>circRNA_008500</td><td>1:299974420|299978323</td><td>circRNA_006670</td><td>1:110526474|110548336</td><td>circRNA_018508</td><td>1:249753770|249863571</td></tr>
<tr><td>circRNA_012692</td><td>14:125000335|125006008</td><td>circRNA_001532</td><td>3:134163529|134167433</td><td>circRNA_016140</td><td>5:38558578|38580442</td><td>circRNA_015232</td><td>1:195020817|195130686</td></tr>
<tr><td>circRNA_017431</td><td>1:59750078|59755752</td><td>circRNA_010160</td><td>X:127737964|127741869</td><td>circRNA_010069</td><td>GL894927.1:10287|32157</td><td>circRNA_006408</td><td>8:74346358|74456314</td></tr>
<tr><td>circRNA_014940</td><td>5:61454150|61459831</td><td>circRNA_020331</td><td>9:115605241|115609146</td><td>circRNA_000305</td><td>10:29342271|29364146</td><td>circRNA_018378</td><td>9:146290018|146400026</td></tr>
<tr><td>circRNA_020258</td><td>7:48254935|48260616</td><td>circRNA_004872</td><td>8:1266332|1270239</td><td>circRNA_015482</td><td>13:21193239|21215121</td><td>circRNA_010851</td><td>8:124119181|124229272</td></tr>
<tr><td>circRNA_011885</td><td>6:81551069|81556752</td><td>circRNA_016473</td><td>9:70879781|70883688</td><td>circRNA_003490</td><td>15:8548841|8570725</td><td>circRNA_010239</td><td>1:229233921|229344099</td></tr>
<tr><td>circRNA_010014</td><td>9:127878075|127883759</td><td>circRNA_017064</td><td>3:42811731|42815639</td><td>circRNA_012706</td><td>15:35923327|35945213</td><td>circRNA_020293</td><td>8:82954008|83064254</td></tr>
<tr><td>circRNA_009456</td><td>4:48134546|48140232</td><td>circRNA_017299</td><td>8:139708449|139712357</td><td>circRNA_011195</td><td>13:4752268|4774160</td><td>circRNA_002311</td><td>9:146290018|146400265</td></tr>
<tr><td>circRNA_010257</td><td>1:307315009|307320696</td><td>circRNA_020573</td><td>13:14244640|14248548</td><td>circRNA_013012</td><td>3:58181677|58203572</td><td>circRNA_000515</td><td>12:60084897|60195211</td></tr>
<tr><td>circRNA_014797</td><td>15:3788416|3794104</td><td>circRNA_007294</td><td>15:146201175|146205084</td><td>circRNA_012770</td><td>15:120872321|120894217</td><td>circRNA_009900</td><td>8:136277085|136387565</td></tr>
<tr><td>circRNA_003797</td><td>17:65770875|65776565</td><td>circRNA_007817</td><td>6:57230731|57234640</td><td>circRNA_018630</td><td>13:42295832|42317731</td><td>circRNA_001123</td><td>17:52872255|52982857</td></tr>
<tr><td>circRNA_013612</td><td>3:60832497|60838187</td><td>circRNA_008806</td><td>13:108510400|108514309</td><td>circRNA_000777</td><td>14:52849487|52871390</td><td>circRNA_000016</td><td>1:16779138|16889979</td></tr>
<tr><td>circRNA_001051</td><td>16:36742926|36748618</td><td>circRNA_015267</td><td>1:227405860|227409769</td><td>circRNA_014418</td><td>4:45545828|45567734</td><td>circRNA_005641</td><td>12:61481535|61592432</td></tr>
<tr><td>circRNA_008163</td><td>9:127753225|127758917</td><td>circRNA_018072</td><td>1:50514833|50518742</td><td>circRNA_011287</td><td>13:205647586|205669502</td><td>circRNA_019467</td><td>12:61481535|61592576</td></tr>
<tr><td>circRNA_004224</td><td>4:2021944|2027637</td><td>circRNA_013848</td><td>15:90260328|90264238</td><td>circRNA_012081</td><td>9:116123437|116145363</td><td>circRNA_020695</td><td>14:81282853|81394088</td></tr>
<tr><td>circRNA_017951</td><td>8:35200345|35206039</td><td>circRNA_019664</td><td>3:25673100|25677010</td><td>circRNA_012468</td><td>12:16041804|16063733</td><td>circRNA_004381</td><td>4:136249373|136360665</td></tr>
<tr><td>circRNA_012379</td><td>10:53370958|53376653</td><td>circRNA_000052</td><td>1:50514830|50518742</td><td>circRNA_005185</td><td>9:134281948|134303881</td><td>circRNA_000220</td><td>1:248529181|248640482</td></tr>
<tr><td>circRNA_005356</td><td>X:85916081|85921778</td><td>circRNA_001259</td><td>2:60728540|60732452</td><td>circRNA_005668</td><td>13:42298387|42320321</td><td>circRNA_005743</td><td>13:198955614|199067054</td></tr>
<tr><td>circRNA_018126</td><td>13:42295832|42301529</td><td>circRNA_003645</td><td>15:137963814|137967726</td><td>circRNA_017642</td><td>14:85854168|85876103</td><td>circRNA_007127</td><td>14:52135828|52247318</td></tr>
<tr><td>circRNA_004972</td><td>8:89359060|89364758</td><td>circRNA_017362</td><td>GL893669.1:73398|77310</td><td>circRNA_011775</td><td>4:73760914|73782852</td><td>circRNA_005991</td><td>2:78571331|78682981</td></tr>
</table>

circRNA_015545	13:131172419|131178117	circRNA_019118	6:101468757|101472669	circRNA_011096	10:29515051|29536990	circRNA_007658	4:67851823|67963521
circRNA_016644	1:124969291|124974989	circRNA_019795	7:42039235|42043148	circRNA_002015	7:94511177|94533117	circRNA_015647	14:83544233|83656052
circRNA_019809	7:106571291|106576989	circRNA_008927	14:78268555|78272470	circRNA_011906	6:132195677|132217626	circRNA_003887	2:16383642|16495559
circRNA_009015	15:66100572|66106274	circRNA_005930	17:45160772|45164688	circRNA_017786	3:33356500|33378455	circRNA_011246	13:102212196|102324338
circRNA_011056	1:257756332|257762035	circRNA_014907	1:25536401|25540318	circRNA_021016	4:73577490|73599445	circRNA_004757	7:38746540|38858691
circRNA_013622	1:282190279|282195982	circRNA_018699	14:9473581|9477499	circRNA_007104	14:25744381|25766337	circRNA_002130	8:118153294|118265460
circRNA_013506	9:144811158|144816862	circRNA_014546	9:19334549|19338468	circRNA_000897	15:28808655|28830613	circRNA_005755	14:418144|530434
circRNA_004580	6:79809424|79815135	circRNA_019674	3:72414222|72418141	circRNA_005191	9:138832114|138854073	circRNA_011754	4:35844936|35957356
circRNA_002378	GL896471.1:3244|8966	circRNA_005692	13:82208452|82212373	circRNA_018099	1:283798123|283820084	circRNA_000270	1:306822439|306934873
circRNA_003113	13:42386578|42392300	circRNA_009835	8:33351020|33354941	circRNA_015273	1:230890866|230912836	circRNA_008419	1:195020817|195133308
circRNA_006515	GL892421.2:51224|56946	circRNA_013214	5:109537953|109541874	circRNA_004722	7:14649628|14671601	circRNA_021205	8:71895225|72007844
circRNA_002570	1:127622340|127628063	circRNA_021299	GL892550.1:11977|15898	circRNA_008888	14:32141473|32163450	circRNA_010950	1:13973894|14086548
circRNA_008651	12:22314210|22319934	circRNA_016488	9:101476729|101480651	circRNA_016201	6:32224765|32246744	circRNA_005925	17:40347131|40459796
circRNA_004794	7:69490627|69496352	circRNA_017124	4:116151405|116155329	circRNA_005763	14:13378699|13400680	circRNA_000356	10:63252929|63365702
circRNA_007533	3:11566431|11572160	circRNA_005995	2:82153520|82157445	circRNA_020746	15:85355707|85377689	circRNA_020306	9:21273530|21386369
circRNA_021215	8:89838198|89843927	circRNA_009087	16:25422928|25426853	circRNA_012946	2:84438188|84460171	circRNA_012537	13:44863307|44976165
circRNA_016230	6:86659126|86664856	circRNA_004893	8:30564837|30568764	circRNA_021057	5:63631299|63653282	circRNA_013396	8:83030549|83143453
circRNA_010128	X:68413010|68418741	circRNA_015769	16:51611666|51615593	circRNA_004353	4:122750450|122772434	circRNA_011966	7:101076419|101189459
circRNA_020397	1:103209533|103215268	circRNA_009716	6:148615305|148619233	circRNA_011954	7:73042261|73064245	circRNA_011038	1:216338622|216451723
circRNA_017683	15:106832344|106838081	circRNA_002562	1:123398065|123401996	circRNA_000315	10:35020852|35042842	circRNA_011039	1:216338622|216451726
circRNA_000428	11:70434369|70440107	circRNA_004987	8:103899707|103903638	circRNA_000260	1:283819938|283841933	circRNA_001164	18:18602505|18715721
circRNA_006705	1:154682795|154688535	circRNA_020881	2:65359607|65363538	circRNA_007311	16:36676971|36698971	circRNA_017440	1:86117059|86230282
circRNA_008575	11:317743|323483	circRNA_007219	15:28837231|28841166	circRNA_012447	11:72374926|72396926	circRNA_001679	5:3524350|3637671
circRNA_000637	13:117332280|117338022	circRNA_006838	10:50698422|50702358	circRNA_006465	9:36645265|36667272	circRNA_001001	15:118142455|118255877
circRNA_001745	5:73514778|73520522	circRNA_000788	14:60179346|60183283	circRNA_007445	2:52827621|52849629	circRNA_011439	15:118142455|118255893
circRNA_006963	13:8092764|8098509	circRNA_001957	7:33109626|33113564	circRNA_003579	15:89439897|89461914	circRNA_015686	15:36264244|36377697
circRNA_008703	13:3107945|3113691	circRNA_011211	13:35643826|35647767	circRNA_001774	5:111001237|111023271	circRNA_016703	1:299301119|299414632
circRNA_012037	9:7103733|7109479	circRNA_013968	GL892430.1:58116|62058	circRNA_011812	5:38580339|38602389	circRNA_000602	13:76734202|76847836
circRNA_008498	1:298098190|298103937	circRNA_009933	9:31874975|31878919	circRNA_002732	1:267668334|267690386	circRNA_018877	18:36649587|36763439
circRNA_009642	6:66688637|66694384	circRNA_016028	3:119656395|119660341	circRNA_018185	15:115183250|115205304	circRNA_014998	14:136774429|136888438
circRNA_008289	1:8838522|8844270	circRNA_019147	7:14114606|14118552	circRNA_012455	12:301659|323714	circRNA_018279	5:3524350|3638368
circRNA_007286	15:133589366|133595116	circRNA_009133	17:13663699|13667649	circRNA_004366	4:129522757|129544827	circRNA_016981	18:18854092|18968216
circRNA_001953	7:27922038|27927789	circRNA_015084	13:26034153|26038103	circRNA_008027	8:80764338|80786409	circRNA_007899	7:16992242|17106510
circRNA_005038	9:39472|45223	circRNA_012181	1:16112729|16116681	circRNA_015544	13:130118894|130140966	circRNA_014338	17:67365430|67479794
circRNA_010455	14:123996752|124002504	circRNA_004320	4:98051660|98055613	circRNA_010729	6:13071374|13093451	circRNA_021149	7:16923592|17037985
circRNA_011368	14:120739490|120745242	circRNA_000582	13:42388345|42392300	circRNA_006862	11:6780496|6802586	circRNA_000350	10:58798085|58912493
circRNA_013830	13:202648396|202654149	circRNA_006996	13:52502787|52506744	circRNA_020158	3:130566262|130588353	circRNA_010843	8:83358407|83472898
circRNA_018146	13:212957466|212963222	circRNA_007416	2:3016339|3020299	circRNA_012540	13:50636313|50658407	circRNA_010933	X:60908671|61023192
circRNA_010565	2:29219247|29225004	circRNA_020644	13:158559403|158563363	circRNA_004055	3:11566431|11588535	circRNA_020107	18:59806892|59921726
circRNA_019086	5:93484744|93490501	circRNA_009550	5:57061408|57065369	circRNA_012853	17:65833013|65855120	circRNA_008999	15:53022089|53136929
circRNA_000736	14:13400448|13406208	circRNA_012271	1:161054128|161058089	circRNA_004762	7:46357417|46379549	circRNA_012701	14:142735102|142850048
circRNA_018199	17:42873824|42879589	circRNA_017760	2:74939891|74943853	circRNA_012186	1:21209786|21231923	circRNA_019253	9:29312607|29427613
circRNA_007799	6:30402013|30407779	circRNA_013202	5:92494556|92498519	circRNA_014061	GL893259.1:34612|56752	circRNA_002566	1:125384211|125499226
circRNA_000245	1:275800353|275806120	circRNA_012092	9:138813342|138817306	circRNA_016808	13:104350793|104372935	circRNA_014137	1:229131344|229246387
circRNA_001298	2:87512163|87517930	circRNA_005653	13:28570512|28574477	circRNA_002198	9:19264985|19287128	circRNA_015728	15:122363114|122478331
circRNA_013890	2:125045156|125050923	circRNA_008535	10:38205877|38209843	circRNA_013733	9:11779155|11801305	circRNA_011871	6:47675862|47791083
circRNA_002795	10:29531220|29536990	circRNA_010383	13:90946479|90950445	circRNA_003483	14:148219514|148241667	circRNA_011870	6:47675856|47791083
circRNA_011087	10:18659807|18665581	circRNA_012470	12:16864048|16868014	circRNA_000317	10:35240929|35263088	circRNA_014754	X:51122704|51238066
circRNA_004234	4:16723581|16729356	circRNA_005244	GL894350.2:26486|30453	circRNA_012265	1:145408743|145430902	circRNA_015021	18:59442183|59557766
circRNA_011333	14:58796714|58802491	circRNA_020957	3:53685299|53689266	circRNA_011983	7:134300825|134322985	circRNA_011751	4:34502003|34617596
circRNA_014686	2:121549319|121555096	circRNA_003171	13:91072247|91076217	circRNA_015615	14:57864008|57886171	circRNA_001680	5:3524350|3640010
circRNA_014990	13:192362571|192368348	circRNA_009557	5:65034546|65038516	circRNA_010617	3:47791184|47813349	circRNA_010189	1:83687836|83803523

circRNA_005665	13:35737581|35743359	circRNA_002285	9:126101169|126105141	circRNA_014777	12:38779681|38801846	circRNA_001323	2:118846107|118961796
circRNA_005439	1:110532620|110538400	circRNA_010652	4:30239675|30243647	circRNA_004692	6:147045770|147067936	circRNA_000348	10:58766088|58881810
circRNA_004009	2:128799479|128805266	circRNA_003027	12:45332161|45336134	circRNA_021381	X:109514540|109536709	circRNA_002844	10:58912390|59028119
circRNA_005049	9:8944659|8950452	circRNA_014157	10:45908068|45912041	circRNA_003956	2:87101409|87123585	circRNA_009853	8:67267695|67383579
circRNA_001662	4:134785876|134791670	circRNA_001718	5:54725893|54729869	circRNA_009842	8:43018351|43040527	circRNA_001873	6:101697609|101813590
circRNA_013366	8:110687|116481	circRNA_008853	13:205655759|205659736	circRNA_020739	15:74127283|74149480	circRNA_001831	6:82232928|82348935
circRNA_017984	9:36273393|36279187	circRNA_011279	13:160856562|160860539	circRNA_008309	1:40745355|40767556	circRNA_003252	13:198955614|199071690
circRNA_008969	14:141071069|141076866	circRNA_004370	4:129639341|129643320	circRNA_001800	6:48344625|48366827	circRNA_020239	6:139883957|140000113
circRNA_015748	16:23643977|23649775	circRNA_006472	9:40572085|40576066	circRNA_000479	12:37398861|37421064	circRNA_009891	8:124800369|124916536
circRNA_020923	2:142628762|142634560	circRNA_009354	3:46250087|46254068	circRNA_007669	4:77292991|77315196	circRNA_002052	8:1651183|1767388
circRNA_003213	13:143278146|143283946	circRNA_010874	9:40613311|40617293	circRNA_000621	13:97282134|97304344	circRNA_018637	13:61839615|61956028
circRNA_014838	4:117897403|117903206	circRNA_006867	11:18975972|18979956	circRNA_003041	12:49414058|49436273	circRNA_008144	9:98631547|98748026
circRNA_011346	14:82341879|82347683	circRNA_006703	1:154649289|154653274	circRNA_000412	11:35045323|35067542	circRNA_020500	10:18033408|18149975
circRNA_010916	GL894339.1:49150|54958	circRNA_017401	X:109532724|109536709	circRNA_013868	17:41996687|42018906	circRNA_006449	9:12135530|12252146
circRNA_007561	3:62627636|62633446	circRNA_019301	GL892541.1:27382|31367	circRNA_018441	1:127648623|127670842	circRNA_009656	6:82232928|82349654
circRNA_000926	15:66307639|66313450	circRNA_007653	4:55364472|55368458	circRNA_005399	1:4635856|4658081	circRNA_011688	3:66450790|66567718
circRNA_003993	2:118941644|118947455	circRNA_010864	9:13245753|13249740	circRNA_006864	11:11363633|11385864	circRNA_009988	9:88615508|88732539
circRNA_011993	8:32728074|32733886	circRNA_015187	1:94542249|94546236	circRNA_016222	6:73334893|73357138	circRNA_002102	8:78303121|78420195
circRNA_001684	5:4617234|4623047	circRNA_005186	9:136556072|136560060	circRNA_002365	GL895718.2:21463|43709	circRNA_005961	18:59804518|59921726
circRNA_002103	8:78476244|78482057	circRNA_002640	1:188117293|188121283	circRNA_000740	14:14155674|14177923	circRNA_016885	15:8169168|8286604
circRNA_003691	16:37494597|37500411	circRNA_000018	1:17087947|17091938	circRNA_013400	8:85952805|85975055	circRNA_002131	8:118153294|118270866
circRNA_008530	10:35389780|35395594	circRNA_007947	7:99001662|99005656	circRNA_009369	3:63149385|63171638	circRNA_008002	8:40572430|40690111
circRNA_014891	9:138721213|138727029	circRNA_015121	14:116886125|116890119	circRNA_017896	6:85428674|85450927	circRNA_020456	1:225691245|225808956
circRNA_019587	16:51317149|51322965	circRNA_007977	8:97513|101509	circRNA_004409	5:14247237|14269491	circRNA_011784	4:101569868|101687692
circRNA_011491	16:47823935|47829752	circRNA_000272	1:311343239|311347236	circRNA_007433	2:41866547|41888804	circRNA_016083	4:73977625|74095485
circRNA_017499	1:248853148|248858965	circRNA_019729	5:52435212|52439209	circRNA_020811	17:43208017|43230275	circRNA_005138	9:81987561|82105500
circRNA_003523	15:55066576|55072396	circRNA_012163	X:102838373|102842371	circRNA_005873	15:122439223|122461484	circRNA_010314	11:24688121|24806062
circRNA_006521	GL893520.1:14693|20513	circRNA_002856	10:63471347|63475346	circRNA_020458	1:227166226|227188489	circRNA_020572	13:4752268|4870221
circRNA_006200	5:38575947|38581769	circRNA_013605	13:44104348|44108347	circRNA_015426	12:14814795|14837065	circRNA_002806	10:35717098|35835067
circRNA_011772	4:68358494|68364318	circRNA_020546	12:18786985|18790985	circRNA_019638	2:104729953|104752223	circRNA_001269	2:72958959|73076955
circRNA_015782	16:69012237|69018061	circRNA_021380	X:109093199|109097201	circRNA_010606	3:10470262|10492534	circRNA_000293	10:18101700|18219757
circRNA_019365	1:55626102|55631926	circRNA_006906	12:5063509|5067513	circRNA_018941	2:121383217|121405489	circRNA_006029	2:118253397|118371589
circRNA_020393	1:87065801|87071625	circRNA_007024	13:90959930|90963935	circRNA_005016	8:140909641|140931918	circRNA_011552	18:44199254|44317450
circRNA_008875	14:12647694|12653521	circRNA_002759	1:299554717|299558723	circRNA_011883	6:78476142|78498419	circRNA_019687	3:118042427|118160655
circRNA_008619	11:70173370|70179198	circRNA_006510	9:138768104|138772110	circRNA_005241	GL894261.2:68764|91044	circRNA_015505	13:44857853|44976165
circRNA_001096	17:29783317|29789147	circRNA_001740	5:71388114|71392121	circRNA_008376	1:134628972|134651252	circRNA_019429	10:53011741|53130056
circRNA_004035	2:147048493|147054323	circRNA_016568	JH118963.1:56856|60866	circRNA_000250	1:278917340|278939621	circRNA_015360	10:58909674|59028119
circRNA_017658	15:18564240|18570071	circRNA_017582	13:18743779|18747789	circRNA_002940	11:75110052|75132336	circRNA_006294	6:113003755|113122204
circRNA_019368	1:82008874|82014706	circRNA_009416	3:125919196|125923207	circRNA_006794	1:299554717|299577014	circRNA_010587	2:118253397|118371870
circRNA_001643	4:121741759|121747592	circRNA_021265	9:49802705|49806716	circRNA_008343	1:101036384|101058690	circRNA_007331	16:72618407|72736948
circRNA_010148	X:106113721|106119554	circRNA_001695	5:23680116|23684128	circRNA_009244	2:50783412|50805720	circRNA_019886	X:30975313|31093895
circRNA_000789	14:60200334|60206170	circRNA_006666	1:107929920|107933933	circRNA_001664	4:136390669|136412978	circRNA_014520	8:30952024|31070799
circRNA_006803	10:10312706|10318544	circRNA_007828	6:69990483|69994497	circRNA_007842	6:92070354|92092664	circRNA_016835	14:18906641|19025509
circRNA_010921	GL896344.1:8222|14062	circRNA_015336	10:34280932|34284947	circRNA_012583	13:132319701|132342011	circRNA_007443	2:52797534|52916460
circRNA_009263	2:83180615|83186456	circRNA_000175	1:202012272|202016291	circRNA_012879	18:41980502|42002812	circRNA_012531	13:43001666|43120627
circRNA_009830	8:32689706|32695547	circRNA_000302	10:29321805|29325824	circRNA_015784	16:77938945|77961255	circRNA_019572	15:106250222|106369211
circRNA_013792	10:17369643|17375485	circRNA_000384	11:12807920|12811939	circRNA_007516	2:142522073|142544386	circRNA_002081	8:40572430|40691447
circRNA_019471	13:25332051|25337895	circRNA_001320	2:112627933|112631954	circRNA_014273	15:18567544|18589857	circRNA_019560	15:8167430|8286604
circRNA_004401	5:3528356|3534201	circRNA_009775	7:73295407|73299428	circRNA_018465	1:176374364|176396678	circRNA_008972	14:142735102|142854338
circRNA_009334	3:19646832|19652677	circRNA_014808	17:53655378|53659399	circRNA_018980	3:74739616|74761931	circRNA_010375	13:75071541|75190913
circRNA_000283	10:14918794|14924640	circRNA_016422	9:16686|20707	circRNA_020029	14:21553858|21576174	circRNA_021369	X:60915080|61034602
circRNA_018477	1:185076768|185082615	circRNA_018494	1:216017056|216021077	circRNA_003485	15:3788416|3810739	circRNA_000672	13:144478832|144598460

circRNA_018710	14:31061409|31067257	circRNA_000480	12:37425385|37429409	circRNA_009311	2:142522062|142544386	circRNA_012311	1:254817267|254937077
circRNA_001510	3:120136072|120141921	circRNA_004305	4:88603967|88607994	circRNA_001950	7:23927741|23950067	circRNA_019093	6:32224765|32344661
circRNA_020112	2:12807112|12812963	circRNA_018572	11:18580300|18584327	circRNA_007766	5:79676712|79699042	circRNA_001194	18:59442183|59562118
circRNA_008105	9:40552034|40557886	circRNA_009792	7:93704801|93708829	circRNA_002866	11:266572|288907	circRNA_002218	9:40746631|40866659
circRNA_009800	7:104147580|104153432	circRNA_009665	6:89994039|89998069	circRNA_001528	3:130629324|130651664	circRNA_015282	1:244316290|244436380
circRNA_013300	7:9031840|9037692	circRNA_003896	2:35603576|35607609	circRNA_004630	6:99525347|99547687	circRNA_012426	11:14717311|14837488
circRNA_020992	3:122014186|122020038	circRNA_002244	9:72670797|72674834	circRNA_000969	15:93043493|93065848	circRNA_007212	15:8003860|8124108
circRNA_014542	9:13285632|13291486	circRNA_011538	18:12308719|12312757	circRNA_020925	2:145993266|146015631	circRNA_003488	15:8169168|8289420
circRNA_002812	10:37465102|37470957	circRNA_014602	X:112711247|112715285	circRNA_007465	2:79596775|79619142	circRNA_005595	11:25953241|26073707
circRNA_004333	4:107938850|107944705	circRNA_000638	13:117567899|117571938	circRNA_014648	13:190918409|190940782	circRNA_016673	1:204823927|204944457
circRNA_020738	15:73581814|73587669	circRNA_010523	17:29789957|29793996	circRNA_017578	12:53676699|53699076	circRNA_015114	1:225691245|225811866
circRNA_006737	1:202605803|202611659	circRNA_018652	13:97310831|97314870	circRNA_009428	4:2602892|2625276	circRNA_000390	11:14714358|14835206
circRNA_006420	8:88158147|88164007	circRNA_012598	13:166847412|166851452	circRNA_002821	10:47064149|47086534	circRNA_006274	6:82227996|82348935
circRNA_001114	17:45685173|45691034	circRNA_015502	13:42831648|42835690	circRNA_013712	3:122008499|122030888	circRNA_019775	6:143955826|144076909
circRNA_007691	4:121576595|121582456	circRNA_018722	14:42346799|42350841	circRNA_010269	10:18101700|18124093	circRNA_011667	3:24856143|24977247
circRNA_020763	15:124776531|124782394	circRNA_016088	4:91277960|91276003	circRNA_000753	14:30050907|30073303	circRNA_001794	6:42522912|42644185
circRNA_015504	13:44084999|44090863	circRNA_020852	18:60693248|60697291	circRNA_004914	8:41193695|41216093	circRNA_019998	13:43006670|43128005
circRNA_001915	6:143547653|143553519	circRNA_007827	6:69726120|69730166	circRNA_000367	10:76283712|76306111	circRNA_012376	10:53339997|53461410
circRNA_017295	8:134740929|134746802	circRNA_016784	13:35773486|35777533	circRNA_014779	13:4770593|4792993	circRNA_020198	5:49152457|49273921
circRNA_007551	3:48100930|48106804	circRNA_002633	1:181349907|181353955	circRNA_000184	1:202963753|202986161	circRNA_019062	5:31629973|31751484
circRNA_007026	13:91070338|91076217	circRNA_013159	5:32064191|32068240	circRNA_004105	3:73352412|73374822	circRNA_002456	X:144028110|144149638
circRNA_018787	15:75766309|75772189	circRNA_021122	6:99190172|99194221	circRNA_007844	6:94766483|94788895	circRNA_002460	1:10697954|10819507
circRNA_012875	18:27408621|27414502	circRNA_013958	9:29829631|29833681	circRNA_004938	8:58675796|58698211	circRNA_002139	8:124794853|124916536
circRNA_019313	GL894386.1:42299|48180	circRNA_006595	1:3256431|3260484	circRNA_005388	X:126963525|126985943	circRNA_013543	GL895864.2:56902|178678
circRNA_020862	2:27609102|27614983	circRNA_013075	4:16785783|16789839	circRNA_016513	GL892685.2:116779|139197	circRNA_017766	2:89865397|89987226
circRNA_009382	3:90658682|90664564	circRNA_008546	10:52907541|52911598	circRNA_013143	5:2668479|2690899	circRNA_015588	14:418144|540053
circRNA_004327	4:103267690|103273573	circRNA_008976	14:145330719|145334778	circRNA_001063	16:47778049|47800473	circRNA_000883	15:8167430|8289420
circRNA_007620	3:135162081|135167966	circRNA_015882	2:50799143|50803203	circRNA_012591	13:156196271|156218697	circRNA_013468	9:77935439|78057429
circRNA_013284	6:145415404|145421289	circRNA_018952	2:145725178|145729238	circRNA_019830	8:116894873|116917309	circRNA_016351	8:40601314|40723404
circRNA_016216	6:64512834|64518721	circRNA_010230	1:205002128|205006189	circRNA_021120	6:98777693|98800136	circRNA_003468	14:142735102|142857217
circRNA_017243	7:123584305|123590194	circRNA_003283	13:214811583|214815645	circRNA_007794	6:18424722|18447166	circRNA_001257	2:59939009|60061247
circRNA_005006	8:127091752|127097642	circRNA_010947	X:116392501|116396564	circRNA_002887	11:13202083|13224528	circRNA_018429	1:101723122|101845399
circRNA_012086	9:133341202|133347093	circRNA_020515	10:54209843|54213906	circRNA_014695	3:114326822|114349268	circRNA_020100	18:26314382|26436682
circRNA_003801	18:2767700|2773593	circRNA_000689	13:167100656|167104720	circRNA_007735	5:35894045|35916494	circRNA_018548	10:46597688|46720028
circRNA_004118	3:81726931|81732824	circRNA_019430	10:54054021|54058085	circRNA_013021	3:73352373|73374822	circRNA_005555	10:46597662|46720028
circRNA_005315	X:14300648|14306541	circRNA_009660	6:85275333|85279398	circRNA_015260	1:218551332|218573783	circRNA_014371	2:119165841|119288360
circRNA_000002	1:2808657|2814551	circRNA_007808	6:39555556|39559624	circRNA_016403	8:127051429|127073884	circRNA_020591	13:53761873|53884648
circRNA_007003	13:72897966|72903860	circRNA_008116	9:50889716|50893786	circRNA_014723	6:141363206|141385665	circRNA_012301	1:229173474|229296319
circRNA_012116	GL893873.1:50185|56079	circRNA_012636	14:34701542|34705614	circRNA_013352	7:109851808|109874274	circRNA_001270	2:72958959|73081910
circRNA_005676	13:55544176|55550074	circRNA_014048	3:38764978|38769050	circRNA_015253	1:212196049|212218515	circRNA_001564	4:40501976|40624960
circRNA_010723	5:92464571|92470469	circRNA_011304	14:10542537|10546610	circRNA_010517	17:5159655|5182123	circRNA_013086	4:40501973|40624960
circRNA_001042	16:24989252|24995151	circRNA_009123	16:78181186|78185260	circRNA_001987	7:67564824|67587294	circRNA_008585	11:14714358|14837488
circRNA_011695	3:74772971|74778873	circRNA_010486	15:94327571|94331645	circRNA_010071	GL894982.1:22842|45312	circRNA_012530	13:42997356|43120627
circRNA_005913	17:6311352|6317255	circRNA_014485	7:4687223|4691297	circRNA_020888	2:83206943|83229420	circRNA_012363	10:37747971|37871409
circRNA_008507	10:11881512|11887415	circRNA_016764	12:37851010|37855085	circRNA_002921	11:52212325|52234803	circRNA_014979	11:84964946|85088442
circRNA_008591	11:17971238|17977141	circRNA_003212	13:143278146|143282223	circRNA_004088	3:54939544|54962024	circRNA_015628	14:66692910|66816589
circRNA_011531	17:59308383|59314288	circRNA_016847	14:57803787|57807865	circRNA_003375	14:77561976|77584462	circRNA_010264	10:17046557|17170362
circRNA_012351	10:24649203|24655110	circRNA_019259	9:40639762|40643840	circRNA_008664	12:37358205|37380695	circRNA_001951	7:24626858|24750730
circRNA_019520	14:10483485|10489394	circRNA_012176	1:253254|257333	circRNA_009678	6:99360832|99383323	circRNA_004731	7:24627308|24751226
circRNA_006077	3:61505088|61510998	circRNA_002531	1:87778133|87782213	circRNA_021255	9:36603896|36626387	circRNA_002916	11:48285840|48409967
circRNA_013881	2:50802485|50808396	circRNA_008385	1:145412858|145416938	circRNA_020406	1:127113152|127135644	circRNA_009127	17:1250517|1375056
circRNA_006760	1:242727958|242733870	circRNA_015421	12:13502996|13507076	circRNA_000299	10:25171043|25193546	circRNA_000134	1:142085957|142210683

circRNA_013551	JH118545.1:47146|53058	circRNA_018115	11:78473522|78477602	circRNA_008808	13:117558744|117581247	circRNA_006660	1:101723122|101848069
circRNA_020753	15:97981496|97987408	circRNA_001067	16:47900020|47904101	circRNA_007771	5:83045372|83067876	circRNA_004100	3:66325973|66450959
circRNA_008709	13:6091992|6097905	circRNA_004250	4:37102480|37106561	circRNA_011786	4:109324468|109346978	circRNA_005456	1:142085631|142210683
circRNA_010602	2:154050733|154056647	circRNA_000676	13:149623912|149627995	circRNA_004627	6:99236964|99259476	circRNA_020628	13:132258977|132384029
circRNA_015105	8:120767276|120773190	circRNA_002988	12:23993281|23997364	circRNA_004665	6:132260942|132283455	circRNA_019961	10:43816346|43941718
circRNA_015162	1:44467551|44473465	circRNA_018828	16:37507586|37511669	circRNA_013263	6:99075904|99098418	circRNA_018533	10:17976426|18101825
circRNA_009067	15:152336067|152341982	circRNA_000882	15:3858677|3862761	circRNA_017471	1:176738721|176761238	circRNA_006122	4:2565801|2691206
circRNA_017868	5:93446116|93452031	circRNA_012859	18:5973179|5977263	circRNA_005726	13:144058321|144080843	circRNA_020499	10:17998633|18124093
circRNA_010138	X:70848297|70854213	circRNA_021330	GL895417.2:33163|37248	circRNA_009715	6:148463817|148486340	circRNA_002711	1:249782241|249907750
circRNA_001328	2:119260427|119266346	circRNA_006853	10:71303539|71307626	circRNA_007481	2:87512163|87534693	circRNA_001493	3:110351644|110477188
circRNA_020685	14:59627212|59633131	circRNA_020498	10:17493388|17497480	circRNA_011572	2:28863997|28886529	circRNA_004355	4:126532107|126657790
circRNA_011972	7:118637693|118643613	circRNA_000693	13:168412276|168416370	circRNA_004910	8:40321067|40343610	circRNA_001645	4:128114453|128240169
circRNA_021106	6:77191256|77197176	circRNA_015058	8:140242798|140246892	circRNA_004243	4:33438195|33460740	circRNA_021356	X:31329225|31455092
circRNA_004833	7:100611865|100617788	circRNA_008837	13:157186116|157190211	circRNA_008241	X:17765641|17788187	circRNA_013580	X:60908671|61034602
circRNA_006236	5:88176218|88182143	circRNA_018035	GL896294.1:76434|80529	circRNA_011088	10:25171000|25193546	circRNA_010307	11:14714358|14840306
circRNA_016092	4:98128291|98134217	circRNA_019875	GL895802.1:6508|10603	circRNA_016680	1:227387222|227409769	circRNA_005003	8:124790578|124916536
circRNA_017892	6:82339265|82345192	circRNA_006559	X:29457522|29461618	circRNA_014933	4:8469596|8492146	circRNA_009249	2:59752658|59878707
circRNA_014692	3:55588291|55594219	circRNA_013386	8:58349146|58353243	circRNA_004599	6:88173912|88196465	circRNA_006586	X:138428178|138554287
circRNA_019240	8:144330206|144336134	circRNA_015496	13:35906453|35910553	circRNA_001484	3:107388014|107410577	circRNA_006126	4:31337248|31463369
circRNA_020820	17:65846664|65852595	circRNA_000345	10:54048970|54053071	circRNA_012358	10:36414580|36437146	circRNA_004385	4:139285257|139411462
circRNA_004951	8:76268268|76274203	circRNA_012035	9:2724847|2728948	circRNA_009671	6:92600537|92623104	circRNA_003448	14:124568819|124695046
circRNA_006143	4:48150336|48156272	circRNA_012957	2:97459167|97463270	circRNA_009883	8:109416257|109438829	circRNA_019486	13:81422080|81548387
circRNA_007439	2:48072495|48078432	circRNA_007837	6:83154364|83158468	circRNA_009997	9:102545646|102568218	circRNA_012557	13:81422076|81548387
circRNA_007677	4:102444968|102450907	circRNA_020687	14:60224613|60228717	circRNA_016125	5:14001255|14023831	circRNA_015200	1:115168887|115295202
circRNA_018733	14:60224613|60230553	circRNA_008785	13:90157834|90161939	circRNA_015213	1:134656768|134679346	circRNA_011830	5:81262979|81389340
circRNA_005683	13:73300581|73306523	circRNA_007277	15:119473764|119477870	circRNA_012670	14:94931029|94953609	circRNA_019577	15:122363114|122489651
circRNA_003493	15:18583913|18589857	circRNA_015452	12:44463879|44467986	circRNA_001594	4:75094374|75116961	circRNA_001882	6:101811909|101938585
circRNA_016544	GL895834.1:5944|11889	circRNA_001837	6:83154360|83158468	circRNA_000509	12:53698948|53721538	circRNA_011207	13:32911605|33038281
circRNA_009504	4:135701275|135707221	circRNA_008087	9:13612179|13616289	circRNA_018740	14:69961603|69984195	circRNA_012837	17:28370298|28497121
circRNA_017888	6:75821619|75827565	circRNA_021334	GL896348.1:9399|13509	circRNA_001105	17:43030480|43053075	circRNA_018927	2:91104032|91230855
circRNA_019867	GL893811.1:36160|42106	circRNA_019464	12:45358111|45362223	circRNA_006008	2:87193263|87215862	circRNA_006292	6:101811732|101938585
circRNA_021135	6:141379719|141385665	circRNA_019993	13:35785991|35790103	circRNA_011014	1:143403895|143426509	circRNA_011617	2:93467543|93594522
circRNA_015808	18:6253741|6259688	circRNA_012496	12:46872301|46876415	circRNA_020176	4:79478679|79501305	circRNA_015152	1:24730806|24857916
circRNA_017623	14:13298066|13304015	circRNA_021343	JH118622.1:1140|5254	circRNA_016839	14:31361690|31384322	circRNA_008291	1:14572861|14700009
circRNA_003543	15:67043433|67049383	circRNA_021326	GL895152.1:27183|31299	circRNA_000457	12:16845381|16868014	circRNA_011486	16:38550225|38677510
circRNA_011800	5:4583340|4589293	circRNA_002785	10:22255281|22259398	circRNA_016160	5:70565540|70588176	circRNA_020466	1:248694275|248821652
circRNA_015817	18:11044484|11050437	circRNA_000653	13:131233764|131237882	circRNA_019673	3:61075382|61098022	circRNA_018224	2:59753164|59880619
circRNA_012741	15:89580329|89586283	circRNA_014624	10:54052939|54057057	circRNA_018336	7:73306062|73328704	circRNA_003566	15:85397016|85524609
circRNA_003909	2:47993416|47999371	circRNA_005824	14:148216754|148220874	circRNA_016921	15:157073813|157096463	circRNA_000644	13:118617384|118745223
circRNA_016670	1:202606369|202612325	circRNA_009816	7:132103585|132107705	circRNA_005943	18:15609679|15632330	circRNA_015695	15:62005102|62132941
circRNA_016288	7:47638914|47644875	circRNA_013921	4:135338282|135342402	circRNA_014583	GL894048.1:10330|32987	circRNA_001256	2:59752658|59880619
circRNA_002225	9:44289455|44295418	circRNA_009858	8:74026389|74030510	circRNA_008978	14:148266744|148289403	circRNA_005705	13:109189530|109317545
circRNA_018030	GL894485.1:17438|23401	circRNA_013974	JH118536.1:67052|71173	circRNA_017194	6:113167419|113190078	circRNA_015671	14:136831008|136959046
circRNA_019782	6:157477813|157483776	circRNA_020263	7:61904969|61909090	circRNA_013473	9:85856790|85879451	circRNA_013392	8:82828282|82956402
circRNA_013939	6:146057311|146063275	circRNA_019425	10:37127268|37131391	circRNA_010263	10:16978306|17000982	circRNA_020939	3:29633710|29761873
circRNA_020181	4:89196620|89202585	circRNA_009011	15:61680916|61685041	circRNA_016453	9:40653401|40676078	circRNA_001735	5:70565540|70693708
circRNA_002652	1:199984968|199990936	circRNA_006944	12:44742567|44746693	circRNA_004990	8:109019833|109042519	circRNA_012357	10:34185291|34313540
circRNA_013041	3:102684388|102690358	circRNA_008050	8:120759223|120763350	circRNA_002321	GL892430.1:39370|62058	circRNA_017270	8:40899827|41028187
circRNA_007251	15:79278095|79284066	circRNA_011208	13:33180088|33184216	circRNA_004201	3:121371409|121394097	circRNA_011398	15:53008500|53136929
circRNA_005490	1:208265992|208271965	circRNA_020031	14:41904619|41908747	circRNA_007406	18:44530678|44553372	circRNA_012094	9:141099533|141228233
circRNA_003231	13:150105423|150111397	circRNA_001317	2:106419586|106423715	circRNA_021025	4:89318648|89341342	circRNA_003222	13:144478832|144607544
circRNA_014665	15:101687076|101693050	circRNA_011166	12:22570181|22574310	circRNA_009586	5:88156032|88178730	circRNA_003972	2:91206107|91335022

circRNA_017864	5:83944270|83950244	circRNA_019213	8:76038759|76042889	circRNA_015078	11:5210653|5233354	circRNA_006101	3:108496197|108625553
circRNA_004479	5:82622400|82628375	circRNA_004682	6:145562097|145566228	circRNA_014560	9:88615508|88638210	circRNA_018903	2:43662771|43792254
circRNA_009252	2:61866064|61872040	circRNA_009943	9:40553755|40557886	circRNA_019715	5:3454359|3477062	circRNA_020326	9:98614769|98744386
circRNA_001262	2:61866062|61872040	circRNA_003542	15:67038844|67042977	circRNA_002499	1:56083815|56106529	circRNA_001711	5:48749541|48879189
circRNA_015571	13:179735222|179741200	circRNA_011734	3:122012247|122016380	circRNA_011515	17:36694691|36717412	circRNA_014861	7:18392249|18521914
circRNA_015857	2:3016339|3022317	circRNA_005769	14:28594322|28598456	circRNA_009024	15:79278095|79300817	circRNA_016055	4:34502003|34632009
circRNA_010008	9:116098827|116104806	circRNA_011251	13:108395217|108399351	circRNA_002613	1:148474711|148497434	circRNA_020781	16:27711139|27841472
circRNA_010644	3:126256536|126262515	circRNA_016499	9:125462130|125466266	circRNA_015629	14:66861565|66884293	circRNA_008481	1:276560955|276691357
circRNA_010065	GL894654.1:53813|59794	circRNA_003170	13:91070338|91074476	circRNA_020378	1:58468249|58490977	circRNA_012789	16:20241801|20372260
circRNA_013711	3:83018888|83024870	circRNA_005546	10:29342271|29346409	circRNA_002120	8:93218969|93241704	circRNA_012336	10:7611088|7741573
circRNA_015732	15:131239708|131245690	circRNA_011749	4:30435928|30440066	circRNA_013139	4:137324015|137346750	circRNA_001037	16:24863564|24994149
circRNA_002053	8:1832006|1837990	circRNA_017140	5:38341812|38345950	circRNA_002854	10:63257104|63279841	circRNA_003968	2:89865397|89996112
circRNA_006555	X:16250920|16256906	circRNA_011123	10:58856976|58861115	circRNA_010074	GL895038.1:26630|49369	circRNA_006384	8:9383044|9513782
circRNA_017244	7:124815434|124821420	circRNA_016454	9:40659448|40663587	circRNA_003308	14:21462664|21485412	circRNA_003986	2:105006195|105136942
circRNA_017759	2:74635393|74641379	circRNA_019575	15:115739284|115743423	circRNA_009854	8:69688714|69711465	circRNA_005345	X:70509183|70639982
circRNA_015382	11:22240397|22246384	circRNA_009132	17:13125525|13129665	circRNA_017234	7:91930063|91952828	circRNA_010932	X:60892361|61023192
circRNA_005781	14:42346799|42352788	circRNA_012290	1:205261252|205265392	circRNA_003480	14:147004006|147026778	circRNA_019921	1:103946010|104076842
circRNA_012908	2:28877843|28883832	circRNA_003334	14:33446619|33450760	circRNA_003026	12:44795503|44818283	circRNA_020170	4:40466582|40597565
circRNA_020283	8:39572078|39578069	circRNA_015045	7:43282112|43286255	circRNA_008573	11:244344|267124	circRNA_000306	10:29900373|30031437
circRNA_012500	12:48316172|48322165	circRNA_013659	1:176836675|176840819	circRNA_002986	12:23727465|23750252	circRNA_009341	3:29637831|29768928
circRNA_009954	9:43027884|43033878	circRNA_005096	9:37674853|37678998	circRNA_003319	14:30062323|30085114	circRNA_018305	6:38017733|38149056
circRNA_011529	17:57040110|57046105	circRNA_017996	9:78959290|78963438	circRNA_003680	16:27888389|27911180	circRNA_012795	16:24863564|24995151
circRNA_019603	18:834115|840110	circRNA_015429	12:16190418|16194567	circRNA_006823	10:30411251|30434042	circRNA_001202	18:59790060|59921726
circRNA_004489	5:88199605|88205602	circRNA_004441	5:50345400|50349551	circRNA_015251	1:211745761|211768556	circRNA_000362	10:73581212|73713062
circRNA_001279	2:79850166|79856164	circRNA_011220	13:47838870|47843023	circRNA_015868	2:19819539|19842334	circRNA_021125	6:101802150|101934045
circRNA_011897	6:101014811|101020809	circRNA_004518	6:9691062|9695218	circRNA_004626	6:99220825|99243627	circRNA_005742	13:191655032|191786950
circRNA_009965	9:46891203|46897203	circRNA_013635	4:60858276|60862433	circRNA_004103	3:72497166|72519969	circRNA_005452	1:135900642|136032804
circRNA_002272	9:101637290|101643291	circRNA_016200	6:26453983|26458140	circRNA_011463	16:18574324|18597138	circRNA_019657	2:144853391|144985707
circRNA_015404	11:69874965|69880966	circRNA_017948	8:3356607|3360764	circRNA_019926	1:146713282|146736096	circRNA_021160	7:38746540|38878866
circRNA_019548	14:116995830|117001831	circRNA_010873	9:40569292|40573450	circRNA_001311	2:98306632|98329451	circRNA_013234	6:40979784|41112219
circRNA_001420	3:51121496|51127498	circRNA_018831	16:46608541|46612699	circRNA_003311	14:21553354|21576174	circRNA_005548	10:29898685|30031437
circRNA_013464	9:72020043|72026045	circRNA_007523	2:153598277|153602436	circRNA_013235	6:40990421|41013246	circRNA_003557	15:85315348|85448116
circRNA_014411	3:135192228|135198230	circRNA_006966	13:14280479|14284639	circRNA_020226	6:85271142|85293970	circRNA_020225	6:82216149|82348935
circRNA_019260	9:40763445|40769449	circRNA_012640	14:49264881|49269041	circRNA_003363	14:60101240|60124073	circRNA_000694	13:169207897|169340724
circRNA_005496	1:216057822|216063829	circRNA_015885	2:60797706|60801866	circRNA_015322	10:2279240|2302077	circRNA_009377	3:78256245|78389094
circRNA_011284	13:192284884|192290892	circRNA_020147	3:81444405|81448565	circRNA_014501	7:91890126|91912965	circRNA_000197	1:216314524|216447657
circRNA_014093	1:23010569|23016578	circRNA_000635	13:108413402|108417563	circRNA_002564	1:124861734|124884580	circRNA_005412	1:24415655|24549017
circRNA_019829	8:108489456|108495465	circRNA_001009	15:122873048|122877209	circRNA_013217	6:10323362|10346209	circRNA_006618	1:24724428|24857916
circRNA_001524	3:125921425|125927436	circRNA_002919	11:52182965|52187127	circRNA_004846	7:117047645|117070498	circRNA_000247	1:276557775|276691357
circRNA_004919	8:46580336|46586348	circRNA_011768	4:51225215|51229377	circRNA_019619	2:28385524|28408378	circRNA_010989	1:125384211|125517917
circRNA_005460	1:144474045|144480057	circRNA_019956	10:11769844|11774006	circRNA_007604	3:120136072|120158933	circRNA_008586	11:14714358|14848094
circRNA_004282	4:67773102|67779115	circRNA_000028	1:23262198|23266361	circRNA_012456	12:367331|390195	circRNA_011397	15:42712143|42845918
circRNA_015565	13:159585336|159591350	circRNA_002558	1:117504416|117508579	circRNA_016553	GL896387.1:21398|44265	circRNA_000904	15:42711885|42845918
circRNA_006530	GL894597.1:39761|45776	circRNA_014985	13:81420907|81425070	circRNA_016820	13:144099062|144121936	circRNA_000955	15:85315348|85449431
circRNA_006655	1:97325990|97332007	circRNA_016314	7:100613625|100617788	circRNA_003178	13:97291986|97314870	circRNA_017514	10:17707596|17841794
circRNA_007761	5:72241037|72247057	circRNA_006056	3:5929433|5933598	circRNA_003076	13:20785157|20808042	circRNA_007597	3:113310620|113444950
circRNA_011974	7:120263165|120269187	circRNA_017435	1:80301417|80305582	circRNA_003745	17:9730340|9753228	circRNA_008511	10:17035878|17170362
circRNA_012526	13:38021930|38027952	circRNA_021077	5:92460533|92464698	circRNA_003988	2:105133377|105156268	circRNA_003108	13:41489623|41624117
circRNA_008922	14:69978172|69984195	circRNA_001725	5:61500662|61504828	circRNA_003590	15:104515243|104538139	circRNA_013280	6:138823936|138958631
circRNA_002232	9:51073473|51079497	circRNA_009077	16:18451019|18455186	circRNA_001261	2:61502930|61525829	circRNA_004878	8:9736245|9871034
circRNA_019760	6:73305899|73311923	circRNA_005411	1:23262192|23266361	circRNA_019974	12:424100|447000	circRNA_017410	X:138419390|138554287
circRNA_002360	GL895152.1:20630|26657	circRNA_007273	15:112541222|112545392	circRNA_002659	1:200817316|200840229	circRNA_000888	15:20231068|20366014

circRNA_002471	1:18496821|18502849	circRNA_016796	13:67643034|67647204	circRNA_014457	5:76358127|76381045	circRNA_016072	4:67851823|67986787
circRNA_002601	1:143279275|143285303	circRNA_008373	1:134611516|134615687	circRNA_021340	JH118536.1:110872|133790	circRNA_004890	8:29175726|29310723
circRNA_015511	13:55486493|55492521	circRNA_015946	3:4712078|4716249	circRNA_019994	13:37315215|37338139	circRNA_001080	16:86331873|86467016
circRNA_018416	1:59709107|59715138	circRNA_018006	9:127436529|127440700	circRNA_000762	14:31384107|31407036	circRNA_006208	5:53477815|53612985
circRNA_005823	14:148062722|148068754	circRNA_005320	X:17547484|17551656	circRNA_010802	7:73236844|73259774	circRNA_001079	16:86331835|86467016
circRNA_012318	1:278933588|278939621	circRNA_015388	11:25528481|25532653	circRNA_019761	6:85261799|85284732	circRNA_004072	3:29633710|29768928
circRNA_012624	14:30058998|30065031	circRNA_018546	10:38235616|38239788	circRNA_007477	2:85467251|85490186	circRNA_021368	X:60892361|61027603
circRNA_021046	5:23806156|23812189	circRNA_010206	1:134653309|134657483	circRNA_016615	1:6463387|6486326	circRNA_002419	X:30958644|31093895
circRNA_006602	1:13637433|13643469	circRNA_019841	9:13731293|13735468	circRNA_001113	17:45653745|45676687	circRNA_000032	1:30747601|30883016
circRNA_017336	9:70877652|70883688	circRNA_000114	1:132995834|133000011	circRNA_003210	13:142814626|142837573	circRNA_008290	1:10697954|10833574
circRNA_005584	11:16102041|16108078	circRNA_012460	12:3452757|3456934	circRNA_000543	13:24109347|24132299	circRNA_019300	9:148682574|148818195
circRNA_005907	16:71618182|71624219	circRNA_015594	14:10546491|10550668	circRNA_008690	12:59357665|59380626	circRNA_010481	15:89986891|90122644
circRNA_010747	6:75400769|75406806	circRNA_016999	2:8540194|8544371	circRNA_013279	6:137838723|137861689	circRNA_012288	1:202361787|202497584
circRNA_021318	GL894485.1:17364|23401	circRNA_019227	8:103694327|103698505	circRNA_015858	2:3016339|3039309	circRNA_016667	1:187487437|187623258
circRNA_007394	18:27278420|27284461	circRNA_009840	8:42394633|42398815	circRNA_008669	12:37697401|37720372	circRNA_002478	1:24721757|24857916
circRNA_017599	13:82208452|82214494	circRNA_014174	12:319532|323714	circRNA_012917	2:43651009|43673981	circRNA_014271	15:3551454|3687801
circRNA_008260	X:73963388|73969431	circRNA_004171	3:109759159|109763342	circRNA_004819	7:91959137|91982115	circRNA_012643	14:52135828|52272182
circRNA_014749	GL893841.1:80332|86375	circRNA_013973	GL894597.1:41592|45776	circRNA_000587	13:49972583|49995564	circRNA_006416	8:85838692|85975055
circRNA_000488	12:39721598|39727643	circRNA_017227	7:64448638|64452823	circRNA_005603	11:73765041|73788022	circRNA_010886	9:77278140|77414550
circRNA_013285	6:146174273|146180319	circRNA_009302	2:138953586|138957772	circRNA_005694	13:83179635|83202617	circRNA_004635	6:101802150|101938585
circRNA_016978	18:12308719|12314765	circRNA_013785	1:222076706|222080892	circRNA_002687	1:224091690|224114675	circRNA_000274	1:311558220|311694687
circRNA_009120	16:71618182|71624229	circRNA_004729	7:21527557|21531745	circRNA_006597	1:4648182|4671167	circRNA_010470	15:36264244|36400720
circRNA_014716	6:18418914|18424962	circRNA_018983	3:75752217|75756405	circRNA_016425	9:8923702|8946694	circRNA_017062	3:29625387|29761873
circRNA_016381	8:83694630|83700678	circRNA_002077	8:39363978|39368167	circRNA_021261	9:43027884|43050877	circRNA_004174	3:110340669|110477188
circRNA_003200	13:126706625|126712674	circRNA_004288	4:70738380|70742572	circRNA_006078	3:62627636|62650638	circRNA_012384	10:55772042|55908729
circRNA_016520	GL893884.1:35252|41304	circRNA_011032	1:200296262|200300454	circRNA_000103	1:125356988|125379993	circRNA_006344	7:72233626|72370421
circRNA_021385	X:127109815|127115867	circRNA_014249	14:53911449|53915641	circRNA_014253	14:64835671|64858680	circRNA_019145	7:5331103|5467989
circRNA_012625	14:30929985|30936038	circRNA_016011	3:109429044|109433236	circRNA_001610	4:93598992|93622015	circRNA_002109	8:83143287|83280539
circRNA_017219	7:43188490|43194543	circRNA_002076	8:39363973|39368167	circRNA_015883	2:50878012|50901039	circRNA_000288	10:17067380|17204667
circRNA_008012	8:58299514|58305568	circRNA_012596	13:158890936|158895132	circRNA_012525	13:37965105|37988133	circRNA_005894	16:38539919|38677510
circRNA_013292	6:151972841|151978896	circRNA_014146	1:282228442|282232638	circRNA_016180	5:93506418|93529450	circRNA_015924	2:130662211|130799830
circRNA_004663	6:132167543|132173603	circRNA_014466	6:18436857|18441054	circRNA_002971	12:16062482|16085519	circRNA_017266	8:39610566|39748311
circRNA_019588	16:51707557|51713618	circRNA_019384	1:144113381|144117578	circRNA_015093	3:77329460|77352520	circRNA_005577	11:1483999|1621832
circRNA_005654	13:28757838|28763904	circRNA_007710	5:284160|288359	circRNA_018660	13:131105127|131128200	circRNA_007949	7:101076419|101214404
circRNA_001287	2:86203222|86209289	circRNA_016990	18:36574348|36578547	circRNA_012091	9:138768104|138791180	circRNA_001937	6:155228017|155366003
circRNA_012931	2:66303298|66309366	circRNA_010088	JH118500.1:18089|22289	circRNA_002798	10:31451602|31474683	circRNA_003599	15:111700846|111838997
circRNA_011835	5:86522733|86528802	circRNA_001820	6:69449174|69453376	circRNA_017225	7:61994439|62017523	circRNA_000986	15:111700822|111838997
circRNA_020782	16:29069843|29075913	circRNA_000120	1:134632355|134636559	circRNA_005289	JH118638.1:257421|280507	circRNA_018326	6:155228017|155366257
circRNA_021113	6:88284256|88290327	circRNA_000093	1:119639317|119643524	circRNA_007221	15:36377634|36400720	circRNA_009058	15:128043602|128181867
circRNA_008340	1:93560104|93566176	circRNA_009351	3:43187440|43191647	circRNA_018337	7:94502841|94525927	circRNA_019544	14:112021949|112160229
circRNA_002026	7:102134420|102140493	circRNA_012261	1:143361257|143365464	circRNA_009332	3:19045470|19068557	circRNA_018457	1:152242325|152380690
circRNA_005629	12:44738008|44744081	circRNA_011133	11:3318336|3322545	circRNA_001508	3:119833596|119856704	circRNA_006448	9:11252773|11391169
circRNA_013269	6:110742597|110748671	circRNA_012038	9:7316506|7320716	circRNA_012793	16:22354808|22377921	circRNA_002200	9:22356187|22495114
circRNA_002787	10:24480147|24486223	circRNA_020094	17:57209999|57214209	circRNA_002355	GL894855.1:11155|34269	circRNA_004633	6:101794657|101934045
circRNA_013178	5:68121984|68128063	circRNA_001896	6:125473732|125477945	circRNA_019680	3:103798859|103821979	circRNA_001492	3:110337622|110477188
circRNA_007485	2:90152679|90158760	circRNA_004211	3:130451751|130455965	circRNA_010897	9:115756518|115779641	circRNA_011752	4:34502003|34641574
circRNA_005818	14:141297378|141303460	circRNA_004652	6:125473729|125477945	circRNA_005288	JH118611.1:2203|25329	circRNA_016465	9:54516427|54656123
circRNA_017415	1:2764696|2770778	circRNA_004880	8:11027197|11031413	circRNA_019894	X:121774097|121797223	circRNA_001550	4:17112350|17252337
circRNA_017897	6:88253944|88260029	circRNA_015318	1:307316479|307320696	circRNA_017261	8:32104644|32127777	circRNA_015373	11:16411857|16552093
circRNA_008006	8:43554347|43560434	circRNA_021054	5:49645990|49650207	circRNA_005355	X:85729279|85752413	circRNA_007499	2:105006195|105146567
circRNA_020864	2:29207475|29213562	circRNA_000436	11:86983006|86987226	circRNA_020789	16:47777338|47800473	circRNA_006066	3:39139678|39280065
circRNA_000799	14:71895250|71901340	circRNA_013683	13:97278057|97282277	circRNA_001024	15:145717023|145740163	circRNA_007113	14:38303346|38443845

circRNA_002012	7:91976025|91982115	circRNA_008832	13:148413481|148417703	circRNA_008528	10:35288939|35312084	circRNA_010829	8:44428186|44568805
circRNA_018453	1:143833232|143839322	circRNA_006207	5:50996616|51000839	circRNA_003998	2:119219842|119242988	circRNA_014904	1:12093964|12234659
circRNA_019810	7:117149223|117155315	circRNA_005299	JH118990.1:88314|92538	circRNA_016363	8:57221410|57244558	circRNA_008182	9:148677390|148818195
circRNA_017436	1:83300056|83306149	circRNA_017944	7:120861235|120865459	circRNA_012162	X:85729279|85752440	circRNA_003352	14:52118651|52259674
circRNA_005146	9:101932475|101938570	circRNA_007059	13:142624887|142629113	circRNA_010861	9:8923702|8946866	circRNA_006418	8:86895451|87036717
circRNA_005176	9:129718279|129724374	circRNA_000520	12:62443557|62447784	circRNA_020265	7:73050148|73073314	circRNA_002989	12:26628108|26769452
circRNA_007091	14:6687380|6693475	circRNA_004992	8:112056641|112060868	circRNA_002607	1:145394702|145417869	circRNA_014899	X:70498623|70639982
circRNA_015627	14:66665847|66671943	circRNA_005205	9:144311825|144316052	circRNA_021091	6:16261615|16284783	circRNA_020308	9:22359943|22501577
circRNA_018291	5:88034234|88040330	circRNA_009357	3:46305647|46309875	circRNA_018631	13:43394660|43417833	circRNA_008870	14:2697618|2839255
circRNA_003147	13:81204047|81210144	circRNA_020312	9:31714697|31718925	circRNA_006857	11:472454|495629	circRNA_012269	1:155637573|155779292
circRNA_012684	14:116845427|116851525	circRNA_000497	12:45493347|45497578	circRNA_015434	12:23370369|23393554	circRNA_007089	14:2697410|2839255
circRNA_013055	3:122049816|122055914	circRNA_002600	1:143271555|143275786	circRNA_014989	13:190108609|190131796	circRNA_004666	6:134374868|134517145
circRNA_015528	13:90909955|90916053	circRNA_007151	14:77678428|77682661	circRNA_017084	3:113260630|113283820	circRNA_013067	4:2565801|2708122
circRNA_019648	2:134971674|134977773	circRNA_000850	14:117646846|117651080	circRNA_009401	3:110351644|110374835	circRNA_011068	1:282416588|282559008
circRNA_012547	13:57980796|57986896	circRNA_003282	13:213488484|213492718	circRNA_007475	2:83895486|83918678	circRNA_012320	1:282416588|282559011
circRNA_001435	3:67426807|67432910	circRNA_005218	GL892547.1:5870|10104	circRNA_008358	1:118422479|118445673	circRNA_009577	5:80219696|80362315
circRNA_019799	7:81021047|81027150	circRNA_017924	7:21301250|21305484	circRNA_014663	15:86615927|86639123	circRNA_005598	11:52209155|52351983
circRNA_003019	12:40652196|40658300	circRNA_017090	3:122019922|122024159	circRNA_006955	13:1169521|1192718	circRNA_001142	18:5280797|5423652
circRNA_018964	3:19102747|19108851	circRNA_007064	13:144109883|144114121	circRNA_019256	9:37001749|37024947	circRNA_004963	8:85838692|85981573
circRNA_012371	10:48888172|48894277	circRNA_016591	X:64983276|64987514	circRNA_012660	14:68425345|68448550	circRNA_020079	16:47180663|47323675
circRNA_017518	10:31147639|31153744	circRNA_010688	5:3341916|3346156	circRNA_005992	2:79850166|79873381	circRNA_014357	2:43654513|43797607
circRNA_017560	12:23060087|23066192	circRNA_012618	14:14155674|14159914	circRNA_004741	7:31326169|31349385	circRNA_008361	1:125384211|125527319
circRNA_000404	11:22552052|22558158	circRNA_013399	8:85727369|85731609	circRNA_010844	8:89343634|89366855	circRNA_001135	18:1417740|1560859
circRNA_003398	14:82226229|82232335	circRNA_018112	11:21071689|21075929	circRNA_000998	15:117863548|117886776	circRNA_005684	13:74656936|74800063
circRNA_006442	9:255216|261322	circRNA_001925	6:148116282|148120523	circRNA_006160	4:90913408|90936640	circRNA_019364	1:55488775|55631926
circRNA_015928	2:133243083|133249189	circRNA_003367	14:63598743|63602985	circRNA_001601	4:88603967|88627201	circRNA_021021	4:74061445|74204622
circRNA_003067	13:15488018|15494125	circRNA_010541	18:16027728|16031971	circRNA_008477	1:267357282|267380520	circRNA_009979	9:73422558|73565768
circRNA_000884	15:18673987|18680096	circRNA_007524	2:153837681|153841925	circRNA_002465	1:16142012|16165251	circRNA_000629	13:104105327|104248782
circRNA_011423	15:88650686|88656798	circRNA_017907	6:125764845|125769090	circRNA_016641	1:110340582|110363821	circRNA_001398	3:29625387|29768928
circRNA_008896	14:42062995|42069112	circRNA_019641	2:107140522|107144767	circRNA_015474	13:7975063|7998309	circRNA_002617	1:152237006|152380690
circRNA_002088	8:57794720|57800838	circRNA_015682	15:19240976|19245225	circRNA_008334	1:86310250|86333500	circRNA_012024	8:124800369|124944168
circRNA_011034	1:200530333|200536451	circRNA_005578	11:5189667|5193917	circRNA_019536	14:77573358|77596608	circRNA_001881	6:101794657|101938585
circRNA_017005	2:12191539|12197660	circRNA_019100	6:43974527|43978777	circRNA_004608	6:89974808|89998069	circRNA_012773	15:126139921|126283979
circRNA_017733	18:14900760|14906881	circRNA_009612	6:13244631|13248882	circRNA_004889	8:20670183|20693450	circRNA_017986	9:49400246|49544907
circRNA_004659	6:127528135|127534257	circRNA_016606	X:115972959|115977210	circRNA_013362	7:128864504|128887774	circRNA_006574	X:70495253|70639982
circRNA_016715	10:15161153|15167275	circRNA_001425	3:55619049|55623302	circRNA_006615	1:23166052|23189331	circRNA_008750	13:44599569|44744392
circRNA_012786	15:150711593|150717716	circRNA_005658	13:33153278|33157534	circRNA_018434	1:110525054|110548336	circRNA_001788	6:32239126|32383981
circRNA_013313	7:40044112|40050237	circRNA_011052	1:248085318|248089574	circRNA_013492	9:120346324|120369612	circRNA_007809	6:40223596|40368469
circRNA_008480	1:275512758|275518884	circRNA_014563	9:108507681|108511937	circRNA_000268	1:304797554|304820843	circRNA_010781	7:10297937|10442822
circRNA_018544	10:37488219|37494346	circRNA_019518	14:7197260|7201516	circRNA_006517	GL892789.1:71280|94569	circRNA_008725	13:28075791|28220691
circRNA_000484	12:37781159|37787288	circRNA_017987	9:49650669|49654926	circRNA_007854	6:103831012|103854303	circRNA_013049	3:119558816|119703933
circRNA_009254	2:65409394|65415523	circRNA_001661	4:134766749|134771008	circRNA_000868	14:143526339|143549632	circRNA_015193	1:101723122|101868259
circRNA_009809	7:118972758|118978888	circRNA_004709	6:153869620|153873880	circRNA_001895	6:125472096|125495394	circRNA_000040	1:37400352|37545495
circRNA_014309	16:23689485|23695616	circRNA_012103	GL893011.1:29007|33267	circRNA_016908	15:101687076|101710376	circRNA_014858	6:148924023|149069232
circRNA_001136	18:1457168|1463300	circRNA_015570	13:168399300|168403560	circRNA_019546	14:116588489|116611789	circRNA_002201	9:22356187|22501577
circRNA_009528	5:29928554|29934686	circRNA_021201	8:57881908|57886168	circRNA_008614	11:56283465|56306768	circRNA_013852	15:124483398|124629189
circRNA_016395	8:109063414|109069549	circRNA_009707	6:146168407|146172668	circRNA_003060	13:2382115|2405429	circRNA_021150	7:17106407|17252211
circRNA_007450	2:61771776|61777912	circRNA_016210	6:50127662|50131923	circRNA_012117	GL893884.1:17986|41304	circRNA_010011	9:124334896|124480730
circRNA_002315	GL892332.2:318148|324287	circRNA_020848	18:52365395|52369658	circRNA_019064	5:33923729|33947048	circRNA_000604	13:79310756|79456601
circRNA_004466	5:73403403|73409542	circRNA_010386	13:115692729|115696994	circRNA_005166	9:127417379|127440700	circRNA_015943	2:148072198|148218077
circRNA_000985	15:111160776|111166916	circRNA_012498	12:47119831|47124096	circRNA_018776	15:20134421|20157746	circRNA_016716	10:17000906|17146941
circRNA_005863	15:112575780|112581920	circRNA_001453	3:84172862|84177128	circRNA_000085	1:110525008|110548336	circRNA_021056	5:51954308|52100360

circRNA_002215	9:40148558|40154700	circRNA_001512	3:120260142|120264408	circRNA_018644	13:86952848|86976189	circRNA_017097	4:34570313|34716368
circRNA_002316	GL892334.1:16859|23003	circRNA_003719	16:51757091|51761357	circRNA_008517	10:18187787|18211136	circRNA_013896	3:39133909|39280065
circRNA_021209	8:76009572|76015718	circRNA_019354	X:122510620|122514886	circRNA_016806	13:99622614|99645964	circRNA_008566	10:66012330|66158539
circRNA_006436	8:144334629|144340776	circRNA_003387	14:78059545|78063812	circRNA_018606	12:44468807|44492157	circRNA_019416	1:297882991|298029257
circRNA_006317	6:157496770|157502922	circRNA_013600	1:17236721|17240990	circRNA_005819	14:142519572|142542925	circRNA_011122	10:58766088|58912493
circRNA_014381	3:16859282|16865434	circRNA_014083	3:81728554|81732824	circRNA_008729	13:34710521|34733874	circRNA_011485	16:38531076|38677510
circRNA_018700	14:10552563|10558715	circRNA_017232	7:86329719|86333989	circRNA_004887	8:19245871|19269225	circRNA_020966	3:73853247|73999685
circRNA_007272	15:111934423|111940576	circRNA_001730	5:69536942|69541214	circRNA_003414	14:98721243|98744598	circRNA_010863	9:12430238|12576733
circRNA_019209	8:43034371|43040527	circRNA_002669	1:202809063|202813336	circRNA_005602	11:57026791|57050146	circRNA_001315	2:101213695|101360310
circRNA_015520	13:81437153|81443311	circRNA_016565	JH118804.1:26494|30767	circRNA_009051	15:118153598|118176962	circRNA_012232	1:101723122|101869910
circRNA_016057	4:37761441|37767599	circRNA_003347	14:47009549|47013823	circRNA_007775	5:91292773|91316139	circRNA_007126	14:51990364|52137218
circRNA_016633	1:64737843|64744001	circRNA_018020	GL893374.2:642|4916	circRNA_009773	7:73129142|73152508	circRNA_001451	3:81720523|81867496
circRNA_004132	3:84816403|84822563	circRNA_008970	14:141164858|141169134	circRNA_014951	9:129701005|129724374	circRNA_007112	14:38296637|38443845
circRNA_021238	8:144579683|144585844	circRNA_018584	11:73783745|73788022	circRNA_010508	16:55636224|55659606	circRNA_018105	10:43794349|43941568
circRNA_017045	2:135129191|135135354	circRNA_020054	15:19408233|19412511	circRNA_014647	13:154030700|154054087	circRNA_004672	6:138456117|138603577
circRNA_020704	14:112947191|112953354	circRNA_011859	6:10723002|10727281	circRNA_000946	15:79683656|79707046	circRNA_008257	X:70506113|70653651
circRNA_006713	1:161765502|161771667	circRNA_019057	5:4780019|4784298	circRNA_004582	6:79809424|79832814	circRNA_000290	10:17976426|18124093
circRNA_011283	13:190993013|190999178	circRNA_011015	1:144479851|144484131	circRNA_011417	15:85377561|85400955	circRNA_010648	4:15915883|16063617
circRNA_018089	1:180819278|180825445	circRNA_000005	1:11314741|11319029	circRNA_002701	1:241901877|241925272	circRNA_011963	7:100470025|100617788
circRNA_019489	13:99646867|99653035	circRNA_009112	16:53279948|53284237	circRNA_002714	1:250031211|250054617	circRNA_008962	14:136612215|136760286
circRNA_002684	1:212154210|212160381	circRNA_008270	X:112858629|112862919	circRNA_012280	1:181354539|181377948	circRNA_020022	13:157561666|157709777
circRNA_010062	GL894396.1:31788|37959	circRNA_021066	5:84028946|84033236	circRNA_021030	4:116997042|117020457	circRNA_000325	10:37797097|37945250
circRNA_006536	GL895476.1:2448|8621	circRNA_001292	2:86313971|86318262	circRNA_018487	1:202012272|202035691	circRNA_001828	6:80072109|80220368
circRNA_011649	2:153835752|153841925	circRNA_010460	14:144091535|144095826	circRNA_005953	18:43963437|43986861	circRNA_002301	9:141099533|141247877
circRNA_017503	1:275479065|275485240	circRNA_006789	1:293365114|293369407	circRNA_006971	13:20785157|20808581	circRNA_005541	10:18101700|18250156
circRNA_018227	2:71199117|71205292	circRNA_008874	14:10569830|10574128	circRNA_012638	14:41706715|41730148	circRNA_010706	5:63577496|63726108
circRNA_018967	3:29713966|29720141	circRNA_013732	8:140632643|140636941	circRNA_012667	14:81008366|81031799	circRNA_001726	5:63577496|63726112
circRNA_008266	X:101603649|101609826	circRNA_016044	3:135362530|135366829	circRNA_005478	1:200310792|200334231	circRNA_014367	2:104362868|104511554
circRNA_013429	9:34787|40964	circRNA_000917	15:55436411|55440711	circRNA_011467	16:23568811|23592263	circRNA_008183	9:150229063|150377795
circRNA_003747	17:13115098|13121277	circRNA_014722	6:137857387|137861689	circRNA_000066	1:80332936|80356402	circRNA_018687	13:191655032|191803897
circRNA_020775	16:19057467|19063646	circRNA_015837	18:36874126|36878428	circRNA_015669	14:135894355|135917823	circRNA_013089	4:41705077|41854004
circRNA_002834	10:54052939|54059121	circRNA_001353	2:140877263|140881566	circRNA_017276	8:69681334|69704803	circRNA_013088	4:41704933|41854004
circRNA_007177	14:120772985|120779168	circRNA_021273	9:79036909|79041213	circRNA_012744	15:97994575|98018046	circRNA_016435	9:19770895|19919969
circRNA_012410	11:2121496|2127679	circRNA_005976	2:43984992|43989297	circRNA_002578	1:132344010|132367489	circRNA_015081	12:20704130|20853326
circRNA_021173	7:94867449|94873632	circRNA_018087	1:151801299|151805605	circRNA_001086	17:13778285|13801776	circRNA_015386	11:24688121|24837421
circRNA_017112	4:55333062|55339246	circRNA_019972	11:70152479|70156785	circRNA_000382	11:11037891|11061383	circRNA_010614	3:44108245|44257790
circRNA_019997	13:42380107|42385318	circRNA_020391	1:83296707|83301016	circRNA_011943	7:57257302|57280795	circRNA_019899	1:11017377|11167099
circRNA_017804	3:113971998|113977210	circRNA_020485	1:293346808|293351119	circRNA_002509	1:60917934|60941431	circRNA_004528	6:14027559|14177354
circRNA_017143	5:63653157|63658370	circRNA_000648	13:130356500|130360814	circRNA_007754	5:63605306|63628804	circRNA_005013	8:134408464|134558516
circRNA_005033	8:144844945|144850159	circRNA_000535	13:19726699|19731015	circRNA_010161	X:140015694|140039198	circRNA_007500	2:105006195|105156268
circRNA_015043	6:137344230|137349444	circRNA_006891	11:52212325|52216641	circRNA_000934	15:71703524|71727032	circRNA_010422	14:26145419|26295541
circRNA_017465	1:157512255|157517469	circRNA_000100	1:125143608|125147925	circRNA_007358	17:43066397|43089928	circRNA_005198	9:141099533|141249686
circRNA_000616	13:87209879|87215095	circRNA_006279	6:85033033|85037351	circRNA_018206	18:27396026|27419562	circRNA_001397	3:29618701|29768928
circRNA_014956	1:16119378|16124594	circRNA_020084	16:78841787|78846106	circRNA_013065	4:2565801|2589339	circRNA_011770	4:61796451|61946991
circRNA_015345	10:44199589|44204806	circRNA_006045	2:142522062|142526384	circRNA_000699	13:199361578|199385121	circRNA_016759	12:35849683|36000267
circRNA_000227	1:255743644|255748862	circRNA_008377	1:134656768|134661090	circRNA_009274	2:91207311|91230855	circRNA_007411	18:59169585|59320297
circRNA_006621	1:28834008|28839227	circRNA_018198	17:33108518|33112840	circRNA_004265	4:43494812|43518358	circRNA_002249	9:77278140|77428858
circRNA_015530	13:90980638|90985857	circRNA_010376	13:75527377|75531702	circRNA_017240	7:116970559|116994106	circRNA_016267	7:10291894|10442822
circRNA_011379	14:136289720|136294940	circRNA_009878	8:104001015|104005341	circRNA_013910	4:40202123|40225678	circRNA_018229	2:78532006|78682981
circRNA_016569	X:3677199|3682419	circRNA_013259	6:90434708|90439034	circRNA_019019	4:20250184|20273742	circRNA_010132	X:70495253|70646265
circRNA_000065	1:72651152|72656373	circRNA_001101	17:42143862|42148189	circRNA_016390	8:108342978|108366542	circRNA_017865	5:87157448|87308493
circRNA_002397	MT:1680|6901	circRNA_002513	1:62694950|62699277	circRNA_003059	13:1141433|1164999	circRNA_017513	10:17019078|17170362

circRNA_005841	15:66679247|66684469	circRNA_012980	2:146097371|146101698	circRNA_006546	JH118763.1:25291|48859	circRNA_001450	3:81716210|81867496
circRNA_002043	7:124977818|124983041	circRNA_016637	1:101706423|101710750	circRNA_002775	10:17146793|17170362	circRNA_007941	7:97005367|97156735
circRNA_013364	7:129081843|129087067	circRNA_018163	14:98657157|98661484	circRNA_019380	1:134632355|134655932	circRNA_008869	14:2687446|2839255
circRNA_013956	8:140235965|140241189	circRNA_003418	14:108069201|108073530	circRNA_011454	15:147691480|147715063	circRNA_009547	5:53477815|53630601
circRNA_000643	13:117702728|117707954	circRNA_010450	14:116344443|116348772	circRNA_020733	15:66049072|66072656	circRNA_002141	8:124800369|124953169
circRNA_004874	8:2813165|2818391	circRNA_001825	6:77568549|77572879	circRNA_002865	10:77696541|77720129	circRNA_006609	1:18201035|18353859
circRNA_005529	1:299807814|299813040	circRNA_021036	4:135644679|135649009	circRNA_003914	2:52228455|52252052	circRNA_015287	1:254784151|254937077
circRNA_009814	7:129886421|129891648	circRNA_002534	1:89946850|89951181	circRNA_010355	13:23702427|23726024	circRNA_018159	14:63568122|63721084
circRNA_007010	13:82870481|82875709	circRNA_011468	16:23582249|23586581	circRNA_010576	2:87512167|87535775	circRNA_009648	6:73383552|73536700
circRNA_015356	10:54438706|54443934	circRNA_016040	3:135029602|135033934	circRNA_020508	10:46596019|46619628	circRNA_015420	12:13391189|13544430
circRNA_021114	6:88384555|88389783	circRNA_001278	2:78551140|78555473	circRNA_010926	X:8558016|8581627	circRNA_012023	8:124790578|124944168
circRNA_009166	18:4983023|4988253	circRNA_015576	13:202631408|202635742	circRNA_015643	14:81745652|81769280	circRNA_014943	7:22160522|22314115
circRNA_012897	2:2992758|2997988	circRNA_007048	13:130356479|130360814	circRNA_012677	14:113218104|113241733	circRNA_015679	15:4735614|4889299
circRNA_003905	2:45062228|45067459	circRNA_005814	14:140515705|140520043	circRNA_015070	X:77219474|77243106	circRNA_009507	4:137358677|137512400
circRNA_006197	5:32061014|32066245	circRNA_017450	1:125143587|125147925	circRNA_001593	4:73759207|73782852	circRNA_019170	7:71386159|71540188
circRNA_013260	6:91868362|91873593	circRNA_002911	11:25904563|25908902	circRNA_002297	9:139626975|139650620	circRNA_016624	1:23831908|23986113
circRNA_007128	14:52822859|52828091	circRNA_004711	7:4587208|4591548	circRNA_016470	9:62385033|62408681	circRNA_013267	6:101779796|101934045
circRNA_020276	7:119961617|119966852	circRNA_008562	10:63278994|63283334	circRNA_018989	3:90623434|90647086	circRNA_002057	8:9816450|9970770
circRNA_010125	X:61034499|61039735	circRNA_008578	11:6345844|6350185	circRNA_006850	10:62765488|62789150	circRNA_005074	9:22356187|22510615
circRNA_001310	2:93851810|93857047	circRNA_002664	1:202614698|202619041	circRNA_007950	7:102205505|102229168	circRNA_017073	3:86884387|87038982
circRNA_014720	6:90321035|90326273	circRNA_009333	3:19607424|19611768	circRNA_019856	9:128107257|128130921	circRNA_001244	2:49161621|49316282
circRNA_017251	8:12580305|12585547	circRNA_007363	17:49167454|49171800	circRNA_012352	10:29340480|29364146	circRNA_005687	13:77089983|77244716
circRNA_018490	1:203729744|203734986	circRNA_000908	15:51637238|51641585	circRNA_016971	18:10187954|10211621	circRNA_013701	18:59169585|59324563
circRNA_013401	8:89369835|89375078	circRNA_003751	17:15481218|15485565	circRNA_014106	1:86871594|86895269	circRNA_003415	14:104378120|104533235
circRNA_017602	13:97307290|97312533	circRNA_009609	6:11922509|11926858	circRNA_008178	9:139606828|139630507	circRNA_015395	11:42113634|42268766
circRNA_018219	2:30037235|30042478	circRNA_015065	GL893953.2:77588|81938	circRNA_003948	2:85774886|85798570	circRNA_012022	8:123634095|123789497
circRNA_001056	16:36914607|36919852	circRNA_012499	12:48106851|48111203	circRNA_018669	13:145052451|145076141	circRNA_018566	11:4191852|4347288
circRNA_005187	9:137428472|137433718	circRNA_013809	13:23908180|23912532	circRNA_020903	2:119185369|119209060	circRNA_019958	10:18525333|18681007
circRNA_014206	13:90755537|90760783	circRNA_014301	15:116468837|116473189	circRNA_018176	15:53153877|53177571	circRNA_006121	3:137626196|137781908
circRNA_011507	17:5309483|5314733	circRNA_018053	X:70837211|70841564	circRNA_019219	8:85707913|85731609	circRNA_004220	3:137626196|137781978
circRNA_012883	18:44790011|44795261	circRNA_005459	1:143833232|143837586	circRNA_006749	1:215885411|215909111	circRNA_015358	10:58751706|58907497
circRNA_011344	14:78267219|78272470	circRNA_007042	13:119253884|119258239	circRNA_015222	1:176789554|176813255	circRNA_006717	1:176208697|176364616
circRNA_016434	9:19338367|19343618	circRNA_008288	1:8311684|8316039	circRNA_015572	13:190926158|190949861	circRNA_020218	6:52501776|52657827
circRNA_017473	1:181041196|181046448	circRNA_001530	3:130917081|130921437	circRNA_016677	1:206962043|206985758	circRNA_011924	7:5331103|5487200
circRNA_017103	4:45282363|45287617	circRNA_002548	1:106924970|106929326	circRNA_002064	8:19705280|19729001	circRNA_007903	7:29196300|29352423
circRNA_019459	12:23410097|23415351	circRNA_008061	8:140973217|140977574	circRNA_003900	2:36302936|36326660	circRNA_014559	9:82893806|83049946
circRNA_019737	5:71597125|71602379	circRNA_009767	7:69725716|69730073	circRNA_006880	11:24782336|24806062	circRNA_004363	4:129336647|129492850
circRNA_005437	1:109565536|109570791	circRNA_006777	1:276560955|276565315	circRNA_016359	8:44836636|44860365	circRNA_002172	9:2455541|2612054
circRNA_016064	4:45188864|45194119	circRNA_002630	1:181028804|181033165	circRNA_014642	13:102280273|102304005	circRNA_020280	8:32498046|32654641
circRNA_019283	9:114852775|114858031	circRNA_020255	7:39684476|39688837	circRNA_001016	15:129615579|129639319	circRNA_017304	9:2455541|2612219
circRNA_005472	1:181325593|181330850	circRNA_012655	14:65185232|65189595	circRNA_014031	9:144775236|144798978	circRNA_017305	9:2455541|2612323
circRNA_010567	2:43970357|43975616	circRNA_014545	9:14197445|14201808	circRNA_012137	GL896504.1:17264|41007	circRNA_018067	1:16524475|16681535
circRNA_017271	8:42394633|42399892	circRNA_016567	JH118940.1:71062|75425	circRNA_007423	2:12792393|12816141	circRNA_013233	6:40223596|40381022
circRNA_020148	3:84809485|84814747	circRNA_005363	X:113783886|113788254	circRNA_001631	4:113204856|113228605	circRNA_018237	2:95823771|95981259
circRNA_002586	1:134612798|134618061	circRNA_015290	1:256850826|256855195	circRNA_020563	12:47804289|47828038	circRNA_019034	4:72458457|72615981
circRNA_014014	17:9407803|9413066	circRNA_018723	14:43975605|43979974	circRNA_007360	17:44388746|44412496	circRNA_004673	6:138456117|138614036
circRNA_013177	5:64068909|64074173	circRNA_006937	12:37716002|37720372	circRNA_006096	3:106700866|106724618	circRNA_000276	10:450816|608944
circRNA_010309	11:15979885|15985150	circRNA_005172	9:129664193|129668565	circRNA_018469	1:181031656|181055410	circRNA_016824	13:162690808|162849056
circRNA_008980	15:18318571|18323838	circRNA_011361	14:115744138|115748510	circRNA_013970	GL893593.1:2765|26520	circRNA_006260	6:40223596|40381892
circRNA_003384	14:78014861|78020135	circRNA_010098	MT:1739|6112	circRNA_001570	4:42006515|42030271	circRNA_002140	8:124794853|124953169
circRNA_004811	7:86333710|86338985	circRNA_019193	7:123617616|123621989	circRNA_011630	2:119260427|119284184	circRNA_016595	X:70495253|70653651
circRNA_004485	5:86854716|86859993	circRNA_007455	2:69768792|69773166	circRNA_010972	1:80305182|80328942	circRNA_014487	7:10317123|10475528

circRNA_002317	GL892397.1:109233|114511	circRNA_001036	16:23465932|23470307	circRNA_004104	3:72580187|72603949	circRNA_000911	15:54007386|54165794
circRNA_004482	5:83062597|83067876	circRNA_004091	3:56250670|56255045	circRNA_010514	16:78165123|78188885	circRNA_017107	4:49131198|49289682
circRNA_010804	7:91917856|91923135	circRNA_011739	3:135147245|135151621	circRNA_011124	10:59635863|59659625	circRNA_007407	18:52198463|52356954
circRNA_011006	1:141758092|141763372	circRNA_012129	GL895678.1:19441|23818	circRNA_018550	10:48125462|48149235	circRNA_006456	9:22356187|22514744
circRNA_000584	13:43420849|43426130	circRNA_013435	9:11747252|11751629	circRNA_014819	2:119205853|119229632	circRNA_008122	9:62188102|62346821
circRNA_012283	1:188021788|188027071	circRNA_006440	9:12465|16844	circRNA_003674	16:24935172|24958953	circRNA_001879	6:101779796|101938585
circRNA_016196	6:17199325|17204608	circRNA_009615	6:17199325|17203705	circRNA_019766	6:98777693|98801480	circRNA_000008	1:12075808|12234659
circRNA_009375	3:77625137|77630421	circRNA_017557	12:1625884|1630264	circRNA_016041	3:135140353|135164143	circRNA_001994	7:72211421|72370421
circRNA_009944	9:40653401|40658685	circRNA_020721	14:146744468|146748848	circRNA_003862	18:49455064|49478855	circRNA_000148	1:146138925|146297987
circRNA_015902	2:97492639|97497925	circRNA_004036	2:147049942|147054323	circRNA_008362	1:126926296|126950090	circRNA_004544	6:32224765|32383981
circRNA_017182	6:89210915|89216201	circRNA_003254	13:202649767|202654149	circRNA_014327	17:9730340|9754135	circRNA_001549	4:17093068|17252337
circRNA_017663	15:63845947|63851235	circRNA_019689	3:133992658|133997040	circRNA_012850	17:59415978|59439777	circRNA_006242	5:103598506|103757970
circRNA_009359	3:54745413|54750702	circRNA_000801	14:77056392|77060776	circRNA_004619	6:99060571|99084372	circRNA_018667	13:142522109|142682001
circRNA_015406	11:72144288|72149577	circRNA_017715	16:61137593|61141977	circRNA_008476	1:265189347|265213158	circRNA_012889	18:59324466|59484705
circRNA_019508	13:202691952|202697241	circRNA_000974	15:97987252|97991640	circRNA_017235	7:94509306|94533117	circRNA_000186	1:203916561|204076963
circRNA_018489	1:202606369|202611659	circRNA_021024	4:89003508|89007896	circRNA_009658	6:85260919|85284732	circRNA_018507	1:245505757|245666238
circRNA_003265	13:210782621|210787912	circRNA_011380	14:136306844|136311233	circRNA_019651	2:139046097|139069912	circRNA_002841	10:58751706|58912493
circRNA_011635	2:132259706|132264997	circRNA_015731	15:130321702|130326093	circRNA_006787	1:287936886|287960703	circRNA_010550	18:43963437|44124425
circRNA_015734	15:133537128|133542419	circRNA_012650	14:58594277|58598669	circRNA_000554	13:33160394|33184216	circRNA_014652	14:20942251|21103601
circRNA_012082	9:116263256|116268549	circRNA_014178	12:20322532|20326925	circRNA_007088	13:211131404|211155228	circRNA_000793	14:63568122|63729898
circRNA_015712	15:90006668|90011961	circRNA_016822	13:147692290|147696683	circRNA_014467	6:38271273|38295097	circRNA_014802	15:137822150|137984032
circRNA_020907	2:121334453|121339748	circRNA_017381	JH118743.1:14904|19297	circRNA_003583	15:93564278|93588108	circRNA_006512	9:148672107|148834317
circRNA_006074	3:49910032|49915628	circRNA_012694	14:131870012|131874408	circRNA_019201	8:32689706|32713541	circRNA_010782	7:10313309|10475528
circRNA_002205	9:24285558|24290855	circRNA_016231	6:86668305|86672701	circRNA_002028	7:102696376|102720215	circRNA_014696	3:118472317|118634611
circRNA_009082	16:23723018|23728315	circRNA_010789	7:53319728|53324125	circRNA_021026	4:92624449|92648299	circRNA_019083	5:85316246|85478798
circRNA_011213	13:35734591|35739889	circRNA_005089	9:29740807|29745207	circRNA_010825	8:20657917|20681770	circRNA_017823	4:17089767|17252337
circRNA_006107	3:114015538|114020837	circRNA_009298	2:128512404|128516806	circRNA_009280	2:108535329|108559190	circRNA_005004	8:124790578|124953169
circRNA_009466	4:71202236|71207535	circRNA_014828	3:47969752|47974155	circRNA_014163	10:62781970|62805834	circRNA_015123	15:111548704|111711408
circRNA_013058	3:126280689|126285991	circRNA_011270	13:146901535|146905943	circRNA_016472	9:70144680|70168548	circRNA_011937	7:38746540|38909427
circRNA_015049	7:124823524|124828826	circRNA_016578	X:21211868|21216280	circRNA_009374	3:76466177|76490049	circRNA_007193	14:136612215|136775595
circRNA_009142	17:36153160|36158466	circRNA_011891	6:88356874|88361287	circRNA_013591	X:115779067|115802942	circRNA_017402	X:111954804|112118218
circRNA_019621	2:35597336|35602642	circRNA_014416	4:42126976|42131389	circRNA_002704	1:243190928|243214804	circRNA_000610	13:86734228|86897820
circRNA_019499	13:155932248|155937561	circRNA_013273	6:125767057|125771471	circRNA_009669	6:92078907|92102784	circRNA_009275	2:92155128|92318938
circRNA_002346	GL894320.1:28024|33338	circRNA_001095	17:29783317|29787734	circRNA_007227	15:51612536|51636427	circRNA_019860	9:143721400|143885319
circRNA_003893	2:30338578|30343892	circRNA_012947	2:86065162|86069580	circRNA_012765	15:116646273|116670164	circRNA_005537	10:17976426|18140410
circRNA_003292	14:7197260|7202575	circRNA_008843	13:167207506|167211925	circRNA_014279	15:66048762|66072656	circRNA_007506	2:113404664|113568728
circRNA_007086	13:210034357|210039672	circRNA_006189	5:4779877|4784298	circRNA_003358	14:59110245|59134144	circRNA_018043	X:16108943|16273055
circRNA_014774	11:19657687|19663002	circRNA_018712	14:31282576|31286998	circRNA_004625	6:99216722|99240623	circRNA_017118	4:89590867|89755076
circRNA_018073	1:62685635|62690950	circRNA_000438	11:87126535|87130958	circRNA_017366	GL893841.1:45245|69147	circRNA_012554	13:79292174|79456725
circRNA_005862	15:112540074|112545392	circRNA_013744	1:187609942|187614366	circRNA_001942	7:82092|105997	circRNA_003661	16:21136449|21301351
circRNA_012941	2:80561214|80566532	circRNA_013170	5:50656496|50660921	circRNA_009551	5:59928308|59952213	circRNA_015418	12:12797259|12962192
circRNA_014433	4:117917205|117922524	circRNA_014815	2:17348985|17353410	circRNA_009338	3:24699762|24723668	circRNA_012955	2:91169756|91335022
circRNA_002318	GL892407.1:21562|26882	circRNA_004780	7:62884149|62888577	circRNA_016635	1:93363329|93387236	circRNA_019932	1:200015802|200181093
circRNA_007497	2:103773335|103778655	circRNA_010709	5:68113554|68117984	circRNA_006652	1:96782128|96806044	circRNA_019074	5:67204165|67369694
circRNA_019456	11:87125637|87130958	circRNA_015706	15:85064789|85069219	circRNA_010198	1:103206286|103230210	circRNA_013419	8:133875351|134040905
circRNA_006589	X:140033874|140039198	circRNA_012764	15:116646273|116650704	circRNA_001371	2:148139473|148163399	circRNA_002259	9:82893806|83059471
circRNA_010647	3:134654689|134660013	circRNA_005968	2:16095958|16100390	circRNA_000956	15:85340631|85364566	circRNA_012510	13:15593786|15759519
circRNA_004149	3:99288249|99293574	circRNA_005278	GL896440.1:2853|7286	circRNA_001260	2:60743119|60767058	circRNA_003869	18:59169585|59335593
circRNA_015024	2:78344472|78349798	circRNA_012993	3:11496127|11500560	circRNA_014614	1:250481322|250505262	circRNA_004360	4:129315285|129481379
circRNA_017000	2:8546796|8552123	circRNA_004558	6:64288025|64292459	circRNA_018324	6:148040878|148064823	circRNA_017447	1:105572600|105738705
circRNA_005156	9:115774313|115779641	circRNA_014442	5:17121476|17125911	circRNA_009306	2:140378789|140402736	circRNA_012739	15:87414896|87581136
circRNA_007577	3:92579061|92584389	circRNA_003329	14:32141473|32145909	circRNA_013368	8:4049479|4073427	circRNA_001909	6:138456117|138622445

circRNA_006183	4:129968066|129973398	circRNA_001617	4:102444968|102449406	circRNA_014375	2:131561222|131585171	circRNA_000223	1:249743639|249910066
circRNA_010329	12:23746349|23751682	circRNA_004497	5:93481383|93485824	circRNA_020034	14:46217501|46241450	circRNA_007036	13:104082327|104248782
circRNA_012621	14:25915299|25920635	circRNA_010976	1:84728132|84732574	circRNA_004715	7:12247873|12271827	circRNA_019759	6:73263962|73430424
circRNA_020777	16:23598429|23603765	circRNA_018203	18:9582225|9586667	circRNA_000172	1:200698016|200721973	circRNA_003510	15:44747209|44913761
circRNA_009177	18:10806735|10812072	circRNA_020676	14:41995768|42000211	circRNA_010629	3:91102645|91126604	circRNA_014903	1:985624|1152805
circRNA_021254	9:29830958|29836296	circRNA_010875	9:44289455|44293899	circRNA_013931	5:92237091|92261050	circRNA_001936	6:154791377|154958719
circRNA_002237	9:56547706|56553048	circRNA_019442	11:8732824|8737268	circRNA_015704	15:73492950|73516910	circRNA_018213	18:59169585|59336944
circRNA_017250	8:8754983|8760325	circRNA_013558	MT:2456|6901	circRNA_011311	14:21508995|21532972	circRNA_019942	1:254769627|254937077
circRNA_019104	6:74110044|74115386	circRNA_010438	14:60133197|60137643	circRNA_004290	4:72772834|72796823	circRNA_019348	X:86326323|86494087
circRNA_019417	1:302073496|302078838	circRNA_011672	3:40969228|40973674	circRNA_016128	5:16681220|16705209	circRNA_015652	14:95077720|95245531
circRNA_020422	1:157867647|157872989	circRNA_009869	8:92844853|92849300	circRNA_011850	5:103774497|103798487	circRNA_008465	1:249743639|249911706
circRNA_000152	1:154901709|154907052	circRNA_000422	11:56142026|56146474	circRNA_017811	3:128289492|128313490	circRNA_004221	3:137626196|137794989
circRNA_012595	13:156897027|156902372	circRNA_006145	4:51356571|51361020	circRNA_009647	6:73359648|73383647	circRNA_019030	4:67851823|68020671
circRNA_020830	18:11257880|11263226	circRNA_007136	14:60727790|60732239	circRNA_005941	18:14762483|14786488	circRNA_010133	X:70495253|70664153
circRNA_008792	13:90954665|90960012	circRNA_006844	10:54171547|54175997	circRNA_001120	17:49147794|49171800	circRNA_016987	18:24946650|25115893
circRNA_018837	16:77916180|77921527	circRNA_011400	15:54870904|54875355	circRNA_009744	7:43157539|43181547	circRNA_010004	9:113288178|113457461
circRNA_007320	16:47180663|47186013	circRNA_003146	13:81190634|81195086	circRNA_012315	1:265966523|265990532	circRNA_015506	13:45820382|45989708
circRNA_017661	15:53217508|53222860	circRNA_019667	3:29618701|29623155	circRNA_002032	7:109744938|109768951	circRNA_013238	6:43583696|43753125
circRNA_020478	1:282201505|282206858	circRNA_003512	15:51629686|51634142	circRNA_013208	5:101608532|101632551	circRNA_006513	9:150229063|150398500
circRNA_000528	13:4374208|4379562	circRNA_011842	5:92238775|92243231	circRNA_010150	X:109061119|109085139	circRNA_014427	4:72446101|72615981
circRNA_016219	6:71591510|71596865	circRNA_017067	3:45557522|45561978	circRNA_001239	2:41866547|41890569	circRNA_015298	1:271529541|271699437
circRNA_011518	17:41844789|41850145	circRNA_016760	12:35930283|35934746	circRNA_009057	15:124765665|124789687	circRNA_015900	2:92155128|92325227
circRNA_012323	1:284738023|284743379	circRNA_018950	2:142544228|142548691	circRNA_000533	13:18323062|18347086	circRNA_000131	1:141824210|141994380
circRNA_018145	13:206959475|206964833	circRNA_005717	13:140985332|140989796	circRNA_008263	X:85919732|85943763	circRNA_002255	9:81818339|81988509
circRNA_007534	3:16409127|16414487	circRNA_006939	12:39225286|39229750	circRNA_016127	5:15620434|15644469	circRNA_004498	5:93592779|93763194
circRNA_019157	7:53603281|53608643	circRNA_006439	8:146911444|146915909	circRNA_010205	1:134651078|134675125	circRNA_013098	4:61776537|61946991
circRNA_014738	9:13614978|13620342	circRNA_012365	10:45220144|45224612	circRNA_012107	GL893095.2:34178|58246	circRNA_001443	3:75528674|75699282
circRNA_012492	12:40666195|40671560	circRNA_009911	9:22715|27187	circRNA_014572	9:140018765|140042838	circRNA_001629	4:110255189|110425808
circRNA_001003	15:118292366|118297732	circRNA_010295	10:61461861|61466336	circRNA_006911	12:13937086|13961162	circRNA_008136	9:81817887|81988509
circRNA_000439	12:151796|157165	circRNA_014115	1:132148098|132152573	circRNA_004244	4:34617495|34641574	circRNA_004338	4:110255147|110425808
circRNA_004382	4:136425709|136431078	circRNA_021300	GL892594.2:38122|42597	circRNA_009958	9:44922267|44946346	circRNA_012076	9:102437613|102608569
circRNA_000650	13:131008844|131014214	circRNA_002330	GL893171.1:18281|22757	circRNA_007723	5:16670864|16694947	circRNA_009723	7:10304180|10475528
circRNA_016787	13:38222884|38228255	circRNA_005454	1:141274414|141278890	circRNA_007724	5:16670864|16694950	circRNA_009167	18:5252251|5423652
circRNA_008724	13:26317162|26322534	circRNA_007728	5:17107980|17112458	circRNA_015369	11:5274152|5298238	circRNA_004361	4:129315285|129486702
circRNA_008618	11:69830374|69835750	circRNA_013804	12:44376919|44381399	circRNA_006516	GL892680.2:154968|179057	circRNA_007812	6:44589851|44761408
circRNA_011826	5:68122687|68128063	circRNA_016876	14:122994227|122998707	circRNA_001160	18:15010668|15034759	circRNA_000130	1:141822796|141994380
circRNA_016340	8:10479631|10485008	circRNA_018439	1:127637224|127641705	circRNA_020012	13:133439605|133463697	circRNA_014380	3:12224076|12395931
circRNA_021373	X:71003031|71008408	circRNA_012104	GL893077.1:45934|50416	circRNA_002902	11:21795724|21819817	circRNA_009441	4:35890687|36062940
circRNA_008487	1:283077826|283083205	circRNA_003366	14:63365860|63370343	circRNA_010655	4:34617495|34641595	circRNA_010293	10:58751706|58923959
circRNA_006427	8:109416257|109421638	circRNA_006504	9:125610011|125614494	circRNA_015554	13:146716493|146740593	circRNA_020104	18:43942640|44115219
circRNA_010310	11:18665770|18671153	circRNA_012337	10:11677711|11682194	circRNA_003591	15:104515243|104539355	circRNA_020132	3:39133909|39306568
circRNA_018567	11:5188534|5193917	circRNA_010858	8:144704211|144708695	circRNA_006034	2:121986386|122010500	circRNA_018300	6:3299821|3472494
circRNA_003491	15:8565341|8570725	circRNA_011712	3:95543189|95547673	circRNA_006079	3:72556230|72580346	circRNA_001590	4:68314313|68487502
circRNA_009805	7:116970559|116975944	circRNA_015618	14:58819704|58824188	circRNA_021031	4:117893543|117917659	circRNA_006750	1:217832622|218005872
circRNA_016102	4:117802095|117807480	circRNA_016939	16:42288019|42292505	circRNA_011259	13:132975613|132999736	circRNA_002884	11:10887979|11061383
circRNA_016600	X:90783736|90789121	circRNA_014184	12:56179696|56184183	circRNA_011723	3:110477015|110501138	circRNA_005538	10:17976426|18149975
circRNA_013738	9:115694105|115699492	circRNA_003592	15:105377422|105381910	circRNA_004443	5:50595002|50619132	circRNA_019953	1:297746361|297920099
circRNA_018934	2:112484489|112489877	circRNA_006904	12:4644826|4649314	circRNA_014062	GL894943.1:12044|36174	circRNA_004362	4:129319056|129492850
circRNA_020082	16:56970111|56975499	circRNA_010342	12:44782366|44786854	circRNA_016413	8:143282889|143307020	circRNA_002254	9:81814473|81988509
circRNA_003100	13:35085945|35091334	circRNA_002913	11:26425715|26430206	circRNA_019781	6:154758636|154782770	circRNA_020584	13:40544513|40718631
circRNA_003671	16:23686581|23691973	circRNA_021195	8:40467324|40471815	circRNA_001744	5:73490736|73514893	circRNA_000967	15:89948304|90122644
circRNA_018268	4:88588344|88593736	circRNA_011736	3:128289492|128293984	circRNA_016585	X:41433204|41457363	circRNA_002709	1:249743639|249918475

circRNA_019649	2:134977702|134983095	circRNA_019194	7:124952565|124957057	circRNA_000383	11:11212642|11236804	circRNA_014938	5:51567416|51742379
circRNA_015793	17:20703157|20708552	circRNA_017223	7:60799836|60804330	circRNA_010292	10:58742098|58766266	circRNA_004720	7:14247016|14422083
circRNA_018212	18:53779233|53784629	circRNA_005939	18:10874962|10879457	circRNA_001307	2:91905341|91929512	circRNA_006817	10:21572992|21748095
circRNA_001428	3:56860581|56865978	circRNA_013942	6:150870199|150874694	circRNA_014791	14:34693433|34717605	circRNA_012964	2:118253397|118428523
circRNA_002731	1:267412457|267417857	circRNA_001436	3:72422874|72427371	circRNA_014653	14:34693427|34717605	circRNA_017820	4:15522028|15697154
circRNA_005336	X:53122424|53127827	circRNA_003304	14:14197504|14202002	circRNA_003195	13:118705600|118729779	circRNA_015209	1:128023703|128198830
circRNA_011065	1:278921973|278927376	circRNA_015878	2:45070552|45075050	circRNA_011418	15:85380889|85405068	circRNA_001022	15:137808684|137984032
circRNA_014862	7:73282517|73287920	circRNA_000442	12:3324833|3329332	circRNA_011588	2:48842971|48867151	circRNA_005677	13:57974186|58149818
circRNA_012991	3:6065347|6070751	circRNA_021147	7:14645264|14649763	circRNA_005833	15:36536596|36560784	circRNA_017960	8:83876690|84052387
circRNA_001700	5:35894045|35899452	circRNA_009981	9:78740877|78745378	circRNA_019135	6:144709992|144734185	circRNA_009439	4:34441658|34617596
circRNA_014183	12:51985203|51990610	circRNA_000821	14:83828649|83833151	circRNA_016174	5:87303583|87327780	circRNA_001431	3:61149766|61326049
circRNA_020612	13:103784117|103789525	circRNA_002101	8:77450887|77455389	circRNA_009053	15:120966272|120990471	circRNA_000039	1:37369084|37545495
circRNA_019360	1:29141351|29146761	circRNA_011040	1:218647971|218652474	circRNA_009585	5:88028715|88052914	circRNA_000595	13:72821916|72998456
circRNA_007649	4:45567652|45573063	circRNA_017404	X:115991642|115996147	circRNA_000871	14:145557672|145581876	circRNA_008448	1:229799248|229975830
circRNA_010323	12:13030858|13036271	circRNA_020558	12:37420987|37425492	circRNA_016610	X:127302637|127326842	circRNA_000966	15:89945975|90122644
circRNA_011365	14:116401067|116406483	circRNA_017989	9:50856194|50860700	circRNA_002610	1:145462507|145486713	circRNA_006566	X:56693856|56870525
circRNA_008437	1:208343617|208349034	circRNA_005229	GL893319.1:1791|6298	circRNA_020633	13:144085719|144109931	circRNA_009901	8:139497435|139674620
circRNA_012622	14:27779206|27784623	circRNA_012110	GL893374.2:3775|8283	circRNA_009134	17:14861638|14885851	circRNA_012807	16:41762187|41939412
circRNA_021315	GL894315.1:49672|55092	circRNA_016512	GL892680.2:178981|183490	circRNA_015436	12:23727465|23751682	circRNA_001442	3:75521914|75699282
circRNA_002013	7:92763263|92768684	circRNA_009047	15:117726405|117730915	circRNA_004645	6:108959030|108983252	circRNA_000594	13:72820933|72998456
circRNA_008464	1:245565427|245570849	circRNA_021331	GL895430.1:677|5187	circRNA_013185	5:77354899|77379122	circRNA_001650	4:129315285|129492850
circRNA_019461	12:37420069|37425492	circRNA_020381	1:60892775|60897286	circRNA_014901	X:126963525|126987749	circRNA_013615	7:10297937|10475528
circRNA_007280	15:120985046|120990471	circRNA_002227	9:44920845|44925357	circRNA_012901	2:12793656|12817882	circRNA_016159	5:70515829|70693708
circRNA_017669	15:85031042|85036467	circRNA_014666	15:103657066|103661579	circRNA_020975	3:92584231|92608458	circRNA_000702	13:202601098|202779045
circRNA_016543	GL895413.1:1369|6795	circRNA_015898	2:89030620|89035133	circRNA_010817	7:134411388|134435617	circRNA_004959	8:82828282|83006817
circRNA_007242	15:66305630|66311057	circRNA_005926	17:40696156|40700671	circRNA_005979	2:52803495|52827726	circRNA_001765	5:93584635|93763194
circRNA_018923	2:83895486|83900913	circRNA_012936	2:75687401|75691918	circRNA_019011	3:126256536|126280769	circRNA_009185	18:16661859|16840736
circRNA_004015	2:134977667|134983095	circRNA_009331	3:18516901|18521420	circRNA_002758	1:297536723|297560957	circRNA_003962	2:87624115|87803029
circRNA_019349	X:90783693|90789121	circRNA_000820	14:83540591|83545111	circRNA_015103	8:71459662|71483897	circRNA_006322	7:16831954|17011103
circRNA_009844	8:43039144|43044573	circRNA_005246	GL894597.1:22475|26995	circRNA_001185	18:42650214|42674455	circRNA_001276	2:78532006|78711160
circRNA_015843	18:44791057|44796486	circRNA_010978	1:87186695|87191215	circRNA_013125	4:116997042|117021283	circRNA_001544	4:10938078|11117660
circRNA_020311	9:31044963|31050392	circRNA_020707	14:120352081|120356604	circRNA_001053	16:36895597|36919852	circRNA_006270	6:73357016|73536700
circRNA_000538	13:21209687|21215121	circRNA_007745	5:46852469|46856993	circRNA_002491	1:38556810|38581068	circRNA_004260	4:41064761|41244579
circRNA_007862	6:125781000|125786434	circRNA_013200	5:92243038|92247562	circRNA_013224	6:25280812|25305075	circRNA_007979	8:4207014|4386964
circRNA_011620	2:104498556|104503990	circRNA_018820	16:23719895|23724419	circRNA_010007	9:114861767|114886040	circRNA_001103	17:42526614|42706643
circRNA_004457	5:70582741|70588176	circRNA_005798	14:107971717|107976245	circRNA_010473	15:65947361|65971654	circRNA_007438	2:47522256|47702515
circRNA_014776	12:32299469|32304905	circRNA_020328	9:102762643|102767173	circRNA_008046	8:110767547|110791848	circRNA_013074	4:15714361|15894635
circRNA_016168	5:85963066|85968506	circRNA_003017	12:40118868|40123399	circRNA_008256	X:70474455|70498760	circRNA_020121	2:118253397|118433729
circRNA_003810	18:9603361|9608802	circRNA_017603	13:97310339|97314870	circRNA_010115	X:40479559|40503864	circRNA_009206	18:57436709|57617076
circRNA_005688	13:78980508|78985950	circRNA_010497	15:124662048|124666583	circRNA_021354	X:14455714|14480020	circRNA_002136	8:123713176|123893742
circRNA_018597	12:32299469|32304911	circRNA_006778	1:280497623|280502159	circRNA_007122	14:48100016|48124333	circRNA_014363	2:84438188|84619391
circRNA_013527	GL894031.1:207260|212705	circRNA_012080	9:116098827|116103363	circRNA_018135	13:138031949|138056269	circRNA_015219	1:152171873|152353082
circRNA_016862	14:82889383|82894828	circRNA_019225	8:93218969|93223505	circRNA_018164	14:113241615|113265936	circRNA_000151	1:152171873|152353086
circRNA_003659	16:18673613|18679059	circRNA_019131	6:137844860|137849397	circRNA_021095	6:23732839|23757166	circRNA_011689	3:67119328|67300623
circRNA_004029	2:142518692|142524139	circRNA_004897	8:32691009|32695547	circRNA_008894	14:34705432|34729763	circRNA_015158	1:37322545|37503843
circRNA_019115	6:92749934|92755383	circRNA_010313	11:22558053|22562591	circRNA_008184	GL892381.1:50931|75265	circRNA_017523	10:46597762|46779066
circRNA_004738	7:26960012|26965463	circRNA_019656	2:142592917|142597455	circRNA_002083	8:41183979|41208316	circRNA_000332	10:46597662|46779066
circRNA_008212	GL894927.1:26706|32157	circRNA_000242	1:270471815|270476354	circRNA_015392	11:26280706|26305046	circRNA_001118	17:47358184|47539848
circRNA_009373	3:76077846|76083297	circRNA_006798	1:310030060|310034599	circRNA_000238	1:270329795|270354137	circRNA_014516	8:11361730|11543409
circRNA_010437	14:60123994|60129445	circRNA_008911	14:59632979|59637522	circRNA_005237	GL893884.1:23405|47747	circRNA_000906	15:50970696|51152444
circRNA_012532	13:43115175|43120627	circRNA_011033	1:200334067|200338610	circRNA_020013	13:137317424|137341766	circRNA_001237	2:39869318|40051068
circRNA_018169	14:132247177|132252629	circRNA_015948	3:10316443|10320986	circRNA_013510	GL892628.1:24048|48395	circRNA_011476	16:27729400|27911180

circRNA_006354	7:91959137|91964590	circRNA_009945	9:40763445|40767990	circRNA_018930	2:91881042|91905389	circRNA_018887	18:43942640|44124425
circRNA_015770	16:51778584|51784038	circRNA_016436	9:22403235|22407781	circRNA_008412	1:181145447|181169796	circRNA_002137	8:123713176|123895142
circRNA_016681	1:230081272|230086726	circRNA_005060	9:12986704|12991251	circRNA_011342	14:77561976|77586330	circRNA_002135	8:123711620|123893742
circRNA_004122	3:83072793|83078248	circRNA_011935	7:36582514|36587062	circRNA_004048	2:157822105|157846464	circRNA_018529	1:300208622|300390846
circRNA_019793	7:39245483|39250939	circRNA_009813	7:128918056|128922605	circRNA_002808	10:37017761|37042122	circRNA_017088	3:117786132|117968462
circRNA_000165	1:195169049|195174506	circRNA_012973	2:140473312|140477861	circRNA_011336	14:65832702|65857064	circRNA_009380	3:84139238|84321910
circRNA_015490	13:34853744|34859202	circRNA_002025	7:100799295|100803845	circRNA_021279	9:98624238|98648601	circRNA_015055	8:114724143|114906853
circRNA_020702	14:110489135|110494594	circRNA_004294	4:72916723|72921273	circRNA_002148	8:134355181|134379557	circRNA_010500	15:146627679|146810404
circRNA_019373	1:124856766|124862227	circRNA_004556	6:57239931|57244482	circRNA_003163	13:86966559|86990937	circRNA_020766	15:150059437|150242212
circRNA_017187	6:99130075|99135537	circRNA_015054	8:73313186|73317737	circRNA_011067	1:282182480|282206858	circRNA_014007	14:95077720|95260651
circRNA_018869	18:19800728|19806190	circRNA_000096	1:124982100|124986652	circRNA_009060	15:133515011|133539392	circRNA_011107	10:46596019|46779066
circRNA_019120	6:108049955|108055419	circRNA_002387	JH118886.1:85067|89619	circRNA_005140	9:82869539|82893921	circRNA_015279	1:231976626|232159897
circRNA_007921	7:63633654|63639120	circRNA_018714	14:33137182|33141734	circRNA_002685	1:218551332|218575722	circRNA_015879	2:45928640|46111970
circRNA_001081	17:728755|734222	circRNA_008718	13:20986223|20990776	circRNA_012792	16:22192703|22217102	circRNA_012831	17:15555715|15739103
circRNA_020883	2:74686457|74691924	circRNA_005789	14:77581776|77586330	circRNA_001322	2:117898216|117922618	circRNA_005001	8:123711620|123895142
circRNA_012015	8:103910915|103916383	circRNA_006407	8:73274904|73279459	circRNA_018014	9:150330175|150354580	circRNA_013641	7:10291894|10475528
circRNA_000471	12:26840115|26845587	circRNA_016376	8:73274904|73279463	circRNA_021233	8:138513823|138538228	circRNA_004529	6:14292517|14476162
circRNA_003487	15:7974905|7980378	circRNA_004552	6:41572044|41576604	circRNA_004885	8:17042628|17067041	circRNA_006051	3:3092717|3276364
circRNA_016955	17:23453956|23459431	circRNA_000809	14:78015574|78020135	circRNA_002406	X:3659514|3683935	circRNA_002283	9:119027170|119211078
circRNA_012142	JH118624.1:89567|95044	circRNA_011310	14:14864654|14869215	circRNA_006092	3:92608281|92632715	circRNA_006715	1:173582741|173767461
circRNA_003408	14:94770180|94775658	circRNA_018079	1:97373715|97378276	circRNA_012582	13:131008844|131033279	circRNA_012165	X:111422724|111607531
circRNA_014082	14:31863914|31869392	circRNA_004980	8:93236367|93240931	circRNA_005947	18:18661914|18686351	circRNA_012096	9:141916855|142101684
circRNA_010549	18:42190501|42195980	circRNA_013044	3:106710521|106715085	circRNA_011345	14:81015098|81039544	circRNA_007244	15:68610560|68795464
circRNA_003520	15:53217508|53222989	circRNA_002962	12:14670745|14675311	circRNA_006388	8:29273357|29297807	circRNA_010605	3:3091458|3276364
circRNA_009927	9:22402298|22407781	circRNA_001263	2:64601109|64605676	circRNA_019020	4:31023893|31048345	circRNA_018497	1:226163141|226348087
circRNA_002402	MT:2824|8308	circRNA_021084	5:107182209|107186776	circRNA_014491	7:57815439|57839892	circRNA_013047	3:110292158|110477188
circRNA_017791	3:46256147|46261633	circRNA_002146	8:133762122|133766690	circRNA_000994	15:115990305|116014762	circRNA_016953	17:13616684|13801776
circRNA_008093	9:25924923|25930412	circRNA_005530	1:299815881|299820449	circRNA_016389	8:103675822|103700283	circRNA_016940	16:47303095|47488722
circRNA_010256	1:301529706|301535195	circRNA_019254	9:36274619|36279187	circRNA_012027	8:141440630|141465095	circRNA_010697	5:32847751|33033688
circRNA_013438	9:13023046|13028536	circRNA_021033	4:129654725|129659296	circRNA_001282	2:82132977|82157445	circRNA_011792	4:129295358|129481379
circRNA_002718	1:254931586|254937077	circRNA_011434	15:112472251|112476823	circRNA_002619	1:154199344|154223816	circRNA_019991	13:28131575|28317612
circRNA_004899	8:32705476|32710967	circRNA_003725	16:54857645|54862220	circRNA_009372	3:74739616|74764088	circRNA_000571	13:36292241|36478554
circRNA_011648	2:149781429|149786920	circRNA_008117	9:51073473|51078048	circRNA_001472	3:101033374|101057850	circRNA_015497	13:36292237|36478554
circRNA_019601	17:57173869|57179361	circRNA_002163	8:144763071|144767647	circRNA_006995	13:44062045|44086522	circRNA_001268	2:72670686|72857035
circRNA_000972	15:96079624|96085117	circRNA_019564	15:61350519|61355095	circRNA_016714	10:15003526|15028004	circRNA_001522	3:123000541|123186934
circRNA_008033	8:89361362|89366855	circRNA_012652	14:59554083|59558660	circRNA_006746	1:212980811|213005294	circRNA_015848	18:59149023|59335593
circRNA_009808	7:117248697|117254190	circRNA_011987	8:3341344|3345924	circRNA_003061	13:3089207|3113691	circRNA_017821	4:15707963|15894635
circRNA_008023	8:75573570|75579064	circRNA_019805	7:102244418|102248998	circRNA_015923	2:128801546|128826032	circRNA_020930	2:156478928|156666029
circRNA_016880	14:135913412|135918906	circRNA_007383	18:10898787|10903368	circRNA_016789	13:42295832|42320321	circRNA_020217	6:52470228|52657827
circRNA_011261	13:140189867|140195362	circRNA_009323	2:156478928|156483509	circRNA_005710	13:126801546|126826040	circRNA_018002	9:110559561|110747415
circRNA_007871	6:136013083|136018579	circRNA_011914	6:148034940|148039521	circRNA_007642	4:39835278|39859777	circRNA_019139	6:148924023|149112188
circRNA_014676	2:15680718|15686214	circRNA_016548	GL896056.1:12756|17337	circRNA_013647	9:51132612|51157112	circRNA_018197	17:13778285|13966843
circRNA_000678	13:150586617|150592114	circRNA_020489	1:305204959|305209542	circRNA_007384	18:14695794|14720296	circRNA_019717	5:19353117|19541808
circRNA_009049	15:117903370|117908868	circRNA_000618	13:90075586|90080170	circRNA_008693	12:61592325|61616828	circRNA_018177	15:68606291|68795464
circRNA_007432	2:30750436|30755936	circRNA_003257	13:205664917|205669502	circRNA_006497	9:115681075|115705584	circRNA_018910	2:54101961|54291281
circRNA_013344	7:97836215|97841717	circRNA_001801	6:48351537|48356123	circRNA_004728	7:21032268|21056778	circRNA_017283	8:82954008|83143453
circRNA_006108	3:114039113|114044616	circRNA_006941	12:39721598|39726184	circRNA_004339	4:113269353|113293869	circRNA_015774	16:52742120|52931695
circRNA_006528	GL894055.2:32769|38272	circRNA_002476	1:23215715|23220303	circRNA_020570	13:2382115|2406634	circRNA_020587	13:42997356|43186998
circRNA_014013	16:29110372|29115875	circRNA_011923	7:2466501|2471089	circRNA_012985	2:156366504|156391029	circRNA_015840	18:43942640|44132284
circRNA_009090	16:29110372|29115878	circRNA_000940	15:73569195|73573786	circRNA_018077	1:84754696|84779222	circRNA_017507	1:300208622|300398288
circRNA_000710	13:210039530|210045037	circRNA_011145	11:69873672|69878264	circRNA_018515	1:265966005|265990532	circRNA_013171	5:51910032|52100360
circRNA_020762	15:117907981|117913489	circRNA_014566	9:116101588|116106183	circRNA_018850	17:43030480|43055022	circRNA_004359	4:129302194|129492850

circRNA_010093	JH118963.1:53673|59182	circRNA_020496	10:14513332|14517927	circRNA_010236	1:227101708|227126254	circRNA_019289	9:124334896|124525558
circRNA_000782	14:59040543|59046053	circRNA_014902	X:140034602|140039198	circRNA_007190	14:135600858|135625410	circRNA_008939	14:95061035|95251819
circRNA_006696	1:145031571|145037081	circRNA_009637	6:64286222|64290820	circRNA_017298	8:137647387|137671945	circRNA_005901	16:52742120|52933109
circRNA_003264	13:210044909|210050422	circRNA_012758	15:116078917|116083516	circRNA_017224	7:61636840|61661399	circRNA_001377	3:3470907|3662413
circRNA_007709	4:143299843|143305357	circRNA_015641	14:79067119|79071718	circRNA_020915	2:128789190|128813749	circRNA_005131	9:77278140|77469954
circRNA_003262	13:210039519|210045037	circRNA_016663	1:179194086|179198685	circRNA_009770	7:73042261|73066822	circRNA_016073	4:67851823|68043681
circRNA_016380	8:82565568|82571086	circRNA_013093	4:44536790|44541391	circRNA_015064	GL892680.2:158928|183490	circRNA_000291	10:17976426|18168555
circRNA_000784	14:59074186|59079705	circRNA_021272	9:78745146|78749748	circRNA_005662	13:34693141|34717707	circRNA_007159	14:95061035|95253340
circRNA_003966	2:89577917|89583437	circRNA_020636	13:148398444|148403047	circRNA_002345	GL894261.2:66477|91044	circRNA_001876	6:101745126|101938585
circRNA_007087	13:210036903|210042423	circRNA_011026	1:181400580|181405184	circRNA_008593	11:19712163|19736732	circRNA_015112	1:37316187|37509843
circRNA_014129	1:202650920|202656440	circRNA_002314	GL892332.2:318148|322754	circRNA_002604	1:143396359|143420938	circRNA_012310	1:254743246|254937077
circRNA_006157	4:90623622|90629143	circRNA_003071	13:18743182|18747789	circRNA_016790	13:43394660|43419245	circRNA_001886	6:107633401|107827270
circRNA_013262	6:98736019|98741540	circRNA_006529	GL894597.1:35377|39985	circRNA_015037	5:50996616|51021202	circRNA_007925	7:71192296|71386403
circRNA_008188	GL893011.1:27745|33267	circRNA_017121	4:107275821|107280430	circRNA_007350	17:36480170|36504762	circRNA_012098	9:150229063|150423880
circRNA_007895	7:159104|164628	circRNA_006603	1:14099143|14103753	circRNA_005124	9:62322223|62346821	circRNA_000841	14:114079542|114274829
circRNA_008859	13:210042276|210047801	circRNA_004681	6:145356549|145361162	circRNA_013087	4:41090877|41115480	circRNA_004696	6:147566043|147761690
circRNA_011641	2:142520859|142526384	circRNA_015381	11:22173482|22178095	circRNA_019249	9:13674798|13699408	circRNA_014657	14:95077720|95273617
circRNA_006345	7:72364895|72370421	circRNA_005234	GL893655.1:50527|55141	circRNA_017427	1:45713269|45737883	circRNA_007116	14:42167264|42363300
circRNA_018677	13:157180910|157186436	circRNA_020953	3:45711563|45716178	circRNA_001877	6:101779796|101804423	circRNA_008990	15:34935787|35132183
circRNA_011901	6:118042636|118048164	circRNA_003644	15:133539228|133543845	circRNA_006003	2:86079351|86103979	circRNA_002589	1:135303493|135500052
circRNA_001944	7:2429518|2435049	circRNA_005422	1:59739762|59744379	circRNA_012685	14:117822762|117847392	circRNA_009414	3:122990300|123186934
circRNA_004001	2:120022510|120028042	circRNA_012173	X:139925204|139929821	circRNA_006622	1:29058116|29082752	circRNA_002590	1:135303493|135500271
circRNA_008171	9:132289659|132295195	circRNA_013940	6:146123459|146128074	circRNA_005871	15:121417853|121442490	circRNA_016626	1:29403196|29600145
circRNA_010178	1:59707496|59713035	circRNA_002660	1:201225539|201230161	circRNA_006594	1:2789914|2814551	circRNA_013967	9:143721400|143918357
circRNA_011428	15:97956675|97962214	circRNA_002417	X:21221667|21226291	circRNA_005339	X:63569820|63594470	circRNA_006188	5:3779360|3976461
circRNA_021041	5:4589216|4594756	circRNA_020000	13:43142769|43147393	circRNA_003647	15:145589555|145614211	circRNA_020716	14:136612215|136809619
circRNA_003766	17:36448992|36454536	circRNA_002353	GL894772.1:18966|23591	circRNA_020914	2:125069347|125094005	circRNA_015523	13:86473669|86671223
circRNA_020221	6:74975291|74980836	circRNA_010958	1:21865210|21869835	circRNA_001624	4:106709011|106733675	circRNA_014939	5:51567416|51765461
circRNA_004214	3:135203818|135209364	circRNA_011864	6:13110937|13115563	circRNA_011308	14:14224732|14249401	circRNA_014141	1:254738972|254937077
circRNA_002451	X:139963482|139969029	circRNA_009962	9:45843608|45848235	circRNA_015586	13:216655489|216680164	circRNA_003199	13:120826360|121025438
circRNA_018561	11:112280|117832	circRNA_006612	1:21865210|21869839	circRNA_019092	6:23732487|23757166	circRNA_000603	13:77089983|77289727
circRNA_011532	17:59958882|59964436	circRNA_010928	X:12690941|12695570	circRNA_016421	8:146891228|146915909	circRNA_013653	4:34441658|34641574
circRNA_020436	1:182248035|182253589	circRNA_006378	7:130882992|130887622	circRNA_011678	3:45588646|45613329	circRNA_013863	16:27711139|27911180
circRNA_016117	4:137176115|137181671	circRNA_010149	X:109061119|109065749	circRNA_009553	5:61337118|61361802	circRNA_019562	15:38071423|38271793
circRNA_006222	5:80679719|80685276	circRNA_012896	2:504157|508787	circRNA_005557	10:48048771|48073466	circRNA_001273	2:76805870|77006314
circRNA_012504	12:62913559|62919117	circRNA_019658	2:145884681|145889313	circRNA_004067	3:23052402|23077100	circRNA_006623	1:29399686|29600145
circRNA_020876	2:60047801|60053359	circRNA_010380	13:86594955|86599588	circRNA_006482	9:78882583|78907286	circRNA_013684	13:116036816|116237469
circRNA_017161	6:14363834|14369395	circRNA_002924	11:53778989|53783624	circRNA_004565	6:69705462|69730166	circRNA_011364	14:115931100|116131769
circRNA_006500	9:117723725|117729287	circRNA_007442	2:50799143|50803783	circRNA_010535	18:1942538|1967245	circRNA_007507	2:118253397|118454133
circRNA_020958	3:55082265|55087827	circRNA_001084	17:9749494|9754135	circRNA_011138	11:21081332|21106042	circRNA_004658	6:127017993|127218750
circRNA_007650	4:51266360|51271923	circRNA_008905	14:51751565|51756206	circRNA_012742	15:97032573|97057285	circRNA_004588	6:82706661|82907607
circRNA_001399	3:29691348|29696912	circRNA_015073	1:143237581|143242222	circRNA_001589	4:67947307|67972035	circRNA_018166	14:115928981|116129972
circRNA_006600	1:9392359|9397924	circRNA_016870	14:113982858|113987500	circRNA_012221	1:84935055|84959783	circRNA_020800	17:29720|231216
circRNA_003377	14:77567920|77573487	circRNA_014817	2:82132977|82137621	circRNA_000452	12:16046492|16071225	circRNA_021372	X:70438343|70639982
circRNA_011430	15:101683537|101689104	circRNA_017268	8:40115524|40120168	circRNA_018746	14:81977600|82002333	circRNA_001772	5:108030113|108231765
circRNA_016047	4:15229913|15235480	circRNA_019774	6:143684715|143689360	circRNA_005058	9:11747252|11771988	circRNA_019482	13:57893409|58095301
circRNA_001246	2:50800152|50805720	circRNA_019779	6:148072394|148077039	circRNA_001288	2:86260574|86285313	circRNA_020487	1:299949670|300151730
circRNA_006293	6:112448404|112453978	circRNA_019672	3:46305365|46310011	circRNA_005685	13:75379989|75404735	circRNA_019096	6:32419351|32621437
circRNA_016945	16:51797704|51803278	circRNA_010418	13:212995887|213000535	circRNA_002292	9:129708161|129732908	circRNA_000502	12:49600053|49802316
circRNA_006959	13:2730329|2735905	circRNA_009066	15:151463142|151467791	circRNA_001306	2:91206107|91230855	circRNA_012009	8:82828282|83030613
circRNA_007464	2:79260572|79266148	circRNA_010151	X:116234276|116238925	circRNA_004297	4:73760914|73785662	circRNA_020519	11:129136|331862
circRNA_010220	1:183970965|183976541	circRNA_020472	1:266578997|266583649	circRNA_011049	1:242785952|242810712	circRNA_020882	2:66950440|67153172

circRNA_008321	1:59707496|59713073	circRNA_000221	1:248853148|248857802	circRNA_020960	3:58923020|58947781	circRNA_005495	1:211957500|212160381
circRNA_001569	4:42006515|42012095	circRNA_002113	8:88019043|88023699	circRNA_009706	6:146044580|146069344	circRNA_000501	12:49599112|49802316
circRNA_021197	8:43550245|43555826	circRNA_004502	5:97681889|97686545	circRNA_006859	11:4045654|4070420	circRNA_015460	12:49599108|49802316
circRNA_009535	5:38350856|38356438	circRNA_015471	13:1164933|1169589	circRNA_008775	13:81350434|81375200	circRNA_011886	6:82704332|82907607
circRNA_000932	15:71703524|71709108	circRNA_002512	1:62688792|62693450	circRNA_012273	1:176374364|176399133	circRNA_008059	8:134355181|134558516
circRNA_011890	6:85446257|85451841	circRNA_004208	3:125868498|125873156	circRNA_018977	3:61083712|61108481	circRNA_004570	6:73884323|74087879
circRNA_013811	13:28831208|28836794	circRNA_004344	4:117913001|117917659	circRNA_016755	12:20097144|20121928	circRNA_010584	2:104942961|105146567
circRNA_013646	9:50851165|50856753	circRNA_005614	12:18570378|18575036	circRNA_010371	13:55530278|55555064	circRNA_019941	1:252251743|252455387
circRNA_017687	15:116470717|116476309	circRNA_016706	1:303952596|303957254	circRNA_011865	6:16284471|16309258	circRNA_002857	10:71842679|72046341
circRNA_008609	11:52164913|52170506	circRNA_002837	10:55803599|55808258	circRNA_014330	17:43054927|43079731	circRNA_012930	2:66266081|66469763
circRNA_014659	14:136012794|136018388	circRNA_005021	8:141415791|141420453	circRNA_020725	15:18299032|18323838	circRNA_009255	2:66266081|66469766
circRNA_014153	10:31872484|31878079	circRNA_009747	7:47364244|47368909	circRNA_013032	3:92436939|92461755	circRNA_017620	14:7872804|8076556
circRNA_012801	16:36573083|36578679	circRNA_001991	7:69874055|69878724	circRNA_001917	6:146123453|146148271	circRNA_014232	14:7879060|8082837
circRNA_017856	5:23510712|23516308	circRNA_004597	6:85446257|85450927	circRNA_008826	13:144384384|144409204	circRNA_010610	3:25027237|25231144
circRNA_001141	18:5280797|5286395	circRNA_005597	11:26031169|26035840	circRNA_011172	12:27543194|27568015	circRNA_002262	9:89509653|89713831
circRNA_013023	3:74760760|74766358	circRNA_005113	9:44920845|44925517	circRNA_013822	13:117581097|117605927	circRNA_012154	X:60818920|61023192
circRNA_011282	13:190957762|190963361	circRNA_007000	13:72415384|72420056	circRNA_002239	9:61784105|61808937	circRNA_020849	18:59130851|59335593
circRNA_020129	3:19413230|19418829	circRNA_013241	6:56970252|56974925	circRNA_002842	10:58856976|58881810	circRNA_004147	3:99088814|99293574
circRNA_007647	4:44324577|44330177	circRNA_016067	4:51531332|51536005	circRNA_000475	12:31812043|31836878	circRNA_007539	3:25026227|25231144
circRNA_019632	2:85385765|85391366	circRNA_019771	6:136124951|136129624	circRNA_011720	3:104182825|104207660	circRNA_018172	14:136683518|136888438
circRNA_000285	10:16225280|16230884	circRNA_002772	10:16565804|16570479	circRNA_012737	15:86614285|86639123	circRNA_017347	9:113115091|113320145
circRNA_007119	14:43482274|43487878	circRNA_012722	15:62337487|62342162	circRNA_013687	13:166273794|166298634	circRNA_000037	1:37134829|37340400
circRNA_002680	1:207016864|207022469	circRNA_014621	10:16947860|16952536	circRNA_007688	4:117084933|117109792	circRNA_020216	6:52454181|52659809
circRNA_007386	18:16116196|16121801	circRNA_010252	1:282191305|282195982	circRNA_009400	3:110292158|110317020	circRNA_007662	4:72410011|72615981
circRNA_011517	17:41836737|41842344	circRNA_013794	10:37491247|37495924	circRNA_003816	18:10792104|10816967	circRNA_019149	7:16831904|17037985
circRNA_000338	10:48227259|48232868	circRNA_013333	7:73057336|73062014	circRNA_009296	2:124331328|124356191	circRNA_014470	6:54760479|54967024
circRNA_005463	1:146435563|146441172	circRNA_018012	9:144919270|144923948	circRNA_007218	15:28526428|28551296	circRNA_007549	3:43020441|43227527
circRNA_009533	5:38330531|38336140	circRNA_005435	1:101722145|101726824	circRNA_017385	X:17526788|17551656	circRNA_006708	1:160189429|160396819
circRNA_014658	14:97914495|97920104	circRNA_019332	JH118993.1:13905|18585	circRNA_009704	6:145384185|145409067	circRNA_002572	1:127822367|128030115
circRNA_007293	15:145119846|145125460	circRNA_006400	8:43039144|43043827	circRNA_009493	4:116997042|117021925	circRNA_010195	1:96497075|96704876
circRNA_000278	10:10312706|10318322	circRNA_009365	3:61062817|61067500	circRNA_017967	8:118871562|118896447	circRNA_011676	3:43019324|43227527
circRNA_020620	13:126737883|126743499	circRNA_006301	6:125939729|125944413	circRNA_003269	13:210819581|210844473	circRNA_000500	12:49593957|49802316
circRNA_001373	2:154312784|154318402	circRNA_013213	5:109487613|109492299	circRNA_008269	X:109068407|109093306	circRNA_002457	1:4427583|4636526
circRNA_001561	4:39632783|39638405	circRNA_001459	3:91185717|91190405	circRNA_018509	1:254957497|254982407	circRNA_011415	15:85315348|85524609
circRNA_011773	4:73582505|73588127	circRNA_003318	14:30062323|30067011	circRNA_014011	15:131248910|131273823	circRNA_017454	1:127822367|128031634
circRNA_021312	GL893852.2:61918|67540	circRNA_016842	14:34671930|34676618	circRNA_000748	14:21472380|21497303	circRNA_005462	1:146253719|146463424
circRNA_011525	17:46535955|46541578	circRNA_010056	GL893735.2:22131|26822	circRNA_005194	9:139626975|139651900	circRNA_018805	15:124483398|124693334
circRNA_005414	1:28658853|28664480	circRNA_012254	1:132962462|132967155	circRNA_018318	6:127467983|127492913	circRNA_015723	15:116425424|116636020
circRNA_006514	GL892277.2:90379|96006	circRNA_012662	14:71920692|71925385	circRNA_018833	16:47823935|47848867	circRNA_000150	1:146252562|146463424
circRNA_016109	4:129636623|129642250	circRNA_000126	1:134770024|134774718	circRNA_005144	9:101227171|101252107	circRNA_002777	10:17976426|18187909
circRNA_006461	9:29699977|29705606	circRNA_005958	18:52291604|52296298	circRNA_015812	18:9728802|9753741	circRNA_007542	3:29618701|29830248
circRNA_008273	X:121770822|121776451	circRNA_014832	3:128268804|128273499	circRNA_005727	13:144096992|144121936	circRNA_015535	13:98848273|99060604
circRNA_008304	1:30397448|30403077	circRNA_016343	8:10593044|10597739	circRNA_009993	9:96050798|96075749	circRNA_016136	5:32847751|33060293
circRNA_000874	14:145858903|145864533	circRNA_006192	5:9128682|9133379	circRNA_018734	14:61608442|61633400	circRNA_010712	5:70959926|71172480
circRNA_012335	10:92171|97801	circRNA_015389	11:26247793|26252490	circRNA_002236	9:55395986|55420953	circRNA_004416	5:22894103|23106699
circRNA_012463	12:13088210|13093840	circRNA_004912	8:40572430|40577131	circRNA_002875	11:5263855|5288837	circRNA_002197	9:13522653|13735468
circRNA_012715	15:51628512|51634142	circRNA_013037	3:99317794|99322497	circRNA_011590	2:50783412|50808396	circRNA_021097	6:32171146|32383981
circRNA_007883	6:148098232|148103865	circRNA_014054	6:89828173|89832876	circRNA_005313	X:12384750|12409755	circRNA_001304	2:90636261|90849505
circRNA_015971	3:55061561|55067194	circRNA_012158	X:70832475|70837179	circRNA_008062	8:141381057|141406067	circRNA_006181	4:129268028|129481379
circRNA_018803	15:116470717|116476350	circRNA_015681	15:18325856|18330560	circRNA_014893	9:144068891|144093904	circRNA_015575	13:202561687|202775160
circRNA_013665	10:47064149|47069783	circRNA_011394	15:34890619|34895324	circRNA_019255	9:36642256|36667272	circRNA_008255	X:70426183|70639982
circRNA_002433	X:105403828|105409464	circRNA_006248	6:10030267|10034973	circRNA_008317	1:56058918|56083940	circRNA_019906	1:37126558|37340400

circRNA_004515	6:2770529|2776167	circRNA_019631	2:84499015|84503722	circRNA_015938	2:145702685|145727710	circRNA_009349	3:43019324|43233296
circRNA_006549	MT:2476|8114	circRNA_012804	16:37509996|37514704	circRNA_006580	X:105384438|105409464	circRNA_007082	13:202560796|202775160
circRNA_008271	X:116346682|116352321	circRNA_003376	14:77567920|77572629	circRNA_006616	1:23208792|23233819	circRNA_007853	6:101724150|101938585
circRNA_009745	7:43370412|43376051	circRNA_014553	9:51074787|51079497	circRNA_007890	6:150870199|150895226	circRNA_003181	13:98845780|99060604
circRNA_002348	GL894341.1:34020|39660	circRNA_005675	13:50643382|50648094	circRNA_016144	5:47366057|47391091	circRNA_014644	13:130157827|130372748
circRNA_012783	15:143217390|143223032	circRNA_017095	4:19745849|19750562	circRNA_000119	1:134611516|134636559	circRNA_017549	11:52209155|52424136
circRNA_014118	1:142576039|142581681	circRNA_019739	5:82929939|82934653	circRNA_008827	13:144391256|144416300	circRNA_020599	13:77074123|77289727
circRNA_008998	15:51396170|51401102	circRNA_020856	2:4159831|4164547	circRNA_010297	10:66158459|66183519	circRNA_014110	1:120698326|120914329
circRNA_010767	6:136176938|136181870	circRNA_006266	6:63739623|63744340	circRNA_002935	11:72371861|72396926	circRNA_019295	9:134113705|134329949
circRNA_005931	17:52892065|52897001	circRNA_021137	6:145377688|145382405	circRNA_005359	X:105384438|105409507	circRNA_013772	1:134889882|135106264
circRNA_019374	1:126092089|126097028	circRNA_008666	12:37380479|37385204	circRNA_021108	6:81553639|81578716	circRNA_004962	8:83064146|83280539
circRNA_020317	9:44480031|44484973	circRNA_012479	12:25020435|25025161	circRNA_004875	8:3206790|3231881	circRNA_007693	4:126965792|127183082
circRNA_003176	13:91490811|91495754	circRNA_020490	1:314052726|314057452	circRNA_005948	18:19271818|19296928	circRNA_011558	18:59118039|59335593
circRNA_003390	14:78243180|78248123	circRNA_016701	1:286228472|286233200	circRNA_019389	1:181357855|181382973	circRNA_018289	5:80177918|80396308
circRNA_006518	GL893095.2:53303|58246	circRNA_001979	7:62801435|62806164	circRNA_001637	4:117897403|117922524	circRNA_004718	7:13095681|13314490
circRNA_006548	MT:2343|7286	circRNA_009750	7:53606391|53611120	circRNA_002637	1:183810304|183835431	circRNA_013844	14:136612215|136831101
circRNA_020964	3:72515026|72519969	circRNA_017145	5:72090687|72095416	circRNA_006914	12:15250677|15275808	circRNA_000038	1:37134829|37353844
circRNA_007109	14:33386567|33391511	circRNA_005695	13:90161833|90166564	circRNA_001075	16:71578933|71604069	circRNA_000512	12:56136238|56355578
circRNA_011339	14:69264712|69269656	circRNA_013553	JH118963.1:42907|47640	circRNA_007266	15:94342050|94367189	circRNA_002196	9:13516002|13735468
circRNA_016447	9:38455021|38459965	circRNA_009059	15:131253281|131258016	circRNA_007168	14:113982858|114008000	circRNA_014596	X:31558043|31777642
circRNA_006604	1:16385819|16390764	circRNA_009265	2:85485451|85490186	circRNA_008482	1:280564252|280589395	circRNA_012996	3:25027237|25247061
circRNA_014889	9:115756518|115761463	circRNA_003519	15:53217508|53222244	circRNA_011189	12:62428106|62453250	circRNA_017617	13:202561687|202781523
circRNA_010967	1:56058918|56063865	circRNA_009467	4:71305686|71310422	circRNA_016289	7:48909170|48934323	circRNA_003253	13:202560796|202781523
circRNA_002050	8:1266332|1271280	circRNA_012034	9:71135|75871	circRNA_020172	4:42102055|42127212	circRNA_010609	3:25026227|25247061
circRNA_019412	1:282139680|282144628	circRNA_005506	1:233128640|233133377	circRNA_004205	3:122078615|122103795	circRNA_006491	9:102309677|102530830
circRNA_003667	16:23393877|23398827	circRNA_020248	7:7836456|7841194	circRNA_009822	8:6793647|6818840	circRNA_002858	10:71842679|72064853
circRNA_017927	7:37133067|37138017	circRNA_008590	11:16160343|16165084	circRNA_019124	6:125316556|125341751	circRNA_000212	1:231411149|231633622
circRNA_006919	12:20322486|20327437	circRNA_002156	8:141404602|141409344	circRNA_017425	1:31387491|31412699	circRNA_006855	10:71845711|72068597
circRNA_007054	13:132982287|132987238	circRNA_005167	9:127853331|127858073	circRNA_012588	13:148316595|148341820	circRNA_012197	1:31299808|31522880
circRNA_018083	1:127648623|127653574	circRNA_013091	4:42001869|42006613	circRNA_001885	6:104029971|104055208	circRNA_018396	X:43139794|43362956
circRNA_018880	18:41980502|41985453	circRNA_009200	18:46987008|46991753	circRNA_014584	GL894654.1:30323|55560	circRNA_002778	10:17976426|18199788
circRNA_015651	14:94932160|94937112	circRNA_020117	2:76207608|76212354	circRNA_011564	2:4157505|4182749	circRNA_000361	10:71835595|72059048
circRNA_000887	15:19321481|19326434	circRNA_012992	3:11449859|11454606	circRNA_013460	9:51514094|51539338	circRNA_012446	11:71092929|71316450
circRNA_008438	1:210150968|210155927	circRNA_019189	7:103941332|103946081	circRNA_002167	8:146890664|146915909	circRNA_020899	2:90636261|90860554
circRNA_007495	2:97529618|97534578	circRNA_015186	1:87763598|87768349	circRNA_002738	1:270451103|270476354	circRNA_017849	4:129256921|129481379
circRNA_009022	15:73568823|73573786	circRNA_008053	8:123888989|123893742	circRNA_015534	13:97282134|97307385	circRNA_021244	9:10497641|10722417
circRNA_021166	7:73061859|73066822	circRNA_015237	1:201971402|201976155	circRNA_007074	13:159951029|159976282	circRNA_006479	9:62120957|62346821
circRNA_003174	13:91221808|91226772	circRNA_001057	16:37377919|37382673	circRNA_002410	X:17724969|17750223	circRNA_001770	5:106346589|106572931
circRNA_008721	13:26009326|26014292	circRNA_018835	16:50732900|50737656	circRNA_019595	17:24573209|24598468	circRNA_020165	4:30384145|30610583
circRNA_019483	13:61951062|61956028	circRNA_006177	4:113223848|113228605	circRNA_021018	4:73582505|73607764	circRNA_006115	3:123290596|123517096
circRNA_005938	18:10153905|10158873	circRNA_001085	17:13778285|13783043	circRNA_010805	7:91925254|91950519	circRNA_017744	18:59324466|59551671
circRNA_010372	13:59859476|59864444	circRNA_004640	6:103715572|103720331	circRNA_003276	13:211131404|211156675	circRNA_005418	1:37113063|37340369
circRNA_014575	GL892594.2:55914|60882	circRNA_019204	8:32884268|32889029	circRNA_003857	18:44782112|44807387	circRNA_005524	1:292352093|292579585
circRNA_014305	15:133614874|133619843	circRNA_004085	3:45711491|45716254	circRNA_007970	7:124972162|124997437	circRNA_002180	9:10497641|10725453
circRNA_014991	14:13658285|13663254	circRNA_000025	1:18698062|18702826	circRNA_003967	2:89767470|89792748	circRNA_017880	6:46558196|46786093
circRNA_019058	5:7471445|7476414	circRNA_016391	8:108424880|108429644	circRNA_009169	18:5609233|5634518	circRNA_017451	1:127113152|127342796
circRNA_006628	1:45701230|45706203	circRNA_000623	13:97302620|97307385	circRNA_021277	9:88634775|88660063	circRNA_006070	3:44877899|45107781
circRNA_009424	3:135192228|135197201	circRNA_016341	8:10503213|10507978	circRNA_002832	10:51236326|51261622	circRNA_002768	10:2811299|3041254
circRNA_016313	7:97321612|97326586	circRNA_005162	9:120394455|120399224	circRNA_016456	9:43030233|43055529	circRNA_008284	X:138419390|138651247
circRNA_020284	8:39573095|39578069	circRNA_007932	7:73436271|73441040	circRNA_020210	6:16284471|16309767	circRNA_005067	9:18898160|19130096
circRNA_006639	1:61006639|61011614	circRNA_005321	X:17560477|17565248	circRNA_002568	1:126147153|126172455	circRNA_009353	3:44877899|45110472
circRNA_020167	4:38935694|38940670	circRNA_008222	GL896320.1:15488|20259	circRNA_009802	7:105383271|105408581	circRNA_004507	5:106346589|106579236

circRNA_020956	3:51879967|51884944	circRNA_011881	6:73940101|73944873	circRNA_004883	8:15232138|15257460	circRNA_005369	X:117046354|117279315
circRNA_016271	7:15449794|15454773	circRNA_014217	13:132977618|132982390	circRNA_015721	15:115465676|115491006	circRNA_018646	13:87373670|87606658
circRNA_017071	3:75778196|75783176	circRNA_009903	8:141381057|141385830	circRNA_000504	12:50456560|50481892	circRNA_015862	2:8281327|8514578
circRNA_006818	10:27621291|27626272	circRNA_003122	13:50653633|50658407	circRNA_000815	14:81955876|81981214	circRNA_010592	2:130566550|130799830
circRNA_008283	X:130750807|130755789	circRNA_011680	3:46226093|46230867	circRNA_000854	14:122832506|122857850	circRNA_019979	12:35080276|35313861
circRNA_000346	10:54218225|54223208	circRNA_014230	14:936878|941652	circRNA_020364	1:10921743|10947088	circRNA_010615	3:44873984|45107781
circRNA_014725	7:125461|130444	circRNA_015871	2:28881755|28886529	circRNA_019406	1:256829848|256855195	circRNA_008564	10:65034439|65268916
circRNA_020641	13:156865631|156870615	circRNA_009448	4:41737282|41742058	circRNA_003553	15:85315348|85340697	circRNA_019527	14:55634893|55869595
circRNA_014353	2:27609998|27614983	circRNA_007824	6:64542087|64546864	circRNA_014897	X:46490203|46515564	circRNA_010267	10:17976426|18211136
circRNA_015771	16:51802418|51807404	circRNA_006480	9:62830495|62835273	circRNA_001579	4:55272747|55298110	circRNA_010683	4:126947969|127183082
circRNA_011892	6:88379721|88384709	circRNA_011759	4:39632783|39637561	circRNA_016910	15:112941304|112966677	circRNA_008606	11:25838572|26073707
circRNA_002420	X:45333003|45337993	circRNA_013439	9:19264985|19269763	circRNA_002042	7:124952565|124977939	circRNA_010842	8:82828282|83064254
circRNA_006243	5:106605085|106610077	circRNA_020571	13:2389104|2393883	circRNA_009034	15:86889126|86914503	circRNA_021165	7:71385997|71622185
circRNA_010484	15:93751641|93756633	circRNA_013698	17:44398277|44403057	circRNA_007894	7:112298|137678	circRNA_003634	15:124483398|124720160
circRNA_007265	15:93751638|93756633	circRNA_001329	2:120022510|120027291	circRNA_015467	12:59671317|59696697	circRNA_019535	14:75745036|75982921
circRNA_017828	4:41938633|41943632	circRNA_004287	4:70407823|70412608	circRNA_018801	15:115391991|115417375	circRNA_011853	5:106346589|106585027
circRNA_001033	16:22463630|22468631	circRNA_015829	18:27408621|27413407	circRNA_004766	7:56418413|56443801	circRNA_005758	14:7872804|8111867
circRNA_007805	6:38144053|38149056	circRNA_020386	1:62791099|62795885	circRNA_005102	9:40532481|40557886	circRNA_018401	X:144028110|144267481
circRNA_015602	14:36436272|36441275	circRNA_004858	7:125239034|125243823	circRNA_015815	18:10902368|10927784	circRNA_005139	9:82058623|82298140
circRNA_008417	1:188117293|188122298	circRNA_016774	13:2814140|2818929	circRNA_018495	1:216271971|216297389	circRNA_005826	15:7838580|8078171
circRNA_015089	17:58803349|58808354	circRNA_006924	12:23727465|23732256	circRNA_003055	12:62513102|62538538	circRNA_000273	1:311422004|311661929
circRNA_017195	6:113405301|113410311	circRNA_002893	11:19661652|19666444	circRNA_016261	6:147623208|147648651	circRNA_001417	3:49218842|49459764
circRNA_014037	1:145412858|145417869	circRNA_006729	1:191691090|191695882	circRNA_010677	4:90616840|90642287	circRNA_007851	6:101697609|101938585
circRNA_007548	3:42877609|42882621	circRNA_015462	12:52746087|52750879	circRNA_006129	4:32690092|32715543	circRNA_000730	14:7879060|8120115
circRNA_008154	9:115681075|115686087	circRNA_016039	3:134075578|134080373	circRNA_014261	14:114543856|114569311	circRNA_005877	15:137710958|137952597
circRNA_002791	10:29339600|29344613	circRNA_007365	17:51654188|51658984	circRNA_013992	1:293274600|293300073	circRNA_010168	1:31299808|31541885
circRNA_008024	8:76856232|76861250	circRNA_009479	4:91017657|91022453	circRNA_009437	4:27585033|27610508	circRNA_019490	13:122249994|122492244
circRNA_003614	15:116468167|116473189	circRNA_011424	15:89632723|89637520	circRNA_011410	15:73548305|73573786	circRNA_010391	13:121696322|121938801
circRNA_014009	15:62040539|62045562	circRNA_015333	10:31870846|31875643	circRNA_014610	1:75096804|75122290	circRNA_006102	3:109395414|109638430
circRNA_006009	2:87542278|87547302	circRNA_004787	7:65188149|65192947	circRNA_019327	GL896504.1:29972|55460	circRNA_006624	1:31299808|31542935
circRNA_016511	GL892680.2:158928|163954	circRNA_008955	14:120786139|120790937	circRNA_018421	1:75096804|75122293	circRNA_005539	10:17976426|18219757
circRNA_007013	13:84710600|84715627	circRNA_019110	6:86661788|86666587	circRNA_019880	JH118602.1:26199|51695	circRNA_001688	5:13724605|13968507
circRNA_009525	5:17117262|17122289	circRNA_008633	12:11461813|11466613	circRNA_009956	9:44920845|44946346	circRNA_021082	5:106346589|106590817
circRNA_012756	15:112941304|112946331	circRNA_002896	11:19744404|19749205	circRNA_017259	8:31861798|31887300	circRNA_012820	16:68067221|68311833
circRNA_021377	X:106079635|106084662	circRNA_005100	9:40527988|40532789	circRNA_014352	2:18674710|18700214	circRNA_011226	13:58571575|58816633
circRNA_003871	18:59916697|59921726	circRNA_018304	6:28766216|28771018	circRNA_005775	14:33448366|33473875	circRNA_011381	14:136612215|136857384
circRNA_005783	14:44212456|44217487	circRNA_003873	2:1469366|1474171	circRNA_001998	7:73060730|73086240	circRNA_000327	10:45755013|46000278
circRNA_003859	18:44787541|44792573	circRNA_008773	13:75857204|75862009	circRNA_008960	14:132237197|132262710	circRNA_001215	2:4334166|4579464
circRNA_007975	7:130871892|130876924	circRNA_012857	18:837419|842224	circRNA_000381	11:6761843|6787357	circRNA_002275	9:102309677|102554986
circRNA_009912	9:22715|27748	circRNA_002099	8:75774299|75779107	circRNA_021314	GL894290.1:26833|52351	circRNA_001243	2:47573699|47819708
circRNA_010399	13:146716493|146721526	circRNA_009567	5:72062877|72067689	circRNA_002228	9:44920845|44946366	circRNA_013131	4:126965792|127212472
circRNA_015325	10:11923439|11928472	circRNA_014432	4:104473253|104478066	circRNA_014080	1:205472022|205497543	circRNA_010112	X:30303924|30550767
circRNA_018806	15:124719044|124724077	circRNA_009812	7:128841625|128846439	circRNA_003834	18:18690198|18715721	circRNA_001316	2:103773335|104020777
circRNA_011714	3:101033374|101038408	circRNA_014407	3:122011566|122016380	circRNA_005353	X:78856057|78881582	circRNA_006492	9:102345868|102594047
circRNA_020062	15:86614285|86619319	circRNA_015674	14:140970111|140974925	circRNA_013004	3:45690728|45716254	circRNA_007622	4:7967422|8215800
circRNA_019370	1:101756560|101761598	circRNA_003288	14:6706029|6710845	circRNA_001687	5:13361763|13387293	circRNA_010224	1:199178179|199426835
circRNA_004210	3:130387896|130392936	circRNA_019191	7:118366454|118371270	circRNA_001932	6:153309526|153335056	circRNA_001432	3:61786857|62035671
circRNA_019758	6:64478265|64483305	circRNA_009797	7:97791041|97795858	circRNA_005291	JH118724.1:73289|98820	circRNA_017155	5:106346589|106595873
circRNA_018189	15:133558194|133563235	circRNA_014736	9:255216|260033	circRNA_019350	X:106962557|106988101	circRNA_019543	14:106458147|106707652
circRNA_011944	7:57942275|57947318	circRNA_000460	12:18731949|18736767	circRNA_002949	12:1724782|1750329	circRNA_019613	18:59169585|59419284
circRNA_012408	11:508651|513694	circRNA_015666	14:131874195|131879014	circRNA_011854	5:109516321|109541874	circRNA_012010	8:83030549|83280539
circRNA_002716	1:251721539|251726583	circRNA_006391	8:31876392|31881213	circRNA_017842	4:103229893|103255448	circRNA_017637	14:75664798|75915191

circRNA_016951	16:86029403|86034448	circRNA_015977	3:60073410|60078231	circRNA_000296	10:21700951|21726508	circRNA_005868	15:116434114|116684653
circRNA_019725	5:42899527|42904572	circRNA_008461	1:245015947|245020769	circRNA_016684	1:233128640|233154197	circRNA_000416	11:52492560|52743301
circRNA_002466	1:16327908|16332954	circRNA_010613	3:42964694|42969522	circRNA_008541	10:49667206|49692765	circRNA_013762	1:1098250|1349139
circRNA_010055	GL893689.1:47831|52877	circRNA_018846	17:29497087|29501915	circRNA_009460	4:55324656|55350215	circRNA_020163	4:30352899|30604164
circRNA_008360	1:125156966|125162014	circRNA_011681	3:48103861|48108692	circRNA_011794	4:134737116|134762676	circRNA_001011	15:124467909|124720160
circRNA_009765	7:69530612|69535660	circRNA_016385	8:89359060|89363891	circRNA_004336	4:107957216|107982777	circRNA_008294	1:16870879|17123292
circRNA_015744	16:23467262|23472310	circRNA_013258	6:88792955|88797787	circRNA_006493	9:102587388|102612959	circRNA_000732	14:8079522|8332107
circRNA_014447	5:45551752|45556801	circRNA_017166	6:39985890|39990722	circRNA_014556	9:79015624|79041213	circRNA_021167	7:76473147|76726160
circRNA_016551	GL896381.1:8217|13266	circRNA_014397	3:75694449|75699282	circRNA_011903	6:119987798|120013390	circRNA_009779	7:76473147|76726179
circRNA_006324	7:21551602|21556652	circRNA_001782	6:16298300|16303134	circRNA_020578	13:29719703|29745296	circRNA_005523	1:292340588|292594140
circRNA_005163	9:125506887|125511940	circRNA_016773	13:2389048|2393883	circRNA_008604	11:25838572|25864166	circRNA_014944	7:100363470|100617788
circRNA_002576	1:132216531|132221585	circRNA_005888	16:23993547|23998383	circRNA_006776	1:275780524|275806120	circRNA_010582	2:92063632|92318020
circRNA_006728	1:188418093|188423148	circRNA_001814	6:66397830|66402669	circRNA_010242	1:242160698|242186297	circRNA_013977	X:54411969|54666437
circRNA_003618	15:117842519|117847576	circRNA_019807	7:103850529|103855368	circRNA_003886	2:16134534|16160140	circRNA_009960	9:44922267|45177387
circRNA_018138	13:145249409|145254468	circRNA_002341	GL894053.2:50106|54948	circRNA_008997	15:50827984|50853593	circRNA_018286	5:50734973|50990139
circRNA_018252	3:25761999|25767058	circRNA_019910	1:62517228|62522071	circRNA_009906	8:143249769|143275381	circRNA_010175	1:41253114|41508508
circRNA_003934	2:80548536|80553596	circRNA_000950	15:85065639|85070483	circRNA_012834	17:22546656|22572272	circRNA_013020	3:67152058|67408623
circRNA_018039	GL896553.1:668|5728	circRNA_003836	18:25506902|25511747	circRNA_006469	9:40043108|40068725	circRNA_009546	5:52910885|53167931
circRNA_013656	1:132216524|132221585	circRNA_014751	GL894982.1:12114|16959	circRNA_002760	1:300126112|300151730	circRNA_006811	10:17000906|17259104
circRNA_006738	1:202793118|202798180	circRNA_015141	1:16139074|16143919	circRNA_003270	13:210819581|210845206	circRNA_001587	4:61688167|61946991
circRNA_016938	16:37513854|37518916	circRNA_005533	1:306219172|306224018	circRNA_002859	10:75648814|75674446	circRNA_010577	2:88879257|89138199
circRNA_000923	15:61777761|61782824	circRNA_001683	5:4603986|4608833	circRNA_000041	1:39602177|39627811	circRNA_014619	1:294250139|294509756
circRNA_019296	9:136028063|136033126	circRNA_014908	1:186645420|186650267	circRNA_009983	9:78881650|78907286	circRNA_020977	3:103625813|103885769
circRNA_009861	8:78097469|78102533	circRNA_008439	1:215873363|215878211	circRNA_009789	7:91938950|91964590	circRNA_001207	2:2086144|2346816
circRNA_006441	9:32780|37845	circRNA_008509	10:16256296|16261144	circRNA_008140	9:96027814|96053461	circRNA_014863	7:76463695|76726179
circRNA_009111	16:51834324|51839390	circRNA_021043	5:5263234|5268083	circRNA_000413	11:52209155|52234803	circRNA_006927	12:24040529|24303120
circRNA_020282	8:39536968|39542034	circRNA_021089	6:11853451|11858302	circRNA_014098	1:32143461|32169111	circRNA_001382	3:9225531|9489014
circRNA_010039	GL892407.1:97049|102116	circRNA_018729	14:59602182|59607034	circRNA_007355	17:42175058|42200711	circRNA_014812	18:59071515|59335593
circRNA_001385	3:10315918|10320986	circRNA_012011	8:86001857|86006710	circRNA_019174	7:88992382|89018039	circRNA_005342	X:70438343|70705622
circRNA_001751	5:79767220|79772288	circRNA_020507	10:45979341|45984194	circRNA_017423	1:21206251|21231923	circRNA_001098	17:31364763|31633002
circRNA_003576	15:87535488|87540556	circRNA_019703	4:102437791|102442646	circRNA_017703	16:27064859|27090533	circRNA_017230	7:76473147|76743403
circRNA_002747	1:283781181|283786252	circRNA_011614	2:89782086|89786942	circRNA_007170	14:114868474|114894153	circRNA_012366	10:45728965|46000278
circRNA_007249	15:73552151|73557223	circRNA_017345	9:101641679|101646535	circRNA_015558	13:149631857|149657541	circRNA_001026	15:147470007|147741406
circRNA_013855	15:133579638|133584711	circRNA_015580	13:209980722|209985579	circRNA_004546	6:32419351|32445038	circRNA_013874	18:59169585|59442278
circRNA_016545	GL895834.1:6816|11889	circRNA_008335	1:86322010|86326869	circRNA_004217	3:135341139|135366829	circRNA_009975	9:62074009|62346821
circRNA_017930	7:57275722|57280795	circRNA_003615	15:117366653|117371514	circRNA_015175	1:72029648|72055341	circRNA_016063	4:42651856|42925266
circRNA_009893	8:128735200|128740274	circRNA_018798	15:106592271|106597133	circRNA_018653	13:100697258|100722959	circRNA_011329	14:55361383|55634995
circRNA_019154	7:33504832|33509906	circRNA_018856	17:51680010|51684873	circRNA_002982	12:23250407|23276109	circRNA_008514	10:17976426|18250156
circRNA_000667	13:143414634|143419711	circRNA_019015	4:16810092|16814955	circRNA_009288	2:118428431|118454133	circRNA_001097	17:31192683|31467207
circRNA_016485	9:96037782|96042859	circRNA_014003	13:210787792|210792658	circRNA_018836	16:68473826|68499529	circRNA_013691	14:136612215|136888438
circRNA_020182	4:107464006|107469084	circRNA_006426	8:109055311|109060178	circRNA_016205	6:39446578|39472285	circRNA_011121	10:58751706|59028119
circRNA_010740	6:46781014|46786093	circRNA_017068	3:47808482|47813349	circRNA_006699	1:145758225|145783940	circRNA_003609	15:115686877|115963395
circRNA_015295	1:264888802|264893881	circRNA_003511	15:51607781|51612650	circRNA_010827	8:42317790|42343516	circRNA_000992	15:115686849|115963395
circRNA_019111	6:88476536|88481618	circRNA_017652	14:131868321|131873191	circRNA_015644	14:81910115|81935846	circRNA_010435	14:55592489|55869595
circRNA_018715	14:33398976|33404059	circRNA_017727	17:55235758|55240628	circRNA_019310	GL893726.1:21500|47232	circRNA_021152	7:21278745|21556652
circRNA_012328	1:299796549|299801634	circRNA_005477	1:199966226|199971098	circRNA_015216	1:140671383|140697116	circRNA_005965	2:4301101|4579464
circRNA_007838	6:85026828|85031914	circRNA_013950	8:57807091|57811963	circRNA_011187	12:53698948|53724685	circRNA_010551	18:52077580|52356954
circRNA_004654	6:125666114|125671201	circRNA_014042	13:90784807|90789682	circRNA_007918	7:62801435|62827182	circRNA_002055	8:4036951|4316462
circRNA_015239	1:202645930|202651017	circRNA_007102	14:21827434|21832311	circRNA_013614	7:10297937|10323693	circRNA_010409	13:168196194|168476335
circRNA_009471	4:72849038|72854126	circRNA_008581	11:11212642|11217519	circRNA_016070	4:62973617|62999378	circRNA_011746	4:27328016|27608273
circRNA_005032	8:144760599|144765688	circRNA_012672	14:98549486|98554363	circRNA_008951	14:117074459|117100223	circRNA_000692	13:168193917|168476335
circRNA_007716	5:4783980|4789070	circRNA_016022	3:114044395|114049273	circRNA_001884	6:103828536|103854303	circRNA_011747	4:27328016|27610508

circRNA_014986	13:102343369|102348459	circRNA_018706	14:30933428|30938306	circRNA_019922	1:130193941|130219709	circRNA_018265	4:61661225|61946991
circRNA_000353	10:58907402|58912493	circRNA_018202	17:65689980|65694859	circRNA_011873	6:50954116|50979886	circRNA_013397	8:83143287|83429055
circRNA_020637	13:148492058|148497149	circRNA_017647	14:115739339|115744220	circRNA_002454	X:143993560|144019339	circRNA_018886	18:43829041|44115219
circRNA_007504	2:108990313|108995405	circRNA_005396	X:143162695|143167577	circRNA_015359	10:58881715|58907497	circRNA_001546	4:15714361|16001218
circRNA_005702	13:103721266|103726359	circRNA_012833	17:19795612|19800494	circRNA_010073	GL894993.1:4503|30287	circRNA_013652	10:17976426|18263614
circRNA_008758	13:55549969|55555064	circRNA_020174	4:69938859|69943741	circRNA_004207	3:125854763|125880552	circRNA_017059	3:13957642|14246578
circRNA_009348	3:42678974|42684070	circRNA_015438	12:23738975|23743858	circRNA_021280	9:101932475|101958266	circRNA_011755	4:35980007|36269370
circRNA_010078	GL895293.1:90424|95521	circRNA_020607	13:90382800|90387683	circRNA_002428	X:70638360|70664153	circRNA_020715	14:136597966|136888438
circRNA_005227	GL893006.1:40845|45943	circRNA_006543	JH118544.1:1008|5893	circRNA_015814	18:10774476|10800270	circRNA_002222	9:43174650|43465932
circRNA_000077	1:101186411|101191510	circRNA_001948	7:21066190|21071076	circRNA_014122	1:161699776|161725572	circRNA_011857	6:8570907|8862389
circRNA_020145	3:72371962|72377063	circRNA_002295	9:138716542|138721428	circRNA_020368	1:18877993|18903790	circRNA_001213	2:4157505|4450312
circRNA_006277	6:82975244|82980346	circRNA_017372	GL894597.1:39761|44647	circRNA_019586	16:27667699|27693497	circRNA_007623	4:15707963|16001218
circRNA_008287	1:970393|975495	circRNA_008547	10:53350655|53355543	circRNA_010089	JH118901.1:410484|436283	circRNA_001214	2:4285881|4579464
circRNA_004927	8:57800743|57805849	circRNA_012560	13:86952848|86957736	circRNA_021383	X:122527958|122553757	circRNA_008293	1:16577027|16871069
circRNA_007404	18:44454732|44459840	circRNA_008102	9:38455021|38459910	circRNA_021287	9:124454928|124480730	circRNA_018167	14:115928981|116223189
circRNA_021352	X:12862202|12867311	circRNA_017209	6:148398330|148403219	circRNA_011151	11:76375686|76401491	circRNA_001816	6:69118812|69413634
circRNA_006332	7:43203479|43208589	circRNA_014661	15:61682878|61687771	circRNA_020743	15:80970228|80996035	circRNA_016402	8:123718584|124015123
circRNA_007866	6:127538813|127543923	circRNA_004367	4:129539588|129544482	circRNA_009591	5:92227690|92253499	circRNA_005952	18:41381384|41679822
circRNA_002177	9:8237835|8242946	circRNA_013372	8:30877162|30882057	circRNA_013383	8:46471841|46497652	circRNA_018208	18:35300414|35598886
circRNA_005561	10:54048970|54054083	circRNA_001041	16:24989252|24994149	circRNA_016509	GL892422.2:13578|39398	circRNA_004982	8:102838308|103138543
circRNA_012687	14:118366026|118371139	circRNA_002300	9:140014053|140018950	circRNA_020281	8:39516211|39542034	circRNA_001460	3:92198033|92502441
circRNA_015720	15:115363166|115368279	circRNA_018839	16:78195435|78200333	circRNA_008526	10:33542966|33568796	circRNA_009508	4:139280719|139586641
circRNA_019971	11:56136069|56141183	circRNA_020120	2:109792135|109797035	circRNA_011114	10:51413904|51439734	circRNA_016307	7:77398719|77705779
circRNA_013797	10:54218092|54223208	circRNA_000248	1:278917340|278922241	circRNA_013532	GL894666.1:112|25943	circRNA_018617	13:1571330|1879429
circRNA_020828	18:10795154|10800270	circRNA_010022	9:138880103|138885004	circRNA_010526	17:43227568|43253419	circRNA_005046	9:8593726|8902239
circRNA_021344	JH118701.1:16917|22033	circRNA_012633	14:33212834|33217736	circRNA_008165	9:128105060|128130921	circRNA_010887	9:77278140|77587010
circRNA_008880	14:23972014|23977134	circRNA_014600	X:85941401|85946304	circRNA_002192	9:13293149|13319023	circRNA_016650	1:141684631|141994380
circRNA_000281	10:13600749|13605870	circRNA_002980	12:20322532|20327437	circRNA_010714	5:79126662|79152538	circRNA_001182	18:35334600|35645277
circRNA_019506	13:199380000|199385121	circRNA_013107	4:81043876|81048781	circRNA_014729	8:20681698|20707578	circRNA_000129	1:141682675|141994380
circRNA_004656	6:125923718|125928840	circRNA_007006	13:77114677|77119583	circRNA_003368	14:66861565|66887449	circRNA_013633	2:118253397|118565484
circRNA_016329	7:124987073|124992196	circRNA_013045	3:107577150|107582057	circRNA_018041	JH118669.1:191011|216895	circRNA_000082	1:103946010|104258600
circRNA_019431	10:56267463|56272586	circRNA_013448	9:38455021|38459928	circRNA_005663	13:34707985|34733874	circRNA_002468	1:16577027|16889979
circRNA_020784	16:34053808|34058931	circRNA_003649	15:146530961|146535869	circRNA_004276	4:55364472|55390366	circRNA_003965	2:89270254|89583437
circRNA_009037	15:89681054|89686178	circRNA_020389	1:71050734|71055642	circRNA_007041	13:119243439|119269335	circRNA_005754	13:217844120|218158154
circRNA_003305	14:14668506|14673631	circRNA_010732	6:25836379|25841288	circRNA_010506	16:47791556|47817458	circRNA_015849	18:59169585|59484705
circRNA_001280	2:80561406|80566532	circRNA_006231	5:87303583|87308493	circRNA_009429	4:10825873|10851780	circRNA_009482	4:95979275|96295240
circRNA_005104	9:40658461|40663587	circRNA_011956	7:73081330|73086240	circRNA_004771	7:57942275|57968188	circRNA_008866	13:217835283|218153454
circRNA_004993	8:112720112|112725240	circRNA_009855	8:71347911|71352823	circRNA_004748	7:35362834|35388749	circRNA_016828	13:195196282|195514753
circRNA_009544	5:52435212|52440345	circRNA_012489	12:38659145|38664057	circRNA_009795	7:97300671|97326586	circRNA_015217	1:141682675|142001805
circRNA_008009	8:57798326|57803460	circRNA_010471	15:53787125|53792040	circRNA_016867	14:105105415|105131340	circRNA_007526	3:249816|570628
circRNA_012245	1:121462435|121467570	circRNA_009161	17:49550949|49555865	circRNA_014392	3:71982481|72008414	circRNA_001301	2:89270254|89591671
circRNA_015797	17:43623889|43629025	circRNA_000128	1:141607417|141612336	circRNA_014922	15:68860687|68886621	circRNA_017247	7:124953728|125277020
circRNA_007804	6:38087910|38093047	circRNA_010219	1:182855369|182860290	circRNA_013651	X:126961800|126987749	circRNA_004186	3:117737002|118060942
circRNA_004255	4:39637447|39642587	circRNA_008091	9:22394608|22399530	circRNA_004641	6:103715572|103741524	circRNA_005813	14:136612215|136938518
circRNA_006234	5:88153461|88158602	circRNA_009478	4:90624154|90629076	circRNA_009287	2:118253397|118279349	circRNA_018057	X:85891093|86217418
circRNA_008135	9:81539754|81544896	circRNA_013920	4:121581861|121586783	circRNA_013475	9:98305550|98331507	circRNA_004960	8:82954008|83280539
circRNA_008292	1:16375887|16381029	circRNA_017334	9:61794758|61799680	circRNA_012486	12:37694413|37720372	circRNA_000590	13:58244443|58571704
circRNA_009898	8:134701991|134707133	circRNA_017985	9:37674853|37679777	circRNA_020016	13:144152728|144178688	circRNA_003158	13:85132558|85460294
circRNA_003348	14:49392310|49397454	circRNA_006282	6:86771336|86776261	circRNA_003832	18:18675366|18701330	circRNA_020294	8:83143287|83472898
circRNA_004508	5:106590727|106595873	circRNA_011378	14:133487770|133492695	circRNA_007001	13:72802675|72828640	circRNA_018650	13:94465702|94796948
circRNA_018864	18:14552503|14557650	circRNA_012571	13:103721434|103726359	circRNA_005542	10:18123982|18149975	circRNA_004455	5:70515829|70847537
circRNA_011929	7:20645945|20651095	circRNA_020875	2:60043010|60047935	circRNA_005294	JH118921.1:9922|35922	circRNA_005528	1:297692640|298029257

<table>
<tr><td>circRNA_011706</td><td>3:90750338|90755491</td><td>circRNA_015738</td><td>15:133584611|133589537</td><td>circRNA_013849</td><td>15:90260328|90286332</td><td>circRNA_020718</td><td>14:136774429|137113957</td></tr>
<tr><td>circRNA_020304</td><td>9:19259967|19265120</td><td>circRNA_001358</td><td>2:142524037|142528964</td><td>circRNA_021211</td><td>8:82980802|83006817</td><td>circRNA_010809</td><td>7:108652047|108994426</td></tr>
<tr><td>circRNA_008726</td><td>13:28753936|28759090</td><td>circRNA_014443</td><td>5:33867909|33872838</td><td>circRNA_017458</td><td>1:134774498|134800523</td><td>circRNA_010346</td><td>12:48068342|48414455</td></tr>
<tr><td>circRNA_017762</td><td>2:81863649|81868804</td><td>circRNA_005028</td><td>8:144641851|144646781</td><td>circRNA_008444</td><td>1:218549684|218575722</td><td>circRNA_018765</td><td>14:136612215|136959046</td></tr>
<tr><td>circRNA_009846</td><td>8:44869888|44875044</td><td>circRNA_005249</td><td>GL894597.1:29121|34052</td><td>circRNA_001920</td><td>6:147026312|147052351</td><td>circRNA_020110</td><td>2:4103081|4450312</td></tr>
<tr><td>circRNA_000393</td><td>11:19591313|19596472</td><td>circRNA_010090</td><td>JH118902.1:28272|33203</td><td>circRNA_007742</td><td>5:43289784|43315838</td><td>circRNA_001092</td><td>17:19090796|19438735</td></tr>
<tr><td>circRNA_016710</td><td>10:236936|242095</td><td>circRNA_005966</td><td>2:8665085|8670273</td><td>circRNA_002744</td><td>1:280627976|280654037</td><td>circRNA_005525</td><td>1:293750060|294102001</td></tr>
<tr><td>circRNA_017993</td><td>9:57793732|57798891</td><td>circRNA_011587</td><td>2:47994183|47999371</td><td>circRNA_004905</td><td>8:39363973|39390036</td><td>circRNA_010345</td><td>12:48061363|48414455</td></tr>
<tr><td>circRNA_020388</td><td>1:64127213|64132372</td><td>circRNA_021145</td><td>7:2065136|2070324</td><td>circRNA_018471</td><td>1:181051819|181077882</td><td>circRNA_009950</td><td>9:42347056|42701853</td></tr>
<tr><td>circRNA_009014</td><td>15:66048762|66053922</td><td>circRNA_014825</td><td>3:24136275|24141464</td><td>circRNA_010122</td><td>X:58737103|58763171</td><td>circRNA_021216</td><td>8:90137348|90496491</td></tr>
<tr><td>circRNA_000112</td><td>1:132825590|132830751</td><td>circRNA_005039</td><td>9:364340|369530</td><td>circRNA_007226</td><td>15:44368123|44394195</td><td>circRNA_010140</td><td>X:78856057|79215288</td></tr>
<tr><td>circRNA_016618</td><td>1:9393570|9398731</td><td>circRNA_020700</td><td>14:97945819|97951009</td><td>circRNA_014496</td><td>7:69062211|69088285</td><td>circRNA_013453</td><td>9:42341153|42704335</td></tr>
<tr><td>circRNA_000204</td><td>1:227181487|227186649</td><td>circRNA_006026</td><td>2:107426104|107431295</td><td>circRNA_019713</td><td>5:200465|226541</td><td>circRNA_006245</td><td>6:826070|1190569</td></tr>
<tr><td>circRNA_020338</td><td>GL893509.1:54154|59316</td><td>circRNA_016237</td><td>6:92092464|92097655</td><td>circRNA_011337</td><td>14:65832702|65858779</td><td>circRNA_013786</td><td>1:224744341|225112654</td></tr>
<tr><td>circRNA_004915</td><td>8:41671944|41677107</td><td>circRNA_016891</td><td>15:36428546|36433738</td><td>circRNA_003753</td><td>17:19809062|19835142</td><td>circRNA_020842</td><td>18:43942640|44317450</td></tr>
<tr><td>circRNA_010298</td><td>10:76245922|76251085</td><td>circRNA_018241</td><td>2:139606241|139611434</td><td>circRNA_016730</td><td>10:70167741|70193825</td><td>circRNA_001912</td><td>6:139872924|140249378</td></tr>
<tr><td>circRNA_012238</td><td>1:116835411|116840574</td><td>circRNA_016080</td><td>4:73577490|73582684</td><td>circRNA_000108</td><td>1:127997793|128023881</td><td>circRNA_015262</td><td>1:224730841|225112654</td></tr>
<tr><td>circRNA_017352</td><td>9:137659557|137664720</td><td>circRNA_020771</td><td>15:157062557|157067751</td><td>circRNA_012517</td><td>13:33815459|33841548</td><td>circRNA_018890</td><td>18:59169585|59551671</td></tr>
<tr><td>circRNA_002588</td><td>1:134782801|134787969</td><td>circRNA_002164</td><td>8:145151051|145156246</td><td>circRNA_017190</td><td>6:103715572|103741661</td><td>circRNA_020443</td><td>1:199044683|199426813</td></tr>
<tr><td>circRNA_014434</td><td>4:118832318|118837487</td><td>circRNA_003729</td><td>16:55491215|55496410</td><td>circRNA_001993</td><td>7:72211421|72237514</td><td>circRNA_016054</td><td>4:30987156|31369823</td></tr>
<tr><td>circRNA_019484</td><td>13:66451168|66456337</td><td>circRNA_005405</td><td>1:17243547|17248742</td><td>circRNA_019888</td><td>X:73904558|73930652</td><td>circRNA_021212</td><td>8:84184070|84572018</td></tr>
<tr><td>circRNA_005467</td><td>1:176389138|176394308</td><td>circRNA_006318</td><td>7:94024|99219</td><td>circRNA_005128</td><td>9:72069020|72095126</td><td>circRNA_012888</td><td>18:59169585|59562118</td></tr>
<tr><td>circRNA_004943</td><td>8:71481618|71486789</td><td>circRNA_009539</td><td>5:43303529|43308724</td><td>circRNA_003051</td><td>12:59357665|59383777</td><td>circRNA_017391</td><td>X:54411969|54805230</td></tr>
<tr><td>circRNA_016245</td><td>6:99765860|99771031</td><td>circRNA_006205</td><td>5:50304124|50309320</td><td>circRNA_000755</td><td>14:30058998|30085114</td><td>circRNA_002529</td><td>1:86117059|86512992</td></tr>
<tr><td>circRNA_012656</td><td>14:65218520|65223693</td><td>circRNA_010258</td><td>10:20007|25203</td><td>circRNA_008819</td><td>13:140891503|140917627</td><td>circRNA_002527</td><td>1:86114517|86512992</td></tr>
<tr><td>circRNA_004879</td><td>8:10536893|10542067</td><td>circRNA_014811</td><td>18:5545554|5550750</td><td>circRNA_001050</td><td>16:36722483|36748618</td><td>circRNA_000071</td><td>1:86114517|86519619</td></tr>
<tr><td>circRNA_006634</td><td>1:59728051|59733226</td><td>circRNA_006373</td><td>7:122497132|122502329</td><td>circRNA_013339</td><td>7:91955977|91982115</td><td>circRNA_012070</td><td>9:77278140|77686459</td></tr>
<tr><td>circRNA_008592</td><td>11:19591297|19596472</td><td>circRNA_007923</td><td>7:65664182|65669379</td><td>circRNA_002178</td><td>9:8237835|8263976</td><td>circRNA_014349</td><td>18:58926636|59335593</td></tr>
<tr><td>circRNA_002157</td><td>8:141415791|141420967</td><td>circRNA_009307</td><td>2:140556463|140561662</td><td>circRNA_013872</td><td>18:41970023|41996168</td><td>circRNA_013806</td><td>12:60949301|61365239</td></tr>
<tr><td>circRNA_000491</td><td>12:44463879|44469057</td><td>circRNA_010433</td><td>14:44316366|44321566</td><td>circRNA_001499</td><td>3:114326822|114352969</td><td>circRNA_002425</td><td>X:66183818|66606485</td></tr>
<tr><td>circRNA_013290</td><td>6:148814622|148819803</td><td>circRNA_020061</td><td>15:85116262|85121462</td><td>circRNA_018445</td><td>1:133499719|133525870</td><td>circRNA_013081</td><td>4:35844936|36269370</td></tr>
<tr><td>circRNA_017598</td><td>13:81435164|81440345</td><td>circRNA_006227</td><td>5:86794414|86799615</td><td>circRNA_011321</td><td>14:34675990|34702143</td><td>circRNA_021357</td><td>X:31350228|31777642</td></tr>
<tr><td>circRNA_003486</td><td>15:7044923|7050105</td><td>circRNA_007934</td><td>7:91890126|91895327</td><td>circRNA_012136</td><td>GL896447.1:26049|52203</td><td>circRNA_002060</td><td>8:11361730|11791743</td></tr>
<tr><td>circRNA_007613</td><td>3:122639438|122644620</td><td>circRNA_015090</td><td>2:95629644|95634846</td><td>circRNA_002252</td><td>9:78959290|78985453</td><td>circRNA_018372</td><td>9:77428764|77865548</td></tr>
<tr><td>circRNA_001674</td><td>5:3457119|3462302</td><td>circRNA_005909</td><td>16:78181186|78186389</td><td>circRNA_014982</td><td>13:58123649|58149818</td><td>circRNA_008168</td><td>9:128160907|128604851</td></tr>
<tr><td>circRNA_012497</td><td>12:46917895|46923078</td><td>circRNA_020395</td><td>1:93271929|93277132</td><td>circRNA_005403</td><td>1:16139074|16165251</td><td>circRNA_014742</td><td>9:77278140|77725461</td></tr>
<tr><td>circRNA_007939</td><td>7:91964457|91969641</td><td>circRNA_006341</td><td>7:65184011|65189215</td><td>circRNA_011674</td><td>3:42027596|42053774</td><td>circRNA_000731</td><td>14:7879060|8335138</td></tr>
<tr><td>circRNA_019265</td><td>9:46856389|46861574</td><td>circRNA_004870</td><td>8:435392|440597</td><td>circRNA_008282</td><td>X:130737537|130763718</td><td>circRNA_001335</td><td>2:121661803|122118806</td></tr>
<tr><td>circRNA_005745</td><td>13:210047704|210052890</td><td>circRNA_010463</td><td>15:3815433|3820638</td><td>circRNA_000158</td><td>1:176208697|176234880</td><td>circRNA_010216</td><td>1:175695558|176159751</td></tr>
<tr><td>circRNA_011171</td><td>12:25019975|25025161</td><td>circRNA_017449</td><td>1:124875218|124880424</td><td>circRNA_012266</td><td>1:145436546|145462732</td><td>circRNA_020719</td><td>14:136774429|137241302</td></tr>
<tr><td>circRNA_015537</td><td>13:102373644|102378830</td><td>circRNA_018794</td><td>15:94277962|94283168</td><td>circRNA_011487</td><td>16:45789525|45815714</td><td>circRNA_020262</td><td>7:60758058|61224967</td></tr>
<tr><td>circRNA_017646</td><td>14:115555579|115560765</td><td>circRNA_004066</td><td>3:19864113|19869320</td><td>circRNA_014590</td><td>JH118963.1:42907|69109</td><td>circRNA_018113</td><td>11:70731187|71203103</td></tr>
<tr><td>circRNA_012242</td><td>1:120220840|120226027</td><td>circRNA_014410</td><td>3:133994994|134000202</td><td>circRNA_008652</td><td>12:23717648|23743858</td><td>circRNA_005794</td><td>14:83204457|83685562</td></tr>
<tr><td>circRNA_018640</td><td>13:72888860|72894047</td><td>circRNA_009877</td><td>8:103922072|103927281</td><td>circRNA_015981</td><td>3:61299839|61326049</td><td>circRNA_020278</td><td>8:11296931|11791743</td></tr>
<tr><td>circRNA_019785</td><td>7:4865345|4870532</td><td>circRNA_003943</td><td>2:83796563|83801773</td><td>circRNA_019428</td><td>10:49647635|49673847</td><td>circRNA_020078</td><td>16:37297286|37794392</td></tr>
<tr><td>circRNA_009477</td><td>4:89235641|89240851</td><td>circRNA_002107</td><td>8:82954008|83453422</td><td></td><td></td><td></td><td></td></tr>
</table>

Table S2-1. Differential expression analysis of circRNAs in the LH vs. LL comparison

circRNA_ID	Locus	Gene_id	GeneSymbol	Value (LH)	Value (LL)	log2 (FoldChange)	*P* value
circRNA_007796	6:25836379|25914846	n/a	_	620	0	-11.99801189	1.01E-11
circRNA_007082	13:202560796|202775160	n/a	_	84	0	-9.168728995	4.37E-06
circRNA_001382	3:9225531|9489014	n/a	_	0	228	9.794220993	7.83E-06
circRNA_007491	2:93830099|93833038	ENSSSCG00000014136,	VCAN	62.75	0	-8.76089517	2.13E-05
circRNA_006815	10:19943997|19946705	n/a	_	61.75	0	-8.639074113	3.22E-05
circRNA_007884	6:148118708|148135855	ENSSSCG00000021292,	OSBPL9	48.25	0	-8.247117894	0.000109589
circRNA_007499	2:105006195|105146567	n/a	_	75.5	0	-8.478137978	0.000110439
circRNA_002273	9:101923667|101924057	ENSSSCG00000029174,	CCDC126	0	40	8.315043915	0.000120104
circRNA_010775	6:156410969|156413339	n/a	_	77.75	0	-8.458129823	0.000145053
circRNA_002126	8:103694327|103712082	n/a	_	0	53.25	8.195495019	0.000216774
circRNA_001465	3:97935477|97953112	n/a	_	0	40.5	8.166770063	0.000238712
circRNA_008193	GL893520.1:9921|20513	n/a	_	40.25	0	-7.912133425	0.000306868
circRNA_007593	3:109680739|109684412	ENSSSCG00000008497,	GPATCH11	41.75	0	-7.900158676	0.000314117
circRNA_008142	9:97084813|97187229	n/a	_	38.75	0	-7.887715447	0.000325454
circRNA_000556	13:33494610|33528483	n/a	_	0	35.5	7.912522007	0.000374021
circRNA_007086	13:210034357|210039672	ENSSSCG00000012055,	MORC3	38	0	-7.769501852	0.000439263
circRNA_007267	15:97716153|97716961	ENSSSCG00000016017,	DNAJC10	45	0	-7.87230049	0.000451628
circRNA_002150	8:134379373|134477954	ENSSSCG00000009192,	PDLIM5	0	38.25	7.917609718	0.000458755
circRNA_010388	13:117603168|117605927	ENSSSCG00000030467,	PHC3	43.25	0	-7.858192817	0.000470354
circRNA_010354	13:18138045|18176107	ENSSSCG00000011222,	-	42.25	0	-7.842898496	0.000502889
circRNA_008171	9:132289659|132295195	ENSSSCG00000027614,	-	35.25	0	-7.717721265	0.000522868
circRNA_007030	13:92217338|92244533	ENSSSCG00000011686,	-	46.25	0	-7.811924454	0.000526997
circRNA_006918	12:19868684|19869889	ENSSSCG00000017378,	DHX8	36	0	-7.685323126	0.000560256
circRNA_010471	15:53787125|53792040	ENSSSCG00000015802,	-	39.75	0	-7.70959286	0.000690085
circRNA_010920	GL895500.1:2619|11834	ENSSSCG00000024266,	-	39.75	0	-7.662276413	0.000768856
circRNA_001514	3:122008499|122016380	ENSSSCG00000008591,	-	0	31.5	7.7115534	0.000776358
circRNA_010899	9:132262224|132368236	n/a	_	778	0	-8.267525137	0.000782213
circRNA_010609	3:25026227|25247061	n/a	_	39	0	-7.639824976	0.000815989
circRNA_010940	X:85941401|85961011	ENSSSCG00000012474,	DIAPH2	37.75	0	-7.602009389	0.000897513
circRNA_001088	17:14843150|14885851	n/a	_	1203	2637	1.304873183	0.000921559
circRNA_000136	1:143460231|143474541	ENSSSCG00000004718,	TTBK2	0	29.25	7.63386913	0.000926429
circRNA_007459	2:72132914|72198308	n/a	_	32	0	-7.408803645	0.001115151
circRNA_000012	1:15907346|15956308	n/a	_	0	27.25	7.537988547	0.001164572
circRNA_010653	4:33426747|33460740	ENSSSCG00000006037,	-	35.25	0	-7.487844345	0.001193653
circRNA_001240	2:43788778|43816968	n/a	_	0	368.75	8.003952678	0.001202646
circRNA_006611	1:18675426|18725069	ENSSSCG00000028867,	PPIL4	31.75	0	-7.366839626	0.001241393
circRNA_008244	X:30820948|30910575	n/a	_	36.5	0	-7.458544884	0.001257395
circRNA_007786	6:10251024|10299712	n/a	_	35.25	0	-7.465129412	0.001266736
circRNA_000813	14:81008366|81039544	ENSSSCG00000023130,	-	0	26.5	7.420242308	0.00126681
circRNA_003408	14:94770180|94775658	ENSSSCG00000010357,	WAPL	0	35.5	7.484470836	0.001284673
circRNA_004920	8:50518546|50565554	ENSSSCG00000008881,	RAPGEF2	0	37.5	7.451895461	0.001376672
circRNA_007372	17:59438248|59456350	ENSSSCG00000007478,	ATP9A	28.75	0	-7.311651243	0.001413489
circRNA_000527	13:3592151|3595080	n/a	_	0	29.25	7.439690091	0.001434453
circRNA_002235	9:55394479|55420953	n/a	_	0	216.25	7.844902612	0.001547531
circRNA_006665	1:106924970|106964621	ENSSSCG00000004505,	SMAD2	33.5	0	-7.338499808	0.001651725
circRNA_007191	14:136612215|136643431	ENSSSCG00000010654,	ATRNL1	28	0	-7.233861901	0.001662327
circRNA_006726	1:188117293|188127728	ENSSSCG00000004979,	-	26.75	0	-7.188581573	0.001859183
circRNA_007831	6:74096530|74110479	ENSSSCG00000003520,	CDC42	26.75	0	-7.16446166	0.001922409
circRNA_002833	10:51236326|51267368	ENSSSCG00000011047,	FAM171A1	0	35.25	7.288669743	0.001969798

circRNA_010906	9:141723625|141736681	ENSSSCG00000015581,	CENPF	32	0	-7.234367585	0.002065892
circRNA_004513	6:869929|870256	n/a	_	0	35.25	7.26092898	0.002084795
circRNA_000911	15:54007386|54165794	n/a	_	0	31	7.259070053	0.002093423
circRNA_012118	GL893884.1:31770|41304	ENSSSCG00000027457,	UBE2Q2	263.75	0	-7.636971228	0.002133384
circRNA_000656	13:132927553|132943099	ENSSSCG00000011793,	LIPH	0	33	7.238383863	0.002201858
circRNA_007251	15:79278095|79284066	ENSSSCG00000015901,	GRB14	25.5	0	-7.085612572	0.002293155
circRNA_002108	8:82960858|83064254	ENSSSCG00000009021,	-	0	29	7.193131273	0.002401544
circRNA_007301	16:23404831|23405864	ENSSSCG00000016842,	NIPBL	34.5	0	-7.161702793	0.002409893
circRNA_006627	1:44537106|44540267	ENSSSCG00000025075,	-	33.25	0	-7.13746056	0.002526349
circRNA_000709	13:207175610|207194401	n/a	_	0	28.75	7.153346939	0.002598298
circRNA_001057	16:37377919|37382673	ENSSSCG00000016917,	ANKRD55	0	26.75	7.1538952	0.002601784
circRNA_010404	13:156006479|156014054	ENSSSCG00000024663,	SPICE1	31.25	0	-7.113861709	0.002689587
circRNA_002439	X:121797075|121823999	ENSSSCG00000012649,	SMARCA1	0	24.5	7.134000634	0.002722323
circRNA_003637	15:128043602|128078889	ENSSSCG00000016166,	-	0	28.75	7.11756816	0.002789525
circRNA_007243	15:66307639|66311057	ENSSSCG00000015861,	WDR33	29.25	0	-7.080553277	0.002883481
circRNA_006814	10:17555075|17567128	ENSSSCG00000010866,	SCCPDH	29.75	0	-7.077338247	0.002890186
circRNA_003666	16:23393877|23396120	ENSSSCG00000016842,	NIPBL	0	28.75	7.092953815	0.002941498
circRNA_010257	1:307315009|307320696	ENSSSCG00000005743,	VAV2	29.75	0	-7.061633145	0.00295419
circRNA_007109	14:33386567|33391511	ENSSSCG00000009818,	-	30	0	-7.054213421	0.002999439
circRNA_001328	2:119260427|119266346	ENSSSCG00000014195,	MAN2A1	0	19.75	6.989068818	0.003085889
circRNA_001787	6:31800795|31818387	n/a	_	0	24.5	7.063025198	0.003129201
circRNA_004895	8:31876392|31947867	ENSSSCG00000008787,	KLHL5	0	30.75	7.03695722	0.00325149
circRNA_007134	14:60097680|60107836	ENSSSCG00000010156,	ARID4B	23	0	-6.895232045	0.003311945
circRNA_010820	8:9814040|9871034	ENSSSCG00000008736,	CPEB2	28	0	-7.000849991	0.003354698
circRNA_007470	2:82120327|82157445	ENSSSCG00000014044,	NSD1	30.25	0	-6.975470325	0.003499256
circRNA_006599	1:6464869|6486326	ENSSSCG00000004029,	QKI	27.5	0	-6.977729139	0.003516321
circRNA_002715	1:251516590|251552157	n/a	_	0	152	7.279973155	0.003625009
circRNA_001292	2:86313971|86318262	ENSSSCG00000014083,	ANKDD1B	0	22.25	6.981884378	0.003660424
circRNA_003806	18:6042153|6048736	ENSSSCG00000016434,	RHEB	0	28.5	6.960447564	0.003757399
circRNA_000188	1:205472022|205484559	ENSSSCG00000005060,	KTN1	0	20.25	6.96696893	0.003784188
circRNA_007383	18:10898787|10903368	ENSSSCG00000016510,	UBN2	27.5	0	-6.916575028	0.003927842
circRNA_007794	6:18424722|18447166	ENSSSCG00000002799,	CNOT1	27.75	0	-6.912820696	0.00395285
circRNA_007728	5:17107980|17112458	ENSSSCG00000029196,	DIP2B	27.75	0	-6.912820696	0.00395285
circRNA_001295	2:87204233|87215862	ENSSSCG00000014088,	-	55	185.25	1.856080967	0.003980008
circRNA_007147	14:71895250|71904925	ENSSSCG00000010226,	JMJD1C	26.25	0	-6.908793127	0.00406046
circRNA_008030	8:85966163|85981573	ENSSSCG00000009029,	ARHGAP10	28.25	0	-6.884335345	0.004157088
circRNA_007341	17:10389674|10421420	n/a	_	29.75	0	-6.882535148	0.004220114
circRNA_001596	4:79089337|79096209	ENSSSCG00000006231,	CHD7	0	22.25	6.895249596	0.004273696
circRNA_003230	13:148398444|148408637	n/a	_	0	24.25	6.855114089	0.004587241
circRNA_007395	18:27396026|27448992	ENSSSCG00000016618,	CPED1	26.75	0	-6.831526644	0.004593728
circRNA_002029	7:102785672|102822653	n/a	_	283	614	1.357885962	0.004751221
circRNA_007321	16:47827438|47829752	ENSSSCG00000016953,	-	27.5	0	-6.772992532	0.005096209
circRNA_007677	4:102444968|102450907	ENSSSCG00000006487,	CCT3	27.75	0	-6.769560669	0.005129135
circRNA_006865	11:12807920|12827511	n/a	_	25	0	-6.77238651	0.005152859
circRNA_001520	3:122510653|122518540	n/a	_	0	24.25	6.779586464	0.005225183
circRNA_008114	9:50848480|50856753	ENSSSCG00000029649,	IFT46	25.5	0	-6.752178005	0.005316987
circRNA_003708	16:47778049|47825304	n/a	_	0	24.25	6.760338106	0.005384774
circRNA_007189	14:132243465|132256940	ENSSSCG00000010627,	PDCD4	26.25	0	-6.735679462	0.005447097
circRNA_007528	3:9324579|9335045	ENSSSCG00000007681,	CUX1	26.5	0	-6.73440554	0.005450585
circRNA_001477	3:102273355|102294111	ENSSSCG00000008448,	PREPL	0	20	6.754872519	0.005487973
circRNA_010803	7:80266702|80267078	ENSSSCG00000001991,	DHRS1	23.5	0	-6.705740182	0.005790673
circRNA_000726	14:933854|949498	ENSSSCG00000009583,	-	0	20.25	6.718589942	0.005827443

circRNA_002453	X:143993560|144011055	n/a	_	259.5	581	1.41329036	0.005874453
circRNA_003889	2:19750076|19758365	ENSSSCG00000013281,	EXT2	0	24	6.70988469	0.005878265
circRNA_003232	13:150105423|150126999	ENSSSCG00000011901,	TMEM39A	0	22	6.6730486	0.006262925
circRNA_008129	9:77414305|77428858	ENSSSCG00000015307,	-	25	0	-6.655774223	0.00631986
circRNA_000778	14:53600684|53609301	ENSSSCG00000010081,	MAPK1	0	20	6.655050683	0.006457661
circRNA_002358	GL895028.1:36544|44671	ENSSSCG00000025306,	-	0	22	6.654863866	0.006473181
circRNA_008006	8:43554347|43560434	ENSSSCG00000008842,	KIT	25.5	0	-6.627212676	0.006550158
circRNA_008147	9:101480476|101486805	ENSSSCG00000026353,	-	25.5	0	-6.624269603	0.006610023
circRNA_002272	9:101637290|101643291	ENSSSCG00000015387,	TRA2A	0	20	6.638344442	0.006644657
circRNA_001856	6:97024076|97096660	n/a	_	315.5	109.25	-1.388299333	0.006665997
circRNA_006607	1:16603165|16603500	ENSSSCG00000004084,	-	23.5	0	-6.61753635	0.00669356
circRNA_007808	6:39555556|39559624	ENSSSCG00000002876,	-	23.75	0	-6.611978173	0.006725161
circRNA_006593	1:2150622|2238552	n/a	_	24.5	0	-6.601588517	0.006850611
circRNA_007546	3:33370869|33378455	ENSSSCG00000020675,	ATF7IP2	24	0	-6.598858912	0.006867161
circRNA_002332	GL893394.1:17824|55189	ENSSSCG00000027571,	-	0	18	6.607583342	0.007004113
circRNA_000875	14:145928322|145990152	n/a	_	0	18	6.607583342	0.007004113
circRNA_001237	2:39869318|40051068	n/a	_	0	18	6.607583342	0.007004113
circRNA_007402	18:42650214|42689446	ENSSSCG00000027063,	DPY19L1	22.25	0	-6.579004881	0.00712812
circRNA_010565	2:29219247|29225004	ENSSSCG00000013305,	CAPRIN1	21.5	0	-6.568740543	0.00729211
circRNA_007127	14:52135828|52247318	n/a	_	22.5	0	-6.562433044	0.007308493
circRNA_007714	5:4603986|4611101	ENSSSCG00000000068,	EP300	21.75	0	-6.563271643	0.007325216
circRNA_003893	2:30338578|30343892	ENSSSCG00000020674,	DEPDC7	0	22	6.561570176	0.0074748
circRNA_007974	7:130799674|130851846	n/a	_	65	0	-6.756999136	0.007496313
circRNA_005041	9:386764|389227	ENSSSCG00000014578,	DENND5A	0	24	6.555992665	0.007552639
circRNA_005271	GL896137.1:66267|74377	ENSSSCG00000025188,	-	0	24	6.555992665	0.007552639
circRNA_002321	GL892430.1:39370|62058	ENSSSCG00000021380,	UBXN7	0	17.75	6.540138521	0.007784286
circRNA_003041	12:49414058|49436273	ENSSSCG00000017812,	VPS53	0	19.75	6.534348851	0.007824308
circRNA_004144	3:92450641|92461755	ENSSSCG00000008412,	PSME4	0	19.75	6.534348851	0.007824308
circRNA_002380	GL896504.1:52653|55460	ENSSSCG00000027115,	-	0	19.75	6.531604946	0.007852364
circRNA_006702	1:154139505|154150097	ENSSSCG00000004813,	TTC23	23.75	0	-6.511650245	0.007938631
circRNA_007110	14:33910035|33922776	ENSSSCG00000009826,	TCTN1	23.75	0	-6.511650245	0.007938631
circRNA_007675	4:98044696|98065430	ENSSSCG00000006386,	COPA	23	0	-6.502730484	0.007991655
circRNA_002718	1:254931586|254937077	n/a	_	118.5	21.75	-2.53805923	0.007996974
circRNA_007784	6:871815|872594	n/a	_	23.25	0	-6.50206332	0.008016551
circRNA_010222	1:195020817|195029660	ENSSSCG00000027765,	FAM179B	56	0	-6.69113916	0.00818146
circRNA_006952	12:59263043|59266273	ENSSSCG00000018014,	ZNF18	23.5	0	-6.492702333	0.008219847
circRNA_006868	11:19157893|19158675	ENSSSCG00000029933,	-	23	0	-6.484184614	0.008281721
circRNA_007799	6:30402013|30407779	ENSSSCG00000002837,	-	23	0	-6.484184614	0.008281721
circRNA_007182	14:122455302|122456060	ENSSSCG00000010566,	FBXW4	21.5	0	-6.481321919	0.008288397
circRNA_010851	8:124119181|124229272	n/a	_	57.25	0	-6.671244847	0.008398986
circRNA_006723	1:181050309|181059423	ENSSSCG00000004934,	DPP8	20.5	0	-6.456785433	0.008670521
circRNA_011030	1:195112533|195130686	ENSSSCG00000027765,	FAM179B	59.75	0	-6.644203748	0.008702807
circRNA_002241	9:69056402|69057120	ENSSSCG00000024613,	GUCY1A2	0	36.5	6.641044083	0.008744894
circRNA_000912	15:55069306|55072396	ENSSSCG00000015814,	TACC1	0	17.75	6.456022804	0.00884352
circRNA_001378	3:5151251|5152058	n/a	_	0	19.5	6.454166916	0.008847565
circRNA_002500	1:56096587|56109914	ENSSSCG00000004272,	LMBRD1	0	19.75	6.448269101	0.008922565
circRNA_010300	11:495509|513694	ENSSSCG00000026744,	ZDHHC20	48.25	0	-6.589295127	0.009349585
circRNA_004163	3:107521846|107608026	n/a	_	0	19.75	6.39847773	0.009558509
circRNA_007997	8:33308376|33317260	ENSSSCG00000022168,	APBB2	21.5	0	-6.380835209	0.009664813
circRNA_006630	1:48408106|48487606	n/a	_	21.5	0	-6.377515577	0.009699951
circRNA_006666	1:107929920|107933933	ENSSSCG00000004506,	CTIF	21	0	-6.369562894	0.009781496
circRNA_007797	6:25841148|25914846	n/a	_	20.75	0	-6.367550049	0.0098034

circRNA_007180	14:121761204|121809144	n/a	_	21.75	0	-6.368539613	0.00989079
circRNA_007850	6:99544759|99547687	ENSSSCG00000025478,	MIB1	20.75	0	-6.356737311	0.00998665
circRNA_006787	1:287936886|287960703	ENSSSCG00000005498,	PAPPA	20.5	0	-6.35409396	0.010023131
circRNA_007654	4:55368160|55379564	ENSSSCG00000006137,	WWP1	19	0	-6.332796127	0.010369458
circRNA_000479	12:37398861|37421064	ENSSSCG00000017670,	RPS6KB1	0	15.5	6.347699006	0.010383682
circRNA_001175	18:25506902|25510551	ENSSSCG00000016608,	IQUB	0	15.5	6.347699006	0.010383682
circRNA_000629	13:104105327|104248782	n/a	_	38	123.5	1.825454873	0.010402525
circRNA_003175	13:91488790|91495754	ENSSSCG00000011684,	U2SURP	0	17.5	6.328496026	0.010608628
circRNA_002850	10:62738557|62767160	ENSSSCG00000011103,	PARD3	0	17.5	6.328496026	0.010608628
circRNA_008199	GL893884.1:31770|40337	ENSSSCG00000027457,	UBE2Q2	19.75	0	-6.31012475	0.010694379
circRNA_007642	4:39835278|39859777	n/a	_	19.5	0	-6.305413213	0.010801025
circRNA_005130	9:73323019|73336315	ENSSSCG00000015645,	-	0	17.5	6.313081386	0.010859196
circRNA_010446	14:108816383|108826406	ENSSSCG00000010438,	ATAD1	17.75	0	-6.303135909	0.010908949
circRNA_007520	2:147053805|147054323	ENSSSCG00000014343,	-	43	0	-6.45747173	0.011077074
circRNA_003042	12:50480103|50481892	ENSSSCG00000017836,	PAFAH1B1	0	17.5	6.288134601	0.011217289
circRNA_002786	10:24473176|24475663	ENSSSCG00000010894,	TP53BP2	0	17.5	6.288134601	0.011217289
circRNA_010754	6:103836621|103854303	ENSSSCG00000003718,	TAF4B	17.75	0	-6.270386525	0.01132692
circRNA_001491	3:109787455|109805619	ENSSSCG00000008498,	HEATR5B	0	13.25	6.288417826	0.011354016
circRNA_010607	3:24575402|24632228	n/a	_	43	0	-6.429968083	0.011470721
circRNA_010763	6:128736988|128830137	n/a	_	43	0	-6.429968083	0.011470721
circRNA_008095	9:29830958|29833681	ENSSSCG00000014949,	MED17	45.5	0	-6.428420001	0.011493236
circRNA_007789	6:16261615|16309258	ENSSSCG00000002755,	NFAT5	19.25	0	-6.247778446	0.011667119
circRNA_006738	1:202793118|202798180	ENSSSCG00000005041,	FERMT2	19.25	0	-6.247778446	0.011667119
circRNA_007005	13:73331002|73340904	ENSSSCG00000011563,	FANCD2	19.25	0	-6.241748151	0.011766295
circRNA_007179	14:120779066|120790937	ENSSSCG00000010548,	CHUK	19.25	0	-6.241748151	0.011766295
circRNA_005049	9:8944659|8950452	ENSSSCG00000014828,	-	0	17.5	6.252318142	0.011808487
circRNA_007176	14:120768685|120779168	ENSSSCG00000010548,	CHUK	18.75	0	-6.23332331	0.011864202
circRNA_007385	18:15081212|15102829	ENSSSCG00000016535,	CALD1	18.75	0	-6.222456655	0.012076018
circRNA_010758	6:119320977|119321733	ENSSSCG00000003756,	LPAR3	41.25	0	-6.384060632	0.01215495
circRNA_010308	11:15976293|15979955	ENSSSCG00000009376,	VPS36	37.75	0	-6.375491173	0.012286532
circRNA_010697	5:32847751|33033688	n/a	_	38.5	0	-6.374483018	0.012302093
circRNA_010577	2:88879257|89138199	n/a	_	38.5	0	-6.36265443	0.012485954
circRNA_008084	9:13256666|13259345	ENSSSCG00000014881,	CLNS1A	17.75	0	-6.190937545	0.01263665
circRNA_008261	X:77199964|77219590	ENSSSCG00000012456,	RPS6KA6	17.75	0	-6.190937545	0.01263665
circRNA_011873	6:50954116|50979886	ENSSSCG00000027407,	-	41	0	-6.344956796	0.012765498
circRNA_010345	12:48061363|48414455	n/a	_	35	0	-6.343723611	0.012785178
circRNA_001479	3:102415331|102422235	n/a	_	0	25	6.333895054	0.01295405
circRNA_003253	13:202560796|202781523	n/a	_	0	43.75	6.329220911	0.013029782
circRNA_006624	1:31299808|31542935	n/a	_	17.5	0	-6.167302244	0.013057296
circRNA_006847	10:56160513|56208948	ENSSSCG00000011075,	KIAA1217	40.5	0	-6.318696919	0.013190296
circRNA_008073	9:250376|261322	ENSSSCG00000014579,	-	37.25	0	-6.310951886	0.013317903
circRNA_004975	8:93140698|93168617	ENSSSCG00000030396,	SETD7	0	15.25	6.165783783	0.013338481
circRNA_002661	1:202606369|202619041	ENSSSCG00000005038,	PSMC6	0	15.25	6.165783783	0.013338481
circRNA_010237	1:227193880|227197323	ENSSSCG00000005170,	DENND4C	15.75	0	-6.15466072	0.013350226
circRNA_007468	2:80452934|80462260	ENSSSCG00000014020,	CANX	33.25	0	-6.305073589	0.013415467
circRNA_011788	4:110330609|110415274	n/a	_	38	0	-6.294961104	0.013584755
circRNA_000902	15:36556955|36583358	n/a	_	0	24.75	6.2867859	0.013735102
circRNA_010418	13:212995887|213000535	ENSSSCG00000028428,	-	16.25	0	-6.127647532	0.013814999
circRNA_010263	10:16978306|17000982	ENSSSCG00000010862,	CDC42BPA	15.5	0	-6.125488771	0.013825536
circRNA_010696	5:31935410|31975642	n/a	_	15.5	0	-6.125488771	0.013825536
circRNA_010734	6:33100577|33107171	ENSSSCG00000022333,	C16orf87	15.5	0	-6.125488771	0.013825536
circRNA_010951	1:14083797|14085261	ENSSSCG00000004070,	SCAF8	36.25	0	-6.274204424	0.013938041

circRNA_005237	GL893884.1:23405|47747	ENSSSCG00000027457,	UBE2Q2	0	15.25	6.131561316	0.01396538
circRNA_010702	5:51910032|51916859	ENSSSCG00000027144,	LMNTD1	15.75	0	-6.111872571	0.014063308
circRNA_007077	13:167100656|167103460	ENSSSCG00000021791,	SENP7	17	0	-6.105803531	0.014128532
circRNA_003448	14:124568819|124695046	n/a	_	0	26.75	6.251905491	0.014339391
circRNA_002226	9:44916087|44925357	n/a	_	0	26.75	6.25025345	0.014368573
circRNA_008214	GL895204.1:2317|4664	n/a	_	17	0	-6.094648516	0.014376002
circRNA_007007	13:79420085|79420841	ENSSSCG00000011616,	-	17	0	-6.094648516	0.014376002
circRNA_007951	7:102242791|102244568	ENSSSCG00000028663,	RBM25	31.5	0	-6.247985266	0.014395645
circRNA_000509	12:53698948|53721538	ENSSSCG00000017895,	-	0	24.75	6.2471574	0.014423402
circRNA_007529	3:10470262|10488752	ENSSSCG00000027342,	BAZ1B	16.75	0	-6.091650982	0.014430055
circRNA_010422	14:26145419|26295541	n/a	_	35	0	-6.245943136	0.014431826
circRNA_005993	2:81251743|81304602	n/a	_	0	33	6.246502251	0.014435027
circRNA_008010	8:57803396|57816363	ENSSSCG00000008900,	SRP72	16.25	0	-6.075146111	0.01470117
circRNA_001214	2:4285881|4579464	n/a	_	4.25	66.5	3.753568783	0.014728569
circRNA_010427	14:31364465|31372689	ENSSSCG00000009779,	MPHOSPH9	29.5	0	-6.228843127	0.014737884
circRNA_006913	12:14691671|14701912	ENSSSCG00000017277,	PECAM1	16	0	-6.067946889	0.014867818
circRNA_010322	12:12797259|12804024	n/a	_	31.75	0	-6.218314408	0.014929103
circRNA_010284	10:46613551|46681760	ENSSSCG00000011025,	-	29.25	0	-6.216445168	0.014963275
circRNA_004606	6:89159443|89216201	ENSSSCG00000003670,	RLF	0	26.75	6.216139844	0.014982757
circRNA_006012	2:90128254|90132036	ENSSSCG00000014114,	PAPD4	0	33	6.215507341	0.014994355
circRNA_002112	8:83452129|83472898	ENSSSCG00000009021,	-	0	18.5	6.214092933	0.01502032
circRNA_008148	9:101484691|101492888	ENSSSCG00000026353,	-	33.25	0	-6.208059146	0.015117412
circRNA_005571	10:76253466|76306111	ENSSSCG00000011166,	ZMYND11	0	31	6.20780085	0.015136296
circRNA_010518	17:5196690|5217932	ENSSSCG00000006981,	MICU3	28.75	0	-6.196628103	0.01532972
circRNA_004269	4:45282363|45283104	ENSSSCG00000006098,	DPY19L4	0	24.5	6.194903456	0.015376441
circRNA_001180	18:25520260|25551680	ENSSSCG00000016608,	IQUB	0	22.5	6.189711843	0.01547403
circRNA_000351	10:58881715|58909767	ENSSSCG00000011085,	MLLT10	0	22.5	6.189711843	0.01547403
circRNA_002327	GL892843.1:74235|80602	ENSSSCG00000029109,	MTMR9	0	20.5	6.189570236	0.015476699
circRNA_004106	3:73358334|73374822	ENSSSCG00000022848,	-	0	35	6.179188986	0.015673474
circRNA_004457	5:70582741|70588176	ENSSSCG00000000755,	ERC1	0	24.5	6.175788305	0.0157384
circRNA_006095	3:104192112|104199715	ENSSSCG00000008467,	EML4	0	24.5	6.173801748	0.015776435
circRNA_000689	13:167100656|167104720	ENSSSCG00000021791,	SENP7	0	18.25	6.151894886	0.016201147
circRNA_001809	6:63443230|63452235	ENSSSCG00000025445,	-	0	22.5	6.144305118	0.016350569
circRNA_007707	4:142643211|142645666	n/a	_	27.25	0	-6.14310163	0.016358428
circRNA_010625	3:82691426|82695002	n/a	_	13.75	0	-5.989237734	0.01656195
circRNA_006853	10:71303539|71307626	ENSSSCG00000011137,	GDI2	30.75	0	-6.132374321	0.016571587
circRNA_000949	15:84437803|84439082	ENSSSCG00000015923,	NOSTRIN	0	18.25	6.125057488	0.016734833
circRNA_007390	18:20672465|20675630	ENSSSCG00000016572,	TNPO3	32.25	0	-6.12207198	0.016778546
circRNA_007440	2:48076528|48078432	ENSSSCG00000021317,	-	31.75	0	-6.121993041	0.01678014
circRNA_000158	1:176208697|176234880	ENSSSCG00000004896,	PHLPP1	0	20.25	6.116580312	0.016906518
circRNA_012123	GL894597.1:33450|45776	n/a	_	29	0	-6.114409681	0.016933907
circRNA_004617	6:94778502|94788895	ENSSSCG00000003687,	EPB41L3	0	28.5	6.110241722	0.017035881
circRNA_000465	12:21068070|21075089	ENSSSCG00000017415,	TTC25	0	18	6.109952187	0.01704181
circRNA_011134	11:5274155|5288837	ENSSSCG00000009319,	PAN3	29.75	0	-6.101750459	0.017193294
circRNA_007726	5:16683843|16694950	ENSSSCG00000028516,	LARP4	30.5	0	-6.100790884	0.017213094
circRNA_010455	14:123996752|124002504	ENSSSCG00000010593,	CNNM2	27.75	0	-6.1001597	0.017226128
circRNA_006175	4:112851286|112891675	ENSSSCG00000006728,	GDAP2	0	24.25	6.097708923	0.017294164
circRNA_001144	18:5395091|5439747	n/a	_	0	16	6.09287272	0.017394727
circRNA_002334	GL893684.1:19920|20953	ENSSSCG00000030514,	-	0	16	6.09287272	0.017394727
circRNA_004772	7:58507983|58508405	ENSSSCG00000001818,	FES	0	22.25	6.090842667	0.017437088
circRNA_010362	13:35728481|35735863	ENSSSCG00000011394,	RBM6	26	0	-6.087701568	0.017485139
circRNA_007169	14:114857540|114878790	n/a	_	14.75	0	-5.942451112	0.017505721

circRNA_008089	9:19259967|19300140	ENSSSCG00000014902,	ANKRD42	24.75	0	-6.082501287	0.017594241
circRNA_000811	14:78371743|78385948	n/a	_	0	22.25	6.08275148	0.017606811
circRNA_000077	1:101186411|101191510	ENSSSCG00000020702,	SENP6	0	22.25	6.08275148	0.017606811
circRNA_006715	1:173582741|173767461	n/a	_	25.25	0	-6.079965773	0.017647648
circRNA_006778	1:280497623|280502159	ENSSSCG00000005441,	IKBKAP	14.5	0	-5.931838824	0.017740157
circRNA_008108	9:42973775|42993947	ENSSSCG00000015013,	RDX	14.5	0	-5.931838824	0.017740157
circRNA_005932	17:55857987|55860379	ENSSSCG00000007458,	NCOA3	0	26.5	6.075038304	0.017769922
circRNA_011662	3:19058493|19062370	ENSSSCG00000007812,	XPO6	27.75	0	-6.073068696	0.017793628
circRNA_008067	8:144596236|144599873	ENSSSCG00000009241,	COPS4	25.5	0	-6.07103451	0.017836879
circRNA_003430	14:117093628|117107550	n/a	_	0	22.25	6.068806571	0.01790265
circRNA_011455	15:148789557|148861405	n/a	_	27.25	0	-6.064233262	0.017982146
circRNA_003059	13:1141433|1164999	ENSSSCG00000011183,	-	0	28.5	6.064437054	0.017996221
circRNA_007252	15:79516524|79520402	n/a	_	29.25	0	-6.06220077	0.018025754
circRNA_001321	2:112811828|112831718	n/a	_	0	18	6.059166073	0.018109654
circRNA_006789	1:293365114|293369407	n/a	_	25.5	0	-6.053505023	0.018213349
circRNA_007042	13:119253884|119258239	ENSSSCG00000011750,	PLD1	28.5	0	-6.042062765	0.018462741
circRNA_007590	3:106723747|106724618	ENSSSCG00000008470,	THUMPD2	26.25	0	-6.041587272	0.018473168
circRNA_011971	7:109814818|109851924	n/a	_	27.25	0	-6.040058514	0.018506725
circRNA_000731	14:7879060|8335138	n/a	_	0	15.75	6.04001725	0.018526916
circRNA_011334	14:62469263|62480280	ENSSSCG00000010169,	SIPA1L2	26.75	0	-6.0308166	0.018710703
circRNA_006944	12:44742567|44746693	ENSSSCG00000017743,	CRLF3	27	0	-6.023268606	0.018878718
circRNA_004232	4:15264775|15272443	ENSSSCG00000005970,	SQLE	0	22.25	6.022700866	0.018911311
circRNA_003290	14:7103656|7106034	ENSSSCG00000009623,	SLC39A14	0	20	6.019174736	0.018990416
circRNA_010807	7:97005367|97079790	ENSSSCG00000002285,	GPHN	22.5	0	-6.011945375	0.019133189
circRNA_010383	13:90946479|90950445	ENSSSCG00000011678,	XRN1	22.5	0	-6.011945375	0.019133189
circRNA_010398	13:145987809|146004021	n/a	_	22.5	0	-6.011945375	0.019133189
circRNA_007739	5:38471187|38482048	ENSSSCG00000000511,	RAB21	26	0	-6.010803913	0.019159003
circRNA_002993	12:31875147|31915569	ENSSSCG00000017602,	-	0	26.25	6.008041356	0.019242039
circRNA_002215	9:40148558|40154700	ENSSSCG00000024628,	ALKBH8	0	15.75	6.00724813	0.019260075
circRNA_001081	17:728755|734222	ENSSSCG00000006968,	LONRF1	0	18	6.004712569	0.019317824
circRNA_007586	3:104197942|104199715	ENSSSCG00000008467,	EML4	27.25	0	-6.000039625	0.019403907
circRNA_004692	6:147045770|147067936	ENSSSCG00000003853,	-	0	24	6.00034515	0.019417642
circRNA_006969	13:18089043|18140422	ENSSSCG00000011222,	-	23.25	0	-5.991883339	0.019591252
circRNA_011483	16:36595410|36602208	ENSSSCG00000016911,	SKIV2L2	24.5	0	-5.991017366	0.019611233
circRNA_004599	6:88173912|88196465	ENSSSCG00000003654,	MACF1	0	24.25	5.99102822	0.019632057
circRNA_010726	5:106543058|106572931	n/a	_	22.5	0	-5.988376982	0.019672264
circRNA_000170	1:200269189|200281803	ENSSSCG00000005015,	SOS2	0	15.75	5.986935676	0.019726878
circRNA_012119	GL894386.1:27340|42489	ENSSSCG00000004921,	ATP8B1	25.75	0	-5.986016765	0.019726956
circRNA_001127	17:57540295|57575864	n/a	_	0	15.75	5.984053781	0.019793884
circRNA_001076	16:75043882|75057142	ENSSSCG00000017073,	FAM114A2	0	15.75	5.984053781	0.019793884
circRNA_001451	3:81720523|81867496	n/a	_	0	15.75	5.984053781	0.019793884
circRNA_010749	6:79809424|79853129	ENSSSCG00000003586,	EPB41	21.5	0	-5.983029161	0.019796373
circRNA_010283	10:44197866|44204806	ENSSSCG00000011013,	WAC	23	0	-5.981400849	0.019834291
circRNA_010440	14:68165377|68175427	ENSSSCG00000010211,	CCDC6	22.5	0	-5.979503835	0.019878549
circRNA_006797	1:304822634|304823214	ENSSSCG00000005706,	ABL1	24	0	-5.978941646	0.019891681
circRNA_011685	3:60805128|60822724	ENSSSCG00000008216,	RMND5A	25.25	0	-5.976070064	0.019958873
circRNA_010833	8:49959084|49960052	ENSSSCG00000008875,	RXFP1	23.25	0	-5.974114401	0.020004744
circRNA_002599	1:142976243|142977395	ENSSSCG00000004710,	ADAL	0	20	5.969799831	0.020128161
circRNA_000983	15:106500764|106503077	ENSSSCG00000016053,	NEMP2	0	20	5.969537568	0.020134357
circRNA_007749	5:49928478|49989587	n/a	_	25.25	0	-5.966267159	0.020189712
circRNA_004878	8:9736245|9871034	n/a	_	0	17.75	5.965965972	0.020218889
circRNA_010506	16:47791556|47817458	ENSSSCG00000016953,	-	21.25	0	-5.964520493	0.02023108

circRNA_010534	18:1417740|1420536	ENSSSCG00000026652,	-	21.25	0	-5.964520493	0.02023108
circRNA_005700	13:97307290|97314870	ENSSSCG00000025293,	HLTF	0	22	5.95930237	0.020377413
circRNA_010228	1:204835343|204836699	ENSSSCG00000005052,	WDHD1	21	0	-5.946711144	0.020657025
circRNA_005203	9:143478477|143491288	ENSSSCG00000015590,	FLVCR1	0	24	5.947031144	0.020672114
circRNA_004206	3:123743295|123751557	n/a	_	0	20	5.943858233	0.020748902
circRNA_003417	14:104732283|104759197	ENSSSCG00000010426,	PCDH15	0	20	5.943858233	0.020748902
circRNA_003597	15:111197129|111198713	ENSSSCG00000016068,	-	0	20	5.943858233	0.020748902
circRNA_004484	5:86283657|86292147	ENSSSCG00000023972,	DRAM1	0	20	5.943858233	0.020748902
circRNA_004283	4:67884090|67893087	ENSSSCG00000022013,	-	60.75	4.25	-3.571639023	0.020846296
circRNA_007950	7:102205505|102229168	ENSSSCG00000028663,	RBM25	20.75	0	-5.937822583	0.020872455
circRNA_010655	4:34617495|34641595	ENSSSCG00000006038,	ZFPM2	11.75	0	-5.802073104	0.020891649
circRNA_007579	3:97836618|97908682	n/a	_	24.75	0	-5.935143519	0.020937761
circRNA_006698	1:145435888|145438147	ENSSSCG00000004753,	INO80	25	0	-5.933676436	0.020973596
circRNA_007643	4:39885800|39888370	ENSSSCG00000006069,	RGS22	24.75	0	-5.93208314	0.021012574
circRNA_008161	9:120426043|120436242	ENSSSCG00000015455,	CUL1	25	0	-5.930610307	0.02104866
circRNA_010168	1:31299808|31541885	n/a	_	20.75	0	-5.928470008	0.021101194
circRNA_010630	3:100584279|100593451	ENSSSCG00000008444,	PRKCE	20.75	0	-5.928470008	0.021101194
circRNA_001068	16:51704344|51708056	ENSSSCG00000016970,	-	0	15.75	5.929298348	0.021104391
circRNA_000937	15:73337972|73339303	ENSSSCG00000015880,	-	0	15.75	5.929298348	0.021104391
circRNA_000802	14:77561976|77573487	ENSSSCG00000010239,	RUFY2	0	15.75	5.929298348	0.021104391
circRNA_002001	7:73514061|73523294	ENSSSCG00000001974,	G2E3	0	15.75	5.929298348	0.021104391
circRNA_000563	13:34853744|34867936	ENSSSCG00000011367,	ARIH2	0	13.75	5.929032894	0.02111092
circRNA_001208	2:3009236|3029700	ENSSSCG00000012884,	PPP6R3	0	13.75	5.929032894	0.02111092
circRNA_001585	4:56555763|56556388	ENSSSCG00000006146,	-	0	13.75	5.929032894	0.02111092
circRNA_001887	6:108161644|108202404	ENSSSCG00000025005,	B4GALT6	0	15.75	5.923651149	0.02124366
circRNA_006795	1:301515256|301535195	ENSSSCG00000005607,	RALGPS1	22.5	0	-5.922619625	0.021245361
circRNA_003620	15:117863548|117913489	n/a	_	60.25	4.25	-3.560495385	0.021333511
circRNA_011568	2:17189969|17190503	ENSSSCG00000013256,	ARHGAP1	23.5	0	-5.915187247	0.021429719
circRNA_005396	X:143162695|143167577	ENSSSCG00000012818,	F8	0	17.75	5.912842266	0.021512408
circRNA_010419	14:8637132|8696994	n/a	_	20.75	0	-5.910048178	0.021557988
circRNA_005290	JH118724.1:73289|92294	n/a	_	0	22	5.908679595	0.021616675
circRNA_004217	3:135341139|135366829	ENSSSCG00000008642,	ASAP2	0	22	5.908679595	0.021616675
circRNA_005110	9:44292300|44293899	ENSSSCG00000029538,	PPP2R1B	0	24	5.907803251	0.021638681
circRNA_002406	X:3659514|3683935	n/a	_	0	17.75	5.904968298	0.021709999
circRNA_001958	7:33242142|33263635	ENSSSCG00000001498,	BEND6	0	13.5	5.900502448	0.021822751
circRNA_006956	13:1187021|1197644	ENSSSCG00000011183,	-	12.25	0	-5.759051441	0.021874328
circRNA_010825	8:20657917|20681770	ENSSSCG00000008761,	TBC1D19	19.25	0	-5.89329038	0.021980797
circRNA_010503	16:31977938|31992995	ENSSSCG00000016882,	PARP8	19.25	0	-5.89329038	0.021980797
circRNA_002275	9:102309677|102554986	n/a	_	3.5	55.75	3.696537817	0.022051227
circRNA_002499	1:56083815|56106529	ENSSSCG00000004272,	LMBRD1	0	22	5.888295467	0.022133488
circRNA_003395	14:81282853|81361983	ENSSSCG00000010289,	MICU1	0	24	5.887407643	0.022156234
circRNA_004877	8:9011621|9028319	n/a	_	0	24	5.887407643	0.022156234
circRNA_004755	7:37462945|37463824	ENSSSCG00000001569,	C6orf89	0	17.75	5.884832915	0.022222312
circRNA_004298	4:74151645|74189892	ENSSSCG00000023518,	-	0	17.75	5.884832915	0.022222312
circRNA_004262	4:42104548|42114243	ENSSSCG00000006084,	MTDH	0	17.75	5.884832915	0.022222312
circRNA_003742	17:5885678|5937612	ENSSSCG00000006989,	-	0	17.75	5.884832915	0.022222312
circRNA_006693	1:143396359|143426509	ENSSSCG00000004718,	TTBK2	22.25	0	-5.880467186	0.022309074
circRNA_001560	4:36253818|36269370	n/a	_	0	13.5	5.875338458	0.022467427
circRNA_006971	13:20785157|20808581	ENSSSCG00000011235,	UBP1	21.5	0	-5.873728802	0.022483241
circRNA_007776	5:91482491|91496070	ENSSSCG00000028182,	-	21.75	0	-5.872323942	0.022519697
circRNA_012071	9:78724734|78745378	ENSSSCG00000015310,	AKAP9	21	0	-5.866554615	0.022669936
circRNA_004372	4:132005794|132023793	ENSSSCG00000006878,	DPYD	0	24	5.866455752	0.022698823

circRNA_010797	7:65666367|65709436	ENSSSCG00000001934,	SEC23A	19	0	-5.863345657	0.022753869
circRNA_006269	6:73284406|73304281	ENSSSCG00000003512,	-	0	22	5.864275565	0.022755925
circRNA_002353	GL894772.1:18966|23591	ENSSSCG00000021427,	DCTN4	0	13.5	5.860892188	0.022844782
circRNA_001635	4:116017070|116017460	ENSSSCG00000006752,	CSDE1	0	13.5	5.860892188	0.022844782
circRNA_007606	3:121950526|121958538	ENSSSCG00000026283,	UBXN2A	23	0	-5.859236497	0.022861733
circRNA_004677	6:143048817|143059562	ENSSSCG00000003833,	DAB1	0	21.75	5.859465735	0.022882333
circRNA_006833	10:48227051|48227265	ENSSSCG00000011033,	VIM	22.5	0	-5.855704856	0.022954783
circRNA_011203	13:28113282|28176864	n/a	_	20.5	0	-5.854864251	0.022976978
circRNA_003049	12:54662564|54665529	n/a	_	0	17.75	5.855767941	0.02297992
circRNA_007727	5:17065100|17094072	ENSSSCG00000029196,	DIP2B	22.75	0	-5.854080999	0.022997676
circRNA_006761	1:244821954|244834415	ENSSSCG00000005227,	-	22.5	0	-5.852304873	0.023044668
circRNA_004821	7:91969534|91977940	ENSSSCG00000002266,	CHD2	0	15.5	5.85117732	0.02310156
circRNA_005920	17:36500876|36516369	ENSSSCG00000007153,	ATRN	0	19.75	5.843612674	0.023303196
circRNA_005836	15:55345812|55352780	ENSSSCG00000015820,	WHSC1L1	0	19.75	5.843612674	0.023303196
circRNA_006688	1:137635283|137638195	ENSSSCG00000004661,	MYEF2	18.5	0	-5.84215186	0.023314859
circRNA_007856	6:112319400|112329035	ENSSSCG00000027700,	RPRD1A	18.5	0	-5.84215186	0.023314859
circRNA_007004	13:73190426|73192256	ENSSSCG00000011553,	-	18.5	0	-5.84215186	0.023314859
circRNA_002092	8:69668235|69682886	ENSSSCG00000008921,	-	0	15.75	5.842494992	0.023333114
circRNA_010834	8:57989763|57990564	n/a	_	18.25	0	-5.839958506	0.02337358
circRNA_010238	1:229173474|229246387	ENSSSCG00000005178,	CNTLN	18.25	0	-5.839958506	0.02337358
circRNA_000111	1:128682226|128695939	n/a	_	0	15.5	5.839912626	0.023402363
circRNA_000391	11:16104484|16108078	ENSSSCG00000009379,	NEK3	0	15.5	5.839912626	0.023402363
circRNA_002237	9:56547706|56553048	ENSSSCG00000015175,	VWA5A	0	15.5	5.839912626	0.023402363
circRNA_001973	7:57934610|57969569	ENSSSCG00000001810,	PDE8A	0	15.5	5.839912626	0.023402363
circRNA_000021	1:17240890|17261849	ENSSSCG00000004089,	RMND1	0	15.5	5.839912626	0.023402363
circRNA_001706	5:38350856|38351426	ENSSSCG00000000509,	-	0	15.5	5.839912626	0.023402363
circRNA_007296	15:157089364|157096463	ENSSSCG00000016395,	RIF1	19	0	-5.838699443	0.023407344
circRNA_004033	2:146504494|146516158	ENSSSCG00000014339,	CTNNA1	0	19.75	5.838627186	0.023436899
circRNA_011996	8:33447628|33495808	ENSSSCG00000024496,	NSUN7	21.25	0	-5.836962133	0.023454002
circRNA_010992	1:132344010|132347527	ENSSSCG00000004620,	MYO5A	21.25	0	-5.836962133	0.023454002
circRNA_010989	1:125384211|125517917	n/a	_	21.25	0	-5.836962133	0.023454002
circRNA_002158	8:143239037|143275381	ENSSSCG00000009230,	WDFY3	0	13.5	5.832752839	0.023595273
circRNA_001288	2:86260574|86285313	ENSSSCG00000014083,	ANKDD1B	0	13.5	5.832752839	0.023595273
circRNA_010350	13:8094772|8096149	ENSSSCG00000011207,	SGO1	18.5	0	-5.831174197	0.023610012
circRNA_010278	10:35240929|35244709	n/a	_	18.5	0	-5.831174197	0.023610012
circRNA_010632	3:103476557|103560207	n/a	_	18.5	0	-5.831174197	0.023610012
circRNA_010842	8:82828282|83064254	n/a	_	18.5	0	-5.831174197	0.023610012
circRNA_010415	13:206274720|206284694	ENSSSCG00000012035,	SYNJ1	18.5	0	-5.831174197	0.023610012
circRNA_010826	8:34753681|34757340	ENSSSCG00000008801,	SLC30A9	18.5	0	-5.831174197	0.023610012
circRNA_010926	X:8558016|8581627	ENSSSCG00000012112,	ARHGAP6	18.5	0	-5.831174197	0.023610012
circRNA_010942	X:97827755|97835641	ENSSSCG00000012530,	TCEAL2	18.5	0	-5.831174197	0.023610012
circRNA_010659	4:39870935|39888370	ENSSSCG00000006069,	RGS22	18.5	0	-5.831174197	0.023610012
circRNA_001223	2:16545939|16546678	ENSSSCG00000013241,	NR1H3	221.25	387	1.000821346	0.023642273
circRNA_007149	14:77561976|77588230	ENSSSCG00000010239,	RUFY2	19.25	0	-5.826381959	0.023739847
circRNA_011013	1:143396359|143449923	ENSSSCG00000004718,	TTBK2	20.5	0	-5.822758564	0.023838417
circRNA_010889	9:78740877|78749748	ENSSSCG00000015310,	AKAP9	18.75	0	-5.822290118	0.023851185
circRNA_010813	7:124821065|124821420	ENSSSCG00000002502,	ATG2B	18.5	0	-5.821981539	0.0238596
circRNA_004984	8:103659931|103712082	n/a	_	0	15.5	5.820754985	0.023921556
circRNA_003602	15:111918691|111930245	ENSSSCG00000016073,	-	0	15.5	5.820754985	0.023921556
circRNA_004812	7:86745779|86748861	n/a	_	0	19.75	5.816253871	0.024044947
circRNA_002556	1:116900620|116917868	ENSSSCG00000004538,	WDR7	0	19.75	5.816253871	0.024044947
circRNA_003721	16:51821929|51829999	ENSSSCG00000016968,	BDP1	0	19.75	5.816253871	0.024044947

circRNA_002581	1:132965230|132974980	ENSSSCG00000004626,	TMOD3	0	21.75	5.815328013	0.024070394
circRNA_006657	1:101247404|101262436	ENSSSCG00000028780,	-	19.5	0	-5.813866358	0.024081786
circRNA_010929	X:14612680|14670417	ENSSSCG00000012150,	REPS2	19	0	-5.813304144	0.024097243
circRNA_010451	14:116594582|116621990	ENSSSCG00000010494,	SORBS1	19	0	-5.813304144	0.024097243
circRNA_010367	13:42372497|42378144	ENSSSCG00000024201,	FAM208A	19	0	-5.813304144	0.024097243
circRNA_010598	2:142541102|142544386	ENSSSCG00000014304,	SEC24A	19	0	-5.813304144	0.024097243
circRNA_011584	2:45045796|45051984	ENSSSCG00000025406,	PIK3C2A	20	0	-5.810364736	0.024178196
circRNA_005861	15:112461241|112463699	ENSSSCG00000016075,	SF3B1	0	15.5	5.808965054	0.024245899
circRNA_002140	8:124794853|124953169	ENSSSCG00000022365,	-	0	11.5	5.807682004	0.02428142
circRNA_000956	15:85340631|85364566	ENSSSCG00000015939,	-	0	11.5	5.807682004	0.02428142
circRNA_002341	GL894053.2:50106|54948	ENSSSCG00000017083,	SPARC	0	11.5	5.807682004	0.02428142
circRNA_010279	10:35254557|35289091	ENSSSCG00000026087,	GKAP1	17.25	0	-5.803401507	0.024370886
circRNA_010528	17:43615013|43623946	ENSSSCG00000007289,	TRPC4AP	17.25	0	-5.803401507	0.024370886
circRNA_002223	9:44284359|44292473	ENSSSCG00000029538,	PPP2R1B	0	17.5	5.79609668	0.02460415
circRNA_004942	8:71458415|71459779	ENSSSCG00000025514,	RUFY3	0	19.75	5.793213673	0.024685022
circRNA_004767	7:57234885|57280795	n/a	_	0	21.75	5.792277147	0.024711341
circRNA_004830	7:98966300|99004586	n/a	_	0	15.5	5.789091712	0.024801039
circRNA_005028	8:144641851|144646781	ENSSSCG00000009242,	LIN54	0	15.5	5.789091712	0.024801039
circRNA_005086	9:29210583|29288870	n/a	_	0	15.5	5.789091712	0.024801039
circRNA_002990	12:27366725|27408291	n/a	_	0	15.5	5.789091712	0.024801039
circRNA_007320	16:47180663|47186013	ENSSSCG00000016943,	ADAMTS6	19	0	-5.785154184	0.024882003
circRNA_011453	15:146501208|146535869	ENSSSCG00000022177,	-	19	0	-5.784946463	0.024887873
circRNA_011183	12:49750004|49751815	ENSSSCG00000017817,	PRPF8	19	0	-5.784946463	0.024887873
circRNA_011767	4:45157523|45168955	ENSSSCG00000006096,	INTS8	19	0	-5.784946463	0.024887873
circRNA_006879	11:23622906|23650191	ENSSSCG00000009426,	CCDC122	20.25	0	-5.774501108	0.025184566
circRNA_007368	17:57066927|57077079	ENSSSCG00000007460,	ARFGEF2	20.5	0	-5.772701887	0.025235972
circRNA_006712	1:161699776|161765610	ENSSSCG00000004857,	-	20.5	0	-5.772701887	0.025235972
circRNA_008269	X:109068407|109093306	ENSSSCG00000012595,	LRCH2	20.25	0	-5.770718228	0.02529275
circRNA_007370	17:57295480|57299121	ENSSSCG00000029603,	-	20.25	0	-5.770718228	0.02529275
circRNA_000210	1:229233921|229246387	ENSSSCG00000005178,	CNTLN	290.5	102.75	-1.328641943	0.025335104
circRNA_007122	14:48100016|48124333	ENSSSCG00000009967,	PITPNB	20.5	0	-5.768910515	0.025344586
circRNA_006903	12:4631315|4637765	ENSSSCG00000029343,	MFSD11	20	0	-5.768729083	0.025349793
circRNA_005720	13:142562710|142629113	ENSSSCG00000011833,	DLG1	0	17.5	5.766907898	0.025433369
circRNA_005885	16:23393877|23405864	ENSSSCG00000016842,	NIPBL	0	17.5	5.766907898	0.025433369
circRNA_001638	4:117897403|117957301	ENSSSCG00000006779,	ST7L	0	13.5	5.765405284	0.025476688
circRNA_001545	4:15260718|15262454	ENSSSCG00000005970,	SQLE	0	13.5	5.765405284	0.025476688
circRNA_003790	17:52395572|52399297	ENSSSCG00000007375,	SERINC3	0	19.5	5.760491192	0.025618791
circRNA_005251	GL894597.1:48506|49753	n/a	_	0	19.5	5.760491192	0.025618791
circRNA_004911	8:40451377|40459063	ENSSSCG00000008826,	-	0	19.5	5.760491192	0.025618791
circRNA_002474	1:18661830|18663491	ENSSSCG00000004104,	-	0	19.5	5.760491192	0.025618791
circRNA_004740	7:31326169|31333945	ENSSSCG00000001486,	LRRC1	0	15.5	5.756087997	0.025746687
circRNA_004885	8:17042628|17067041	n/a	_	0	15.5	5.756087997	0.025746687
circRNA_007119	14:43482274|43487878	ENSSSCG00000009924,	-	19.75	0	-5.752358388	0.025823398
circRNA_005842	15:67009488|67021943	ENSSSCG00000015866,	FMNL2	0	15.5	5.752475993	0.025852004
circRNA_005471	1:181055349|181066687	ENSSSCG00000004934,	DPP8	0	15.5	5.752475993	0.025852004
circRNA_006547	JH118963.1:42907|52126	ENSSSCG00000025611,	-	0	15.5	5.752475993	0.025852004
circRNA_006355	7:91976025|91977940	ENSSSCG00000002266,	CHD2	0	15.5	5.752475993	0.025852004
circRNA_011890	6:85446257|85451841	ENSSSCG00000024887,	-	19	0	-5.749696477	0.025901108
circRNA_011871	6:47675862|47791083	n/a	_	19	0	-5.749696477	0.025901108
circRNA_011457	15:152395889|152396274	ENSSSCG00000016340,	ASB1	19	0	-5.749696477	0.025901108
circRNA_010890	9:79212011|79220647	ENSSSCG00000015317,	FAM133B	16.75	0	-5.746501876	0.025994626
circRNA_007697	4:129642123|129650114	ENSSSCG00000006870,	-	16.5	0	-5.745583323	0.026021573

circRNA_002085	8:43586936|43588846	ENSSSCG00000008842,	KIT	0	11.25	5.744384756	0.026089244
circRNA_002331	GL893171.1:21567|22757	n/a	_	0	11.25	5.744384756	0.026089244
circRNA_001544	4:10938078|11117660	n/a	_	0	11.25	5.744384756	0.026089244
circRNA_010665	4:45690143|45704929	n/a	_	16.25	0	-5.74308422	0.026094992
circRNA_010249	1:270602784|270655882	ENSSSCG00000005391,	-	16.25	0	-5.74308422	0.026094992
circRNA_010394	13:131006932|131033279	n/a	_	16.25	0	-5.74308422	0.026094992
circRNA_010537	18:6267079|6269124	ENSSSCG00000016438,	NUB1	16.25	0	-5.74308422	0.026094992
circRNA_007249	15:73552151|73557223	ENSSSCG00000015882,	BAZ2B	17	0	-5.741688148	0.026136084
circRNA_006887	11:26301535|26305046	ENSSSCG00000009435,	NAA16	17	0	-5.741688148	0.026136084
circRNA_001999	7:73120980|73123784	ENSSSCG00000001970,	HEATR5A	0	13.25	5.737783251	0.026284162
circRNA_002056	8:4258670|4260865	ENSSSCG00000008721,	ABLIM2	0	13.25	5.737783251	0.026284162
circRNA_000298	10:24486122|24493093	ENSSSCG00000010894,	TP53BP2	0	13.25	5.737783251	0.026284162
circRNA_011870	6:47675856|47791083	n/a	_	18.5	0	-5.736036253	0.026303001
circRNA_012078	9:113153990|113161978	ENSSSCG00000025166,	-	18.5	0	-5.736036253	0.026303001
circRNA_002998	12:36535677|36549647	ENSSSCG00000023727,	TRIM37	0	17.5	5.736666969	0.026317242
circRNA_004939	8:64483430|64514152	n/a	_	0	19.5	5.735706687	0.026345728
circRNA_007152	14:77678428|77699105	n/a	_	18.75	0	-5.73436895	0.026352412
circRNA_011312	14:27785622|27797023	ENSSSCG00000025820,	SLC15A4	18.25	0	-5.733729867	0.026371372
circRNA_011932	7:25228359|25229283	ENSSSCG00000001242,	GABBR1	18.25	0	-5.733729867	0.026371372
circRNA_010672	4:73714673|73721931	ENSSSCG00000006202,	-	16.5	0	-5.733120191	0.026389471
circRNA_010215	1:169133734|169146593	n/a	_	16.5	0	-5.733120191	0.026389471
circRNA_010721	5:87303583|87321627	ENSSSCG00000000873,	ANO4	16.5	0	-5.733120191	0.026389471
circRNA_010836	8:58689278|58698211	ENSSSCG00000008909,	-	16.5	0	-5.733120191	0.026389471
circRNA_000941	15:73900098|73912653	ENSSSCG00000015883,	7-Mar	0	11.25	5.729410359	0.026533146
circRNA_006663	1:103173037|103183452	ENSSSCG00000004485,	CD109	17.25	0	-5.727705439	0.02655067
circRNA_006957	13:1433020|1440862	ENSSSCG00000029094,	-	17.25	0	-5.727705439	0.02655067
circRNA_010348	12:63565756|63575963	ENSSSCG00000018057,	SOCS7	15.5	0	-5.72591921	0.026604028
circRNA_007866	6:127538813|127543923	ENSSSCG00000003776,	ACADM	18.25	0	-5.724468195	0.026647439
circRNA_007017	13:90496250|90509814	ENSSSCG00000011676,	-	18.25	0	-5.724468195	0.026647439
circRNA_007690	4:117897403|117913079	ENSSSCG00000006779,	ST7L	18.25	0	-5.724468195	0.026647439
circRNA_007566	3:81720523|81732824	ENSSSCG00000027509,	-	18.25	0	-5.724468195	0.026647439
circRNA_010856	8:143175337|143275381	ENSSSCG00000009230,	WDFY3	16.75	0	-5.723029393	0.026690544
circRNA_011675	3:42032747|42053774	ENSSSCG00000022963,	-	17.75	0	-5.719774724	0.026788266
circRNA_011037	1:208292449|208295366	ENSSSCG00000005073,	ARID4A	17.75	0	-5.719774724	0.026788266
circRNA_005161	9:120362998|120369612	ENSSSCG00000028840,	EZH2	0	17.25	5.70426422	0.026883765
circRNA_002841	10:58751706|58912493	ENSSSCG00000011085,	MLLT10	0	17.25	5.70426422	0.026883765
circRNA_004613	6:92090650|92102784	ENSSSCG00000003682,	ANKRD12	0	17.25	5.70426422	0.026883765
circRNA_003642	15:133380261|133382701	ENSSSCG00000016185,	ARPC2	0	17.25	5.70426422	0.026883765
circRNA_004909	8:39666147|39761604	ENSSSCG00000008813,	CORIN	0	17.25	5.70426422	0.026883765
circRNA_003434	14:120292442|120293042	ENSSSCG00000010540,	ENTPD7	0	17.25	5.70426422	0.026883765
circRNA_005239	GL894022.1:9043|30826	ENSSSCG00000029153,	-	0	17.25	5.70426422	0.026883765
circRNA_003331	14:32154185|32181866	ENSSSCG00000009793,	CLIP1	0	17.25	5.70426422	0.026883765
circRNA_003207	13:138907625|138960618	ENSSSCG00000029252,	FGF12	0	13.25	5.715950418	0.026937566
circRNA_006896	11:56283465|56299174	ENSSSCG00000009480,	NDFIP2	17.75	0	-5.714434898	0.026949248
circRNA_007248	15:73552151|73552402	ENSSSCG00000015882,	BAZ2B	17.75	0	-5.714434898	0.026949248
circRNA_008219	GL896192.1:22918|26125	ENSSSCG00000028008,	VAMP3	17.5	0	-5.713470295	0.026978414
circRNA_001574	4:45282363|45291466	ENSSSCG00000006098,	DPY19L4	0	15.25	5.714055296	0.026994922
circRNA_000837	14:112935251|112953354	ENSSSCG00000010465,	TNKS2	0	15.25	5.714055296	0.026994922
circRNA_001495	3:113380479|113430806	ENSSSCG00000008510,	LTBP1	0	15.25	5.714055296	0.026994922
circRNA_000461	12:18731949|18742385	ENSSSCG00000017346,	EFTUD2	0	15.25	5.714055296	0.026994922
circRNA_010484	15:93751641|93756633	n/a	_	17	0	-5.712808807	0.026998431
circRNA_004900	8:32728074|32737837	ENSSSCG00000023112,	PDS5A	0	17.5	5.71107883	0.027085212

circRNA_002914	11:32036344|32036709	ENSSSCG00000009446,	PCDH17	0	17.5	5.71107883	0.027085212
circRNA_003754	17:19828222|19841369	ENSSSCG00000007056,	PLCB1	0	17.5	5.71107883	0.027085212
circRNA_004153	3:102653727|102663182	ENSSSCG00000008452,	LRPPRC	0	17.5	5.71107883	0.027085212
circRNA_004425	5:36458959|36460887	ENSSSCG00000000496,	CCT2	0	17.5	5.71107883	0.027085212
circRNA_003998	2:119219842|119242988	ENSSSCG00000014195,	MAN2A1	0	19.5	5.710109974	0.027114657
circRNA_006507	9:129689965|129732908	ENSSSCG00000015509,	-	0	17.5	5.707235748	0.027202166
circRNA_005797	14:104690448|104710775	ENSSSCG00000010426,	PCDH15	0	17.5	5.707235748	0.027202166
circRNA_011555	18:49340344|49341282	ENSSSCG00000016692,	-	17.25	0	-5.705557413	0.027218681
circRNA_010745	6:69689581|69690621	ENSSSCG00000024794,	FBXO42	17.25	0	-5.702455312	0.027313363
circRNA_010227	1:202793118|202807301	ENSSSCG00000005041,	FERMT2	15.25	0	-5.701419417	0.027345043
circRNA_010318	11:75001213|75016218	ENSSSCG00000022112,	DOCK9	15.25	0	-5.701419417	0.027345043
circRNA_004247	4:36253818|36254028	n/a	_	0	17.5	5.698891445	0.027357589
circRNA_003148	13:81424354|81437313	ENSSSCG00000011632,	-	0	17.5	5.698891445	0.027357589
circRNA_011738	3:135114875|135117428	ENSSSCG00000008641,	ADAM17	16.75	0	-5.691068156	0.027663298
circRNA_005666	13:35739632|35749546	ENSSSCG00000011394,	RBM6	0	15.5	5.69157626	0.027683125
circRNA_005697	13:90919603|90950445	ENSSSCG00000011678,	XRN1	0	15.5	5.69157626	0.027683125
circRNA_005773	14:31408084|31409493	ENSSSCG00000009779,	MPHOSPH9	0	15.5	5.69157626	0.027683125
circRNA_006034	2:121986386|122010500	ENSSSCG00000014209,	EPB41L4A	0	15.5	5.69157626	0.027683125
circRNA_010622	3:72585580|72603949	n/a	_	15.25	0	-5.688449558	0.0277443
circRNA_007691	4:121576595|121582456	ENSSSCG00000006841,	-	18	0	-5.679315718	0.028028401
circRNA_007519	2:146476770|146516158	ENSSSCG00000014339,	CTNNA1	18	0	-5.679315718	0.028028401
circRNA_007928	7:73073215|73086240	ENSSSCG00000001970,	HEATR5A	18	0	-5.679315718	0.028028401
circRNA_003348	14:49392310|49397454	ENSSSCG00000009974,	EWSR1	0	13.25	5.679584687	0.028056244
circRNA_004384	4:137324329|137358737	n/a	_	0	13.25	5.679584687	0.028056244
circRNA_000991	15:115465676|115466348	ENSSSCG00000029606,	AOX1	0	11.25	5.679574604	0.028056559
circRNA_000427	11:70156594|70190434	ENSSSCG00000026996,	-	0	11.25	5.679574604	0.028056559
circRNA_000314	10:33646093|33649475	ENSSSCG00000010957,	-	0	11.25	5.679574604	0.028056559
circRNA_000407	11:25529781|25564920	ENSSSCG00000009431,	DGKH	0	11.25	5.679574604	0.028056559
circRNA_000104	1:126578348|126579567	n/a	_	0	11.25	5.679574604	0.028056559
circRNA_006678	1:125143608|125147013	ENSSSCG00000004592,	-	18.25	0	-5.677290098	0.028091736
circRNA_007782	5:106793019|106794736	n/a	_	18.25	0	-5.677290098	0.028091736
circRNA_007851	6:101697609|101938585	n/a	_	18	0	-5.675033663	0.02816243
circRNA_007890	6:150870199|150895226	ENSSSCG00000003881,	SPATA6	18	0	-5.675033663	0.02816243
circRNA_006608	1:17075533|17091938	ENSSSCG00000004087,	CCDC170	18	0	-5.675033663	0.02816243
circRNA_007872	6:136802506|136808752	ENSSSCG00000003811,	ROR1	18	0	-5.675033663	0.02816243
circRNA_007228	15:51629686|51636427	ENSSSCG00000015777,	TRAPPC11	17.75	0	-5.672770142	0.028233495
circRNA_007091	14:6687380|6693475	ENSSSCG00000009611,	XPO7	18	0	-5.670727974	0.02829774
circRNA_006052	3:3687221|3695971	ENSSSCG00000007574,	SDK1	0	13.25	5.66537519	0.028503833
circRNA_005903	16:56200707|56212695	ENSSSCG00000027378,	SH3PXD2B	0	13.25	5.66537519	0.028503833
circRNA_006573	X:70426183|70498760	ENSSSCG00000012434,	-	0	13.25	5.66537519	0.028503833
circRNA_010923	GL896447.1:54004|54475	ENSSSCG00000025653,	THOC1	15	0	-5.662714805	0.028551014
circRNA_007283	15:122942696|122959141	ENSSSCG00000016147,	PIKFYVE	17.5	0	-5.658588174	0.028682186
circRNA_002477	1:23236777|23243695	n/a	_	0	13.25	5.641462848	0.029270576
circRNA_003749	17:14861638|14933267	n/a	_	0	13.25	5.641462848	0.029270576
circRNA_002775	10:17146793|17170362	ENSSSCG00000010862,	CDC42BPA	0	15.25	5.630009642	0.029336394
circRNA_004061	3:17185508|17191996	ENSSSCG00000007746,	-	0	15.25	5.630009642	0.029336394
circRNA_003162	13:86954020|86954463	ENSSSCG00000026746,	CEP70	0	15.25	5.630009642	0.029336394
circRNA_004839	7:103045223|103061148	n/a	_	0	15.25	5.630009642	0.029336394
circRNA_004276	4:55364472|55390366	ENSSSCG00000006137,	WWP1	0	15.25	5.630009642	0.029336394
circRNA_003104	13:37111091|37113036	ENSSSCG00000011425,	-	0	15.25	5.630009642	0.029336394
circRNA_002957	12:14242194|14284284	ENSSSCG00000017274,	PITPNC1	0	15.25	5.630009642	0.029336394
circRNA_004600	6:88187287|88196465	ENSSSCG00000003654,	MACF1	0	15.25	5.630009642	0.029336394

circRNA_002986	12:23727465|23750252	ENSSSCG00000017513,	NPEPPS	0	15.25	5.630009642	0.029336394
circRNA_003921	2:61950218|61953288	ENSSSCG00000025176,	-	0	15.25	5.630009642	0.029336394
circRNA_007623	4:15707963|16001218	n/a	_	16.5	0	-5.63819505	0.029337856
circRNA_007277	15:119473764|119477870	n/a	_	16.5	0	-5.63819505	0.029337856
circRNA_010752	6:88641987|88645235	ENSSSCG00000003664,	-	14.75	0	-5.63609714	0.029406013
circRNA_001975	7:57954480|57969573	ENSSSCG00000001810,	PDE8A	58	151.75	1.495249997	0.029410865
circRNA_007596	3:109890287|109910797	ENSSSCG00000008499,	STRN	14.5	0	-5.635007604	0.029441463
circRNA_008247	X:40497155|40507872	ENSSSCG00000012250,	MED14	14.5	0	-5.635007604	0.029441463
circRNA_007650	4:51266360|51271923	ENSSSCG00000006127,	-	14.5	0	-5.635007604	0.029441463
circRNA_003343	14:42660884|42663883	ENSSSCG00000009896,	BICDL1	0	17.25	5.631578448	0.029492418
circRNA_003964	2:88698666|88719584	ENSSSCG00000014101,	-	0	17.25	5.631578448	0.029492418
circRNA_004597	6:85446257|85450927	ENSSSCG00000024887,	-	0	17.25	5.631578448	0.029492418
circRNA_011116	10:54381760|54384166	ENSSSCG00000030156,	-	16	0	-5.632924135	0.029509351
circRNA_011762	4:40343834|40354146	n/a	_	16	0	-5.632924135	0.029509351
circRNA_011228	13:67530900|67547482	n/a	_	16	0	-5.632924135	0.029509351
circRNA_011279	13:160856562|160860539	ENSSSCG00000011943,	-	16	0	-5.632924135	0.029509351
circRNA_011683	3:56415476|56418177	n/a	_	16	0	-5.632924135	0.029509351
circRNA_010824	8:19208010|19209747	ENSSSCG00000023001,	CCDC149	14.25	0	-5.632109536	0.02953593
circRNA_010273	10:19934535|19935498	n/a	_	14.25	0	-5.632109536	0.02953593
circRNA_010457	14:136012794|136046886	ENSSSCG00000010651,	ABLIM1	14.25	0	-5.632109536	0.02953593
circRNA_010645	3:128289492|128296495	ENSSSCG00000008614,	SMC6	14.25	0	-5.632109536	0.02953593
circRNA_010875	9:44289455|44293899	ENSSSCG00000029538,	PPP2R1B	14.25	0	-5.632109536	0.02953593
circRNA_000281	10:13600749|13605870	ENSSSCG00000010835,	AIDA	0	13.25	5.63290286	0.02954922
circRNA_000128	1:141607417|141612336	ENSSSCG00000024783,	-	0	13.25	5.63290286	0.02954922
circRNA_000448	12:14679564|14695314	ENSSSCG00000017277,	PECAM1	0	13.25	5.63290286	0.02954922
circRNA_007817	6:57230731|57234640	n/a	_	15	0	-5.63054709	0.029586965
circRNA_010524	17:36201364|36202682	ENSSSCG00000007143,	MAVS	14.5	0	-5.62061699	0.029913048
circRNA_010658	4:39870935|39873716	ENSSSCG00000006069,	RGS22	14.5	0	-5.62061699	0.029913048
circRNA_010452	14:117614090|117651080	ENSSSCG00000010508,	TM9SF3	14.5	0	-5.62061699	0.029913048
circRNA_000812	14:78371743|78434807	n/a	_	0	11	5.619862033	0.029977993
circRNA_001709	5:45994543|45998196	ENSSSCG00000026593,	-	0	11	5.619862033	0.029977993
circRNA_001456	3:84809485|84822563	n/a	_	0	11	5.619862033	0.029977993
circRNA_001209	2:3029587|3109853	n/a	_	0	11	5.619862033	0.029977993
circRNA_000418	11:53756939|53758513	ENSSSCG00000009471,	-	0	11	5.619862033	0.029977993
circRNA_003196	13:119202413|119216757	ENSSSCG00000011750,	PLD1	0	15.25	5.619096451	0.030003325
circRNA_003635	15:124628985|124693334	ENSSSCG00000022830,	KANSL1L	0	15.25	5.619096451	0.030003325
circRNA_002745	1:280627976|280656034	ENSSSCG00000005444,	TMEM245	0	15.25	5.619096451	0.030003325
circRNA_002726	1:262203573|262203983	n/a	_	0	15.25	5.619096451	0.030003325
circRNA_003788	17:51655197|51658984	ENSSSCG00000007364,	IFT52	0	15.25	5.619096451	0.030003325
circRNA_011651	2:154284494|154293122	ENSSSCG00000014416,	TCERG1	15.5	0	-5.616976736	0.030033334
circRNA_011444	15:122453532|122461484	ENSSSCG00000024696,	CCNYL1	15.5	0	-5.616976736	0.030033334
circRNA_011652	2:157845326|157846464	ENSSSCG00000014440,	-	15.5	0	-5.616976736	0.030033334
circRNA_011011	1:143361257|143405910	ENSSSCG00000004718,	TTBK2	15.5	0	-5.616976736	0.030033334
circRNA_006974	13:24879745|24890118	ENSSSCG00000028740,	CTDSPL	15.25	0	-5.614403065	0.030118622
circRNA_006383	8:9011621|9025604	n/a	_	0	15.25	5.614718184	0.030148545
circRNA_005940	18:10898787|10902471	ENSSSCG00000016510,	UBN2	0	15.25	5.614718184	0.030148545
circRNA_005529	1:299807814|299813040	ENSSSCG00000005602,	GAPVD1	0	15.25	5.614718184	0.030148545
circRNA_006193	5:13399178|13399786	ENSSSCG00000000167,	RIC8B	0	15.25	5.614718184	0.030148545
circRNA_005830	15:28533728|28551296	ENSSSCG00000015718,	C2orf76	0	15.25	5.614718184	0.030148545
circRNA_005466	1:175580337|175588657	ENSSSCG00000004893,	VPS4B	0	15.25	5.614718184	0.030148545
circRNA_010913	GL893259.1:18126|36041	ENSSSCG00000026158,	RAD18	13.5	0	-5.612211179	0.030191419
circRNA_010344	12:47450863|47452593	ENSSSCG00000017785,	NUFIP2	13.5	0	-5.612211179	0.030191419

circRNA_010251	1:282142302|282144628	ENSSSCG00000005455,	SVEP1	13.5	0	-5.612211179	0.030191419
circRNA_010381	13:90760724|90763531	ENSSSCG00000011677,	GK5	14.75	0	-5.608957793	0.03029974
circRNA_004381	4:136249373|136360665	n/a	_	0	15.25	5.597790641	0.030409074
circRNA_003429	14:117074459|117107550	n/a	_	0	15.25	5.597790641	0.030409074
circRNA_004161	3:106269291|106310785	n/a	_	0	15.25	5.597790641	0.030409074
circRNA_002589	1:135303493|135500052	ENSSSCG00000004646,	-	0	15.25	5.597790641	0.030409074
circRNA_003167	13:90496250|90502795	ENSSSCG00000011676,	-	0	15.25	5.597790641	0.030409074
circRNA_002486	1:28422430|28512553	n/a	_	0	15.25	5.597790641	0.030409074
circRNA_003404	14:92407605|92416252	ENSSSCG00000010351,	CCSER2	0	15.25	5.597790641	0.030409074
circRNA_003031	12:45448728|45449787	ENSSSCG00000017748,	NF1	0	15.25	5.597790641	0.030409074
circRNA_006995	13:44062045|44086522	ENSSSCG00000011474,	PXK	15.25	0	-5.603707803	0.030475225
circRNA_006653	1:96785770|96802706	n/a	_	15.25	0	-5.603707803	0.030475225
circRNA_007859	6:118020841|118048164	ENSSSCG00000003749,	PIK3C3	15.25	0	-5.603707803	0.030475225
circRNA_012164	X:105396055|105407934	ENSSSCG00000012583,	-	15	0	-5.600689119	0.03057651
circRNA_010965	1:44462994|44469456	ENSSSCG00000004238,	HSF2	15	0	-5.600689119	0.03057651
circRNA_010551	18:52077580|52356954	n/a	_	15	0	-5.597128222	0.03069635
circRNA_005999	2:82538960|82565744	ENSSSCG00000022508,	UIMC1	0	13.25	5.596923414	0.030744832
circRNA_006025	2:106257761|106273263	ENSSSCG00000029805,	RHOBTB3	0	13.25	5.596923414	0.030744832
circRNA_006098	3:107382061|107410577	ENSSSCG00000008478,	-	0	13.25	5.596923414	0.030744832
circRNA_006143	4:48150336|48156272	ENSSSCG00000006115,	RUNX1T1	0	13.25	5.596923414	0.030744832
circRNA_006141	4:43558505|43561270	ENSSSCG00000006090,	MTERF3	0	13.25	5.596923414	0.030744832
circRNA_005719	13:142522109|142551946	ENSSSCG00000011833,	DLG1	0	13.25	5.596923414	0.030744832
circRNA_001529	3:130810562|130868878	ENSSSCG00000008621,	-	0	9	5.586321253	0.031104765
circRNA_001584	4:55349876|55364656	ENSSSCG00000006137,	WWP1	0	9	5.586321253	0.031104765
circRNA_001446	3:76334887|76336888	ENSSSCG00000008337,	AAK1	0	9	5.586321253	0.031104765
circRNA_001707	5:38557794|38561578	ENSSSCG00000000512,	TBC1D15	0	9	5.586321253	0.031104765
circRNA_000969	15:93043493|93065848	n/a	_	0	9	5.586321253	0.031104765
circRNA_002149	8:134379373|134409025	ENSSSCG00000009192,	PDLIM5	147	363.25	1.49896741	0.031106321
circRNA_007130	14:53590430|53592330	ENSSSCG00000010081,	MAPK1	15.75	0	-5.57062986	0.031600519
circRNA_007019	13:90686409|90695250	n/a	_	15.75	0	-5.57062986	0.031600519
circRNA_007929	7:73092645|73111840	ENSSSCG00000001970,	HEATR5A	15.75	0	-5.57062986	0.031600519
circRNA_007157	14:85796577|85798382	n/a	_	15.75	0	-5.57062986	0.031600519
circRNA_007715	5:4607780|4611101	ENSSSCG00000000068,	EP300	15.75	0	-5.57062986	0.031600519
circRNA_007571	3:90414201|90417350	ENSSSCG00000008399,	-	15.75	0	-5.57062986	0.031600519
circRNA_008027	8:80764338|80786409	ENSSSCG00000009010,	ARFIP1	15.75	0	-5.57062986	0.031600519
circRNA_003587	15:101683537|101693050	ENSSSCG00000016027,	ITGAV	0	11	5.566859923	0.031656652
circRNA_005151	9:102608469|102614132	ENSSSCG00000015390,	ABCB1	0	11	5.566859923	0.031656652
circRNA_004664	6:132251968|132261114	ENSSSCG00000003792,	LRRC40	0	11	5.566859923	0.031656652
circRNA_005241	GL894261.2:68764|91044	ENSSSCG00000020878,	HNRNPLL	0	11	5.566859923	0.031656652
circRNA_004883	8:15232138|15257460	n/a	_	0	11	5.566859923	0.031656652
circRNA_003496	15:18701372|18730119	ENSSSCG00000015692,	-	0	11	5.566859923	0.031656652
circRNA_002849	10:61727666|61744522	ENSSSCG00000011102,	NRP1	0	11	5.566859923	0.031656652
circRNA_004858	7:125239034|125243823	n/a	_	0	11	5.566859923	0.031656652
circRNA_004947	8:74346358|74363926	ENSSSCG00000008961,	MTHFD2L	0	11	5.566859923	0.031656652
circRNA_010680	4:98128291|98143200	ENSSSCG00000006388,	-	13.25	0	-5.568646811	0.031669068
circRNA_007415	18:59685146|59697010	n/a	_	15.75	0	-5.565703495	0.03177104
circRNA_007308	16:29539650|29620216	n/a	_	15.75	0	-5.565703495	0.03177104
circRNA_006965	13:14230737|14246148	ENSSSCG00000011214,	NGLY1	15.75	0	-5.565703495	0.03177104
circRNA_006866	11:15977687|15993129	ENSSSCG00000009376,	VPS36	15.75	0	-5.565703495	0.03177104
circRNA_007054	13:132982287|132987238	ENSSSCG00000011794,	SENP2	15.75	0	-5.565703495	0.03177104
circRNA_008060	8:140670588|140679241	ENSSSCG00000009223,	NUDT9	15.75	0	-5.565703495	0.03177104
circRNA_007966	7:123615110|123629846	n/a	_	15.75	0	-5.565703495	0.03177104

circRNA_007175	14:116594582|116611789	ENSSSCG00000010494,	SORBS1	15.5	0	-5.56308179	0.0318621
circRNA_008054	8:124317943|124329383	n/a	_	15.5	0	-5.56308179	0.0318621
circRNA_007952	7:102425698|102436732	ENSSSCG00000022178,	-	15.5	0	-5.56308179	0.0318621
circRNA_007338	17:5933495|5979140	n/a	_	15.75	0	-5.560746186	0.031943406
circRNA_007202	14:142735102|142804005	ENSSSCG00000010699,	ATE1	15.75	0	-5.560746186	0.031943406
circRNA_006648	1:92286461|92286929	ENSSSCG00000004441,	DSE	15.75	0	-5.560746186	0.031943406
circRNA_006709	1:161192141|161193203	ENSSSCG00000030470,	-	15.75	0	-5.560746186	0.031943406
circRNA_006924	12:23727465|23732256	ENSSSCG00000017513,	NPEPPS	15.75	0	-5.560746186	0.031943406
circRNA_008100	9:36691555|36736202	n/a	_	15.75	0	-5.560746186	0.031943406
circRNA_007026	13:91070338|91076217	ENSSSCG00000011679,	ATR	15.75	0	-5.560746186	0.031943406
circRNA_006013	2:90927901|90947683	ENSSSCG00000014121,	-	0	13.25	5.547151934	0.032263025
circRNA_005853	15:94283009|94309970	ENSSSCG00000016005,	-	0	13.25	5.547151934	0.032263025
circRNA_006430	8:120817823|120827260	ENSSSCG00000009142,	SEC24B	0	13.25	5.547151934	0.032263025
circRNA_006283	6:90255339|90263205	n/a	_	0	13.25	5.547151934	0.032263025
circRNA_006354	7:91959137|91964590	ENSSSCG00000002266,	CHD2	0	13.25	5.547151934	0.032263025
circRNA_005425	1:72497312|72548400	ENSSSCG00000028811,	-	0	13.25	5.547151934	0.032263025
circRNA_005729	13:148398444|148417703	n/a	_	0	13.25	5.547151934	0.032263025
circRNA_005552	10:37689024|37696115	ENSSSCG00000011000,	DNAJA1	0	13.25	5.547151934	0.032263025
circRNA_006129	4:32690092|32715543	ENSSSCG00000006035,	ANGPT1	0	13.25	5.547151934	0.032263025
circRNA_005972	2:28201552|28213565	ENSSSCG00000013297,	CD44	0	13.25	5.547151934	0.032263025
circRNA_005223	GL892805.1:48659|58930	ENSSSCG00000028563,	HNRNPR	0	15	5.545052375	0.032286336
circRNA_004031	2:145725178|145741087	ENSSSCG00000029125,	FAM13B	0	15	5.545052375	0.032286336
circRNA_002940	11:75110052|75132336	ENSSSCG00000022112,	DOCK9	0	15	5.545052375	0.032286336
circRNA_004081	3:43036166|43046846	ENSSSCG00000024292,	-	0	15	5.545052375	0.032286336
circRNA_000578	13:42377857|42378144	ENSSSCG00000024201,	FAM208A	0	11	5.545594109	0.032395256
circRNA_001158	18:14986350|15002971	n/a	_	0	11	5.545594109	0.032395256
circRNA_002145	8:130481169|130491478	ENSSSCG00000009186,	METAP1	0	11	5.545594109	0.032395256
circRNA_001735	5:70565540|70693708	n/a	_	0	11	5.545594109	0.032395256
circRNA_000824	14:83832559|83851733	ENSSSCG00000010319,	SAMD8	0	11	5.545594109	0.032395256
circRNA_000119	1:134611516|134636559	ENSSSCG00000004636,	TRPM7	0	11	5.545594109	0.032395256
circRNA_006724	1:181057330|181066687	ENSSSCG00000004934,	DPP8	15	0	-5.538923292	0.032711488
circRNA_007123	14:50021607|50034842	ENSSSCG00000009994,	MTMR3	15	0	-5.538923292	0.032711488
circRNA_007488	2:91206107|91217079	ENSSSCG00000014126,	MSH3	15	0	-5.538923292	0.032711488
circRNA_007837	6:83154364|83158468	n/a	_	15	0	-5.538923292	0.032711488
circRNA_007874	6:137387063|137397644	ENSSSCG00000003815,	ALG6	15	0	-5.538923292	0.032711488
circRNA_007582	3:102273355|102275943	ENSSSCG00000008448,	PREPL	15	0	-5.538923292	0.032711488
circRNA_007246	15:73524173|73545942	ENSSSCG00000015882,	BAZ2B	15	0	-5.538923292	0.032711488
circRNA_011443	15:122266857|122273427	n/a	_	14.5	0	-5.53804313	0.032742786
circRNA_011811	5:35948460|35958087	ENSSSCG00000000488,	MDM2	14.5	0	-5.53804313	0.032742786
circRNA_011374	14:131878361|131882008	ENSSSCG00000010625,	SMC3	14.5	0	-5.53804313	0.032742786
circRNA_011640	2:141893166|141895222	ENSSSCG00000014296,	VDAC1	14.5	0	-5.53804313	0.032742786
circRNA_012112	GL893511.2:215624|229217	ENSSSCG00000024716,	-	14.5	0	-5.53804313	0.032742786
circRNA_011201	13:19755935|19774230	ENSSSCG00000011229,	STT3B	14.5	0	-5.53804313	0.032742786
circRNA_007919	7:62884149|62919050	n/a	_	14.25	0	-5.52825094	0.033092676
circRNA_006651	1:94535554|94542426	ENSSSCG00000004460,	IBTK	14.25	0	-5.52825094	0.033092676
circRNA_008087	9:13612179|13616289	ENSSSCG00000014887,	-	14.25	0	-5.52825094	0.033092676
circRNA_007084	13:205982161|205994684	ENSSSCG00000029392,	-	14.25	0	-5.52825094	0.033092676
circRNA_007661	4:71185799|71207535	ENSSSCG00000006196,	SLCO5A1	14.25	0	-5.52825094	0.033092676
circRNA_007747	5:49575425|49587521	ENSSSCG00000000547,	MRPS35	14.25	0	-5.52825094	0.033092676
circRNA_007101	14:21576022|21611312	ENSSSCG00000009714,	NEK1	14.5	0	-5.526105804	0.033169738
circRNA_006954	12:62458832|62523689	ENSSSCG00000018039,	NCOR1	14.5	0	-5.526105804	0.033169738
circRNA_008049	8:112784240|112786887	ENSSSCG00000030024,	-	14.5	0	-5.526105804	0.033169738

circRNA_007035	13:103719305|103732607	ENSSSCG00000025316,	SLC33A1	14.5	0	-5.526105804	0.033169738
circRNA_004840	7:106678476|106692160	ENSSSCG00000002404,	SPTLC2	0	11	5.522648161	0.033211967
circRNA_004329	4:104224057|104235575	ENSSSCG00000006549,	IL6R	0	11	5.522648161	0.033211967
circRNA_004406	5:5192560|5193695	ENSSSCG00000000075,	MKL1	0	11	5.522648161	0.033211967
circRNA_003833	18:18686159|18701330	ENSSSCG00000016549,	MKLN1	0	11	5.522648161	0.033211967
circRNA_004204	3:122008499|122024159	ENSSSCG00000008591,	-	0	11	5.522648161	0.033211967
circRNA_005393	X:138587662|138651247	ENSSSCG00000012730,	AFF2	0	11	5.522648161	0.033211967
circRNA_004200	3:120585899|120586512	n/a	_	0	11	5.522648161	0.033211967
circRNA_003317	14:30058998|30067011	ENSSSCG00000009759,	SCARB1	0	11	5.522648161	0.033211967
circRNA_011795	4:136390669|136404063	ENSSSCG00000006903,	RPAP2	14	0	-5.519981609	0.033390569
circRNA_012033	8:146890664|146931194	ENSSSCG00000009256,	ANTXR2	14	0	-5.519981609	0.033390569
circRNA_011876	6:65200370|65203979	ENSSSCG00000003413,	MTOR	14	0	-5.519981609	0.033390569
circRNA_011448	15:124666386|124720160	ENSSSCG00000022830,	KANSL1L	14	0	-5.519981609	0.033390569
circRNA_011364	14:115931100|116131769	n/a	_	14	0	-5.519981609	0.033390569
circRNA_011519	17:42006762|42018906	ENSSSCG00000027410,	CBFA2T2	14	0	-5.519981609	0.033390569
circRNA_011980	7:125280803|125289007	ENSSSCG00000002506,	VRK1	14	0	-5.519981609	0.033390569
circRNA_010956	1:16870879|16889979	n/a	_	14	0	-5.519981609	0.033390569
circRNA_011381	14:136612215|136857384	ENSSSCG00000010654,	ATRNL1	13.75	0	-5.516896632	0.033502271
circRNA_011354	14:110126707|110130346	ENSSSCG00000010450,	LIPA	13.75	0	-5.516896632	0.033502271
circRNA_012116	GL893873.1:50185|56079	n/a	_	13.75	0	-5.516896632	0.033502271
circRNA_011791	4:121321317|121332168	n/a	_	13.75	0	-5.516896632	0.033502271
circRNA_012054	9:29830958|29840619	ENSSSCG00000014949,	MED17	13.75	0	-5.516896632	0.033502271
circRNA_012014	8:103811005|103820414	ENSSSCG00000009075,	MFSD8	13.75	0	-5.516896632	0.033502271
circRNA_004498	5:93592779|93763194	n/a	_	0	13	5.506115983	0.033730358
circRNA_003025	12:44665244|44665704	ENSSSCG00000017741,	TEFM	0	13	5.506115983	0.033730358
circRNA_004616	6:94773159|94788895	ENSSSCG00000003687,	EPB41L3	0	13	5.506115983	0.033730358
circRNA_004716	7:12247873|12298480	ENSSSCG00000001062,	DTNBP1	0	13	5.506115983	0.033730358
circRNA_004962	8:83064146|83280539	ENSSSCG00000009021,	-	0	13	5.506115983	0.033730358
circRNA_003143	13:79284414|79285728	ENSSSCG00000011612,	RPN1	0	13	5.506115983	0.033730358
circRNA_006222	5:80679719|80685276	n/a	_	0	11	5.50480093	0.033860682
circRNA_006339	7:62835978|62848707	ENSSSCG00000001878,	PTPN9	0	11	5.50480093	0.033860682
circRNA_005725	13:143902275|143903196	ENSSSCG00000011853,	RUBCN	0	11	5.50480093	0.033860682
circRNA_005974	2:39869318|39899493	ENSSSCG00000030498,	-	0	11	5.50480093	0.033860682
circRNA_006118	3:128253444|128273499	ENSSSCG00000008614,	SMC6	0	11	5.50480093	0.033860682
circRNA_006461	9:29699977|29705606	ENSSSCG00000014946,	CEP295	0	11	5.50480093	0.033860682
circRNA_005710	13:126801546|126826040	ENSSSCG00000025738,	-	0	11	5.50480093	0.033860682
circRNA_006127	4:31712187|31714845	ENSSSCG00000006033,	EIF3E	0	11	5.50480093	0.033860682
circRNA_006594	1:2789914|2814551	ENSSSCG00000004018,	AFDN	12.5	0	-5.505883446	0.03390358
circRNA_007286	15:133589366|133595116	ENSSSCG00000016194,	USP37	12.5	0	-5.505883446	0.03390358
circRNA_008236	X:14292770|14302172	ENSSSCG00000012149,	RBBP7	12.5	0	-5.505883446	0.03390358
circRNA_010180	1:60892775|60905657	ENSSSCG00000004293,	SNX14	12.25	0	-5.502441231	0.034029826
circRNA_010868	9:28646434|28712693	ENSSSCG00000014935,	FAT3	12.25	0	-5.502441231	0.034029826
circRNA_010731	6:16298300|16300646	ENSSSCG00000002755,	NFAT5	12.25	0	-5.502441231	0.034029826
circRNA_007047	13:130256872|130257767	ENSSSCG00000025942,	MCCC1	13.5	0	-5.501779293	0.034054148
circRNA_008228	JH118611.1:2203|33429	ENSSSCG00000022081,	-	13.5	0	-5.501779293	0.034054148
circRNA_007597	3:113310620|113444950	ENSSSCG00000008510,	LTBP1	13.5	0	-5.501779293	0.034054148
circRNA_006735	1:201668388|201671933	ENSSSCG00000005027,	FRMD6	13.5	0	-5.501779293	0.034054148
circRNA_006619	1:25360229|25360852	ENSSSCG00000004139,	ADGRG6	13.5	0	-5.501779293	0.034054148
circRNA_006643	1:80241844|80313405	ENSSSCG00000004364,	HACE1	13.5	0	-5.501779293	0.034054148
circRNA_006801	10:177153|178895	ENSSSCG00000010799,	COG7	13	0	-5.500674784	0.034094764
circRNA_007990	8:31012141|31024807	ENSSSCG00000008774,	-	13	0	-5.500674784	0.034094764
circRNA_007682	4:107957216|107966186	ENSSSCG00000030291,	RPRD2	13	0	-5.500674784	0.034094764

circRNA_011204	13:28131575|28146602	n/a	_	13.25	0	-5.498322057	0.034181414
circRNA_011293	13:209969995|209973552	ENSSSCG00000012054,	DOPEY2	13.25	0	-5.498322057	0.034181414
circRNA_011701	3:80412430|80424109	ENSSSCG00000008357,	SPRED2	13.25	0	-5.498322057	0.034181414
circRNA_011702	3:84698909|84710843	ENSSSCG00000008386,	-	13.25	0	-5.498322057	0.034181414
circRNA_011940	7:45327224|45329571	ENSSSCG00000001708,	CDC5L	13.25	0	-5.498322057	0.034181414
circRNA_011669	3:29719350|29720141	n/a	_	13.25	0	-5.498322057	0.034181414
circRNA_011409	15:71690220|71745408	ENSSSCG00000015874,	ACVR1	13.25	0	-5.498322057	0.034181414
circRNA_012171	X:126562222|126569599	ENSSSCG00000012686,	MOSPD1	13.25	0	-5.498322057	0.034181414
circRNA_000206	1:227181487|227210811	ENSSSCG00000005170,	DENND4C	23.25	86	2.233958459	0.034464146
circRNA_010361	13:34856835|34859202	ENSSSCG00000011367,	ARIH2	12.5	0	-5.488891729	0.034530568
circRNA_010500	15:146627679|146810404	n/a	_	12.75	0	-5.483030095	0.034683226
circRNA_004482	5:83062597|83067876	ENSSSCG00000000838,	SLC41A2	0	13	5.476310471	0.034782541
circRNA_002570	1:127622340|127628063	ENSSSCG00000026960,	ZNF280D	0	13	5.476310471	0.034782541
circRNA_003954	2:86260574|86291987	ENSSSCG00000014083,	ANKDD1B	0	13	5.476310471	0.034782541
circRNA_003461	14:136289720|136311233	ENSSSCG00000010652,	FAM160B1	0	13	5.476310471	0.034782541
circRNA_003298	14:11428131|11443856	ENSSSCG00000009657,	PPP2R2A	0	13	5.476310471	0.034782541
circRNA_003994	2:118941644|118961796	ENSSSCG00000022845,	-	0	13	5.476310471	0.034782541
circRNA_004605	6:88792955|88849787	n/a	_	0	13	5.476310471	0.034782541
circRNA_003204	13:131203137|131237882	ENSSSCG00000011779,	PARL	0	13	5.476310471	0.034782541
circRNA_003456	14:135311335|135314257	ENSSSCG00000010645,	-	0	13	5.476310471	0.034782541
circRNA_005117	9:50825051|50833295	ENSSSCG00000030053,	ARCN1	0	13	5.476310471	0.034782541
circRNA_001174	18:25506902|25510292	ENSSSCG00000016608,	IQUB	0	8.75	5.482550123	0.034800455
circRNA_007802	6:32195144|32242475	ENSSSCG00000002844,	PHKB	13.25	0	-5.481617089	0.034801922
circRNA_010903	9:133538054|133549074	ENSSSCG00000030296,	-	11.5	0	-5.479106193	0.034893627
circRNA_007439	2:48072495|48078432	ENSSSCG00000021317,	-	13.5	0	-5.460700572	0.035451395
circRNA_007096	14:10459275|10463058	ENSSSCG00000009650,	DOCK5	13.5	0	-5.460700572	0.035451395
circRNA_006858	11:472454|502116	ENSSSCG00000026744,	ZDHHC20	13.5	0	-5.460700572	0.035451395
circRNA_008057	8:133985141|134040905	ENSSSCG00000029621,	BMPR1B	13.5	0	-5.460700572	0.035451395
circRNA_007414	18:59639958|59688893	ENSSSCG00000016769,	CDK13	13.5	0	-5.460700572	0.035451395
circRNA_006859	11:4045654|4070420	ENSSSCG00000009302,	USP12	13.5	0	-5.460700572	0.035451395
circRNA_007578	3:92579061|92608458	ENSSSCG00000008415,	-	13.5	0	-5.460700572	0.035451395
circRNA_006826	10:35382114|35384249	ENSSSCG00000021005,	UBQLN1	13.5	0	-5.460700572	0.035451395
circRNA_007944	7:97739664|97747301	ENSSSCG00000002296,	RDH11	13.5	0	-5.460700572	0.035451395
circRNA_007820	6:62370730|62371821	ENSSSCG00000003384,	DNAJC11	13.5	0	-5.460700572	0.035451395
circRNA_006690	1:143245293|143262310	ENSSSCG00000004717,	UBR1	13.5	0	-5.460700572	0.035451395
circRNA_007901	7:24168721|24169843	n/a	_	13.5	0	-5.460700572	0.035451395
circRNA_007750	5:50820506|50832839	ENSSSCG00000000555,	-	13.5	0	-5.449658475	0.035918519
circRNA_006900	11:76193953|76202790	ENSSSCG00000024542,	-	13.5	0	-5.449658475	0.035918519
circRNA_006931	12:32369890|32381760	ENSSSCG00000017605,	MMD	13.5	0	-5.449658475	0.035918519
circRNA_007828	6:69990483|69994497	n/a	_	13.5	0	-5.449658475	0.035918519
circRNA_007058	13:138069734|138078218	ENSSSCG00000011817,	CCDC50	13.5	0	-5.449658475	0.035918519
circRNA_007335	17:1345076|1346223	ENSSSCG00000006971,	-	13.5	0	-5.449658475	0.035918519
circRNA_007236	15:59997322|60079510	n/a	_	13.5	0	-5.449658475	0.035918519
circRNA_001599	4:85304081|85304511	ENSSSCG00000006267,	PCMTD1	116	37.75	-1.618399537	0.035972378
circRNA_007551	3:48100930|48106804	ENSSSCG00000008113,	MRPS5	13.25	0	-5.446680439	0.036031964
circRNA_008123	9:62346713|62408681	n/a	_	13.25	0	-5.446680439	0.036031964
circRNA_006718	1:176376707|176394308	ENSSSCG00000004897,	ZCCHC2	13.25	0	-5.446680439	0.036031964
circRNA_006869	11:19712163|19723353	ENSSSCG00000009401,	RB1	13.25	0	-5.446680439	0.036031964
circRNA_007206	14:145714289|145730657	ENSSSCG00000010737,	FAM175B	13.25	0	-5.446680439	0.036031964
circRNA_010370	13:53872565|53902188	ENSSSCG00000011501,	SUCLG2	11.25	0	-5.448377466	0.036035155
circRNA_010870	9:29836166|29846096	ENSSSCG00000014949,	MED17	11.25	0	-5.448377466	0.036035155
circRNA_010531	17:59227820|59231706	ENSSSCG00000007477,	NFATC2	11.25	0	-5.448377466	0.036035155

circRNA_002590	1:135303493|135500271	ENSSSCG00000004646,	-	0	13	5.438143377	0.036244497
circRNA_002892	11:18595342|18605445	n/a	_	0	13	5.438143377	0.036244497
circRNA_003558	15:85325487|85326240	ENSSSCG00000015939,	-	0	13	5.438143377	0.036244497
circRNA_003293	14:9504665|9506415	ENSSSCG00000009645,	ADAMDEC1	0	13	5.438143377	0.036244497
circRNA_003883	2:11355620|11365773	ENSSSCG00000013125,	OSBP	0	13	5.438143377	0.036244497
circRNA_004249	4:36900162|36917862	ENSSSCG00000006054,	ATP6V1C1	0	13	5.438143377	0.036244497
circRNA_002622	1:159899221|159905970	ENSSSCG00000004845,	FAN1	0	13	5.438143377	0.036244497
circRNA_001122	17:51997158|52042134	ENSSSCG00000007368,	-	133.75	37.75	-1.584503716	0.036402871
circRNA_005575	11:466843|495629	ENSSSCG00000026744,	ZDHHC20	0	11	5.425768611	0.036793211
circRNA_005743	13:198955614|199067054	n/a	_	0	11	5.425768611	0.036793211
circRNA_005811	14:136612215|136683701	ENSSSCG00000010654,	ATRNL1	0	11	5.425768611	0.036793211
circRNA_006176	4:113204856|113255612	ENSSSCG00000006730,	MAN1A2	0	11	5.425768611	0.036793211
circRNA_005542	10:18123982|18149975	ENSSSCG00000010872,	AKT3	0	11	5.425768611	0.036793211
circRNA_006451	9:13693438|13704113	ENSSSCG00000014884,	-	0	11	5.425768611	0.036793211
circRNA_006492	9:102345868|102594047	n/a	_	0	11	5.425768611	0.036793211
circRNA_005931	17:52892065|52897001	ENSSSCG00000007383,	TOMM34	0	11	5.425768611	0.036793211
circRNA_006113	3:120136072|120155720	ENSSSCG00000008575,	ASXL2	0	11	5.425768611	0.036793211
circRNA_002338	GL893884.1:31770|47747	ENSSSCG00000027457,	UBE2Q2	134.25	48.75	-1.421524261	0.036831984
circRNA_010372	13:59859476|59864444	ENSSSCG00000011518,	SHQ1	11.25	0	-5.427954898	0.036849604
circRNA_001228	2:20100182|20116135	ENSSSCG00000021739,	HSD17B12	0	8.75	5.4196739	0.037165709
circRNA_001124	17:55235758|55247885	ENSSSCG00000007454,	ZMYND8	0	8.75	5.4196739	0.037165709
circRNA_000639	13:117567899|117594523	n/a	_	0	8.75	5.4196739	0.037165709
circRNA_001458	3:90751781|90755491	ENSSSCG00000008404,	MTIF2	0	8.75	5.4196739	0.037165709
circRNA_002324	GL892547.1:5870|14345	ENSSSCG00000024030,	-	0	8.75	5.4196739	0.037165709
circRNA_007705	4:139822139|139856337	ENSSSCG00000006927,	PKN2	12.75	0	-5.417451991	0.037231589
circRNA_006779	1:280497623|280505832	ENSSSCG00000005441,	IKBKAP	12.75	0	-5.417451991	0.037231589
circRNA_007968	7:124683310|124719293	n/a	_	12.75	0	-5.417451991	0.037231589
circRNA_007922	7:65167966|65184904	ENSSSCG00000001922,	ARIH1	12.25	0	-5.408040181	0.037554986
circRNA_007945	7:97744029|97746028	ENSSSCG00000002296,	RDH11	12.25	0	-5.408040181	0.037554986
circRNA_007411	18:59169585|59320297	n/a	_	12.25	0	-5.408040181	0.037554986
circRNA_007294	15:146201175|146205084	ENSSSCG00000022392,	COPS7B	12.25	0	-5.408040181	0.037554986
circRNA_008124	9:70812448|70812810	ENSSSCG00000028069,	-	12.25	0	-5.408040181	0.037554986
circRNA_007655	4:60858276|60860227	n/a	_	12.25	0	-5.408040181	0.037554986
circRNA_007632	4:32658673|32699400	ENSSSCG00000006035,	ANGPT1	12.25	0	-5.408040181	0.037554986
circRNA_011550	18:36480482|36515630	ENSSSCG00000025602,	-	12.25	0	-5.408850707	0.037586331
circRNA_011957	7:73287698|73291470	ENSSSCG00000001971,	HECTD1	12.25	0	-5.408850707	0.037586331
circRNA_010990	1:125491718|125499226	ENSSSCG00000004595,	ADAM10	12.25	0	-5.408850707	0.037586331
circRNA_011622	2:105165760|105179579	ENSSSCG00000014164,	-	12.25	0	-5.408850707	0.037586331
circRNA_011727	3:114039950|114049273	ENSSSCG00000008513,	BIRC6	12.25	0	-5.408850707	0.037586331
circRNA_011565	2:5926645|5927323	n/a	_	12.25	0	-5.408850707	0.037586331
circRNA_011513	17:30276183|30284814	ENSSSCG00000007097,	SEC23B	12.25	0	-5.408850707	0.037586331
circRNA_011754	4:35844936|35957356	n/a	_	12.25	0	-5.408850707	0.037586331
circRNA_011984	7:134694054|134704085	n/a	_	12.25	0	-5.408850707	0.037586331
circRNA_011994	8:32728074|32759376	ENSSSCG00000023112,	PDS5A	11.75	0	-5.392855321	0.038179745
circRNA_011886	6:82704332|82907607	n/a	_	11.75	0	-5.392855321	0.038179745
circRNA_011929	7:20645945|20651095	ENSSSCG00000001092,	TDP2	11.75	0	-5.392855321	0.038179745
circRNA_011198	13:19730728|19731015	ENSSSCG00000011229,	STT3B	11.75	0	-5.392855321	0.038179745
circRNA_011273	13:149836368|149854053	ENSSSCG00000011892,	MAATS1	11.75	0	-5.392855321	0.038179745
circRNA_011814	5:50595002|50627564	n/a	_	11.75	0	-5.392855321	0.038179745
circRNA_011529	17:57040110|57046105	ENSSSCG00000007460,	ARFGEF2	11.75	0	-5.392855321	0.038179745
circRNA_011229	13:72897241|72903860	ENSSSCG00000011540,	-	11.75	0	-5.392855321	0.038179745
circRNA_011325	14:48121030|48124333	ENSSSCG00000009967,	PITPNB	11.75	0	-5.392855321	0.038179745

circRNA_011710	3:92450105|92461755	ENSSSCG00000008412,	PSME4	11.75	0	-5.392855321	0.038179745
circRNA_011251	13:108395217|108399351	ENSSSCG00000011731,	SMC4	11.75	0	-5.392855321	0.038179745
circRNA_011987	8:3341344|3345924	ENSSSCG00000027349,	TBC1D14	11.75	0	-5.392855321	0.038179745
circRNA_000741	14:16172107|16181494	ENSSSCG00000026044,	FDFT1	20.75	91.5	2.177857957	0.038204202
circRNA_001243	2:47573699|47819708	n/a	_	134	255.25	1.231165021	0.038241406
circRNA_007914	7:57914632|57969573	ENSSSCG00000001810,	PDE8A	11.75	0	-5.391574487	0.038246082
circRNA_007036	13:104082327|104248782	n/a	_	11.75	0	-5.391574487	0.038246082
circRNA_007472	2:82502736|82558770	ENSSSCG00000022508,	UIMC1	11.75	0	-5.391574487	0.038246082
circRNA_006691	1:143245293|143275786	ENSSSCG00000004717,	UBR1	11.75	0	-5.391574487	0.038246082
circRNA_007408	18:52580507|52607712	ENSSSCG00000016717,	MPP6	11.75	0	-5.391574487	0.038246082
circRNA_007417	2:5185942|5186610	n/a	_	11.75	0	-5.391574487	0.038246082
circRNA_007487	2:91169756|91197361	ENSSSCG00000014126,	MSH3	11.75	0	-5.391574487	0.038246082
circRNA_011653	3:4984727|4986764	ENSSSCG00000030444,	-	11.5	0	-5.385469856	0.038512392
circRNA_011752	4:34502003|34641574	ENSSSCG00000006038,	ZFPM2	11.5	0	-5.385469856	0.038512392
circRNA_010963	1:40737383|40745673	ENSSSCG00000004220,	TRMT11	11.5	0	-5.385469856	0.038512392
circRNA_011847	5:94439224|94445562	ENSSSCG00000000912,	EEA1	11.5	0	-5.385469856	0.038512392
circRNA_012166	X:112398998|112413221	n/a	_	11.5	0	-5.385469856	0.038512392
circRNA_011671	3:39449044|39450156	ENSSSCG00000007948,	-	11.5	0	-5.385469856	0.038512392
circRNA_011695	3:74772971|74778873	ENSSSCG00000022708,	-	11.5	0	-5.385469856	0.038512392
circRNA_012157	X:70438343|70498760	ENSSSCG00000012434,	-	11.5	0	-5.385469856	0.038512392
circRNA_001386	3:11510637|11513212	ENSSSCG00000007721,	GTF2I	163	62	-1.319997194	0.038698014
circRNA_004558	6:64288025|64292459	ENSSSCG00000003398,	-	0	8.75	5.361099081	0.039462891
circRNA_003066	13:14159901|14175018	ENSSSCG00000011213,	TOP2B	0	8.75	5.361099081	0.039462891
circRNA_004302	4:79094980|79115918	ENSSSCG00000006231,	CHD7	0	8.75	5.361099081	0.039462891
circRNA_004873	8:1522764|1524515	ENSSSCG00000008697,	HTT	0	8.75	5.361099081	0.039462891
circRNA_004609	6:91839899|91919964	ENSSSCG00000003679,	-	0	8.75	5.361099081	0.039462891
circRNA_004717	7:12919796|12921890	n/a	_	0	8.75	5.361099081	0.039462891
circRNA_004761	7:46279250|46357590	ENSSSCG00000025551,	-	0	8.75	5.361099081	0.039462891
circRNA_004509	5:106607717|106610077	ENSSSCG00000025095,	-	0	8.75	5.361099081	0.039462891
circRNA_005083	9:25250548|25276993	ENSSSCG00000014927,	NOX4	0	8.75	5.361099081	0.039462891
circRNA_004871	8:1048082|1056670	n/a	_	0	8.75	5.361099081	0.039462891
circRNA_002646	1:195169049|195171383	ENSSSCG00000004997,	PRPF39	0	8.75	5.361099081	0.039462891
circRNA_005386	X:124531921|124639457	n/a	_	0	8.75	5.361099081	0.039462891
circRNA_003578	15:88315046|88322471	ENSSSCG00000015959,	RAPGEF4	0	8.75	5.361099081	0.039462891
circRNA_004527	6:12648433|12663291	ENSSSCG00000020668,	-	0	8.75	5.361099081	0.039462891
circRNA_005106	9:40859458|40862053	ENSSSCG00000027597,	-	0	8.75	5.361099081	0.039462891
circRNA_004225	4:2602892|2708122	ENSSSCG00000005934,	TRAPPC9	0	8.75	5.361099081	0.039462891
circRNA_007683	4:108244663|108258099	ENSSSCG00000006661,	VPS45	10.5	0	-5.354084595	0.039820674
circRNA_006996	13:52502787|52506744	ENSSSCG00000011498,	SLC25A26	10.5	0	-5.354084595	0.039820674
circRNA_007067	13:145987809|145990602	ENSSSCG00000025349,	CCDC14	10.5	0	-5.354084595	0.039820674
circRNA_006766	1:257754306|257762035	ENSSSCG00000005286,	CEP78	10.5	0	-5.354084595	0.039820674
circRNA_006875	11:21787387|21819817	ENSSSCG00000009414,	ZC3H13	10.5	0	-5.354084595	0.039820674
circRNA_007501	2:105082209|105156268	n/a	_	10.5	0	-5.354084595	0.039820674
circRNA_007647	4:44324577|44330177	n/a	_	10.5	0	-5.354084595	0.039820674
circRNA_007539	3:25026227|25231144	n/a	_	10.5	0	-5.354084595	0.039820674
circRNA_007279	15:120964226|120990471	ENSSSCG00000016127,	NDUFS1	10.5	0	-5.354084595	0.039820674
circRNA_007778	5:92237091|92247562	ENSSSCG00000000900,	-	10.5	0	-5.354084595	0.039820674
circRNA_004324	4:98616995|98618234	ENSSSCG00000006395,	TAGLN2	0	10.75	5.344885509	0.039992102
circRNA_004099	3:63158910|63171638	ENSSSCG00000008247,	SUCLG1	0	10.75	5.344885509	0.039992102
circRNA_004080	3:42066184|42077233	ENSSSCG00000022963,	-	0	10.75	5.344885509	0.039992102
circRNA_003507	15:22768074|22785191	ENSSSCG00000015710,	ACTR3	0	10.75	5.344885509	0.039992102
circRNA_004029	2:142518692|142524139	ENSSSCG00000014304,	SEC24A	0	10.75	5.344885509	0.039992102

circRNA_003829	18:18661914|18675443	ENSSSCG00000016549,	MKLN1	0	10.75	5.344885509	0.039992102
circRNA_004638	6:103686782|103695506	ENSSSCG00000003716,	SS18	0	10.75	5.344885509	0.039992102
circRNA_003239	13:166927031|166930220	ENSSSCG00000011953,	ZBTB11	0	10.75	5.344885509	0.039992102
circRNA_003295	14:10459275|10465714	ENSSSCG00000009650,	DOCK5	0	10.75	5.344885509	0.039992102
circRNA_003916	2:53089550|53096699	ENSSSCG00000013410,	SWAP70	0	10.75	5.344885509	0.039992102
circRNA_002631	1:181041196|181052777	ENSSSCG00000004934,	DPP8	0	10.75	5.344885509	0.039992102
circRNA_003030	12:45421101|45437647	ENSSSCG00000017748,	NF1	0	10.75	5.344885509	0.039992102
circRNA_003527	15:61383234|61398639	ENSSSCG00000015840,	WRN	0	10.75	5.344885509	0.039992102
circRNA_003625	15:120969439|120990471	ENSSSCG00000016127,	NDUFS1	0	10.75	5.344885509	0.039992102
circRNA_007295	15:157067652|157081773	ENSSSCG00000016395,	RIF1	11	0	-5.348847068	0.04000719
circRNA_006654	1:96792819|96806044	ENSSSCG00000004466,	TTK	11	0	-5.348847068	0.04000719
circRNA_010897	9:115756518|115779641	ENSSSCG00000026110,	SRPK2	10.25	0	-5.348172343	0.04007651
circRNA_010667	4:55335507|55364656	ENSSSCG00000006137,	WWP1	10.25	0	-5.348172343	0.04007651
circRNA_010165	1:16356085|16357950	ENSSSCG00000021273,	-	10.25	0	-5.348172343	0.04007651
circRNA_010289	10:56109733|56118632	ENSSSCG00000011074,	ARHGAP21	10.25	0	-5.348172343	0.04007651
circRNA_010806	7:91964457|91982115	ENSSSCG00000002266,	CHD2	10.25	0	-5.348172343	0.04007651
circRNA_010435	14:55592489|55869595	n/a	_	10.25	0	-5.348172343	0.04007651
circRNA_010894	9:113302666|113320145	ENSSSCG00000015414,	FAM185A	10.25	0	-5.348172343	0.04007651
circRNA_010240	1:233126038|233133377	ENSSSCG00000005191,	MPDZ	10.25	0	-5.348172343	0.04007651
circRNA_010722	5:90584373|90622092	n/a	_	10.25	0	-5.348172343	0.04007651
circRNA_010179	1:59726631|59733226	ENSSSCG00000004289,	CEP162	10.25	0	-5.348172343	0.04007651
circRNA_010657	4:39835278|39850235	n/a	_	10.5	0	-5.33228838	0.040687598
circRNA_010671	4:73577490|73591712	ENSSSCG00000006201,	ARFGEF1	10.5	0	-5.33228838	0.040687598
circRNA_010317	11:74618777|74621124	ENSSSCG00000026863,	-	10.5	0	-5.33228838	0.040687598
circRNA_010730	6:13110937|13117545	ENSSSCG00000030420,	GLG1	10.5	0	-5.33228838	0.040687598
circRNA_010879	9:51124702|51157112	n/a	_	10.5	0	-5.33228838	0.040687598
circRNA_010456	14:131390060|131408058	ENSSSCG00000010621,	ADD3	9.5	0	-5.315092952	0.041445264
circRNA_010887	9:77278140|77587010	ENSSSCG00000015307,	-	9.5	0	-5.315092952	0.041445264
circRNA_010727	5:106543058|106585027	n/a	_	9.5	0	-5.315092952	0.041445264
circRNA_010413	13:184052539|184059739	ENSSSCG00000012000,	-	9.5	0	-5.315092952	0.041445264
circRNA_010805	7:91925254|91950519	ENSSSCG00000002266,	CHD2	9.5	0	-5.315092952	0.041445264
circRNA_002538	1:94556573|94577735	ENSSSCG00000004460,	IBTK	0	10.75	5.305601531	0.041622844
circRNA_004088	3:54939544|54962024	n/a	_	0	10.75	5.305601531	0.041622844
circRNA_003098	13:34918254|34919128	ENSSSCG00000011373,	QRICH1	0	10.75	5.305601531	0.041622844
circRNA_003198	13:120826360|120827676	ENSSSCG00000021786,	-	0	10.75	5.305601531	0.041622844
circRNA_004044	2:150641324|150660330	ENSSSCG00000014399,	ARHGAP26	0	10.75	5.305601531	0.041622844
circRNA_003048	12:54621571|54665529	n/a	_	0	10.75	5.305601531	0.041622844
circRNA_004434	5:42104599|42116997	ENSSSCG00000000517,	CAPS2	0	10.75	5.305601531	0.041622844
circRNA_003045	12:52150744|52168979	ENSSSCG00000017877,	ANKFY1	0	10.75	5.305601531	0.041622844
circRNA_004236	4:16778493|16785890	ENSSSCG00000005983,	ATAD2	0	10.75	5.305601531	0.041622844
circRNA_002469	1:17123107|17134778	ENSSSCG00000004087,	CCDC170	0	10.75	5.305601531	0.041622844
circRNA_004167	3:108496197|108536751	ENSSSCG00000008488,	-	0	10.75	5.305601531	0.041622844
circRNA_004493	5:92098435|92114918	ENSSSCG00000030608,	METAP2	0	10.75	5.305601531	0.041622844
circRNA_005331	X:46125649|46128130	ENSSSCG00000012266,	SLC9A7	0	10.75	5.305601531	0.041622844
circRNA_005280	GL896504.1:29972|31588	ENSSSCG00000027115,	-	0	10.75	5.305601531	0.041622844
circRNA_007559	3:61090978|61099647	ENSSSCG00000008221,	KDM3A	11.25	0	-5.300373623	0.041871381
circRNA_006863	11:9504246|9507889	n/a	_	11.25	0	-5.300373623	0.041871381
circRNA_006945	12:45334013|45365521	ENSSSCG00000017748,	NF1	11.25	0	-5.300373623	0.041871381
circRNA_006905	12:4804161|4805931	ENSSSCG00000017177,	ST6GALNAC2	11.25	0	-5.300373623	0.041871381
circRNA_007754	5:63605306|63628804	ENSSSCG00000000625,	LRP6	11.25	0	-5.300373623	0.041871381
circRNA_007752	5:56918746|56928732	ENSSSCG00000000587,	AEBP2	11.25	0	-5.300373623	0.041871381
circRNA_007930	7:73100247|73111840	ENSSSCG00000001970,	HEATR5A	11.25	0	-5.300373623	0.041871381

circRNA_007449	2:60996624|60999141	ENSSSCG00000013856,	AP1M1	11.25	0	-5.300373623	0.041871381
circRNA_007687	4:116732431|116735096	ENSSSCG00000006760,	HIPK1	11.25	0	-5.300373623	0.041871381
circRNA_007660	4:67947307|67986787	ENSSSCG00000006179,	STAU2	11.25	0	-5.300373623	0.041871381
circRNA_007347	17:28241701|28268431	ENSSSCG00000007080,	-	11	0	-5.289397535	0.042318049
circRNA_006707	1:160169128|160271923	ENSSSCG00000004849,	APBA2	11	0	-5.289397535	0.042318049
circRNA_007098	14:14027675|14028151	ENSSSCG00000009682,	HMBOX1	11	0	-5.289397535	0.042318049
circRNA_006776	1:275780524|275806120	ENSSSCG00000005423,	-	11	0	-5.289397535	0.042318049
circRNA_008044	8:109011842|109042519	ENSSSCG00000009090,	KIAA1109	11	0	-5.289397535	0.042318049
circRNA_006805	10:12825482|12929568	n/a	_	11	0	-5.289397535	0.042318049
circRNA_006930	12:27608857|27616955	ENSSSCG00000017593,	UTP18	11	0	-5.289397535	0.042318049
circRNA_007618	3:134697661|134711682	ENSSSCG00000008635,	NOL10	11	0	-5.289397535	0.042318049
circRNA_006183	4:129968066|129973398	ENSSSCG00000006874,	PALMD	0	8.75	5.286903837	0.042564844
circRNA_006294	6:113003755|113122204	ENSSSCG00000027935,	FHOD3	0	8.75	5.286903837	0.042564844
circRNA_006192	5:9128682|9133379	ENSSSCG00000000145,	MYH9	0	8.75	5.286903837	0.042564844
circRNA_005582	11:15979885|15993129	ENSSSCG00000009376,	VPS36	0	8.75	5.286903837	0.042564844
circRNA_005498	1:225740193|225811866	ENSSSCG00000028406,	-	0	8.75	5.286903837	0.042564844
circRNA_010898	9:128129181|128170463	ENSSSCG00000015499,	-	9.25	0	-5.272764284	0.043174555
circRNA_010368	13:43409382|43417833	ENSSSCG00000011467,	DENND6A	9.25	0	-5.272764284	0.043174555
circRNA_010430	14:31918604|31979781	n/a	_	9.25	0	-5.272764284	0.043174555
circRNA_007227	15:51612536|51636427	ENSSSCG00000015777,	TRAPPC11	10.5	0	-5.263673976	0.043469085
circRNA_007946	7:97833510|97834004	ENSSSCG00000002298,	ZFYVE26	10.5	0	-5.263673976	0.043469085
circRNA_007143	14:65749789|65761210	n/a	_	10.5	0	-5.263673976	0.043469085
circRNA_007760	5:70576776|70629435	ENSSSCG00000000755,	ERC1	10.5	0	-5.263673976	0.043469085
circRNA_007893	7:112298|130444	ENSSSCG00000000985,	EXOC2	10.5	0	-5.263673976	0.043469085
circRNA_007318	16:38670029|38677510	n/a	_	10.5	0	-5.263673976	0.043469085
circRNA_007474	2:83218053|83229420	ENSSSCG00000014062,	SIMC1	10.5	0	-5.263673976	0.043469085
circRNA_008055	8:127051429|127054470	ENSSSCG00000009162,	SLC9B1	10.5	0	-5.263673976	0.043469085
circRNA_007755	5:68106961|68128063	ENSSSCG00000000723,	C12orf4	10.5	0	-5.263673976	0.043469085
circRNA_011979	7:124952553|124954675	ENSSSCG00000002505,	PAPOLA	10	0	-5.245838791	0.044246035
circRNA_011594	2:54339863|54395084	ENSSSCG00000013984,	SNAP47	10	0	-5.245838791	0.044246035
circRNA_011175	12:37690214|37691140	ENSSSCG00000017672,	MED13	10	0	-5.245838791	0.044246035
circRNA_011598	2:69998260|69999081	ENSSSCG00000013630,	DNM2	10	0	-5.245838791	0.044246035
circRNA_011562	2:3009236|3109853	n/a	_	10	0	-5.245838791	0.044246035
circRNA_011776	4:73920402|73950380	n/a	_	10	0	-5.245838791	0.044246035
circRNA_011291	13:207175610|207188840	n/a	_	10	0	-5.245838791	0.044246035
circRNA_011698	3:76466177|76482619	ENSSSCG00000008339,	GFPT1	10	0	-5.245838791	0.044246035
circRNA_011995	8:32861923|32893069	n/a	_	10	0	-5.245838791	0.044246035
circRNA_011837	5:87157448|87174095	ENSSSCG00000000873,	ANO4	10	0	-5.245838791	0.044246035
circRNA_010950	1:13973894|14086548	n/a	_	10	0	-5.245838791	0.044246035
circRNA_011075	1:305174205|305175146	ENSSSCG00000005711,	NUP214	10	0	-5.245838791	0.044246035
circRNA_011899	6:108611407|108627548	ENSSSCG00000003730,	RNF138	10	0	-5.245838791	0.044246035
circRNA_011276	13:156013911|156023826	ENSSSCG00000024663,	SPICE1	10	0	-5.245838791	0.044246035
circRNA_008185	GL892789.1:66335|94569	n/a	_	9.75	0	-5.22702754	0.044998892
circRNA_007465	2:79596775|79619142	ENSSSCG00000014009,	CNOT6	9.75	0	-5.22702754	0.044998892
circRNA_006901	11:86869925|86879529	ENSSSCG00000009570,	MPHOSPH8	9.75	0	-5.22702754	0.044998892
circRNA_006906	12:5063509|5067513	ENSSSCG00000017184,	PRPSAP1	9.75	0	-5.22702754	0.044998892
circRNA_006655	1:97325990|97332007	ENSSSCG00000004469,	LCA5	9.75	0	-5.22702754	0.044998892
circRNA_007064	13:144109883|144114121	ENSSSCG00000011854,	LRCH3	9.75	0	-5.22702754	0.044998892
circRNA_008239	X:16343606|16355173	ENSSSCG00000030274,	PPEF1	9.75	0	-5.22702754	0.044998892
circRNA_008018	8:69704199|69711465	ENSSSCG00000008921,	-	9.75	0	-5.22702754	0.044998892
circRNA_011054	1:256337526|256346024	ENSSSCG00000005278,	PRUNE2	9.5	0	-5.220405592	0.045311876
circRNA_011774	4:73730788|73750002	ENSSSCG00000006202,	-	9.5	0	-5.220405592	0.045311876

circRNA_012117	GL893884.1:17986|41304	ENSSSCG00000027457,	UBE2Q2	9.5	0	-5.220405592	0.045311876
circRNA_011864	6:13110937|13115563	ENSSSCG00000030420,	GLG1	9.5	0	-5.220405592	0.045311876
circRNA_010987	1:124875218|124884580	ENSSSCG00000004587,	MYO1E	9.5	0	-5.220405592	0.045311876
circRNA_011094	10:29342271|29360924	ENSSSCG00000010928,	KDM5B	9.5	0	-5.220405592	0.045311876
circRNA_011600	2:73695861|73696503	ENSSSCG00000013533,	-	9.5	0	-5.220405592	0.045311876
circRNA_011580	2:34839877|34879139	ENSSSCG00000013332,	KIF18A	9.5	0	-5.220405592	0.045311876
circRNA_011686	3:61098540|61108481	ENSSSCG00000008221,	KDM3A	9.5	0	-5.220405592	0.045311876
circRNA_011954	7:73042261|73064245	ENSSSCG00000001970,	HEATR5A	9.5	0	-5.220405592	0.045311876
circRNA_011469	16:23613621|23649775	n/a	_	9.5	0	-5.220405592	0.045311876
circRNA_011404	15:61774735|61782824	ENSSSCG00000015844,	GSR	9.5	0	-5.220405592	0.045311876
circRNA_011385	14:142803973|142840902	ENSSSCG00000010699,	ATE1	9.5	0	-5.220405592	0.045311876
circRNA_011422	15:88381955|88389043	ENSSSCG00000015959,	RAPGEF4	9.5	0	-5.220405592	0.045311876
circRNA_011956	7:73081330|73086240	ENSSSCG00000001970,	HEATR5A	9.5	0	-5.220405592	0.045311876
circRNA_004972	8:89359060|89364758	ENSSSCG00000009047,	SMARCA5	78	6.5	-3.326346623	0.046409966
circRNA_000433	11:86933456|86962417	ENSSSCG00000009569,	PSPC1	15.5	101	2.754738814	0.046805712
circRNA_007485	2:90152679|90158760	ENSSSCG00000014114,	PAPD4	8.5	0	-5.153802411	0.048348261
circRNA_007605	3:120307356|120350634	ENSSSCG00000008576,	-	8.5	0	-5.153802411	0.048348261
circRNA_007298	16:6173606|6186594	ENSSSCG00000016792,	FAM134B	8.5	0	-5.153802411	0.048348261
circRNA_007451	2:65119742|65120211	ENSSSCG00000013776,	DDX39A	8.5	0	-5.153802411	0.048348261
circRNA_007484	2:90151050|90166516	ENSSSCG00000014114,	PAPD4	8.5	0	-5.153802411	0.048348261
circRNA_010930	X:16108943|16112710	ENSSSCG00000012156,	CDKL5	8.25	0	-5.146654552	0.04870546
circRNA_010450	14:116344443|116348772	n/a	_	8.25	0	-5.146654552	0.04870546
circRNA_010871	9:36992574|37001797	ENSSSCG00000027529,	BIRC2	8.25	0	-5.146654552	0.04870546
circRNA_010831	8:46471841|46490433	ENSSSCG00000008866,	GUCY1A3	8.25	0	-5.146654552	0.04870546
circRNA_010744	6:61842290|61923073	n/a	_	8.25	0	-5.146654552	0.04870546
circRNA_004773	7:59801505|59817124	ENSSSCG00000001837,	FANCI	0	8.5	5.141051264	0.049240634
circRNA_004199	3:120287019|120350634	ENSSSCG00000008576,	-	0	8.5	5.141051264	0.049240634
circRNA_002887	11:13202083|13224528	ENSSSCG00000009361,	POSTN	0	8.5	5.141051264	0.049240634
circRNA_004583	6:81306905|81322888	ENSSSCG00000028074,	-	0	8.5	5.141051264	0.049240634
circRNA_003122	13:50653633|50658407	ENSSSCG00000011496,	ADAMTS9	0	8.5	5.141051264	0.049240634
circRNA_004130	3:84814587|84822563	n/a	_	0	8.5	5.141051264	0.049240634
circRNA_005334	X:51812778|51813630	ENSSSCG00000012328,	HUWE1	0	8.5	5.141051264	0.049240634
circRNA_004030	2:142624803|142634560	ENSSSCG00000026644,	DDX46	0	8.5	5.141051264	0.049240634
circRNA_003173	13:91207372|91249175	ENSSSCG00000011681,	TRPC1	0	8.5	5.141051264	0.049240634
circRNA_003746	17:13088600|13092479	ENSSSCG00000007030,	IKBKB	0	8.5	5.141051264	0.049240634
circRNA_003634	15:124483398|124720160	n/a	_	0	8.5	5.141051264	0.049240634
circRNA_003704	16:47777338|47778112	n/a	_	0	8.5	5.141051264	0.049240634
circRNA_004327	4:103267690|103273573	ENSSSCG00000006508,	-	0	8.5	5.141051264	0.049240634
circRNA_004182	3:114087675|114096420	ENSSSCG00000008513,	BIRC6	0	8.5	5.141051264	0.049240634
circRNA_004191	3:120081729|120136187	ENSSSCG00000008575,	ASXL2	0	8.5	5.141051264	0.049240634
circRNA_007244	15:68610560|68795464	n/a	_	121.5	10.75	-3.563901727	0.049895022
circRNA_000946	15:79683656|79707046	ENSSSCG00000015902,	-	25.75	90	2.029079794	0.049900224

Table S2-2. Differential expression analysis of circRNAs in the FH vs. FL comparison.

circRNA_ID	Locus	Gene_id	GeneSymbol	Value (FH)	Value (FL)	log2(FoldChange)	*P* value
circRNA_007796	6:25836379|25914846	n/a	_	0	719	12.91430056	6.51E-14
circRNA_000008	1:12075808|12234659	n/a	_	0	289	10.25335438	1.45E-06
circRNA_012118	GL893884.1:31770|41304	ENSSSCG00000027457,	UBE2Q2	850.25	0	-10.12824192	3.35E-06
circRNA_008114	9:50848480|50856753	ENSSSCG00000029649,	IFT46	0	54.25	9.288763965	3.75E-06
circRNA_009634	6:52578943|52597083	n/a	_	0	42.5	8.745150115	2.92E-05
circRNA_001598	4:83633287|83686353	n/a	_	0	61.25	8.795792192	8.54E-05
circRNA_001402	3:32848231|32861950	ENSSSCG00000026569,	-	62.75	0	-8.21450311	8.90E-05
circRNA_005272	GL896250.1:7783|16183	ENSSSCG00000027508,	-	66.5	0	-8.207036581	8.95E-05
circRNA_007499	2:105006195|105146567	n/a	_	110	0	-8.470613008	0.00010054
circRNA_010779	7:1978363|1987535	n/a	_	0	43.25	8.42970477	0.00011558
circRNA_013750	15:124782328|124789687	ENSSSCG00000016156,	ACADL	63.75	0	-8.087723707	0.000136023
circRNA_007702	4:136805197|136820329	ENSSSCG00000006911,	-	54	0	-8.014805052	0.000175888
circRNA_013941	6:150842053|150895226	ENSSSCG00000003881,	SPATA6	0	40.75	8.240495035	0.000200038
circRNA_005049	9:8944659|8950452	ENSSSCG00000014828,	-	50.5	0	-7.848530871	0.00026758
circRNA_001558	4:35844936|35911000	ENSSSCG00000006045,	-	93.75	0	-8.25618465	0.00027531
circRNA_013762	1:1098250|1349139	n/a	_	0	34.25	8.113456266	0.000284307
circRNA_001433	3:62982989|63080743	n/a	_	97.25	1455.5	4.722752967	0.000330232
circRNA_000197	1:216314524|216447657	n/a	_	50.25	0	-7.760032056	0.000344555
circRNA_009587	5:88167732|88170249	ENSSSCG00000026697,	UHRF1BP1L	0	39.25	8.019858553	0.000356971
circRNA_012372	10:49568556|49595487	ENSSSCG00000011040,	CACNB2	55.5	0	-7.75363978	0.000363344
circRNA_009785	7:89134396|89135137	n/a	_	70.25	0	-7.877246902	0.000376432
circRNA_012503	12:62407440|62416175	ENSSSCG00000018039,	NCOR1	46.75	0	-7.643033926	0.000461625
circRNA_013010	3:57772902|57787529	ENSSSCG00000008186,	TSGA10	0	25.25	7.932841848	0.000462673
circRNA_003926	2:71199117|71202659	ENSSSCG00000013602,	HNRNPM	44.25	0	-7.646716785	0.000474322
circRNA_000696	13:170324267|170392219	n/a	_	51.75	0	-7.791149026	0.000477323
circRNA_003180	13:98155627|98191973	ENSSSCG00000027459,	RNF13	48.75	0	-7.634371761	0.000478477
circRNA_006327	7:24626858|24644364	n/a	_	0	1065.75	8.511742915	0.000488129
circRNA_014024	6:156189678|156190959	n/a	_	57	0	-7.773593193	0.00049658
circRNA_004144	3:92450641|92461755	ENSSSCG00000008412,	PSME4	45	0	-7.604397015	0.000517689
circRNA_004535	6:18449174|18463615	ENSSSCG00000002799,	CNOT1	0	31	7.867587791	0.000526506
circRNA_004245	4:35980007|36062940	n/a	_	66.5	0	-7.895423836	0.000568468
circRNA_009392	3:104168480|104171085	ENSSSCG00000008467,	EML4	0	27.25	7.819255535	0.000595714
circRNA_006076	3:57385109|57402361	ENSSSCG00000008179,	REV1	45.5	0	-7.539850124	0.000611111
circRNA_003374	14:77540655|77542707	ENSSSCG00000020688,	HNRNPH3	60.75	0	-7.678911014	0.000628503
circRNA_000685	13:166909686|166917343	ENSSSCG00000011953,	ZBTB11	42	0	-7.486597264	0.000693063
circRNA_002620	1:154901709|154916931	n/a	_	43.25	0	-7.495825732	0.000703288
circRNA_008336	1:86577009|86580240	ENSSSCG00000025703,	-	40.25	0	-7.436291602	0.000797369
circRNA_004442	5:50595002|50601679	n/a	_	40.25	0	-7.436291602	0.000797369
circRNA_012358	10:36414580|36437146	n/a	_	0	358.5	8.208551327	0.000815815
circRNA_009360	3:56414602|56418177	n/a	_	60.5	0	-7.568841718	0.00081956
circRNA_003613	15:116425424|116441903	n/a	_	44.25	0	-7.424909045	0.000824853
circRNA_002157	8:141415791|141420967	ENSSSCG00000026655,	PTPN13	0	28.5	7.673473155	0.000827926
circRNA_007236	15:59997322|60079510	n/a	_	0	22.25	7.670122395	0.000852529
circRNA_004321	4:98051660|98065430	ENSSSCG00000006386,	COPA	53.25	0	-7.498740335	0.00096792
circRNA_003146	13:81190634|81195086	ENSSSCG00000011628,	DNAJC13	38	0	-7.352501472	0.000969951
circRNA_012259	1:135829826|135910108	n/a	_	0	23.75	7.596581689	0.000997946
circRNA_011555	18:49340344|49341282	ENSSSCG00000016692,	-	47.75	0	-7.422048429	0.001188939
circRNA_013289	6:148116282|148136997	ENSSSCG00000021292,	OSBPL9	37.25	0	-7.259094019	0.00122954
circRNA_014002	13:207192306|207194401	n/a	_	45.5	0	-7.393789719	0.001245498

circRNA_001655	4:129789461|129792899	ENSSSCG00000006872,	-	34.75	0	-7.194166556	0.001413492
circRNA_000484	12:37781159|37787288	ENSSSCG00000017673,	INTS2	36.25	0	-7.181583102	0.001471315
circRNA_001874	6:101724150|101813590	ENSSSCG00000003712,	OSBPL1A	31.5	0	-7.14599081	0.001600365
circRNA_008434	1:206990528|206993410	ENSSSCG00000005066,	EXOC5	31.5	0	-7.132825194	0.00163236
circRNA_011774	4:73730788|73750002	ENSSSCG00000006202,	-	40.5	0	-7.267338896	0.001652784
circRNA_004760	7:43188490|43197345	ENSSSCG00000001641,	-	33.75	0	-7.11814661	0.001661421
circRNA_014017	3:75420602|75437890	n/a	_	38.75	0	-7.271325242	0.001686005
circRNA_006658	1:101693272|101694439	n/a	_	33.75	0	-7.09883463	0.001728475
circRNA_007645	4:39915930|39943326	ENSSSCG00000006069,	RGS22	34.5	0	-7.088966395	0.001769318
circRNA_007043	13:126270468|126299787	n/a	_	0	26.75	7.291786158	0.001892266
circRNA_010055	GL893689.1:47831|52877	ENSSSCG00000022145,	RPF1	46	0	-7.182493738	0.001944373
circRNA_001889	6:108173849|108202404	ENSSSCG00000025005,	B4GALT6	37.5	0	-7.182473764	0.002002584
circRNA_006791	1:293511501|293517866	ENSSSCG00000005516,	STOM	43.75	0	-7.171775799	0.002017672
circRNA_001556	4:32672829|32715543	ENSSSCG00000006035,	ANGPT1	39	0	-7.163628084	0.002064855
circRNA_006953	12:62413971|62416175	ENSSSCG00000018039,	NCOR1	31	0	-7.011115276	0.002094693
circRNA_005609	12:315609|323714	ENSSSCG00000017136,	TBCD	45.5	0	-7.149946949	0.002102211
circRNA_002928	11:55451765|55459959	ENSSSCG00000029113,	RNF219	31.5	0	-7.011989993	0.002111764
circRNA_001934	6:153309526|153393008	ENSSSCG00000003917,	TESK2	39.5	0	-7.141754098	0.00216132
circRNA_007183	14:122832506|122841532	ENSSSCG00000010571,	C10orf76	43.5	0	-7.133419813	0.002167184
circRNA_007432	2:30750436|30755936	ENSSSCG00000013315,	EIF3M	29.75	0	-6.948222031	0.002438605
circRNA_013912	4:48056407|48140232	n/a	_	0	21.25	7.155079021	0.002517879
circRNA_005237	GL893884.1:23405|47747	ENSSSCG00000027457,	UBE2Q2	38	0	-7.067032821	0.002555099
circRNA_005621	12:23750110|23756693	ENSSSCG00000017513,	NPEPPS	28.5	0	-6.907733884	0.002626569
circRNA_006378	7:130882992|130887622	ENSSSCG00000002547,	-	0	24.5	7.114786876	0.002709634
circRNA_001108	17:43061013|43089928	ENSSSCG00000007280,	ITCH	27.5	0	-6.886156907	0.002764419
circRNA_000160	1:180714491|180715180	ENSSSCG00000004927,	CLPX	41.75	0	-6.999720608	0.002869305
circRNA_000240	1:270464385|270476354	ENSSSCG00000005389,	TEX10	36.25	0	-6.987828376	0.002974214
circRNA_012992	3:11449859|11454606	ENSSSCG00000007721,	GTF2I	35.75	0	-6.974360155	0.003024306
circRNA_005651	13:24353502|24371577	ENSSSCG00000011245,	-	35.25	0	-6.970110484	0.00305714
circRNA_013049	3:119558816|119703933	n/a	_	0	70.75	7.364986404	0.00306151
circRNA_008084	9:13256666|13259345	ENSSSCG00000014881,	CLNS1A	38.5	0	-6.959197528	0.003122162
circRNA_012516	13:32774767|32862490	n/a	_	0	68.75	7.342062409	0.003166692
circRNA_005975	2:41825697|41930028	n/a	_	0	69.25	7.333329888	0.003207608
circRNA_005613	12:16053861|16071225	ENSSSCG00000017301,	TLK2	38.25	0	-6.940441408	0.003227731
circRNA_003654	15:155094318|155097886	n/a	_	41.5	0	-6.935008842	0.003335244
circRNA_006128	4:32646874|32699400	ENSSSCG00000006035,	ANGPT1	30.75	0	-6.914023377	0.003469009
circRNA_012123	GL894597.1:33450|45776	n/a	_	38	0	-6.899597426	0.003515102
circRNA_006639	1:61006639|61011614	ENSSSCG00000004294,	SYNCRIP	0	18	6.973788816	0.00353769
circRNA_013944	7:76463695|76509406	n/a	_	0	23.5	6.95831107	0.003607453
circRNA_003834	18:18690198|18715721	ENSSSCG00000016549,	MKLN1	32.25	0	-6.892454931	0.003609317
circRNA_006504	9:125610011|125614494	ENSSSCG00000015482,	VAMP4	38.5	0	-6.881218174	0.003615791
circRNA_004787	7:65188149|65192947	ENSSSCG00000001922,	ARIH1	34	0	-6.878740735	0.003689249
circRNA_002031	7:105392983|105408581	ENSSSCG00000002387,	GPATCH2L	39	0	-6.865707142	0.003696261
circRNA_006112	3:120081729|120141921	ENSSSCG00000008575,	ASXL2	39	0	-6.865707142	0.003696261
circRNA_013952	8:75546785|75573210	ENSSSCG00000008971,	USO1	0	20	6.946487221	0.003703628
circRNA_014013	16:29110372|29115875	n/a	_	33.25	0	-6.872209916	0.003718805
circRNA_001744	5:73490736|73514893	ENSSSCG00000000779,	KIF21A	27.75	0	-6.730781452	0.003742079
circRNA_003451	14:131872215|131874408	ENSSSCG00000010625,	SMC3	36.75	0	-6.843764248	0.003921014
circRNA_012838	17:31763922|31790621	ENSSSCG00000028257,	-	0	54.75	7.19450289	0.003925208
circRNA_005877	15:137710958|137952597	n/a	_	0	54.75	7.188811965	0.00395748
circRNA_005526	1:297466978|297468840	ENSSSCG00000023197,	-	32.5	0	-6.823855232	0.004062561
circRNA_013992	1:293274600|293300073	n/a	_	36.5	0	-6.82044815	0.00406859

circRNA_013857	15:133579638|133595116	ENSSSCG00000016194,	USP37	0	20.5	6.887774638	0.00410853
circRNA_012054	9:29830958|29840619	ENSSSCG00000014949,	MED17	0	20.5	6.887774638	0.00410853
circRNA_002112	8:83452129|83472898	ENSSSCG00000009021,	-	36	0	-6.813126528	0.004117748
circRNA_000277	10:9709752|9731216	ENSSSCG00000027207,	-	0	19	6.869156185	0.004255479
circRNA_000839	14:113066724|113077453	ENSSSCG00000010467,	-	31.75	0	-6.78702814	0.004361941
circRNA_001454	3:84178465|84179842	ENSSSCG00000028228,	XPO1	37.75	0	-6.779116818	0.00447895
circRNA_014037	1:145412858|145417869	ENSSSCG00000004753,	INO80	34	0	-6.764403991	0.004546128
circRNA_012467	12:14819804|14837065	ENSSSCG00000017278,	TEX2	32.5	0	-6.756568217	0.004571118
circRNA_008313	1:45640355|45737883	ENSSSCG00000004242,	TBC1D32	32.75	0	-6.756564837	0.004609648
circRNA_006106	3:113967495|113977210	ENSSSCG00000008513,	BIRC6	0	19	6.811808145	0.004702058
circRNA_003909	2:47993416|47999371	ENSSSCG00000013392,	-	0	16.5	6.803508885	0.004790952
circRNA_004107	3:74727498|74773032	n/a	_	30.25	0	-6.7270544	0.004853165
circRNA_014912	11:49690381|49708915	ENSSSCG00000022078,	-	0	55.25	7.04519554	0.00485473
circRNA_013894	3:9520792|9527059	ENSSSCG00000007684,	PRKRIP1	0	17.5	6.791665179	0.00488085
circRNA_012793	16:22354808|22377921	ENSSSCG00000016833,	CAPSL	0	42.5	7.027442053	0.004977408
circRNA_005881	16:4095540|4198345	n/a	_	0	52.5	7.02375098	0.005003258
circRNA_003099	13:34925731|34926774	ENSSSCG00000011373,	QRICH1	34.5	0	-6.699375448	0.005040256
circRNA_000111	1:128682226|128695939	n/a	_	34.5	0	-6.702642491	0.005056919
circRNA_001996	7:73042261|73057496	ENSSSCG00000001970,	HEATR5A	34.5	0	-6.702642491	0.005056919
circRNA_008446	1:229131344|229195144	ENSSSCG00000005178,	CNTLN	30.25	0	-6.675668818	0.005344417
circRNA_001809	6:63443230|63452235	ENSSSCG00000025445,	-	34	0	-6.661810252	0.005393004
circRNA_011202	13:24111612|24132299	ENSSSCG00000011243,	GOLGA4	27.5	0	-6.664617066	0.005447722
circRNA_001486	3:107521846|107610973	n/a	_	84	348.5	2.975458173	0.005499342
circRNA_010939	X:85916081|85961011	ENSSSCG00000012474,	DIAPH2	26.75	0	-6.652547177	0.005571136
circRNA_009530	5:32833666|32887030	n/a	_	22	0	-6.506335711	0.005599096
circRNA_013985	1:127637224|127676835	ENSSSCG00000026960,	ZNF280D	30.25	0	-6.643778935	0.00562101
circRNA_012395	10:64310358|64322806	n/a	_	0	36.25	6.915177449	0.005819228
circRNA_011669	3:29719350|29720141	n/a	_	32	0	-6.617304549	0.005843768
circRNA_007235	15:59538969|59541410	ENSSSCG00000015833,	TTI2	30.5	0	-6.600123984	0.006083651
circRNA_009251	2:61866062|61867966	ENSSSCG00000022227,	BRD4	27.25	0	-6.575799591	0.006367647
circRNA_004712	7:4587208|4621123	n/a	_	27.25	0	-6.564035926	0.006465317
circRNA_007144	14:66224279|66231536	ENSSSCG00000010200,	CSGALNACT2	32.25	0	-6.556780623	0.006497545
circRNA_002098	8:74357360|74402890	ENSSSCG00000008961,	MTHFD2L	27	0	-6.558025017	0.006553867
circRNA_014009	15:62040539|62045562	ENSSSCG00000015846,	RBPMS	26.5	0	-6.557795981	0.006601647
circRNA_004434	5:42104599|42116997	ENSSSCG00000000517,	CAPS2	0	38.25	6.807226831	0.006745709
circRNA_013998	12:49749481|49750243	ENSSSCG00000017817,	PRPF8	28.5	0	-6.535203379	0.006756873
circRNA_014047	15:130331479|130335001	ENSSSCG00000016173,	ATIC	31.75	0	-6.529552474	0.006794003
circRNA_008027	8:80764338|80786409	ENSSSCG00000009010,	ARFIP1	24.5	0	-6.53894062	0.006807513
circRNA_011662	3:19058493|19062370	ENSSSCG00000007812,	XPO6	24.25	0	-6.533547053	0.006875196
circRNA_007254	15:85042800|85046450	ENSSSCG00000015933,	FASTKD1	0	38.75	6.775183526	0.007044784
circRNA_013788	1:233128640|233186782	ENSSSCG00000005191,	MPDZ	0	16	6.568752922	0.007069462
circRNA_003270	13:210819581|210845206	ENSSSCG00000012062,	TTC3	29.75	0	-6.506355485	0.007085056
circRNA_005391	X:127725495|127741869	n/a	_	29	0	-6.505387684	0.007106647
circRNA_000850	14:117646846|117651080	ENSSSCG00000010508,	TM9SF3	32	0	-6.504720296	0.007108225
circRNA_014036	1:143995379|144007016	ENSSSCG00000004730,	TMEM87A	32	0	-6.504720296	0.007108225
circRNA_002408	X:13331565|13339634	ENSSSCG00000012142,	AP1S2	25.25	0	-6.507164535	0.007156325
circRNA_007449	2:60996624|60999141	ENSSSCG00000013856,	AP1M1	29.75	0	-6.491640458	0.007286971
circRNA_014551	9:49298595|49299945	ENSSSCG00000015071,	SIK3	0	38.25	6.742465346	0.007362203
circRNA_003129	13:58776339|58816633	n/a	_	29.5	0	-6.479095112	0.007397211
circRNA_014927	2:5807333|5807491	n/a	_	0	28.75	6.717634013	0.007611477
circRNA_000992	15:115686849|115963395	n/a	_	279.75	404	1.212139933	0.007893385
circRNA_012379	10:53370958|53376653	ENSSSCG00000021311,	-	0	26	6.685499536	0.007945109

circRNA_003783	17:45690868|45697572	ENSSSCG00000007331,	RBL1	25	0	-6.437798683	0.0080693
circRNA_005848	15:85340631|85357154	ENSSSCG00000015939,	-	26.25	0	-6.423810897	0.00813237
circRNA_014053	6:79983161|79983682	n/a	_	27.25	0	-6.406635287	0.008409391
circRNA_008465	1:249743639|249911706	n/a	_	27.5	0	-6.407466927	0.008451733
circRNA_009456	4:48134546|48140232	ENSSSCG00000006115,	RUNX1T1	25.5	0	-6.408362043	0.008461467
circRNA_001918	6:146619070|146622749	ENSSSCG00000003848,	LRP8	23.25	0	-6.392339984	0.008612119
circRNA_007067	13:145987809|145990602	ENSSSCG00000025349,	CCDC14	27.25	0	-6.387928242	0.008635409
circRNA_007069	13:148444729|148446635	ENSSSCG00000011882,	GOLGB1	27.25	0	-6.385602887	0.008651021
circRNA_012932	2:66875163|66896675	n/a	_	0	24.25	6.619428738	0.008671838
circRNA_014939	5:51567416|51765461	n/a	_	0	24.25	6.619428738	0.008671838
circRNA_010338	12:37378287|37380695	ENSSSCG00000017669,	TUBD1	22	0	-6.392922134	0.008687095
circRNA_003336	14:34675990|34695463	ENSSSCG00000009834,	ATXN2	25.5	0	-6.389769111	0.008687796
circRNA_006645	1:86322010|86338016	ENSSSCG00000004394,	RPF2	25.75	0	-6.384717584	0.008695587
circRNA_014008	15:59683347|59692013	n/a	_	27.75	0	-6.380766442	0.008726226
circRNA_009970	9:51067117|51079497	ENSSSCG00000015097,	DDX6	23.5	0	-6.379443183	0.008779447
circRNA_004604	6:88476536|88480409	ENSSSCG00000003660,	PABPC4	27.5	0	-6.370995689	0.008824228
circRNA_000597	13:73329729|73340904	ENSSSCG00000011563,	FANCD2	27.75	0	-6.369184305	0.008888147
circRNA_007849	6:99525347|99544888	ENSSSCG00000025478,	MIB1	27.25	0	-6.370705477	0.008888641
circRNA_007852	6:101724150|101804423	ENSSSCG00000003712,	OSBPL1A	27.25	0	-6.370705477	0.008888641
circRNA_000999	15:117863548|117910594	n/a	_	27.25	0	-6.365333765	0.008903394
circRNA_014010	15:101703689|101710376	ENSSSCG00000016027,	ITGAV	25.5	0	-6.367360624	0.008988324
circRNA_002028	7:102696376|102720215	ENSSSCG00000002347,	DNAL1	28.25	0	-6.342936091	0.009298406
circRNA_014943	7:22160522|22314115	n/a	_	0	22	6.556459685	0.009418231
circRNA_014930	3:57812353|57828426	ENSSSCG00000008186,	TSGA10	0	22	6.556459685	0.009418231
circRNA_012191	1:23213758|23252035	n/a	_	27.5	0	-6.311417066	0.009667617
circRNA_006555	X:16250920|16256906	ENSSSCG00000012156,	CDKL5	27.5	0	-6.311417066	0.009667617
circRNA_014015	17:43054927|43082579	ENSSSCG00000007280,	ITCH	24	0	-6.316868816	0.009716206
circRNA_001315	2:101213695|101360310	n/a	_	0	28.5	6.526489679	0.009792868
circRNA_008374	1:134611516|134618061	ENSSSCG00000004636,	TRPM7	24	0	-6.305862614	0.00982626
circRNA_000908	15:51637238|51641585	ENSSSCG00000015777,	TRAPPC11	23.25	0	-6.305992358	0.009871735
circRNA_014021	5:63625257|63632464	ENSSSCG00000000625,	LRP6	23.25	0	-6.305992358	0.009871735
circRNA_000532	13:14168314|14175033	ENSSSCG00000011213,	TOP2B	23.25	0	-6.295165371	0.010036624
circRNA_014014	17:9407803|9413066	ENSSSCG00000007000,	FAT1	24.5	0	-6.27797408	0.010237148
circRNA_008219	GL896192.1:22918|26125	ENSSSCG00000028008,	VAMP3	21	0	-6.276580265	0.010363408
circRNA_007438	2:47522256|47702515	n/a	_	21	0	-6.276580265	0.010363408
circRNA_012376	10:53339997|53461410	n/a	_	0	20.25	6.481366958	0.010381555
circRNA_001063	16:47778049|47800473	n/a	_	24.25	0	-6.269718524	0.010382419
circRNA_003461	14:136289720|136311233	ENSSSCG00000010652,	FAM160B1	25.25	0	-6.266426073	0.010427518
circRNA_013162	5:33031868|33033688	ENSSSCG00000026004,	MSRB3	22.25	0	-6.265957387	0.010480749
circRNA_000634	13:108413402|108415199	ENSSSCG00000011731,	SMC4	24.75	0	-6.265673827	0.010495912
circRNA_008475	1:264881379|264891198	ENSSSCG00000005343,	RNF38	25	0	-6.261576299	0.010546444
circRNA_008709	13:6091992|6097905	ENSSSCG00000011201,	SATB1	25.25	0	-6.261221246	0.010658513
circRNA_006861	11:6772276|6802586	ENSSSCG00000009326,	KATNAL1	25	0	-6.247855449	0.010728319
circRNA_004473	5:77642285|77643213	ENSSSCG00000000800,	PUS7L	25	0	-6.247855449	0.010728319
circRNA_004250	4:37102480|37106561	ENSSSCG00000023873,	AZIN1	22.5	0	-6.248565954	0.010767734
circRNA_006904	12:4644826|4649314	ENSSSCG00000029343,	MFSD11	0	12.5	6.294687416	0.010781281
circRNA_000731	14:7879060|8335138	n/a	_	0	30	6.447298616	0.010846317
circRNA_008978	14:148266744|148289403	n/a	_	21	0	-6.244632333	0.010873737
circRNA_010726	5:106543058|106572931	n/a	_	64.5	0	-6.441430992	0.010898046
circRNA_014006	14:50023889|50034842	ENSSSCG00000009994,	MTMR3	25.75	0	-6.226271453	0.011132277
circRNA_000471	12:26840115|26845587	n/a	_	26	0	-6.225364538	0.011149385
circRNA_006558	X:19232020|19243457	ENSSSCG00000012165,	MBTPS2	0	24	6.422465898	0.011196444

circRNA_012913	2:30477465|30481157	ENSSSCG00000013314,	QSER1	25	0	-6.211358372	0.011276032
circRNA_008433	1:204783996|204798169	ENSSSCG00000005052,	WDHD1	24.75	0	-6.205404478	0.011375641
circRNA_015085	13:126801546|126818519	ENSSSCG00000025738,	-	0	22.25	6.402955833	0.011478407
circRNA_002848	10:61381029|61398475	n/a	_	0	23	6.392916509	0.011625896
circRNA_001314	2:101141685|101157198	n/a	_	0	34.75	6.391924351	0.011640561
circRNA_011261	13:140189867|140195362	n/a	_	21.5	0	-6.18101931	0.01191468
circRNA_012471	12:16977581|16979444	ENSSSCG00000017310,	KANSL1	21.5	0	-6.177931035	0.011942464
circRNA_001005	15:120872321|120884747	ENSSSCG00000016125,	INO80D	21.5	0	-6.177931035	0.011942464
circRNA_007632	4:32658673|32699400	ENSSSCG00000006035,	ANGPT1	21.75	0	-6.179399759	0.011950099
circRNA_009116	16:57092936|57121075	ENSSSCG00000017000,	RANBP17	21	0	-6.176448175	0.012049491
circRNA_013313	7:40044112|40050237	n/a	_	0	18	6.355283493	0.012193573
circRNA_001167	18:18661914|18690307	ENSSSCG00000016549,	MKLN1	20.5	0	-6.160437981	0.012355343
circRNA_015078	11:5210653|5233354	ENSSSCG00000009319,	PAN3	0	20	6.340966639	0.012415787
circRNA_015075	10:9667997|9674820	ENSSSCG00000027207,	-	0	20	6.340966639	0.012415787
circRNA_000237	1:270329795|270347874	ENSSSCG00000005388,	INVS	224.25	42.25	-1.674023224	0.012492522
circRNA_013984	1:113957632|113959372	ENSSSCG00000004528,	MBD2	19.5	0	-6.149254337	0.012530247
circRNA_001337	2:122033113|122041690	ENSSSCG00000014209,	EPB41L4A	0	39.25	6.328922209	0.012605452
circRNA_014934	4:83569528|83597759	n/a	_	0	16	6.311829065	0.012878943
circRNA_012132	GL895853.2:76998|83937	ENSSSCG00000010599,	-	0	20.25	6.311531063	0.012883756
circRNA_010165	1:16356085|16357950	ENSSSCG00000021273,	-	22.75	0	-6.123743874	0.012905398
circRNA_006015	2:91169756|91230855	ENSSSCG00000014126,	MSH3	18.75	0	-6.124697371	0.01302971
circRNA_009963	9:46271320|46282234	ENSSSCG00000015052,	USP28	0	21	6.300032552	0.013070681
circRNA_006502	9:120426043|120441453	ENSSSCG00000015455,	CUL1	0	21	6.300032552	0.013070681
circRNA_014531	8:103708394|103712082	n/a	_	0	21	6.300032552	0.013070681
circRNA_014385	3:45554717|45556311	ENSSSCG00000008098,	POLR1B	0	21	6.300032552	0.013070681
circRNA_004010	2:128801546|128820597	n/a	_	20	0	-6.116888693	0.013077324
circRNA_006913	12:14691671|14701912	ENSSSCG00000017277,	PECAM1	20	0	-6.116888693	0.013077324
circRNA_005571	10:76253466|76306111	ENSSSCG00000011166,	ZMYND11	20	0	-6.116888693	0.013077324
circRNA_014007	14:95077720|95260651	n/a	_	23.75	0	-6.111845898	0.013158689
circRNA_007054	13:132982287|132987238	ENSSSCG00000011794,	SENP2	0	22.75	6.29355147	0.013177074
circRNA_000704	13:205921704|205921996	ENSSSCG00000029392,	-	41.25	0	-6.290590951	0.013185073
circRNA_002888	11:13305769|13335221	ENSSSCG00000009362,	TRPC4	0	25.25	6.283698984	0.013340253
circRNA_011762	4:40343834|40354146	n/a	_	23.25	0	-6.095968357	0.013487898
circRNA_014506	7:97312228|97326586	ENSSSCG00000002287,	MPP5	0	19.5	6.272136462	0.013533989
circRNA_005678	13:72554931|72556469	n/a	_	0	19.5	6.272136462	0.013533989
circRNA_002504	1:60892775|60900407	ENSSSCG00000004293,	SNX14	0	26	6.264699491	0.013659884
circRNA_009803	7:109768787|109825592	n/a	_	22.5	0	-6.079461332	0.013675985
circRNA_005684	13:74656936|74800063	ENSSSCG00000011575,	ATG7	21.25	0	-6.086541408	0.013684037
circRNA_003475	14:143705043|143706167	ENSSSCG00000010706,	-	0	16	6.25683992	0.013794036
circRNA_006738	1:202793118|202798180	ENSSSCG00000005041,	FERMT2	0	28.25	6.254113184	0.013840842
circRNA_010478	15:73542910|73573786	ENSSSCG00000015882,	BAZ2B	0	20.25	6.249075705	0.01392768
circRNA_010781	7:10297937|10442822	n/a	_	0	20.25	6.249075705	0.01392768
circRNA_006782	1:282195331|282195982	ENSSSCG00000005455,	SVEP1	0	20.25	6.249075705	0.01392768
circRNA_006635	1:60893530|60924967	ENSSSCG00000004293,	SNX14	0	20.25	6.249075705	0.01392768
circRNA_000612	13:86966559|86985258	ENSSSCG00000026746,	CEP70	22.5	0	-6.052618187	0.014182283
circRNA_014039	11:75129058|75132336	ENSSSCG00000022112,	DOCK9	22.5	0	-6.052618187	0.014182283
circRNA_008556	10:56077005|56083293	ENSSSCG00000011074,	ARHGAP21	47.75	0	-6.224977187	0.014303163
circRNA_009141	17:36148045|36154601	ENSSSCG00000007142,	PANK2	0	31	6.224991853	0.014349392
circRNA_014682	2:90660850|90673745	ENSSSCG00000014119,	-	36.5	0	-6.215654096	0.014468503
circRNA_014923	15:73179254|73179527	n/a	_	0	20.25	6.215434234	0.014519787
circRNA_014905	1:15928649|15948518	n/a	_	0	14	6.215172579	0.014524476
circRNA_014938	5:51567416|51742379	n/a	_	0	14	6.215172579	0.014524476

circRNA_002313	9:150621076|150667131	n/a	_	0	14	6.215172579	0.014524476
circRNA_003040	12:49362946|49370193	ENSSSCG00000017812,	VPS53	0	16	6.215040299	0.014526847
circRNA_009967	9:50817859|50834603	ENSSSCG00000030053,	ARCN1	48.25	0	-6.209478111	0.014578939
circRNA_013573	X:35456706|35478544	ENSSSCG00000030800,	CXorf59	0	14.25	6.211096007	0.014597705
circRNA_014928	2:119947335|120001743	n/a	_	0	14.25	6.211096007	0.014597705
circRNA_014924	16:23583623|23598577	ENSSSCG00000016843,	C5orf42	0	14	6.206617896	0.014678515
circRNA_013126	4:117099716|117115606	ENSSSCG00000006767,	MAGI3	0	18.25	6.206488962	0.014680848
circRNA_006123	4:12615332|12643641	n/a	_	0	18.25	6.206488962	0.014680848
circRNA_012138	GL896504.1:24299|55460	ENSSSCG00000027115,	-	0	14.25	6.2024949	0.014753259
circRNA_000311	10:33080414|33081898	ENSSSCG00000010952,	ZCCHC6	19.75	0	-6.024807772	0.014870885
circRNA_002965	12:15273975|15275808	ENSSSCG00000024813,	MAP3K3	19.75	0	-6.021373355	0.014908885
circRNA_006408	8:74346358|74456314	n/a	_	0	19	6.193073623	0.014925289
circRNA_008647	12:18731949|18746173	ENSSSCG00000017346,	EFTUD2	20.75	0	-6.01783746	0.014971476
circRNA_009181	18:14762483|14814873	n/a	_	0	26.25	6.185981106	0.015055937
circRNA_004991	8:112040320|112052306	ENSSSCG00000009106,	PDE5A	0	18.25	6.180263118	0.015161985
circRNA_011450	15:138663710|138681538	ENSSSCG00000016223,	ACSL3	0	27.25	6.178468965	0.015195392
circRNA_014470	6:54760479|54967024	n/a	_	0	16.75	6.173568047	0.015286973
circRNA_015077	10:43878947|43927930	n/a	_	0	16	6.170684808	0.015341073
circRNA_015107	JH118995.1:394|812	n/a	_	0	16	6.170684808	0.015341073
circRNA_011822	5:57093473|57093836	ENSSSCG00000000588,	-	0	23.75	6.161014946	0.015523722
circRNA_014396	3:74760760|74764088	ENSSSCG00000022708,	-	0	17.5	6.160532281	0.015532888
circRNA_003910	2:48859588|48876280	ENSSSCG00000021170,	FAR1	0	16.25	6.147977927	0.015772942
circRNA_002172	9:2455541|2612054	n/a	_	342.75	486	1.114843646	0.015799182
circRNA_005462	1:146253719|146463424	n/a	_	0	25.75	6.14538404	0.015822935
circRNA_003170	13:91070338|91074476	ENSSSCG00000011679,	ATR	18	0	-5.970400862	0.016052632
circRNA_011835	5:86522733|86528802	ENSSSCG00000023236,	-	16.75	0	-5.975381135	0.016052651
circRNA_014019	4:137104657|137136291	n/a	_	16.75	0	-5.975381135	0.016052651
circRNA_010033	GL892277.2:75154|83123	ENSSSCG00000004124,	-	21.5	0	-5.968722537	0.016144394
circRNA_001255	2:59237034|59274491	n/a	_	0	17.75	6.127996076	0.016161616
circRNA_009644	6:69159389|69166153	ENSSSCG00000003462,	DDI2	0	17.75	6.127996076	0.016161616
circRNA_014425	4:72410011|72458585	n/a	_	0	16	6.126429945	0.016192425
circRNA_014025	7:35546228|35546770	ENSSSCG00000001533,	TAF11	18.5	0	-5.959805213	0.016338739
circRNA_014099	1:34740831|34759948	n/a	_	0	23.25	6.11893164	0.016340637
circRNA_009564	5:70588009|70663997	ENSSSCG00000000755,	ERC1	17.75	0	-5.957584014	0.016341288
circRNA_007002	13:72865581|72879916	ENSSSCG00000011542,	-	18.75	0	-5.954018139	0.01640823
circRNA_005109	9:43033759|43050877	n/a	_	20.25	0	-5.945567454	0.016491481
circRNA_014241	14:42332310|42352788	ENSSSCG00000009890,	MAPKAPK5	0	24.25	6.110701728	0.016504653
circRNA_007156	14:83858791|83860077	ENSSSCG00000010319,	SAMD8	0	24.25	6.110701728	0.016504653
circRNA_002556	1:116900620|116917868	ENSSSCG00000004538,	WDR7	0	18.5	6.104290648	0.016633401
circRNA_003104	13:37111091|37113036	ENSSSCG00000011425,	-	18	0	-5.939009113	0.0167571
circRNA_015071	1:25518797|25530074	ENSSSCG00000004140,	VTA1	0	14	6.093346647	0.016855178
circRNA_005399	1:4635856|4658081	ENSSSCG00000004027,	PDE10A	0	14	6.093346647	0.016855178
circRNA_001734	5:70319577|70339367	ENSSSCG00000000753,	WNK1	0	14	6.093346647	0.016855178
circRNA_005132	9:78693459|78702418	ENSSSCG00000015310,	AKAP9	0	20.75	6.091451752	0.016893835
circRNA_014050	5:35894045|35903325	ENSSSCG00000000488,	MDM2	20.25	0	-5.925871476	0.016894722
circRNA_014300	15:116138978|116154892	ENSSSCG00000026940,	CASP10	0	14.5	6.090616786	0.016910893
circRNA_008576	11:2098052|2101796	ENSSSCG00000009290,	-	0	12.25	6.088561974	0.016952935
circRNA_003209	13:142562710|142600017	ENSSSCG00000011833,	DLG1	37	0	-6.084913287	0.016966926
circRNA_007736	5:35903030|35916494	ENSSSCG00000000488,	MDM2	0	12	6.083230176	0.017062444
circRNA_014208	13:90760724|90767547	ENSSSCG00000011677,	GK5	0	21.75	6.082907479	0.017069092
circRNA_005667	13:37977080|37988133	n/a	_	78.75	6	-3.567116685	0.017410899
circRNA_006640	1:62359384|62387755	ENSSSCG00000025140,	ZNF292	20.5	0	-5.89679587	0.017532058

circRNA_014568	9:117749747|117752827	n/a	_	0	14.75	6.054749345	0.01765777
circRNA_014299	15:112469260|112476823	ENSSSCG00000016075,	SF3B1	0	14.75	6.054749345	0.01765777
circRNA_001245	2:50789562|50805720	ENSSSCG00000013402,	-	18.25	0	-5.884091743	0.017942675
circRNA_014004	14:21462664|21481453	ENSSSCG00000009714,	NEK1	18.25	0	-5.884091743	0.017942675
circRNA_010552	2:3011482|3022317	ENSSSCG00000012884,	PPP6R3	0	14	6.040653405	0.017958953
circRNA_006288	6:98799538|98801480	ENSSSCG00000021893,	ROCK1	18.5	0	-5.882527172	0.017989844
circRNA_011073	1:303998762|304017650	ENSSSCG00000005689,	FNBP1	18.5	0	-5.882527172	0.017989844
circRNA_013993	1:299933010|299949846	n/a	_	18.25	0	-5.880364142	0.01799001
circRNA_002411	X:17758636|17759302	ENSSSCG00000012163,	RPS6KA3	32.5	0	-6.028038455	0.018164456
circRNA_014560	9:88615508|88638210	ENSSSCG00000026722,	PHF14	0	22	6.015493717	0.018507499
circRNA_014926	18:44782112|44784505	ENSSSCG00000023445,	AVL9	0	14	6.007622228	0.018682041
circRNA_009317	2:148351052|148352296	ENSSSCG00000014376,	HARS2	0	14	6.007622228	0.018682041
circRNA_013197	5:88358137|88439154	n/a	_	0	12	6.006710876	0.01870234
circRNA_007997	8:33308376|33317260	ENSSSCG00000022168,	APBB2	0	18.75	6.004994319	0.018740625
circRNA_011508	17:12515432|12529955	ENSSSCG00000007023,	KAT6A	0	19.75	5.995404807	0.018955742
circRNA_014192	13:35035493|35038004	ENSSSCG00000026739,	-	0	19.75	5.995404807	0.018955742
circRNA_009931	9:29012165|29015206	ENSSSCG00000024933,	SLC36A4	36.25	0	-5.986849074	0.019076278
circRNA_014201	13:56459055|56462244	ENSSSCG00000011512,	-	0	21.5	5.98440999	0.019100831
circRNA_014307	15:156396928|156442590	n/a	_	0	14	5.987356347	0.019137918
circRNA_014380	3:12224076|12395931	n/a	_	0	14	5.987356347	0.019137918
circRNA_003286	14:1158578|1159827	ENSSSCG00000028033,	-	0	17	5.954506501	0.019157924
circRNA_014223	13:149286710|149288041	ENSSSCG00000011887,	-	0	17	5.954506501	0.019157924
circRNA_014931	3:107521846|107609161	n/a	_	0	12.25	5.978278048	0.019345202
circRNA_001485	3:107521846|107609954	n/a	_	84.25	173.75	1.910988158	0.019352737
circRNA_014204	13:83142279|83202617	ENSSSCG00000011645,	CEP63	0	20.5	5.977248576	0.019368829
circRNA_008756	13:55492221|55503168	ENSSSCG00000011504,	EOGT	0	18.5	5.954392594	0.019453323
circRNA_010519	17:5409813|5418002	ENSSSCG00000006984,	-	0	18.5	5.954392594	0.019453323
circRNA_014048	3:38764978|38769050	ENSSSCG00000007930,	MGRN1	18	0	-5.816187337	0.019540613
circRNA_009289	2:119260427|119296023	ENSSSCG00000014195,	MAN2A1	0	18	5.968551899	0.019569406
circRNA_005211	GL892277.2:80612|83123	ENSSSCG00000004124,	-	0	21	5.957352351	0.019571835
circRNA_014408	3:122012247|122024159	ENSSSCG00000008591,	-	0	18	5.936267749	0.019613242
circRNA_008073	9:250376|261322	ENSSSCG00000014579,	-	70.75	6	-3.525574251	0.019707041
circRNA_009622	6:26462255|26462658	ENSSSCG00000025417,	BBS2	0	16.5	5.960733891	0.019751232
circRNA_012829	17:13090591|13092479	ENSSSCG00000007030,	IKBKB	0	14	5.960373956	0.019759638
circRNA_014032	GL896250.1:9584|16183	ENSSSCG00000027508,	-	15	0	-5.811987613	0.019810609
circRNA_011875	6:64311271|64321438	ENSSSCG00000003398,	-	0	14.25	5.958193352	0.019810628
circRNA_014444	5:33927679|33928557	ENSSSCG00000000477,	HELB	0	14.25	5.958193352	0.019810628
circRNA_014460	5:87266266|87308493	ENSSSCG00000000873,	ANO4	0	14.25	5.958193352	0.019810628
circRNA_009334	3:19646832|19652677	ENSSSCG00000007815,	GTF3C1	34	0	-5.953995468	0.019831356
circRNA_010534	18:1417740|1420536	ENSSSCG00000026652,	-	16.75	0	-5.808116429	0.019836163
circRNA_014031	9:144775236|144798978	n/a	_	16.75	0	-5.808116429	0.019836163
circRNA_010028	9:143383683|143393975	ENSSSCG00000015588,	ANGEL2	0	18.25	5.952530901	0.019943562
circRNA_014536	8:122432854|122435684	n/a	_	0	18.25	5.952530901	0.019943562
circRNA_000666	13:143037257|143052323	n/a	_	0	18.25	5.952530901	0.019943562
circRNA_006938	12:38732180|38801846	ENSSSCG00000017676,	BCAS3	0	19	5.946256073	0.020004641
circRNA_008682	12:48582940|48591386	ENSSSCG00000017799,	CPD	34	0	-5.94384694	0.020069696
circRNA_013009	3:56863189|56865978	ENSSSCG00000008177,	-	0	12	5.94718295	0.020069812
circRNA_015092	3:72409632|72422934	n/a	_	0	12	5.94718295	0.020069812
circRNA_015109	X:101973310|102002622	ENSSSCG00000012557,	NUP62CL	0	12	5.94718295	0.020069812
circRNA_013994	10:48609499|48609985	ENSSSCG00000011035,	-	16.25	0	-5.799219138	0.020080582
circRNA_014234	14:30886263|30887464	ENSSSCG00000009765,	DNAH10	0	12.5	5.943766432	0.020150824
circRNA_014114	1:128019978|128030115	ENSSSCG00000004602,	TEX9	0	12.5	5.943766432	0.020150824

circRNA_014430	4:75094374|75105835	ENSSSCG00000006219,	MTFR1	0	12.5	5.943766432	0.020150824
circRNA_014418	4:45545828|45567734	ENSSSCG00000006100,	-	0	12.5	5.943766432	0.020150824
circRNA_008173	9:134281948|134292300	ENSSSCG00000015537,	XPR1	0	12.5	5.943766432	0.020150824
circRNA_009980	9:77414305|77469954	ENSSSCG00000015307,	-	0	12.5	5.943766432	0.020150824
circRNA_014051	6:11749560|11759367	n/a	_	18	0	-5.783569523	0.020274248
circRNA_014034	1:16395773|16402143	ENSSSCG00000004082,	-	18	0	-5.783569523	0.020274248
circRNA_001456	3:84809485|84822563	n/a	_	0	10	5.934588593	0.020369829
circRNA_002611	1:148284301|148292614	ENSSSCG00000004792,	FAM98B	0	16.5	5.923937137	0.020626542
circRNA_010929	X:14612680|14670417	ENSSSCG00000012150,	REPS2	0	16.5	5.923937137	0.020626542
circRNA_012735	15:86509217|86510646	ENSSSCG00000015943,	GORASP2	0	17.5	5.915973661	0.020638676
circRNA_008417	1:188117293|188122298	ENSSSCG00000004979,	-	35.25	0	-5.915272651	0.020753931
circRNA_014889	9:115756518|115761463	ENSSSCG00000026110,	SRPK2	32.25	0	-5.911324891	0.020850008
circRNA_007714	5:4603986|4611101	ENSSSCG00000000068,	EP300	0	12.75	5.912829832	0.020897178
circRNA_014359	2:50769530|50805720	ENSSSCG00000013402,	-	0	12.75	5.912829832	0.020897178
circRNA_008523	10:33050271|33057782	ENSSSCG00000010952,	ZCCHC6	18	0	-5.758927934	0.020906564
circRNA_000895	15:26926656|26939836	ENSSSCG00000015714,	CCDC93	16.25	0	-5.766654962	0.020922912
circRNA_009138	17:29448874|29449181	ENSSSCG00000007085,	DSTN	28	0	-5.90723099	0.020950041
circRNA_014061	GL893259.1:34612|56752	ENSSSCG00000026158,	RAD18	18	0	-5.754862304	0.020963995
circRNA_014951	9:129701005|129724374	ENSSSCG00000015509,	-	0	12	5.909636345	0.020975548
circRNA_014915	13:33957923|33961723	n/a	_	0	12	5.909636345	0.020975548
circRNA_005171	9:128949520|128949783	ENSSSCG00000015507,	-	0	12	5.909636345	0.020975548
circRNA_014946	8:40455259|40459063	ENSSSCG00000008826,	-	0	12	5.909636345	0.020975548
circRNA_010187	1:75082709|75101925	ENSSSCG00000004351,	USP45	0	16.75	5.906998223	0.021040478
circRNA_014453	5:61454150|61467100	ENSSSCG00000000612,	ATF7IP	0	16.75	5.906998223	0.021040478
circRNA_014530	8:89346255|89366855	ENSSSCG00000009047,	SMARCA5	0	16.75	5.906998223	0.021040478
circRNA_004982	8:102838308|103138543	n/a	_	0	20.5	5.893283208	0.021129466
circRNA_003768	17:39462650|39463937	ENSSSCG00000007203,	-	15.25	0	-5.755015067	0.021176751
circRNA_001821	6:69714724|69730166	ENSSSCG00000024794,	FBXO42	15.25	0	-5.755015067	0.021176751
circRNA_005251	GL894597.1:48506|49753	n/a	_	15.25	0	-5.755015067	0.021176751
circRNA_008893	14:34693433|34697216	ENSSSCG00000009834,	ATXN2	15.25	0	-5.755015067	0.021176751
circRNA_014718	6:81553639|81580900	ENSSSCG00000003596,	SNRNP40	27.75	0	-5.895929687	0.021228309
circRNA_014710	5:43354518|43380786	n/a	_	27.75	0	-5.895929687	0.021228309
circRNA_014345	18:36472459|36535413	ENSSSCG00000025602,	-	0	15.5	5.871669286	0.021302307
circRNA_014188	13:14307735|14340843	n/a	_	0	15.5	5.871669286	0.021302307
circRNA_014304	15:133579638|133580228	ENSSSCG00000016194,	USP37	0	15.5	5.871669286	0.021302307
circRNA_014510	7:105823028|105833951	n/a	_	0	15.5	5.871669286	0.021302307
circRNA_012722	15:62337487|62342162	ENSSSCG00000015849,	SARAF	0	15.5	5.871669286	0.021302307
circRNA_002603	1:143285207|143291819	ENSSSCG00000004717,	UBR1	0	15.5	5.871669286	0.021302307
circRNA_014022	6:145705905|145707525	ENSSSCG00000003844,	TTC4	16	0	-5.740523366	0.021487603
circRNA_006010	2:87837872|87846078	ENSSSCG00000014097,	PDE8B	16	0	-5.740523366	0.021487603
circRNA_009718	6:153309526|153309840	ENSSSCG00000003917,	TESK2	16	0	-5.740523366	0.021487603
circRNA_014003	13:210787792|210792658	ENSSSCG00000012062,	TTC3	16	0	-5.740523366	0.021487603
circRNA_014140	1:247961314|247973138	ENSSSCG00000005243,	PGM5	0	16	5.874135427	0.021626367
circRNA_014410	3:133994994|134000202	ENSSSCG00000025483,	-	0	16	5.874135427	0.021626367
circRNA_013100	4:68353630|68360347	ENSSSCG00000006184,	TERF1	0	16.5	5.856394745	0.02169995
circRNA_002501	1:57105762|57195641	ENSSSCG00000004276,	SMAP1	0	16.5	5.856394745	0.02169995
circRNA_013063	3:135328996|135332107	ENSSSCG00000008642,	ASAP2	0	16.5	5.856394745	0.02169995
circRNA_006157	4:90623622|90629143	ENSSSCG00000006303,	-	37	0	-5.874479793	0.021765115
circRNA_009574	5:79772211|79785608	ENSSSCG00000000806,	SCAF11	0	15	5.876433961	0.021805305
circRNA_014428	4:73714673|73732960	ENSSSCG00000006202,	-	0	15	5.876433961	0.021805305
circRNA_010766	6:136000651|136018579	ENSSSCG00000025672,	RAVER2	0	15	5.876433961	0.021805305
circRNA_010144	X:101651839|101668730	ENSSSCG00000022993,	TBC1D8B	0	15	5.876433961	0.021805305

circRNA_014869	7:109866218|109874274	n/a	_	31.5	0	-5.86943177	0.021893111
circRNA_009983	9:78881650|78907286	ENSSSCG00000015313,	KRIT1	31.5	0	-5.86943177	0.021893111
circRNA_014292	15:86498639|86504888	ENSSSCG00000015943,	GORASP2	0	13.5	5.867436741	0.022034908
circRNA_007930	7:73100247|73111840	ENSSSCG00000001970,	HEATR5A	0	13.5	5.867436741	0.022034908
circRNA_007467	2:80452934|80456550	ENSSSCG00000014020,	CANX	0	11.25	5.864467762	0.022111124
circRNA_004678	6:143689203|143689360	ENSSSCG00000003839,	PLPP3	0	11.25	5.864467762	0.022111124
circRNA_014918	14:29911026|29913773	ENSSSCG00000009758,	DHX37	0	12	5.855530436	0.022341907
circRNA_011214	13:35734591|35743359	ENSSSCG00000011394,	RBM6	0	12	5.855530436	0.022341907
circRNA_014685	2:98312972|98326796	ENSSSCG00000014148,	TMEM161B	24.75	0	-5.851529456	0.022352202
circRNA_005027	8:144223745|144239660	ENSSSCG00000009233,	GPAT3	16.5	0	-5.708849223	0.022353335
circRNA_013989	1:269689085|269720498	n/a	_	16.5	0	-5.708849223	0.022353335
circRNA_012053	9:28144692|28147997	n/a	_	16.75	0	-5.707306423	0.022407781
circRNA_015097	5:18745419|18745819	ENSSSCG00000021278,	-	0	10	5.840055688	0.022746346
circRNA_003212	13:143278146|143282223	ENSSSCG00000011845,	PCYT1A	0	10	5.840055688	0.022746346
circRNA_014756	X:70664081|70685159	ENSSSCG00000012434,	-	28	0	-5.836120098	0.022753883
circRNA_002686	1:218683998|218686093	ENSSSCG00000005127,	CAAP1	32	0	-5.829039183	0.022940507
circRNA_008582	11:11405097|11406962	n/a	_	0	13.5	5.825656253	0.023128252
circRNA_002522	1:80356327|80366530	ENSSSCG00000004364,	HACE1	0	13.5	5.825656253	0.023128252
circRNA_007745	5:46852469|46856993	ENSSSCG00000000534,	IPO8	0	14.5	5.814277143	0.023219847
circRNA_003934	2:80548536|80553596	ENSSSCG00000014023,	RUFY1	0	14.5	5.814277143	0.023219847
circRNA_005549	10:31143161|31153744	n/a	_	0	14.5	5.814277143	0.023219847
circRNA_014288	15:83538630|83594895	n/a	_	0	14.5	5.814277143	0.023219847
circRNA_002467	1:16415160|16473752	ENSSSCG00000004082,	-	0	16	5.818601681	0.023252549
circRNA_003169	13:90954665|90963935	ENSSSCG00000011678,	XRN1	33.75	0	-5.816601613	0.023271451
circRNA_010951	1:14083797|14085261	ENSSSCG00000004070,	SCAF8	33.75	0	-5.816601613	0.023271451
circRNA_014538	8:140621348|140624242	ENSSSCG00000009222,	SPARCL1	0	13	5.799185583	0.023381769
circRNA_011680	3:46226093|46230867	ENSSSCG00000008103,	-	0	12	5.81593249	0.023389213
circRNA_014606	1:16169680|16183454	ENSSSCG00000004081,	-	30.25	0	-5.805460553	0.023571323
circRNA_009651	6:78476142|78485300	ENSSSCG00000024927,	STX12	0	14	5.788248078	0.023632672
circRNA_014209	13:90903716|90952792	ENSSSCG00000011678,	XRN1	0	14	5.788248078	0.023632672
circRNA_012576	13:117571688|117581247	ENSSSCG00000030467,	PHC3	30.5	0	-5.802048067	0.023663822
circRNA_002267	9:101227171|101245683	ENSSSCG00000015386,	FAM126A	28.5	0	-5.795169587	0.023851211
circRNA_004713	7:10291894|10310057	ENSSSCG00000001056,	RANBP9	15.75	0	-5.647990031	0.023895913
circRNA_005907	16:71618182|71624219	ENSSSCG00000017052,	ADAM19	15.75	0	-5.647990031	0.023895913
circRNA_007415	18:59685146|59697010	n/a	_	0	10	5.796632448	0.023914589
circRNA_014949	9:24125972|24168454	n/a	_	0	10	5.796632448	0.023914589
circRNA_014941	6:143739937|143741629	n/a	_	0	10	5.796632448	0.023914589
circRNA_005940	18:10898787|10902471	ENSSSCG00000016510,	UBN2	0	15	5.774099722	0.024021046
circRNA_002832	10:51236326|51261622	ENSSSCG00000011047,	FAM171A1	0	15	5.774099722	0.024021046
circRNA_007364	17:51437349|51437753	n/a	_	0	15	5.774099722	0.024021046
circRNA_000314	10:33646093|33649475	ENSSSCG00000010957,	-	0	15	5.774099722	0.024021046
circRNA_012157	X:70438343|70498760	ENSSSCG00000012434,	-	0	15	5.774099722	0.024021046
circRNA_014456	5:73046399|73047734	ENSSSCG00000000778,	CPNE8	0	14.5	5.790885462	0.024023394
circRNA_005108	9:43030233|43050877	n/a	_	21.75	0	-5.781637734	0.024223524
circRNA_013997	12:22572340|22574310	ENSSSCG00000017477,	CASC3	14.5	0	-5.636109768	0.024405902
circRNA_015021	18:59442183|59557766	n/a	_	32	0	-5.76772508	0.024611426
circRNA_004465	5:70819577|70847537	n/a	_	0	12	5.771134932	0.024623977
circRNA_014335	17:49887473|49948154	n/a	_	0	12	5.771134932	0.024623977
circRNA_014353	2:27609998|27614983	n/a	_	0	12	5.771134932	0.024623977
circRNA_009010	15:60961638|60963094	n/a	_	0	12	5.771134932	0.024623977
circRNA_014211	13:91226436|91260862	ENSSSCG00000011681,	TRPC1	0	12	5.771134932	0.024623977
circRNA_014340	18:10874962|10888869	ENSSSCG00000016510,	UBN2	0	16	5.75216772	0.024684489

circRNA_008602	11:25517563|25532653	ENSSSCG00000009431,	DGKH	0	16	5.75216772	0.024684489
circRNA_014016	2:132248051|132259847	ENSSSCG00000014239,	CEP120	14	0	-5.626813553	0.024696708
circRNA_014035	1:39747136|39754248	ENSSSCG00000004218,	RSPO3	15.75	0	-5.617764272	0.024697227
circRNA_014895	JH118921.1:16802|29587	n/a	_	27	0	-5.76434444	0.02470647
circRNA_001826	6:77623182|77623775	n/a	_	0	10	5.765082847	0.024794948
circRNA_015096	4:96074696|96087170	ENSSSCG00000022220,	-	0	10	5.765082847	0.024794948
circRNA_006620	1:28460560|28531105	n/a	_	0	10	5.765082847	0.024794948
circRNA_014917	13:144384384|144403607	ENSSSCG00000026753,	-	0	8	5.76426425	0.024818151
circRNA_005874	15:128043602|128074514	ENSSSCG00000016166,	-	0	8	5.76426425	0.024818151
circRNA_014944	7:100363470|100617788	n/a	_	0	8	5.76426425	0.024818151
circRNA_012388	10:56167668|56208948	ENSSSCG00000011075,	KIAA1217	28	0	-5.759866525	0.024832842
circRNA_014466	6:18436857|18441054	ENSSSCG00000002799,	CNOT1	0	10.5	5.760564272	0.024923252
circRNA_014169	11:9798241|9881009	n/a	_	0	10.5	5.760564272	0.024923252
circRNA_005933	17:57170779|57179361	n/a	_	0	10.5	5.760564272	0.024923252
circRNA_014275	15:34947373|34955787	ENSSSCG00000015731,	-	0	10.5	5.760564272	0.024923252
circRNA_009097	16:36664765|36698971	ENSSSCG00000016911,	SKIV2L2	20.75	0	-5.749641525	0.02512345
circRNA_000159	1:180708440|180715180	ENSSSCG00000004927,	CLPX	13.75	0	-5.603230172	0.025293145
circRNA_003239	13:166927031|166930220	ENSSSCG00000011953,	ZBTB11	23	0	-5.739055815	0.025427329
circRNA_010824	8:19208010|19209747	ENSSSCG00000023001,	CCDC149	23.5	0	-5.736713825	0.025494976
circRNA_004238	4:20042957|20052708	ENSSSCG00000005998,	DEPTOR	21.75	0	-5.733105111	0.02559951
circRNA_004445	5:54839918|54843310	ENSSSCG00000000571,	ABCC9	22.25	0	-5.728345385	0.025737938
circRNA_014776	12:32299469|32304905	n/a	_	25.25	0	-5.725936318	0.025808241
circRNA_009169	18:5609233|5634518	n/a	_	0	11.5	5.716954312	0.025826581
circRNA_013287	6:148034940|148044395	ENSSSCG00000003870,	NRDC	0	11.5	5.716954312	0.025826581
circRNA_014877	8:57805777|57808175	ENSSSCG00000008900,	SRP72	27	0	-5.724295051	0.02585623
circRNA_012345	10:17127705|17204667	n/a	_	21.5	0	-5.722314254	0.025914248
circRNA_007349	17:36419116|36449044	ENSSSCG00000007153,	ATRN	20.25	0	-5.718937004	0.02601342
circRNA_009914	9:6833607|6873499	n/a	_	27.5	0	-5.717676307	0.026050522
circRNA_014547	9:31820568|31829919	ENSSSCG00000014969,	-	0	13	5.721492231	0.026051577
circRNA_014294	15:86637917|86639123	ENSSSCG00000015944,	TLK1	0	13	5.721492231	0.026051577
circRNA_014276	15:37967726|37969934	ENSSSCG00000023419,	-	0	13	5.721492231	0.026051577
circRNA_002538	1:94556573|94577735	ENSSSCG00000004460,	IBTK	22.25	0	-5.716986118	0.026070852
circRNA_007255	15:85045931|85046450	ENSSSCG00000015933,	FASTKD1	28.25	0	-5.714080018	0.026156604
circRNA_000792	14:62473110|62480280	ENSSSCG00000010169,	SIPA1L2	19.5	0	-5.710388551	0.026265873
circRNA_001266	2:70193426|70194875	ENSSSCG00000030900,	LDLR	19.5	0	-5.710388551	0.026265873
circRNA_014556	9:79015624|79041213	ENSSSCG00000015314,	ANKIB1	0	12.5	5.701004918	0.026275261
circRNA_014467	6:38271273|38295097	ENSSSCG00000025152,	CEP89	0	12.5	5.701004918	0.026275261
circRNA_001095	17:29783317|29787734	ENSSSCG00000007088,	SNX5	0	12.5	5.701004918	0.026275261
circRNA_009717	6:150809988|150895226	ENSSSCG00000003881,	SPATA6	0	12.5	5.701004918	0.026275261
circRNA_006999	13:60155936|60158313	n/a	_	0	12.5	5.701004918	0.026275261
circRNA_007281	15:121417853|121432247	ENSSSCG00000016131,	ADAM23	30.25	0	-5.708794966	0.026313162
circRNA_004501	5:94425846|94439340	ENSSSCG00000000912,	EEA1	30.25	0	-5.708794966	0.026313162
circRNA_010366	13:42364695|42392300	ENSSSCG00000024201,	FAM208A	0	10.5	5.712292681	0.026329309
circRNA_014274	15:28884310|28893785	ENSSSCG00000015721,	ERCC3	0	10.5	5.712292681	0.026329309
circRNA_004813	7:89101045|89134969	n/a	_	29.75	0	-5.706047766	0.026394853
circRNA_002115	8:88098100|88185045	n/a	_	65	112.5	1.516345847	0.026408758
circRNA_014125	1:179091950|179102403	ENSSSCG00000004907,	CCBE1	0	13.5	5.692040042	0.0264991
circRNA_014505	7:94524184|94525927	ENSSSCG00000022453,	-	0	13.5	5.692040042	0.0264991
circRNA_014489	7:37126123|37133150	ENSSSCG00000001563,	STK38	0	13.5	5.692040042	0.0264991
circRNA_014264	14:116579352|116611789	ENSSSCG00000010494,	SORBS1	0	13.5	5.692040042	0.0264991
circRNA_013983	1:107795204|107835060	ENSSSCG00000004506,	CTIF	14.25	0	-5.556762171	0.026638926
circRNA_007946	7:97833510|97834004	ENSSSCG00000002298,	ZFYVE26	0	9	5.700720936	0.026676141

circRNA_014513	7:128613568|128613730	n/a	_	0	9	5.700720936	0.026676141
circRNA_014613	1:188117293|188136843	ENSSSCG00000004979,	-	23.5	0	-5.695069118	0.026723449
circRNA_014846	5:106543058|106579236	n/a	_	24.75	0	-5.691486162	0.026831431
circRNA_014786	13:130101830|130118975	ENSSSCG00000011772,	-	24.75	0	-5.691486162	0.026831431
circRNA_014092	1:14096258|14110711	ENSSSCG00000004070,	SCAF8	0	9	5.681971372	0.02701792
circRNA_005457	1:143267765|143285303	ENSSSCG00000004717,	UBR1	0	9	5.681971372	0.02701792
circRNA_008340	1:93560104|93566176	ENSSSCG00000025745,	-	0	11.5	5.67602536	0.027085601
circRNA_014478	6:88257154|88264397	ENSSSCG00000003654,	MACF1	0	11.5	5.67602536	0.027085601
circRNA_014529	8:86035739|86042328	ENSSSCG00000009029,	ARHGAP10	0	14.5	5.673018026	0.027099777
circRNA_003690	16:36914607|36926011	ENSSSCG00000030343,	SLC38A9	0	14.5	5.673018026	0.027099777
circRNA_001496	3:113999359|114020837	ENSSSCG00000008513,	BIRC6	26.75	0	-5.673365282	0.027383187
circRNA_000115	1:133338956|133348678	n/a	_	25.75	0	-5.668508271	0.027532688
circRNA_012034	9:71135|75871	n/a	_	20.75	0	-5.663501825	0.027687505
circRNA_006941	12:39721598|39726184	ENSSSCG00000017688,	GGNBP2	19.75	0	-5.663346867	0.027692309
circRNA_002649	1:195242228|195244121	ENSSSCG00000005000,	FANCM	19.75	0	-5.663346867	0.027692309
circRNA_008768	13:74656936|74736227	ENSSSCG00000011575,	ATG7	19.75	0	-5.663346867	0.027692309
circRNA_014653	14:34693427|34717605	ENSSSCG00000009834,	ATXN2	20.75	0	-5.660855189	0.027769644
circRNA_009459	4:55324656|55335142	ENSSSCG00000006137,	WWP1	20.75	0	-5.660855189	0.027769644
circRNA_000778	14:53600684|53609301	ENSSSCG00000010081,	MAPK1	19.25	0	-5.660020563	0.027795589
circRNA_001398	3:29625387|29768928	n/a	_	19.5	0	-5.656887167	0.027893175
circRNA_015088	17:5330457|5341673	ENSSSCG00000006983,	CNOT7	0	8	5.652491441	0.027976739
circRNA_004164	3:107521846|107611131	n/a	_	0	8	5.652491441	0.027976739
circRNA_000620	13:91211618|91249175	ENSSSCG00000011681,	TRPC1	0	8	5.652491441	0.027976739
circRNA_003882	2:9163330|9164458	ENSSSCG00000013073,	FADS3	23	0	-5.649052968	0.028138421
circRNA_012234	1:103929815|103946660	n/a	_	21.5	0	-5.646463892	0.028219868
circRNA_010343	12:44859641|44863025	ENSSSCG00000017745,	UTP6	21.5	0	-5.646463892	0.028219868
circRNA_006541	GL896549.1:20832|23514	ENSSSCG00000026239,	VPS35	27.75	0	-5.645827639	0.028239914
circRNA_002578	1:132344010|132367489	ENSSSCG00000004620,	MYO5A	27.75	0	-5.645827639	0.028239914
circRNA_010894	9:113302666|113320145	ENSSSCG00000015414,	FAM185A	27.25	0	-5.642835944	0.028334329
circRNA_009019	15:71720780|71727032	ENSSSCG00000015874,	ACVR1	22.5	0	-5.642028854	0.028359845
circRNA_004412	5:16681220|16694950	ENSSSCG00000028516,	LARP4	18	0	-5.641158629	0.028387379
circRNA_014736	9:255216|260033	ENSSSCG00000014579,	-	18	0	-5.641158629	0.028387379
circRNA_011984	7:134694054|134704085	n/a	_	20.5	0	-5.636384279	0.028538838
circRNA_014028	7:105387843|105396660	ENSSSCG00000002387,	GPATCH2L	12.25	0	-5.493258369	0.028682293
circRNA_004786	7:65162015|65165588	ENSSSCG00000001924,	BBS4	17.25	0	-5.631657324	0.028689461
circRNA_006554	X:14262458|14266237	ENSSSCG00000029764,	TXLNG	50.75	3	-3.785270188	0.028720924
circRNA_014801	15:117901943|117913489	ENSSSCG00000016117,	CARF	23	0	-5.630061763	0.028740453
circRNA_010442	14:78009691|78026373	ENSSSCG00000010246,	DDX50	22.5	0	-5.62658887	0.028851706
circRNA_014784	13:97274219|97281017	ENSSSCG00000025293,	HLTF	22.5	0	-5.62658887	0.028851706
circRNA_007282	15:122904851|122914434	ENSSSCG00000016147,	PIKFYVE	24.5	0	-5.622699162	0.028976739
circRNA_009273	2:90148542|90166516	ENSSSCG00000014114,	PAPD4	24.5	0	-5.622699162	0.028976739
circRNA_014805	16:78211482|78214023	ENSSSCG00000017094,	6-Mar	23.25	0	-5.617819819	0.029134225
circRNA_014151	10:29520111|29531391	ENSSSCG00000010934,	-	0	12	5.609375018	0.029164451
circRNA_014142	1:257085934|257093728	ENSSSCG00000005282,	VPS13A	0	12	5.609375018	0.029164451
circRNA_010447	14:113550575|113552254	ENSSSCG00000010470,	IDE	0	12	5.609375018	0.029164451
circRNA_001372	2:148349328|148350512	ENSSSCG00000014376,	HARS2	0	12	5.609375018	0.029164451
circRNA_014328	17:42861078|42862152	ENSSSCG00000026617,	-	0	12	5.609375018	0.029164451
circRNA_003007	12:37398861|37425492	ENSSSCG00000017670,	RPS6KB1	0	11	5.610524345	0.029242432
circRNA_014336	17:52398129|52399297	ENSSSCG00000007375,	SERINC3	0	11	5.610524345	0.029242432
circRNA_014909	1:283070491|283100341	ENSSSCG00000005459,	KIAA0368	0	8	5.612264323	0.029279634
circRNA_014942	6:145384185|145414300	ENSSSCG00000029633,	USP24	0	8	5.612264323	0.029279634
circRNA_010867	9:25669692|25676436	ENSSSCG00000027093,	FOLH1B	0	8	5.612264323	0.029279634

circRNA_010912	GL892407.1:19130|21635	ENSSSCG00000026889,	TMEM237	22.5	0	-5.61083188	0.029361016
circRNA_003922	2:69812430|69813013	ENSSSCG00000013638,	ILF3	22.5	0	-5.605894959	0.029522132
circRNA_005126	9:63116301|63132287	ENSSSCG00000015247,	ZBTB44	22.5	0	-5.605894959	0.029522132
circRNA_004262	4:42104548|42114243	ENSSSCG00000006084,	MTDH	23.5	0	-5.605381265	0.029538939
circRNA_006956	13:1187021|1197644	ENSSSCG00000011183,	-	23.5	0	-5.605381265	0.029538939
circRNA_014859	7:82092|145901	ENSSSCG00000000985,	EXOC2	23.5	0	-5.605381265	0.029538939
circRNA_008328	1:80283905|80293999	ENSSSCG00000004364,	HACE1	17.5	0	-5.599616791	0.029728088
circRNA_005875	15:133558194|133570976	ENSSSCG00000016194,	USP37	24.5	0	-5.596957079	0.029815702
circRNA_014103	1:64727564|64737924	ENSSSCG00000004329,	-	0	10	5.591885646	0.029854273
circRNA_006199	5:38471187|38486778	ENSSSCG00000000511,	RAB21	0	10	5.591885646	0.029854273
circRNA_014539	8:141381057|141401000	ENSSSCG00000026655,	PTPN13	0	10	5.591885646	0.029854273
circRNA_014878	8:71459662|71473071	ENSSSCG00000025514,	RUFY3	22.5	0	-5.594745225	0.029888727
circRNA_014617	1:282147236|282150027	ENSSSCG00000005455,	SVEP1	17	0	-5.592098681	0.029976301
circRNA_012484	12:37398861|37415450	ENSSSCG00000017670,	RPS6KB1	18.25	0	-5.589481151	0.030063125
circRNA_013487	9:116165727|116182100	ENSSSCG00000015430,	RINT1	18.25	0	-5.589481151	0.030063125
circRNA_013090	4:41735670|41742058	ENSSSCG00000006082,	-	23	0	-5.586886185	0.030149408
circRNA_007746	5:48617786|48627688	ENSSSCG00000000543,	-	17.75	0	-5.58580674	0.030185361
circRNA_009329	3:13957642|13957854	ENSSSCG00000007726,	-	18.5	0	-5.579537703	0.030394872
circRNA_006961	13:3130975|3132568	ENSSSCG00000011194,	ANKRD28	18.5	0	-5.579537703	0.030394872
circRNA_014615	1:255073188|255085199	ENSSSCG00000005273,	OSTF1	18.5	0	-5.579537703	0.030394872
circRNA_009176	18:10795154|10806911	ENSSSCG00000016509,	-	25.25	0	-5.577138454	0.030475375
circRNA_014649	13:206362190|206376938	ENSSSCG00000012037,	PAXBP1	19	0	-5.57668674	0.030490552
circRNA_000016	1:16779138|16889979	n/a	_	19	0	-5.573682613	0.030591644
circRNA_012949	2:86103896|86111828	ENSSSCG00000014081,	COL4A3BP	19	0	-5.573682613	0.030591644
circRNA_007298	16:6173606|6186594	ENSSSCG00000016792,	FAM134B	19	0	-5.573682613	0.030591644
circRNA_003205	13:132656250|132656809	ENSSSCG00000028398,	-	25	0	-5.573509548	0.030597477
circRNA_003230	13:148398444|148408637	n/a	_	25	0	-5.573509548	0.030597477
circRNA_015046	7:87883201|87894833	n/a	_	25	0	-5.573509548	0.030597477
circRNA_003408	14:94770180|94775658	ENSSSCG00000010357,	WAPL	25.25	0	-5.573162579	0.030609172
circRNA_010306	11:14463282|14465565	n/a	_	25.25	0	-5.573162579	0.030609172
circRNA_005878	15:137963814|138047261	n/a	_	25.25	0	-5.573162579	0.030609172
circRNA_006265	6:56191827|56192904	n/a	_	20	0	-5.570915681	0.030685002
circRNA_006189	5:4779877|4784298	ENSSSCG00000000071,	ST13	20	0	-5.570915681	0.030685002
circRNA_015027	2:119213114|119228003	ENSSSCG00000014195,	MAN2A1	24.75	0	-5.569863031	0.030720582
circRNA_004599	6:88173912|88196465	ENSSSCG00000003654,	MACF1	24.75	0	-5.569863031	0.030720582
circRNA_014657	14:95077720|95273617	n/a	_	17.75	0	-5.569221305	0.030742289
circRNA_008705	13:3130975|3137949	ENSSSCG00000011194,	ANKRD28	17.75	0	-5.569221305	0.030742289
circRNA_001900	6:128733782|128753848	ENSSSCG00000003784,	-	17.75	0	-5.569221305	0.030742289
circRNA_014812	18:59071515|59335593	n/a	_	22.25	0	-5.568385413	0.030770584
circRNA_014626	11:12312481|12318821	ENSSSCG00000026564,	-	18.25	0	-5.566257377	0.030842714
circRNA_014635	13:12433863|12443091	ENSSSCG00000011211,	THRB	18.25	0	-5.566257377	0.030842714
circRNA_009394	3:104169248|104199715	ENSSSCG00000008467,	EML4	17	0	-5.562066029	0.030985195
circRNA_004106	3:73358334|73374822	ENSSSCG00000022848,	-	19	0	-5.55976231	0.031063741
circRNA_014747	9:139983624|139990100	ENSSSCG00000015576,	TPR	19	0	-5.55976231	0.031063741
circRNA_008727	13:33146950|33157534	ENSSSCG00000011332,	SETD2	19	0	-5.55976231	0.031063741
circRNA_004189	3:119833596|119834135	ENSSSCG00000008573,	RAB10	16.25	0	-5.551497654	0.031346898
circRNA_002268	9:101231884|101252107	ENSSSCG00000015386,	FAM126A	23.75	0	-5.548914308	0.031435842
circRNA_002303	9:143198081|143209415	ENSSSCG00000015586,	RPS6KC1	23.75	0	-5.548914308	0.031435842
circRNA_014441	5:3454359|3458242	ENSSSCG00000000037,	ARFGAP3	0	8.5	5.546100574	0.031482975
circRNA_014512	7:120152807|120165036	ENSSSCG00000002445,	TRIP11	0	8.5	5.546100574	0.031482975
circRNA_014432	4:104473253|104478066	ENSSSCG00000006556,	-	0	8.5	5.546100574	0.031482975
circRNA_014325	17:5203741|5217932	ENSSSCG00000006981,	MICU3	0	8.5	5.546100574	0.031482975

circRNA_014222	13:148454748|148463208	ENSSSCG00000011882,	GOLGB1	0	8.5	5.546100574	0.031482975
circRNA_012731	15:82802125|82904514	n/a	_	0	8.5	5.546100574	0.031482975
circRNA_014184	12:56179696|56184183	ENSSSCG00000017987,	MYH10	0	8.5	5.546100574	0.031482975
circRNA_014341	18:19294616|19296928	ENSSSCG00000016553,	COPG2	0	8.5	5.546100574	0.031482975
circRNA_014126	1:182110736|182124651	ENSSSCG00000004942,	TIPIN	0	8.5	5.546100574	0.031482975
circRNA_007313	16:36895597|36932871	ENSSSCG00000030343,	SLC38A9	20.5	0	-5.546880249	0.031506027
circRNA_014885	9:32422597|32435131	n/a	_	20.75	0	-5.542202238	0.031667935
circRNA_009258	2:72278982|72281790	ENSSSCG00000013566,	-	19.5	0	-5.5395521	0.031759965
circRNA_010510	16:59797585|59878165	n/a	_	19.5	0	-5.5395521	0.031759965
circRNA_014785	13:117698446|117706831	ENSSSCG00000011745,	PRKCI	22.25	0	-5.53398731	0.031953936
circRNA_005639	12:59331765|59383777	ENSSSCG00000018016,	MAP2K4	22.25	0	-5.53398731	0.031953936
circRNA_007832	6:74096530|74115559	n/a	_	22.25	0	-5.53398731	0.031953936
circRNA_014691	3:31095867|31097743	n/a	_	20.25	0	-5.532841606	0.031993994
circRNA_006575	X:85891093|85921778	ENSSSCG00000012474,	DIAPH2	21	0	-5.528467183	0.032147325
circRNA_007099	14:21492868|21532972	ENSSSCG00000009714,	NEK1	21	0	-5.528467183	0.032147325
circRNA_006094	3:104183075|104199715	ENSSSCG00000008467,	EML4	21	0	-5.528467183	0.032147325
circRNA_010834	8:57989763|57990564	n/a	_	22.25	0	-5.527303374	0.032188221
circRNA_012117	GL893884.1:17986|41304	ENSSSCG00000027457,	UBE2Q2	22.25	0	-5.527303374	0.032188221
circRNA_014487	7:10317123|10475528	n/a	_	0	10.5	5.522067039	0.032191386
circRNA_014327	17:9730340|9754135	ENSSSCG00000007004,	INTS10	0	10.5	5.522067039	0.032191386
circRNA_006603	1:14099143|14103753	ENSSSCG00000004070,	SCAF8	0	10.5	5.522067039	0.032191386
circRNA_014527	8:71508448|71512104	ENSSSCG00000008940,	GRSF1	0	10.5	5.522067039	0.032191386
circRNA_014461	5:92111058|92114918	ENSSSCG00000030608,	METAP2	0	10.5	5.522067039	0.032191386
circRNA_014483	6:132108169|132117840	ENSSSCG00000003790,	ANKRD13C	0	10.5	5.522067039	0.032191386
circRNA_011760	4:39632783|39642587	ENSSSCG00000006066,	RNF19A	0	10.5	5.522067039	0.032191386
circRNA_010463	15:3815433|3820638	ENSSSCG00000015668,	ORC4	0	10.5	5.522067039	0.032191386
circRNA_012636	14:34701542|34705614	ENSSSCG00000009834,	ATXN2	20.25	0	-5.520592917	0.032424874
circRNA_003861	18:49341108|49354669	ENSSSCG00000016692,	-	20.25	0	-5.520592917	0.032424874
circRNA_006318	7:94024|99219	ENSSSCG00000000985,	EXOC2	20.25	0	-5.520592917	0.032424874
circRNA_001379	3:5184976|5192337	ENSSSCG00000007590,	PMS2	16	0	-5.518923458	0.032483974
circRNA_002976	12:17069503|17085916	ENSSSCG00000017310,	KANSL1	16	0	-5.518923458	0.032483974
circRNA_003811	18:9603361|9617560	ENSSSCG00000016494,	BRAF	0	11.5	5.508228875	0.032643575
circRNA_003931	2:80454230|80463317	ENSSSCG00000014020,	CANX	0	11.5	5.508228875	0.032643575
circRNA_011192	13:1164933|1171370	ENSSSCG00000011183,	-	0	11.5	5.508228875	0.032643575
circRNA_014246	14:49144364|49157965	n/a	_	0	11.5	5.508228875	0.032643575
circRNA_014354	2:30462773|30464133	ENSSSCG00000013314,	QSER1	0	11.5	5.508228875	0.032643575
circRNA_011912	6:145384185|145402576	ENSSSCG00000029633,	USP24	20.75	0	-5.511929226	0.032732551
circRNA_000752	14:30050907|30067011	ENSSSCG00000009759,	SCARB1	15.5	0	-5.510554294	0.032781603
circRNA_014681	2:90657589|90660943	ENSSSCG00000014119,	-	15.5	0	-5.510554294	0.032781603
circRNA_008087	9:13612179|13616289	ENSSSCG00000014887,	-	0	8.5	5.504062152	0.032955687
circRNA_003438	14:120564864|120566873	ENSSSCG00000010544,	DNMBP	0	8.5	5.504062152	0.032955687
circRNA_014474	6:85037207|85040777	ENSSSCG00000003627,	ZMYM4	0	8.5	5.504062152	0.032955687
circRNA_009061	15:133617126|133627254	ENSSSCG00000016193,	CNOT9	0	8.5	5.504062152	0.032955687
circRNA_006538	GL896223.1:1634|2452	ENSSSCG00000026475,	RRN3	0	8.5	5.504062152	0.032955687
circRNA_006170	4:107403982|107410192	ENSSSCG00000006636,	GABPB2	0	8.5	5.504062152	0.032955687
circRNA_014217	13:132977618|132982390	ENSSSCG00000011794,	SENP2	0	8.5	5.504062152	0.032955687
circRNA_014132	1:207015843|207018021	ENSSSCG00000005065,	AP5M1	0	8.5	5.504062152	0.032955687
circRNA_006802	10:2279240|2287065	ENSSSCG00000010801,	-	21	0	-5.503687148	0.033027507
circRNA_007594	3:109726110|109727889	ENSSSCG00000008498,	HEATR5B	23	0	-5.501250116	0.033115143
circRNA_000912	15:55069306|55072396	ENSSSCG00000015814,	TACC1	23	0	-5.501250116	0.033115143
circRNA_015068	X:30975313|30979082	n/a	_	23	0	-5.501250116	0.033115143
circRNA_010181	1:60903252|60939946	ENSSSCG00000004293,	SNX14	23	0	-5.501250116	0.033115143

circRNA_015026	2:119165841|119266346	ENSSSCG00000014195,	MAN2A1	22.75	0	-5.497238474	0.033259822
circRNA_011593	2:52990682|53006824	ENSSSCG00000013409,	-	22.75	0	-5.497238474	0.033259822
circRNA_015001	15:36338366|36347101	ENSSSCG00000015743,	PTPN4	23	0	-5.496860662	0.033273475
circRNA_014965	1:225104208|225112654	ENSSSCG00000027736,	IFN-ALPHA-15	23	0	-5.496860662	0.033273475
circRNA_015038	5:83970725|83977701	ENSSSCG00000000846,	HCFC2	22.5	0	-5.49320541	0.033405803
circRNA_014998	14:136774429|136888438	n/a	_	22.5	0	-5.49320541	0.033405803
circRNA_010037	GL892397.1:109233|123194	ENSSSCG00000022673,	-	22.5	0	-5.49320541	0.033405803
circRNA_011188	12:62428106|62447784	ENSSSCG00000018039,	NCOR1	16.5	0	-5.49316232	0.033407366
circRNA_011013	1:143396359|143449923	ENSSSCG00000004718,	TTBK2	16.5	0	-5.49316232	0.033407366
circRNA_012279	1:181161592|181172411	ENSSSCG00000004936,	VWA9	16.5	0	-5.49316232	0.033407366
circRNA_014865	7:89101045|89135137	n/a	_	20	0	-5.49150467	0.033467523
circRNA_001299	2:87624115|87625457	n/a	_	17.5	0	-5.490126503	0.033517606
circRNA_000441	12:1401699|1403815	ENSSSCG00000022317,	SLC38A10	17.5	0	-5.490126503	0.033517606
circRNA_012922	2:50783412|50803783	ENSSSCG00000013402,	-	16.25	0	-5.485137936	0.033699413
circRNA_014673	16:70068372|70102462	ENSSSCG00000017043,	RNF145	16.25	0	-5.485137936	0.033699413
circRNA_014705	4:129946021|129949307	ENSSSCG00000006874,	PALMD	16.75	0	-5.481944015	0.033816244
circRNA_002613	1:148474711|148497434	ENSSSCG00000004793,	SPRED1	16.75	0	-5.481826004	0.033820568
circRNA_014745	9:94148684|94242462	n/a	_	16.75	0	-5.481826004	0.033820568
circRNA_008389	1:154299284|154339318	ENSSSCG00000004814,	-	16.75	0	-5.481826004	0.033820568
circRNA_014799	15:53434689|53449943	n/a	_	20	0	-5.472689672	0.034156663
circRNA_003381	14:77896241|77896962	ENSSSCG00000010243,	-	18.25	0	-5.472176368	0.034175628
circRNA_005437	1:109565536|109570791	ENSSSCG00000027690,	-	18.25	0	-5.472176368	0.034175628
circRNA_014802	15:137822150|137984032	n/a	_	18.5	0	-5.467067947	0.034364842
circRNA_004027	2:140877263|140891153	ENSSSCG00000014292,	HSPA4	15.25	0	-5.461999431	0.034553437
circRNA_012655	14:65185232|65189595	ENSSSCG00000010189,	NUP133	15.25	0	-5.461999431	0.034553437
circRNA_003758	17:30263142|30275014	ENSSSCG00000007097,	SEC23B	19	0	-5.46026995	0.034617986
circRNA_006421	8:90731115|90744992	ENSSSCG00000009050,	INPP4B	19	0	-5.46026995	0.034617986
circRNA_002162	8:144739680|144748563	ENSSSCG00000009244,	SEC31A	17.25	0	-5.459455129	0.034648432
circRNA_013462	9:63000517|63001823	ENSSSCG00000015244,	APLP2	18	0	-5.458125034	0.034698179
circRNA_014894	GL894386.1:44564|48180	ENSSSCG00000004921,	ATP8B1	18	0	-5.458125034	0.034698179
circRNA_012219	1:84723988|84732574	ENSSSCG00000004414,	CD164	18	0	-5.458125034	0.034698179
circRNA_013644	8:78366796|78377848	n/a	_	6.5	51.5	3.462503097	0.034755638
circRNA_004945	8:73375718|73386901	ENSSSCG00000024592,	ANKRD17	14	0	-5.453232817	0.034881662
circRNA_014729	8:20681698|20707578	ENSSSCG00000008761,	TBC1D19	14	0	-5.453232817	0.034881662
circRNA_002942	11:76626393|76642442	ENSSSCG00000009524,	TMTC4	18	0	-5.45204033	0.034926508
circRNA_014389	3:50658315|50658607	n/a	_	0	7	5.45277748	0.034939861
circRNA_012631	14:32135166|32136110	ENSSSCG00000009793,	CLIP1	0	7	5.45277748	0.034939861
circRNA_014514	7:130875508|130883781	ENSSSCG00000002547,	-	0	7	5.45277748	0.034939861
circRNA_013310	7:33242142|33263702	ENSSSCG00000001498,	BEND6	18.5	0	-5.442922694	0.035270979
circRNA_002703	1:242727958|242737129	ENSSSCG00000005215,	JAK2	18.5	0	-5.442922694	0.035270979
circRNA_014897	X:46490203|46515564	ENSSSCG00000012267,	JADE3	18.5	0	-5.442922694	0.035270979
circRNA_009003	15:53415811|53437329	n/a	_	19.75	0	-5.441640477	0.035319648
circRNA_014772	10:60057311|60067090	n/a	_	18	0	-5.438460932	0.035440571
circRNA_014553	9:51074787|51079497	ENSSSCG00000015097,	DDX6	0	9	5.427643518	0.035731924
circRNA_011159	12:13030858|13093840	ENSSSCG00000023992,	-	0	9	5.427643518	0.035731924
circRNA_008195	GL893669.1:73398|79691	ENSSSCG00000030404,	PAPD5	0	9	5.427643518	0.035731924
circRNA_002074	8:34341118|34372263	ENSSSCG00000008799,	-	0	9	5.427643518	0.035731924
circRNA_014502	7:91917856|91939138	ENSSSCG00000002266,	CHD2	0	9	5.427643518	0.035731924
circRNA_014548	9:32326743|32329262	n/a	_	0	10	5.419535551	0.035934759
circRNA_008865	13:216669717|216696594	ENSSSCG00000022164,	HSF2BP	0	10	5.419535551	0.035934759
circRNA_014104	1:83289162|83323066	ENSSSCG00000004382,	SEC63	0	10	5.419535551	0.035934759
circRNA_004706	6:152329597|152336837	ENSSSCG00000003895,	EFCAB14	0	10	5.419535551	0.035934759

circRNA_014129	1:202650920|202656440	ENSSSCG00000005039,	STYX	0	10	5.419535551	0.035934759
circRNA_008355	1:110527369|110538400	ENSSSCG00000004524,	SMAD4	0	10	5.419535551	0.035934759
circRNA_014164	10:66012330|66042667	n/a	_	0	10	5.419535551	0.035934759
circRNA_010045	GL892553.2:91193|103174	ENSSSCG00000022860,	SASH1	0	10	5.419535551	0.035934759
circRNA_012741	15:89580329|89586283	ENSSSCG00000023297,	-	0	10	5.419535551	0.035934759
circRNA_006029	2:118253397|118371589	ENSSSCG00000014191,	-	48.75	3	-3.691182416	0.036127199
circRNA_000460	12:18731949|18736767	ENSSSCG00000017346,	EFTUD2	4.25	37.25	3.566311024	0.036139328
circRNA_013042	3:102852283|102863985	n/a	_	20.75	0	-5.417016635	0.036265118
circRNA_005800	14:113064543|113074428	ENSSSCG00000010467,	-	20.75	0	-5.417016635	0.036265118
circRNA_014975	11:495509|508754	ENSSSCG00000026744,	ZDHHC20	20.75	0	-5.417016635	0.036265118
circRNA_010615	3:44873984|45107781	n/a	_	20.75	0	-5.417016635	0.036265118
circRNA_015060	9:52673623|52688827	ENSSSCG00000015129,	ARHGEF12	20.75	0	-5.417016635	0.036265118
circRNA_004608	6:89974808|89998069	ENSSSCG00000003673,	CEP192	21	0	-5.41660441	0.036281123
circRNA_003077	13:21193239|21199595	ENSSSCG00000011237,	PDCD6IP	21	0	-5.41660441	0.036281123
circRNA_007188	14:132243465|132252629	ENSSSCG00000010627,	PDCD4	21	0	-5.41660441	0.036281123
circRNA_008499	1:299797832|299801634	ENSSSCG00000005602,	GAPVD1	18.5	0	-5.415189159	0.036336114
circRNA_014828	3:47969752|47974155	ENSSSCG00000008111,	NPHP1	18.5	0	-5.415189159	0.036336114
circRNA_004940	8:69688714|69704803	ENSSSCG00000008921,	-	18.5	0	-5.415189159	0.036336114
circRNA_015019	18:36505709|36552661	ENSSSCG00000025602,	-	20.25	0	-5.412949541	0.036423278
circRNA_011380	14:136306844|136311233	ENSSSCG00000010652,	FAM160B1	14.25	0	-5.412615705	0.036436286
circRNA_005168	9:127867416|127868250	ENSSSCG00000015498,	RC3H1	20.5	0	-5.412535459	0.036439413
circRNA_007869	6:132199188|132217626	ENSSSCG00000003791,	SRSF11	20.5	0	-5.412535459	0.036439413
circRNA_011390	15:19253545|19263276	ENSSSCG00000015695,	RAB3GAP1	20.75	0	-5.412121232	0.036455559
circRNA_014167	11:3766438|3769323	ENSSSCG00000009300,	WASF3	0	7	5.409058772	0.036582818
circRNA_007828	6:69990483|69994497	n/a	_	0	7	5.409058772	0.036582818
circRNA_014404	3:104155199|104195188	ENSSSCG00000008467,	EML4	0	7	5.409058772	0.036582818
circRNA_011711	3:92457168|92458722	ENSSSCG00000008412,	PSME4	0	7	5.409058772	0.036582818
circRNA_005098	9:40513547|40532789	ENSSSCG00000029198,	CUL5	0	7	5.409058772	0.036582818
circRNA_014170	11:19712163|19749205	ENSSSCG00000009401,	RB1	0	7	5.409058772	0.036582818
circRNA_004253	4:37761441|37770008	ENSSSCG00000022479,	-	20.25	0	-5.40802767	0.036615441
circRNA_004791	7:67504695|67587294	n/a	_	17.75	0	-5.40603626	0.036693428
circRNA_014771	10:37797097|37817442	ENSSSCG00000011001,	APTX	17.75	0	-5.40603626	0.036693428
circRNA_006223	5:81374500|81393509	n/a	_	17.75	0	-5.40603626	0.036693428
circRNA_014811	18:5545554|5550750	n/a	_	17.75	0	-5.40603626	0.036693428
circRNA_014860	7:9900963|9904235	ENSSSCG00000001052,	PHACTR1	17.75	0	-5.40603626	0.036693428
circRNA_010132	X:70495253|70646265	ENSSSCG00000012434,	-	15	0	-5.399759584	0.036940134
circRNA_014627	11:19108869|19158675	ENSSSCG00000029933,	-	15	0	-5.399759584	0.036940134
circRNA_001060	16:37511575|37514704	ENSSSCG00000016916,	IL6ST	15	0	-5.399759584	0.036940134
circRNA_014699	4:31396526|31463369	ENSSSCG00000006032,	EMC2	15	0	-5.399759584	0.036940134
circRNA_014644	13:130157827|130372748	n/a	_	15	0	-5.399759584	0.036940134
circRNA_008452	1:233195418|233196805	ENSSSCG00000005191,	MPDZ	15	0	-5.399759584	0.036940134
circRNA_014819	2:119205853|119229632	ENSSSCG00000014195,	MAN2A1	18.75	0	-5.398997642	0.036970175
circRNA_014887	9:72025883|72035603	ENSSSCG00000015288,	RBBP5	18.75	0	-5.398997642	0.036970175
circRNA_014716	6:18418914|18424962	ENSSSCG00000002799,	CNOT1	14.5	0	-5.395024232	0.037127164
circRNA_014708	5:3468414|3471014	ENSSSCG00000000037,	ARFGAP3	14.5	0	-5.395024232	0.037127164
circRNA_006404	8:58349146|58350639	ENSSSCG00000023269,	EXOC1	14.5	0	-5.395024232	0.037127164
circRNA_014733	8:71567714|71589148	ENSSSCG00000008941,	MOB1B	14.5	0	-5.395024232	0.037127164
circRNA_002067	8:29293951|29297807	ENSSSCG00000008768,	ARAP2	14.5	0	-5.395024232	0.037127164
circRNA_001328	2:119260427|119266346	ENSSSCG00000014195,	MAN2A1	14.5	0	-5.395024232	0.037127164
circRNA_014814	2:15925033|15933940	ENSSSCG00000013223,	-	17.75	0	-5.384999773	0.037525675
circRNA_001529	3:130810562|130868878	ENSSSCG00000008621,	-	15.75	0	-5.379601822	0.037741723
circRNA_010671	4:73577490|73591712	ENSSSCG00000006201,	ARFGEF1	15.75	0	-5.379601822	0.037741723

circRNA_007413	18:59628324|59641061	ENSSSCG00000016769,	CDK13	15.75	0	-5.379601822	0.037741723
circRNA_001081	17:728755|734222	ENSSSCG00000006968,	LONRF1	15.75	0	-5.379601822	0.037741723
circRNA_003356	14:53590430|53609301	ENSSSCG00000010081,	MAPK1	16.75	0	-5.376158899	0.037880058
circRNA_004716	7:12247873|12298480	ENSSSCG00000001062,	DTNBP1	16.75	0	-5.376158899	0.037880058
circRNA_002812	10:37465102|37470957	n/a	_	16.75	0	-5.376158899	0.037880058
circRNA_009160	17:49153503|49171103	ENSSSCG00000007355,	TOP1	14.5	0	-5.373823444	0.037974134
circRNA_005434	1:101009473|101026424	ENSSSCG00000004478,	MYO6	14.5	0	-5.373823444	0.037974134
circRNA_014901	X:126963525|126987749	ENSSSCG00000012695,	INTS6L	18.75	0	-5.372626404	0.038022427
circRNA_014662	15:61682878|61690094	ENSSSCG00000015842,	PPP2CB	15	0	-5.36999117	0.038128921
circRNA_009032	15:86615927|86622694	ENSSSCG00000015944,	TLK1	15	0	-5.36999117	0.038128921
circRNA_001965	7:46279250|46286677	ENSSSCG00000025551,	-	15	0	-5.36999117	0.038128921
circRNA_014726	7:87834158|87836047	n/a	_	15	0	-5.36999117	0.038128921
circRNA_014721	6:99190172|99196807	ENSSSCG00000003699,	GREB1L	15	0	-5.36999117	0.038128921
circRNA_014752	X:10411139|10427663	ENSSSCG00000012116,	PRPS2	13.75	0	-5.364499252	0.038351648
circRNA_013302	7:10553476|10565336	ENSSSCG00000001057,	MCUR1	13.75	0	-5.364499252	0.038351648
circRNA_014762	1:125491718|125495611	ENSSSCG00000004595,	ADAM10	15.75	0	-5.360489022	0.038390876
circRNA_014813	2:3011482|3029700	ENSSSCG00000012884,	PPP6R3	15.75	0	-5.360489022	0.038390876
circRNA_008069	8:144739677|144751930	n/a	_	15.75	0	-5.360489022	0.038390876
circRNA_005769	14:28594322|28598456	n/a	_	15.75	0	-5.360489022	0.038390876
circRNA_011563	2:3011482|3039309	ENSSSCG00000012884,	PPP6R3	15.75	0	-5.360489022	0.038390876
circRNA_008918	14:67540897|67575848	ENSSSCG00000010209,	FAM13C	15.75	0	-5.360489022	0.038390876
circRNA_014856	6:144709992|144717668	n/a	_	15.75	0	-5.360489022	0.038390876
circRNA_014881	8:140973217|140974563	ENSSSCG00000023593,	-	17.75	0	-5.363385336	0.038396954
circRNA_014847	6:31447413|31456726	ENSSSCG00000002841,	N4BP1	17.75	0	-5.363385336	0.038396954
circRNA_002992	12:31696118|31699978	ENSSSCG00000017601,	TOM1L1	17.75	0	-5.363385336	0.038396954
circRNA_014774	11:19657687|19663002	ENSSSCG00000009401,	RB1	17.75	0	-5.363385336	0.038396954
circRNA_014823	2:141885830|141895222	ENSSSCG00000014296,	VDAC1	17.75	0	-5.363385336	0.038396954
circRNA_014641	13:98121118|98162682	ENSSSCG00000027459,	RNF13	13	0	-5.360951999	0.038405598
circRNA_010665	4:45690143|45704929	n/a	_	13	0	-5.360951999	0.038405598
circRNA_006633	1:57195566|57275978	ENSSSCG00000004276,	SMAP1	13	0	-5.360951999	0.038405598
circRNA_014659	14:136012794|136018388	ENSSSCG00000010651,	ABLIM1	13	0	-5.360951999	0.038405598
circRNA_014748	GL893689.1:3069|10757	n/a	_	13	0	-5.360951999	0.038405598
circRNA_014666	15:103657066|103661579	ENSSSCG00000016033,	GULP1	13	0	-5.360951999	0.038405598
circRNA_007074	13:159951029|159976282	ENSSSCG00000011940,	DZIP3	15.75	0	-5.361712652	0.03846507
circRNA_004045	2:150648583|150660330	ENSSSCG00000014399,	ARHGAP26	15.75	0	-5.361712652	0.03846507
circRNA_003337	14:34693427|34697216	ENSSSCG00000009834,	ATXN2	15.75	0	-5.361712652	0.03846507
circRNA_014723	6:141363206|141385665	ENSSSCG00000003830,	MYSM1	15.75	0	-5.361712652	0.03846507
circRNA_014840	4:136805197|136880888	ENSSSCG00000006911,	-	16.25	0	-5.356826729	0.038664606
circRNA_014835	4:39929686|39932326	ENSSSCG00000006069,	RGS22	16.25	0	-5.356826729	0.038664606
circRNA_014706	4:135605447|135620204	ENSSSCG00000006897,	-	17.25	0	-5.354020642	0.038701339
circRNA_005815	14:140519939|140529589	ENSSSCG00000010679,	EIF3A	12.5	0	-5.354458048	0.038761647
circRNA_005068	9:18937518|18948522	ENSSSCG00000014899,	PRCP	12.5	0	-5.354458048	0.038761647
circRNA_014956	1:16119378|16124594	ENSSSCG00000004081,	-	18.5	0	-5.347944843	0.038800193
circRNA_005739	13:167147677|167149553	ENSSSCG00000021791,	SENP7	18.5	0	-5.347944843	0.038800193
circRNA_015067	JH118611.1:2203|14701	ENSSSCG00000022081,	-	18.5	0	-5.347944843	0.038800193
circRNA_002851	10:62755545|62767160	ENSSSCG00000011103,	PARD3	18.25	0	-5.344433293	0.03893035
circRNA_014989	13:190108609|190131796	n/a	_	18.25	0	-5.344433293	0.03893035
circRNA_006641	1:71041497|71055642	ENSSSCG00000004337,	MANEA	18.25	0	-5.344433293	0.03893035
circRNA_014964	1:224091690|224111707	ENSSSCG00000005135,	-	18.25	0	-5.344433293	0.03893035
circRNA_015024	2:78344472|78349798	ENSSSCG00000013424,	-	18.25	0	-5.344433293	0.03893035
circRNA_015039	6:32224765|32242475	ENSSSCG00000002844,	PHKB	18.25	0	-5.344433293	0.03893035
circRNA_008878	14:14027675|14036603	ENSSSCG00000009682,	HMBOX1	18.25	0	-5.344433293	0.03893035

circRNA_010053	GL893514.2:12087|53528	n/a	_	18.25	0	-5.344433293	0.03893035
circRNA_015059	9:51522099|51522697	ENSSSCG00000015115,	CBL	18.25	0	-5.344433293	0.03893035
circRNA_002149	8:134379373|134409025	ENSSSCG00000009192,	PDLIM5	190	52	-1.39662779	0.039277252
circRNA_006574	X:70495253|70639982	ENSSSCG00000012434,	-	18	0	-5.336127946	0.039303013
circRNA_001810	6:63452187|63496102	ENSSSCG00000025445,	-	18	0	-5.336127946	0.039303013
circRNA_009484	4:98044696|98053981	ENSSSCG00000006386,	COPA	18	0	-5.336127946	0.039303013
circRNA_015012	17:43030480|43037474	ENSSSCG00000007280,	ITCH	18	0	-5.336127946	0.039303013
circRNA_007955	7:104451564|104454866	ENSSSCG00000021606,	-	18	0	-5.336127946	0.039303013
circRNA_004204	3:122008499|122024159	ENSSSCG00000008591,	-	18	0	-5.336127946	0.039303013
circRNA_006680	1:132819046|132828697	ENSSSCG00000004624,	MAPK6	18	0	-5.336127946	0.039303013
circRNA_015058	8:140242798|140246892	ENSSSCG00000009217,	PKD2	18	0	-5.336127946	0.039303013
circRNA_007184	14:122832506|122870587	ENSSSCG00000010571,	C10orf76	16.25	0	-5.340082816	0.039314884
circRNA_005589	11:18975972|18988063	ENSSSCG00000022159,	-	18	0	-5.336115729	0.039354547
circRNA_015056	8:127787109|127797662	ENSSSCG00000009169,	SLC39A8	18	0	-5.336115729	0.039354547
circRNA_007473	2:83206943|83219454	ENSSSCG00000014062,	SIMC1	18	0	-5.336115729	0.039354547
circRNA_015004	15:61774735|61788609	ENSSSCG00000015844,	GSR	18	0	-5.336115729	0.039354547
circRNA_011213	13:35734591|35739889	ENSSSCG00000011394,	RBM6	18	0	-5.336115729	0.039354547
circRNA_015033	3:114476160|114487607	ENSSSCG00000008517,	SPAST	18	0	-5.336115729	0.039354547
circRNA_005814	14:140515705|140520043	ENSSSCG00000010679,	EIF3A	18	0	-5.336115729	0.039354547
circRNA_014825	3:24136275|24141464	ENSSSCG00000030373,	METTL9	16	0	-5.333497124	0.039463015
circRNA_014888	9:101847443|101851121	ENSSSCG00000020870,	DBF4	16	0	-5.333497124	0.039463015
circRNA_004115	3:81212631|81219219	ENSSSCG00000008363,	AFTPH	16	0	-5.333497124	0.039463015
circRNA_001648	4:129295358|129336726	ENSSSCG00000006864,	CDC14A	18.5	0	-5.333653785	0.039471078
circRNA_006795	1:301515256|301535195	ENSSSCG00000005607,	RALGPS1	0	8.5	5.322122849	0.039834728
circRNA_012828	17:12529639|12536452	ENSSSCG00000007023,	KAT6A	0	8.5	5.322122849	0.039834728
circRNA_014581	GL893655.1:37492|55141	ENSSSCG00000029119,	PRPF6	0	8.5	5.322122849	0.039834728
circRNA_004406	5:5192560|5193695	ENSSSCG00000000075,	MKL1	0	8.5	5.322122849	0.039834728
circRNA_014271	15:3551454|3687801	n/a	_	0	8.5	5.322122849	0.039834728
circRNA_014317	16:52884700|52933109	n/a	_	0	8.5	5.322122849	0.039834728
circRNA_009925	9:21947403|21949364	ENSSSCG00000014909,	SYTL2	0	8.5	5.322122849	0.039834728
circRNA_014203	13:75857204|75864289	ENSSSCG00000022633,	-	0	8.5	5.322122849	0.039834728
circRNA_014841	5:23685402|23692412	ENSSSCG00000000406,	PTGES3	15.5	0	-5.32503805	0.039845676
circRNA_002536	1:93269648|93277132	ENSSSCG00000004456,	PGM3	15.5	0	-5.32503805	0.039845676
circRNA_014864	7:89078260|89135137	n/a	_	15.5	0	-5.32503805	0.039845676
circRNA_006967	13:16472252|16526599	ENSSSCG00000028258,	AZI2	15.5	0	-5.32503805	0.039845676
circRNA_014797	15:3788416|3794104	ENSSSCG00000015668,	ORC4	15.5	0	-5.32503805	0.039845676
circRNA_014803	16:23583623|23586581	ENSSSCG00000016843,	C5orf42	15.5	0	-5.32503805	0.039845676
circRNA_010072	GL894982.1:32013|45312	n/a	_	15.5	0	-5.32503805	0.039845676
circRNA_006704	1:154649289|154667163	ENSSSCG00000028771,	-	15.5	0	-5.32503805	0.039845676
circRNA_012589	13:148879705|148909385	n/a	_	15.5	0	-5.32503805	0.039845676
circRNA_014898	X:67062592|67071339	ENSSSCG00000012416,	CHIC1	15.5	0	-5.32503805	0.039845676
circRNA_014837	4:109041942|109042735	ENSSSCG00000026502,	LIX1L	15.5	0	-5.32503805	0.039845676
circRNA_012294	1:212171161|212172500	ENSSSCG00000005096,	HIF1A	16.25	0	-5.313554846	0.040314716
circRNA_014834	4:37582966|37583979	ENSSSCG00000025427,	CU459197.4	16.25	0	-5.313554846	0.040314716
circRNA_000717	13:210819581|210836997	ENSSSCG00000012062,	TTC3	181	260.25	1.008440075	0.040492555
circRNA_011212	13:35728481|35737676	ENSSSCG00000011394,	RBM6	12.75	0	-5.308785694	0.040666894
circRNA_006193	5:13399178|13399786	ENSSSCG00000000167,	RIC8B	12.75	0	-5.308785694	0.040666894
circRNA_014607	1:30979937|30990296	ENSSSCG00000026041,	-	12.75	0	-5.308785694	0.040666894
circRNA_009158	17:47003535|47067299	ENSSSCG00000007345,	-	12.75	0	-5.308785694	0.040666894
circRNA_014900	X:90715380|90717029	ENSSSCG00000012491,	CENPI	15.5	0	-5.299078418	0.040967294
circRNA_014838	4:117897403|117903206	ENSSSCG00000006779,	ST7L	15.5	0	-5.299078418	0.040967294
circRNA_014760	1:75089371|75101925	ENSSSCG00000004351,	USP45	15.5	0	-5.299078418	0.040967294

circRNA_007724	5:16670864|16694950	ENSSSCG00000028516,	LARP4	15.5	0	-5.299078418	0.040967294
circRNA_014709	5:29938324|29947722	ENSSSCG00000000458,	MON2	12.25	0	-5.297147226	0.041171396
circRNA_014687	2:121986386|122033234	ENSSSCG00000014209,	EPB41L4A	12.25	0	-5.297147226	0.041171396
circRNA_014609	1:63729107|63748701	n/a	_	12.25	0	-5.297147226	0.041171396
circRNA_014689	2:128512404|128524824	ENSSSCG00000027857,	DMXL1	12.25	0	-5.297147226	0.041171396
circRNA_011041	1:224111540|224114675	ENSSSCG00000005135,	-	12.25	0	-5.297147226	0.041171396
circRNA_009087	16:25422928|25426853	ENSSSCG00000016853,	RICTOR	12.25	0	-5.297147226	0.041171396
circRNA_014753	X:21174276|21179921	ENSSSCG00000012171,	PRDX4	12.25	0	-5.297147226	0.041171396
circRNA_000420	11:53974514|53991328	ENSSSCG00000009473,	MYCBP2	12.25	0	-5.297147226	0.041171396
circRNA_014663	15:86615927|86639123	ENSSSCG00000015944,	TLK1	12.25	0	-5.297147226	0.041171396
circRNA_001215	2:4334166|4579464	n/a	_	16.5	0	-5.286986866	0.041526276
circRNA_014848	6:40797702|40798115	ENSSSCG00000002921,	CLIP3	16.5	0	-5.286986866	0.041526276
circRNA_000233	1:263840380|263879756	n/a	_	11	109.25	3.867899381	0.041868699
circRNA_004301	4:79094980|79101679	ENSSSCG00000006231,	CHD7	53.75	98.75	1.42913345	0.042034239
circRNA_009310	2:142522062|142528964	ENSSSCG00000014304,	SEC24A	13.25	0	-5.276345143	0.042057094
circRNA_006966	13:14280479|14284639	ENSSSCG00000011214,	NGLY1	13.25	0	-5.276345143	0.042057094
circRNA_011855	5:109528884|109541874	n/a	_	13.25	0	-5.276345143	0.042057094
circRNA_014680	2:80575287|80577295	ENSSSCG00000014023,	RUFY1	13.25	0	-5.276345143	0.042057094
circRNA_014622	10:49568556|49571722	ENSSSCG00000011040,	CACNB2	13.25	0	-5.276345143	0.042057094
circRNA_007470	2:82120327|82157445	ENSSSCG00000014044,	NSD1	13.25	0	-5.276345143	0.042057094
circRNA_014669	16:23465932|23467865	ENSSSCG00000016842,	NIPBL	13.25	0	-5.276345143	0.042057094
circRNA_014630	12:161887|164543	ENSSSCG00000017125,	FOXK2	13.25	0	-5.276345143	0.042057094
circRNA_006200	5:38575947|38581769	ENSSSCG00000000512,	TBC1D15	14.25	0	-5.273048749	0.042196571
circRNA_014742	9:77278140|77725461	ENSSSCG00000015307,	-	14.25	0	-5.273048749	0.042196571
circRNA_014743	9:78882583|78909044	n/a	_	14.25	0	-5.273048749	0.042196571
circRNA_000165	1:195169049|195174506	ENSSSCG00000004997,	PRPF39	14.25	0	-5.273048749	0.042196571
circRNA_005926	17:40696156|40700671	ENSSSCG00000007246,	TM9SF4	14.25	0	-5.273048749	0.042196571
circRNA_007273	15:112541222|112545392	ENSSSCG00000016077,	HSPD1	14.25	0	-5.273048749	0.042196571
circRNA_014727	7:121217521|121232103	ENSSSCG00000002461,	BTBD7	14.25	0	-5.273048749	0.042196571
circRNA_003225	13:145860014|145922001	n/a	_	4.75	328.25	4.940918495	0.042218106
circRNA_014614	1:250481322|250505262	ENSSSCG00000005257,	TRPM3	12.75	0	-5.26875287	0.042410064
circRNA_014703	4:121746651|121754075	ENSSSCG00000006843,	STXBP3	12.75	0	-5.26875287	0.042410064
circRNA_003149	13:82870481|82899646	ENSSSCG00000011642,	RYK	12.75	0	-5.26875287	0.042410064
circRNA_014724	6:148405581|148440705	ENSSSCG00000003872,	EPS15	12.75	0	-5.26875287	0.042410064
circRNA_013489	9:116762218|116762697	n/a	_	12.75	0	-5.26875287	0.042410064
circRNA_010767	6:136176938|136181870	ENSSSCG00000004829,	CACHD1	12.75	0	-5.26875287	0.042410064
circRNA_010674	4:85302651|85304511	ENSSSCG00000006267,	PCMTD1	15.5	0	-5.266219945	0.042477163
circRNA_010538	18:9534503|9570018	ENSSSCG00000016494,	BRAF	15.5	0	-5.266219945	0.042477163
circRNA_014620	10:2342778|2355944	ENSSSCG00000010802,	-	13.5	0	-5.259390307	0.042825124
circRNA_005417	1:37113063|37126736	ENSSSCG00000004205,	-	13.5	0	-5.259390307	0.042825124
circRNA_014750	GL894855.1:11155|29194	ENSSSCG00000025025,	-	13.5	0	-5.259390307	0.042825124
circRNA_014707	4:139715852|139724066	ENSSSCG00000006925,	KYAT3	13.5	0	-5.259390307	0.042825124
circRNA_005910	16:78913434|78966976	ENSSSCG00000017095,	SEMA5A	13.5	0	-5.259390307	0.042825124
circRNA_006138	4:40343834|40360156	n/a	_	13.5	0	-5.259390307	0.042825124
circRNA_014612	1:176836675|176838127	ENSSSCG00000004901,	PIGN	13.5	0	-5.259390307	0.042825124
circRNA_014711	5:87252145|87266366	ENSSSCG00000000873,	ANO4	13.5	0	-5.259390307	0.042825124
circRNA_000037	1:37134829|37340400	n/a	_	51	4.5	-3.428284338	0.043023786
circRNA_006893	11:53888439|53891329	ENSSSCG00000009473,	MYCBP2	15	0	-5.246775516	0.043389613
circRNA_014672	16:47942252|47945309	n/a	_	11.5	0	-5.237024434	0.043830112
circRNA_006016	2:91513977|91546743	ENSSSCG00000014127,	RASGRF2	11.5	0	-5.237024434	0.043830112
circRNA_014734	8:89347874|89348339	ENSSSCG00000009047,	SMARCA5	11.5	0	-5.237024434	0.043830112
circRNA_004352	4:122750450|122766803	ENSSSCG00000006851,	-	11.5	0	-5.237024434	0.043830112

circRNA_010837	8:71582612|71589148	ENSSSCG00000008941,	MOB1B	11.5	0	-5.237024434	0.043830112
circRNA_012966	2:121253565|121295422	ENSSSCG00000029033,	-	11.5	0	-5.237024434	0.043830112
circRNA_014676	2:15680718|15686214	ENSSSCG00000023709,	PTPRJ	11.5	0	-5.237024434	0.043830112
circRNA_014645	13:130358254|130372748	ENSSSCG00000022783,	DCUN1D1	11.5	0	-5.237024434	0.043830112
circRNA_007512	2:132227980|132248199	ENSSSCG00000014239,	CEP120	11.5	0	-5.237024434	0.043830112
circRNA_014740	9:63604082|63618674	n/a	_	11.5	0	-5.237024434	0.043830112
circRNA_007029	13:91490811|91493168	ENSSSCG00000011684,	U2SURP	12	0	-5.232763885	0.044023724
circRNA_008111	9:46187651|46193994	ENSSSCG00000015050,	ZW10	16	0	-5.217433085	0.044547533
circRNA_008131	9:78959290|78997862	ENSSSCG00000015314,	ANKIB1	16	0	-5.217433085	0.044547533
circRNA_003972	2:91206107|91335022	n/a	_	16	0	-5.217433085	0.044547533
circRNA_005140	9:82869539|82893921	ENSSSCG00000015335,	DYNC1I1	16	0	-5.217433085	0.044547533
circRNA_006915	12:16876107|16878244	ENSSSCG00000017308,	CDC27	16	0	-5.217433085	0.044547533
circRNA_003623	15:120243210|120244394	ENSSSCG00000016123,	-	16	0	-5.217433085	0.044547533
circRNA_002197	9:13522653|13735468	n/a	_	16	0	-5.217433085	0.044547533
circRNA_002521	1:80241844|80293999	ENSSSCG00000004364,	HACE1	16	0	-5.217433085	0.044547533
circRNA_015044	7:10297937|10304324	ENSSSCG00000001056,	RANBP9	16	0	-5.217433085	0.044547533
circRNA_001459	3:91185717|91190405	ENSSSCG00000008408,	-	16	0	-5.217433085	0.044547533
circRNA_005982	2:60043010|60053359	ENSSSCG00000013870,	-	16	0	-5.217433085	0.044547533
circRNA_015011	16:65140674|65154889	ENSSSCG00000017022,	HMMR	16	0	-5.217433085	0.044547533
circRNA_011591	2:50802485|50805720	ENSSSCG00000013402,	-	10.75	0	-5.220161398	0.044586731
circRNA_014618	1:282152370|282195982	n/a	_	10.75	0	-5.220161398	0.044586731
circRNA_010881	9:63116301|63143034	n/a	_	16.25	0	-5.217575933	0.044639321
circRNA_008624	11:75120545|75132336	ENSSSCG00000022112,	DOCK9	16.25	0	-5.217575933	0.044639321
circRNA_002517	1:72497312|72506164	ENSSSCG00000028811,	-	16.25	0	-5.217575933	0.044639321
circRNA_015062	9:78882583|78889684	ENSSSCG00000015313,	KRIT1	16.25	0	-5.217575933	0.044639321
circRNA_009104	16:47310150|47349817	n/a	_	16.25	0	-5.217575933	0.044639321
circRNA_014999	14:140512869|140520043	ENSSSCG00000010679,	EIF3A	16.25	0	-5.217575933	0.044639321
circRNA_015015	17:58803349|58814020	ENSSSCG00000007474,	DPM1	15.75	0	-5.21081346	0.04484216
circRNA_004370	4:129639341|129643320	ENSSSCG00000006870,	-	15.75	0	-5.21081346	0.04484216
circRNA_005842	15:67009488|67021943	ENSSSCG00000015866,	FMNL2	15.75	0	-5.21081346	0.04484216
circRNA_013013	3:58911870|58947781	n/a	_	15.75	0	-5.21081346	0.04484216
circRNA_014980	12:13065330|13093840	ENSSSCG00000023992,	-	15.75	0	-5.21081346	0.04484216
circRNA_004985	8:103675822|103712082	n/a	_	15.75	0	-5.21081346	0.04484216
circRNA_015057	8:140241026|140255386	ENSSSCG00000009217,	PKD2	15.75	0	-5.21081346	0.04484216
circRNA_014137	1:229131344|229246387	ENSSSCG00000005178,	CNTLN	0	7	5.20624521	0.044850077
circRNA_000392	11:18571298|18580399	ENSSSCG00000009395,	SETDB2	0	7	5.20624521	0.044850077
circRNA_014588	GL896504.1:27999|31588	ENSSSCG00000027115,	-	0	7	5.20624521	0.044850077
circRNA_014268	14:142840673|142840902	ENSSSCG00000010699,	ATE1	0	7	5.20624521	0.044850077
circRNA_009616	6:23732487|23753262	ENSSSCG00000002795,	CDH11	15.75	0	-5.212519987	0.044850179
circRNA_011381	14:136612215|136857384	ENSSSCG00000010654,	ATRNL1	15.75	0	-5.212519987	0.044850179
circRNA_014986	13:102343369|102348459	ENSSSCG00000024916,	DHX36	15.75	0	-5.212519987	0.044850179
circRNA_004939	8:64483430|64514152	n/a	_	15.75	0	-5.212519987	0.044850179
circRNA_006945	12:45334013|45365521	ENSSSCG00000017748,	NF1	15.75	0	-5.212519987	0.044850179
circRNA_015040	6:73334893|73383647	n/a	_	15.75	0	-5.212519987	0.044850179
circRNA_004126	3:84274641|84291468	ENSSSCG00000008382,	-	15.75	0	-5.212519987	0.044850179
circRNA_014836	4:80758319|80776999	ENSSSCG00000006235,	TOX	13.75	0	-5.211340153	0.0449753
circRNA_009125	16:78211482|78217793	ENSSSCG00000017094,	6-Mar	13.75	0	-5.211340153	0.0449753
circRNA_014883	9:14158052|14201808	ENSSSCG00000014893,	NARS2	13.75	0	-5.211340153	0.0449753
circRNA_014780	13:31934660|31945984	ENSSSCG00000026885,	-	13.75	0	-5.211340153	0.0449753
circRNA_004461	5:70588009|70693708	n/a	_	13.75	0	-5.211340153	0.0449753
circRNA_014873	8:40452798|40459063	ENSSSCG00000008826,	-	13.75	0	-5.211340153	0.0449753
circRNA_014886	9:46280538|46282234	ENSSSCG00000015052,	USP28	13.75	0	-5.211340153	0.0449753

circRNA_011399	15:53790613|53800256	ENSSSCG00000015802,	-	13.25	0	-5.197743649	0.045579694
circRNA_014777	12:38779681|38801846	ENSSSCG00000017676,	BCAS3	13.25	0	-5.197743649	0.045579694
circRNA_014891	9:138721213|138727029	ENSSSCG00000015568,	RNF2	13.25	0	-5.197743649	0.045579694
circRNA_014816	2:80461235|80463317	ENSSSCG00000014020,	CANX	13.25	0	-5.197743649	0.045579694
circRNA_004796	7:69874055|69882364	ENSSSCG00000001958,	BAZ1A	13.25	0	-5.197743649	0.045579694
circRNA_014871	8:3351703|3360764	ENSSSCG00000027349,	TBC1D14	13.25	0	-5.197743649	0.045579694
circRNA_014788	13:209723825|209726611	ENSSSCG00000028883,	SETD4	13.25	0	-5.197743649	0.045579694
circRNA_014798	15:35514849|35544893	n/a	_	13.25	0	-5.197743649	0.045579694
circRNA_003937	2:82120327|82137621	ENSSSCG00000014044,	NSD1	13.25	0	-5.197743649	0.045579694
circRNA_014787	13:202682827|202698955	ENSSSCG00000027594,	USP16	13.75	0	-5.177042918	0.046475255
circRNA_014850	6:69145548|69154853	ENSSSCG00000003462,	DDI2	13.75	0	-5.177042918	0.046475255
circRNA_014899	X:70498623|70639982	ENSSSCG00000012434,	-	13.75	0	-5.177042918	0.046475255
circRNA_003484	15:3788416|3806724	ENSSSCG00000015668,	ORC4	13.75	0	-5.177042918	0.046475255
circRNA_014764	1:163944258|163953086	ENSSSCG00000004859,	ZNF516	13.75	0	-5.177042918	0.046475255
circRNA_014832	3:128268804|128273499	ENSSSCG00000008614,	SMC6	13.75	0	-5.177042918	0.046475255
circRNA_012635	14:34675990|34705614	ENSSSCG00000009834,	ATXN2	13.75	0	-5.177042918	0.046475255
circRNA_002272	9:101637290|101643291	ENSSSCG00000015387,	TRA2A	13.75	0	-5.177042918	0.046475255
circRNA_009363	3:59005137|59006428	ENSSSCG00000008192,	TMEM131	13.75	0	-5.177042918	0.046475255
circRNA_006765	1:257051731|257059164	ENSSSCG00000005282,	VPS13A	13.75	0	-5.177042918	0.046475255
circRNA_014874	8:44840811|44860365	n/a	_	13.75	0	-5.177042918	0.046475255
circRNA_009288	2:118428431|118454133	ENSSSCG00000014191,	-	13.75	0	-5.177042918	0.046475255
circRNA_001868	6:99489396|99547687	ENSSSCG00000025478,	MIB1	193.75	59.25	-1.250197027	0.046483845
circRNA_000993	15:115960373|116031233	n/a	_	300	381.75	1.016424643	0.046535556
circRNA_004429	5:37153142|37173822	ENSSSCG00000000502,	CNOT2	13.25	0	-5.16833927	0.046901512
circRNA_014789	13:212111311|212112404	ENSSSCG00000022318,	ERG	13.25	0	-5.16833927	0.046901512
circRNA_014792	14:60101240|60137643	ENSSSCG00000010156,	ARID4B	13.25	0	-5.16833927	0.046901512
circRNA_002770	10:15048938|15049680	ENSSSCG00000024484,	DEGS1	13.25	0	-5.16833927	0.046901512
circRNA_014826	3:34715827|34716828	ENSSSCG00000007907,	USP7	13.25	0	-5.16833927	0.046901512
circRNA_014815	2:17348985|17353410	ENSSSCG00000013262,	AMBRA1	13.25	0	-5.16833927	0.046901512
circRNA_002071	8:33236820|33237635	ENSSSCG00000022168,	APBB2	13.25	0	-5.16833927	0.046901512
circRNA_010284	10:46613551|46681760	ENSSSCG00000011025,	-	13.25	0	-5.16833927	0.046901512
circRNA_014879	8:86231350|86247013	ENSSSCG00000030044,	-	13.25	0	-5.16833927	0.046901512
circRNA_014821	2:128516665|128530836	ENSSSCG00000027857,	DMXL1	13.25	0	-5.16833927	0.046901512
circRNA_005453	1:136475794|136477100	ENSSSCG00000004653,	SECISBP2L	13.25	0	-5.16833927	0.046901512
circRNA_009492	4:112825591|112827827	ENSSSCG00000006727,	WDR3	13.25	0	-5.16833927	0.046901512
circRNA_011637	2:140494862|140515197	ENSSSCG00000014283,	KIF3A	11	0	-5.160109602	0.04728765
circRNA_011590	2:50783412|50808396	ENSSSCG00000013402,	-	11	0	-5.160109602	0.04728765
circRNA_014731	8:63565251|63594474	n/a	_	11	0	-5.160109602	0.04728765
circRNA_007044	13:126877865|126894393	ENSSSCG00000011765,	-	11	0	-5.160109602	0.04728765
circRNA_007367	17:57066927|57068443	ENSSSCG00000007460,	ARFGEF2	11	0	-5.160109602	0.04728765
circRNA_007505	2:112591635|112593124	ENSSSCG00000025287,	GIN1	11	0	-5.160109602	0.04728765
circRNA_009193	18:40626773|40659540	ENSSSCG00000016656,	-	11	0	-5.160109602	0.04728765
circRNA_007346	17:19717427|19768679	ENSSSCG00000007056,	PLCB1	11	0	-5.160109602	0.04728765
circRNA_014738	9:13614978|13620342	ENSSSCG00000014887,	-	11	0	-5.160109602	0.04728765
circRNA_007097	14:13477887|13486104	ENSSSCG00000009679,	FZD3	14	0	-5.151011435	0.04770611
circRNA_010327	12:23727465|23739114	ENSSSCG00000017513,	NPEPPS	14	0	-5.151011435	0.04770611
circRNA_007098	14:14027675|14028151	ENSSSCG00000009682,	HMBOX1	14	0	-5.151011435	0.04770611
circRNA_014884	9:25673758|25676436	ENSSSCG00000027093,	FOLH1B	14	0	-5.151011435	0.04770611
circRNA_008135	9:81539754|81544896	ENSSSCG00000015327,	CASD1	14	0	-5.151011435	0.04770611
circRNA_009041	15:92657331|92657618	ENSSSCG00000023983,	AGPS	14	0	-5.151011435	0.04770611
circRNA_004778	7:62827072|62836071	ENSSSCG00000001878,	PTPN9	10.5	0	-5.145623157	0.047958987
circRNA_014668	15:156203226|156310381	n/a	_	10.5	0	-5.145623157	0.047958987

circRNA_014719	6:82991958|82992781	ENSSSCG00000021457,	ZBTB8A	10.5	0	-5.145623157	0.047958987
circRNA_010933	X:60908671|61023192	n/a	_	10.5	0	-5.145623157	0.047958987
circRNA_006194	5:16670864|16688220	ENSSSCG00000028516,	LARP4	10.5	0	-5.145623157	0.047958987
circRNA_001742	5:73490736|73508782	ENSSSCG00000000779,	KIF21A	10.5	0	-5.145623157	0.047958987
circRNA_014638	13:81371686|81375200	ENSSSCG00000011629,	ACAD11	10.5	0	-5.145623157	0.047958987
circRNA_005255	GL894864.1:45296|55859	ENSSSCG00000026147,	-	11.75	0	-5.141098393	0.048170275
circRNA_001525	3:126269305|126285991	ENSSSCG00000008604,	WDR35	11.75	0	-5.141098393	0.048170275
circRNA_001127	17:57540295|57575864	n/a	_	11.75	0	-5.141098393	0.048170275
circRNA_004044	2:150641324|150660330	ENSSSCG00000014399,	ARHGAP26	11.75	0	-5.141098393	0.048170275
circRNA_010630	3:100584279|100593451	ENSSSCG00000008444,	PRKCE	11.75	0	-5.141098393	0.048170275
circRNA_014643	13:108395217|108395772	ENSSSCG00000011731,	SMC4	11.75	0	-5.141098393	0.048170275
circRNA_014637	13:74688185|74736227	ENSSSCG00000011575,	ATG7	11.75	0	-5.141098393	0.048170275
circRNA_003482	14:147294220|147352065	ENSSSCG00000028253,	-	11.75	0	-5.141098393	0.048170275
circRNA_014684	2:95754776|95757053	ENSSSCG00000029382,	-	11.75	0	-5.141098393	0.048170275
circRNA_014665	15:101687076|101693050	ENSSSCG00000016027,	ITGAV	11.75	0	-5.141098393	0.048170275
circRNA_014769	10:11694054|11696196	ENSSSCG00000010821,	EPRS	13.25	0	-5.136876431	0.048368114
circRNA_011254	13:126563388|126579257	n/a	_	13.25	0	-5.136876431	0.048368114
circRNA_005120	9:57693529|57730623	ENSSSCG00000015206,	CCDC15	13.25	0	-5.136876431	0.048368114
circRNA_006537	GL896152.2:40782|42458	n/a	_	11.25	0	-5.133905233	0.048507742
circRNA_005483	1:202611390|202619041	ENSSSCG00000005038,	PSMC6	11.25	0	-5.133905233	0.048507742
circRNA_005232	GL893560.1:9170|12365	ENSSSCG00000009674,	-	11.25	0	-5.133905233	0.048507742
circRNA_014623	10:54023860|54031658	ENSSSCG00000011066,	YME1L1	11.25	0	-5.133905233	0.048507742
circRNA_009899	8:134701991|134715585	ENSSSCG00000009193,	SMARCAD1	11.25	0	-5.133905233	0.048507742
circRNA_014746	9:100572778|100599583	ENSSSCG00000015383,	RAPGEF5	11.25	0	-5.133905233	0.048507742
circRNA_014683	2:91601749|91654443	ENSSSCG00000014127,	RASGRF2	11.25	0	-5.133905233	0.048507742
circRNA_014621	10:16947860|16952536	ENSSSCG00000010862,	CDC42BPA	11.25	0	-5.133905233	0.048507742
circRNA_014628	11:21001197|21003912	ENSSSCG00000009407,	ESD	11.25	0	-5.133905233	0.048507742
circRNA_014702	4:117893543|117957301	ENSSSCG00000006779,	ST7L	12.5	0	-5.111003679	0.049595133
circRNA_009523	5:16400508|16402243	ENSSSCG00000000217,	-	12.5	0	-5.111003679	0.049595133
circRNA_011876	6:65200370|65203979	ENSSSCG00000003413,	MTOR	12.5	0	-5.111003679	0.049595133
circRNA_001297	2:87512163|87514471	ENSSSCG00000014094,	AGGF1	12.5	0	-5.111003679	0.049595133
circRNA_014698	3:135328996|135350662	ENSSSCG00000008642,	ASAP2	12.5	0	-5.111003679	0.049595133
circRNA_000297	10:24484010|24486223	ENSSSCG00000010894,	TP53BP2	12.5	0	-5.111003679	0.049595133
circRNA_005100	9:40527988|40532789	ENSSSCG00000029198,	CUL5	12.5	0	-5.111003679	0.049595133
circRNA_009462	4:56554006|56556388	ENSSSCG00000006146,	-	12.5	0	-5.111003679	0.049595133
circRNA_000560	13:34006058|34020867	n/a	_	12.5	0	-5.111003679	0.049595133
circRNA_014652	14:20942251|21103601	n/a	_	12.5	0	-5.111003679	0.049595133
circRNA_010201	1:124967959|124977239	ENSSSCG00000004589,	RNF111	12.5	0	-5.111003679	0.049595133
circRNA_014656	14:60727790|60746720	ENSSSCG00000010160,	TARBP1	12.5	0	-5.111003679	0.049595133
circRNA_013473	9:85856790|85879451	ENSSSCG00000015345,	GLCCI1	66	8.5	-2.703905944	0.049748673

Table S3-1. GO analysis of the host genes for differentially expressed circRNAs in LH vs. LL.

Gene Ontology term	Term_type	Cluster frequency	Genome frequency of use	Corrected *P*value	Genes annotated to the term
catalytic activity	molecular_function	30 out of 52 genes, 57.7%	868 out of 3284 genes, 26.4%	9.30E-05	ENSSSCG00000015874, ENSSSCG00000015840, ENSSSCG00000012649, ENSSSCG00000012583, ENSSSCG00000015844, ENSSSCG00000026044, ENSSSCG00000009223, ENSSSCG00000003815, ENSSSCG00000027342, ENSSSCG00000004845, ENSSSCG00000011102, ENSSSCG00000010548, ENSSSCG00000024628, ENSSSCG00000008842, ENSSSCG00000029621, ENSSSCG00000017670, ENSSSCG00000011213, ENSSSCG00000009994, ENSSSCG00000011229, ENSSSCG00000013332, ENSSSCG00000003776, ENSSSCG00000001092, ENSSSCG00000005970, ENSSSCG00000009230, ENSSSCG00000008339, ENSSSCG00000011794, ENSSSCG00000008247, ENSSSCG00000006878, ENSSSCG00000006127, ENSSSCG00000011183

cofactor transporter activity	molecular_function	3 out of 52 genes, 5.8%	4 out of 3284 genes, 0.1%	0.00087	ENSSSCG00000015590, ENSSSCG00000025316, ENSSSCG00000011498
pyrophosphatase activity	molecular_function	7 out of 52 genes, 13.5%	54 out of 3284 genes, 1.6%	0.00097	ENSSSCG00000011213, ENSSSCG00000012649, ENSSSCG00000015840, ENSSSCG00000013332, ENSSSCG00000009223, ENSSSCG00000006127, ENSSSCG00000011183
hydrolase activity, acting on acid anhydrides, in phosphorus-containing anhydrides	molecular_function	7 out of 52 genes, 13.5%	55 out of 3284 genes, 1.7%	0.0011	ENSSSCG00000009223, ENSSSCG00000011183, ENSSSCG00000006127, ENSSSCG00000012649, ENSSSCG00000011213, ENSSSCG00000015840, ENSSSCG00000013332
protein kinase activity	molecular_function	7 out of 52 genes, 13.5%	57 out of 3284 genes, 1.7%	0.0014	ENSSSCG00000010548, ENSSSCG00000011102, ENSSSCG00000027342, ENSSSCG00000029621, ENSSSCG00000008842, ENSSSCG00000017670, ENSSSCG00000015874
hydrolase activity, acting on acid anhydrides	molecular_function	7 out of 52 genes, 13.5%	57 out of 3284 genes, 1.7%	0.0014	ENSSSCG00000013332, ENSSSCG00000015840, ENSSSCG00000012649, ENSSSCG00000011213, ENSSSCG00000011183, ENSSSCG00000006127, ENSSSCG00000009223
nucleoside-triphosphatase activity	molecular_function	6 out of 52 genes, 11.5%	41 out of 3284 genes, 1.2%	0.00203	ENSSSCG00000012649, ENSSSCG00000011213, ENSSSCG00000015840, ENSSSCG00000013332, ENSSSCG00000011183, ENSSSCG00000006127
ATPase activity, coupled	molecular_function	5 out of 52 genes, 9.6%	28 out of 3284 genes, 0.9%	0.0036	ENSSSCG00000013332, ENSSSCG00000011213, ENSSSCG00000012649, ENSSSCG00000011183, ENSSSCG00000006127
ATPase activity	molecular_function	5 out of 52 genes, 9.6%	31 out of 3284 genes, 0.9%	0.006	ENSSSCG00000006127, ENSSSCG00000011183, ENSSSCG00000012649, ENSSSCG00000011213, ENSSSCG00000013332
protein binding	molecular_function	15 out of 52 genes, 28.8%	329 out of 3284 genes, 10.0%	0.00624	ENSSSCG00000006127, ENSSSCG00000026283, ENSSSCG00000016027, ENSSSCG00000006201, ENSSSCG00000000625, ENSSSCG00000012112, ENSSSCG00000000985, ENSSSCG00000013776, ENSSSCG00000027342, ENSSSCG00000012434, ENSSSCG00000008499, ENSSSCG00000017836, ENSSSCG00000001818, ENSSSCG00000016185, ENSSSCG00000004505
transmembrane receptor protein kinase activity	molecular_function	4 out of 52 genes, 7.7%	20 out of 3284 genes, 0.6%	0.01325	ENSSSCG00000011102, ENSSSCG00000029621, ENSSSCG00000008842, ENSSSCG00000015874
coenzyme transporter activity	molecular_function	2 out of 52 genes, 3.8%	2 out of 3284 genes, 0.1%	0.01451	ENSSSCG00000011498, ENSSSCG00000025316
binding	molecular_function	19 out of 52 genes, 36.5%	545 out of 3284 genes, 16.6%	0.0223	ENSSSCG00000001818, ENSSSCG00000027342, ENSSSCG00000008499, ENSSSCG00000028563, ENSSSCG00000012112, ENSSSCG00000000985, ENSSSCG00000026283, ENSSSCG00000017836, ENSSSCG00000016185, ENSSSCG00000004505, ENSSSCG00000012434, ENSSSCG00000026158, ENSSSCG00000000625, ENSSSCG00000013241, ENSSSCG00000013776, ENSSSCG00000006878, ENSSSCG00000006127, ENSSSCG00000016027, ENSSSCG00000006201
transferase activity	molecular_function	14 out of 52 genes, 26.9%	329 out of 3284 genes, 10.0%	0.02355	ENSSSCG00000029621, ENSSSCG00000008247, ENSSSCG00000024628, ENSSSCG00000008339, ENSSSCG00000008842, ENSSSCG00000027342, ENSSSCG00000003815, ENSSSCG00000009230, ENSSSCG00000010548, ENSSSCG00000011102, ENSSSCG00000015874, ENSSSCG00000017670, ENSSSCG00000011229, ENSSSCG00000026044
phosphotransferase activity, alcohol group as acceptor	molecular_function	7 out of 52 genes, 13.5%	89 out of 3284 genes, 2.7%	0.02479	ENSSSCG00000027342, ENSSSCG00000029621, ENSSSCG00000010548, ENSSSCG00000011102, ENSSSCG00000017670, ENSSSCG00000015874, ENSSSCG00000008842
cell	cellular_component	26 out of 27 genes, 96.3%	605 out of 3284 genes, 18.4%	8.18E-17	ENSSSCG00000009047, ENSSSCG00000027342, ENSSSCG00000017836, ENSSSCG00000011794, ENSSSCG00000010625, ENSSSCG00000016185, ENSSSCG00000017277, ENSSSCG00000021380, ENSSSCG00000011207, ENSSSCG00000006874, ENSSSCG00000000912, ENSSSCG00000012112, ENSSSCG00000004620, ENSSSCG00000016027, ENSSSCG00000024887, ENSSSCG00000015840, ENSSSCG00000004893, ENSSSCG00000011853, ENSSSCG00000006084, ENSSSCG00000012649, ENSSSCG00000013776, ENSSSCG00000001242, ENSSSCG00000011229, ENSSSCG00000011213, ENSSSCG00000006127, ENSSSCG00000002799

cell part	cellular_component	26 out of 27 genes, 96.3%	605 out of 3284 genes, 18.4%	8.18E-17	ENSSSCG00000024887, ENSSSCG00000012112, ENSSSCG00000016027, ENSSSCG00000004620, ENSSSCG00000000912, ENSSSCG00000011207, ENSSSCG00000006874, ENSSSCG00000010625, ENSSSCG00000016185, ENSSSCG00000021380, ENSSSCG00000017277, ENSSSCG00000011794, ENSSSCG00000017836, ENSSSCG00000027342, ENSSSCG00000009047, ENSSSCG00000002799, ENSSSCG00000006127, ENSSSCG00000011213, ENSSSCG00000011229, ENSSSCG00000001242, ENSSSCG00000012649, ENSSSCG00000013776, ENSSSCG00000006084, ENSSSCG00000011853, ENSSSCG00000004893, ENSSSCG00000015840
intracellular part	cellular_component	22 out of 27 genes, 81.5%	485 out of 3284 genes, 14.8%	9.85E-13	ENSSSCG00000015840, ENSSSCG00000004893, ENSSSCG00000011853, ENSSSCG00000012649, ENSSSCG00000013776, ENSSSCG00000006084, ENSSSCG00000011229, ENSSSCG00000006127, ENSSSCG00000002799, ENSSSCG00000011213, ENSSSCG00000009047, ENSSSCG00000027342, ENSSSCG00000017836, ENSSSCG00000011794, ENSSSCG00000010625, ENSSSCG00000021380, ENSSSCG00000017277, ENSSSCG00000011207, ENSSSCG00000000912, ENSSSCG00000012112, ENSSSCG00000004620, ENSSSCG00000024887
intracellular	cellular_component	22 out of 27 genes, 81.5%	486 out of 3284 genes, 14.8%	1.03E-12	ENSSSCG00000012112, ENSSSCG00000004620, ENSSSCG00000024887, ENSSSCG00000011207, ENSSSCG00000000912, ENSSSCG00000011794, ENSSSCG00000010625, ENSSSCG00000021380, ENSSSCG00000017277, ENSSSCG00000009047, ENSSSCG00000027342, ENSSSCG00000017836, ENSSSCG00000006127, ENSSSCG00000011213, ENSSSCG00000002799, ENSSSCG00000011229, ENSSSCG00000011853, ENSSSCG00000012649, ENSSSCG00000013776, ENSSSCG00000006084, ENSSSCG00000015840, ENSSSCG00000004893
intracellular organelle	cellular_component	20 out of 27 genes, 74.1%	381 out of 3284 genes, 11.6%	3.65E-12	ENSSSCG00000011207, ENSSSCG00000004620, ENSSSCG00000012112, ENSSSCG00000027342, ENSSSCG00000017836, ENSSSCG00000009047, ENSSSCG00000021380, ENSSSCG00000017277, ENSSSCG00000010625, ENSSSCG00000011794, ENSSSCG00000011229, ENSSSCG00000002799, ENSSSCG00000006127, ENSSSCG00000011213, ENSSSCG00000004893, ENSSSCG00000015840, ENSSSCG00000013776, ENSSSCG00000012649, ENSSSCG00000006084, ENSSSCG00000011853
organelle	cellular_component	20 out of 27 genes, 74.1%	398 out of 3284 genes, 12.1%	8.59E-12	ENSSSCG00000011794, ENSSSCG00000021380, ENSSSCG00000017277, ENSSSCG00000010625, ENSSSCG00000009047, ENSSSCG00000027342, ENSSSCG00000017836, ENSSSCG00000004620, ENSSSCG00000012112, ENSSSCG00000011207, ENSSSCG00000011853, ENSSSCG00000012649, ENSSSCG00000013776, ENSSSCG00000006084, ENSSSCG00000015840, ENSSSCG00000004893, ENSSSCG00000006127, ENSSSCG00000011213, ENSSSCG00000002799, ENSSSCG00000011229
organelle part	cellular_component	19 out of 27 genes, 70.4%	342 out of 3284 genes, 10.4%	9.93E-12	ENSSSCG00000012649, ENSSSCG00000006084, ENSSSCG00000013776, ENSSSCG00000004893, ENSSSCG00000015840, ENSSSCG00000002799, ENSSSCG00000006127, ENSSSCG00000011213, ENSSSCG00000011229, ENSSSCG00000010625, ENSSSCG00000021380, ENSSSCG00000017277, ENSSSCG00000011794, ENSSSCG00000017836, ENSSSCG00000027342, ENSSSCG00000009047, ENSSSCG00000012112, ENSSSCG00000004620, ENSSSCG00000011207
intracellular organelle part	cellular_component	18 out of 27 genes, 66.7%	321 out of 3284 genes, 9.8%	6.30E-11	ENSSSCG00000021380, ENSSSCG00000010625, ENSSSCG00000011794, ENSSSCG00000017836, ENSSSCG00000027342, ENSSSCG00000009047, ENSSSCG00000004620, ENSSSCG00000012112, ENSSSCG00000011207, ENSSSCG00000006084, ENSSSCG00000012649, ENSSSCG00000013776, ENSSSCG00000004893, ENSSSCG00000015840, ENSSSCG00000011213, ENSSSCG00000006127, ENSSSCG00000002799, ENSSSCG00000011229
membrane-bounded organelle	cellular_component	15 out of 27 genes, 55.6%	291 out of 3284 genes, 8.9%	5.42E-08	ENSSSCG00000011853, ENSSSCG00000011794, ENSSSCG00000012649, ENSSSCG00000010625, ENSSSCG00000006084, ENSSSCG00000013776, ENSSSCG00000021380, ENSSSCG00000015840, ENSSSCG00000009047, ENSSSCG00000027342, ENSSSCG00000004893, ENSSSCG00000004620, ENSSSCG00000002799, ENSSSCG00000006127, ENSSSCG00000011229
intracellular membrane-bounded organelle	cellular_component	13 out of 27 genes, 48.1%	241 out of 3284 genes, 7.3%	7.46E-07	ENSSSCG00000011229, ENSSSCG00000002799, ENSSSCG00000006127, ENSSSCG00000027342, ENSSSCG00000004893, ENSSSCG00000009047, ENSSSCG00000015840, ENSSSCG00000013776, ENSSSCG00000012649, ENSSSCG00000006084, ENSSSCG00000010625, ENSSSCG00000021380, ENSSSCG00000011794
macromolecular complex	cellular_component	14 out of 27 genes, 51.9%	388 out of 3284 genes, 11.8%	2.79E-05	ENSSSCG00000006127, ENSSSCG00000024887, ENSSSCG00000016027, ENSSSCG00000000912, ENSSSCG00000011229, ENSSSCG00000001242, ENSSSCG00000011207, ENSSSCG00000013776, ENSSSCG00000012649, ENSSSCG00000010625, ENSSSCG00000021380, ENSSSCG00000011794, ENSSSCG00000015840, ENSSSCG00000009047
nuclear part	cellular_component	9 out of 27 genes, 33.3%	129 out of 3284 genes, 3.9%	3.12E-05	ENSSSCG00000006127, ENSSSCG00000013776, ENSSSCG00000006084, ENSSSCG00000010625, ENSSSCG00000012649, ENSSSCG00000011794, ENSSSCG00000027342, ENSSSCG00000015840, ENSSSCG00000009047

nucleus	cellular_component	9 out of 27 genes, 33.3%	133 out of 3284 genes, 4.0%	4.06E-05	ENSSSCG00000013776, ENSSSCG00000006084, ENSSSCG00000012649, ENSSSCG00000010625, ENSSSCG00000011794, ENSSSCG00000027342, ENSSSCG00000009047, ENSSSCG00000015840, ENSSSCG00000006127
non-membrane-bounded organelle	cellular_component	9 out of 27 genes, 33.3%	152 out of 3284 genes, 4.6%	0.00012	ENSSSCG00000011207, ENSSSCG00000006127, ENSSSCG00000011213, ENSSSCG00000004620, ENSSSCG00000012112, ENSSSCG00000017836, ENSSSCG00000027342, ENSSSCG00000017277, ENSSSCG00000010625
intracellular non-membrane-bounded organelle	cellular_component	9 out of 27 genes, 33.3%	152 out of 3284 genes, 4.6%	0.00012	ENSSSCG00000011207, ENSSSCG00000004620, ENSSSCG00000012112, ENSSSCG00000011213, ENSSSCG00000006127, ENSSSCG00000017836, ENSSSCG00000027342, ENSSSCG00000017277, ENSSSCG00000010625
nuclear lumen	cellular_component	6 out of 27 genes, 22.2%	80 out of 3284 genes, 2.4%	0.00245	ENSSSCG00000011794, ENSSSCG00000010625, ENSSSCG00000006127, ENSSSCG00000006084, ENSSSCG00000009047, ENSSSCG00000027342
chromosomal part	cellular_component	5 out of 27 genes, 18.5%	49 out of 3284 genes, 1.5%	0.00272	ENSSSCG00000011213, ENSSSCG00000006127, ENSSSCG00000010625, ENSSSCG00000011207, ENSSSCG00000027342
chromosome	cellular_component	5 out of 27 genes, 18.5%	54 out of 3284 genes, 1.6%	0.00439	ENSSSCG00000010625, ENSSSCG00000006127, ENSSSCG00000011213, ENSSSCG00000027342, ENSSSCG00000011207
cytoplasmic part	cellular_component	9 out of 27 genes, 33.3%	249 out of 3284 genes, 7.6%	0.00718	ENSSSCG00000011229, ENSSSCG00000000912, ENSSSCG00000002799, ENSSSCG00000004620, ENSSSCG00000004893, ENSSSCG00000017836, ENSSSCG00000017277, ENSSSCG00000021380, ENSSSCG00000011853
cytoplasm	cellular_component	9 out of 27 genes, 33.3%	251 out of 3284 genes, 7.6%	0.00765	ENSSSCG00000000912, ENSSSCG00000011229, ENSSSCG00000004620, ENSSSCG00000002799, ENSSSCG00000017836, ENSSSCG00000004893, ENSSSCG00000011853, ENSSSCG00000021380, ENSSSCG00000017277
intracellular organelle lumen	cellular_component	6 out of 27 genes, 22.2%	100 out of 3284 genes, 3.0%	0.00869	ENSSSCG00000027342, ENSSSCG00000009047, ENSSSCG00000010625, ENSSSCG00000006127, ENSSSCG00000006084, ENSSSCG00000011794
membrane-enclosed lumen	cellular_component	6 out of 27 genes, 22.2%	101 out of 3284 genes, 3.1%	0.00919	ENSSSCG00000011794, ENSSSCG00000006084, ENSSSCG00000006127, ENSSSCG00000010625, ENSSSCG00000009047, ENSSSCG00000027342
organelle lumen	cellular_component	6 out of 27 genes, 22.2%	101 out of 3284 genes, 3.1%	0.00919	ENSSSCG00000009047, ENSSSCG00000027342, ENSSSCG00000011794, ENSSSCG00000006127, ENSSSCG00000006084, ENSSSCG00000010625
supramolecular complex	cellular_component	4 out of 27 genes, 14.8%	35 out of 3284 genes, 1.1%	0.01148	ENSSSCG00000017277, ENSSSCG00000012112, ENSSSCG00000004620, ENSSSCG00000017836
supramolecular polymer	cellular_component	4 out of 27 genes, 14.8%	35 out of 3284 genes, 1.1%	0.01148	ENSSSCG00000017836, ENSSSCG00000012112, ENSSSCG00000004620, ENSSSCG00000017277
supramolecular fiber	cellular_component	4 out of 27 genes, 14.8%	35 out of 3284 genes, 1.1%	0.01148	ENSSSCG00000017277, ENSSSCG00000004620, ENSSSCG00000012112, ENSSSCG00000017836
ISWI-type complex	cellular_component	2 out of 27 genes, 7.4%	3 out of 3284 genes, 0.1%	0.01399	ENSSSCG00000009047, ENSSSCG00000012649
polymeric cytoskeletal fiber	cellular_component	3 out of 27 genes, 11.1%	15 out of 3284 genes, 0.5%	0.01521	ENSSSCG00000004620, ENSSSCG00000012112, ENSSSCG00000017836
cohesin complex	cellular_component	2 out of 27 genes, 7.4%	4 out of 3284 genes, 0.1%	0.02784	ENSSSCG00000011207, ENSSSCG00000010625
biological regulation	biological_process	55 out of 96 genes, 57.3%	1250 out of 3284 genes, 38.1%	0.04714	ENSSSCG00000012583, ENSSSCG00000014114, ENSSSCG00000006201, ENSSSCG00000008339, ENSSSCG00000009047, ENSSSCG00000006035, ENSSSCG00000008614, ENSSSCG00000017670, ENSSSCG00000025551, ENSSSCG00000011632, ENSSSCG00000011794, ENSSSCG00000011853, ENSSSCG00000029252, ENSSSCG00000009480, ENSSSCG00000004505, ENSSSCG00000007143,

	ENSSSCG00000010835, ENSSSCG00000000488, ENSSSCG00000001062, ENSSSCG00000008842, ENSSSCG00000006127, ENSSSCG00000001818, ENSSSCG00000008866, ENSSSCG00000011681, ENSSSCG00000028780, ENSSSCG00000024887, ENSSSCG00000003520, ENSSSCG00000012112, ENSSSCG00000007153, ENSSSCG00000011518, ENSSSCG00000004753, ENSSSCG00000010625, ENSSSCG00000002755, ENSSSCG00000015590, ENSSSCG00000010081, ENSSSCG00000011102, ENSSSCG00000007460, ENSSSCG00000011025, ENSSSCG00000011467, ENSSSCG00000028840, ENSSSCG00000025478, ENSSSCG00000005027, ENSSSCG00000014009, ENSSSCG00000004620, ENSSSCG00000004595, ENSSSCG00000016027, ENSSSCG00000011183, ENSSSCG00000013241, ENSSSCG00000026158, ENSSSCG00000012649, ENSSSCG00000007030, ENSSSCG00000010593, ENSSSCG00000016185, ENSSSCG00000000625, ENSSSCG00000007056

Table S3-2. GO analysis of the host genes for differentially expressed circRNAs in FH vs. FL.

Gene Ontology term	Term_type	Cluster frequency	Genome frequency of use	Corrected *P*value	Genes annotated to the term
catalytic activity	molecular_function	28 out of 50 genes, 56.0%	868 out of 3284 genes, 26.4%	0.00059	ENSSSCG00000006864, ENSSSCG00000011183, ENSSSCG00000011794, ENSSSCG00000017670, ENSSSCG00000014283, ENSSSCG00000015874, ENSSSCG00000011213, ENSSSCG00000006872, ENSSSCG00000014893, ENSSSCG00000011575, ENSSSCG00000006983, ENSSSCG00000021170, ENSSSCG00000005243, ENSSSCG00000013073, ENSSSCG00000006911, ENSSSCG00000016173, ENSSSCG00000005215, ENSSSCG00000011001, ENSSSCG00000009994, ENSSSCG00000009407, ENSSSCG00000025427, ENSSSCG00000000477, ENSSSCG00000015844, ENSSSCG00000015576, ENSSSCG00000007474, ENSSSCG00000017799, ENSSSCG00000005135, ENSSSCG00000012116
hydrolase activity	molecular_function	13 out of 50 genes, 26.0%	254 out of 3284 genes, 7.7%	0.00524	ENSSSCG00000011794, ENSSSCG00000009407, ENSSSCG00000009994, ENSSSCG00000016173, ENSSSCG00000011001, ENSSSCG00000011183, ENSSSCG00000006864, ENSSSCG00000006872, ENSSSCG00000011213, ENSSSCG00000014283, ENSSSCG00000000477, ENSSSCG00000017799, ENSSSCG00000006983
cell	cellular_component	38 out of 39 genes, 97.4%	605 out of 3284 genes, 18.4%	1.24E-25	ENSSSCG00000011201, ENSSSCG00000013602, ENSSSCG00000011845, ENSSSCG00000006864, ENSSSCG00000004927, ENSSSCG00000016027, ENSSSCG00000005243, ENSSSCG00000004620, ENSSSCG00000024927, ENSSSCG00000004524, ENSSSCG00000001924, ENSSSCG00000016916, ENSSSCG00000015576, ENSSSCG00000014283, ENSSSCG00000016077, ENSSSCG00000010189, ENSSSCG00000001563, ENSSSCG00000006084, ENSSSCG00000009217, ENSSSCG00000007088, ENSSSCG00000017301, ENSSSCG00000008573, ENSSSCG00000011213, ENSSSCG00000002799, ENSSSCG00000017277, ENSSSCG00000001958, ENSSSCG00000010625, ENSSSCG00000006874, ENSSSCG00000029382, ENSSSCG00000004792, ENSSSCG00000011794, ENSSSCG00000016853, ENSSSCG00000009047, ENSSSCG00000000477, ENSSSCG00000000217, ENSSSCG00000005065, ENSSSCG00000015335, ENSSSCG00000000912
cell part	cellular_component	38 out of 39 genes, 97.4%	605 out of 3284 genes, 18.4%	1.24E-25	ENSSSCG00000007088, ENSSSCG00000017301, ENSSSCG00000001563, ENSSSCG00000009217, ENSSSCG00000006084, ENSSSCG00000002799, ENSSSCG00000008573, ENSSSCG00000011213, ENSSSCG00000014283, ENSSSCG00000015576, ENSSSCG00000010189, ENSSSCG00000016077, ENSSSCG00000016916, ENSSSCG00000001924, ENSSSCG00000004620, ENSSSCG00000005243, ENSSSCG00000024927, ENSSSCG00000004524, ENSSSCG00000006864, ENSSSCG00000004927, ENSSSCG00000016027, ENSSSCG00000011845, ENSSSCG00000013602, ENSSSCG00000011201, ENSSSCG00000015335, ENSSSCG00000005065, ENSSSCG00000000912, ENSSSCG00000009047, ENSSSCG00000016853, ENSSSCG00000000477, ENSSSCG00000000217, ENSSSCG00000011794, ENSSSCG00000029382, ENSSSCG00000004792, ENSSSCG00000006874, ENSSSCG00000010625, ENSSSCG00000001958, ENSSSCG00000017277
intracellular part	cellular_component	35 out of 39 genes, 89.7%	485 out of 3284 genes, 14.8%	1.11E-23	ENSSSCG00000009217, ENSSSCG00000006084, ENSSSCG00000001563, ENSSSCG00000007088, ENSSSCG00000017301, ENSSSCG00000008573, ENSSSCG00000011213, ENSSSCG00000002799, ENSSSCG00000015576, ENSSSCG00000014283, ENSSSCG00000016077, ENSSSCG00000010189, ENSSSCG00000001924, ENSSSCG00000005243, ENSSSCG00000004620, ENSSSCG00000024927, ENSSSCG00000004524, ENSSSCG00000004927, ENSSSCG00000006864, ENSSSCG00000013602, ENSSSCG00000011845, ENSSSCG00000011201, ENSSSCG00000005065, ENSSSCG00000015335, ENSSSCG00000000912, ENSSSCG00000016853, ENSSSCG00000009047, ENSSSCG00000000477, ENSSSCG00000000217, ENSSSCG00000011794, ENSSSCG00000029382, ENSSSCG00000004792, ENSSSCG00000010625, ENSSSCG00000001958, ENSSSCG00000017277

intracellular	cellular_component	35 out of 39 genes, 89.7%	486 out of 3284 genes, 14.8%	1.20E-23	ENSSSCG00000006864, ENSSSCG00000004927, ENSSSCG00000005243, ENSSSCG00000004620, ENSSSCG00000004524, ENSSSCG00000024927, ENSSSCG00000011201, ENSSSCG00000013602, ENSSSCG00000011845, ENSSSCG00000015576, ENSSSCG00000014283, ENSSSCG00000016077, ENSSSCG00000010189, ENSSSCG00000009217, ENSSSCG00000001563, ENSSSCG00000006084, ENSSSCG00000007088, ENSSSCG00000017301, ENSSSCG00000008573, ENSSSCG00000011213, ENSSSCG00000002799, ENSSSCG00000001924, ENSSSCG00000010625, ENSSSCG00000001958, ENSSSCG00000029382, ENSSSCG00000004792, ENSSSCG00000017277, ENSSSCG00000005065, ENSSSCG00000015335, ENSSSCG00000000912, ENSSSCG00000011794, ENSSSCG00000016853, ENSSSCG00000009047, ENSSSCG00000000477, ENSSSCG00000000217
intracellular organelle	cellular_component	29 out of 39 genes, 74.4%	381 out of 3284 genes, 11.6%	4.98E-18	ENSSSCG00000013602, ENSSSCG00000017277, ENSSSCG00000011201, ENSSSCG00000024927, ENSSSCG00000004524, ENSSSCG00000004620, ENSSSCG00000005243, ENSSSCG00000006864, ENSSSCG00000010625, ENSSSCG00000004927, ENSSSCG00000001958, ENSSSCG00000000477, ENSSSCG00000000217, ENSSSCG00000009047, ENSSSCG00000001924, ENSSSCG00000011794, ENSSSCG00000002799, ENSSSCG00000008573, ENSSSCG00000011213, ENSSSCG00000007088, ENSSSCG00000017301, ENSSSCG00000009217, ENSSSCG00000006084, ENSSSCG00000001563, ENSSSCG00000010189, ENSSSCG00000016077, ENSSSCG00000014283, ENSSSCG00000015335, ENSSSCG00000015576
organelle	cellular_component	29 out of 39 genes, 74.4%	398 out of 3284 genes, 12.1%	1.75E-17	ENSSSCG00000001924, ENSSSCG00000011794, ENSSSCG00000000217, ENSSSCG00000000477, ENSSSCG00000009047, ENSSSCG00000016077, ENSSSCG00000010189, ENSSSCG00000015576, ENSSSCG00000015335, ENSSSCG00000014283, ENSSSCG00000008573, ENSSSCG00000011213, ENSSSCG00000002799, ENSSSCG00000009217, ENSSSCG00000001563, ENSSSCG00000006084, ENSSSCG00000017301, ENSSSCG00000007088, ENSSSCG00000011201, ENSSSCG00000017277, ENSSSCG00000013602, ENSSSCG00000010625, ENSSSCG00000006864, ENSSSCG00000001958, ENSSSCG00000004927, ENSSSCG00000004524, ENSSSCG00000024927, ENSSSCG00000005243, ENSSSCG00000004620
organelle part	cellular_component	27 out of 39 genes, 69.2%	342 out of 3284 genes, 10.4%	1.17E-16	ENSSSCG00000001924, ENSSSCG00000011794, ENSSSCG00000000477, ENSSSCG00000000217, ENSSSCG00000009047, ENSSSCG00000010189, ENSSSCG00000014283, ENSSSCG00000015335, ENSSSCG00000015576, ENSSSCG00000002799, ENSSSCG00000011213, ENSSSCG00000008573, ENSSSCG00000007088, ENSSSCG00000017301, ENSSSCG00000006084, ENSSSCG00000009217, ENSSSCG00000001563, ENSSSCG00000011201, ENSSSCG00000013602, ENSSSCG00000017277, ENSSSCG00000006864, ENSSSCG00000010625, ENSSSCG00000004927, ENSSSCG00000001958, ENSSSCG00000004524, ENSSSCG00000004620, ENSSSCG00000005243
intracellular organelle part	cellular_component	25 out of 39 genes, 64.1%	321 out of 3284 genes, 9.8%	8.36E-15	ENSSSCG00000011794, ENSSSCG00000001924, ENSSSCG00000009047, ENSSSCG00000000217, ENSSSCG00000000477, ENSSSCG00000015576, ENSSSCG00000014283, ENSSSCG00000015335, ENSSSCG00000010189, ENSSSCG00000006084, ENSSSCG00000009217, ENSSSCG00000001563, ENSSSCG00000017301, ENSSSCG00000007088, ENSSSCG00000011213, ENSSSCG00000008573, ENSSSCG00000002799, ENSSSCG00000011201, ENSSSCG00000013602, ENSSSCG00000001958, ENSSSCG00000006864, ENSSSCG00000010625, ENSSSCG00000004927, ENSSSCG00000004620, ENSSSCG00000004524
macromolecular complex	cellular_component	24 out of 39 genes, 61.5%	388 out of 3284 genes, 11.8%	1.10E-11	ENSSSCG00000009047, ENSSSCG00000016916, ENSSSCG00000016853, ENSSSCG00000000217, ENSSSCG00000000477, ENSSSCG00000011794, ENSSSCG00000012116, ENSSSCG00000001563, ENSSSCG00000009217, ENSSSCG00000015335, ENSSSCG00000015576, ENSSSCG00000005065, ENSSSCG00000010189, ENSSSCG00000000912, ENSSSCG00000013602, ENSSSCG00000004792, ENSSSCG00000029382, ENSSSCG00000005243, ENSSSCG00000004524, ENSSSCG00000024927, ENSSSCG00000010625, ENSSSCG00000004927, ENSSSCG00000001958, ENSSSCG00000016027
membrane-bounded organelle	cellular_component	21 out of 39 genes, 53.8%	291 out of 3284 genes, 8.9%	4.78E-11	ENSSSCG00000015335, ENSSSCG00000015576, ENSSSCG00000010189, ENSSSCG00000016077, ENSSSCG00000007088, ENSSSCG00000001563, ENSSSCG00000009217, ENSSSCG00000006084, ENSSSCG00000002799, ENSSSCG00000008573, ENSSSCG00000011794, ENSSSCG00000009047, ENSSSCG00000000477, ENSSSCG00000004927, ENSSSCG00000010625, ENSSSCG00000001958, ENSSSCG00000004620, ENSSSCG00000004524, ENSSSCG00000024927, ENSSSCG00000011201, ENSSSCG00000013602

non-membrane-bounded organelle	cellular_component	14 out of 39 genes, 35.9%	152 out of 3284 genes, 4.6%	5.32E-08	ENSSSCG00000010189, ENSSSCG00000015335, ENSSSCG00000014283, ENSSSCG00000010625, ENSSSCG00000006864, ENSSSCG00000011213, ENSSSCG00000017301, ENSSSCG00000004620, ENSSSCG00000005243, ENSSSCG00000001924, ENSSSCG00000011201, ENSSSCG00000000477, ENSSSCG00000000217, ENSSSCG00000017277
intracellular non-membrane-bounded organelle	cellular_component	14 out of 39 genes, 35.9%	152 out of 3284 genes, 4.6%	5.32E-08	ENSSSCG00000000217, ENSSSCG00000017277, ENSSSCG00000000477, ENSSSCG00000011201, ENSSSCG00000001924, ENSSSCG00000004620, ENSSSCG00000017301, ENSSSCG00000005243, ENSSSCG00000011213, ENSSSCG00000015335, ENSSSCG00000014283, ENSSSCG00000010625, ENSSSCG00000006864, ENSSSCG00000010189
intracellular membrane-bounded organelle	cellular_component	16 out of 39 genes, 41.0%	241 out of 3284 genes, 7.3%	2.99E-07	ENSSSCG00000004524, ENSSSCG00000010625, ENSSSCG00000004927, ENSSSCG00000001958, ENSSSCG00000013602, ENSSSCG00000011201, ENSSSCG00000008573, ENSSSCG00000002799, ENSSSCG00000006084, ENSSSCG00000001563, ENSSSCG00000009217, ENSSSCG00000010189, ENSSSCG00000015576, ENSSSCG00000000477, ENSSSCG00000009047, ENSSSCG00000011794
protein complex	cellular_component	16 out of 39 genes, 41.0%	245 out of 3284 genes, 7.5%	3.81E-07	ENSSSCG00000010189, ENSSSCG00000015335, ENSSSCG00000005065, ENSSSCG00000015576, ENSSSCG00000009217, ENSSSCG00000011794, ENSSSCG00000000217, ENSSSCG00000016853, ENSSSCG00000016027, ENSSSCG00000010625, ENSSSCG00000004927, ENSSSCG00000004524, ENSSSCG00000024927, ENSSSCG00000004792, ENSSSCG00000029382, ENSSSCG00000005243
nuclear part	cellular_component	12 out of 39 genes, 30.8%	129 out of 3284 genes, 3.9%	1.07E-06	ENSSSCG00000004524, ENSSSCG00000006084, ENSSSCG00000001563, ENSSSCG00000010189, ENSSSCG00000001958, ENSSSCG00000010625, ENSSSCG00000015576, ENSSSCG00000013602, ENSSSCG00000000477, ENSSSCG00000009047, ENSSSCG00000011794, ENSSSCG00000011201
nucleus	cellular_component	12 out of 39 genes, 30.8%	133 out of 3284 genes, 4.0%	1.53E-06	ENSSSCG00000009047, ENSSSCG00000013602, ENSSSCG00000000477, ENSSSCG00000011201, ENSSSCG00000011794, ENSSSCG00000006084, ENSSSCG00000001563, ENSSSCG00000004524, ENSSSCG00000010625, ENSSSCG00000015576, ENSSSCG00000001958, ENSSSCG00000010189
cytoplasmic part	cellular_component	15 out of 39 genes, 38.5%	249 out of 3284 genes, 7.6%	4.21E-06	ENSSSCG00000005065, ENSSSCG00000004927, ENSSSCG00000000912, ENSSSCG00000016077, ENSSSCG00000029382, ENSSSCG00000007088, ENSSSCG00000004620, ENSSSCG00000009217, ENSSSCG00000005243, ENSSSCG00000002799, ENSSSCG00000024927, ENSSSCG00000008573, ENSSSCG00000001924, ENSSSCG00000011845, ENSSSCG00000017277
cytoplasm	cellular_component	15 out of 39 genes, 38.5%	251 out of 3284 genes, 7.6%	4.69E-06	ENSSSCG00000017277, ENSSSCG00000011845, ENSSSCG00000001924, ENSSSCG00000005243, ENSSSCG00000009217, ENSSSCG00000029382, ENSSSCG00000007088, ENSSSCG00000004620, ENSSSCG00000008573, ENSSSCG00000024927, ENSSSCG00000002799, ENSSSCG00000004927, ENSSSCG00000005065, ENSSSCG00000016077, ENSSSCG00000000912
microtubule cytoskeleton	cellular_component	6 out of 39 genes, 15.4%	32 out of 3284 genes, 1.0%	0.00011	ENSSSCG00000001924, ENSSSCG00000006864, ENSSSCG00000015335, ENSSSCG00000014283, ENSSSCG00000000217, ENSSSCG00000004620
nuclear pore	cellular_component	3 out of 39 genes, 7.7%	3 out of 3284 genes, 0.1%	0.00013	ENSSSCG00000010189, ENSSSCG00000011794, ENSSSCG00000015576
intracellular organelle lumen	cellular_component	9 out of 39 genes, 23.1%	100 out of 3284 genes, 3.0%	0.00013	ENSSSCG00000006084, ENSSSCG00000001563, ENSSSCG00000004927, ENSSSCG00000010625, ENSSSCG00000009047, ENSSSCG00000013602, ENSSSCG00000000477, ENSSSCG00000011201, ENSSSCG00000011794

membrane-enclosed lumen	cellular_component	9 out of 39 genes, 23.1%	101 out of 3284 genes, 3.1%	0.00014	ENSSSCG00000011201, ENSSSCG00000011794, ENSSSCG00000009047, ENSSSCG00000013602, ENSSSCG00000000477, ENSSSCG00000010625, ENSSSCG00000004927, ENSSSCG00000001563, ENSSSCG00000006084
organelle lumen	cellular_component	9 out of 39 genes, 23.1%	101 out of 3284 genes, 3.1%	0.00014	ENSSSCG00000006084, ENSSSCG00000001563, ENSSSCG00000010625, ENSSSCG00000004927, ENSSSCG00000009047, ENSSSCG00000000477, ENSSSCG00000013602, ENSSSCG00000011201, ENSSSCG00000011794
nuclear lumen	cellular_component	8 out of 39 genes, 20.5%	80 out of 3284 genes, 2.4%	0.00024	ENSSSCG00000013602, ENSSSCG00000000477, ENSSSCG00000009047, ENSSSCG00000011794, ENSSSCG00000011201, ENSSSCG00000006084, ENSSSCG00000001563, ENSSSCG00000010625
cytoskeletal part	cellular_component	7 out of 39 genes, 17.9%	59 out of 3284 genes, 1.8%	0.00034	ENSSSCG00000000217, ENSSSCG00000017301, ENSSSCG00000004620, ENSSSCG00000001924, ENSSSCG00000015335, ENSSSCG00000014283, ENSSSCG00000006864
cytoskeleton	cellular_component	7 out of 39 genes, 17.9%	63 out of 3284 genes, 1.9%	0.00054	ENSSSCG00000006864, ENSSSCG00000014283, ENSSSCG00000015335, ENSSSCG00000001924, ENSSSCG00000004620, ENSSSCG00000017301, ENSSSCG00000000217
chromosomal part	cellular_component	6 out of 39 genes, 15.4%	49 out of 3284 genes, 1.5%	0.00152	ENSSSCG00000011213, ENSSSCG00000000477, ENSSSCG00000010189, ENSSSCG00000015335, ENSSSCG00000010625, ENSSSCG00000011201
chromosome	cellular_component	6 out of 39 genes, 15.4%	54 out of 3284 genes, 1.6%	0.00269	ENSSSCG00000000477, ENSSSCG00000011213, ENSSSCG00000015335, ENSSSCG00000011201, ENSSSCG00000010625, ENSSSCG00000010189
catalytic complex	cellular_component	8 out of 39 genes, 20.5%	113 out of 3284 genes, 3.4%	0.00325	ENSSSCG00000000477, ENSSSCG00000013602, ENSSSCG00000009047, ENSSSCG00000000912, ENSSSCG00000001958, ENSSSCG00000015335, ENSSSCG00000001563, ENSSSCG00000012116
supramolecular complex	cellular_component	5 out of 39 genes, 12.8%	35 out of 3284 genes, 1.1%	0.00381	ENSSSCG00000014283, ENSSSCG00000017277, ENSSSCG00000005243, ENSSSCG00000017301, ENSSSCG00000004620
supramolecular polymer	cellular_component	5 out of 39 genes, 12.8%	35 out of 3284 genes, 1.1%	0.00381	ENSSSCG00000014283, ENSSSCG00000017277, ENSSSCG00000004620, ENSSSCG00000017301, ENSSSCG00000005243
supramolecular fiber	cellular_component	5 out of 39 genes, 12.8%	35 out of 3284 genes, 1.1%	0.00381	ENSSSCG00000004620, ENSSSCG00000017301, ENSSSCG00000005243, ENSSSCG00000017277, ENSSSCG00000014283
nuclear envelope	cellular_component	3 out of 39 genes, 7.7%	9 out of 3284 genes, 0.3%	0.0104	ENSSSCG00000011794, ENSSSCG00000010189, ENSSSCG00000015576
endomembrane system	cellular_component	7 out of 39 genes, 17.9%	104 out of 3284 genes, 3.2%	0.01462	ENSSSCG00000008573, ENSSSCG00000009217, ENSSSCG00000007088, ENSSSCG00000004620, ENSSSCG00000010189, ENSSSCG00000011794, ENSSSCG00000015576

ISWI-type complex	cellular_component	2 out of 39 genes, 5.1%	3 out of 3284 genes, 0.1%	0.03437	ENSSSCG00000009047, ENSSSCG00000001958
positive regulation of transport	biological_process	11 out of 100 genes, 11.0%	72 out of 3284 genes, 2.2%	0.00477	ENSSSCG00000006035, ENSSSCG00000016223, ENSSSCG00000016027, ENSSSCG00000004524, ENSSSCG00000011681, ENSSSCG00000025427, ENSSSCG00000009217, ENSSSCG00000002921, ENSSSCG00000001062, ENSSSCG00000007030, ENSSSCG00000025478
regulation of localization	biological_process	21 out of 100 genes, 21.0%	252 out of 3284 genes, 7.7%	0.00848	ENSSSCG00000025427, ENSSSCG00000009217, ENSSSCG00000002921, ENSSSCG00000007030, ENSSSCG00000001062, ENSSSCG00000015482, ENSSSCG00000006035, ENSSSCG00000007056, ENSSSCG00000016223, ENSSSCG00000004595, ENSSSCG00000009834, ENSSSCG00000015576, ENSSSCG00000011681, ENSSSCG00000004524, ENSSSCG00000025478, ENSSSCG00000001924, ENSSSCG00000010081, ENSSSCG00000017670, ENSSSCG00000004620, ENSSSCG00000006911, ENSSSCG00000016027
cellular process	biological_process	67 out of 100 genes, 67.0%	1527 out of 3284 genes, 46.5%	0.01337	ENSSSCG00000001924, ENSSSCG00000011794, ENSSSCG00000016916, ENSSSCG00000016027, ENSSSCG00000006983, ENSSSCG00000008614, ENSSSCG00000009047, ENSSSCG00000025551, ENSSSCG00000005243, ENSSSCG00000017301, ENSSSCG00000016194, ENSSSCG00000025427, ENSSSCG00000016223, ENSSSCG00000011183, ENSSSCG00000014283, ENSSSCG00000008186, ENSSSCG00000025478, ENSSSCG00000011103, ENSSSCG00000013602, ENSSSCG00000004524, ENSSSCG00000014893, ENSSSCG00000015874, ENSSSCG00000015576, ENSSSCG00000011681, ENSSSCG00000027093, ENSSSCG00000009473, ENSSSCG00000026475, ENSSSCG00000007460, ENSSSCG00000011001, ENSSSCG00000005066, ENSSSCG00000000477, ENSSSCG00000004595, ENSSSCG00000009994, ENSSSCG00000007056, ENSSSCG00000010081, ENSSSCG00000029933, ENSSSCG00000010625, ENSSSCG00000014114, ENSSSCG00000016156, ENSSSCG00000008408, ENSSSCG00000004620, ENSSSCG00000014292, ENSSSCG00000017136, ENSSSCG00000015814, ENSSSCG00000015455, ENSSSCG00000000625, ENSSSCG00000006201, ENSSSCG00000011201, ENSSSCG00000006035, ENSSSCG00000010679, ENSSSCG00000013856, ENSSSCG00000006864, ENSSSCG00000005998, ENSSSCG00000015568, ENSSSCG00000012163, ENSSSCG00000005096, ENSSSCG00000028771, ENSSSCG00000016077, ENSSSCG00000007153, ENSSSCG00000012116, ENSSSCG00000010189, ENSSSCG00000009217, ENSSSCG00000005215, ENSSSCG00000001958, ENSSSCG00000023873, ENSSSCG00000000217, ENSSSCG00000007474
regulation of cellular localization	biological_process	10 out of 100 genes, 10.0%	66 out of 3284 genes, 2.0%	0.01372	ENSSSCG00000004524, ENSSSCG00000009217, ENSSSCG00000025427, ENSSSCG00000011681, ENSSSCG00000015576, ENSSSCG00000015482, ENSSSCG00000001062, ENSSSCG00000010081, ENSSSCG00000016223, ENSSSCG00000004620
macromolecule metabolic process	biological_process	27 out of 100 genes, 27.0%	392 out of 3284 genes, 11.9%	0.01436	ENSSSCG00000006983, ENSSSCG00000014114, ENSSSCG00000007460, ENSSSCG00000026475, ENSSSCG00000005096, ENSSSCG00000028771, ENSSSCG00000016077, ENSSSCG00000008614, ENSSSCG00000006864, ENSSSCG00000010679, ENSSSCG00000015576, ENSSSCG00000011794, ENSSSCG00000014893, ENSSSCG00000013602, ENSSSCG00000011201, ENSSSCG00000004595, ENSSSCG00000001958, ENSSSCG00000015455, ENSSSCG00000007474, ENSSSCG00000006035, ENSSSCG00000011001, ENSSSCG00000005215, ENSSSCG00000016194, ENSSSCG00000017301, ENSSSCG00000000477, ENSSSCG00000010189, ENSSSCG00000005243
regulation of transport	biological_process	17 out of 100 genes, 17.0%	188 out of 3284 genes, 5.7%	0.02256	ENSSSCG00000016223, ENSSSCG00000006035, ENSSSCG00000009834, ENSSSCG00000009217, ENSSSCG00000025427, ENSSSCG00000015482, ENSSSCG00000007030, ENSSSCG00000001062, ENSSSCG00000002921, ENSSSCG00000004620, ENSSSCG00000016027, ENSSSCG00000015576, ENSSSCG00000011681, ENSSSCG00000004524, ENSSSCG00000025478, ENSSSCG00000010081, ENSSSCG00000017670
cellular macromolecule metabolic process	biological_process	24 out of 100 genes, 24.0%	344 out of 3284 genes, 10.5%	0.0378	ENSSSCG00000007460, ENSSSCG00000026475, ENSSSCG00000016077, ENSSSCG00000005096, ENSSSCG00000028771, ENSSSCG00000008614, ENSSSCG00000006983, ENSSSCG00000014114, ENSSSCG00000015576, ENSSSCG00000011794, ENSSSCG00000014893, ENSSSCG00000013602, ENSSSCG00000006864, ENSSSCG00000010679, ENSSSCG00000007474, ENSSSCG00000011201, ENSSSCG00000001958, ENSSSCG00000015455, ENSSSCG00000016194, ENSSSCG00000005215, ENSSSCG00000017301, ENSSSCG00000000477, ENSSSCG00000005243, ENSSSCG00000011001

regulation of cell cycle process	biological_process	8 out of 100 genes, 8.0%	48 out of 3284 genes, 1.5%	0.04736	ENSSSCG00000010625, ENSSSCG00000004753, ENSSSCG00000007056, ENSSSCG00000029764, ENSSSCG00000001924, ENSSSCG00000000488, ENSSSCG00000015576, ENSSSCG00000009217

Table S3-3. KEGG pathway analysis of the host genes for differentially expressed circRNAs in LH vs. LL.

Pathway ID	Pathway	Kategory	DEGs with pathway annotation (160)	All genes with pathway annotation (3975)	*P* value	Qvalue	Genelist	KOlist
ko05215	Prostate cancer	Human Diseases	7(4.38%)	53(1.33%)	1.09E-03	0.03605244	ENSSSCG00000010548,ENSSSCG00000005015,ENSSSCG00000000068,ENSSSCG00000007030,ENSSSCG00000010872,ENSSSCG00000000488,ENSSSCG00000009401,	K04467,K03099,K04498,K07209,K04456,K06643,K06618,
ko05131	Shigellosis	Human Diseases	5(3.12%)	34(0.86%)	2.04E-03	0.041110942	ENSSSCG00000010548,ENSSSCG00000003520,ENSSSCG00000007030,ENSSSCG00000016185,ENSSSCG00000013297,	K04467,K04393,K07209,K05758,K06256,
ko00100	Steroid biosynthesis	Metabolism	3(1.88%)	17(0.43%)	3.99E-03	0.048510153	ENSSSCG00000026044,ENSSSCG00000005970,ENSSSCG00000010450,	K00801,K00511,K01052,
ko01522	Endocrine resistance		8(5.00%)	53(1.33%)	2.14E-04	0.009881074	ENSSSCG00000005015,ENSSSCG00000024716,ENSSSCG00000007458,ENSSSCG00000010872,ENSSSCG00000017670,ENSSSCG00000000488,ENSSSCG00000009401,ENSSSCG00000018039,	K03099,K02599,K11256,K04456,K04688,K06643,K06618,K04650,
ko04530	Tight junction	Cellular Processes	8(5.00%)	76(1.91%)	3.11E-03	0.044689841	ENSSSCG00000015013,ENSSSCG00000003520,ENSSSCG00000030470,ENSSSCG00000011833,ENSSSCG00000008444,ENSSSCG00000011103,ENSSSCG00000008881,ENSSSCG00000009657,	K05762,K04393,K05701,K12076,K18050,K04237,K08018,K04354,
ko04931	Insulin resistance		6(3.75%)	50(1.26%)	3.48E-03	0.044689841	ENSSSCG00000008339,ENSSSCG00000013241,ENSSSCG00000007030,ENSSSCG00000008444,ENSSSCG00000010872,ENSSSCG00000017670,	K00820,K08536,K07209,K18050,K04456,K04688,
ko00909	Sesquiterpenoid and triterpenoid biosynthesis	Metabolism	2(1.25%)	2(0.05%)	0.00E+00	0	ENSSSCG00000026044,ENSSSCG00000005970,	K00801,K00511,
ko04919	Thyroid hormone signaling pathway	Organismal Systems	11(6.88%)	65(1.64%)	7.56E-06	0.000873312	ENSSSCG00000014949,ENSSSCG00000024716,ENSSSCG00000007458,ENSSSCG00000000068,ENSSSCG00000010872,ENSSSCG00000012250,ENSSSCG00000000488,ENSSSCG00000018039,ENSSSCG00000017672,ENSSSCG00000016434,ENSSSCG00000011676,	K15133,K02599,K11256,K04498,K04456,K15156,K06643,K04650,K15164,K07208,K01540,
ko05220	Chronic myeloid leukemia	Human Diseases	6(3.75%)	46(1.16%)	2.14E-03	0.041110942	ENSSSCG00000010548,ENSSSCG00000005015,ENSSSCG00000007030,ENSSSCG00000010872,ENSSSCG00000000488,ENSSSCG00000009401,	K04467,K03099,K07209,K04456,K06643,K06618,
ko04110	Cell cycle	Cellular Processes	9(5.62%)	88(2.21%)	2.53E-03	0.041690122	ENSSSCG00000015455,ENSSSCG00000011679,ENSSSCG00000000068,ENSSSCG00000004466,ENSSSCG00000010625,ENSSSCG00000004505,ENSSSCG00000000488,ENSSSCG00000030514,ENSSSCG00000009401,	K03347,K06640,K04498,K08866,K06669,K04500,K06643,K16332,K06618,
ko05160	Hepatitis C	Human Diseases	8(5.00%)	62(1.56%)	7.17E-04	0.027621347	ENSSSCG00000010548,ENSSSCG00000005015,ENSSSCG00000013241,ENSSSCG00000009759,ENSSSCG00000007030,ENSSSCG00000010872,ENSSSCG00000006033,ENSSSCG00000009657,	K04467,K03099,K08536,K13885,K07209,K04456,K03250,K04354,
ko04068	FoxO signaling pathway	Environmental Information Processing	9(5.62%)	83(2.09%)	1.62E-03	0.041110942	ENSSSCG00000010548,ENSSSCG00000005015,ENSSSCG00000030396,ENSSSCG00000000068,ENSSSCG00000007030,ENSSSCG00000010872,ENSSSCG00000004505,ENSSSCG00000000488,ENSSSCG00000030514,	K04467,K03099,K11431,K04498,K07209,K04456,K04500,K06643,K16332,
ko05221	Acute myeloid leukemia	Human Diseases	7(4.38%)	33(0.83%)	3.40E-05	0.00261805	ENSSSCG00000006115,ENSSSCG00000010548,ENSSSCG00000005015,ENSSSCG00000007030,ENSSSCG00000010872,ENSSSCG00000017670,ENSSSCG00000008842,	K10053,K04467,K03099,K07209,K04456,K04688,K05091,

ko00510	N-Glycan biosynthesis	Metabolism	5(3.12%)	36(0.91%)	2.76E-03	0.042569694	ENSSSCG00000003815,ENSSSCG00000014887,ENSSSCG00000011612,ENSSSCG00000011229,ENSSSCG00000014195,	K03848,K03849,K12666,K07151,K01231,
ko05206	MicroRNAs in cancer	Human Diseases	11(6.88%)	86(2.16%)	1.38E-04	0.007941918	ENSSSCG00000006038,ENSSSCG00000005015,ENSSSCG00000015013,ENSSSCG00000024716,ENSSSCG00000010627,ENSSSCG00000000068,ENSSSCG00000007030,ENSSSCG00000008444,ENSSSCG00000013297,ENSSSCG00000000488,ENSSSCG00000028840,	K17442,K03099,K05762,K02599,K16865,K04498,K07209,K18050,K06256,K06643,K11430,
ko05224	Breast cancer		8(5.00%)	71(1.79%)	1.93E-03	0.041110942	ENSSSCG00000005015,ENSSSCG00000000625,ENSSSCG00000024716,ENSSSCG00000007458,ENSSSCG00000010872,ENSSSCG00000017670,ENSSSCG00000009401,ENSSSCG00000008842,	K03099,K03068,K02599,K11256,K04456,K04688,K06618,K05091,
ko05212	Pancreatic cancer	Human Diseases	6(3.75%)	43(1.08%)	1.42E-03	0.041087161	ENSSSCG00000010548,ENSSSCG00000003520,ENSSSCG00000007030,ENSSSCG00000010872,ENSSSCG00000004505,ENSSSCG00000009401,	K04467,K04393,K07209,K04456,K04500,K06618,
ko04111	Cell cycle - yeast	Cellular Processes	6(3.75%)	47(1.18%)	2.43E-03	0.041690122	ENSSSCG00000015455,ENSSSCG00000016842,ENSSSCG00000011731,ENSSSCG00000004466,ENSSSCG00000010625,ENSSSCG00000009657,	K03347,K06672,K06675,K08866,K06669,K04354,
ko04141	Protein processing in endoplasmic reticulum	Genetic Information Processing	10(6.25%)	107(2.69%)	3.47E-03	0.044689841	ENSSSCG00000016017,ENSSSCG00000015455,ENSSSCG00000003398,ENSSSCG00000014304,ENSSSCG00000011214,ENSSSCG00000007097,ENSSSCG00000014020,ENSSSCG00000011612,ENSSSCG00000011229,ENSSSCG00000011000,	K09530,K03347,K10597,K14007,K01456,K14006,K08054,K12666,K07151,K09502,

Table S3-4. KEGG pathway analysis of the host genes for differentially expressed circRNAs in FH vs. FL.

Pathway ID	Pathway	Kategory	DEGs with pathway annotation (179)	All genes with pathway annotation (3975)	*P* value	Qvalue	Genelist	KOlist
ko04391	Hippo signaling pathway - fly	Environmental Information Processing	6(3.35%)	34(0.86%)	6.39E-04	0.017272317	ENSSSCG00000015842,ENSSSCG00000026502,ENSSSCG00000011103,ENSSSCG00000002287,ENSSSCG00000011745,ENSSSCG00000011833,	K04382,K16673,K04237,K06091,K06069,K12076,
ko04144	Endocytosis	Cellular Processes	14(7.82%)	145(3.65%)	1.95E-03	0.045460061	ENSSSCG00000006137,ENSSSCG00000015115,ENSSSCG00000011237,ENSSSCG00000000488,ENSSSCG00000000912,ENSSSCG00000006201,ENSSSCG00000008768,ENSSSCG00000000037,ENSSSCG00000004140,ENSSSCG00000011103,ENSSSCG00000026239,ENSSSCG00000008573,ENSSSCG00000011745,ENSSSCG00000014023,	K05633,K04707,K12200,K06643,K12478,K18442,K18440,K12493,K12199,K04237,K18468,K07903,K06069,K12482,
ko04111	Cell cycle - yeast	Cellular Processes	8(4.47%)	47(1.18%)	1.93E-04	0.008087253	ENSSSCG00000017308,ENSSSCG00000010625,ENSSSCG00000016842,ENSSSCG00000015842,ENSSSCG00000011731,ENSSSCG00000015668,ENSSSCG00000006864,ENSSSCG00000015455,	K03350,K06669,K06672,K04382,K06675,K02606,K06639,K03347,
ko04212	Longevity regulating pathway - worm		6(3.35%)	33(0.83%)	5.27E-04	0.017272317	ENSSSCG00000015313,ENSSSCG00000016077,ENSSSCG00000021170,ENSSSCG00000017670,ENSSSCG00000005096,ENSSSCG00000000846,	K17705,K04077,K13356,K04688,K08268,K14966,
ko03018	RNA degradation	Genetic Information Processing	9(5.03%)	50(1.26%)	5.70E-05	0.005985641	ENSSSCG00000016911,ENSSSCG00000002799,ENSSSCG00000000502,ENSSSCG00000016077,ENSSSCG00000015097,ENSSSCG00000030404,ENSSSCG00000024916,ENSSSCG00000009319,ENSSSCG00000016193,	K12598,K12604,K12605,K04077,K12614,K03514,K14442,K12572,K12606,
ko04110	Cell cycle	Cellular Processes	12(6.70%)	88(2.21%)	1.25E-04	0.006571748	ENSSSCG00000017308,ENSSSCG00000000488,ENSSSCG00000011679,ENSSSCG00000007331,ENSSSCG00000004524,ENSSSCG00000010625,ENSSSCG00000000068,ENSSSCG00000020870,ENSSSCG00000009401,ENSSSCG00000015668,ENSSSCG00000006864,ENSSSCG00000015455,	K03350,K06643,K06640,K04681,K04501,K06669,K04498,K06629,K06618,K02606,K06639,K03347,
ko04350	TGF-beta signaling pathway	Environmental Information Processing	8(4.47%)	55(1.38%)	6.58E-04	0.017272317	ENSSSCG00000021893,ENSSSCG00000015874,ENSSSCG00000007331,ENSSSCG00000004524,ENSSSCG00000015842,ENSSSCG00000000068,ENSSSCG00000017670,ENSSSCG00000015455,	K04514,K04675,K04681,K04501,K04382,K04498,K04688,K03347,
ko00073	Cutin, suberine and wax biosynthesis	Metabolism	1(0.56%)	1(0.03%)	0.00E+00	0	ENSSSCG00000021170,	K13356,
ko04712	Circadian rhythm - plant	Organismal Systems	2(1.12%)	3(0.08%)	8.99E-05	0.006290147	ENSSSCG00000015509,ENSSSCG00000007203,	K10143,K03097,

Table S4-1. Gene symbols in LH vs. LL.

Genelist	Gene name
ENSSSCG00000010548	CHUK
ENSSSCG00000026044	FDFT1
ENSSSCG00000005015	SOS2
ENSSSCG00000008339	GFPT1
ENSSSCG00000014949	MED17
ENSSSCG00000015455	CUL1
ENSSSCG00000006115	RUNX1T1
ENSSSCG00000016017	DNAJC10
ENSSSCG00000017672	MED13
ENSSSCG00000009401	RB1
ENSSSCG00000016434	RHEB
ENSSSCG00000030514	-
ENSSSCG00000011000	DNAJA1
ENSSSCG00000018039	NCOR1
ENSSSCG00000000488	MDM2
ENSSSCG00000011676	-
ENSSSCG00000011612	RPN1
ENSSSCG00000011229	STT3B
ENSSSCG00000004505	SMAD2
ENSSSCG00000008842	KIT
ENSSSCG00000014020	CANX
ENSSSCG00000005970	SQLE
ENSSSCG00000024716	-
ENSSSCG00000013241	NR1H3
ENSSSCG00000011679	ATR
ENSSSCG00000017670	RPS6KB1
ENSSSCG00000010872	AKT3
ENSSSCG00000010625	SMC3
ENSSSCG00000007030	IKBKB
ENSSSCG00000011214	NGLY1
ENSSSCG00000012250	MED14
ENSSSCG00000007097	SEC23B
ENSSSCG00000008444	PRKCE
ENSSSCG00000000068	EP300
ENSSSCG00000004466	TTK
ENSSSCG00000014304	SEC24A
ENSSSCG00000010450	LIPA
ENSSSCG00000007458	NCOA3
ENSSSCG00000030396	SETD7
ENSSSCG00000003398	-

Table 4. Significantly enriched pathways related to reproduction in LH vs. LL.

Pathway ID	Pathway	Kategory	DEGs with pathway annotation (160)	All genes with pathway annotation (3975)	*P* value	Qvalue	Genelist	KOlist
ko05215	Prostate cancer	Human Diseases	7(4.38%)	53(1.33%)	1.09E-03	0.03605244	ENSSSCG00000010548,ENSSSCG00000005015,ENSSSCG00000000068,ENSSSCG00000007030,ENSSSCG00000010872,ENSSSCG00000000488,ENSSSCG00000009401,	K04467,K03099,K04498,K07209,K04456,K06643,K06618,
ko00100	Steroid biosynthesis	Metabolism	3(1.88%)	17(0.43%)	3.99E-03	0.048510153	ENSSSCG00000026044,ENSSSCG00000005970,ENSSSCG00000010450,	K00801,K00511,K01052,
ko01522	Endocrine resistance		8(5.00%)	53(1.33%)	2.14E-04	0.009881074	ENSSSCG00000005015,ENSSSCG00000024716,ENSSSCG00000007458,ENSSSCG00000010872,ENSSSCG00000017670,ENSSSCG00000000488,ENSSSCG00000009401,ENSSSCG00000018039,	K03099,K02599,K11256,K04456,K04688,K06643,K06618,K04650,
ko04931	Insulin resistance		6(3.75%)	50(1.26%)	3.48E-03	0.044689841	ENSSSCG00000008339,ENSSSCG00000013241,ENSSSCG00000007030,ENSSSCG00000008444,ENSSSCG00000010872,ENSSSCG00000017670,	K00820,K08536,K07209,K18050,K04456,K04688,
ko04919	Thyroid hormone signaling pathway	Organismal Systems	11(6.88%)	65(1.64%)	7.56E-06	0.000873312	ENSSSCG00000014949,ENSSSCG00000024716,ENSSSCG00000007458,ENSSSCG00000000068,ENSSSCG00000010872,ENSSSCG00000012250,ENSSSCG00000000488,ENSSSCG00000018039,ENSSSCG00000017672,ENSSSCG00000016434,ENSSSCG00000011676,	K15133,K02599,K11256,K04498,K04456,K15156,K06643,K04650,K15164,K07208,K01540,
ko04110	Cell cycle	Cellular Processes	9(5.62%)	88(2.21%)	2.53E-03	0.041690122	ENSSSCG00000015455,ENSSSCG00000011679,ENSSSCG00000000068,ENSSSCG00000004466,ENSSSCG00000010625,ENSSSCG00000004505,ENSSSCG00000000488,ENSSSCG00000030514,ENSSSCG00000009401,	K03347,K06640,K04498,K08866,K06669,K04500,K06643,K16332,K06618,
ko04068	FoxO signaling pathway	Environmental Information Processing	9(5.62%)	83(2.09%)	1.62E-03	0.041110942	ENSSSCG00000010548,ENSSSCG00000005015,ENSSSCG00000030396,ENSSSCG00000000068,ENSSSCG00000007030,ENSSSCG00000010872,ENSSSCG00000004505,ENSSSCG00000000488,ENSSSCG00000030514,	K04467,K03099,K11431,K04498,K07209,K04456,K04500,K06643,K16332,
ko05221	Acute myeloid leukemia	Human Diseases	7(4.38%)	33(0.83%)	3.40E-05	0.00261805	ENSSSCG00000006115,ENSSSCG00000010548,ENSSSCG00000005015,ENSSSCG00000007030,ENSSSCG00000010872,ENSSSCG00000017670,ENSSSCG00000008842,	K10053,K04467,K03099,K07209,K04456,K04688,K05091,
ko04141	Protein processing in endoplasmic reticulum	Genetic Information Processing	10(6.25%)	107(2.69%)	3.47E-03	0.044689841	ENSSSCG00000016017,ENSSSCG00000015455,ENSSSCG00000003398,ENSSSCG00000014304,ENSSSCG00000011214,ENSSSCG00000007097,ENSSSCG00000014020,ENSSSCG00000011612,ENSSSCG00000011229,ENSSSCG00000011000,	K09530,K03347,K10597,K14007,K01456,K14006,K08054,K12666,K07151,K09502,

Table S4-3. Gene symbols in FH vs. FL.

Genelist	Gene name
ENSSSCG00000011679	ATR
ENSSSCG00000007331	RBL1
ENSSSCG00000004524	SMAD4
ENSSSCG00000000488	MDM2
ENSSSCG00000015874	ACVR1
ENSSSCG00000017308	CDC27
ENSSSCG00000021893	ROCK1
ENSSSCG00000020870	DBF4
ENSSSCG00000015455	CUL1
ENSSSCG00000006864	CDC14A
ENSSSCG00000015668	ORC4
ENSSSCG00000009401	RB1
ENSSSCG00000000068	EP300
ENSSSCG00000017670	RPS6KB1
ENSSSCG00000010625	SMC3
ENSSSCG00000015842	PPP2CB
ENSSSCG00000011745	PRKCI
ENSSSCG00000011103	PARD3
ENSSSCG00000026502	LIX1L
ENSSSCG00000011833	DLG1
ENSSSCG00000002287	MPP5

Table S4-4. Significantly enriched pathways related to reproduction in FH vs. FL.

Pathway ID	Pathway	Kategory	DEGs with pathway annotation (179)	All genes with pathway annotation (3975)	Pvalue	Qvalue	Genelist	KOlist
ko04391	Hippo signaling pathway - fly	Environmental Information Processing	6(3.35%)	34(0.86%)	6.39E-04	0.017272317	ENSSSCG00000015842,ENSSSCG00000026502,ENSSSCG00000011103,ENSSSCG00000002287,ENSSSCG00000011745,ENSSSCG00000011833,	K04382,K16673,K04237,K06091,K06069,K12076,
ko04110	Cell cycle	Cellular Processes	12(6.70%)	88(2.21%)	1.25E-04	0.006571748	ENSSSCG00000017308,ENSSSCG00000000488,ENSSSCG00000011679,ENSSSCG00000007331,ENSSSCG00000004524,ENSSSCG00000010625,ENSSSCG00000000068,ENSSSCG00000020870,ENSSSCG00000009401,ENSSSCG00000015668,ENSSSCG00000006864,ENSSSCG00000015455,	K03350,K06643,K06640,K04681,K04501,K06669,K04498,K06629,K06618,K02606,K06639,K03347,
ko04350	TGF-beta signaling pathway	Environmental Information Processing	8(4.47%)	55(1.38%)	6.58E-04	0.017272317	ENSSSCG00000021893,ENSSSCG00000015874,ENSSSCG00000007331,ENSSSCG00000004524,ENSSSCG00000015842,ENSSSCG00000000068,ENSSSCG00000017670,ENSSSCG00000015455,	K04514,K04675,K04681,K04501,K04382,K04498,K04688,K03347,

Table S4-5. Protein-protein interaction information in LH vs. LL.

node1	node2	node1 accession	node2 accession	score
AKT3	CHUK	ENSSSCP00000011590	ENSSSCP00000029824	0.948
AKT3	MDM2	ENSSSCP00000011590	ENSSSCP00000000518	0.977
AKT3	RPS6KB1	ENSSSCP00000011590	ENSSSCP00000018724	0.946
CANX	SEC24A	ENSSSCP00000014907	ENSSSCP00000015215	0.944
CHUK	AKT3	ENSSSCP00000029824	ENSSSCP00000011590	0.948
CHUK	IKBKB	ENSSSCP00000029824	ENSSSCP00000007492	0.978
EP300	NCOA3	ENSSSCP00000020812	ENSSSCP00000007946	0.987
FDFT1	SQLE	ENSSSCP00000022970	ENSSSCP00000006378	0.994
IKBKB	CHUK	ENSSSCP00000007492	ENSSSCP00000029824	0.978
MDM2	AKT3	ENSSSCP00000000518	ENSSSCP00000011590	0.977
MED13	MED14	ENSSSCP00000023123	ENSSSCP00000013036	0.98
MED13	MED17	ENSSSCP00000023123	ENSSSCP00000015871	0.977
MED14	MED13	ENSSSCP00000013036	ENSSSCP00000023123	0.98
MED14	MED17	ENSSSCP00000013036	ENSSSCP00000015871	0.996
MED17	MED13	ENSSSCP00000015871	ENSSSCP00000023123	0.977
MED17	MED14	ENSSSCP00000015871	ENSSSCP00000013036	0.996
NCOA3	EP300	ENSSSCP00000007946	ENSSSCP00000020812	0.987
NCOR1	SMAD2	ENSSSCP00000019114	ENSSSCP00000004860	0.912
RHEB	RPS6KB1	ENSSSCP00000017414	ENSSSCP00000018724	0.978
RPN1	STT3B	ENSSSCP00000012376	ENSSSCP00000011978	0.997
RPS6KB1	AKT3	ENSSSCP00000018724	ENSSSCP00000011590	0.946
RPS6KB1	RHEB	ENSSSCP00000018724	ENSSSCP00000017414	0.978
SEC24A	CANX	ENSSSCP00000015215	ENSSSCP00000014907	0.944
SMAD2	NCOR1	ENSSSCP00000004860	ENSSSCP00000019114	0.912
SQLE	FDFT1	ENSSSCP00000006378	ENSSSCP00000022970	0.994
STT3B	RPN1	ENSSSCP00000011978	ENSSSCP00000012376	0.997

Table S4-6. Protein-protein interaction information in FH vs. FL.

node1	node2	node1 accession	node2 accession	score
ATR	DBF4	ENSSSCP00000012449	ENSSSCP00000022275	0.962
ATR	ORC4	ENSSSCP00000012449	ENSSSCP00000016611	0.908
CDC27	CUL1	ENSSSCP00000018341	ENSSSCP00000016387	0.958
CUL1	CDC27	ENSSSCP00000016387	ENSSSCP00000018341	0.958
CUL1	PPP2CB	ENSSSCP00000016387	ENSSSCP00000016788	0.927
DBF4	ATR	ENSSSCP00000022275	ENSSSCP00000012449	0.962
DBF4	ORC4	ENSSSCP00000022275	ENSSSCP00000016611	0.984
DLG1	MDM2	ENSSSCP00000030935	ENSSSCP00000000518	0.922
MDM2	DLG1	ENSSSCP00000000518	ENSSSCP00000030935	0.922
MDM2	PPP2CB	ENSSSCP00000000518	ENSSSCP00000016788	0.904
ORC4	ATR	ENSSSCP00000016611	ENSSSCP00000012449	0.908
ORC4	DBF4	ENSSSCP00000016611	ENSSSCP00000022275	0.984
PARD3	PRKCI	ENSSSCP00000020289	ENSSSCP00000012516	0.98
PPP2CB	CUL1	ENSSSCP00000016788	ENSSSCP00000016387	0.927
PPP2CB	MDM2	ENSSSCP00000016788	ENSSSCP00000000518	0.904
PPP2CB	RB1	ENSSSCP00000016788	ENSSSCP00000010032	0.936
PPP2CB	RBL1	ENSSSCP00000016788	ENSSSCP00000007808	0.937
PPP2CB	SMC3	ENSSSCP00000016788	ENSSSCP00000011319	0.918
PRKCI	PARD3	ENSSSCP00000012516	ENSSSCP00000020289	0.98
RB1	PPP2CB	ENSSSCP00000010032	ENSSSCP00000016788	0.936
RBL1	PPP2CB	ENSSSCP00000007808	ENSSSCP00000016788	0.937
RBL1	SMAD4	ENSSSCP00000007808	ENSSSCP00000004879	0.945
SMAD4	RBL1	ENSSSCP00000004879	ENSSSCP00000007808	0.945
SMC3	PPP2CB	ENSSSCP00000011319	ENSSSCP00000016788	0.918

Table S5-1. Differential expression analysis of miRNAs in the LH vs. LL comparison.

gene_id	length	Value (LH)	Value (LL)	log2 (FoldChange)	*P* value
all_ssc-miR-1	22	1845.93	502.7525	-2.263443889	3.51E-10
NovelmiRNA-1408	25	0	11.91	9.348411859	5.54E-07
NovelmiRNA-1168	19	3.075	0	-8.446829999	1.31E-05
NovelmiRNA-467	23	2.955	0	-8.217452336	2.66E-05
NovelmiRNA-329	23	0	2.9925	7.986035747	2.84E-05
NovelmiRNA-904	23	0	2.9925	7.986035747	2.84E-05
NovelmiRNA-842	22	0	3.75	7.882218366	6.61E-05
NovelmiRNA-1577	22	0	3.5625	7.779191454	9.24E-05
NovelmiRNA-1526	22	66.765	13.3375	-2.748515469	0.000106869
all_ssc-miR-338	21	1.2275	0	-7.565840544	0.000115404
all_ssc-miR-185	22	407.9825	1485.7875	1.476028234	0.000125398
all_ssc-miR-100	21	5880.2375	3352.35	-1.256473886	0.000268406
all_ssc-miR-107	22	454.7225	1396.37	1.234071291	0.000346611
all_ssc-miR-148a-5p	22	38489.1575	18165.1075	-1.462590517	0.000450987
NovelmiRNA-1380	24	0	1.955	7.07972895	0.000519545
NovelmiRNA-780	18	0	1.215	6.782288175	0.000798403
NovelmiRNA-1071	21	0	1.155	6.733539051	0.00088831
NovelmiRNA-1466	21	0	1.155	6.733539051	0.00088831
NovelmiRNA-1717	22	0	1.1125	6.646534875	0.001113666
NovelmiRNA-1564	24	0.8975	0	-6.69994487	0.001343927
NovelmiRNA-453	22	0	1.3375	6.587871599	0.001573712
all_ssc-miR-195	22	810.1825	447.9225	-1.331255836	0.001935099
all_ssc-miR-26a	22	49872.7625	30856.62	-1.164928776	0.002099805
all_ssc-miR-10a-5p	22	2259.6825	309.9725	-3.054047673	0.002318199
NovelmiRNA-693	22	0.6075	0	-6.403937959	0.002440385
all_ssc-miR-450a	20	419.805	1320.475	1.281880428	0.002480361
NovelmiRNA-335	21	0	1.1175	6.359235834	0.002532185
NovelmiRNA-1308	21	0	1.115	6.349028896	0.002601714
all_ssc-miR-99a	21	97174.0825	36995.39	-1.846793754	0.003105245
all_ssc-miR-361-3p	22	80.805	0	-6.64026204	0.003233559
NovelmiRNA-688	24	64.7525	0	-6.633113654	0.003270202
NovelmiRNA-653	23	43.3325	0	-6.594790981	0.003473113
NovelmiRNA-14	18	0	0.925	6.129881431	0.003952109
NovelmiRNA-1651	22	0.7725	0	-6.174163631	0.004455952
all_ssc-miR-450c-5p	20	613.5075	1844.58	1.218958472	0.004979712
NovelmiRNA-1701	24	8.245	0	-6.341101165	0.005127373
NovelmiRNA-1068	21	9.0125	0	-6.318225172	0.005306626
NovelmiRNA-574	18	0	16.1725	6.317264906	0.005312358
NovelmiRNA-1631	23	0	12.0475	6.248881488	0.005882098
NovelmiRNA-1694	21	0.555	0	-5.924383752	0.006329401
all_ssc-miR-196a	23	31.5475	0.6525	-4.871375777	0.006368564
NovelmiRNA-1359	21	0	0.755	5.857467872	0.006608648
NovelmiRNA-1507	21	0	0.755	5.857467872	0.006608648
NovelmiRNA-35	19	5.1325	0	-6.140139252	0.006904478
NovelmiRNA-482	18	0	1.07	5.909210129	0.006917797
NovelmiRNA-1041	22	0.6175	0	-5.892626852	0.007340729
NovelmiRNA-96	23	0.5075	0	-5.871805286	0.007553383
NovelmiRNA-721	22	0	0.9175	5.829303486	0.00786721
NovelmiRNA-1718	23	0.5825	0	-5.825601157	0.008191914

all_ssc-miR-542-5p	22	664.655	2028.2125	1.174792156	0.00932336
NovelmiRNA-1671	18	8.545	48.5275	1.96414682	0.009955086
NovelmiRNA-439	24	0	0.61	5.608179241	0.010146138
NovelmiRNA-1659	18	0.565	0	-5.685197656	0.010320445
NovelmiRNA-1447	21	0	0.6025	5.59172159	0.010420186
NovelmiRNA-937	20	0.545	0	-5.676505878	0.010481256
NovelmiRNA-1285	18	8.2325	46.67	1.959241785	0.010759343
all_ssc-miR-204	22	105.2175	51.99	-1.415625697	0.010938725
all_ssc-miR-184	22	125.295	441.615	1.374133434	0.010951586
NovelmiRNA-1320	19	0.3475	0	-5.578305655	0.011069438
NovelmiRNA-1459	18	0	0.575	5.555999236	0.011110588
NovelmiRNA-252	21	0	0.7675	5.604258536	0.011392994
NovelmiRNA-1714	19	0	0.8025	5.585658271	0.011721365
NovelmiRNA-508	21	0.54	0	-5.576585022	0.01235663
all_ssc-miR-105-1	22	0.2325	0	-5.401539468	0.013434957
NovelmiRNA-741	18	0	0.71	5.440995317	0.014675437
NovelmiRNA-1450	18	0	0.71	5.440995317	0.014675437
NovelmiRNA-22	23	0.425	0	-5.438886209	0.015137645
NovelmiRNA-1139	23	0.425	0	-5.438886209	0.015137645
NovelmiRNA-973	23	0	0.5	5.345466252	0.015536616
all_ssc-miR-1307	23	180.1875	528.8925	1.150053443	0.015702566
NovelmiRNA-1639	18	0	0.49	5.335850173	0.015703408
NovelmiRNA-1162	22	0.41	0	-5.406532108	0.015881938
NovelmiRNA-464	25	0.405	0	-5.401690274	0.016010713
NovelmiRNA-1491	19	0	2.28	5.517703277	0.016305194
all_ssc-miR-362	24	70.0325	190.565	1.045740206	0.017062137
all_ssc-miR-1343	22	36.7025	117.785	1.262515085	0.017506731
NovelmiRNA-1475	22	0	0.4625	5.262078783	0.017519348
NovelmiRNA-1117	20	0	0.6225	5.321556025	0.017671172
NovelmiRNA-826	21	0	0.6475	5.303411091	0.018072418
NovelmiRNA-641	21	0	0.59	5.237889585	0.019920579
NovelmiRNA-1634	21	0	0.59	5.237889585	0.019920579
NovelmiRNA-299	22	0	0.6175	5.212901534	0.020730055
all_ssc-miR-504	22	504.6575	296.4825	-1.196402728	0.021282529
all_ssc-miR-133a-5p	23	128.6425	67.0425	-1.325780851	0.022547224
NovelmiRNA-629	21	0	0.395	5.084465236	0.022826124
NovelmiRNA-385	19	0.875	0	-5.249953582	0.022997027
NovelmiRNA-1554	18	0	1.3625	5.185799488	0.02484975
NovelmiRNA-306	23	0.3075	0	-5.069616763	0.025748698
all_ssc-miR-132	23	87.3175	243.2525	1.139537289	0.026410333
NovelmiRNA-1437	18	0	0.5225	5.035519602	0.026496002
all_ssc-miR-652	22	49.3575	136.9	1.089804197	0.027052367
NovelmiRNA-939	21	0	0.505	5.014899521	0.027192784
NovelmiRNA-625	21	0	0.36	4.931013064	0.028448005
all_ssc-miR-135	22	64.0475	24.425	-1.754890611	0.029752227
NovelmiRNA-1608	21	0	0.46	4.93661947	0.030200089
NovelmiRNA-121	21	0.2875	0	-4.931714006	0.031039914
all_ssc-miR-9	23	48.715	26.52	-1.284255991	0.031055878
NovelmiRNA-217	22	0	0.305	4.800062756	0.033860797
NovelmiRNA-1118	19	74.27	200.26	1.081457372	0.034006381
NovelmiRNA-1417	19	0.2475	0	-4.835781866	0.035127358
NovelmiRNA-662	21	0	0.415	4.815835785	0.035373858

NovelmiRNA-544	22	0.1975	0	-4.772040644	0.038104553
NovelmiRNA-1026	18	0	0.39	4.738474991	0.038939611
NovelmiRNA-1235	24	0	0.3725	4.730763746	0.039357873
NovelmiRNA-1382	23	0.2025	0	-4.743112226	0.039452966
NovelmiRNA-498	18	0	0.38	4.706037011	0.040554064
all_ssc-miR-150	22	64.5125	33.715	-1.321285225	0.041524343
NovelmiRNA-324	21	36.8975	20.95	-1.296553416	0.042109734
NovelmiRNA-962	19	0	0.37	4.665152401	0.042669524
NovelmiRNA-1376	22	0.2175	0	-4.656651715	0.043865925
NovelmiRNA-1192	23	0.19	0	-4.654729353	0.04395348
NovelmiRNA-1302	22	0.2575	3.8125	3.240277797	0.044332453
NovelmiRNA-560	18	0.79	23.64	3.823631195	0.045578121
NovelmiRNA-219	22	0	0.2525	4.561730071	0.045894848
NovelmiRNA-1518	21	0	0.345	4.602757454	0.046111627
NovelmiRNA-523	21	0.1625	0	-4.572356907	0.0463111
NovelmiRNA-776	23	0.2275	0	-4.60170175	0.046891578
NovelmiRNA-930	21	0.135	0	-4.554852378	0.047000808
NovelmiRNA-1677	22	0.18	0	-4.57615619	0.048362108
all_ssc-miR-1249	22	11.2875	46.095	1.677809666	0.049035297
NovelmiRNA-1154	25	0	0.33	4.546188377	0.049237302
NovelmiRNA-546	19	0.17	0	-4.554118308	0.049570433

Table S5-2. Differential expression analysis of miRNAs in the FH vs. FL comparison.

gene_id	length	Value (FH)	Value (FL)	log2(FoldChange)	*P* value
NovelmiRNA-131	23	0	2.1525	6.730018154	1.34E-05
NovelmiRNA-664	22	1.8275	0	-6.433891113	4.14E-05
NovelmiRNA-1515	22	2.21	169.2975	5.122037043	5.50E-05
all_ssc-miR-190b	22	0.5925	36.195	4.859604665	0.00010341
NovelmiRNA-240	23	13.3675	587.6425	4.494760087	0.000194168
all_ssc-miR-122	21	15.8725	331.4275	3.899697545	0.000232244
all_ssc-miR-34c	21	102.34	4296.12	4.426550598	0.000260687
all_ssc-miR-885-5p	24	0.4825	16.65	4.19787369	0.000275567
all_ssc-miR-429	22	2.705	72.775	3.910163713	0.000849358
NovelmiRNA-337	22	16.8525	575.5125	4.076352944	0.001377303
NovelmiRNA-1528	22	16.8525	575.5125	4.076352944	0.001377303
all_ssc-miR-92b-5p	22	19.4175	242.955	3.117352025	0.001471977
NovelmiRNA-318	18	0.795	0	-5.207745344	0.001814769
NovelmiRNA-1123	18	0	1.025	4.959815371	0.003750405
NovelmiRNA-388	23	11.3425	459.6625	3.898764635	0.008740852
all_ssc-miR-194a	22	40.4325	217.8025	2.073673829	0.010883608
all_ssc-miR-20a	23	550.9475	228.31	-1.157984659	0.011914833
NovelmiRNA-1582	23	0.87	0	-4.208362814	0.017664246
NovelmiRNA-1277	22	0.5	0	-4.176446366	0.017796252
all_ssc-miR-215	20	3.03	16.09	2.055053209	0.01881474
all_ssc-miR-127	22	137.855	409.3975	1.442704154	0.023488054
NovelmiRNA-624	22	0.41	0	-3.942615719	0.026311734
NovelmiRNA-488	21	0.55	0	-3.927904557	0.02798525
NovelmiRNA-1043	22	0.6225	0	-3.853050968	0.031515099
NovelmiRNA-40	18	0.4825	0	-3.792475467	0.034526413
NovelmiRNA-619	18	0.59	5.7275	2.627486382	0.037044796
NovelmiRNA-823	18	0.59	5.7275	2.627486382	0.037044796
all_ssc-miR-671-5p	24	10.4025	35.525	1.573396982	0.037235183
NovelmiRNA-50	18	0	0.525	3.735736995	0.037688706
NovelmiRNA-150	21	11.105	176.6375	2.974965615	0.037886444
NovelmiRNA-626	23	29.18	0	-3.695582197	0.041818882
NovelmiRNA-931	23	29.18	0	-3.695582197	0.041818882
NovelmiRNA-522	25	0	20.045	3.688111934	0.042248119
NovelmiRNA-91	21	0.4325	0	-3.650891607	0.042618523
NovelmiRNA-1503	21	9.995	0	-3.662712252	0.043735925
NovelmiRNA-482	18	0	0.515	3.602719691	0.045742158
NovelmiRNA-702	22	0	0.4475	3.598019192	0.045999253
NovelmiRNA-291	21	0	0.315	3.583586479	0.046011506
NovelmiRNA-1412	18	0	0.5075	3.596768553	0.046103456
NovelmiRNA-4	22	0	5.5625	3.612448256	0.046807095
NovelmiRNA-1177	18	0	0.495	3.568784702	0.047979755
NovelmiRNA-936	21	0	4.5775	3.593765806	0.047993227
NovelmiRNA-376	21	0	0.435	3.565588416	0.048201404
NovelmiRNA-634	21	0	0.435	3.565588416	0.048201404
NovelmiRNA-1474	21	0	0.435	3.565588416	0.048201404
NovelmiRNA-1179	21	0.2675	0	-3.528487869	0.049721164

Table S6-1. Identification of circRNA – miRNA pairs in the LH vs. LL comparison.

miRNA_ID	circRNA_ID	Max Score	Max Energy	Positions	cor	*P* value
ssc-miR-150	circRNA_004920	164	-30.72	25721	0.274206006	0.551794715
ssc-miR-150	circRNA_002275	170	-31.13	198264	-0.484045135	0.271041425
ssc-miR-150	circRNA_000812	166	-30.18	25497	-0.674578049	0.096457231
ssc-miR-150	circRNA_006492	170	-31.13	162073	0.54817722	0.202658857
ssc-miR-150	circRNA_003448	172	-31.23	25722	-0.243799843	0.598302471
ssc-miR-150	circRNA_005086	162	-31.82	69968	-0.375150864	0.406977838
ssc-miR-150	circRNA_005498	174	-35.27	64798	0.478533335	0.277343572
ssc-miR-504	circRNA_001243	165	-32.49	131191	-0.560411399	0.190683476
ssc-miR-504	circRNA_000875	178	-30.34	25854	-0.411401405	0.359154838
ssc-miR-504	circRNA_004984	176	-30.12	28796	-0.175473907	0.70667122
ssc-miR-504	circRNA_001214	165	-32.36	100713 231381	-0.411309028	0.359273591
ssc-miR-504	circRNA_001544	164	-32.58	136663	-0.264976858	0.565785776
ssc-miR-504	circRNA_000629	175	-34.28	116773	-0.645528003	0.117363517
ssc-miR-361-3p	circRNA_002590	166	-30.24	158141	-0.36995792	0.414023463
ssc-miR-361-3p	circRNA_004498	153	-31.59	21196	-0.36995792	0.414023463
ssc-miR-361-3p	circRNA_004329	160	-30.79	6613 10109	-0.390843932	0.385978467
ssc-miR-361-3p	circRNA_003395	163	-30.44	65407	-0.351434254	0.439536393
ssc-miR-361-3p	circRNA_001382	165	-35.29	197925 103455	-0.538901202	0.211971828
ssc-miR-361-3p	circRNA_001638	175	-31.96	20440	-0.399402633	0.374715001
ssc-miR-361-3p	circRNA_004920	161	-30.61	12152	-0.511043818	0.241127584
ssc-miR-361-3p	circRNA_004878	168	-33.06	18715	-0.340295466	0.455156485
ssc-miR-361-3p	circRNA_002275	164	-31.94	200008	-0.402763771	0.370328884
ssc-miR-361-3p	circRNA_004939	160	-30.36	9170	-0.371905094	0.411376036
ssc-miR-361-3p	circRNA_006052	161	-34.85	3376	-0.368045025	0.416630746
ssc-miR-361-3p	circRNA_003253	171	-32.46	135719 16060	-0.307381154	0.502472035
ssc-miR-361-3p	circRNA_000812	165	-33	56458	-0.390843932	0.385978467
ssc-miR-361-3p	circRNA_000731	165	-31.12	228498 342636	-0.351088272	0.44001847
ssc-miR-361-3p	circRNA_002589	166	-30.24	158141	-0.393829885	0.382033511
ssc-miR-361-3p	circRNA_002453	170	-35.6	12461	-0.788660361	0.035065336
ssc-miR-361-3p	circRNA_002150	162	-34.36	2104	-0.524304431	0.227028762
ssc-miR-361-3p	circRNA_004909	166	-32.26	8821	-0.383809965	0.395335928
ssc-miR-361-3p	circRNA_006492	164	-31.94	163817	-0.390843932	0.385978467
ssc-miR-361-3p	circRNA_005471	159	-30.8	674	-0.392698793	0.383525949
ssc-miR-361-3p	circRNA_003448	162	-31.29	99006 87829	-0.391243763	0.385449272
ssc-miR-361-3p	circRNA_002486	160	-30.71	38249	-0.393829885	0.382033511
ssc-miR-361-3p	circRNA_004767	175	-31.22	9425	-0.32431547	0.477917117
ssc-miR-361-3p	circRNA_000709	163	-33.81	12114	-0.53076481	0.220304471
ssc-miR-361-3p	circRNA_002158	157	-31.02	4915	-0.399402633	0.374715001
ssc-miR-361-3p	circRNA_002149	162	-34.36	2104	-0.697518788	0.081458678
ssc-miR-361-3p	circRNA_001209	173	-30.32	6496	-0.390843932	0.385978467
ssc-miR-361-3p	circRNA_000021	151	-34.08	13966	-0.35812393	0.430254919
ssc-miR-361-3p	circRNA_001214	166	-34.97	293328 70451 234009	-0.618675291	0.138572887
ssc-miR-361-3p	circRNA_004225	175	-37.21	97167 87115 85057	-0.39955771	0.374512169
ssc-miR-361-3p	circRNA_004384	165	-34.06	3724	-0.399686374	0.374343917
ssc-miR-204	circRNA_002469	174	-34.88	6244	0.15146743	0.745800417
ssc-miR-652	circRNA_011886	169	-33.26	58615	-0.391790834	0.384725674
ssc-miR-652	circRNA_006995	166	-31.25	17010	-0.452753099	0.307674927
ssc-miR-652	circRNA_007786	162	-30.31	17274	-0.682309744	0.091252862
ssc-miR-652	circRNA_010607	173	-30.71	38759	-0.642314885	0.119806472

ssc-miR-652	circRNA_010856	176	-30.01	45679	-0.642314885	0.119806472
ssc-miR-652	circRNA_007851	170	-33.98	151076	-0.164766251	0.724065464
ssc-miR-652	circRNA_006593	151	-31	65145	-0.455571605	0.304291304
ssc-miR-652	circRNA_010551	179	-31.15	257508	-0.642314885	0.119806472
ssc-miR-652	circRNA_010887	175	-30.42	51510	-0.813555878	0.026002176
ssc-miR-652	circRNA_007180	168	-31.57	44324	-0.265494079	0.564998721
ssc-miR-652	circRNA_006801	175	-31.85	152	-0.59425161	0.159414073
ssc-miR-185	circRNA_010345	165	-30.37	149719	-0.493669797	0.260194103
ssc-miR-185	circRNA_007338	184	-30.16	26664	-0.224122491	0.629001383
ssc-miR-185	circRNA_007851	183	-30.86	41360	-0.224122491	0.629001383
ssc-miR-185	circRNA_006875	176	-31.75	16655	-0.577632938	0.1744267
ssc-miR-1343	circRNA_007914	162	-31.93	38268	-0.525363189	0.225920231
ssc-miR-1343	circRNA_011886	163	-32.53	51172	-0.475556589	0.280774229
ssc-miR-1343	circRNA_010345	164	-35.08	68445	-0.460163786	0.298813557
ssc-miR-1343	circRNA_006611	158	-34.13	15504	-0.479933241	0.27573675
ssc-miR-1343	circRNA_007127	163	-32.58	93039 110118	-0.686389149	0.088568113
ssc-miR-1343	circRNA_006795	163	-32.68	1933 5462	-0.519150821	0.232460984
ssc-miR-1343	circRNA_011662	166	-30.04	2571	-0.475556589	0.280774229
ssc-miR-1343	circRNA_010609	158	-30.63	141647 90564	-0.722898577	0.066425879
ssc-miR-1343	circRNA_007974	167	-37.42	40694 51072 23657	-0.48803667	0.26651842
ssc-miR-1343	circRNA_007623	161	-30.78	112801 58722	-0.451651613	0.309001706
ssc-miR-1343	circRNA_006847	171	-33.51	5680	-0.152631791	0.743891888
ssc-miR-1343	circRNA_011701	158	-30.74	6009	-0.475556589	0.280774229
ssc-miR-1343	circRNA_010500	170	-35.68	160905	-0.460163786	0.298813557
ssc-miR-1343	circRNA_006702	157	-30.02	5935	-0.375093877	0.407054898
ssc-miR-1343	circRNA_011870	171	-34.7	104295 37029	-0.475556589	0.280774229
ssc-miR-1343	circRNA_010856	171	-34.94	56720	-0.460163786	0.298813557
ssc-miR-1343	circRNA_010632	171	-33.49	54160 5271	-0.460163786	0.298813557
ssc-miR-1343	circRNA_007851	165	-30.09	75450 28682	-0.152631791	0.743891888
ssc-miR-1343	circRNA_001856	159	-32.78	45235 46649	-0.709886761	0.073928084
ssc-miR-1343	circRNA_008244	150	-30.18	13044	-0.515211668	0.236653583
ssc-miR-1343	circRNA_007893	158	-34.01	787 825	-0.375093877	0.407054898
ssc-miR-1343	circRNA_006593	167	-37.43	66430 26062 35928 51394 17524 16536	-0.530221932	0.220865856
ssc-miR-1343	circRNA_011455	169	-36.93	23595 51703	-0.475556589	0.280774229
ssc-miR-1343	circRNA_007082	164	-33.94	168919 58577	-0.721786071	0.067050469
ssc-miR-1343	circRNA_010471	151	-33.85	1331	-0.677959616	0.094162356
ssc-miR-1343	circRNA_007122	163	-32.02	3324	-0.152631791	0.743891888
ssc-miR-1343	circRNA_006859	166	-33.85	3043	-0.152631791	0.743891888
ssc-miR-1343	circRNA_010551	164	-34.51	201599 165986 80623	-0.460163786	0.298813557
ssc-miR-1343	circRNA_011228	152	-31.85	12526	-0.475556589	0.280774229
ssc-miR-1343	circRNA_010435	153	-31.47	248600	-0.460163786	0.298813557
ssc-miR-1343	circRNA_011513	163	-30.46	3734	-0.475556589	0.280774229
ssc-miR-1343	circRNA_006954	168	-37.9	21564 40113	-0.396977602	0.377892637
ssc-miR-1343	circRNA_007539	158	-30.63	141647 90564	-0.501584844	0.251425226
ssc-miR-1343	circRNA_010887	170	-32.38	101428	-0.689038637	0.086847085
ssc-miR-1343	circRNA_007180	161	-32.36	25448	-0.274394256	0.551510513
ssc-miR-1343	circRNA_010899	172	-33.6	55817	-0.67827626	0.093948953
ssc-miR-1343	circRNA_011871	171	-34.7	104289 37023	-0.475556589	0.280774229
ssc-miR-1343	circRNA_010820	159	-30.57	44904	-0.63356695	0.126588643
ssc-miR-1343	circRNA_007459	160	-38.99	12527 43466	-0.527892001	0.223282873
ssc-miR-1343	circRNA_007778	157	-30.25	10371	-0.501584844	0.251425226
ssc-miR-1343	circRNA_010422	167	-38.35	113211 148494	-0.460163786	0.298813557

ssc-miR-1343	circRNA_010257	159	-30.98	3470	-0.715855388	0.070433256
ssc-miR-1343	circRNA_011873	161	-31.83	3904	-0.475556589	0.280774229
ssc-miR-1343	circRNA_010744	170	-35.38	6145 56002 17137 53125 74883	-0.460163786	0.298813557
ssc-miR-1343	circRNA_010722	179	-33.17	35504	-0.460163786	0.298813557
ssc-miR-1343	circRNA_010879	161	-30.85	28995	-0.460163786	0.298813557
ssc-miR-1307	circRNA_011886	166	-32.17	73710	-0.5576278	0.193377252
ssc-miR-1307	circRNA_010345	163	-31.64	61293	-0.438385893	0.325174997
ssc-miR-1307	circRNA_006858	153	-30.41	6549	-0.046252417	0.92156342
ssc-miR-1307	circRNA_008193	161	-30.04	2932	-0.511604098	0.240523883
ssc-miR-1307	circRNA_010551	161	-30.02	50904	-0.438385893	0.325174997
ssc-miR-1307	circRNA_006954	155	-32.81	17897	-0.340797114	0.454448625
ssc-miR-1307	circRNA_010950	166	-33.17	47889 29052	-0.5576278	0.193377252
ssc-miR-1307	circRNA_007459	161	-30.54	24927	-0.436778463	0.327158891
ssc-miR-1307	circRNA_007952	164	-30.29	8894	-0.046252417	0.92156342
ssc-miR-362	circRNA_010887	174	-30.43	12605	-0.836109922	0.01908093
ssc-miR-132	circRNA_010887	184	-32.66	43310	-0.83543069	0.019271816
ssc-miR-542-5p	circRNA_010500	161	-31.14	5383	-0.513103341	0.238911908
ssc-miR-542-5p	circRNA_010300	162	-33.72	17591	-0.533776392	0.217202496
ssc-miR-542-5p	circRNA_007082	160	-30.6	70187	-0.69205328	0.084910577
ssc-miR-542-5p	circRNA_007597	158	-30.16	36908	-0.272871033	0.553811494
ssc-miR-542-5p	circRNA_008236	159	-30.05	6335	-0.675574067	0.095778276
ssc-miR-184	circRNA_006901	158	-31.76	3740	-0.445317503	0.316679557
ssc-miR-184	circRNA_007778	167	-31.14	8191	-0.487809079	0.266775387
ssc-miR-1249	circRNA_006593	168	-32.21	75659	-0.414369448	0.35534801
ssc-miR-1249	circRNA_006665	178	-33.27	15401	-0.687412338	0.087901365
ssc-miR-1249	circRNA_010551	170	-30.37	106029	-0.437066305	0.326803254
ssc-miR-1249	circRNA_006954	176	-32.43	50730	-0.243248097	0.599157069

Table S6-2. Identification of circRNA – miRNA pairs in the FH vs. FL comparison.

miRNA_ID	circRNA_ID	Max Score	Max Energy	Positions	cor	*P* value
ssc-miR-20a	circRNA_014944	187	-33.57	183846	-0.556358799	0.194611369
ssc-miR-122	circRNA_006029	181	-34.03	11844	-0.421744387	0.34596233
ssc-miR-190b	circRNA_014668	192	-31.26	5708	-0.192798002	0.678744997
ssc-miR-127	circRNA_000999	156	-31.51	38871	-0.419922068	0.348271698
ssc-miR-127	circRNA_014716	163	-30.88	1767	-0.022023268	0.962621205
ssc-miR-127	circRNA_006574	156	-31.51	25362	-0.275297723	0.550147208
ssc-miR-127	circRNA_011381	156	-31.67	120499	-0.275297723	0.550147208
ssc-miR-127	circRNA_014899	156	-31.51	21992	-0.434664058	0.329776366
ssc-miR-127	circRNA_014801	156	-31.51	476	-0.397661251	0.376995712
ssc-miR-127	circRNA_010132	156	-31.51	25362	-0.21655214	0.640928672
ssc-miR-127	circRNA_005684	157	-31.33	111236	-0.393128031	0.382959306
ssc-miR-127	circRNA_009176	152	-30.1	9223	-0.275297723	0.550147208
ssc-miR-127	circRNA_014792	151	-31.41	9583	-0.286178839	0.5338153
ssc-miR-127	circRNA_014687	156	-30.7	25931	-0.022023268	0.962621205
ssc-miR-127	circRNA_001874	156	-30.42	44900	-0.358524934	0.429700962
ssc-miR-127	circRNA_014657	164	-31.51	162332	-0.282454527	0.539386948
ssc-miR-127	circRNA_007852	156	-30.42	44900	-0.261249534	0.571467888
ssc-miR-127	circRNA_002197	164	-33.24	55513 15527	-0.275297723	0.550147208
ssc-miR-127	circRNA_012635	160	-30.22	2997	-0.434664058	0.329776366
ssc-miR-127	circRNA_002521	151	-31.95	39319	-0.275297723	0.550147208
ssc-miR-127	circRNA_010615	162	-30.2	27684	-0.275297723	0.550147208
ssc-miR-127	circRNA_007235	151	-31.05	942	-0.425895762	0.340725605
ssc-miR-127	circRNA_004985	151	-32.37	21151	-0.275297723	0.550147208
ssc-miR-127	circRNA_010284	159	-31.51	56401	-0.286178839	0.5338153
ssc-miR-127	circRNA_014644	160	-31.35	67156	-0.21655214	0.640928672
ssc-miR-127	circRNA_014879	156	-31.51	4782	-0.286178839	0.5338153
ssc-miR-127	circRNA_005982	151	-31.41	8481	-0.275297723	0.550147208
ssc-miR-127	circRNA_014673	161	-31.89	231	-0.290531444	0.527328277
ssc-miR-127	circRNA_003336	160	-30.22	2997	-0.294709602	0.521126472
ssc-miR-127	circRNA_004126	156	-31.51	3571	-0.275297723	0.550147208
ssc-miR-127	circRNA_005571	156	-30.7	26247	-0.423077096	0.344277523
ssc-miR-127	circRNA_007438	151	-30.66	82858	-0.326927306	0.474169292
ssc-miR-127	circRNA_014007	164	-31.51	162332	-0.283775089	0.537409158
ssc-miR-127	circRNA_014652	155	-30.41	23219	-0.283037566	0.53851344
ssc-miR-127	circRNA_010510	151	-33.67	24162	-0.022023268	0.962621205
ssc-miR-127	circRNA_014823	151	-31.41	8275	-0.286178839	0.5338153
ssc-miR-92b-5p	circRNA_009914	169	-35.42	35626	-0.213366214	0.645966749
ssc-miR-92b-5p	circRNA_011381	174	-31.82	170562 170527	-0.20020496	0.666891862
ssc-miR-92b-5p	circRNA_015019	173	-37.26	37261	-0.20020496	0.666891862
ssc-miR-92b-5p	circRNA_013983	163	-30.82	28514 14281	-0.285871851	0.534273831
ssc-miR-92b-5p	circRNA_003230	166	-34.08	8788	-0.20020496	0.666891862
ssc-miR-92b-5p	circRNA_014812	162	-30.04	111683	-0.217797225	0.638962713
ssc-miR-92b-5p	circRNA_001874	160	-33.34	49013	-0.434866389	0.329525508
ssc-miR-92b-5p	circRNA_004045	156	-31.41	9366	-0.213366214	0.645966749
ssc-miR-92b-5p	circRNA_014659	157	-30.02	914	-0.333764338	0.464409587
ssc-miR-92b-5p	circRNA_004044	156	-31.41	16625	-0.31052789	0.497876296
ssc-miR-92b-5p	circRNA_004712	155	-31.63	28490	-0.319623199	0.484676964
ssc-miR-92b-5p	circRNA_014998	174	-31.82	8348 8313	-0.20020496	0.666891862
ssc-miR-92b-5p	circRNA_005878	171	-33.64	72464	-0.20020496	0.666891862

ssc-miR-92b-5p	circRNA_014698	164	-33.76	2681	-0.268328088	0.560692426
ssc-miR-92b-5p	circRNA_007852	160	-33.34	49013	-0.31052789	0.497876296
ssc-miR-92b-5p	circRNA_002197	173	-38.4	34315	-0.20020496	0.666891862
ssc-miR-92b-5p	circRNA_004716	170	-35.56	17681	-0.281205222	0.541260233
ssc-miR-92b-5p	circRNA_003129	163	-32.23	3887	-0.314337781	0.492331966
ssc-miR-92b-5p	circRNA_014644	159	-32.08	183836	-0.320036374	0.484080359
ssc-miR-92b-5p	circRNA_012655	163	-30.21	3578	-0.327230934	0.473734299
ssc-miR-92b-5p	circRNA_014683	166	-30.46	48962	-0.213366214	0.645966749
ssc-miR-92b-5p	circRNA_008647	167	-32.11	11167	-0.294848518	0.520920703
ssc-miR-92b-5p	circRNA_001063	167	-31.18	16434	-0.319059016	0.485492041
ssc-miR-92b-5p	circRNA_000441	157	-30.97	471	-0.317846568	0.487245326
ssc-miR-92b-5p	circRNA_014856	159	-34.24	1984	-0.217797225	0.638962713
ssc-miR-671-5p	circRNA_002942	171	-31.58	13589	-0.135935305	0.771355276
ssc-miR-671-5p	circRNA_009914	166	-38.05	31950 17623 15994 35709	-0.135935305	0.771355276
ssc-miR-671-5p	circRNA_014614	165	-33.07	16991	-0.135935305	0.771355276
ssc-miR-671-5p	circRNA_000016	173	-37.06	12671 108450 1522 100084 1500	-0.326034021	0.475449896
ssc-miR-671-5p	circRNA_014802	165	-35.46	73142	-0.405162229	0.367211976
ssc-miR-671-5p	circRNA_002098	173	-36.14	34404	-0.251579639	0.586291747
ssc-miR-671-5p	circRNA_006157	162	-31.47	2369	-0.284972807	0.535617439
ssc-miR-671-5p	circRNA_000612	167	-32.56	13374	-0.458553127	0.30072984
ssc-miR-671-5p	circRNA_013983	162	-31.81	7298	-0.336433472	0.460619714
ssc-miR-671-5p	circRNA_014687	156	-30.63	21728	-0.135935305	0.771355276
ssc-miR-671-5p	circRNA_014812	155	-34.98	193719	-0.307564465	0.502203907
ssc-miR-671-5p	circRNA_006112	177	-37.07	51004 17375 17815	-0.427417442	0.338814549
ssc-miR-671-5p	circRNA_001215	173	-41.03	181114 184367 3855 46310 44402 115052	-0.397218242	0.377576826
ssc-miR-671-5p	circRNA_003654	164	-35.31	3049	-0.328811847	0.471471753
ssc-miR-671-5p	circRNA_009125	157	-30.14	4865	-0.43613442	0.327955222
ssc-miR-671-5p	circRNA_001874	164	-42.48	42340	-0.474880152	0.281556448
ssc-miR-671-5p	circRNA_006633	169	-38.48	78915	-0.348286186	0.443930147
ssc-miR-671-5p	circRNA_012966	155	-30.94	21094	-0.359129367	0.428866502
ssc-miR-671-5p	circRNA_007473	166	-31.39	7042	-0.284972807	0.535617439
ssc-miR-671-5p	circRNA_001648	159	-34.84	3926 1155	-0.284972807	0.535617439
ssc-miR-671-5p	circRNA_012191	181	-39.74	30656	-0.431037947	0.33428595
ssc-miR-671-5p	circRNA_004712	164	-32.7	4122 24609	-0.314734618	0.491755736
ssc-miR-671-5p	circRNA_005910	169	-35.58	31795	-0.135935305	0.771355276
ssc-miR-671-5p	circRNA_014771	159	-32.7	4864	-0.307564465	0.502203907
ssc-miR-671-5p	circRNA_014901	176	-36.46	1433	-0.38316129	0.396203393
ssc-miR-671-5p	circRNA_007852	164	-42.48	42340	-0.339534304	0.456231319
ssc-miR-671-5p	circRNA_002197	164	-32.27	7460 165609	-0.284972807	0.535617439
ssc-miR-671-5p	circRNA_000752	162	-36.03	2682	-0.135935305	0.771355276
ssc-miR-671-5p	circRNA_014731	161	-30.39	17822	-0.30632993	0.504010618
ssc-miR-671-5p	circRNA_005526	162	-30.96	291	-0.350505693	0.440830664
ssc-miR-671-5p	circRNA_014742	175	-37.52	21116 10034 114302	-0.339094225	0.456853184
ssc-miR-671-5p	circRNA_010615	173	-34.12	211829 77965 165799 223117	-0.284972807	0.535617439
ssc-miR-671-5p	circRNA_013989	165	-31.65	21885	-0.340169939	0.455333676
ssc-miR-671-5p	circRNA_014788	156	-37.64	2678	-0.307564465	0.502203907
ssc-miR-671-5p	circRNA_014798	176	-36.33	12408 11415	-0.307564465	0.502203907
ssc-miR-671-5p	circRNA_014883	173	-33.33	37282 2443 29865	-0.43613442	0.327955222
ssc-miR-671-5p	circRNA_011041	162	-35.21	2667	-0.135935305	0.771355276
ssc-miR-671-5p	circRNA_007869	169	-30.53	10202	-0.284972807	0.535617439
ssc-miR-671-5p	circRNA_014644	168	-42.46	184161	-0.317351972	0.487961196
ssc-miR-671-5p	circRNA_006223	176	-42.25	6277 15815 1551	-0.307564465	0.502203907

ssc-miR-671-5p	circRNA_007298	175	-35.59	8905 4597	-0.326034021	0.475449896
ssc-miR-671-5p	circRNA_014683	164	-34.15	37957 5831 6642	-0.135935305	0.771355276
ssc-miR-671-5p	circRNA_008313	161	-31.39	80967	-0.511614109	0.240513102
ssc-miR-671-5p	circRNA_004461	171	-32.24	8997	-0.43613442	0.327955222
ssc-miR-671-5p	circRNA_015024	172	-36.5	1135	-0.284972807	0.535617439
ssc-miR-671-5p	circRNA_007438	157	-36.1	817	-0.447558114	0.313954258
ssc-miR-671-5p	circRNA_014048	181	-38.01	455	-0.43613442	0.327955222
ssc-miR-671-5p	circRNA_000441	152	-31.29	1077	-0.334214796	0.463769185
ssc-miR-671-5p	circRNA_014652	169	-31.58	136807 66526	-0.328811847	0.471471753
ssc-miR-671-5p	circRNA_009564	171	-32.24	8997	-0.492031861	0.262025886
ssc-miR-671-5p	circRNA_014721	160	-30.27	3287	-0.135935305	0.771355276
ssc-miR-671-5p	circRNA_010510	155	-31.3	46761	-0.135935305	0.771355276
